# Drivers of spatio-temporal variation in mosquito submissions to the citizen science project ‘Mückenatlas’

**DOI:** 10.1038/s41598-020-80365-3

**Published:** 2021-01-14

**Authors:** Nadja Pernat, Helge Kampen, Florian Ruland, Jonathan M. Jeschke, Doreen Werner

**Affiliations:** 1 Leibniz Centre for Agricultural Landscape Research, Eberswalder Str. 84, 15374 Müncheberg, Germany; 2 Friedrich-Loeffler-Institut, Federal Research Institute for Animal Health, Südufer 10, 17493 Greifswald Insel - Riems, Germany; 3 Department of Biology, Chemistry, Pharmacy, Institute of Biology, Freie Universität Berlin, Königin-Luise-Str. 1-3, 14195 Berlin, Germany; 4 Leibniz Institute of Freshwater Ecology and Inland Fisheries (IGB), Müggelseedamm 310, 12587 Berlin, Germany; 5 Berlin-Brandenburg Institute of Advanced Biodiversity Research (BBIB), Königin-Luise-Str. 2-4, 14195 Berlin, Germany

**Keywords:** Biodiversity, Community ecology, Invasive species, Climate-change ecology

## Abstract

Intensified travel activities of humans and the ever growing global trade create opportunities of arthropod-borne disease agents and their vectors, such as mosquitoes, to establish in new regions. To update the knowledge of mosquito occurrence and distribution, a national mosquito monitoring programme was initiated in Germany in 2011, which has been complemented by a citizen science project, the ‘Mückenatlas’ since 2012. We analysed the ‘Mückenatlas’ dataset to (1) investigate causes of variation in submission numbers from the start of the project until 2017 and to (2) reveal biases induced by opportunistic data collection. Our results show that the temporal variation of submissions over the years is driven by fluctuating topicality of mosquito-borne diseases in the media and large-scale climate conditions. Hurdle models suggest a positive association of submission numbers with human population, catch location in the former political East Germany and the presence of water bodies, whereas precipitation and wind speed are negative predictors. We conclude that most anthropogenic and environmental effects on submission patterns are associated with the participants’ (recording) behaviour. Understanding how the citizen scientists’ behaviour shape opportunistic datasets help to take full advantage of the available information.

## Introduction

Mosquito-borne diseases pose an increasing threat to human and animal health worldwide. Human-mediated dispersal, for example by global trade or travelling, are the main factors for the introduction of non-indigenous species such as the Asian tiger mosquito *Aedes albopictus* or the yellow fever mosquito *Aedes aegypti*^1,2^. Due to their adaptability and potentially facilitated by global warming, both species have succeeded in establishing populations in new regions^3,4^, and are potential vectors of a range of pathogens such as dengue or chikungunya viruses^1^. Comprehensive, long-term data collection about the distribution and phenology of invasive as well as native species that are competent vectors of pathogens^5^ are required to prevent infections, to assess and decrease impacts on human and animal well-being and to predict how particular vectors will spread.

To collect and update data as a basis for risk assessments, the German government initiated a still ongoing nationwide mosquito monitoring programme in 2011, consisting of targeted field efforts by scientists and a complementary citizen science project. While developmental stages and adults have been actively collected by dipping and trapping throughout Germany since the start of the programme^6^, this kind of professional monitoring is limited by staff and funding and can only provide snapshots of mosquito populations in selected habitats. Moreover, lack of access to private properties and of data from the people’s immediate surroundings, where, for example, invasive species readily breed in artificial containers, hampers risk assessments. The importance of such data from densely populated areas is illustrated by the first West Nile virus infections in Berlin in 2019^7^. To enhance data collection and to complement active surveillance by scientists, the citizen science project ‘Mückenatlas’ (mosquito atlas) started in 2012. Contrary to the majority of biodiversity monitoring projects involving citizens and working with online recordings via a website or an app, for instance *eBird*^8^ and *iNaturalist* on a global or the Spanish *Mosquito Alert*^9^ and the Austrian *RoadKill*^10^ on a national scale, ‘Mückenatlas’ participants do not upload records or are involved in the identification of the reported specimens themselves. Instead, they catch and send physical samples that are then determined to species level by the project’s experts according to a standardised protocol^11^. In this way, the ‘Mückenatlas’ has the benefits of citizen science—large observation numbers and a large geographic scale—while taking a rather conservative approach to ensure data quality instead of controlling for data quality issues with tools such as crowdsourcing, external expert validation or in-process data vetting^8,9^.

Another advantage of opportunistic sampling by citizens is to increase the probability of detecting rare or unexpected events, such as the arrival of an invasive species or the return of a particularly rare taxon^12^. With regard to mapping biodiversity and detecting invasive species, the ‘Mückenatlas’ has already attested its efficiency: submissions revealed new populations of *Aedes japonicus* (Asian bush mosquito) and *Ae. albopictus*^11,13–17^, produced first records of *Ae. aegypti* and *Aedes koreicus* in Germany^18,19^, and led to the rediscovery of very rare species after decades without documentation^20,21^.

On the flipside, random collections from citizens result in opportunistic datasets incorporating observation bias caused by recorder activity^22^ that vary in intensity depending on project design. Consequently, only small, species-specific fractions of the growing ‘Mückenatlas’ dataset have been analysed so far, e. g. to describe particular species findings^23^, to investigate nuisance sources^24^ or for population genetics^25^. Kerkow et al.^26^, for example, mitigated bias by combining ‘Mückenatlas’ observations of *Ae. japonicus* with conventional monitoring data to predict species distribution, to some degree discussing patterns resulting from biases of both—in this case over- and underrepresentation of land-use types. Other case studies of observation patterns focus on intrinsic and extrinsic motivations of citizen scientists^27,28^, less commonly on general environmental or anthropogenic factors associated with volunteers’ recordings for large-scale citizen science projects^29,30^. Analysing these driving factors, however, would help utilise opportunistic data collections to full extent and design future citizen science projects.

The ‘Mückenatlas’ submissions have so far neither been explored nor evaluated from a citizen science perspective^31,32^. This study contributes to our knowledge about the complexity of submission patterns for long-term, large-scale citizen science projects with the particularity that, in contrast to other studies, there is no uncertainty concerning species identification. After the ‘Mückenatlas’ has been operating for more than seven years, we here aim at answering the following three questions: (1) Which trends and characteristics shape the opportunistic dataset? (2) Which factors drive the seasonal and annual variations in submission numbers? (3) Which factors drive the spatial distribution of submissions? These questions are tackled by a descriptive analysis of the dataset and by deploying hurdle models to test the association of several anthropogenic and environmental predictors with submission numbers.

## Methods

### ‘Mückenatlas’ workflow

Citizens are asked to catch mosquitoes in a closable container without physically damaging them, and to kill them by freezing for at least 24 h. A form that can be downloaded from the project’s website (http://www.mueckenatlas.com) and is also available at the project’s office must be completed with information about the catch. The participants then send their catch along with the submission form to the project’s laboratory where the sample is identified to species level morphologically^33,34^ or, in difficult cases, such as damaged specimens or cryptic species, genetically^35^. As a reward, every participant receives a personal letter or email including detailed information about the submitted mosquito(es). If desired, the participants also get a mark with their name or a pseudonym on the website’s collectors’ map. The data corresponding to the catch is uploaded to CULBASE, the German mosquito database.

### Data preparation

Data attributed with ‘MA’ (tag for ‘Mückenatlas’) were extracted from CULBASE on July 31, 2018, marking the end of the period of data entry for 2017. Each entry represents the submission of one or more specimen of one mosquito species from one location. We ignored specimen counts for this study as we focused on investigating submissions, irrespective of the exact number of specimens sent. The dataset consisted of a partly automatically generated suite of covariates composed of information from the submission form and database processing such as species, geo-coordinates, collection date and land-use types according to CORINE Land Cover data level 3^36^. Furthermore, the collection site description provided by the participants on the submission forms were categorised to a biotope variable. When information on the catch location was missing, the home addresses of the participants were taken as geo-coordinates by default, but no biotope category was assigned to the corresponding entries. Unclear site descriptions, such as ‘hedge’ or ‘path’ with geo-coordinates, as well as interpretable descriptions without geo-coordinates, such as ‘forest nearby home’, were verified manually by Google Maps. If the catch location could not be verified, biotope category was set invalid. In total, the resulting dataset comprised 21,768 submissions and 15 covariates (Supplementary Table S1). Explorative and descriptive analysis of the covariates to depict submissions and identify temporal trends were conducted with R packages *ggplot2*^37^, *treemap*^38^ and *summarytools*^39^ deploying R version 3.5.2^40^.

### Raster data

**Figure 1:**
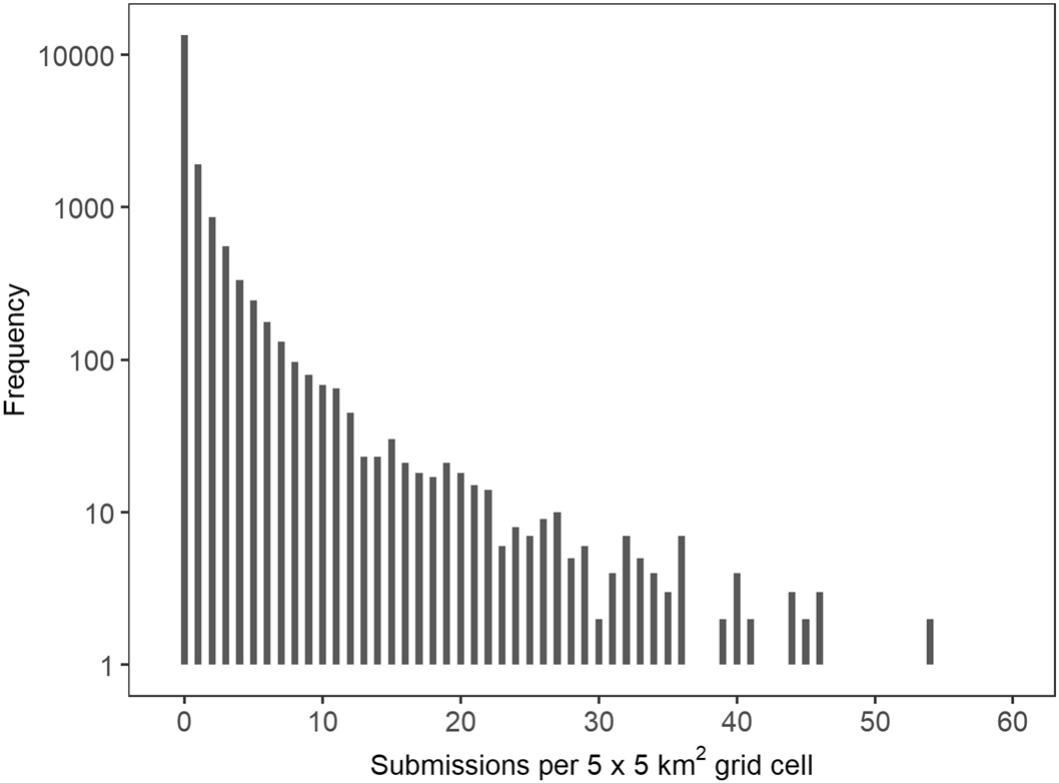
Histogram showing the numbers of grid cells on a log-transformed y-axis against the numbers of submissions per grid cell (limited to 60).

To implement submission numbers as response variable for statistical testing, we drew a raster grid with a 5 km resolution across Germany and counted submissions in every grid cell to create a submission-distribution raster file. Over 73% of the grid cells showed zero submissions, and the frequency distribution was highly skewed to the right (Fig. 1).

We selected four environmental and four anthropogenic predictors per grid cell a priori (Table 1, Supplementary Fig. S1), which were considered candidates to explain the variability of submission distribution and numbers. Predictors were integrated as spatial raster values according to the response variable’s raster extent. The variable ‘population’ was chosen because numerous previous studies in citizen science data have shown a strong positive relationship between total human population and the number of records^29,30^, and we expected the same for our data. The two variables ‘mean age’ and ‘proportion of women’ were used to test patterns in participant engagement according to demographic background. This selection builds on studies with partly contradictory results which have investigated the age structure and gender of citizen scientists^41–44^, giving us the idea of uncovering large-scale trends through spatial analysis rather than through sociological and selective participant surveys. As a fourth anthropogenic predictor, we created a binary variable that assigned the grid cells to former political East or West Germany in order to investigate whether the project headquarters’ locations—both based in federal states located in former East Germany—might affect the engagement of citizens and to test for general and large-scale differences in demographic structure and climatic conditions. We selected the environmental predictors that are generally important for the development and occurrence of the vast majority of mosquito species. These include precipitation, temperature, wind speed and natural water bodies that might be suitable for mosquito breeding^33^. For the latter we used open hydrographical data from a Web Feature Service (WFS)^45^ of a range of hydrological landscape features and included stagnant waters as well as floodplains and wetlands as areas presumably suitable for larval development. The preference for a binary variable over a numeric one or, in other words, whether or not there is a water body in the grid cell in favour of percentage coverage, was based on the assumption of a strong, positive correlation of the number of water bodies with submission numbers. Percentages would lead to meaningless associations due to the nature of the project, e.g. a 100% coverage of the grid cell with water would correlate with a maximum number of submissions, although no potential participants could live there. Wind speed turned out to be an interesting environmental factor in previous studies on predicting mosquito spread^26^, as it induces flight restriction and therefore decreases the probability of a mosquito to be caught. For example, *Ae. japonicus* has not yet been detected in German areas with an average wind speed > 4.7 m/s, based on results from both passive and active surveillance schemes^26^. Data on human population, mean age and proportion of women were derived from the German census in 2011^46^, while the German Weather Service^47^ (Deutscher Wetterdienst Climate Data Center) provided data on mean wind speed (1981–2000) as well as on mean temperature and precipitation for March to November, from 2012 to 2017, to describe the climatic conditions during the mosquito seasons.

**Table 1:** Set of variables pre-selected as predictors.

**Anthropogenic variable**	
Population (pop)	Human population in 2011 within each grid cell
Population age (age)	Mean age of human population within each grid cell in years
Proportion of women (fem)	Mean proportion of women within each grid cell in percent
Region (east)	Grid cell within the federal states located in former political East Germany (0 = no, 1 = yes)
**Environmental variable**	
Temperature (temp)	Mean temperature for March to November 2012 to 2017 in °C
Precipitation (preci)	Mean precipitation for March to November 2012 to 2017 in mm
Wind speed (wind)	Mean wind speed 10 m above ground 1981 to 2000 in m/s
Presence of water (water)	Standing water bodies, floodplains or wetlands in grid cell (0 = no, 1 = yes)

The predictors were grouped into anthropogenic and environmental factors. This allowed us to consider (1) the participants as a driving factor for submissions and (2) the effect of environmental variables on the occurrence of mosquitoes. However, this is a simplification of the ecological interactions of the underlying complex network, as every predictor might influence both participation behaviour and mosquito occurrence. For example, high wind speed might not only prevent mosquitoes from flying but also people from collecting mosquitoes outside, whereas a dense human population also provides a variety of habitats and hosts for container-breeding mosquitoes. Raster files of response variable and predictors were built with packages *sf*^48^, *leaflet*^49^, *raster*^50^, *rgdal*^51^ and *spatstat*^52^.

### Statistical analysis

Predictors were applied to fit hurdle models using either the probability of a zero count (binomial) or the number of submissions per grid cell (truncated negative binomial), accounting for overdispersion and excess zeros in the data. Multicollinearity was checked by calculating variance inflation factors (VIF) and, consequently, the predictors ‘mean age’ and ‘temperature’ with returned values > 5 were removed. The hurdle models allowed us to examine whether a certain set of predictors has an effect on (1) the probability of a non-zero count in the zero and (2) the number of submissions in the count part. All possible combinations of predictors were explored for both modelling parts using Automated Model Selection (AMS; command *dredge* in R package *MuMIn*) that ranks models by AIC and Akaike’s model weight. Models were selected and realised by the R packages *car*^53^*, countreg*^54^*, MuMIn*^55^, *MASS*^56^ and *pscl*^57^.

### Ethical approval

Ethical approval was not required because the collected data were anonymised, location data were aggregated and further processed without geo-referencing. The use of personal data complies with the EU General Data Protection Regulations; no personal sensitive information was obtained during this project or shared outside of the research team.

### Informed consent

Insect samples were provided voluntarily by citizen scientists after consent was given to the processing of the sender data according to EU General Data Protection Regulation.

## Results

### Characteristics of and temporal variation in submissions

**Figure 2:**
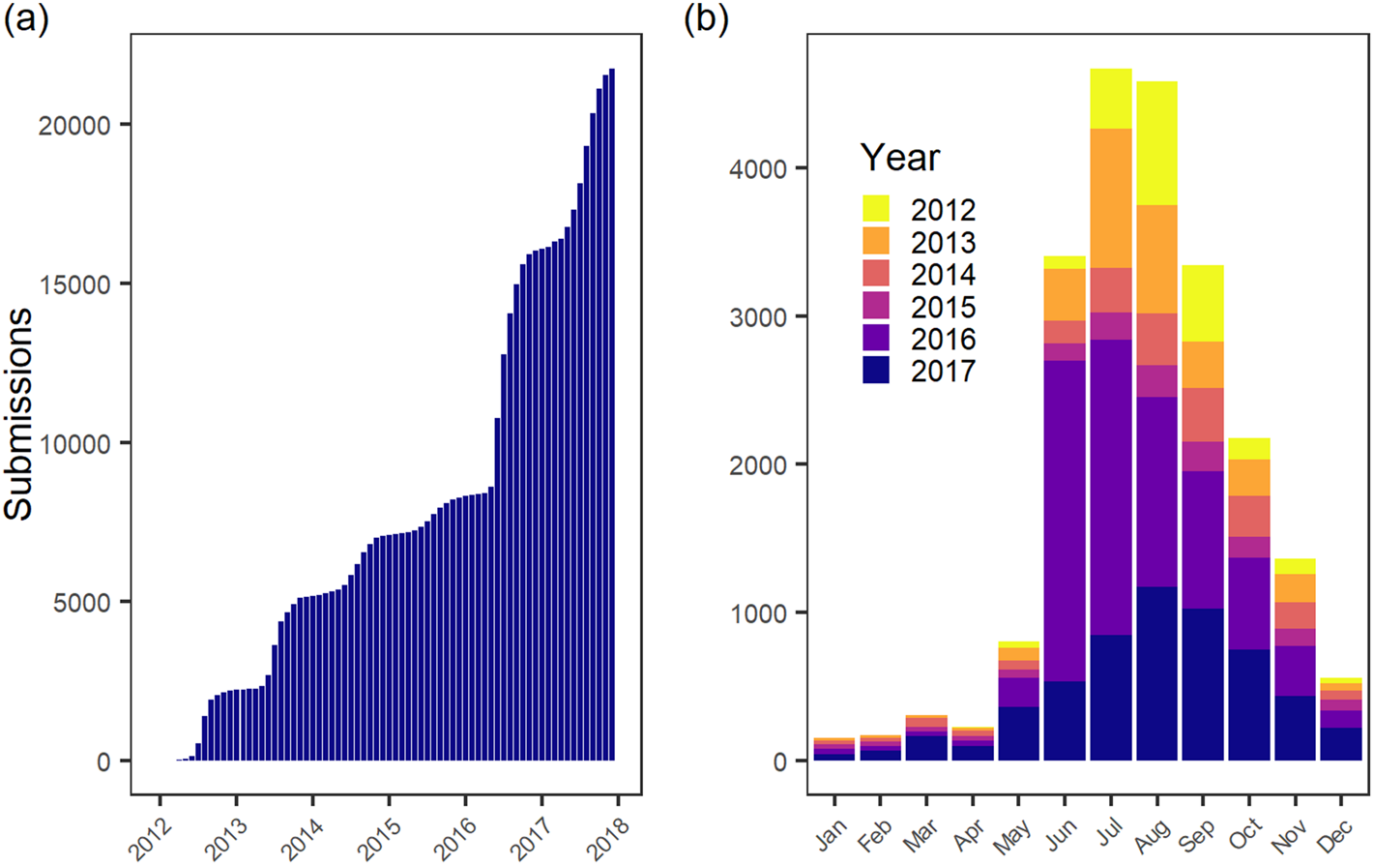
Temporal variation in submission numbers. (**a**) Cumulative submission numbers from 2012 to 2017. (**b**) Total number of submissions per month over all study years.

Between January 23, 2012 (first submission to ‘Mückenatlas’) and December 31, 2017, a total of 21,768 submissions from 11,277 locations (geo-coordinates) in 3221 municipalities were received. The number of specimens sent to the ‘Mückenatlas’ in the above time period adds up to 110,581; 3950 additional samples of arthropods submitted did not belong to the family *Culicidae* and were excluded from the study. Some submissions included several hundreds of specimens of the same species. This explains the difference between number of submissions recorded in the database and total species count, as we only created one CULBASE entry per species and submission, independently of the count which is recorded as a covariate. 2016 and 2017 represent the most productive years with 7756 (35.6% of all submissions from 2012–2017) and 5730 (26.3%) submissions, respectively, with June 2016 holding the monthly record of 2163 (9.9%) submissions (Fig. 2a). When submissions are added up according to month for the total observation period, most mosquitoes were submitted in July (4674, 21.5%), followed by August (4583, 21.1%), June (3405, 15.6%) and September (3341, 15.4%). The autumn months October and November still exhibit over 1000 submissions each, and are followed by a considerable decline during winter and spring, before submission numbers rise again in May (Fig. 2b).

**Figure 3:**
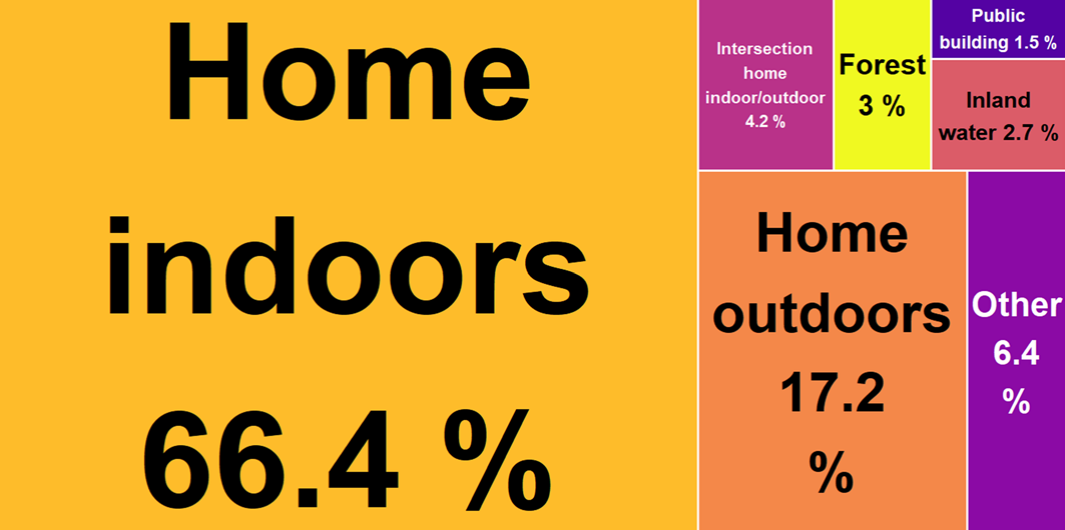
Proportional treemap of categorised participants’ biotope information showing the overrepresentation of submissions from private surroundings. Only biotopes with portions ≥ 1.5% are presented individually, categories with a smaller share have been combined into ‘other’. Plotted with package *treemap* in R version 3.5.2.

Almost every participant (92.9%, not applicable = 7.1%) provided information about the collection site, so that biotope descriptions could be considered during data analysis. According to the CORINE Land Cover Data level 3, most mosquitoes were caught in ‘discontinuous urban fabric’ (12,718, 58.4%), ‘non-irrigated arable land’ (3237, 14.9%) and ‘pastures’ (1247, 5.7%). In agreement, the most frequent specification of the collection sites as provided by the participants were ‘home indoors’ (13,305, 66.4%), followed by ‘home outdoors’ (e.g. garden, backyard, court; 3446, 17.2%) and ‘intersection home indoors/outdoors’ (e.g. house walls, windows, entrance doors; 839, 4.2%) (Fig. 3).

### Engagement hotspots in submission distribution

**Figure 4:**
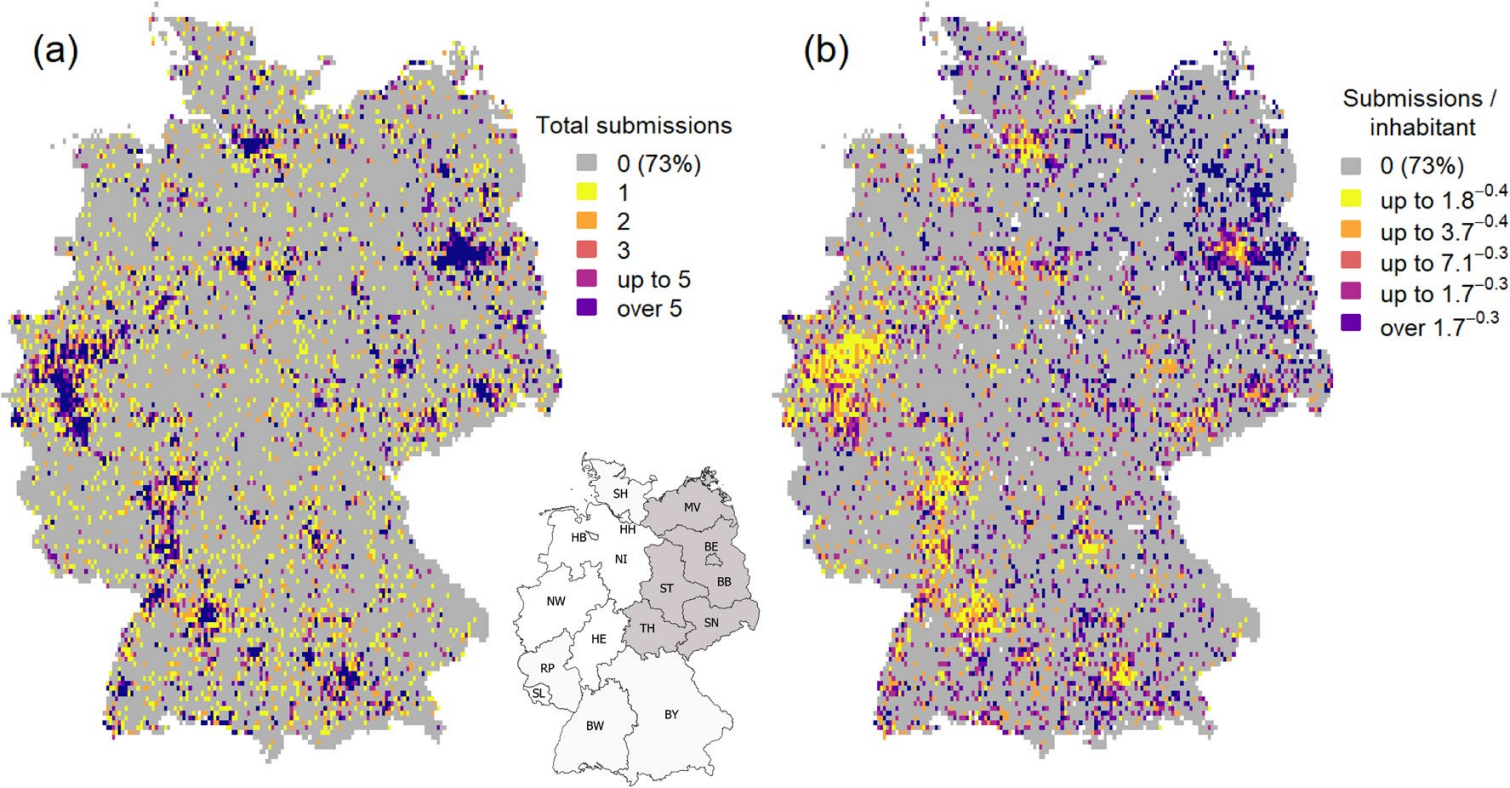
Federal states of Germany (*SH *Schleswig-Holstein, *HH *Hamburg, *HB *Bremen, *NI *Lower Saxony, *NW *North Rhine-Westphalia, *HE *Hesse, *RP *Rhineland Palatinate, *SL *Saarland, *BW *Baden-Wuerttemberg, *BY *Bavaria, *MV *Mecklenburg Western-Pomerania, *BB *Brandenburg, *BE* Berlin, *ST *Saxony-Anhalt, *SN *Saxony, *TH *Thuringia). (**a**) Raster grid of Germany (5 × 5 km^2^ cell size) of total submission numbers. (**b**) Raster grid of Germany (5 × 5 km^2^ cell size) showing per-capita submission rates (white grid cells = zero submission and zero/invalid population data). Both raster maps were created in R version 3.5.2., the German map outlining the federal states was drawn using QGis version 3.4.2. (Quantum GIS Geographic Information System, Open Source Geospatial Foundation Project. http://www.qgis.org/en/site/).

We further investigated the effect of ‘population’, as the map of total submission numbers strongly resembles a German human population map (Fig. 4a), with grid cells in densely populated areas, such as Berlin, Hamburg, Munich, the Ruhrgebiet and the Main-Neckar area displaying extremely high submission numbers. To disclose regions with high engagement independently of human population, we calculated a raster with per-capita submission rate, defined as number of submissions per person, based on the total human population in the respective grid cell. The resulting map (Fig. 4b) exhibits a considerably higher per-capita submission rate for eastern and south-eastern regions with a core area around the capital of Berlin, whereas the western part of the country stands behind. Although ranking first in absolute submission numbers (3119, 14.3%), the federal state of North-Rhine Westphalia as the most densely populated German region results in a low per-capita submission rate. People from Brandenburg (3075, 14.1%) and Bavaria (2917, 13.4%), both less densely populated federal states, participate more often, with a peak of 250 submissions in one of the two project’s hometowns, Müncheberg in Brandenburg (population: 6783), located approximately 50 km east of Berlin. To check if these hotspots are due to urban dwellers making trips to these areas rather than local residents, an additional analysis with entries coming exclusively from the participants' homes or gardens was conducted. The corresponding map (Supplementary Fig. S2) shows that the hotspot density becomes weaker, indicating that indeed some participants seem to be travelling from urban to rural areas to catch mosquitoes. Nonetheless, the pattern of greater involvement in the above-mentioned residential areas remains.

### Distribution patterns by hurdle models applying anthropogenic and environmental predictors

The engagement hotspots suggest that the non-random distribution of submissions might also be caused by further anthropogenic and environmental variables, whose associations were therefore tested using hurdle models. A predictor was considered important when included in the four best ranked models based on the Automated Model Selection (AMS) with an accumulated AIC weight > 0.95 (Table 2). As expected from the previous analysis on engagement hotspots, the number of people living in a grid cell has an effect on both whether there is a submission and how many. Indeed, ‘population’ is included in both parts of the best 995 models (24.9%) calculated by AMS (Supplementary Table S2 for complete AMS output). ’Region’ (East or West Germany), ‘presence of water bodies’ as well as ‘wind speed’ were also meaningful predictors of both submission numbers and submission probability. The importance of proportion of women and precipitation differs for each part of the hurdle models. Precipitation negatively affects the number, but not the probability of submissions. Conversely, the proportion of women in a grid cell may increase the probability of a submission from that unit, but does not influence record numbers.

**Table 2:** Best ranked models according to Akaike’s model weight (cumulative AIC weight > 0.95). Each of the algebraic signs indicates a positive or negative association of predictors (pop = human population, east = binary, region of former political East or West Germany, water = binary, presences of stagnant water bodies, fem = proportion of women, preci = mean precipitation (March to Nov, 2012 to 2017), wind = mean wind speed (1981 to 2000) with submission numbers. In brackets: predictors not present in all of the best models.

Count hurdle model predictors (truncated negbin with log link)	Zero hurdle model predictors (binomial with logit link)	Delta-AIC	AIC weight
+ pop + east + water − preci − wind	+ pop + east + water + fem − wind	0	0.328
+ pop + east + water (+ fem) − preci − wind	+ pop + east + water + fem − wind	0.055	0.319
+ pop + east + water − preci − wind	+ pop + east + water + fem (+ preci) − wind	1.320	0.170
+ pop + east + water (+ fem) − preci − wind	+ pop + east + water + fem (+ preci) − wind	1.374	0.165

**Figure 5:**
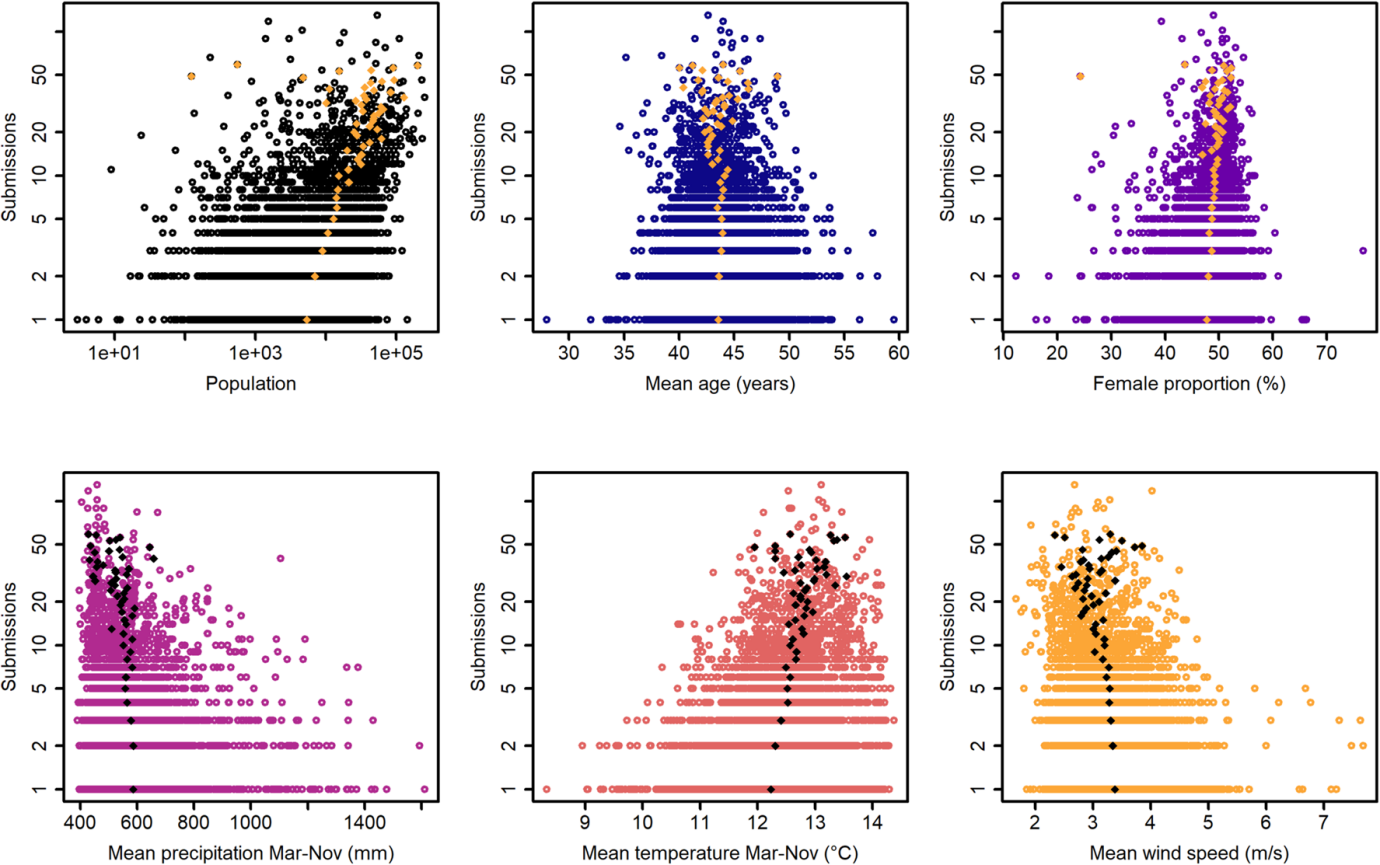
Scatterplots of the six non-factor predictors plotted against the number of submissions on a log-transformed y-axis. In the case of ‘population’, both axes are log-transformed. The yellow and black diamonds indicate average values per submission frequency.

To further explore relationships between predictors and response variables, we plotted the predictor values against the submission counts (Fig. 5). In accordance to model output, the resulting plots show a positive effect of human population and proportion of women on recordings, whereas increasing mean precipitation and mean wind speed per grid cell result in fewer submissions. The plots for mean age and mean temperature, both excluded from modelling due to multicollinearity, suggest a slightly and strongly positive association with submission numbers, respectively.

## Discussion

With the tremendous and continuous numbers of mosquito submissions received between 2012 and 2017, the ‘Mückenatlas’ is one of the most popular and successful citizen science projects in Germany. For the first time, we analysed the underlying dataset to characterise the origin and structure of the submissions, to reveal major spatial and temporal trends in submission numbers and to investigate what might drive these patterns.

The main characteristics of the data are the overrepresentation of indoor samples from the participants’ homes off densely populated areas. As shown in previous studies summarising popular citizen science projects focused on arthropods^58,59^, the ‘Mückenatlas’ participants seem to be genuinely interested in arthropods present in and around their homes. This interest is not confined to mosquitoes, as the participants’ messages on the submission forms indicate that the roughly 20% non-mosquito samples might be sent on purpose when people fail to identify a species they are curious about or suspect to be a pest. Such behaviour substantiates the high potential and success of large-scale and cross-taxonomic citizen science projects such as *iNaturalist* and supports the idea of a global community of citizen scientists recording species around their homes^60^.

It is of advantage to receive specimens found in the direct neighbourhood of people, as private properties are not directly accessible for scientists and at the same time highly important for research^60^. From a public health point of view submissions from people’s homes are of greater epidemiological relevance when addressing (arthropod-) vector-borne diseases^61^ than rare species in non-inhabited areas; the latter are more valuable for biodiversity research^31,62^. From a data science perspective, the over- and underrepresentation of certain land-use types create a spatial bias in the presence-only data to be considered when it comes to species distribution modelling^26,29,63^.

Our analysis to identify temporal trends revealed a noticeable variation in submission numbers over the years and months. The boost in submission numbers in 2016 may be the result of an increased public awareness of mosquito-borne diseases^64^, caused by an extensive flood of news about the South-American ZIKA virus epidemic, possibly triggering the maximum of recordings in June 2016 and sustained high numbers into 2017. We believe people became worried about mosquitoes in their living environment and—in a way of self-provisioning—approached the ‘Mückenatlas’ as an authority that could dissipate their concerns. In addition, the warm and humid weather in both 2016 and 2017 created beneficial conditions for many mosquito species in large parts of the country. This favourable climatic situation combined with the continuing public attention fuelled by the media may have kept the submission numbers high until the end of 2017. Temporal variation in citizens’ observation records can confirm^65^ or extend^66^ findings on the phenology of the target species. In our case, the records even reflect those mosquitoes that enter people’s homes in October and November to find an overwintering place, such as *Culiseta annulata*.

Based on the hurdle models we demonstrated how anthropogenic and environmental predictors relate to the spatial distribution of submissions. The positive correlation of human population with submission numbers was expected, especially since the visualisation revealed a spatial pattern of urban clusters known from other citizen science projects^29,30^. After controlling for the influence of human population, engagement hotspots became apparent in East Germany, predominantly in the sparsely populated federal states of Brandenburg and Mecklenburg-Western Pomerania^67^. This finding is corroborated by the models’ estimated positive association of the predictor ‘east’ with submission numbers. In other words, a location in former East Germany increases the number and probability of submissions. We attributed this trend to the project’s institutional homes in Brandenburg and Mecklenburg-Western Pomerania: frequent regional media coverage and participation in regional (science) events may create a *headquarter effect* that leads to a strong project support and identification by local communities. Newman et al.^68^ discovered that place-related effects play a considerable role in citizen scientists’ motivations to participate, especially in programmes using volunteer data for conservation decisions. This ‘power of place’^68^ may even present a stronger driver for participation than citizen concerns about invasive species: of the regions invaded by *Ae. japonicus* and *Ae. albopictus*, only the infested Southwest showed an increased engagement.

Considering the third investigated anthropogenic effect included in the models, a higher proportion of women per grid cell positively affects participation, but only the odds of a submission, not the number of submissions. However, it would be premature to conclude that women are more likely to contribute to the ‘Mückenatlas’. Much more information is needed to test associations of submissions with the participants’ demographic background, for instance to check if this weak positive correlation is due to the female surplus in urban areas.

Our model estimates imply that submission numbers could partly reflect environmental conditions. The presence of water is one key factors of mosquito development and occurrence^33^, suggesting the positive correlation for both model parts may not only be due to a higher probability of encountering mosquitoes near breeding habitats, but also in larger numbers. The prevalence of water bodies in the north-eastern part of Germany might further contribute to the stronger engagement in this area, where—contrary to other regions with similar landscape features^69^—management actions are not carried out (e.g. large-scale application of *Bacillus thuringiensis israelensis* by helicopter along sections of the river Rhine).

Of the environmental variables that negatively relate to the number of submissions, wind speed affects both the number and probability of submissions, because the absence or inactivity of mosquitoes in breezy coastal or mountainous regions is likely to result in few mosquito encounters. More surprising is the inverse correlation of precipitation with only submission counts, as mosquito breeding depends on the availability of water, with small natural sites such as tree holes or small ponds strongly prone to desiccation. Yet most submissions came from people’s private surroundings, houses or gardens, and therefore from areas that could have persisting water sources independent of precipitation. People inadvertently tend to create perfect mosquito habitats by garden design and irrigation, and the topmost mosquito taxa submitted to the ‘Mückenatlas’ (*Culex pipiens* complex, *Cs. annulata* or *Ae. japonicus)*, readily breed in garden ponds and in a range of artificial containers such as water-filled rain barrels, flower-pot dishes, vases or bird baths^70,71^. The exclusive association of precipitation with submission counts again indicates a possible relationship with engaged communities in the north-eastern part of Germany, where precipitation is lower compared to the rest of the country^72^.

We aimed at reflecting mosquito seasonality by calculating the overall mean of precipitation (and temperature) from March to November for the years 2012 to 2017. While adequate for a first exploration of driving factors as conducted in this study, this simplification would be insufficient and inexact for predicting submission numbers, given the likely influence of climate on their temporal variation (as already discussed). Instead of using the standard approach with weather variables like seasonal means, modelling the distribution of submissions and also of species, could be improved by variable decomposition to allow for inherent spatio-temporal fluctuations as well as for anomalies^73^, which would be especially useful to account for the phenology of semi-aquatic arthropods like mosquitoes.

## Conclusions

The ‘Mückenatlas’ is an analogue citizen science project open to everyone, without any preparation or constraints like collection kits, educational training or sampling protocols. This study lays the foundation to future applications of this specific opportunistic dataset that does not struggle with data quality, but displays massive spatio-temporal variation in citizen submissions of mosquito samples. Although environmental factors do play their part in spatio-temporal variation of ‘Mückenatlas’ submissions, it is the citizens and their recording activities that primarily shape the data.

Our findings have five important implications for (mosquito-related) citizen science monitoring projects. First, an unstructured, opportunistic programme such as the ‘Mückenatlas’ is well suited to collect data from several taxa over a long period of time and over a large area in order to mitigate uncontrollable effects such as climate variability, which, in the case of mosquitoes, strongly influences their occurrence and abundance. In contrast, studies on a smaller spatial scale, for specific species or habitats, or over a shorter period of time, would benefit from a structured approach to better anticipate the spatio-temporal effects of environmental factors through sampling protocols or pre-selection of place and time.

Second, the main anthropogenic causes of spatial bias in opportunistic data collections are human population and the preference of citizens to take part in projects that allow data collection at home or close by^74^. Citizen science monitoring programmes can prepare for this urban clustering and overrepresentation of observations from artificial surfaces by adapting protocol design and regulating the recruitment of participants (e.g. by creating sample units with fixed numbers of participants), by clearly defining their research questions and project goals, and by developing strategies for appropriate data analysis^75,76^.

Third, more engaged communities cause spatial bias, but these location-based effects could also be used to the advantage of the project, e.g. in the case of mosquito-related citizen science projects to tap local knowledge about mosquito abundance^77^.

Fourth, due to their impact on human health and well-being, mosquitoes are a regular media topic in many countries. The rush for the ‘Mückenatlas’ in 2016, probably triggered by the ZIKA epidemic, demonstrate the influence of the media, but also emphasise the usefulness of media for citizen science, whether for general recruitment, as a specific appeal for a particular region (e.g. on the frontline of an invader's spread) or to draw attention to certain species of scientific interest. Eritja et al.^78^ used the media and place-based effects to activate the local community to search for *Ae. japonicus* after a first record in northern Spain.

Finally, if the data situation is suitable, a hurdle model can be used to test if certain variables influence the number and/or probability of an observation. This could be done, for example, in the improvement or planning of citizen science projects, e.g. by taking additional, more targeted measures to recruit from areas for which the model predicts low participation. By including demographic information, we tested the applicability in large-scale spatial modelling in the context of citizen science. Although the approach is promising for identifying bias caused by national demographic trends, it cannot replace the accuracy of a social science survey of participants. For example, it would then also be possible to find out whether stronger regional engagement is associated with the attitude towards participatory formats of former citizens of East versus West Germany.

This study and many existing and emerging citizen science projects show that the public can provide valuable support in monitoring biodiversity, also of arthropod vectors, but there is still great potential to develop methods to improve data robustness.

## Supplementary Information

Supplementary Information.

## Data Availability

For the time being, the raw data used for this study cannot be provided publicly, as the geo-references are connected with the participants’ home addresses. Sharing the raw data would violate the personal privacy of the citizen scientists. However, the subset of spatial data (raster) used for modelling submission counts is available via the Open Research Data repository at the Leibniz-Centre for Agricultural Landscape Research (ZALF), Germany, https://www.doi.org/10.4228/ZALF.DK.153.
