# Supplementary material for: Drivers of spatio-temporal variation in mosquito submissions to the citizen science project ‘Mückenatlas’
Source: Sci Rep. 2021 Jan 14;11:1356. doi: 10.1038/s41598-020-80365-3 (PMC7809264; doi:10.1038/s41598-020-80365-3)
Supplement: Supplementary file 1 — Supplementary Information. [file 41598_2020_80365_MOESM1_ESM.pdf]

Supplementary Information

Drivers of spatio-temporal variation in mosquito submissions to the citizen science project 'Mückenatlas'

Nadja Pernat[*1,3,5], Helge Kampen[2], Florian Ruland[3,4,5], Jonathan M. Jeschke[3,4,5], Doreen Werner[1,5]

[1] Leibniz Centre for Agricultural Landscape Research, Eberswalder Str. 84, 15374 Müncheberg, Germany

[2] Friedrich-Loeffler-Institut, Federal Research Institute for Animal Health, Südufer 10, 17493 Greifswald – Insel Riems, Germany

[3] Freie Universität Berlin, Department of Biology, Chemistry, Pharmacy, Institute of Biology, Königin-Luise-Str. 1-3, 14195 Berlin, Germany

[4] Leibniz-Institute of Freshwater Ecology and Inland Fisheries (IGB), Müggelseedamm 310, 12587 Berlin, Germany

[5] Berlin-Brandenburg Institute of Advanced Biodiversity Research (BBIB), Königin-Luise-Str. 2-4, 14195 Berlin, Germany

*Corresponding author: Nadja Pernat, Leibniz Centre for Agricultural Landscape Research, Eberswalder Str. 84, 15374 Muencheberg, Germany. Email: nadja.pernat@zalf.de

Supplementary Table S1: Covariates (i) automatically exported by the CULBASE database and (ii) manually created for this study (these are highlighted in grey).

Variables	Explanation
Identification (Id)	Identification number
Species code (specc)	Internal hierarchical key for species, numeric
Species name original (spec.o)	Species or - in not determinable cases - genus name, character
Species name adapted (specn)	Species names only, genus set to NA, character
Geo-reference (xvalue)	Geographical latitude, decimal in WSG1984
Geo-reference (yvalue)	Geographical longitude, decimal in WSG1984
Village name (villn)	Nearest village of catch location, character
Corine Landcover Category code (coverc)	Corine Land Cover Category (level 3), numeric
Corine Landcover Category name (covern)	Corine Land Cover Category (level 3), character
County name (countyn)	Federal state name in English, character
County code (countyc)	Federal state by numbers 1 to 16, numeric
Municipality name (munin)	Municipality name of nearest village, character
Date (catchdate)	Date of catch, YYYY-MM-DD
Biotope original (bio.o)	Participant notion about find spot, character
Biotope category (biocat)	Participant notion about find spot categorised (agricultural area, artificial vegetated area, captive breeding, cemetery, coastal wetland, farmstead/stable, forest, home indoors, home outdoors, industrial area, inland water, inland wetland, intersection home indoor/outdoor, mineral extraction site, overwintering ground, public building, transport area, transport vehicle, trap), character

Supplementary Figure S1: Raster maps of the non-factor predictors considered (5 x 5 km^2 grid across Germany)

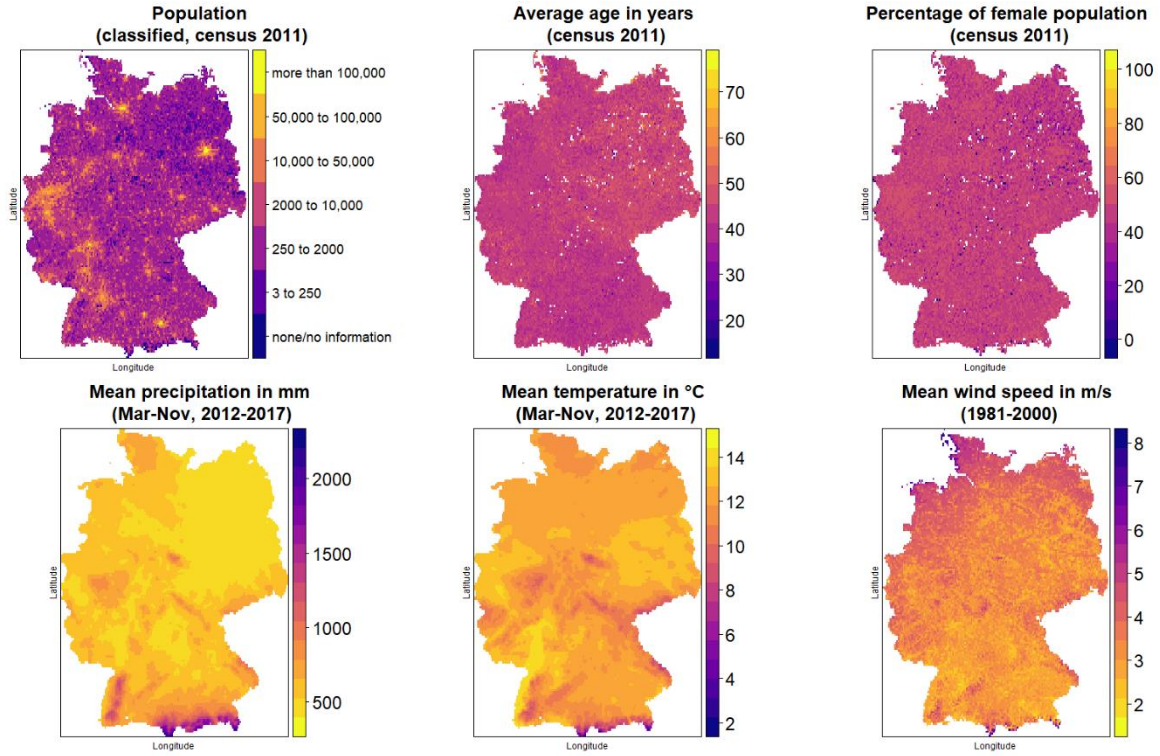

Supplementary Figure S2: Raster grid of Germany (5 x 5 km^2 cell size) showing per-capita rates of submissions from biotope category 'home' (white grid cells = zero submission and zero/invalid population data). Federal states of Germany (SH = Schleswig-Holstein, HH = Hamburg, HB = Bremen, NI = Lower Saxony, NW = North Rhine-Westphalia, HE = Hesse, RP = Rhineland Palatinate, SL = Saarland, BW = Baden-Wuerttemberg, BY = Bavaria, MV = Mecklenburg Western-Pomerania, BB = Brandenburg, BE = Berlin, ST = Saxony-Anhalt, SN = Saxony, TH = Thuringia. Raster map was created in R version 3.5.2., the German map outlining the federal states was drawn using QGis version 3.4.2. (Quantum GIS Geographic Information System, Open Source Geospatial Foundation Project. http://www.qgis.org/en/site/).

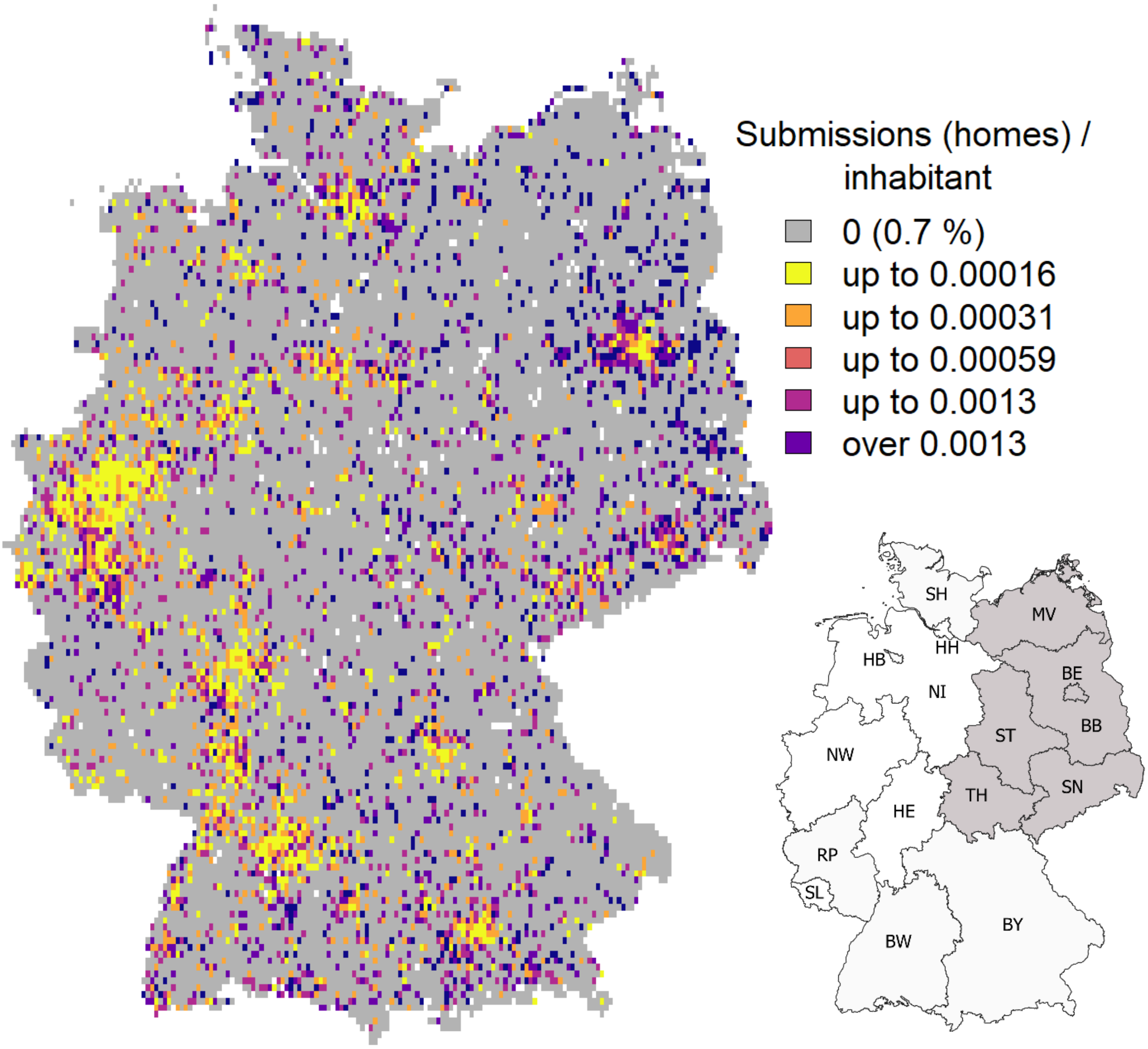

Supplementary Table S2: Complete output of Automated Model Selection (AMS)

	count_(Interc	count_east	count_fem	count_pop	count_preci	count_water	count_wind	zero_(Interce	zero_east	zero_fem	zero_pop	zero_preci	zero_water	zero_wind	df	logLik	AICc	delta	weight
3582	-0,06671637	0,57575795	NA	4,34E-05	-0,00071955	0,41051829	-0,17478186	-2,59012234	0,38593256	0,02081194	0,00021148	NA	0,47514204	-0,138389	13	-18905,1983	37836,4167	0	0.327803950016256
3584	-0,47166397	0,58945108	0,00876766	4,25E-05	-0,00073797	0,4135805	-0,1717464	-2,59012234	0,38593256	0,02081194	0,00021148	NA	0,47514204	-0,138389	14	-18904,2241	37836,4714	0,05472222	0.318956459982774
4094	-0,06671637	0,57575795	NA	4,34E-05	-0,00071955	0,41051829	-0,17478186	-2,66976045	0,40191971	0,02073197	0,0002118	0,00011864	0,47565984	-0,13600353	14	-18904,8563	37837,7358	1,31909727	0.169502399257558
4096	-0,47166397	0,58945108	0,00876766	4,25E-05	-0,00073797	0,4135805	-0,1717464	-2,66976045	0,40191971	0,02073197	0,0002118	0,00011864	0,47565984	-0,13600353	15	-18903,8821	37837,7908	1,37404122	0.164909214144916
3574	-0,67953035	0,68234123	NA	4,46E-05	NA	0,41541375	-0,13795142	-2,59012234	0,38593256	0,02081194	0,00021148	NA	0,47514204	-0,138389	12	-18910,3849	37844,7871	8,37034848	0.00498902988198517
3576	-1,04079715	0,69637088	0,00755047	4,39E-05	NA	0,41816409	-0,13461471	-2,59012234	0,38593256	0,02081194	0,00021148	NA	0,47514204	-0,138389	13	-18909,6657	37845,3515	8,93473439	0.003762367900634
4086	-0,67953035	0,68234123	NA	4,46E-05	NA	0,41541375	-0,13795142	-2,66976045	0,40191971	0,02073197	0,0002118	0,00011864	0,47565984	-0,13600353	13	-18910,0429	37846,1059	9,68922405	0.00258003687090344
4088	-1,04079715	0,69637088	0,00755047	4,39E-05	NA	0,41816409	-0,13461471	-2,66976045	0,40191971	0,02073197	0,0002118	0,00011864	0,47565984	-0,13600353	14	-18909,3237	37846,6706	10,2538317	0.00194546278656941
3552	-1,28042467	0,61751101	0,00965662	4,55E-05	-0,00054807	0,40424516	NA	-2,59012234	0,38593256	0,02081194	0,00021148	NA	0,47514204	-0,138389	13	-18910,4703	37846,9608	10,544065	0.00168267234555645
3550	-0,85074914	0,60334436	NA	4,65E-05	-0,00052401	0,40049867	NA	-2,59012234	0,38593256	0,02081194	0,00021148	NA	0,47514204	-0,138389	12	-18911,6462	37847,3096	10,8928581	0.00141338380492759
4064	-1,28042467	0,61751101	0,00965662	4,55E-05	-0,00054807	0,40424516	NA	-2,66976045	0,40191971	0,02073197	0,0002118	0,00011864	0,47565984	-0,13600353	14	-18910,1283	37848,2799	11,8631623	0.000870084084471887
4062	-0,85074914	0,60334436	NA	4,65E-05	-0,00052401	0,40049867	NA	-2,66976045	0,40191971	0,02073197	0,0002118	0,00011864	0,47565984	-0,13600353	13	-18911,3042	37848,6285	12,2117336	0.0007309201218895
3542	-1,1987332	0,68175498	NA	4,69E-05	NA	0,40581671	NA	-2,59012234	0,38593256	0,02081194	0,00021148	NA	0,47514204	-0,138389	11	-18914,56	37851,1347	14,7179593	0.000208761264435051
3544	-1,59179834	0,69737855	0,00852736	4,60E-05	NA	0,40932129	NA	-2,59012234	0,38593256	0,02081194	0,00021148	NA	0,47514204	-0,138389	12	-18913,6436	37851,3045	14,8877722	0.000191767733508303
4054	-1,1987332	0,68175498	NA	4,69E-05	NA	0,40581671	NA	-2,66976045	0,40191971	0,02073197	0,0002118	0,00011864	0,47565984	-0,13600353	12	-18914,218	37852,4533	16,0366133	0.000107971182499365
4056	-1,59179834	0,69737855	0,00852736	4,60E-05	NA	0,40932129	NA	-2,66976045	0,40191971	0,02073197	0,0002118	0,00011864	0,47565984	-0,13600353	13	-18913,3016	37852,6234	16,2066477	9,92E+09
1534	-0,06671637	0,57575795	NA	4,34E-05	-0,00071955	0,41051829	-0,17478186	-3,07214819	0,38813022	0,02082345	0,00021556	NA	0,44803127	NA	12	-18914,6523	37853,3218	16,9050863	6,99E+09
1536	-0,47166397	0,58945108	0,00876766	4,25E-05	-0,00073797	0,4135805	-0,1717464	-3,07214819	0,38813022	0,02082345	0,00021556	NA	0,44803127	NA	13	-18913,6781	37853,3763	16,9595868	6,81E+09
2046	-0,06671637	0,57575795	NA	4,34E-05	-0,00071955	0,41051829	-0,17478186	-3,17508673	0,41103069	0,02071909	0,00021591	0,0001702	0,4495725	NA	13	-18913,9374	37853,8949	17,478209	5,25E+09
2048	-0,47166397	0,58945108	0,00876766	4,25E-05	-0,00073797	0,4135805	-0,1717464	-3,17508673	0,41103069	0,02071909	0,00021591	0,0001702	0,4495725	NA	14	-18912,9632	37853,9497	17,5329312	5,11E+08
1526	-0,67953035	0,68234123	NA	4,46E-05	NA	0,41541375	-0,13795142	-3,07214819	0,38813022	0,02082345	0,00021556	NA	0,44803127	NA	11	-18919,8389	37861,6924	25,2756564	1,06E+08
1528	-1,04079715	0,69637088	0,00755047	4,39E-05	NA	0,41816409	-0,13461471	-3,07214819	0,38813022	0,02082345	0,00021556	NA	0,44803127	NA	12	-18919,1196	37862,2565	25,8398207	8,03E+07
2038	-0,67953035	0,68234123	NA	4,46E-05	NA	0,41541375	-0,13795142	-3,17508673	0,41103069	0,02071909	0,00021591	0,0001702	0,4495725	NA	12	-18919,124	37862,2653	25,8485575	7,99E+07
2040	-1,04079715	0,69637088	0,00755047	4,39E-05	NA	0,41816409	-0,13461471	-3,17508673	0,41103069	0,02071909	0,00021591	0,0001702	0,4495725	NA	13	-18918,4048	37862,8297	26,4129434	6,03E+07
1504	-1,28042467	0,61751101	0,00965662	4,55E-05	-0,00054807	0,40424516	NA	-3,07214819	0,38813022	0,02082345	0,00021556	NA	0,44803127	NA	12	-18919,9243	37863,8659	27,4491513	3,59E+07
1502	-0,85074914	0,60334436	NA	4,65E-05	-0,00052401	0,40049867	NA	-3,07214819	0,38813022	0,02082345	0,00021556	NA	0,44803127	NA	11	-18921,1001	37864,2149	27,798166	3,02E+07
2016	-1,28042467	0,61751101	0,00965662	4,55E-05	-0,00054807	0,40424516	NA	-3,17508673	0,41103069	0,02071909	0,00021591	0,0001702	0,4495725	NA	13	-18919,2094	37864,439	28,022274	2,70E+06
2014	-0,85074914	0,60334436	NA	4,65E-05	-0,00052401	0,40049867	NA	-3,17508673	0,41103069	0,02071909	0,00021591	0,0001702	0,4495725	NA	12	-18920,3853	37864,7878	28,3710671	2,26E+07
3454	-0,06671637	0,57575795	NA	4,34E-05	-0,00071955	0,41051829	-0,17478186	-1,61349146	0,35721774	NA	0,00021669	NA	0,46315983	-0,13737061	12	-18921,8664	37867,75	31,3332937	5,15E+06
3456	-0,47166397	0,58945108	0,00876766	4,25E-05	-0,00073797	0,4135805	-0,1717464	-1,61349146	0,35721774	NA	0,00021669	NA	0,46315983	-0,13737061	13	-18920,8922	37867,8045	31,3877942	5,01E+05
1494	-1,1987332	0,68175498	NA	4,69E-05	NA	0,40581671	NA	-3,07214819	0,38813022	0,02082345	0,00021556	NA	0,44803127	NA	10	-18924,014	37868,0402	31,6234889	4,45E+06
1496	-1,59179834	0,69737855	0,00852736	4,60E-05	NA	0,40932129	NA	-3,07214819	0,38813022	0,02082345	0,00021556	NA	0,44803127	NA	11	-18923,0976	37868,2098	31,7930801	4,09E+06
2006	-1,1987332	0,68175498	NA	4,69E-05	NA	0,40581671	NA	-3,17508673	0,41103069	0,02071909	0,00021591	0,0001702	0,4495725	NA	11	-18923,2991	37868,6129	32,1961684	3,34E+06
2008	-1,59179834	0,69737855	0,00852736	4,60E-05	NA	0,40932129	NA	-3,17508673	0,41103069	0,02071909	0,00021591	0,0001702	0,4495725	NA	12	-18922,3827	37868,7827	32,3659812	3,07E+05
3966	-0,06671637	0,57575795	NA	4,34E-05	-0,00071955	0,41051829	-0,17478186	-1,71083893	0,37612338	NA	0,00021703	0,00013898	0,46389079	-0,13469622	13	-18921,3931	37868,8064	32,3896692	3,04E+06
3968	-0,47166397	0,58945108	0,00876766	4,25E-05	-0,00073797	0,4135805	-0,1717464	-1,71083893	0,37612338	NA	0,00021703	0,00013898	0,46389079	-0,13469622	14	-18920,4189	37868,8611	32,4443915	2,95E+06
3446	-0,67953035	0,68234123	NA	4,46E-05	NA	0,41541375	-0,13795142	-1,61349146	0,35721774	NA	0,00021669	NA	0,46315983	-0,13737061	11	-18927,053	37876,1206	39,7038638	7,83E+04
3448	-1,04079715	0,69637088	0,00755047	4,39E-05	NA	0,41816409	-0,13461471	-1,61349146	0,35721774	NA	0,00021669	NA	0,46315983	-0,13737061	12	-18926,3337	37876,6848	40,2680281	5,91E+04
3958	-0,67953035	0,68234123	NA	4,46E-05	NA	0,41541375	-0,13795142	-1,71083893	0,37612338	NA	0,00021703	0,00013898	0,46389079	-0,13469622	12	-18926,5797	37877,1767	40,7600177	4,62E+04
3960	-1,04079715	0,69637088	0,00755047	4,39E-05	NA	0,41816409	-0,13461471	-1,71083893	0,37612338	NA	0,00021703	0,00013898	0,46389079	-0,13469622	13	-18925,8605	37877,7411	41,3244036	3,48E+04
3424	-1,28042467	0,61751101	0,00965662	4,55E-05	-0,00054807	0,40424516	NA	-1,61349146	0,35721774	NA	0,00021669	NA	0,46315983	-0,13737061	12	-18927,1384	37878,2941	41,8773587	2,64E+04
3422	-0,85074914	0,60334436	NA	4,65E-05	-0,00052401	0,40049867	NA	-1,61349146	0,35721774	NA	0,00021669	NA	0,46315983	-0,13737061	11	-18928,3142	37878,6431	42,2263734	2,22E+03
3936	-1,28042467	0,61751101	0,00965662	4,55E-05	-0,00054807	0,40424516	NA	-1,71083893	0,37612338	NA	0,00021703	0,00013898	0,46389079	-0,13469622	13	-18926,6652	37879,3505	42,9337343	1,56E+04
3934	-0,85074914	0,60334436	NA	4,65E-05	-0,00052401	0,40049867	NA	-1,71083893	0,37612338	NA	0,00021703	0,00013898	0,46389079	-0,13469622	12	-18927,841	37879,6992	43,2825273	1,31E+04
3414	-1,1987332	0,68175498	NA	4,69E-05	NA	0,40581671	NA	-1,61349146	0,35721774	NA	0,00021669	NA	0,46315983	-0,13737061	10	-18931,2281	37882,4684	46,0516963	3,28E+03
3416	-1,59179834	0,69737855	0,00852736	4,60E-05	NA	0,40932129	NA	-1,61349146	0,35721774	NA	0,00021669	NA	0,46315983	-0,13737061	11	-18930,3117	37882,638	46,2212875	3,01E+03
3926	-1,1987332	0,68175498	NA	4,69E-05	NA	0,40581671	NA	-1,71083893	0,37612338	NA	0,00021703	0,00013898	0,46389079	-0,13469622	11	-18930,7549	37883,5244	47,1076286	1,93E+03
3928	-1,59179834	0,69737855	0,00852736	4,60E-05	NA	0,40932129	NA	-1,71083893	0,37612338	NA	0,00021703	0,00013898	0,46389079	-0,13469622	12	-18929,8385	37883,6942	47,2774414	1,78E+03
1406	-0,06671637	0,57575795	NA	4,34E-05	-0,00071955	0,41051829	-0,17478186	-2,09152745	0,35956015	NA	0,00022076	NA	0,43622845	NA	11	-18931,2287	37884,4719	48,0552078	1,20E+03
1408	-0,47166397	0,58945108	0,00876766	4,25E-05	-0,00073797	0,4135805	-0,1717464	-2,09152745	0,35956015	NA	0,00022076	NA	0,43622845	NA	12	-18930,2545	37884,5262	48,1094867	1,17E+03
1918	-0,06671637	0,57575795	NA	4,34E-05	-0,00071955	0,41051829	-0,17478186	-2,2103983	0,38500536	NA	0,00022113	0,00018745	0,43806177	NA	12	-18930,3546	37884,7265	48,3097419	1,06E+03
1920	-0,47166397	0,58945108	0,00876766	4,25E-05	-0,00073797	0,4135805	-0,1717464	-2,2103983	0,38500536	NA	0,00022113	0,00018745	0,43806177	NA	13	-18929,3804	37884,781	48,3642424	1,03E+03
3566	-0,14195779	0,65649184	NA	4,36E-05	-0,00074998	NA	-0,15445798	-2,59012234	0,38593256	0,02081194	0,00021148	NA	0,47514204	-0,138389	12	-18930,7275	37885,4722	49,0554584	7,30E+02
3568	-0,45110821	0,66723944	0,00674107	4,29E-05	-0,00076432	NA	-0,15224001	-2,59012234	0,38593256	0,02081194	0,00021148	NA	0,47514204	-0,138389	13	-18930,1693	37886,3587	49,9420148	4,69E+02
4078	-0,14195779	0,65649184	NA	4,36E-05	-0,00074998	NA	-0,15445798	-2,66976045	0,40191971	0,02073197	0,0002118	0,00011864	0,47565984	-0,13600353	13	-18930,3855	37886,7911	50,374334	3,78E+02
4080	-0,45110821	0,66723944	0,00674107	4,29E-05	-0,00076432	NA	-0,15224001	-2,66976045	0,40191971	0,02073197	0,0002118	0,00011864	0,47565984	-0,13600353	14	-18929,8273	37887,6778	51,2611121	2,42E+02

1398	-0,67953035	0,68234123	NA	4,46E-05	NA	0,41541375	-0,13795142	-2,09152745	0,35956015	NA	0,00022076	NA	0,43622845	NA	10	-18936,4153	37892,8427	56,4259996	1,83E+01
1910	-0,67953035	0,68234123	NA	4,46E-05	NA	0,41541375	-0,13795142	-2,2103983	0,38500536	NA	0,00022113	0,00018745	0,43806177	NA	11	-18935,5412	37893,097	56,680312	1,61E+01
1400	-1,04079715	0,69637088	0,00755047	4,39E-05	NA	0,41816409	-0,13461471	-2,09152745	0,35956015	NA	0,00022076	NA	0,43622845	NA	11	-18935,696	37893,4067	56,9899422	1,38E+01
3534	-0,8455927	0,67951403	NA	4,64E-05	-0,00057321	NA	NA	-2,59012234	0,38593256	0,02081194	0,00021148	NA	0,47514204	-0,138389	11	-18935,7479	37893,5105	57,093748	1,31E+01
1912	-1,04079715	0,69637088	0,00755047	4,39E-05	NA	0,41816409	-0,13461471	-2,2103983	0,38500536	NA	0,00022113	0,00018745	0,43806177	NA	12	-18934,822	37893,6612	57,2444763	1,22E+01
3536	-1,17938783	0,69098467	0,00751019	4,56E-05	-0,00059207	NA	NA	-2,59012234	0,38593256	0,02081194	0,00021148	NA	0,47514204	-0,138389	12	-18935,058	37894,1332	57,7165045	9,61E+00
3558	-0,79586264	0,77122371	NA	4,50E-05	NA	NA	-0,11503973	-2,59012234	0,38593256	0,02081194	0,00021148	NA	0,47514204	-0,138389	11	-18936,3216	37894,6579	58,2411766	7,39E+00
4046	-0,8455927	0,67951403	NA	4,64E-05	-0,00057321	NA	NA	-2,66976045	0,40191971	0,02073197	0,0002118	0,00011864	0,47565984	-0,13600353	12	-18935,4059	37894,8291	58,4124019	6,78E+00
1376	-1,28042467	0,61751101	0,00965662	4,55E-05	-0,00054807	0,40424516	NA	-2,09152745	0,35956015	NA	0,00022076	NA	0,43622845	NA	11	-18936,5007	37895,016	58,5992728	6,18E+00
1888	-1,28042467	0,61751101	0,00965662	4,55E-05	-0,00054807	0,40424516	NA	-2,2103983	0,38500536	NA	0,00022113	0,00018745	0,43806177	NA	12	-18935,6266	37895,2705	58,8538069	5,44E+00
1374	-0,85074914	0,60334436	NA	4,65E-05	-0,00052401	0,40049867	NA	-2,09152745	0,35956015	NA	0,00022076	NA	0,43622845	NA	10	-18937,6765	37895,3652	58,9485092	5,19E+00
4048	-1,17938783	0,69098467	0,00751019	4,56E-05	-0,00059207	NA	NA	-2,66976045	0,40191971	0,02073197	0,0002118	0,00011864	0,47565984	-0,13600353	13	-18934,716	37895,4521	59,0353801	4,97E+00
1886	-0,85074914	0,60334436	NA	4,65E-05	-0,00052401	0,40049867	NA	-2,2103983	0,38500536	NA	0,00022113	0,00018745	0,43806177	NA	11	-18936,8025	37895,6195	59,2028216	4,57E+00
3560	-1,05409025	0,78157333	0,00543311	4,44E-05	NA	NA	-0,11273577	-2,59012234	0,38593256	0,02081194	0,00021148	NA	0,47514204	-0,138389	12	-18935,9614	37895,94	59,5232554	3,89E+00
4070	-0,79586264	0,77122371	NA	4,50E-05	NA	NA	-0,11503973	-2,66976045	0,40191971	0,02073197	0,0002118	0,00011864	0,47565984	-0,13600353	12	-18935,9796	37895,9766	59,5598305	3,82E+00
4072	-1,05409025	0,78157333	0,00543311	4,44E-05	NA	NA	-0,11273577	-2,66976045	0,40191971	0,02073197	0,0002118	0,00011864	0,47565984	-0,13600353	13	-18935,6194	37897,2589	60,8421309	2,01E+00
3526	-1,235734	0,76857786	NA	4,69E-05	NA	NA	NA	-2,59012234	0,38593256	0,02081194	0,00021148	NA	0,47514204	-0,138389	10	-18939,2203	37898,4528	62,0360279	1,11E+00
1366	-1,1987332	0,68175498	NA	4,69E-05	NA	0,40581671	NA	-2,09152745	0,35956015	NA	0,00022076	NA	0,43622845	NA	9	-18940,5904	37899,1908	62,7740536	7,66E-01
1368	-1,59179834	0,69737855	0,00852736	4,60E-05	NA	0,40932129	NA	-2,09152745	0,35956015	NA	0,00022076	NA	0,43622845	NA	10	-18939,674	37899,3601	62,9434233	7,04E-01
1878	-1,1987332	0,68175498	NA	4,69E-05	NA	0,40581671	NA	-2,2103983	0,38500536	NA	0,00022113	0,00018745	0,43806177	NA	10	-18939,7163	37899,4449	63,0281445	6,75E-01
3528	-1,52051301	0,78035551	0,00624724	4,63E-05	NA	NA	NA	-2,59012234	0,38593256	0,02081194	0,00021148	NA	0,47514204	-0,138389	11	-18938,7442	37899,503	63,0862797	6,56E-01
1880	-1,59179834	0,69737855	0,00852736	4,60E-05	NA	0,40932129	NA	-2,2103983	0,38500536	NA	0,00022113	0,00018745	0,43806177	NA	11	-18938,7999	37899,6145	63,1977357	6,20E-01
4038	-1,235734	0,76857786	NA	4,69E-05	NA	NA	NA	-2,66976045	0,40191971	0,02073197	0,0002118	0,00011864	0,47565984	-0,13600353	11	-18938,8783	37899,7712	63,3544602	5,73E-01
4040	-1,52051301	0,78035551	0,00624724	4,63E-05	NA	NA	NA	-2,66976045	0,40191971	0,02073197	0,0002118	0,00011864	0,47565984	-0,13600353	12	-18938,4022	37900,8217	64,4049336	3,39E-01
1518	-0,14195779	0,65649184	NA	4,36E-05	-0,00074998	NA	-0,15445798	-3,07214819	0,38813022	0,02082345	0,00021556	NA	0,44803127	NA	11	-18940,1814	37902,3775	65,9607664	1,56E-02
2030	-0,14195779	0,65649184	NA	4,36E-05	-0,00074998	NA	-0,15445798	-3,17508673	0,41103069	0,02071909	0,00021591	0,0001702	0,4495725	NA	12	-18939,4666	37902,9504	66,5336675	1,17E-01
1520	-0,45110821	0,66723944	0,00674107	4,29E-05	-0,00076432	NA	-0,15224001	-3,07214819	0,38813022	0,02082345	0,00021556	NA	0,44803127	NA	12	-18939,6233	37903,2638	66,8471011	1,00E-01
2032	-0,45110821	0,66723944	0,00674107	4,29E-05	-0,00076432	NA	-0,15224001	-3,17508673	0,41103069	0,02071909	0,00021591	0,0001702	0,4495725	NA	13	-18938,9084	37903,8369	67,4202238	7,51E-03
3581	0,65451489	NA	NA	3,95E-05	-0,00164357	0,48762971	-0,22170047	-2,59012234	0,38593256	0,02081194	0,00021148	NA	0,47514204	-0,138389	12	-18941,0256	37906,0684	69,6516479	2,46E-02
4093	0,65451489	NA	NA	3,95E-05	-0,00164357	0,48762971	-0,22170047	-2,66976045	0,40191971	0,02073197	0,0002118	0,00011864	0,47565984	-0,13600353	13	-18940,6836	37907,3872	70,9705235	1,27E-02
3583	0,61699723	NA	0,00084798	3,94E-05	-0,00164748	0,4880593	-0,22142621	-2,59012234	0,38593256	0,02081194	0,00021148	NA	0,47514204	-0,138389	13	-18941,0163	37908,0527	71,6359363	9,12E-03
4095	0,61699723	NA	0,00084798	3,94E-05	-0,00164748	0,4880593	-0,22142621	-2,66976045	0,40191971	0,02073197	0,0002118	0,00011864	0,47565984	-0,13600353	14	-18940,6743	37909,3718	72,9550336	4,72E-03
4030	-0,06671637	0,57575795	NA	4,34E-05	-0,00071955	0,41051829	-0,17478186	-2,05206432	NA	0,01786019	0,00020271	-0,00039168	0,51529109	-0,14768431	13	-18941,7322	37909,4844	73,0677171	4,46E-03
4032	-0,47166397	0,58945108	0,00876766	4,25E-05	-0,00073797	0,4135805	-0,1717464	-2,05206432	NA	0,01786019	0,00020271	-0,00039168	0,51529109	-0,14768431	14	-18940,758	37909,5392	73,1224393	4,34E-03
1486	-0,8455927	0,67951403	NA	4,64E-05	-0,00057321	NA	NA	-3,07214819	0,38813022	0,02082345	0,00021556	NA	0,44803127	NA	10	-18945,2019	37910,416	73,9992775	2,80E-04
1998	-0,8455927	0,67951403	NA	4,64E-05	-0,00057321	NA	NA	-3,17508673	0,41103069	0,02071909	0,00021591	0,0001702	0,4495725	NA	11	-18944,487	37910,9887	74,571957	2,10E-03
1488	-1,17938783	0,69098467	0,00751019	4,56E-05	-0,00059207	NA	NA	-3,07214819	0,38813022	0,02082345	0,00021556	NA	0,44803127	NA	11	-18944,512	37911,0385	74,6218125	2,05E-03
1510	-0,79586264	0,77122371	NA	4,50E-05	NA	NA	-0,11503973	-3,07214819	0,38813022	0,02082345	0,00021556	NA	0,44803127	NA	10	-18945,7756	37911,5634	75,1467062	1,58E-03
2000	-1,17938783	0,69098467	0,00751019	4,56E-05	-0,00059207	NA	NA	-3,17508673	0,41103069	0,02071909	0,00021591	0,0001702	0,4495725	NA	12	-18943,7971	37911,6114	75,1947135	1,54E-03
2022	-0,79586264	0,77122371	NA	4,50E-05	NA	NA	-0,11503973	-3,17508673	0,41103069	0,02071909	0,00021591	0,0001702	0,4495725	NA	11	-18945,0608	37912,1361	75,7193856	1,18E-04
1512	-1,05409025	0,78157333	0,00543311	4,44E-05	NA	NA	-0,11273577	-3,07214819	0,38813022	0,02082345	0,00021556	NA	0,44803127	NA	11	-18945,4153	37912,8453	76,4285633	8,31E-04
2024	-1,05409025	0,78157333	0,00543311	4,44E-05	NA	NA	-0,11273577	-3,17508673	0,41103069	0,02071909	0,00021591	0,0001702	0,4495725	NA	12	-18944,7005	37913,4182	77,0014644	6,24E-05
1478	-1,235734	0,76857786	NA	4,69E-05	NA	NA	NA	-3,07214819	0,38813022	0,02082345	0,00021556	NA	0,44803127	NA	9	-18948,6743	37915,3585	78,941779	2,36E-04
1990	-1,235734	0,76857786	NA	4,69E-05	NA	NA	NA	-3,17508673	0,41103069	0,02071909	0,00021591	0,0001702	0,4495725	NA	10	-18947,9594	37915,931	79,5142369	1,78E-04
3518	-0,06671637	0,57575795	NA	4,34E-05	-0,00071955	0,41051829	-0,17478186	-2,27275384	NA	0,01711404	0,00020255	NA	0,52288611	-0,14000571	12	-18946,0729	37916,163	79,7462772	1,58E-04
3520	-0,47166397	0,58945108	0,00876766	4,25E-05	-0,00073797	0,4135805	-0,1717464	-2,27275384	NA	0,01711404	0,00020255	NA	0,52288611	-0,14000571	13	-18945,0987	37916,2175	79,8007777	1,54E-05
1480	-1,52051301	0,78035551	0,00624724	4,63E-05	NA	NA	NA	-3,07214819	0,38813022	0,02082345	0,00021556	NA	0,44803127	NA	10	-18948,1982	37916,4085	79,9918093	1,40E-05
3438	-0,14195779	0,65649184	NA	4,36E-05	-0,00074998	NA	-0,15445798	-1,61349146	0,35721774	NA	0,00021669	NA	0,46315983	-0,13737061	11	-18947,3955	37916,8057	80,3889738	1,15E-04
1992	-1,52051301	0,78035551	0,00624724	4,63E-05	NA	NA	NA	-3,17508673	0,41103069	0,02071909	0,00021591	0,0001702	0,4495725	NA	11	-18947,4833	37916,9812	80,5644887	1,05E-04
3440	-0,45110821	0,66723944	0,00674107	4,29E-05	-0,00076432	NA	-0,15224001	-1,61349146	0,35721774	NA	0,00021669	NA	0,46315983	-0,13737061	12	-18946,8374	37917,692	81,2753085	7,36E-05
4022	-0,67953035	0,68234123	NA	4,46E-05	NA	0,41541375	-0,13795142	-2,05206432	NA	0,01786019	0,00020271	-0,00039168	0,51529109	-0,14768431	12	-18946,9188	37917,8548	81,4380655	6,79E-05
3950	-0,14195779	0,65649184	NA	4,36E-05	-0,00074998	NA	-0,15445798	-1,71083893	0,37612338	NA	0,00021703	0,00013898	0,46389079	-0,13469622	12	-18946,9223	37917,8618	81,4451277	6,76E-05
4024	-1,04079715	0,69637088	0,00755047	4,39E-05	NA	0,41816409	-0,13461471	-2,05206432	NA	0,01786019	0,00020271	-0,00039168	0,51529109	-0,14768431	13	-18946,1995	37918,4192	82,0024515	5,12E-05
3952	-0,45110821	0,66723944	0,00674107	4,29E-05	-0,00076432	NA	-0,15224001	-1,71083893	0,37612338	NA	0,00021703	0,00013898	0,46389079	-0,13469622	13	-18946,3641	37918,7484	82,331684	4,34E-05
4000	-1,28042467	0,61751101	0,00965662	4,55E-05	-0,00054807	0,40424516	NA	-2,05206432	NA	0,01786019	0,00020271	-0,00039168	0,51529109	-0,14768431	13	-18947,0042	37920,0285	83,6117821	2,29E-05
3998	-0,85074914	0,60334436	NA	4,65E-05	-0,00052401	0,40049867	NA	-2,05206432	NA	0,01786019	0,00020271	-0,00039168	0,51529109	-0,14768431	12	-18948,18	37920,3773	83,9605751	1,92E-05
1533	0,65451489	NA	NA	3,95E-05	-0,00164357	0,48762971	-0,22170047	-3,07214819	0,38813022	0,02082345	0,00021556	NA	0,44803127	NA	11	-18950,4795	37922,9737	86,5569558	5,25E-06
2045	0,65451489	NA	NA	3,95E-05	-0,00164357	0,48762971	-0,22170047	-3,17508673	0,41103069	0,02071909	0,00021591	0,0001702	0,4495725	NA	12	-18949,7647	37923,5466	87,1298569	3,94E-06
3549	-0,31227401	NA	NA	4,32E-05	-0,00145314	0,47935513	NA	-2,59012234	0,38593256	0,02081194	0,00021148	NA	0,47514204	-0,138389	11	-18950,9284	37923,8714	87,4546855	3,35E-06
3990	-1,1987332	0,68175498	NA	4,69E-05	NA	0,40581671	NA	-2,05206432	NA	0,01786019	0,00020271	-0,00039168	0,51529109	-0,14768431	11	-18951,0939	37924,2024	87,7856764	2,84E-06
3992	-1,59179834	0,69737855	0,00852736	4,60E-05	NA	0,40932129	NA	-2,05206432	NA	0,01786019	0,00020271	-0,00039168	0,51529109	-0,14768431	12	-18950,1775	37924,3722	87,9554892	2,61E-06

3510	-0,67953035	0,68234123	NA	4,46E-05	NA	0,41541375	-0,13795142	-2,27275384	NA	0,01711404	0,00020255	NA	0,52288611	-0,14000571	11	-18951,2595	37924,5336	88,1168473	2,41E-06
3406	-0,8455927	0,67951403	NA	4,64E-05	-0,00057321	NA	NA	-1,61349146	0,35721774	NA	0,00021669	NA	0,46315983	-0,13737061	10	-18952,416	37924,8442	88,4274849	2,06E-07
1535	0,61699723	NA	0,00084798	3,94E-05	-0,00164748	0,4880593	-0,22142621	-3,07214819	0,38813022	0,02082345	0,00021556	NA	0,44803127	NA	12	-18950,4702	37924,9577	88,5410226	1,95E-07
3512	-1,04079715	0,69637088	0,00755047	4,39E-05	NA	0,41816409	-0,13461471	-2,27275384	NA	0,01711404	0,00020255	NA	0,52288611	-0,14000571	12	-18950,5402	37925,0977	88,6810116	1,81E-06
4061	-0,31227401	NA	NA	4,32E-05	-0,00145314	0,47935513	NA	-2,66976045	0,40191971	0,02073197	0,0002118	0,00011864	0,47565984	-0,13600353	12	-18950,5864	37925,1901	88,7733394	1,73E-07
3408	-1,17938783	0,69098467	0,00751019	4,56E-05	-0,00059207	NA	NA	-1,61349146	0,35721774	NA	0,00021669	NA	0,46315983	-0,13737061	11	-18951,7261	37925,4667	89,0500199	1,51E-06
2047	0,61699723	NA	0,00084798	3,94E-05	-0,00164748	0,4880593	-0,22142621	-3,17508673	0,41103069	0,02071909	0,00021591	0,0001702	0,4495725	NA	13	-18949,7554	37925,5309	89,1141453	1,46E-06
3551	-0,39160679	NA	0,00183718	4,30E-05	-0,00146197	0,48040455	NA	-2,59012234	0,38593256	0,02081194	0,00021148	NA	0,47514204	-0,138389	12	-18950,885	37925,7872	89,3705191	1,29E-06
3918	-0,8455927	0,67951403	NA	4,64E-05	-0,00057321	NA	NA	-1,71083893	0,37612338	NA	0,00021703	0,00013898	0,46389079	-0,13469622	11	-18951,9428	37925,9001	89,4834172	1,21E-06
3430	-0,79586264	0,77122371	NA	4,50E-05	NA	NA	-0,11503973	-1,61349146	0,35721774	NA	0,00021669	NA	0,46315983	-0,13737061	10	-18952,9897	37925,9916	89,5749136	1,16E-06
3920	-1,17938783	0,69098467	0,00751019	4,56E-05	-0,00059207	NA	NA	-1,71083893	0,37612338	NA	0,00021703	0,00013898	0,46389079	-0,13469622	12	-18951,2528	37926,5229	90,1061738	8,90E-07
3488	-1,28042467	0,61751101	0,00965662	4,55E-05	-0,00054807	0,40424516	NA	-2,27275384	NA	0,01711404	0,00020255	NA	0,52288611	-0,14000571	12	-18951,3449	37926,7071	90,2903422	8,12E-07
3942	-0,79586264	0,77122371	NA	4,50E-05	NA	NA	-0,11503973	-1,71083893	0,37612338	NA	0,00021703	0,00013898	0,46389079	-0,13469622	11	-18952,5165	37927,0476	90,6308459	6,85E-07
3486	-0,85074914	0,60334436	NA	4,65E-05	-0,00052401	0,40049867	NA	-2,27275384	NA	0,01711404	0,00020255	NA	0,52288611	-0,14000571	11	-18952,5207	37927,0561	90,6393569	6,82E-07
4063	-0,39160679	NA	0,00183718	4,30E-05	-0,00146197	0,48040455	NA	-2,66976045	0,40191971	0,02073197	0,0002118	0,00011864	0,47565984	-0,13600353	13	-18950,543	37927,1061	90,6893946	6,65E-07
3432	-1,05409025	0,78157333	0,00543311	4,44E-05	NA	NA	-0,11273577	-1,61349146	0,35721774	NA	0,00021669	NA	0,46315983	-0,13737061	11	-18952,6294	37927,2735	90,8567707	6,11E-07
3944	-1,05409025	0,78157333	0,00543311	4,44E-05	NA	NA	-0,11273577	-1,71083893	0,37612338	NA	0,00021703	0,00013898	0,46389079	-0,13469622	12	-18952,1562	37928,3296	91,9129246	3,61E-07
1982	-0,06671637	0,57575795	NA	4,34E-05	-0,00071955	0,41051829	-0,17478186	-2,58652225	NA	0,01772679	0,00020696	-0,00034396	0,48679163	NA	12	-18952,6779	37929,3731	92,9563805	2,14E-07
1984	-0,47166397	0,58945108	0,00876766	4,25E-05	-0,00073797	0,4135805	-0,1717464	-2,58652225	NA	0,01772679	0,00020696	-0,00034396	0,48679163	NA	13	-18951,7037	37929,4276	93,0108811	2,08E-07
3398	-1,235734	0,76857786	NA	4,69E-05	NA	NA	NA	-1,61349146	0,35721774	NA	0,00021669	NA	0,46315983	-0,13737061	9	-18955,8884	37929,7867	93,3699864	1,74E-07
3400	-1,52051301	0,78035551	0,00624724	4,63E-05	NA	NA	NA	-1,61349146	0,35721774	NA	0,00021669	NA	0,46315983	-0,13737061	10	-18955,4123	37930,8367	94,4200167	1,03E-07
3910	-1,235734	0,76857786	NA	4,69E-05	NA	NA	NA	-1,71083893	0,37612338	NA	0,00021703	0,00013898	0,46389079	-0,13469622	10	-18955,4151	37930,8424	94,4256971	1,03E-07
3478	-1,1987332	0,68175498	NA	4,69E-05	NA	0,40581671	NA	-2,27275384	NA	0,01711404	0,00020255	NA	0,52288611	-0,14000571	10	-18955,4346	37930,8814	94,4646798	1,01E-08
3480	-1,59179834	0,69737855	0,00852736	4,60E-05	NA	0,40932129	NA	-2,27275384	NA	0,01711404	0,00020255	NA	0,52288611	-0,14000571	11	-18954,5182	37931,051	94,634271	9,25E-10
3912	-1,52051301	0,78035551	0,00624724	4,63E-05	NA	NA	NA	-1,71083893	0,37612338	NA	0,00021703	0,00013898	0,46389079	-0,13469622	11	-18954,939	37931,8927	95,475949	6,07E-08
3902	-0,06671637	0,57575795	NA	4,34E-05	-0,00071955	0,41051829	-0,17478186	-1,25486087	NA	NA	0,00020772	-0,0003441	0,50313521	-0,14558566	12	-18954,0349	37932,0871	95,6703954	5,51E-08
3904	-0,47166397	0,58945108	0,00876766	4,25E-05	-0,00073797	0,4135805	-0,1717464	-1,25486087	NA	NA	0,00020772	-0,0003441	0,50313521	-0,14558566	13	-18953,0607	37932,1416	95,724896	5,36E-08
1390	-0,14195779	0,65649184	NA	4,36E-05	-0,00074998	NA	-0,15445798	-2,09152745	0,35956015	NA	0,00022076	NA	0,43622845	NA	10	-18956,7578	37933,5278	97,1111095	2,68E-09
1902	-0,14195779	0,65649184	NA	4,36E-05	-0,00074998	NA	-0,15445798	-2,2103983	0,38500536	NA	0,00022113	0,00018745	0,43806177	NA	11	-18955,8838	37933,7821	97,365422	2,36E-09
1470	-0,06671637	0,57575795	NA	4,34E-05	-0,00071955	0,41051829	-0,17478186	-2,75826159	NA	0,01709531	0,00020664	NA	0,4949792	NA	11	-18956,0664	37934,1474	97,7306385	1,97E-08
1472	-0,47166397	0,58945108	0,00876766	4,25E-05	-0,00073797	0,4135805	-0,1717464	-2,75826159	NA	0,01709531	0,00020664	NA	0,4949792	NA	12	-18955,0922	37934,2016	97,7849174	1,91E-08
1392	-0,45110821	0,66723944	0,00674107	4,29E-05	-0,00076432	NA	-0,15224001	-2,09152745	0,35956015	NA	0,00022076	NA	0,43622845	NA	11	-18956,1997	37934,4139	97,9972226	1,72E-08
1904	-0,45110821	0,66723944	0,00674107	4,29E-05	-0,00076432	NA	-0,15224001	-2,2103983	0,38500536	NA	0,00022113	0,00018745	0,43806177	NA	12	-18955,3256	37934,6685	98,2517567	1,52E-08
3390	-0,06671637	0,57575795	NA	4,34E-05	-0,00071955	0,41051829	-0,17478186	-1,4783871	NA	NA	0,00020739	NA	0,51055393	-0,13912561	11	-18957,4289	37936,8723	100,455588	5,03E-09
3392	-0,47166397	0,58945108	0,00876766	4,25E-05	-0,00073797	0,4135805	-0,1717464	-1,4783871	NA	NA	0,00020739	NA	0,51055393	-0,13912561	12	-18956,4547	37936,9266	100,509867	4,90E-09
3453	0,65451489	NA	NA	3,95E-05	-0,00164357	0,48762971	-0,22170047	-1,61349146	0,35721774	NA	0,00021669	NA	0,46315983	-0,13737061	11	-18957,6936	37937,4019	100,985163	3,86E-09
1974	-0,67953035	0,68234123	NA	4,46E-05	NA	0,41541375	-0,13795142	-2,58652225	NA	0,01772679	0,00020696	-0,00034396	0,48679163	NA	11	-18957,8645	37937,7437	101,326951	3,26E-09
1976	-1,04079715	0,69637088	0,00755047	4,39E-05	NA	0,41816409	-0,13461471	-2,58652225	NA	0,01772679	0,00020696	-0,00034396	0,48679163	NA	12	-18957,1453	37938,3078	101,891115	2,46E-09
3965	0,65451489	NA	NA	3,95E-05	-0,00164357	0,48762971	-0,22170047	-1,71083893	0,37612338	NA	0,00021703	0,00013898	0,46389079	-0,13469622	12	-18957,2204	37938,458	102,041317	2,28E-09
3455	0,61699723	NA	0,00084798	3,94E-05	-0,00164748	0,4880593	-0,22142621	-1,61349146	0,35721774	NA	0,00021669	NA	0,46315983	-0,13737061	12	-18957,6843	37939,386	102,96923	1,43E-09
1952	-1,28042467	0,61751101	0,00965662	4,55E-05	-0,00054807	0,40424516	NA	-2,58652225	NA	0,01772679	0,00020696	-0,00034396	0,48679163	NA	12	-18957,95	37939,9172	103,500446	1,10E-09
1950	-0,85074914	0,60334436	NA	4,65E-05	-0,00052401	0,40049867	NA	-2,58652225	NA	0,01772679	0,00020696	-0,00034396	0,48679163	NA	11	-18959,1258	37940,2662	103,84946	9,23E-10
3967	0,61699723	NA	0,00084798	3,94E-05	-0,00164748	0,4880593	-0,22142621	-1,71083893	0,37612338	NA	0,00021703	0,00013898	0,46389079	-0,13469622	13	-18957,2111	37940,4423	104,025606	8,45E-10
3894	-0,67953035	0,68234123	NA	4,46E-05	NA	0,41541375	-0,13795142	-1,25486087	NA	NA	0,00020772	-0,0003441	0,50313521	-0,14558566	11	-18959,2215	37940,4577	104,040966	8,38E-10
1501	-0,31227401	NA	NA	4,32E-05	-0,00145314	0,47935513	NA	-3,07214819	0,38813022	0,02082345	0,00021556	NA	0,44803127	NA	10	-18960,3824	37940,7769	104,360215	7,15E-10
3896	-1,04079715	0,69637088	0,00755047	4,39E-05	NA	0,41816409	-0,13461471	-1,25486087	NA	NA	0,00020772	-0,0003441	0,50313521	-0,14558566	12	-18958,5023	37941,0219	104,60513	6,32E-10
2013	-0,31227401	NA	NA	4,32E-05	-0,00145314	0,47935513	NA	-3,17508673	0,41103069	0,02071909	0,00021591	0,0001702	0,4495725	NA	11	-18959,6675	37941,3496	104,932895	5,37E-11
1358	-0,8455927	0,67951403	NA	4,64E-05	-0,00057321	NA	NA	-2,09152745	0,35956015	NA	0,00022076	NA	0,43622845	NA	9	-18961,7783	37941,5666	105,149842	4,82E-10
1870	-0,8455927	0,67951403	NA	4,64E-05	-0,00057321	NA	NA	-2,2103983	0,38500536	NA	0,00022113	0,00018745	0,43806177	NA	10	-18960,9042	37941,8207	105,403933	4,24E-10
1360	-1,17938783	0,69098467	0,00751019	4,56E-05	-0,00059207	NA	NA	-2,09152745	0,35956015	NA	0,00022076	NA	0,43622845	NA	10	-18961,0884	37942,1889	105,772156	3,53E-10
1872	-1,17938783	0,69098467	0,00751019	4,56E-05	-0,00059207	NA	NA	-2,2103983	0,38500536	NA	0,00022113	0,00018745	0,43806177	NA	11	-18960,2143	37942,4432	106,026468	3,11E-10
1462	-0,67953035	0,68234123	NA	4,46E-05	NA	0,41541375	-0,13795142	-2,75826159	NA	0,01709531	0,00020664	NA	0,4949792	NA	10	-18961,253	37942,5182	106,10143	2,99E-10
3872	-1,28042467	0,61751101	0,00965662	4,55E-05	-0,00054807	0,40424516	NA	-1,25486087	NA	NA	0,00020772	-0,0003441	0,50313521	-0,14558566	12	-18959,307	37942,6312	106,214461	2,83E-10
1503	-0,39160679	NA	0,00183718	4,30E-05	-0,00146197	0,48040455	NA	-3,07214819	0,38813022	0,02082345	0,00021556	NA	0,44803127	NA	11	-18960,339	37942,6925	106,275827	2,74E-10
1382	-0,79586264	0,77122371	NA	4,50E-05	NA	NA	-0,11503973	-2,09152745	0,35956015	NA	0,00022076	NA	0,43622845	NA	9	-18962,352	37942,714	106,297271	2,71E-10
1894	-0,79586264	0,77122371	NA	4,50E-05	NA	NA	-0,11503973	-2,2103983	0,38500536	NA	0,00022113	0,00018745	0,43806177	NA	10	-18961,478	37942,9681	106,551362	2,39E-11
3870	-0,85074914	0,60334436	NA	4,65E-05	-0,00052401	0,40049867	NA	-1,25486087	NA	NA	0,00020772	-0,0003441	0,50313521	-0,14558566	11	-18960,4828	37942,9802	106,563475	2,37E-10
1464	-1,04079715	0,69637088	0,00755047	4,39E-05	NA	0,41816409	-0,13461471	-2,75826159	NA	0,01709531	0,00020664	NA	0,4949792	NA	11	-18960,5337	37943,0821	106,665373	2,26E-10
2015	-0,39160679	NA	0,00183718	4,30E-05	-0,00146197	0,48040455	NA	-3,17508673	0,41103069	0,02071909	0,00021591	0,0001702	0,4495725	NA	12	-18959,6241	37943,2655	106,848728	2,06E-10
1384	-1,05409025	0,78157333	0,00543311	4,44E-05	NA	NA	-0,11273577	-2,09152745	0,35956015	NA	0,00022076	NA	0,43622845	NA	10	-18961,9917	37943,9956	107,578907	1,43E-10
1942	-1,1987332	0,68175498	NA	4,69E-05	NA	0,40581671	NA	-2,58652225	NA	0,01772679	0,00020696	-0,00034396	0,48679163	NA	10	-18962,0397	37944,0915	107,674783	1,36E-10

1896	-1,05409025	0,78157333	0,00543311	4,44E-05	NA	NA	-0,11273577	-2,2103983	0,38500536	NA	0,00022113	0,00018745	0,43806177	NA	11	-18961,1177	37944,2499	107,833219	1,26E-10
1944	-1,59179834	0,69737855	0,00852736	4,60E-05	NA	0,40932129	NA	-2,58652225	NA	0,01772679	0,00020696	-0,00034396	0,48679163	NA	11	-18961,1232	37944,2611	107,844374	1,25E-10
1440	-1,28042467	0,61751101	0,00965662	4,55E-05	-0,00054807	0,40424516	NA	-2,75826159	NA	0,01709531	0,00020664	NA	0,4949792	NA	11	-18961,3384	37944,6914	108,274704	1,01E-10
1438	-0,85074914	0,60334436	NA	4,65E-05	-0,00052401	0,40049867	NA	-2,75826159	NA	0,01709531	0,00020664	NA	0,4949792	NA	10	-18962,5142	37945,0407	108,62394	8,48E-11
3382	-0,67953035	0,68234123	NA	4,46E-05	NA	0,41541375	-0,13795142	-1,4783871	NA	NA	0,00020739	NA	0,51055393	-0,13912561	10	-18962,6155	37945,2431	108,82638	7,66E-11
3384	-1,04079715	0,69637088	0,00755047	4,39E-05	NA	0,41816409	-0,13461471	-1,4783871	NA	NA	0,00020739	NA	0,51055393	-0,13912561	11	-18961,8962	37945,807	109,390323	5,78E-11
1350	-1,235734	0,76857786	NA	4,69E-05	NA	NA	NA	-2,09152745	0,35956015	NA	0,00022076	NA	0,43622845	NA	8	-18965,2507	37946,5093	110,092565	4,07E-11
1862	-1,235734	0,76857786	NA	4,69E-05	NA	NA	NA	-2,2103983	0,38500536	NA	0,00022113	0,00018745	0,43806177	NA	9	-18964,3766	37946,7632	110,346435	3,58E-11
3862	-1,1987332	0,68175498	NA	4,69E-05	NA	0,40581671	NA	-1,25486087	NA	NA	0,00020772	-0,0003441	0,50313521	-0,14558566	10	-18963,3967	37946,8055	110,388798	3,51E-11
3864	-1,59179834	0,69737855	0,00852736	4,60E-05	NA	0,40932129	NA	-1,25486087	NA	NA	0,00020772	-0,0003441	0,50313521	-0,14558566	11	-18962,4803	37946,9751	110,558389	3,22E-11
3360	-1,28042467	0,61751101	0,00965662	4,55E-05	-0,00054807	0,40424516	NA	-1,4783871	NA	NA	0,00020739	NA	0,51055393	-0,13912561	11	-18962,7009	37947,4164	110,999653	2,58E-11
1352	-1,52051301	0,78035551	0,00624724	4,63E-05	NA	NA	NA	-2,09152745	0,35956015	NA	0,00022076	NA	0,43622845	NA	9	-18964,7746	37947,5591	111,142374	2,41E-11
3358	-0,85074914	0,60334436	NA	4,65E-05	-0,00052401	0,40049867	NA	-1,4783871	NA	NA	0,00020739	NA	0,51055393	-0,13912561	10	-18963,8767	37947,7656	111,34889	2,17E-11
1864	-1,52051301	0,78035551	0,00624724	4,63E-05	NA	NA	NA	-2,2103983	0,38500536	NA	0,00022113	0,00018745	0,43806177	NA	10	-18963,9005	37947,8132	111,396465	2,12E-13
1430	-1,1987332	0,68175498	NA	4,69E-05	NA	0,40581671	NA	-2,75826159	NA	0,01709531	0,00020664	NA	0,4949792	NA	9	-18965,4281	37948,8662	112,449484	1,25E-11
1432	-1,59179834	0,69737855	0,00852736	4,60E-05	NA	0,40932129	NA	-2,75826159	NA	0,01709531	0,00020664	NA	0,4949792	NA	10	-18964,5117	37949,0356	112,618854	1,15E-11
1854	-0,06671637	0,57575795	NA	4,34E-05	-0,00071955	0,41051829	-0,17478186	-1,78635429	NA	NA	0,0002119	-0,00029974	0,47515083	NA	11	-18964,7167	37951,448	115,031296	3,44E-12
1856	-0,47166397	0,58945108	0,00876766	4,25E-05	-0,00073797	0,4135805	-0,1717464	-1,78635429	NA	NA	0,0002119	-0,00029974	0,47515083	NA	12	-18963,7425	37951,5023	115,085575	3,35E-12
3350	-1,1987332	0,68175498	NA	4,69E-05	NA	0,40581671	NA	-1,4783871	NA	NA	0,00020739	NA	0,51055393	-0,13912561	9	-18966,7906	37951,5912	115,174434	3,20E-12
3352	-1,59179834	0,69737855	0,00852736	4,60E-05	NA	0,40932129	NA	-1,4783871	NA	NA	0,00020739	NA	0,51055393	-0,13912561	10	-18965,8742	37951,7605	115,343804	2,94E-14
1405	0,65451489	NA	NA	3,95E-05	-0,00164357	0,48762971	-0,22170047	-2,09152745	0,35956015	NA	0,00022076	NA	0,43622845	NA	10	-18967,0559	37954,124	117,707299	9,03E-13
1917	0,65451489	NA	NA	3,95E-05	-0,00164357	0,48762971	-0,22170047	-2,2103983	0,38500536	NA	0,00022113	0,00018745	0,43806177	NA	11	-18966,1819	37954,3783	117,961611	7,95E-13
1342	-0,06671637	0,57575795	NA	4,34E-05	-0,00071955	0,41051829	-0,17478186	-1,96172815	NA	NA	0,00021146	NA	0,48287384	NA	10	-18967,3216	37954,6554	118,238648	6,92E-13
1344	-0,47166397	0,58945108	0,00876766	4,25E-05	-0,00073797	0,4135805	-0,1717464	-1,96172815	NA	NA	0,00021146	NA	0,48287384	NA	11	-18966,3474	37954,7094	118,292705	6,74E-13
3421	-0,31227401	NA	NA	4,32E-05	-0,00145314	0,47935513	NA	-1,61349146	0,35721774	NA	0,00021669	NA	0,46315983	-0,13737061	10	-18967,5965	37955,2051	118,788422	5,26E-13
1407	0,61699723	NA	0,00084798	3,94E-05	-0,00164748	0,4880593	-0,22142621	-2,09152745	0,35956015	NA	0,00022076	NA	0,43622845	NA	11	-18967,0466	37956,1079	119,691144	3,35E-13
3933	-0,31227401	NA	NA	4,32E-05	-0,00145314	0,47935513	NA	-1,71083893	0,37612338	NA	0,00021703	0,00013898	0,46389079	-0,13469622	11	-18967,1232	37956,2611	119,844355	3,10E-13
1919	0,61699723	NA	0,00084798	3,94E-05	-0,00164748	0,4880593	-0,22142621	-2,2103983	0,38500536	NA	0,00022113	0,00018745	0,43806177	NA	12	-18966,1726	37956,3624	119,945678	2,95E-13
3423	-0,39160679	NA	0,00183718	4,30E-05	-0,00146197	0,48040455	NA	-1,61349146	0,35721774	NA	0,00021669	NA	0,46315983	-0,13737061	11	-18967,5531	37957,1208	120,704034	2,02E-13
2558	-0,06671637	0,57575795	NA	4,34E-05	-0,00071955	0,41051829	-0,17478186	-2,55577604	0,44288659	0,01897716	0,00021141	NA	NA	-0,08883356	12	-18966,5983	37957,2138	120,79707	1,93E-14
2560	-0,47166397	0,58945108	0,00876766	4,25E-05	-0,00073797	0,4135805	-0,1717464	-2,55577604	0,44288659	0,01897716	0,00021141	NA	NA	-0,08883356	13	-18965,6241	37957,2683	120,85157	1,88E-13
3935	-0,39160679	NA	0,00183718	4,30E-05	-0,00146197	0,48040455	NA	-1,71083893	0,37612338	NA	0,00021703	0,00013898	0,46389079	-0,13469622	12	-18967,0798	37958,1769	121,760188	1,19E-13
4014	-0,14195779	0,65649184	NA	4,36E-05	-0,00074998	NA	-0,15445798	-2,05206432	NA	0,01786019	0,00020271	-0,00039168	0,51529109	-0,14768431	12	-18967,2613	37958,5399	122,123176	9,93E-14
3070	-0,06671637	0,57575795	NA	4,34E-05	-0,00071955	0,41051829	-0,17478186	-2,61719209	0,45517288	0,01892094	0,00021164	9,21E-05	NA	-0,08713002	13	-18966,3927	37958,8056	122,388878	8,69E-14
3072	-0,47166397	0,58945108	0,00876766	4,25E-05	-0,00073797	0,4135805	-0,1717464	-2,61719209	0,45517288	0,01892094	0,00021164	9,21E-05	NA	-0,08713002	14	-18965,4185	37958,8603	122,4436	8,46E-14
4016	-0,45110821	0,66723944	0,00674107	4,29E-05	-0,00076432	NA	-0,15224001	-2,05206432	NA	0,01786019	0,00020271	-0,00039168	0,51529109	-0,14768431	13	-18966,7032	37959,4265	123,009732	6,37E-14
1846	-0,67953035	0,68234123	NA	4,46E-05	NA	0,41541375	-0,13795142	-1,78635429	NA	NA	0,0002119	-0,00029974	0,47515083	NA	10	-18969,9033	37959,8188	123,402088	5,24E-14
1848	-1,04079715	0,69637088	0,00755047	4,39E-05	NA	0,41816409	-0,13461471	-1,78635429	NA	NA	0,0002119	-0,00029974	0,47515083	NA	11	-18969,1841	37960,3828	123,966031	3,95E-14
1824	-1,28042467	0,61751101	0,00965662	4,55E-05	-0,00054807	0,40424516	NA	-1,78635429	NA	NA	0,0002119	-0,00029974	0,47515083	NA	11	-18969,9887	37961,9921	125,575361	1,77E-14
1822	-0,85074914	0,60334436	NA	4,65E-05	-0,00052401	0,40049867	NA	-1,78635429	NA	NA	0,0002119	-0,00029974	0,47515083	NA	10	-18971,1646	37962,3413	125,924597	1,48E-14
1334	-0,67953035	0,68234123	NA	4,46E-05	NA	0,41541375	-0,13795142	-1,96172815	NA	NA	0,00021146	NA	0,48287384	NA	9	-18972,5082	37963,0264	126,609661	1,05E-14
510	-0,06671637	0,57575795	NA	4,34E-05	-0,00071955	0,41051829	-0,17478186	-2,87228819	0,44207022	0,01904584	0,00021405	NA	NA	NA	11	-18970,5525	37963,1196	126,702927	1,01E-14
512	-0,47166397	0,58945108	0,00876766	4,25E-05	-0,00073797	0,4135805	-0,1717464	-2,87228819	0,44207022	0,01904584	0,00021405	NA	NA	NA	12	-18969,5783	37963,1739	126,757206	9,79E-15
1336	-1,04079715	0,69637088	0,00755047	4,39E-05	NA	0,41816409	-0,13461471	-1,96172815	NA	NA	0,00021146	NA	0,48287384	NA	10	-18971,789	37963,5901	127,173382	7,95E-15
1022	-0,06671637	0,57575795	NA	4,34E-05	-0,00071955	0,41051829	-0,17478186	-2,9468992	0,4586198	0,01897353	0,00021429	0,00012376	NA	NA	12	-18970,1771	37964,3715	127,954825	5,38E-15
1024	-0,47166397	0,58945108	0,00876766	4,25E-05	-0,00073797	0,4135805	-0,1717464	-2,9468992	0,4586198	0,01897353	0,00021429	0,00012376	NA	NA	13	-18969,203	37964,426	128,009326	5,23E-15
1312	-1,28042467	0,61751101	0,00965662	4,55E-05	-0,00054807	0,40424516	NA	-1,96172815	NA	NA	0,00021146	NA	0,48287384	NA	10	-18972,5936	37965,1994	128,782713	3,55E-15
3502	-0,14195779	0,65649184	NA	4,36E-05	-0,00074998	NA	-0,15445798	-2,27275384	NA	0,01711404	0,00020255	NA	0,52288611	-0,14000571	11	-18971,602	37965,2187	128,801957	3,52E-15
1310	-0,85074914	0,60334436	NA	4,65E-05	-0,00052401	0,40049867	NA	-1,96172815	NA	NA	0,00021146	NA	0,48287384	NA	9	-18973,7695	37965,5489	129,132171	2,98E-15
2550	-0,67953035	0,68234123	NA	4,46E-05	NA	0,41541375	-0,13795142	-2,55577604	0,44288659	0,01897716	0,00021141	NA	NA	-0,08883356	11	-18971,7849	37965,5844	129,16764	2,93E-15
3504	-0,45110821	0,66723944	0,00674107	4,29E-05	-0,00076432	NA	-0,15224001	-2,27275384	NA	0,01711404	0,00020255	NA	0,52288611	-0,14000571	12	-18971,0439	37966,105	129,688292	2,26E-15
2552	-1,04079715	0,69637088	0,00755047	4,39E-05	NA	0,41816409	-0,13461471	-2,55577604	0,44288659	0,01897716	0,00021141	NA	NA	-0,08883356	12	-18971,0656	37966,1485	129,731804	2,21E-15
1814	-1,1987332	0,68175498	NA	4,69E-05	NA	0,40581671	NA	-1,78635429	NA	NA	0,0002119	-0,00029974	0,47515083	NA	9	-18974,0785	37966,1669	129,750142	2,19E-15
1816	-1,59179834	0,69737855	0,00852736	4,60E-05	NA	0,40932129	NA	-1,78635429	NA	NA	0,0002119	-0,00029974	0,47515083	NA	10	-18973,162	37966,3362	129,919512	2,01E-15
3982	-0,8455927	0,67951403	NA	4,64E-05	-0,00057321	NA	NA	-2,05206432	NA	0,01786019	0,00020271	-0,00039168	0,51529109	-0,14768431	11	-18972,2818	37966,5782	130,161465	1,78E-15
3062	-0,67953035	0,68234123	NA	4,46E-05	NA	0,41541375	-0,13795142	-2,61719209	0,45517288	0,01892094	0,00021164	9,21E-05	NA	-0,08713002	12	-18971,5793	37967,1759	130,759226	1,32E-15
3984	-1,17938783	0,69098467	0,00751019	4,56E-05	-0,00059207	NA	NA	-2,05206432	NA	0,01786019	0,00020271	-0,00039168	0,51529109	-0,14768431	12	-18971,5918	37967,2009	130,784222	1,31E-16
4006	-0,79586264	0,77122371	NA	4,50E-05	NA	NA	-0,11503973	-2,05206432	NA	0,01786019	0,00020271	-0,00039168	0,51529109	-0,14768431	11	-18972,8555	37967,7256	131,308894	1,01E-15
3064	-1,04079715	0,69637088	0,00755047	4,39E-05	NA	0,41816409	-0,13461471	-2,61719209	0,45517288	0,01892094	0,00021164	9,21E-05	NA	-0,08713002	13	-18970,8601	37967,7403	131,323612	9,98E-16
2528	-1,28042467	0,61751101	0,00965662	4,55E-05	-0,00054807	0,40424516	NA	-2,55577604	0,44288659	0,01897716	0,00021141	NA	NA	-0,08883356	12	-18971,8703	37967,7579	131,341135	9,89E-17
2526	-0,85074914	0,60334436	NA	4,65E-05	-0,00052401	0,40049867	NA	-2,55577604	0,44288659	0,01897716	0,00021141	NA	NA	-0,08883356	11	-18973,0461	37968,1069	131,690149	8,31E-16

4008	-1,05409025	0,78157333	0,00543311	4,44E-05	NA	NA	-0,11273577	-2,05206432	NA	0,01786019	0,00020271	-0,00039168	0,51529109	-0,14768431	12	-18972,4952	37969,0077	132,590972	5,29E-16
3040	-1,28042467	0,61751101	0,00965662	4,55E-05	-0,00054807	0,40424516	NA	-2,61719209	0,45517288	0,01892094	0,00021164	9,21E-05	NA	-0,08713002	13	-18971,6648	37969,3497	132,932943	4,46E-16
1302	-1,1987332	0,68175498	NA	4,69E-05	NA	0,40581671	NA	-1,96172815	NA	NA	0,00021146	NA	0,48287384	NA	8	-18976,6833	37969,3747	132,957937	4,41E-16
1304	-1,59179834	0,69737855	0,00852736	4,60E-05	NA	0,40932129	NA	-1,96172815	NA	NA	0,00021146	NA	0,48287384	NA	9	-18975,7669	37969,5438	133,127085	4,05E-18
3038	-0,85074914	0,60334436	NA	4,65E-05	-0,00052401	0,40049867	NA	-2,61719209	0,45517288	0,01892094	0,00021164	9,21E-05	NA	-0,08713002	12	-18972,8406	37969,6985	133,281736	3,75E-16
502	-0,67953035	0,68234123	NA	4,46E-05	NA	0,41541375	-0,13795142	-2,87228819	0,44207022	0,01904584	0,00021405	NA	NA	NA	10	-18975,7391	37971,4904	135,073719	1,53E-16
3974	-1,235734	0,76857786	NA	4,69E-05	NA	NA	NA	-2,05206432	NA	0,01786019	0,00020271	-0,00039168	0,51529109	-0,14768431	10	-18975,7541	37971,5205	135,103745	1,51E-16
1373	-0,31227401	NA	NA	4,32E-05	-0,00145314	0,47935513	NA	-2,09152745	0,35956015	NA	0,00022076	NA	0,43622845	NA	9	-18976,9588	37971,9275	135,51078	1,23E-16
2518	-1,1987332	0,68175498	NA	4,69E-05	NA	0,40581671	NA	-2,55577604	0,44288659	0,01897716	0,00021141	NA	NA	-0,08883356	10	-18975,96	37971,9322	135,515472	1,23E-16
504	-1,04079715	0,69637088	0,00755047	4,39E-05	NA	0,41816409	-0,13461471	-2,87228819	0,44207022	0,01904584	0,00021405	NA	NA	NA	11	-18975,0199	37972,0544	135,637662	1,15E-16
2520	-1,59179834	0,69737855	0,00852736	4,60E-05	NA	0,40932129	NA	-2,55577604	0,44288659	0,01897716	0,00021141	NA	NA	-0,08883356	11	-18975,0436	37972,1018	135,685064	1,13E-16
1885	-0,31227401	NA	NA	4,32E-05	-0,00145314	0,47935513	NA	-2,2103983	0,38500536	NA	0,00022113	0,00018745	0,43806177	NA	10	-18976,0847	37972,1816	135,764871	1,08E-16
3976	-1,52051301	0,78035551	0,00624724	4,63E-05	NA	NA	NA	-2,05206432	NA	0,01786019	0,00020271	-0,00039168	0,51529109	-0,14768431	11	-18975,2781	37972,5707	136,153997	8,92E-17
1014	-0,67953035	0,68234123	NA	4,46E-05	NA	0,41541375	-0,13795142	-2,9468992	0,4586198	0,01897353	0,00021429	0,00012376	NA	NA	11	-18975,3638	37972,7421	136,325395	8,18E-17
3470	-0,8455927	0,67951403	NA	4,64E-05	-0,00057321	NA	NA	-2,27275384	NA	0,01711404	0,00020255	NA	0,52288611	-0,14000571	10	-18976,6225	37973,2572	136,840468	6,33E-17
1016	-1,04079715	0,69637088	0,00755047	4,39E-05	NA	0,41816409	-0,13461471	-2,9468992	0,4586198	0,01897353	0,00021429	0,00012376	NA	NA	12	-18974,6445	37973,3063	136,889559	6,17E-17
3030	-1,1987332	0,68175498	NA	4,69E-05	NA	0,40581671	NA	-2,61719209	0,45517288	0,01892094	0,00021164	9,21E-05	NA	-0,08713002	11	-18975,7545	37973,5236	137,106837	5,54E-17
480	-1,28042467	0,61751101	0,00965662	4,55E-05	-0,00054807	0,40424516	NA	-2,87228819	0,44207022	0,01904584	0,00021405	NA	NA	NA	11	-18975,8246	37973,6637	137,246992	5,16E-17
3032	-1,59179834	0,69737855	0,00852736	4,60E-05	NA	0,40932129	NA	-2,61719209	0,45517288	0,01892094	0,00021164	9,21E-05	NA	-0,08713002	12	-18974,8381	37973,6934	137,27665	5,09E-17
1375	-0,39160679	NA	0,00183718	4,30E-05	-0,00146197	0,48040455	NA	-2,09152745	0,35956015	NA	0,00022076	NA	0,43622845	NA	10	-18976,9154	37973,8429	137,42617	4,72E-17
3472	-1,17938783	0,69098467	0,00751019	4,56E-05	-0,00059207	NA	NA	-2,27275384	NA	0,01711404	0,00020255	NA	0,52288611	-0,14000571	11	-18975,9326	37973,8797	137,463003	4,63E-18
478	-0,85074914	0,60334436	NA	4,65E-05	-0,00052401	0,40049867	NA	-2,87228819	0,44207022	0,01904584	0,00021405	NA	NA	NA	10	-18977,0004	37974,013	137,596229	4,33E-17
1887	-0,39160679	NA	0,00183718	4,30E-05	-0,00146197	0,48040455	NA	-2,2103983	0,38500536	NA	0,00022113	0,00018745	0,43806177	NA	11	-18976,0413	37974,0972	137,680483	4,16E-17
3494	-0,79586264	0,77122371	NA	4,50E-05	NA	NA	-0,11503973	-2,27275384	NA	0,01711404	0,00020255	NA	0,52288611	-0,14000571	10	-18977,1962	37974,4046	137,987897	3,56E-17
992	-1,28042467	0,61751101	0,00965662	4,55E-05	-0,00054807	0,40424516	NA	-2,9468992	0,4586198	0,01897353	0,00021429	0,00012376	NA	NA	12	-18975,4492	37974,9156	138,49889	2,76E-17
990	-0,85074914	0,60334436	NA	4,65E-05	-0,00052401	0,40049867	NA	-2,9468992	0,4586198	0,01897353	0,00021429	0,00012376	NA	NA	11	-18976,625	37975,2646	138,847905	2,32E-17
3565	0,64576877	NA	NA	3,88E-05	-0,00183092	NA	-0,20438761	-2,59012234	0,38593256	0,02081194	0,00021148	NA	0,47514204	-0,138389	11	-18976,6304	37975,2754	138,858633	2,31E-17
3496	-1,05409025	0,78157333	0,00543311	4,44E-05	NA	NA	-0,11273577	-2,27275384	NA	0,01711404	0,00020255	NA	0,52288611	-0,14000571	11	-18976,8359	37975,6865	139,269754	1,88E-17
3573	-0,84339357	NA	NA	4,07E-05	NA	0,55505772	-0,13640315	-2,59012234	0,38593256	0,02081194	0,00021148	NA	0,47514204	-0,138389	11	-18976,8886	37975,7918	139,375066	1,78E-18
3575	-0,50593593	NA	-0,00700816	4,14E-05	NA	0,55021643	-0,14061875	-2,59012234	0,38593256	0,02081194	0,00021148	NA	0,47514204	-0,138389	12	-18976,2555	37976,5283	140,1116	1,23E-17
4077	0,64576877	NA	NA	3,88E-05	-0,00183092	NA	-0,20438761	-2,66976045	0,40191971	0,02073197	0,0002118	0,00011864	0,47565984	-0,13600353	12	-18976,2884	37976,594	140,177287	1,19E-17
4085	-0,84339357	NA	NA	4,07E-05	NA	0,55505772	-0,13640315	-2,66976045	0,40191971	0,02073197	0,0002118	0,00011864	0,47565984	-0,13600353	12	-18976,5466	37977,1104	140,69372	9,21E-18
3567	0,74969745	NA	-0,00245756	3,91E-05	-0,00181949	NA	-0,20513804	-2,59012234	0,38593256	0,02081194	0,00021148	NA	0,47514204	-0,138389	12	-18976,5548	37977,1269	140,710139	9,14E-18
470	-1,1987332	0,68175498	NA	4,69E-05	NA	0,40581671	NA	-2,87228819	0,44207022	0,01904584	0,00021405	NA	NA	NA	9	-18979,9143	37977,8385	141,421773	6,40E-19
4087	-0,50593593	NA	-0,00700816	4,14E-05	NA	0,55021643	-0,14061875	-2,66976045	0,40191971	0,02073197	0,0002118	0,00011864	0,47565984	-0,13600353	13	-18975,9135	37977,8472	141,430476	6,37E-18
472	-1,59179834	0,69737855	0,00852736	4,60E-05	NA	0,40932129	NA	-2,87228819	0,44207022	0,01904584	0,00021405	NA	NA	NA	10	-18978,9978	37978,0079	141,591143	5,88E-18
3462	-1,235734	0,76857786	NA	4,69E-05	NA	NA	NA	-2,27275384	NA	0,01711404	0,00020255	NA	0,52288611	-0,14000571	9	-18980,0949	37978,1997	141,78297	5,34E-18
1966	-0,14195779	0,65649184	NA	4,36E-05	-0,00074998	NA	-0,15445798	-2,58652225	NA	0,01772679	0,00020696	-0,00034396	0,48679163	NA	11	-18978,2071	37978,4288	142,012061	4,77E-18
4079	0,74969745	NA	-0,00245756	3,91E-05	-0,00181949	NA	-0,20513804	-2,66976045	0,40191971	0,02073197	0,0002118	0,00011864	0,47565984	-0,13600353	13	-18976,2128	37978,4457	142,029015	4,73E-18
982	-1,1987332	0,68175498	NA	4,69E-05	NA	0,40581671	NA	-2,9468992	0,4586198	0,01897353	0,00021429	0,00012376	NA	NA	10	-18979,5389	37979,0899	142,673228	3,42E-18
4029	0,65451489	NA	NA	3,95E-05	-0,00164357	0,48762971	-0,22170047	-2,05206432	NA	0,01786019	0,00020271	-0,00039168	0,51529109	-0,14768431	12	-18977,5594	37979,1361	142,719365	3,35E-18
3464	-1,52051301	0,78035551	0,00624724	4,63E-05	NA	NA	NA	-2,27275384	NA	0,01711404	0,00020255	NA	0,52288611	-0,14000571	10	-18979,6188	37979,2497	142,833	3,16E-18
984	-1,59179834	0,69737855	0,00852736	4,60E-05	NA	0,40932129	NA	-2,9468992	0,4586198	0,01897353	0,00021429	0,00012376	NA	NA	11	-18978,6225	37979,2595	142,842819	3,15E-18
1968	-0,45110821	0,66723944	0,00674107	4,29E-05	-0,00076432	NA	-0,15224001	-2,58652225	NA	0,01772679	0,00020696	-0,00034396	0,48679163	NA	12	-18977,6489	37979,3151	142,898395	3,06E-18
3541	-1,36352414	NA	NA	4,29E-05	NA	0,54467715	NA	-2,59012234	0,38593256	0,02081194	0,00021148	NA	0,47514204	-0,138389	10	-18980,429	37980,8701	144,453391	1,41E-18
4031	0,61699723	NA	0,00084798	3,94E-05	-0,00164748	0,4880593	-0,22142621	-2,05206432	NA	0,01786019	0,00020271	-0,00039168	0,51529109	-0,14768431	13	-18977,5501	37981,1204	144,703653	1,24E-18
3886	-0,14195779	0,65649184	NA	4,36E-05	-0,00074998	NA	-0,15445798	-1,25486087	NA	NA	0,00020772	-0,0003441	0,50313521	-0,14558566	11	-18979,5641	37981,1428	144,726076	1,23E-18
3543	-1,08960867	NA	-0,00576757	4,36E-05	NA	0,53973144	NA	-2,59012234	0,38593256	0,02081194	0,00021148	NA	0,47514204	-0,138389	11	-18979,9967	37982,008	145,591248	7,96E-21
3888	-0,45110821	0,66723944	0,00674107	4,29E-05	-0,00076432	NA	-0,15224001	-1,25486087	NA	NA	0,00020772	-0,0003441	0,50313521	-0,14558566	12	-18979,0059	37982,0291	145,61241	7,88E-19
4053	-1,36352414	NA	NA	4,29E-05	NA	0,54467715	NA	-2,66976045	0,40191971	0,02073197	0,0002118	0,00011864	0,47565984	-0,13600353	11	-18980,087	37982,1885	145,771824	7,27E-19
2430	-0,06671637	0,57575795	NA	4,34E-05	-0,00071955	0,41051829	-0,17478186	-1,66433243	0,4162362	NA	0,00021617	NA	NA	-0,08904476	11	-18980,4798	37982,9742	146,557453	4,91E-19
2432	-0,47166397	0,58945108	0,00876766	4,25E-05	-0,00073797	0,4135805	-0,1717464	-1,66433243	0,4162362	NA	0,00021617	NA	NA	-0,08904476	12	-18979,5056	37983,0285	146,611732	4,78E-20
1454	-0,14195779	0,65649184	NA	4,36E-05	-0,00074998	NA	-0,15445798	-2,75826159	NA	0,01709531	0,00020664	NA	0,4949792	NA	10	-18981,5955	37983,2033	146,78654	4,38E-19
4055	-1,08960867	NA	-0,00576757	4,36E-05	NA	0,53973144	NA	-2,66976045	0,40191971	0,02073197	0,0002118	0,00011864	0,47565984	-0,13600353	12	-18979,6547	37983,3266	146,909901	4,12E-19
1456	-0,45110821	0,66723944	0,00674107	4,29E-05	-0,00076432	NA	-0,15224001	-2,75826159	NA	0,01709531	0,00020664	NA	0,4949792	NA	11	-18981,0374	37984,0894	147,672653	2,81E-19
2942	-0,06671637	0,57575795	NA	4,34E-05	-0,00071955	0,41051829	-0,17478186	-1,73983563	0,43090563	NA	0,00021643	0,00010896	NA	-0,08710954	12	-18980,1901	37984,3974	147,98065	2,41E-19
2944	-0,47166397	0,58945108	0,00876766	4,25E-05	-0,00073797	0,4135805	-0,1717464	-1,73983563	0,43090563	NA	0,00021643	0,00010896	NA	-0,08710954	13	-18979,2159	37984,4519	148,035151	2,35E-19
3517	0,65451489	NA	NA	3,95E-05	-0,00164357	0,48762971	-0,22170047	-2,27275384	NA	0,01711404	0,00020255	NA	0,52288611	-0,14000571	11	-18981,9001	37985,8149	149,398147	1,19E-19
3374	-0,14195779	0,65649184	NA	4,36E-05	-0,00074998	NA	-0,15445798	-1,4783871	NA	NA	0,00020739	NA	0,51055393	-0,13912561	10	-18982,958	37985,9282	149,51149	1,12E-19
1934	-0,8455927	0,67951403	NA	4,64E-05	-0,00057321	NA	NA	-2,58652225	NA	0,01772679	0,00020696	-0,00034396	0,48679163	NA	10	-18983,2276	37986,4673	150,050572	8,56E-20
3376	-0,45110821	0,66723944	0,00674107	4,29E-05	-0,00076432	NA	-0,15224001	-1,4783871	NA	NA	0,00020739	NA	0,51055393	-0,13912561	11	-18982,3999	37986,8143	150,397603	7,20E-20

1936	-1,17938783	0,69098467	0,00751019	4,56E-05	-0,00059207	NA	NA	-2,58652225	NA	0,01772679	0,00020696	-0,00034396	0,48679163	NA	11	-18982,5376	37987,0898	150,673107	6,27E-20
1958	-0,79586264	0,77122371	NA	4,50E-05	NA	NA	-0,11503973	-2,58652225	NA	0,01772679	0,00020696	-0,00034396	0,48679163	NA	10	-18983,8013	37987,6147	151,198	4,82E-20
3519	0,61699723	NA	0,00084798	3,94E-05	-0,00164748	0,4880593	-0,22142621	-2,27275384	NA	0,01711404	0,00020255	NA	0,52288611	-0,14000571	12	-18981,8908	37987,7989	151,382214	4,40E-20
1960	-1,05409025	0,78157333	0,00543311	4,44E-05	NA	NA	-0,11273577	-2,58652225	NA	0,01772679	0,00020696	-0,00034396	0,48679163	NA	11	-18983,441	37988,8966	152,479858	2,54E-20
382	-0,06671637	0,57575795	NA	4,34E-05	-0,00071955	0,41051829	-0,17478186	-1,97841134	0,41538406	NA	0,00021885	NA	NA	NA	10	-18984,4709	37988,954	152,537283	2,47E-20
384	-0,47166397	0,58945108	0,00876766	4,25E-05	-0,00073797	0,4135805	-0,1717464	-1,97841134	0,41538406	NA	0,00021885	NA	NA	NA	11	-18983,4967	37989,0081	152,59134	2,40E-20
3854	-0,8455927	0,67951403	NA	4,64E-05	-0,00057321	NA	NA	-1,25486087	NA	NA	0,00020772	-0,0003441	0,50313521	-0,14558566	10	-18984,5846	37989,1813	152,764587	2,20E-20
3856	-1,17938783	0,69098467	0,00751019	4,56E-05	-0,00059207	NA	NA	-1,25486087	NA	NA	0,00020772	-0,0003441	0,50313521	-0,14558566	11	-18983,8946	37989,8038	153,387122	1,61E-20
3533	-0,28011525	NA	NA	4,24E-05	-0,0016491	NA	NA	-2,59012234	0,38593256	0,02081194	0,00021148	NA	0,47514204	-0,138389	10	-18984,9207	37989,8535	153,436811	1,57E-20
894	-0,06671637	0,57575795	NA	4,34E-05	-0,00071955	0,41051829	-0,17478186	-2,0661368	0,43415925	NA	0,00021911	0,00013912	NA	NA	11	-18983,993	37990,0006	153,583854	1,46E-20
896	-0,47166397	0,58945108	0,00876766	4,25E-05	-0,00073797	0,4135805	-0,1717464	-2,0661368	0,43415925	NA	0,00021911	0,00013912	NA	NA	12	-18983,0188	37990,0549	153,638133	1,42E-20
3878	-0,79586264	0,77122371	NA	4,50E-05	NA	NA	-0,11503973	-1,25486087	NA	NA	0,00020772	-0,0003441	0,50313521	-0,14558566	10	-18985,1583	37990,3287	153,912015	1,24E-20
4045	-0,28011525	NA	NA	4,24E-05	-0,0016491	NA	NA	-2,66976045	0,40191971	0,02073197	0,0002118	0,00011864	0,47565984	-0,13600353	11	-18984,5787	37991,172	154,755243	8,15E-21
1422	-0,8455927	0,67951403	NA	4,64E-05	-0,00057321	NA	NA	-2,75826159	NA	0,01709531	0,00020664	NA	0,4949792	NA	9	-18986,616	37991,242	154,825273	7,87E-21
2422	-0,67953035	0,68234123	NA	4,46E-05	NA	0,41541375	-0,13795142	-1,66433243	0,4162362	NA	0,00021617	NA	NA	-0,08904476	10	-18985,6664	37991,345	154,928244	7,47E-21
1926	-1,235734	0,76857786	NA	4,69E-05	NA	NA	NA	-2,58652225	NA	0,01772679	0,00020696	-0,00034396	0,48679163	NA	9	-18986,6999	37991,4098	154,993073	7,23E-21
3880	-1,05409025	0,78157333	0,00543311	4,44E-05	NA	NA	-0,11273577	-1,25486087	NA	NA	0,00020772	-0,0003441	0,50313521	-0,14558566	11	-18984,798	37991,6106	155,193873	6,54E-21
3535	-0,2131598	NA	-0,0015413	4,25E-05	-0,00164139	NA	NA	-2,59012234	0,38593256	0,02081194	0,00021148	NA	0,47514204	-0,138389	11	-18984,891	37991,7966	155,379857	5,96E-21
1424	-1,17938783	0,69098467	0,00751019	4,56E-05	-0,00059207	NA	NA	-2,75826159	NA	0,01709531	0,00020664	NA	0,4949792	NA	10	-18985,9261	37991,8643	155,447586	5,76E-23
2424	-1,04079715	0,69637088	0,00755047	4,39E-05	NA	0,41816409	-0,13461471	-1,66433243	0,4162362	NA	0,00021617	NA	NA	-0,08904476	11	-18984,9472	37991,9089	155,492187	5,64E-21
1517	0,64576877	NA	NA	3,88E-05	-0,00183092	NA	-0,20438761	-3,07214819	0,38813022	0,02082345	0,00021556	NA	0,44803127	NA	10	-18986,0844	37992,1809	155,764163	4,92E-21
1446	-0,79586264	0,77122371	NA	4,50E-05	NA	NA	-0,11503973	-2,75826159	NA	0,01709531	0,00020664	NA	0,4949792	NA	9	-18987,1897	37992,3894	155,972702	4,43E-21
1928	-1,52051301	0,78035551	0,00624724	4,63E-05	NA	NA	NA	-2,58652225	NA	0,01772679	0,00020696	-0,00034396	0,48679163	NA	10	-18986,2238	37992,4598	156,043104	4,28E-21
1525	-0,84339357	NA	NA	4,07E-05	NA	0,55505772	-0,13640315	-3,07214819	0,38813022	0,02082345	0,00021556	NA	0,44803127	NA	10	-18986,3426	37992,6973	156,280596	3,80E-21
2029	0,64576877	NA	NA	3,88E-05	-0,00183092	NA	-0,20438761	-3,17508673	0,41103069	0,02071909	0,00021591	0,0001702	0,4495725	NA	11	-18985,3695	37992,7536	156,336842	3,69E-21
2934	-0,67953035	0,68234123	NA	4,46E-05	NA	0,41541375	-0,13795142	-1,73983563	0,43090563	NA	0,00021643	0,00010896	NA	-0,08710954	11	-18985,3767	37992,7679	156,351221	3,67E-21
4047	-0,2131598	NA	-0,0015413	4,25E-05	-0,00164139	NA	NA	-2,66976045	0,40191971	0,02073197	0,0002118	0,00011864	0,47565984	-0,13600353	12	-18984,549	37993,1152	156,698511	3,08E-21
2037	-0,84339357	NA	NA	4,07E-05	NA	0,55505772	-0,13640315	-3,17508673	0,41103069	0,02071909	0,00021591	0,0001702	0,4495725	NA	11	-18985,6277	37993,27	156,853275	2,85E-21
2936	-1,04079715	0,69637088	0,00755047	4,39E-05	NA	0,41816409	-0,13461471	-1,73983563	0,43090563	NA	0,00021643	0,00010896	NA	-0,08710954	12	-18984,6574	37993,3321	156,915385	2,77E-22
1527	-0,50593593	NA	-0,00700816	4,14E-05	NA	0,55021643	-0,14061875	-3,07214819	0,38813022	0,02082345	0,00021556	NA	0,44803127	NA	11	-18985,7095	37993,4336	157,016908	2,63E-21
2400	-1,28042467	0,61751101	0,00965662	4,55E-05	-0,00054807	0,40424516	NA	-1,66433243	0,4162362	NA	0,00021617	NA	NA	-0,08904476	11	-18985,7518	37993,5182	157,101518	2,52E-21
1448	-1,05409025	0,78157333	0,00543311	4,44E-05	NA	NA	-0,11273577	-2,75826159	NA	0,01709531	0,00020664	NA	0,4949792	NA	10	-18986,8294	37993,6711	157,254337	2,33E-21
2398	-0,85074914	0,60334436	NA	4,65E-05	-0,00052401	0,40049867	NA	-1,66433243	0,4162362	NA	0,00021617	NA	NA	-0,08904476	10	-18986,9277	37993,8675	157,450754	2,12E-21
3342	-0,8455927	0,67951403	NA	4,64E-05	-0,00057321	NA	NA	-1,4783871	NA	NA	0,00020739	NA	0,51055393	-0,13912561	9	-18987,9785	37993,9669	157,550223	2,01E-21
2039	-0,50593593	NA	-0,00700816	4,14E-05	NA	0,55021643	-0,14061875	-3,17508673	0,41103069	0,02071909	0,00021591	0,0001702	0,4495725	NA	12	-18984,9946	37994,0065	157,589809	1,97E-21
1519	0,74969745	NA	-0,00245756	3,91E-05	-0,00181949	NA	-0,20513804	-3,07214819	0,38813022	0,02082345	0,00021556	NA	0,44803127	NA	11	-18986,0088	37994,0322	157,615447	1,95E-21
3846	-1,235734	0,76857786	NA	4,69E-05	NA	NA	NA	-1,25486087	NA	NA	0,00020772	-0,0003441	0,50313521	-0,14558566	9	-18988,0569	37994,1238	157,707088	1,86E-21
3344	-1,17938783	0,69098467	0,00751019	4,56E-05	-0,00059207	NA	NA	-1,4783871	NA	NA	0,00020739	NA	0,51055393	-0,13912561	10	-18987,2885	37994,5893	158,172536	1,48E-21
2031	0,74969745	NA	-0,00245756	3,91E-05	-0,00181949	NA	-0,20513804	-3,17508673	0,41103069	0,02071909	0,00021591	0,0001702	0,4495725	NA	12	-18985,2939	37994,6051	158,188348	1,46E-21
2912	-1,28042467	0,61751101	0,00965662	4,55E-05	-0,00054807	0,40424516	NA	-1,73983563	0,43090563	NA	0,00021643	0,00010896	NA	-0,08710954	12	-18985,4621	37994,9414	158,524715	1,24E-21
3366	-0,79586264	0,77122371	NA	4,50E-05	NA	NA	-0,11503973	-1,4783871	NA	NA	0,00020739	NA	0,51055393	-0,13912561	9	-18988,5522	37995,1144	158,697651	1,13E-21
3848	-1,52051301	0,78035551	0,00624724	4,63E-05	NA	NA	NA	-1,25486087	NA	NA	0,00020772	-0,0003441	0,50313521	-0,14558566	10	-18987,5808	37995,1738	158,757118	1,10E-21
2910	-0,85074914	0,60334436	NA	4,65E-05	-0,00052401	0,40049867	NA	-1,73983563	0,43090563	NA	0,00021643	0,00010896	NA	-0,08710954	11	-18986,6379	37995,2905	158,87373	1,04E-21
1414	-1,235734	0,76857786	NA	4,69E-05	NA	NA	NA	-2,75826159	NA	0,01709531	0,00020664	NA	0,4949792	NA	8	-18990,0884	37996,1847	159,767996	6,64E-22
3368	-1,05409025	0,78157333	0,00543311	4,44E-05	NA	NA	-0,11273577	-1,4783871	NA	NA	0,00020739	NA	0,51055393	-0,13912561	10	-18988,1919	37996,396	159,979287	5,98E-22
3997	-0,31227401	NA	NA	4,32E-05	-0,00145314	0,47935513	NA	-2,05206432	NA	0,01786019	0,00020271	-0,00039168	0,51529109	-0,14768431	11	-18987,4623	37996,9391	160,522403	4,56E-22
1416	-1,52051301	0,78035551	0,00624724	4,63E-05	NA	NA	NA	-2,75826159	NA	0,01709531	0,00020664	NA	0,4949792	NA	9	-18989,6123	37997,2345	160,817805	3,93E-22
374	-0,67953035	0,68234123	NA	4,46E-05	NA	0,41541375	-0,13795142	-1,97841134	0,41538406	NA	0,00021885	NA	NA	NA	9	-18989,6575	37997,325	160,908296	3,76E-22
2390	-1,1987332	0,68175498	NA	4,69E-05	NA	0,40581671	NA	-1,66433243	0,4162362	NA	0,00021617	NA	NA	-0,08904476	9	-18989,8415	37997,693	161,276299	3,13E-22
1493	-1,36352414	NA	NA	4,29E-05	NA	0,54467715	NA	-3,07214819	0,38813022	0,02082345	0,00021556	NA	0,44803127	NA	9	-18989,8829	37997,7759	161,359142	3,00E-22
2392	-1,59179834	0,69737855	0,00852736	4,60E-05	NA	0,40932129	NA	-1,66433243	0,4162362	NA	0,00021617	NA	NA	-0,08904476	10	-18988,9251	37997,8624	161,445668	2,87E-22
376	-1,04079715	0,69637088	0,00755047	4,39E-05	NA	0,41816409	-0,13461471	-1,97841134	0,41538406	NA	0,00021885	NA	NA	NA	10	-18988,9383	37997,8887	161,472017	2,83E-22
2005	-1,36352414	NA	NA	4,29E-05	NA	0,54467715	NA	-3,17508673	0,41103069	0,02071909	0,00021591	0,0001702	0,4495725	NA	10	-18989,1681	37998,3483	161,9316	2,25E-24
886	-0,67953035	0,68234123	NA	4,46E-05	NA	0,41541375	-0,13795142	-2,0661368	0,43415925	NA	0,00021911	0,00013912	NA	NA	10	-18989,1796	37998,3714	161,954645	2,23E-23
3999	-0,39160679	NA	0,00183718	4,30E-05	-0,00146197	0,48040455	NA	-2,05206432	NA	0,01786019	0,00020271	-0,00039168	0,51529109	-0,14768431	12	-18987,4188	37998,855	162,438236	1,75E-22
3334	-1,235734	0,76857786	NA	4,69E-05	NA	NA	NA	-1,4783871	NA	NA	0,00020739	NA	0,51055393	-0,13912561	8	-18991,4509	37998,9097	162,492946	1,70E-22
1495	-1,08960867	NA	-0,00576757	4,36E-05	NA	0,53973144	NA	-3,07214819	0,38813022	0,02082345	0,00021556	NA	0,44803127	NA	10	-18989,4507	37998,9135	162,496777	1,70E-22
888	-1,04079715	0,69637088	0,00755047	4,39E-05	NA	0,41816409	-0,13461471	-2,0661368	0,43415925	NA	0,00021911	0,00013912	NA	NA	11	-18988,4604	37998,9353	162,518588	1,68E-22
1981	0,65451489	NA	NA	3,95E-05	-0,00164357	0,48762971	-0,22170047	-2,58652225	NA	0,01772679	0,00020696	-0,00034396	0,48679163	NA	11	-18988,5052	37999,025	162,60825	1,61E-22
2902	-1,1987332	0,68175498	NA	4,69E-05	NA	0,40581671	NA	-1,73983563	0,43090563	NA	0,00021643	0,00010896	NA	-0,08710954	10	-18989,5518	37999,1158	162,699053	1,53E-22
2904	-1,59179834	0,69737855	0,00852736	4,60E-05	NA	0,40932129	NA	-1,73983563	0,43090563	NA	0,00021643	0,00010896	NA	-0,08710954	11	-18988,6354	37999,2854	162,868644	1,41E-22

2007	-1,08960867	NA	-0,00576757	4,36E-05	NA	0,53973144	NA	-3,17508673	0,41103069	0,02071909	0,00021591	0,0001702	0,4495725	NA	11	-18988,7358	37999,4862	163,069457	1,28E-22
352	-1,28042467	0,61751101	0,00965662	4,55E-05	-0,00054807	0,40424516	NA	-1,97841134	0,41538406	NA	0,00021885	NA	NA	NA	10	-18989,743	37999,4981	163,081348	1,27E-22
350	-0,85074914	0,60334436	NA	4,65E-05	-0,00052401	0,40049867	NA	-1,97841134	0,41538406	NA	0,00021885	NA	NA	NA	9	-18990,9188	37999,8475	163,430806	1,06E-22
3336	-1,52051301	0,78035551	0,00624724	4,63E-05	NA	NA	NA	-1,4783871	NA	NA	0,00020739	NA	0,51055393	-0,13912561	9	-18990,9748	37999,9595	163,542755	1,01E-22
1838	-0,14195779	0,65649184	NA	4,36E-05	-0,00074998	NA	-0,15445798	-1,78635429	NA	NA	0,0002119	-0,00029974	0,47515083	NA	10	-18990,2459	38000,5039	164,087198	7,67E-23
864	-1,28042467	0,61751101	0,00965662	4,55E-05	-0,00054807	0,40424516	NA	-2,0661368	0,43415925	NA	0,00021911	0,00013912	NA	NA	11	-18989,265	38000,5446	164,127919	7,51E-23
862	-0,85074914	0,60334436	NA	4,65E-05	-0,00052401	0,40049867	NA	-2,0661368	0,43415925	NA	0,00021911	0,00013912	NA	NA	10	-18990,4409	38000,8939	164,477155	6,31E-23
1983	0,61699723	NA	0,00084798	3,94E-05	-0,00164748	0,4880593	-0,22142621	-2,58652225	NA	0,01772679	0,00020696	-0,00034396	0,48679163	NA	12	-18988,4959	38001,009	164,592317	5,95E-23
1840	-0,45110821	0,66723944	0,00674107	4,29E-05	-0,00076432	NA	-0,15224001	-1,78635429	NA	NA	0,0002119	-0,00029974	0,47515083	NA	11	-18989,6877	38001,39	164,973311	4,92E-23
3901	0,65451489	NA	NA	3,95E-05	-0,00164357	0,48762971	-0,22170047	-1,25486087	NA	NA	0,00020772	-0,0003441	0,50313521	-0,14558566	11	-18989,8622	38001,739	165,322265	4,13E-23
3485	-0,31227401	NA	NA	4,32E-05	-0,00145314	0,47935513	NA	-2,27275384	NA	0,01711404	0,00020255	NA	0,52288611	-0,14000571	10	-18991,803	38003,6181	167,201406	1,62E-23
342	-1,1987332	0,68175498	NA	4,69E-05	NA	0,40581671	NA	-1,97841134	0,41538406	NA	0,00021885	NA	NA	NA	8	-18993,8327	38003,6733	167,256572	1,57E-23
1326	-0,14195779	0,65649184	NA	4,36E-05	-0,00074998	NA	-0,15445798	-1,96172815	NA	NA	0,00021146	NA	0,48287384	NA	9	-18992,8508	38003,7115	167,294771	1,54E-23
3903	0,61699723	NA	0,00084798	3,94E-05	-0,00164748	0,4880593	-0,22142621	-1,25486087	NA	NA	0,00020772	-0,0003441	0,50313521	-0,14558566	12	-18989,8529	38003,7231	167,306332	1,53E-23
1469	0,65451489	NA	NA	3,95E-05	-0,00164357	0,48762971	-0,22170047	-2,75826159	NA	0,01709531	0,00020664	NA	0,4949792	NA	10	-18991,8936	38003,7995	167,38273	1,48E-23
344	-1,59179834	0,69737855	0,00852736	4,60E-05	NA	0,40932129	NA	-1,97841134	0,41538406	NA	0,00021885	NA	NA	NA	9	-18992,9162	38003,8424	167,42572	1,44E-23
1328	-0,45110821	0,66723944	0,00674107	4,29E-05	-0,00076432	NA	-0,15224001	-1,96172815	NA	NA	0,00021146	NA	0,48287384	NA	10	-18992,2926	38004,5974	168,180663	9,90E-24
854	-1,1987332	0,68175498	NA	4,69E-05	NA	0,40581671	NA	-2,0661368	0,43415925	NA	0,00021911	0,00013912	NA	NA	9	-18993,3547	38004,7194	168,3027	9,31E-24
856	-1,59179834	0,69737855	0,00852736	4,60E-05	NA	0,40932129	NA	-2,0661368	0,43415925	NA	0,00021911	0,00013912	NA	NA	10	-18992,4383	38004,8888	168,472069	8,56E-24
3487	-0,39160679	NA	0,00183718	4,30E-05	-0,00146197	0,48040455	NA	-2,27275384	NA	0,01711404	0,00020255	NA	0,52288611	-0,14000571	11	-18991,7596	38005,5337	169,117018	6,20E-24
1471	0,61699723	NA	0,00084798	3,94E-05	-0,00164748	0,4880593	-0,22142621	-2,75826159	NA	0,01709531	0,00020664	NA	0,4949792	NA	11	-18991,8843	38005,7833	169,366575	5,47E-24
2542	-0,14195779	0,65649184	NA	4,36E-05	-0,00074998	NA	-0,15445798	-2,55577604	0,44288659	0,01897716	0,00021141	NA	NA	-0,08883356	11	-18992,1274	38006,2695	169,85275	4,29E-24
3389	0,65451489	NA	NA	3,95E-05	-0,00164357	0,48762971	-0,22170047	-1,4783871	NA	NA	0,00020739	NA	0,51055393	-0,13912561	10	-18993,2561	38006,5244	170,10768	3,78E-25
3437	0,64576877	NA	NA	3,88E-05	-0,00183092	NA	-0,20438761	-1,61349146	0,35721774	NA	0,00021669	NA	0,46315983	-0,13737061	10	-18993,2985	38006,6091	170,19237	3,62E-24
1485	-0,28011525	NA	NA	4,24E-05	-0,0016491	NA	NA	-3,07214819	0,38813022	0,02082345	0,00021556	NA	0,44803127	NA	9	-18994,3747	38006,7593	170,342562	3,36E-24
3445	-0,84339357	NA	NA	4,07E-05	NA	0,55505772	-0,13640315	-1,61349146	0,35721774	NA	0,00021669	NA	0,46315983	-0,13737061	10	-18993,5567	38007,1255	170,708803	2,80E-24
2544	-0,45110821	0,66723944	0,00674107	4,29E-05	-0,00076432	NA	-0,15224001	-2,55577604	0,44288659	0,01897716	0,00021141	NA	NA	-0,08883356	12	-18991,5693	38007,1558	170,739085	2,75E-24
1997	-0,28011525	NA	NA	4,24E-05	-0,0016491	NA	NA	-3,17508673	0,41103069	0,02071909	0,00021591	0,0001702	0,4495725	NA	10	-18993,6598	38007,3317	170,91502	2,52E-24
3949	0,64576877	NA	NA	3,88E-05	-0,00183092	NA	-0,20438761	-1,71083893	0,37612338	NA	0,00021703	0,00013898	0,46389079	-0,13469622	11	-18992,8252	38007,665	171,248303	2,14E-24
3054	-0,14195779	0,65649184	NA	4,36E-05	-0,00074998	NA	-0,15445798	-2,61719209	0,45517288	0,01892094	0,00021164	9,21E-05	NA	-0,08713002	12	-18991,9219	38007,8611	171,444336	1,94E-24
3447	-0,50593593	NA	-0,00700816	4,14E-05	NA	0,55021643	-0,14061875	-1,61349146	0,35721774	NA	0,00021669	NA	0,46315983	-0,13737061	11	-18992,9236	38007,8618	171,445115	1,94E-24
3957	-0,84339357	NA	NA	4,07E-05	NA	0,55505772	-0,13640315	-1,71083893	0,37612338	NA	0,00021703	0,00013898	0,46389079	-0,13469622	11	-18993,0834	38008,1815	171,764735	1,65E-24
3439	0,74969745	NA	-0,00245756	3,91E-05	-0,00181949	NA	-0,20513804	-1,61349146	0,35721774	NA	0,00021669	NA	0,46315983	-0,13737061	11	-18993,2229	38008,4604	172,043655	1,43E-24
3391	0,61699723	NA	0,00084798	3,94E-05	-0,00164748	0,4880593	-0,22142621	-1,4783871	NA	NA	0,00020739	NA	0,51055393	-0,13912561	11	-18993,2468	38008,5082	172,091525	1,40E-24
1806	-0,8455927	0,67951403	NA	4,64E-05	-0,00057321	NA	NA	-1,78635429	NA	NA	0,0002119	-0,00029974	0,47515083	NA	9	-18995,2663	38008,5427	172,125931	1,38E-25
1487	-0,2131598	NA	-0,0015413	4,25E-05	-0,00164139	NA	NA	-3,07214819	0,38813022	0,02082345	0,00021556	NA	0,44803127	NA	10	-18994,345	38008,7021	172,285387	1,27E-24
3056	-0,45110821	0,66723944	0,00674107	4,29E-05	-0,00076432	NA	-0,15224001	-2,61719209	0,45517288	0,01892094	0,00021164	9,21E-05	NA	-0,08713002	13	-18991,3637	38008,7476	172,330892	1,24E-24
3959	-0,50593593	NA	-0,00700816	4,14E-05	NA	0,55021643	-0,14061875	-1,71083893	0,37612338	NA	0,00021703	0,00013898	0,46389079	-0,13469622	12	-18992,4504	38008,918	172,501269	1,14E-24
1808	-1,17938783	0,69098467	0,00751019	4,56E-05	-0,00059207	NA	NA	-1,78635429	NA	NA	0,0002119	-0,00029974	0,47515083	NA	10	-18994,5764	38009,165	172,748244	1,01E-24
1999	-0,2131598	NA	-0,0015413	4,25E-05	-0,00164139	NA	NA	-3,17508673	0,41103069	0,02071909	0,00021591	0,0001702	0,4495725	NA	11	-18993,6301	38009,2748	172,858066	9,55E-25
3951	0,74969745	NA	-0,00245756	3,91E-05	-0,00181949	NA	-0,20513804	-1,71083893	0,37612338	NA	0,00021703	0,00013898	0,46389079	-0,13469622	12	-18992,7496	38009,5165	173,099809	8,46E-25
1830	-0,79586264	0,77122371	NA	4,50E-05	NA	NA	-0,11503973	-1,78635429	NA	NA	0,0002119	-0,00029974	0,47515083	NA	9	-18995,8401	38009,6901	173,273359	7,76E-25
1832	-1,05409025	0,78157333	0,00543311	4,44E-05	NA	NA	-0,11273577	-1,78635429	NA	NA	0,0002119	-0,00029974	0,47515083	NA	10	-18995,4798	38010,9717	174,554995	4,09E-25
1294	-0,8455927	0,67951403	NA	4,64E-05	-0,00057321	NA	NA	-1,96172815	NA	NA	0,00021146	NA	0,48287384	NA	8	-18997,8712	38011,7504	175,333726	2,77E-25
494	-0,14195779	0,65649184	NA	4,36E-05	-0,00074998	NA	-0,15445798	-2,87228819	0,44207022	0,01904584	0,00021405	NA	NA	NA	10	-18996,0817	38012,1756	175,758829	2,24E-25
3413	-1,36352414	NA	NA	4,29E-05	NA	0,54467715	NA	-1,61349146	0,35721774	NA	0,00021669	NA	0,46315983	-0,13737061	9	-18997,0971	38012,2041	175,78735	2,21E-25
1296	-1,17938783	0,69098467	0,00751019	4,56E-05	-0,00059207	NA	NA	-1,96172815	NA	NA	0,00021146	NA	0,48287384	NA	9	-18997,1813	38012,3725	175,955817	2,03E-25
1318	-0,79586264	0,77122371	NA	4,50E-05	NA	NA	-0,11503973	-1,96172815	NA	NA	0,00021146	NA	0,48287384	NA	8	-18998,445	38012,8979	176,481154	1,56E-25
496	-0,45110821	0,66723944	0,00674107	4,29E-05	-0,00076432	NA	-0,15224001	-2,87228819	0,44207022	0,01904584	0,00021405	NA	NA	NA	11	-18995,5235	38013,0617	176,644942	1,44E-25
3925	-1,36352414	NA	NA	4,29E-05	NA	0,54467715	NA	-1,71083893	0,37612338	NA	0,00021703	0,00013898	0,46389079	-0,13469622	10	-18996,6238	38013,2598	176,843061	1,30E-25
3415	-1,08960867	NA	-0,00576757	4,36E-05	NA	0,53973144	NA	-1,61349146	0,35721774	NA	0,00021669	NA	0,46315983	-0,13737061	10	-18996,6648	38013,3417	176,924984	1,25E-26
1006	-0,14195779	0,65649184	NA	4,36E-05	-0,00074998	NA	-0,15445798	-2,9468992	0,4586198	0,01897353	0,00021429	0,00012376	NA	NA	11	-18995,7063	38013,4272	177,010505	1,20E-25
1798	-1,235734	0,76857786	NA	4,69E-05	NA	NA	NA	-1,78635429	NA	NA	0,0002119	-0,00029974	0,47515083	NA	8	-18998,7387	38013,4854	177,068654	1,16E-25
1320	-1,05409025	0,78157333	0,00543311	4,44E-05	NA	NA	-0,11273577	-1,96172815	NA	NA	0,00021146	NA	0,48287384	NA	9	-18998,0847	38014,1793	177,762568	8,22E-26
2510	-0,8455927	0,67951403	NA	4,64E-05	-0,00057321	NA	NA	-2,55577604	0,44288659	0,01897716	0,00021141	NA	NA	-0,08883356	10	-18997,1479	38014,308	177,891261	7,71E-26
1008	-0,45110821	0,66723944	0,00674107	4,29E-05	-0,00076432	NA	-0,15224001	-2,9468992	0,4586198	0,01897353	0,00021429	0,00012376	NA	NA	12	-18995,1482	38014,3136	177,89684	7,69E-26
3927	-1,08960867	NA	-0,00576757	4,36E-05	NA	0,53973144	NA	-1,71083893	0,37612338	NA	0,00021703	0,00013898	0,46389079	-0,13469622	11	-18996,1915	38014,3976	177,980917	7,37E-27
1800	-1,52051301	0,78035551	0,00624724	4,63E-05	NA	NA	NA	-1,78635429	NA	NA	0,0002119	-0,00029974	0,47515083	NA	9	-18998,2626	38014,5352	178,118462	6,88E-26
2512	-1,17938783	0,69098467	0,00751019	4,56E-05	-0,00059207	NA	NA	-2,55577604	0,44288659	0,01897716	0,00021141	NA	NA	-0,08883356	11	-18996,458	38014,9305	178,513796	5,65E-26
2534	-0,79586264	0,77122371	NA	4,50E-05	NA	NA	-0,11503973	-2,55577604	0,44288659	0,01897716	0,00021141	NA	NA	-0,08883356	10	-18997,7216	38015,4554	179,03869	4,34E-26
3022	-0,8455927	0,67951403	NA	4,64E-05	-0,00057321	NA	NA	-2,61719209	0,45517288	0,01892094	0,00021164	9,21E-05	NA	-0,08713002	11	-18996,9424	38015,8993	179,482626	3,48E-26

3024	-1,17938783	0,69098467	0,00751019	4,56E-05	-0,00059207	NA	NA	-2,61719209	0,45517288	0,01892094	0,00021164	9,21E-05	NA	-0,08713002	12	-18996,2524	38016,5221	180,105382	2,55E-26
1286	-1,235734	0,76857786	NA	4,69E-05	NA	NA	NA	-1,96172815	NA	NA	0,00021146	NA	0,48287384	NA	7	-19001,3436	38016,6934	180,27667	2,34E-26
2536	-1,05409025	0,78157333	0,00543311	4,44E-05	NA	NA	-0,11273577	-2,55577604	0,44288659	0,01897716	0,00021141	NA	NA	-0,08883356	11	-18997,3613	38016,7373	180,320547	2,29E-26
1949	-0,31227401	NA	NA	4,32E-05	-0,00145314	0,47935513	NA	-2,58652225	NA	0,01772679	0,00020696	-0,00034396	0,48679163	NA	10	-18998,408	38016,8282	180,411509	2,19E-26
3046	-0,79586264	0,77122371	NA	4,50E-05	NA	NA	-0,11503973	-2,61719209	0,45517288	0,01892094	0,00021164	9,21E-05	NA	-0,08713002	11	-18997,5161	38017,0468	180,630054	1,96E-26
1288	-1,52051301	0,78035551	0,00624724	4,63E-05	NA	NA	NA	-1,96172815	NA	NA	0,00021146	NA	0,48287384	NA	8	-19000,8675	38017,743	181,326257	1,38E-26
3048	-1,05409025	0,78157333	0,00543311	4,44E-05	NA	NA	-0,11273577	-2,61719209	0,45517288	0,01892094	0,00021164	9,21E-05	NA	-0,08713002	12	-18997,1558	38018,3289	181,912133	1,03E-26
1951	-0,39160679	NA	0,00183718	4,30E-05	-0,00146197	0,48040455	NA	-2,58652225	NA	0,01772679	0,00020696	-0,00034396	0,48679163	NA	11	-18998,3646	38018,7438	182,327121	8,39E-27
2502	-1,235734	0,76857786	NA	4,69E-05	NA	NA	NA	-2,55577604	0,44288659	0,01897716	0,00021141	NA	NA	-0,08883356	9	-19000,6203	38019,2505	182,833763	6,51E-27
3869	-0,31227401	NA	NA	4,32E-05	-0,00145314	0,47935513	NA	-1,25486087	NA	NA	0,00020772	-0,0003441	0,50313521	-0,14558566	10	-18999,765	38019,5422	183,125524	5,63E-27
462	-0,8455927	0,67951403	NA	4,64E-05	-0,00057321	NA	NA	-2,87228819	0,44207022	0,01904584	0,00021405	NA	NA	NA	9	-19001,1022	38020,2143	183,797562	4,02E-27
2504	-1,52051301	0,78035551	0,00624724	4,63E-05	NA	NA	NA	-2,55577604	0,44288659	0,01897716	0,00021141	NA	NA	-0,08883356	10	-19000,1442	38020,3005	183,883793	3,85E-27
464	-1,17938783	0,69098467	0,00751019	4,56E-05	-0,00059207	NA	NA	-2,87228819	0,44207022	0,01904584	0,00021405	NA	NA	NA	10	-19000,4122	38020,8366	184,419875	2,95E-27
3014	-1,235734	0,76857786	NA	4,69E-05	NA	NA	NA	-2,61719209	0,45517288	0,01892094	0,00021164	9,21E-05	NA	-0,08713002	10	-19000,4147	38020,8416	184,424905	2,94E-27
1853	0,65451489	NA	NA	3,95E-05	-0,00164357	0,48762971	-0,22170047	-1,78635429	NA	NA	0,0002119	-0,00029974	0,47515083	NA	10	-19000,544	38021,1001	184,683387	2,58E-27
3405	-0,28011525	NA	NA	4,24E-05	-0,0016491	NA	NA	-1,61349146	0,35721774	NA	0,00021669	NA	0,46315983	-0,13737061	9	-19001,5888	38021,1875	184,770769	2,47E-27
486	-0,79586264	0,77122371	NA	4,50E-05	NA	NA	-0,11503973	-2,87228819	0,44207022	0,01904584	0,00021405	NA	NA	NA	9	-19001,6759	38021,3617	184,94499	2,27E-27
3871	-0,39160679	NA	0,00183718	4,30E-05	-0,00146197	0,48040455	NA	-1,25486087	NA	NA	0,00020772	-0,0003441	0,50313521	-0,14558566	11	-18999,7216	38021,4579	185,041136	2,16E-27
974	-0,8455927	0,67951403	NA	4,64E-05	-0,00057321	NA	NA	-2,9468992	0,4586198	0,01897353	0,00021429	0,00012376	NA	NA	10	-19000,7268	38021,4657	185,049016	2,15E-27
1437	-0,31227401	NA	NA	4,32E-05	-0,00145314	0,47935513	NA	-2,75826159	NA	0,01709531	0,00020664	NA	0,4949792	NA	9	-19001,7965	38021,6029	185,18621	2,01E-28
3016	-1,52051301	0,78035551	0,00624724	4,63E-05	NA	NA	NA	-2,61719209	0,45517288	0,01892094	0,00021164	9,21E-05	NA	-0,08713002	11	-18999,9386	38021,8919	185,475157	1,74E-27
976	-1,17938783	0,69098467	0,00751019	4,56E-05	-0,00059207	NA	NA	-2,9468992	0,4586198	0,01897353	0,00021429	0,00012376	NA	NA	11	-19000,0368	38022,0883	185,671551	1,58E-27
3917	-0,28011525	NA	NA	4,24E-05	-0,0016491	NA	NA	-1,71083893	0,37612338	NA	0,00021703	0,00013898	0,46389079	-0,13469622	10	-19001,1155	38022,2432	185,82648	1,46E-27
998	-0,79586264	0,77122371	NA	4,50E-05	NA	NA	-0,11503973	-2,9468992	0,4586198	0,01897353	0,00021429	0,00012376	NA	NA	10	-19001,3005	38022,6132	186,196445	1,21E-27
488	-1,05409025	0,78157333	0,00543311	4,44E-05	NA	NA	-0,11273577	-2,87228819	0,44207022	0,01904584	0,00021405	NA	NA	NA	10	-19001,3156	38022,6433	186,226626	1,19E-27
1855	0,61699723	NA	0,00084798	3,94E-05	-0,00164748	0,4880593	-0,22142621	-1,78635429	NA	NA	0,0002119	-0,00029974	0,47515083	NA	11	-19000,5347	38023,084	186,667232	9,58E-28
3407	-0,2131598	NA	-0,0015413	4,25E-05	-0,00164139	NA	NA	-1,61349146	0,35721774	NA	0,00021669	NA	0,46315983	-0,13737061	10	-19001,5591	38023,1303	186,713594	9,36E-28
1389	0,64576877	NA	NA	3,88E-05	-0,00183092	NA	-0,20438761	-2,09152745	0,35956015	NA	0,00022076	NA	0,43622845	NA	9	-19002,6607	38023,3314	186,914728	8,46E-28
1439	-0,39160679	NA	0,00183718	4,30E-05	-0,00146197	0,48040455	NA	-2,75826159	NA	0,01709531	0,00020664	NA	0,4949792	NA	10	-19001,7531	38023,5183	187,101601	7,71E-28
1901	0,64576877	NA	NA	3,88E-05	-0,00183092	NA	-0,20438761	-2,2103983	0,38500536	NA	0,00022113	0,00018745	0,43806177	NA	10	-19001,7867	38023,5855	187,168819	7,45E-29
1397	-0,84339357	NA	NA	4,07E-05	NA	0,55505772	-0,13640315	-2,09152745	0,35956015	NA	0,00022076	NA	0,43622845	NA	9	-19002,919	38023,8479	187,43116	6,54E-28
1000	-1,05409025	0,78157333	0,00543311	4,44E-05	NA	NA	-0,11273577	-2,9468992	0,4586198	0,01897353	0,00021429	0,00012376	NA	NA	11	-19000,9402	38023,895	187,478302	6,39E-28
1909	-0,84339357	NA	NA	4,07E-05	NA	0,55505772	-0,13640315	-2,2103983	0,38500536	NA	0,00022113	0,00018745	0,43806177	NA	10	-19002,0449	38024,102	187,685251	5,76E-28
3919	-0,2131598	NA	-0,0015413	4,25E-05	-0,00164139	NA	NA	-1,71083893	0,37612338	NA	0,00021703	0,00013898	0,46389079	-0,13469622	11	-19001,0858	38024,1862	187,769526	5,52E-28
1341	0,65451489	NA	NA	3,95E-05	-0,00164357	0,48762971	-0,22170047	-1,96172815	NA	NA	0,00021146	NA	0,48287384	NA	9	-19003,1489	38024,3077	187,890961	5,20E-28
3357	-0,31227401	NA	NA	4,32E-05	-0,00145314	0,47935513	NA	-1,4783871	NA	NA	0,00020739	NA	0,51055393	-0,13912561	9	-19003,159	38024,3279	187,91116	5,14E-28
1399	-0,50593593	NA	-0,00700816	4,14E-05	NA	0,55021643	-0,14061875	-2,09152745	0,35956015	NA	0,00022076	NA	0,43622845	NA	10	-19002,2859	38024,584	188,167251	4,52E-28
1911	-0,50593593	NA	-0,00700816	4,14E-05	NA	0,55021643	-0,14061875	-2,2103983	0,38500536	NA	0,00022113	0,00018745	0,43806177	NA	11	-19001,4118	38024,8383	188,421564	3,98E-28
454	-1,235734	0,76857786	NA	4,69E-05	NA	NA	NA	-2,87228819	0,44207022	0,01904584	0,00021405	NA	NA	NA	8	-19004,5745	38025,157	188,740285	3,40E-28
1391	0,74969745	NA	-0,00245756	3,91E-05	-0,00181949	NA	-0,20513804	-2,09152745	0,35956015	NA	0,00022076	NA	0,43622845	NA	10	-19002,5852	38025,1825	188,76579	3,35E-28
1903	0,74969745	NA	-0,00245756	3,91E-05	-0,00181949	NA	-0,20513804	-2,2103983	0,38500536	NA	0,00022113	0,00018745	0,43806177	NA	11	-19001,7111	38025,4368	189,020103	2,95E-28
456	-1,52051301	0,78035551	0,00624724	4,63E-05	NA	NA	NA	-2,87228819	0,44207022	0,01904584	0,00021405	NA	NA	NA	9	-19004,0984	38026,2068	189,790094	2,01E-28
3359	-0,39160679	NA	0,00183718	4,30E-05	-0,00146197	0,48040455	NA	-1,4783871	NA	NA	0,00020739	NA	0,51055393	-0,13912561	10	-19003,1156	38026,2433	189,826551	1,97E-28
1343	0,61699723	NA	0,00084798	3,94E-05	-0,00164748	0,4880593	-0,22142621	-1,96172815	NA	NA	0,00021146	NA	0,48287384	NA	10	-19003,1396	38026,2913	189,874584	1,93E-28
966	-1,235734	0,76857786	NA	4,69E-05	NA	NA	NA	-2,9468992	0,4586198	0,01897353	0,00021429	0,00012376	NA	NA	9	-19004,1991	38026,4082	189,991518	1,82E-28
2557	0,65451489	NA	NA	3,95E-05	-0,00164357	0,48762971	-0,22170047	-2,55577604	0,44288659	0,01897716	0,00021141	NA	NA	-0,08883356	11	-19002,4255	38026,8657	190,448939	1,45E-28
968	-1,52051301	0,78035551	0,00624724	4,63E-05	NA	NA	NA	-2,9468992	0,4586198	0,01897353	0,00021429	0,00012376	NA	NA	10	-19003,7231	38027,4583	191,041548	1,08E-28
3069	0,65451489	NA	NA	3,95E-05	-0,00164357	0,48762971	-0,22170047	-2,61719209	0,45517288	0,01892094	0,00021164	9,21E-05	NA	-0,08713002	12	-19002,22	38028,4572	192,040525	6,52E-29
2559	0,61699723	NA	0,00084798	3,94E-05	-0,00164748	0,4880593	-0,22142621	-2,55577604	0,44288659	0,01897716	0,00021141	NA	NA	-0,08883356	12	-19002,4162	38028,8497	192,433006	5,36E-29
1365	-1,36352414	NA	NA	4,29E-05	NA	0,54467715	NA	-2,09152745	0,35956015	NA	0,00022076	NA	0,43622845	NA	8	-19006,4593	38028,9267	192,509929	5,16E-29
1877	-1,36352414	NA	NA	4,29E-05	NA	0,54467715	NA	-2,2103983	0,38500536	NA	0,00022113	0,00018745	0,43806177	NA	9	-19005,5853	38029,1805	192,763798	4,54E-29
1367	-1,08960867	NA	-0,00576757	4,36E-05	NA	0,53973144	NA	-2,09152745	0,35956015	NA	0,00022076	NA	0,43622845	NA	9	-19006,0271	38030,0641	193,647342	2,92E-29
1879	-1,08960867	NA	-0,00576757	4,36E-05	NA	0,53973144	NA	-2,2103983	0,38500536	NA	0,00022113	0,00018745	0,43806177	NA	10	-19005,153	38030,3182	193,901433	2,57E-29
3071	0,61699723	NA	0,00084798	3,94E-05	-0,00164748	0,4880593	-0,22142621	-2,61719209	0,45517288	0,01892094	0,00021164	9,21E-05	NA	-0,08713002	13	-19002,2107	38030,4415	194,024814	2,42E-29
2414	-0,14195779	0,65649184	NA	4,36E-05	-0,00074998	NA	-0,15445798	-1,66433243	0,4162362	NA	0,00021617	NA	NA	-0,08904476	10	-19006,009	38032,0301	195,613354	1,09E-29
509	0,65451489	NA	NA	3,95E-05	-0,00164357	0,48762971	-0,22170047	-2,87228819	0,44207022	0,01904584	0,00021405	NA	NA	NA	10	-19006,3798	38032,7717	196,355018	7,55E-30
2416	-0,45110821	0,66723944	0,00674107	4,29E-05	-0,00076432	NA	-0,15224001	-1,66433243	0,4162362	NA	0,00021617	NA	NA	-0,08904476	11	-19005,4508	38032,9162	196,499467	7,02E-30
2926	-0,14195779	0,65649184	NA	4,36E-05	-0,00074998	NA	-0,15445798	-1,73983563	0,43090563	NA	0,00021643	0,00010896	NA	-0,08710954	11	-19005,7192	38033,4531	197,036331	5,37E-30
1021	0,65451489	NA	NA	3,95E-05	-0,00164357	0,48762971	-0,22170047	-2,9468992	0,4586198	0,01897353	0,00021429	0,00012376	NA	NA	11	-19006,0044	38034,0234	197,606695	4,04E-30
2928	-0,45110821	0,66723944	0,00674107	4,29E-05	-0,00076432	NA	-0,15224001	-1,73983563	0,43090563	NA	0,00021643	0,00010896	NA	-0,08710954	12	-19005,1611	38034,3394	197,922665	3,45E-30
511	0,61699723	NA	0,00084798	3,94E-05	-0,00164748	0,4880593	-0,22142621	-2,87228819	0,44207022	0,01904584	0,00021405	NA	NA	NA	11	-19006,3705	38034,7556	198,338864	2,80E-30

1023	0,61699723	NA	0,00084798	3,94E-05	-0,00164748	0,4880593	-0,22142621	-2,9468992	0,4586198	0,01897353	0,00021429	0,00012376	NA	NA	12	-19005,9951	38036,0075	199,590761	1,50E-30
1357	-0,28011525	NA	NA	4,24E-05	-0,0016491	NA	NA	-2,09152745	0,35956015	NA	0,00022076	NA	0,43622845	NA	8	-19010,9511	38037,9101	201,493348	5,78E-32
366	-0,14195779	0,65649184	NA	4,36E-05	-0,00074998	NA	-0,15445798	-1,97841134	0,41538406	NA	0,00021885	NA	NA	NA	9	-19010,0001	38038,0101	201,593406	5,50E-31
1869	-0,28011525	NA	NA	4,24E-05	-0,0016491	NA	NA	-2,2103983	0,38500536	NA	0,00022113	0,00018745	0,43806177	NA	9	-19010,077	38038,1639	201,747217	5,09E-32
368	-0,45110821	0,66723944	0,00674107	4,29E-05	-0,00076432	NA	-0,15224001	-1,97841134	0,41538406	NA	0,00021885	NA	NA	NA	10	-19009,4419	38038,896	202,479298	3,53E-31
1821	-0,31227401	NA	NA	4,32E-05	-0,00145314	0,47935513	NA	-1,78635429	NA	NA	0,0002119	-0,00029974	0,47515083	NA	9	-19010,4468	38038,9036	202,486868	3,52E-31
878	-0,14195779	0,65649184	NA	4,36E-05	-0,00074998	NA	-0,15445798	-2,0661368	0,43415925	NA	0,00021911	0,00013912	NA	NA	10	-19009,5222	38039,0565	202,639755	3,26E-31
1359	-0,2131598	NA	-0,0015413	4,25E-05	-0,00164139	NA	NA	-2,09152745	0,35956015	NA	0,00022076	NA	0,43622845	NA	9	-19010,9214	38039,8527	203,435951	2,19E-31
880	-0,45110821	0,66723944	0,00674107	4,29E-05	-0,00076432	NA	-0,15224001	-2,0661368	0,43415925	NA	0,00021911	0,00013912	NA	NA	11	-19008,964	38039,9426	203,525869	2,09E-31
2382	-0,8455927	0,67951403	NA	4,64E-05	-0,00057321	NA	NA	-1,66433243	0,4162362	NA	0,00021617	NA	NA	-0,08904476	9	-19011,0294	38040,0688	203,652087	1,96E-31
1871	-0,2131598	NA	-0,0015413	4,25E-05	-0,00164139	NA	NA	-2,2103983	0,38500536	NA	0,00022113	0,00018745	0,43806177	NA	10	-19010,0473	38040,1068	203,690042	1,93E-31
2384	-1,17938783	0,69098467	0,00751019	4,56E-05	-0,00059207	NA	NA	-1,66433243	0,4162362	NA	0,00021617	NA	NA	-0,08904476	10	-19010,3395	38040,6911	204,274401	1,44E-31
1823	-0,39160679	NA	0,00183718	4,30E-05	-0,00146197	0,48040455	NA	-1,78635429	NA	NA	0,0002119	-0,00029974	0,47515083	NA	10	-19010,4034	38040,819	204,402258	1,35E-31
2406	-0,79586264	0,77122371	NA	4,50E-05	NA	NA	-0,11503973	-1,66433243	0,4162362	NA	0,00021617	NA	NA	-0,08904476	9	-19011,6031	38041,2162	204,799516	1,11E-31
2894	-0,8455927	0,67951403	NA	4,64E-05	-0,00057321	NA	NA	-1,73983563	0,43090563	NA	0,00021643	0,00010896	NA	-0,08710954	10	-19010,7397	38041,4916	205,074842	9,64E-32
1309	-0,31227401	NA	NA	4,32E-05	-0,00145314	0,47935513	NA	-1,96172815	NA	NA	0,00021146	NA	0,48287384	NA	8	-19013,0517	38042,1114	205,694663	7,07E-32
2896	-1,17938783	0,69098467	0,00751019	4,56E-05	-0,00059207	NA	NA	-1,73983563	0,43090563	NA	0,00021643	0,00010896	NA	-0,08710954	11	-19010,0497	38042,1141	205,697377	7,06E-33
2408	-1,05409025	0,78157333	0,00543311	4,44E-05	NA	NA	-0,11273577	-1,66433243	0,4162362	NA	0,00021617	NA	NA	-0,08904476	10	-19011,2429	38042,4979	206,081151	5,83E-32
2918	-0,79586264	0,77122371	NA	4,50E-05	NA	NA	-0,11503973	-1,73983563	0,43090563	NA	0,00021643	0,00010896	NA	-0,08710954	10	-19011,3134	38042,639	206,22227	5,43E-32
2920	-1,05409025	0,78157333	0,00543311	4,44E-05	NA	NA	-0,11273577	-1,73983563	0,43090563	NA	0,00021643	0,00010896	NA	-0,08710954	11	-19010,9531	38043,9208	207,504127	2,86E-32
1311	-0,39160679	NA	0,00183718	4,30E-05	-0,00146197	0,48040455	NA	-1,96172815	NA	NA	0,00021146	NA	0,48287384	NA	9	-19013,0257	38044,0266	207,609832	2,71E-32
2525	-0,31227401	NA	NA	4,32E-05	-0,00145314	0,47935513	NA	-2,55577604	0,44288659	0,01897716	0,00021141	NA	NA	-0,08883356	10	-19012,3284	38044,6689	208,252198	1,97E-32
2374	-1,235734	0,76857786	NA	4,69E-05	NA	NA	NA	-1,66433243	0,4162362	NA	0,00021617	NA	NA	-0,08904476	8	-19014,5018	38045,0115	208,59481	1,66E-32
334	-0,8455927	0,67951403	NA	4,64E-05	-0,00057321	NA	NA	-1,97841134	0,41538406	NA	0,00021885	NA	NA	NA	8	-19015,0206	38046,0491	209,63236	9,87E-33
2376	-1,52051301	0,78035551	0,00624724	4,63E-05	NA	NA	NA	-1,66433243	0,4162362	NA	0,00021617	NA	NA	-0,08904476	9	-19014,0257	38046,0613	209,644619	9,81E-33
3037	-0,31227401	NA	NA	4,32E-05	-0,00145314	0,47935513	NA	-2,61719209	0,45517288	0,01892094	0,00021164	9,21E-05	NA	-0,08713002	11	-19012,1228	38046,2603	209,843563	8,89E-33
2886	-1,235734	0,76857786	NA	4,69E-05	NA	NA	NA	-1,73983563	0,43090563	NA	0,00021643	0,00010896	NA	-0,08710954	9	-19014,2121	38046,4341	210,017343	8,15E-33
2527	-0,39160679	NA	0,00183718	4,30E-05	-0,00146197	0,48040455	NA	-2,55577604	0,44288659	0,01897716	0,00021141	NA	NA	-0,08883356	11	-19012,285	38046,5845	210,16781	7,56E-33
336	-1,17938783	0,69098467	0,00751019	4,56E-05	-0,00059207	NA	NA	-1,97841134	0,41538406	NA	0,00021885	NA	NA	NA	9	-19014,3306	38046,6712	210,254452	7,24E-33
846	-0,8455927	0,67951403	NA	4,64E-05	-0,00057321	NA	NA	-2,0661368	0,43415925	NA	0,00021911	0,00013912	NA	NA	9	-19014,5426	38047,0952	210,678488	5,85E-33
358	-0,79586264	0,77122371	NA	4,50E-05	NA	NA	-0,11503973	-1,97841134	0,41538406	NA	0,00021885	NA	NA	NA	8	-19015,5943	38047,1965	210,779789	5,56E-33
2888	-1,52051301	0,78035551	0,00624724	4,63E-05	NA	NA	NA	-1,73983563	0,43090563	NA	0,00021643	0,00010896	NA	-0,08710954	10	-19013,736	38047,4841	211,067373	4,82E-33
848	-1,17938783	0,69098467	0,00751019	4,56E-05	-0,00059207	NA	NA	-2,0661368	0,43415925	NA	0,00021911	0,00013912	NA	NA	10	-19013,8527	38047,7175	211,300802	4,29E-33
3039	-0,39160679	NA	0,00183718	4,30E-05	-0,00146197	0,48040455	NA	-2,61719209	0,45517288	0,01892094	0,00021164	9,21E-05	NA	-0,08713002	12	-19012,0794	38048,1761	211,759397	3,41E-33
870	-0,79586264	0,77122371	NA	4,50E-05	NA	NA	-0,11503973	-2,0661368	0,43415925	NA	0,00021911	0,00013912	NA	NA	9	-19015,1163	38048,2426	211,825917	3,30E-33
4013	0,64576877	NA	NA	3,88E-05	-0,00183092	NA	-0,20438761	-2,05206432	NA	0,01786019	0,00020271	-0,00039168	0,51529109	-0,14768431	11	-19013,1642	38048,3431	211,926351	3,14E-33
360	-1,05409025	0,78157333	0,00543311	4,44E-05	NA	NA	-0,11273577	-1,97841134	0,41538406	NA	0,00021885	NA	NA	NA	9	-19015,234	38048,4779	212,061203	2,93E-33
4021	-0,84339357	NA	NA	4,07E-05	NA	0,55505772	-0,13640315	-2,05206432	NA	0,01786019	0,00020271	-0,00039168	0,51529109	-0,14768431	11	-19013,4225	38048,8595	212,442783	2,42E-34
872	-1,05409025	0,78157333	0,00543311	4,44E-05	NA	NA	-0,11273577	-2,0661368	0,43415925	NA	0,00021911	0,00013912	NA	NA	10	-19014,7561	38049,5243	213,107552	1,74E-33
4023	-0,50593593	NA	-0,00700816	4,14E-05	NA	0,55021643	-0,14061875	-2,05206432	NA	0,01786019	0,00020271	-0,00039168	0,51529109	-0,14768431	12	-19012,7894	38049,596	213,179317	1,68E-34
4015	0,74969745	NA	-0,00245756	3,91E-05	-0,00181949	NA	-0,20513804	-2,05206432	NA	0,01786019	0,00020271	-0,00039168	0,51529109	-0,14768431	12	-19013,0887	38050,1946	213,777856	1,24E-33
477	-0,31227401	NA	NA	4,32E-05	-0,00145314	0,47935513	NA	-2,87228819	0,44207022	0,01904584	0,00021405	NA	NA	NA	9	-19016,2826	38050,5752	214,158499	1,03E-33
326	-1,235734	0,76857786	NA	4,69E-05	NA	NA	NA	-1,97841134	0,41538406	NA	0,00021885	NA	NA	NA	7	-19018,4929	38050,992	214,575305	8,34E-34
989	-0,31227401	NA	NA	4,32E-05	-0,00145314	0,47935513	NA	-2,9468992	0,4586198	0,01897353	0,00021429	0,00012376	NA	NA	10	-19015,9073	38051,8267	215,409954	5,49E-34
838	-1,235734	0,76857786	NA	4,69E-05	NA	NA	NA	-2,0661368	0,43415925	NA	0,00021911	0,00013912	NA	NA	8	-19018,015	38052,0379	215,621211	4,94E-34
328	-1,52051301	0,78035551	0,00624724	4,63E-05	NA	NA	NA	-1,97841134	0,41538406	NA	0,00021885	NA	NA	NA	8	-19018,0168	38052,0416	215,624892	4,93E-35
479	-0,39160679	NA	0,00183718	4,30E-05	-0,00146197	0,48040455	NA	-2,87228819	0,44207022	0,01904584	0,00021405	NA	NA	NA	10	-19016,2392	38052,4906	216,07389	3,94E-36
2429	0,65451489	NA	NA	3,95E-05	-0,00164357	0,48762971	-0,22170047	-1,66433243	0,4162362	NA	0,00021617	NA	NA	-0,08904476	10	-19016,307	38052,6263	216,209544	3,68E-34
840	-1,52051301	0,78035551	0,00624724	4,63E-05	NA	NA	NA	-2,0661368	0,43415925	NA	0,00021911	0,00013912	NA	NA	9	-19017,5389	38053,0877	216,67102	2,92E-34
3006	-0,06671637	0,57575795	NA	4,34E-05	-0,00071955	0,41051829	-0,17478186	-1,91049389	NA	0,01549989	0,00020133	-0,00049532	NA	-0,09486783	12	-19014,7524	38053,522	217,105241	2,35E-34
3008	-0,47166397	0,58945108	0,00876766	4,25E-05	-0,00073797	0,4135805	-0,1717464	-1,91049389	NA	0,01549989	0,00020133	-0,00049532	NA	-0,09486783	13	-19013,7782	38053,5765	217,159741	2,29E-34
991	-0,39160679	NA	0,00183718	4,30E-05	-0,00146197	0,48040455	NA	-2,9468992	0,4586198	0,01897353	0,00021429	0,00012376	NA	NA	11	-19015,8638	38053,7423	217,325566	2,11E-35
3989	-1,36352414	NA	NA	4,29E-05	NA	0,54467715	NA	-2,05206432	NA	0,01786019	0,00020271	-0,00039168	0,51529109	-0,14768431	10	-19016,9628	38053,9378	217,521108	1,91E-34
2941	0,65451489	NA	NA	3,95E-05	-0,00164357	0,48762971	-0,22170047	-1,73983563	0,43090563	NA	0,00021643	0,00010896	NA	-0,08710954	11	-19016,0173	38054,0492	217,63252	1,81E-34
2431	0,61699723	NA	0,00084798	3,94E-05	-0,00164748	0,4880593	-0,22142621	-1,66433243	0,4162362	NA	0,00021617	NA	NA	-0,08904476	11	-19016,2978	38054,6101	218,193389	1,37E-34
3501	0,64576877	NA	NA	3,88E-05	-0,00183092	NA	-0,20438761	-2,27275384	NA	0,01711404	0,00020255	NA	0,52288611	-0,14000571	10	-19017,505	38055,0221	218,605354	1,11E-35
3991	-1,08960867	NA	-0,00576757	4,36E-05	NA	0,53973144	NA	-2,05206432	NA	0,01786019	0,00020271	-0,00039168	0,51529109	-0,14768431	11	-19016,5305	38055,0757	218,658965	1,08E-34
3509	-0,84339357	NA	NA	4,07E-05	NA	0,55505772	-0,13640315	-2,27275384	NA	0,01711404	0,00020255	NA	0,52288611	-0,14000571	10	-19017,7632	38055,5385	219,121786	8,59E-36
2943	0,61699723	NA	0,00084798	3,94E-05	-0,00164748	0,4880593	-0,22142621	-1,73983563	0,43090563	NA	0,00021643	0,00010896	NA	-0,08710954	12	-19016,008	38056,0333	219,616587	6,71E-35
3511	-0,50593593	NA	-0,00700816	4,14E-05	NA	0,55021643	-0,14061875	-2,27275384	NA	0,01711404	0,00020255	NA	0,52288611	-0,14000571	11	-19017,1301	38056,2748	219,858099	5,94E-35
3503	0,74969745	NA	-0,00245756	3,91E-05	-0,00181949	NA	-0,20513804	-2,27275384	NA	0,01711404	0,00020255	NA	0,52288611	-0,14000571	11	-19017,4294	38056,8734	220,456638	4,41E-35

381	0,65451489	NA	NA	3,95E-05	-0,00164357	0,48762971	-0,22170047	-1,97841134	0,41538406	NA	0,00021885	NA	NA	NA	9	-19020,2982	38058,6063	222,189596	1,85E-35
893	0,65451489	NA	NA	3,95E-05	-0,00164357	0,48762971	-0,22170047	-2,0661368	0,43415925	NA	0,00021911	0,00013912	NA	NA	10	-19019,8202	38059,6527	223,235945	1,10E-35
383	0,61699723	NA	0,00084798	3,94E-05	-0,00164748	0,4880593	-0,22142621	-1,97841134	0,41538406	NA	0,00021885	NA	NA	NA	10	-19020,2889	38060,5899	224,173219	6,87E-36
3477	-1,36352414	NA	NA	4,29E-05	NA	0,54467715	NA	-2,27275384	NA	0,01711404	0,00020255	NA	0,52288611	-0,14000571	9	-19021,3036	38060,6171	224,200333	6,78E-36
958	-0,06671637	0,57575795	NA	4,34E-05	-0,00071955	0,41051829	-0,17478186	-2,26386687	NA	0,01548996	0,00020413	-0,00046266	NA	NA	11	-19019,3499	38060,7144	224,297727	6,46E-37
960	-0,47166397	0,58945108	0,00876766	4,25E-05	-0,00073797	0,4135805	-0,1717464	-2,26386687	NA	0,01548996	0,00020413	-0,00046266	NA	NA	12	-19018,3757	38060,7687	224,352006	6,28E-36
895	0,61699723	NA	0,00084798	3,94E-05	-0,00164748	0,4880593	-0,22142621	-2,0661368	0,43415925	NA	0,00021911	0,00013912	NA	NA	11	-19019,811	38061,6365	225,21979	4,07E-36
3479	-1,08960867	NA	-0,00576757	4,36E-05	NA	0,53973144	NA	-2,27275384	NA	0,01711404	0,00020255	NA	0,52288611	-0,14000571	10	-19020,8713	38061,7547	225,337968	3,84E-36
2998	-0,67953035	0,68234123	NA	4,46E-05	NA	0,41541375	-0,13795142	-1,91049389	NA	0,01549989	0,00020133	-0,00049532	NA	-0,09486783	11	-19019,939	38061,8925	225,475811	3,58E-36
3559	-0,65771633	NA	-0,01187559	4,14E-05	NA	NA	-0,10732692	-2,59012234	0,38593256	0,02081194	0,00021148	NA	0,47514204	-0,138389	11	-19020,1711	38062,3567	225,939996	2,84E-36
3000	-1,04079715	0,69637088	0,00755047	4,39E-05	NA	0,41816409	-0,13461471	-1,91049389	NA	0,01549989	0,00020133	-0,00049532	NA	-0,09486783	12	-19019,2197	38062,4567	226,039975	2,70E-38
3981	-0,28011525	NA	NA	4,24E-05	-0,0016491	NA	NA	-2,05206432	NA	0,01786019	0,00020271	-0,00039168	0,51529109	-0,14768431	10	-19021,4545	38062,9212	226,504528	2,14E-36
4071	-0,65771633	NA	-0,01187559	4,14E-05	NA	NA	-0,10732692	-2,66976045	0,40191971	0,02073197	0,0002118	0,00011864	0,47565984	-0,13600353	12	-19019,8291	38063,6754	227,25865	1,47E-37
3557	-1,22822825	NA	NA	4,02E-05	NA	NA	-0,09995451	-2,59012234	0,38593256	0,02081194	0,00021148	NA	0,47514204	-0,138389	10	-19021,9155	38063,8432	227,426456	1,35E-36
2976	-1,28042467	0,61751101	0,00965662	4,55E-05	-0,00054807	0,40424516	NA	-1,91049389	NA	0,01549989	0,00020133	-0,00049532	NA	-0,09486783	12	-19020,0244	38064,066	227,649306	1,21E-36
2974	-0,85074914	0,60334436	NA	4,65E-05	-0,00052401	0,40049867	NA	-1,91049389	NA	0,01549989	0,00020133	-0,00049532	NA	-0,09486783	11	-19021,2002	38064,415	227,99832	1,01E-36
3527	-1,14142692	NA	-0,01089562	4,31E-05	NA	NA	NA	-2,59012234	0,38593256	0,02081194	0,00021148	NA	0,47514204	-0,138389	10	-19022,2852	38064,5825	228,165764	9,33E-37
3983	-0,2131598	NA	-0,0015413	4,25E-05	-0,00164139	NA	NA	-2,05206432	NA	0,01786019	0,00020271	-0,00039168	0,51529109	-0,14768431	11	-19021,4248	38064,8643	228,447574	8,11E-37
4069	-1,22822825	NA	NA	4,02E-05	NA	NA	-0,09995451	-2,66976045	0,40191971	0,02073197	0,0002118	0,00011864	0,47565984	-0,13600353	11	-19021,5735	38065,1616	228,744888	6,99E-38
2494	-0,06671637	0,57575795	NA	4,34E-05	-0,00071955	0,41051829	-0,17478186	-2,18775169	NA	0,01458917	0,00020109	NA	NA	-0,08533198	11	-19021,6133	38065,2412	228,824483	6,71E-38
2496	-0,47166397	0,58945108	0,00876766	4,25E-05	-0,00073797	0,4135805	-0,1717464	-2,18775169	NA	0,01458917	0,00020109	NA	NA	-0,08533198	12	-19020,6391	38065,2955	228,878761	6,53E-37
3525	-1,63084607	NA	NA	4,18E-05	NA	NA	NA	-2,59012234	0,38593256	0,02081194	0,00021148	NA	0,47514204	-0,138389	9	-19023,7603	38065,5306	229,113912	5,81E-37
4039	-1,14142692	NA	-0,01089562	4,31E-05	NA	NA	NA	-2,66976045	0,40191971	0,02073197	0,0002118	0,00011864	0,47565984	-0,13600353	11	-19021,9432	38065,9009	229,484196	4,83E-37
4037	-1,63084607	NA	NA	4,18E-05	NA	NA	NA	-2,66976045	0,40191971	0,02073197	0,0002118	0,00011864	0,47565984	-0,13600353	10	-19023,4183	38066,8488	230,432123	3,01E-37
1965	0,64576877	NA	NA	3,88E-05	-0,00183092	NA	-0,20438761	-2,58652225	NA	0,01772679	0,00020696	-0,00034396	0,48679163	NA	10	-19024,11	38068,2322	231,815457	1,50E-37
2966	-1,1987332	0,68175498	NA	4,69E-05	NA	0,40581671	NA	-1,91049389	NA	0,01549989	0,00020133	-0,00049532	NA	-0,09486783	10	-19024,1141	38068,2404	231,823643	1,50E-40
2968	-1,59179834	0,69737855	0,00852736	4,60E-05	NA	0,40932129	NA	-1,91049389	NA	0,01549989	0,00020133	-0,00049532	NA	-0,09486783	11	-19023,1977	38068,41	231,993235	1,38E-37
1973	-0,84339357	NA	NA	4,07E-05	NA	0,55505772	-0,13640315	-2,58652225	NA	0,01772679	0,00020696	-0,00034396	0,48679163	NA	10	-19024,3682	38068,7486	232,33189	1,16E-37
950	-0,67953035	0,68234123	NA	4,46E-05	NA	0,41541375	-0,13795142	-2,26386687	NA	0,01548996	0,00020413	-0,00046266	NA	NA	10	-19024,5365	38069,0852	232,668519	9,82E-38
1975	-0,50593593	NA	-0,00700816	4,14E-05	NA	0,55021643	-0,14061875	-2,58652225	NA	0,01772679	0,00020696	-0,00034396	0,48679163	NA	11	-19023,7352	38069,4849	233,068202	8,04E-38
3469	-0,28011525	NA	NA	4,24E-05	-0,0016491	NA	NA	-2,27275384	NA	0,01711404	0,00020255	NA	0,52288611	-0,14000571	9	-19025,7953	38069,6005	233,183753	7,59E-38
952	-1,04079715	0,69637088	0,00755047	4,39E-05	NA	0,41816409	-0,13461471	-2,26386687	NA	0,01548996	0,00020413	-0,00046266	NA	NA	11	-19023,8173	38069,6492	233,232462	7,41E-38
2878	-0,06671637	0,57575795	NA	4,34E-05	-0,00071955	0,41051829	-0,17478186	-1,2176888	NA	NA	0,00020569	-0,00045432	NA	-0,0941422	11	-19024,0221	38070,0589	233,642152	6,04E-38
1967	0,74969745	NA	-0,00245756	3,91E-05	-0,00181949	NA	-0,20513804	-2,58652225	NA	0,01772679	0,00020696	-0,00034396	0,48679163	NA	11	-19024,0344	38070,0835	233,666742	5,96E-40
2880	-0,47166397	0,58945108	0,00876766	4,25E-05	-0,00073797	0,4135805	-0,1717464	-1,2176888	NA	NA	0,00020569	-0,00045432	NA	-0,0941422	12	-19023,0479	38070,1132	233,696431	5,88E-38
2397	-0,31227401	NA	NA	4,32E-05	-0,00145314	0,47935513	NA	-1,66433243	0,4162362	NA	0,00021617	NA	NA	-0,08904476	9	-19026,2099	38070,4297	234,013025	5,02E-39
446	-0,06671637	0,57575795	NA	4,34E-05	-0,00071955	0,41051829	-0,17478186	-2,49206855	NA	0,01465098	0,00020366	NA	NA	NA	10	-19025,4032	38070,8187	234,401943	4,13E-38
448	-0,47166397	0,58945108	0,00876766	4,25E-05	-0,00073797	0,4135805	-0,1717464	-2,49206855	NA	0,01465098	0,00020366	NA	NA	NA	11	-19024,4291	38070,8727	234,456001	4,02E-38
3885	0,64576877	NA	NA	3,88E-05	-0,00183092	NA	-0,20438761	-1,25486087	NA	NA	0,00020772	-0,0003441	0,50313521	-0,14558566	10	-19025,467	38070,9462	234,529472	3,87E-38
928	-1,28042467	0,61751101	0,00965662	4,55E-05	-0,00054807	0,40424516	NA	-2,26386687	NA	0,01548996	0,00020413	-0,00046266	NA	NA	11	-19024,622	38071,2585	234,841792	3,31E-38
3893	-0,84339357	NA	NA	4,07E-05	NA	0,55505772	-0,13640315	-1,25486087	NA	NA	0,00020772	-0,0003441	0,50313521	-0,14558566	10	-19025,7252	38071,4626	235,045905	2,99E-38
3471	-0,2131598	NA	-0,0015413	4,25E-05	-0,00164139	NA	NA	-2,27275384	NA	0,01711404	0,00020255	NA	0,52288611	-0,14000571	10	-19025,7656	38071,5433	235,126577	2,87E-38
926	-0,85074914	0,60334436	NA	4,65E-05	-0,00052401	0,40049867	NA	-2,26386687	NA	0,01548996	0,00020413	-0,00046266	NA	NA	10	-19025,7978	38071,6078	235,191028	2,78E-38
2909	-0,31227401	NA	NA	4,32E-05	-0,00145314	0,47935513	NA	-1,73983563	0,43090563	NA	0,00021643	0,00010896	NA	-0,08710954	10	-19025,9202	38071,8525	235,435779	2,46E-38
3895	-0,50593593	NA	-0,00700816	4,14E-05	NA	0,55021643	-0,14061875	-1,25486087	NA	NA	0,00020772	-0,0003441	0,50313521	-0,14558566	11	-19025,0922	38072,1989	235,782217	2,07E-38
2399	-0,39160679	NA	0,00183718	4,30E-05	-0,00146197	0,48040455	NA	-1,66433243	0,4162362	NA	0,00021617	NA	NA	-0,08904476	10	-19026,1665	38072,3451	235,928415	1,92E-39
3887	0,74969745	NA	-0,00245756	3,91E-05	-0,00181949	NA	-0,20513804	-1,25486087	NA	NA	0,00020772	-0,0003441	0,50313521	-0,14558566	11	-19025,3914	38072,7975	236,380757	1,54E-38
1453	0,64576877	NA	NA	3,88E-05	-0,00183092	NA	-0,20438761	-2,75826159	NA	0,01709531	0,00020664	NA	0,4949792	NA	9	-19027,4985	38073,0069	236,590158	1,38E-38
1461	-0,84339357	NA	NA	4,07E-05	NA	0,55505772	-0,13640315	-2,75826159	NA	0,01709531	0,00020664	NA	0,4949792	NA	9	-19027,7567	38073,5233	237,106591	1,07E-38
2486	-0,67953035	0,68234123	NA	4,46E-05	NA	0,41541375	-0,13795142	-2,18775169	NA	0,01458917	0,00020109	NA	NA	-0,08533198	10	-19026,7999	38073,612	237,195274	1,02E-38
2911	-0,39160679	NA	0,00183718	4,30E-05	-0,00146197	0,48040455	NA	-1,73983563	0,43090563	NA	0,00021643	0,00010896	NA	-0,08710954	11	-19025,8768	38073,7681	237,351391	9,45E-39
1941	-1,36352414	NA	NA	4,29E-05	NA	0,54467715	NA	-2,58652225	NA	0,01772679	0,00020696	-0,00034396	0,48679163	NA	9	-19027,9086	38073,8272	237,410437	9,17E-39
2488	-1,04079715	0,69637088	0,00755047	4,39E-05	NA	0,41816409	-0,13461471	-2,18775169	NA	0,01458917	0,00020109	NA	NA	-0,08533198	11	-19026,0807	38074,1759	237,759217	7,71E-39
1463	-0,50593593	NA	-0,00700816	4,14E-05	NA	0,55021643	-0,14061875	-2,75826159	NA	0,01709531	0,00020664	NA	0,4949792	NA	10	-19027,1236	38074,2594	237,842682	7,39E-39
1455	0,74969745	NA	-0,00245756	3,91E-05	-0,00181949	NA	-0,20513804	-2,75826159	NA	0,01709531	0,00020664	NA	0,4949792	NA	10	-19027,4229	38074,8579	238,441221	5,48E-39
1943	-1,08960867	NA	-0,00576757	4,36E-05	NA	0,53973144	NA	-2,58652225	NA	0,01772679	0,00020696	-0,00034396	0,48679163	NA	10	-19027,4763	38074,9648	238,548071	5,19E-39
918	-1,1987332	0,68175498	NA	4,69E-05	NA	0,40581671	NA	-2,26386687	NA	0,01548996	0,00020413	-0,00046266	NA	NA	9	-19028,7117	38075,4333	239,016573	4,11E-39
920	-1,59179834	0,69737855	0,00852736	4,60E-05	NA	0,40932129	NA	-2,26386687	NA	0,01548996	0,00020413	-0,00046266	NA	NA	10	-19027,7952	38075,6027	239,185943	3,78E-39
3373	0,64576877	NA	NA	3,88E-05	-0,00183092	NA	-0,20438761	-1,4783871	NA	NA	0,00020739	NA	0,51055393	-0,13912561	9	-19028,8609	38075,7318	239,315108	3,54E-39
2464	-1,28042467	0,61751101	0,00965662	4,55E-05	-0,00054807	0,40424516	NA	-2,18775169	NA	0,01458917	0,00020109	NA	NA	-0,08533198	11	-19026,8853	38075,7853	239,368548	3,45E-39
2462	-0,85074914	0,60334436	NA	4,65E-05	-0,00052401	0,40049867	NA	-2,18775169	NA	0,01458917	0,00020109	NA	NA	-0,08533198	10	-19028,0612	38076,1345	239,717784	2,89E-39

3381	-0,84339357	NA	NA	4,07E-05	NA	0,55505772	-0,13640315	-1,4783871	NA	NA	0,00020739	NA	0,51055393	-0,13912561	9	-19029,1192	38076,2483	239,831541	2,73E-39
349	-0,31227401	NA	NA	4,32E-05	-0,00145314	0,47935513	NA	-1,97841134	0,41538406	NA	0,00021885	NA	NA	NA	8	-19030,201	38076,41	239,993298	2,52E-39
3861	-1,36352414	NA	NA	4,29E-05	NA	0,54467715	NA	-1,25486087	NA	NA	0,00020772	-0,0003441	0,50313521	-0,14558566	9	-19029,2656	38076,5412	240,124452	2,36E-39
3383	-0,50593593	NA	-0,00700816	4,14E-05	NA	0,55021643	-0,14061875	-1,4783871	NA	NA	0,00020739	NA	0,51055393	-0,13912561	10	-19028,4861	38076,9844	240,567632	1,89E-39
830	-0,06671637	0,57575795	NA	4,34E-05	-0,00071955	0,41051829	-0,17478186	-1,56810442	NA	NA	0,00020849	-0,0004232	NA	NA	10	-19028,5651	38077,1424	240,725696	1,75E-39
832	-0,47166397	0,58945108	0,00876766	4,25E-05	-0,00073797	0,4135805	-0,1717464	-1,56810442	NA	NA	0,00020849	-0,0004232	NA	NA	11	-19027,5909	38077,1965	240,779753	1,70E-40
861	-0,31227401	NA	NA	4,32E-05	-0,00145314	0,47935513	NA	-2,0661368	0,43415925	NA	0,00021911	0,00013912	NA	NA	9	-19029,7231	38077,4561	241,039426	1,49E-39
3375	0,74969745	NA	-0,00245756	3,91E-05	-0,00181949	NA	-0,20513804	-1,4783871	NA	NA	0,00020739	NA	0,51055393	-0,13912561	10	-19028,7854	38077,5829	241,166171	1,40E-39
3863	-1,08960867	NA	-0,00576757	4,36E-05	NA	0,53973144	NA	-1,25486087	NA	NA	0,00020772	-0,0003441	0,50313521	-0,14558566	10	-19028,8333	38077,6788	241,262086	1,34E-39
351	-0,39160679	NA	0,00183718	4,30E-05	-0,00146197	0,48040455	NA	-1,97841134	0,41538406	NA	0,00021885	NA	NA	NA	9	-19030,1576	38078,3252	241,908467	9,68E-40
2870	-0,67953035	0,68234123	NA	4,46E-05	NA	0,41541375	-0,13795142	-1,2176888	NA	NA	0,00020569	-0,00045432	NA	-0,0941422	10	-19029,2087	38078,4297	242,012944	9,19E-40
1429	-1,36352414	NA	NA	4,29E-05	NA	0,54467715	NA	-2,75826159	NA	0,01709531	0,00020664	NA	0,4949792	NA	8	-19031,2971	38078,6021	242,185359	8,43E-40
2872	-1,04079715	0,69637088	0,00755047	4,39E-05	NA	0,41816409	-0,13461471	-1,2176888	NA	NA	0,00020569	-0,00045432	NA	-0,0941422	11	-19028,4895	38078,9936	242,576887	6,93E-40
438	-0,67953035	0,68234123	NA	4,46E-05	NA	0,41541375	-0,13795142	-2,49206855	NA	0,01465098	0,00020366	NA	NA	NA	9	-19030,5899	38079,1897	242,772957	6,28E-40
1511	-0,65771633	NA	-0,01187559	4,14E-05	NA	NA	-0,10732692	-3,07214819	0,38813022	0,02082345	0,00021556	NA	0,44803127	NA	10	-19029,625	38079,2622	242,845525	6,06E-41
863	-0,39160679	NA	0,00183718	4,30E-05	-0,00146197	0,48040455	NA	-2,0661368	0,43415925	NA	0,00021911	0,00013912	NA	NA	10	-19029,6797	38079,3715	242,954816	5,74E-40
1431	-1,08960867	NA	-0,00576757	4,36E-05	NA	0,53973144	NA	-2,75826159	NA	0,01709531	0,00020664	NA	0,4949792	NA	9	-19030,8648	38079,7395	243,322772	4,77E-40
2366	-0,06671637	0,57575795	NA	4,34E-05	-0,00071955	0,41051829	-0,17478186	-1,50990804	NA	NA	0,00020523	NA	NA	-0,08563427	10	-19029,8674	38079,7471	243,330334	4,75E-40
440	-1,04079715	0,69637088	0,00755047	4,39E-05	NA	0,41816409	-0,13461471	-2,49206855	NA	0,01465098	0,00020366	NA	NA	NA	10	-19029,8706	38079,7534	243,336678	4,74E-40
2368	-0,47166397	0,58945108	0,00876766	4,25E-05	-0,00073797	0,4135805	-0,1717464	-1,50990804	NA	NA	0,00020523	NA	NA	-0,08563427	11	-19028,8933	38079,8011	243,384391	4,63E-40
2023	-0,65771633	NA	-0,01187559	4,14E-05	NA	NA	-0,10732692	-3,17508673	0,41103069	0,02071909	0,00021591	0,0001702	0,4495725	NA	11	-19028,9102	38079,8349	243,418205	4,55E-40
2454	-1,1987332	0,68175498	NA	4,69E-05	NA	0,40581671	NA	-2,18775169	NA	0,01458917	0,00020109	NA	NA	-0,08533198	9	-19030,975	38079,9601	243,543328	4,27E-40
2456	-1,59179834	0,69737855	0,00852736	4,60E-05	NA	0,40932129	NA	-2,18775169	NA	0,01458917	0,00020109	NA	NA	-0,08533198	10	-19030,0586	38080,1294	243,712698	3,93E-40
2848	-1,28042467	0,61751101	0,00965662	4,55E-05	-0,00054807	0,40424516	NA	-1,2176888	NA	NA	0,00020569	-0,00045432	NA	-0,0941422	11	-19029,2942	38080,6029	244,186217	3,10E-41
1509	-1,22822825	NA	NA	4,02E-05	NA	NA	-0,09995451	-3,07214819	0,38813022	0,02082345	0,00021556	NA	0,44803127	NA	9	-19031,3695	38080,7489	244,332207	2,88E-40
2846	-0,85074914	0,60334436	NA	4,65E-05	-0,00052401	0,40049867	NA	-1,2176888	NA	NA	0,00020569	-0,00045432	NA	-0,0941422	10	-19030,47	38080,9522	244,535454	2,60E-40
2021	-1,22822825	NA	NA	4,02E-05	NA	NA	-0,09995451	-3,17508673	0,41103069	0,02071909	0,00021591	0,0001702	0,4495725	NA	10	-19030,6546	38081,3214	244,904665	2,16E-40
3349	-1,36352414	NA	NA	4,29E-05	NA	0,54467715	NA	-1,4783871	NA	NA	0,00020739	NA	0,51055393	-0,13912561	8	-19032,6595	38081,327	244,910309	2,16E-40
416	-1,28042467	0,61751101	0,00965662	4,55E-05	-0,00054807	0,40424516	NA	-2,49206855	NA	0,01465098	0,00020366	NA	NA	NA	10	-19030,6753	38081,3627	244,946008	2,12E-40
1479	-1,14142692	NA	-0,01089562	4,31E-05	NA	NA	NA	-3,07214819	0,38813022	0,02082345	0,00021556	NA	0,44803127	NA	9	-19031,7391	38081,4882	245,071515	1,99E-40
414	-0,85074914	0,60334436	NA	4,65E-05	-0,00052401	0,40049867	NA	-2,49206855	NA	0,01465098	0,00020366	NA	NA	NA	9	-19031,8511	38081,7122	245,295466	1,78E-40
1991	-1,14142692	NA	-0,01089562	4,31E-05	NA	NA	NA	-3,17508673	0,41103069	0,02071909	0,00021591	0,0001702	0,4495725	NA	10	-19031,0243	38082,0607	245,643973	1,50E-40
1477	-1,63084607	NA	NA	4,18E-05	NA	NA	NA	-3,07214819	0,38813022	0,02082345	0,00021556	NA	0,44803127	NA	8	-19033,2143	38082,4366	246,019885	1,24E-40
3351	-1,08960867	NA	-0,00576757	4,36E-05	NA	0,53973144	NA	-1,4783871	NA	NA	0,00020739	NA	0,51055393	-0,13912561	9	-19032,2272	38082,4644	246,047722	1,22E-40
1933	-0,28011525	NA	NA	4,24E-05	-0,0016491	NA	NA	-2,58652225	NA	0,01772679	0,00020696	-0,00034396	0,48679163	NA	9	-19032,4003	38082,8106	246,393856	1,03E-40
1989	-1,63084607	NA	NA	4,18E-05	NA	NA	NA	-3,17508673	0,41103069	0,02071909	0,00021591	0,0001702	0,4495725	NA	9	-19032,4994	38083,0088	246,592121	9,31E-41
1935	-0,2131598	NA	-0,0015413	4,25E-05	-0,00164139	NA	NA	-2,58652225	NA	0,01772679	0,00020696	-0,00034396	0,48679163	NA	10	-19032,3706	38084,7534	248,336681	3,89E-41
2838	-1,1987332	0,68175498	NA	4,69E-05	NA	0,40581671	NA	-1,2176888	NA	NA	0,00020569	-0,00045432	NA	-0,0941422	9	-19033,3839	38084,7777	248,360998	3,84E-41
2840	-1,59179834	0,69737855	0,00852736	4,60E-05	NA	0,40932129	NA	-1,2176888	NA	NA	0,00020569	-0,00045432	NA	-0,0941422	10	-19032,4675	38084,9471	248,530368	3,53E-41
318	-0,06671637	0,57575795	NA	4,34E-05	-0,00071955	0,41051829	-0,17478186	-1,812439	NA	NA	0,00020783	NA	NA	NA	9	-19033,692	38085,3939	248,977144	2,82E-42
320	-0,47166397	0,58945108	0,00876766	4,25E-05	-0,00073797	0,4135805	-0,1717464	-1,812439	NA	NA	0,00020783	NA	NA	NA	10	-19032,7178	38085,4477	249,03098	2,75E-41
822	-0,67953035	0,68234123	NA	4,46E-05	NA	0,41541375	-0,13795142	-1,56810442	NA	NA	0,00020849	-0,0004232	NA	NA	9	-19033,7517	38085,5134	249,096709	2,66E-41
3853	-0,28011525	NA	NA	4,24E-05	-0,0016491	NA	NA	-1,25486087	NA	NA	0,00020772	-0,0003441	0,50313521	-0,14558566	9	-19033,7573	38085,5246	249,107871	2,65E-41
406	-1,1987332	0,68175498	NA	4,69E-05	NA	0,40581671	NA	-2,49206855	NA	0,01465098	0,00020366	NA	NA	NA	8	-19034,765	38085,538	249,121232	2,63E-41
408	-1,59179834	0,69737855	0,00852736	4,60E-05	NA	0,40932129	NA	-2,49206855	NA	0,01465098	0,00020366	NA	NA	NA	9	-19033,8486	38085,7071	249,29038	2,41E-41
824	-1,04079715	0,69637088	0,00755047	4,39E-05	NA	0,41816409	-0,13461471	-1,56810442	NA	NA	0,00020849	-0,0004232	NA	NA	10	-19033,0325	38086,0772	249,66043	2,01E-41
3855	-0,2131598	NA	-0,0015413	4,25E-05	-0,00164139	NA	NA	-1,25486087	NA	NA	0,00020772	-0,0003441	0,50313521	-0,14558566	10	-19033,7276	38087,4674	251,050696	1,00E-41
1421	-0,28011525	NA	NA	4,24E-05	-0,0016491	NA	NA	-2,75826159	NA	0,01709531	0,00020664	NA	0,4949792	NA	8	-19035,7888	38087,5855	251,168779	9,44E-42
800	-1,28042467	0,61751101	0,00965662	4,55E-05	-0,00054807	0,40424516	NA	-1,56810442	NA	NA	0,00020849	-0,0004232	NA	NA	10	-19033,8372	38087,6865	251,269761	8,98E-43
798	-0,85074914	0,60334436	NA	4,65E-05	-0,00052401	0,40049867	NA	-1,56810442	NA	NA	0,00020849	-0,0004232	NA	NA	9	-19035,013	38088,0359	251,619219	7,54E-42
2358	-0,67953035	0,68234123	NA	4,46E-05	NA	0,41541375	-0,13795142	-1,50990804	NA	NA	0,00020523	NA	NA	-0,08563427	9	-19035,0541	38088,1181	251,701347	7,23E-42
2360	-1,04079715	0,69637088	0,00755047	4,39E-05	NA	0,41816409	-0,13461471	-1,50990804	NA	NA	0,00020523	NA	NA	-0,08563427	10	-19034,3348	38088,6818	252,265068	5,46E-42
1423	-0,2131598	NA	-0,0015413	4,25E-05	-0,00164139	NA	NA	-2,75826159	NA	0,01709531	0,00020664	NA	0,4949792	NA	9	-19035,7591	38089,5281	253,111382	3,57E-42
2336	-1,28042467	0,61751101	0,00965662	4,55E-05	-0,00054807	0,40424516	NA	-1,50990804	NA	NA	0,00020523	NA	NA	-0,08563427	10	-19035,1395	38090,2911	253,874399	2,44E-42
1837	0,64576877	NA	NA	3,88E-05	-0,00183092	NA	-0,20438761	-1,78635429	NA	NA	0,0002119	-0,00029974	0,47515083	NA	9	-19036,1488	38090,3075	253,890816	2,42E-42
3341	-0,28011525	NA	NA	4,24E-05	-0,0016491	NA	NA	-1,4783871	NA	NA	0,00020739	NA	0,51055393	-0,13912561	8	-19037,1512	38090,3105	253,893729	2,42E-42
2334	-0,85074914	0,60334436	NA	4,65E-05	-0,00052401	0,40049867	NA	-1,50990804	NA	NA	0,00020523	NA	NA	-0,08563427	9	-19036,3153	38090,6406	254,223857	2,05E-42
1845	-0,84339357	NA	NA	4,07E-05	NA	0,55505772	-0,13640315	-1,78635429	NA	NA	0,0002119	-0,00029974	0,47515083	NA	9	-19036,407	38090,824	254,407249	1,87E-42
1847	-0,50593593	NA	-0,00700816	4,14E-05	NA	0,55021643	-0,14061875	-1,78635429	NA	NA	0,0002119	-0,00029974	0,47515083	NA	10	-19035,7739	38091,5601	255,143339	1,29E-42
790	-1,1987332	0,68175498	NA	4,69E-05	NA	0,40581671	NA	-1,56810442	NA	NA	0,00020849	-0,0004232	NA	NA	8	-19037,9269	38091,8617	255,444985	1,11E-42
792	-1,59179834	0,69737855	0,00852736	4,60E-05	NA	0,40932129	NA	-1,56810442	NA	NA	0,00020849	-0,0004232	NA	NA	9	-19037,0105	38092,0309	255,614133	1,02E-42

1839	0,74969745	NA	-0,00245756	3,91E-05	-0,00181949	NA	-0,20513804	-1,78635429	NA	NA	0,0002119	-0,00029974	0,47515083	NA	10	-19036,0732	38092,1586	255,741879	9,59E-43
3343	-0,2131598	NA	-0,0015413	4,25E-05	-0,00164139	NA	NA	-1,4783871	NA	NA	0,00020739	NA	0,51055393	-0,13912561	9	-19037,1215	38092,2531	255,836332	9,15E-43
1325	0,64576877	NA	NA	3,88E-05	-0,00183092	NA	-0,20438761	-1,96172815	NA	NA	0,00021146	NA	0,48287384	NA	8	-19038,7537	38093,5153	257,098611	4,87E-44
3431	-0,65771633	NA	-0,01187559	4,14E-05	NA	NA	-0,10732692	-1,61349146	0,35721774	NA	0,00021669	NA	0,46315983	-0,13737061	10	-19036,8391	38093,6905	257,273733	4,46E-43
310	-0,67953035	0,68234123	NA	4,46E-05	NA	0,41541375	-0,13795142	-1,812439	NA	NA	0,00020783	NA	NA	NA	8	-19038,8786	38093,7651	257,348379	4,30E-43
1333	-0,84339357	NA	NA	4,07E-05	NA	0,55505772	-0,13640315	-1,96172815	NA	NA	0,00021146	NA	0,48287384	NA	8	-19039,0119	38094,0318	257,615044	3,76E-43
312	-1,04079715	0,69637088	0,00755047	4,39E-05	NA	0,41816409	-0,13461471	-1,812439	NA	NA	0,00020783	NA	NA	NA	9	-19038,1593	38094,3286	257,911878	3,24E-43
2326	-1,1987332	0,68175498	NA	4,69E-05	NA	0,40581671	NA	-1,50990804	NA	NA	0,00020523	NA	NA	-0,08563427	8	-19039,2292	38094,4663	258,049623	3,03E-43
2328	-1,59179834	0,69737855	0,00852736	4,60E-05	NA	0,40932129	NA	-1,50990804	NA	NA	0,00020523	NA	NA	-0,08563427	9	-19038,3128	38094,6355	258,218771	2,78E-43
3943	-0,65771633	NA	-0,01187559	4,14E-05	NA	NA	-0,10732692	-1,71083893	0,37612338	NA	0,00021703	0,00013898	0,46389079	-0,13469622	11	-19036,3659	38094,7464	258,329665	2,63E-43
1335	-0,50593593	NA	-0,00700816	4,14E-05	NA	0,55021643	-0,14061875	-1,96172815	NA	NA	0,00021146	NA	0,48287384	NA	9	-19038,3788	38094,7676	258,350913	2,60E-43
3429	-1,22822825	NA	NA	4,02E-05	NA	NA	-0,09995451	-1,61349146	0,35721774	NA	0,00021669	NA	0,46315983	-0,13737061	9	-19038,5836	38095,1771	258,760415	2,12E-43
1327	0,74969745	NA	-0,00245756	3,91E-05	-0,00181949	NA	-0,20513804	-1,96172815	NA	NA	0,00021146	NA	0,48287384	NA	9	-19038,6781	38095,3662	258,949452	1,93E-43
1813	-1,36352414	NA	NA	4,29E-05	NA	0,54467715	NA	-1,78635429	NA	NA	0,0002119	-0,00029974	0,47515083	NA	8	-19039,9474	38095,9027	259,486017	1,48E-43
3399	-1,14142692	NA	-0,01089562	4,31E-05	NA	NA	NA	-1,61349146	0,35721774	NA	0,00021669	NA	0,46315983	-0,13737061	9	-19038,9532	38095,9164	259,499723	1,47E-43
288	-1,28042467	0,61751101	0,00965662	4,55E-05	-0,00054807	0,40424516	NA	-1,812439	NA	NA	0,00020783	NA	NA	NA	9	-19038,964	38095,9379	259,521209	1,45E-43
2541	0,64576877	NA	NA	3,88E-05	-0,00183092	NA	-0,20438761	-2,55577604	0,44288659	0,01897716	0,00021141	NA	NA	-0,08883356	10	-19038,0304	38096,0729	259,656146	1,36E-43
3941	-1,22822825	NA	NA	4,02E-05	NA	NA	-0,09995451	-1,71083893	0,37612338	NA	0,00021703	0,00013898	0,46389079	-0,13469622	10	-19038,1103	38096,2328	259,816125	1,25E-43
286	-0,85074914	0,60334436	NA	4,65E-05	-0,00052401	0,40049867	NA	-1,812439	NA	NA	0,00020783	NA	NA	NA	8	-19040,1398	38096,2876	259,870888	1,22E-43
2549	-0,84339357	NA	NA	4,07E-05	NA	0,55505772	-0,13640315	-2,55577604	0,44288659	0,01897716	0,00021141	NA	NA	-0,08883356	10	-19038,2886	38096,5893	260,172579	1,05E-43
3397	-1,63084607	NA	NA	4,18E-05	NA	NA	NA	-1,61349146	0,35721774	NA	0,00021669	NA	0,46315983	-0,13737061	8	-19040,4284	38096,8648	260,448092	9,12E-44
3911	-1,14142692	NA	-0,01089562	4,31E-05	NA	NA	NA	-1,71083893	0,37612338	NA	0,00021703	0,00013898	0,46389079	-0,13469622	10	-19038,48	38096,9722	260,555433	8,64E-44
1815	-1,08960867	NA	-0,00576757	4,36E-05	NA	0,53973144	NA	-1,78635429	NA	NA	0,0002119	-0,00029974	0,47515083	NA	9	-19039,5151	38097,0402	260,62343	8,36E-44
2551	-0,50593593	NA	-0,00700816	4,14E-05	NA	0,55021643	-0,14061875	-2,55577604	0,44288659	0,01897716	0,00021141	NA	NA	-0,08883356	11	-19037,6555	38097,3256	260,908891	7,24E-45
3053	0,64576877	NA	NA	3,88E-05	-0,00183092	NA	-0,20438761	-2,61719209	0,45517288	0,01892094	0,00021164	9,21E-05	NA	-0,08713002	11	-19037,8248	38097,6642	261,247511	6,12E-44
3909	-1,63084607	NA	NA	4,18E-05	NA	NA	NA	-1,71083893	0,37612338	NA	0,00021703	0,00013898	0,46389079	-0,13469622	9	-19039,9552	38097,9203	261,503581	5,38E-44
2543	0,74969745	NA	-0,00245756	3,91E-05	-0,00181949	NA	-0,20513804	-2,55577604	0,44288659	0,01897716	0,00021141	NA	NA	-0,08883356	11	-19037,9548	38097,9242	261,507431	5,37E-46
3061	-0,84339357	NA	NA	4,07E-05	NA	0,55505772	-0,13640315	-2,61719209	0,45517288	0,01892094	0,00021164	9,21E-05	NA	-0,08713002	11	-19038,083	38098,1807	261,763944	4,72E-44
3063	-0,50593593	NA	-0,00700816	4,14E-05	NA	0,55021643	-0,14061875	-2,61719209	0,45517288	0,01892094	0,00021164	9,21E-05	NA	-0,08713002	12	-19037,45	38098,9172	262,500478	3,27E-44
1301	-1,36352414	NA	NA	4,29E-05	NA	0,54467715	NA	-1,96172815	NA	NA	0,00021146	NA	0,48287384	NA	7	-19042,5523	38099,1108	262,694034	2,97E-44
3055	0,74969745	NA	-0,00245756	3,91E-05	-0,00181949	NA	-0,20513804	-2,61719209	0,45517288	0,01892094	0,00021164	9,21E-05	NA	-0,08713002	12	-19037,7492	38099,5157	263,099017	2,42E-44
278	-1,1987332	0,68175498	NA	4,69E-05	NA	0,40581671	NA	-1,812439	NA	NA	0,00020783	NA	NA	NA	7	-19043,0537	38100,1136	263,696876	1,80E-45
1303	-1,08960867	NA	-0,00576757	4,36E-05	NA	0,53973144	NA	-1,96172815	NA	NA	0,00021146	NA	0,48287384	NA	8	-19042,12	38100,2479	263,831225	1,68E-44
280	-1,59179834	0,69737855	0,00852736	4,60E-05	NA	0,40932129	NA	-1,812439	NA	NA	0,00020783	NA	NA	NA	8	-19042,1373	38100,2825	263,865803	1,65E-44
2517	-1,36352414	NA	NA	4,29E-05	NA	0,54467715	NA	-2,55577604	0,44288659	0,01897716	0,00021141	NA	NA	-0,08883356	9	-19041,8289	38101,6678	265,251126	8,26E-45
493	0,64576877	NA	NA	3,88E-05	-0,00183092	NA	-0,20438761	-2,87228819	0,44207022	0,01904584	0,00021405	NA	NA	NA	9	-19041,9846	38101,9792	265,562447	7,07E-45
501	-0,84339357	NA	NA	4,07E-05	NA	0,55505772	-0,13640315	-2,87228819	0,44207022	0,01904584	0,00021405	NA	NA	NA	9	-19042,2428	38102,4956	266,07888	5,46E-45
2990	-0,14195779	0,65649184	NA	4,36E-05	-0,00074998	NA	-0,15445798	-1,91049389	NA	0,01549989	0,00020133	-0,00049532	NA	-0,09486783	11	-19040,2815	38102,5776	266,160921	5,24E-45
2519	-1,08960867	NA	-0,00576757	4,36E-05	NA	0,53973144	NA	-2,55577604	0,44288659	0,01897716	0,00021141	NA	NA	-0,08883356	10	-19041,3967	38102,8055	266,388761	4,68E-45
1005	0,64576877	NA	NA	3,88E-05	-0,00183092	NA	-0,20438761	-2,9468992	0,4586198	0,01897353	0,00021429	0,00012376	NA	NA	10	-19041,6092	38103,2306	266,813902	3,78E-45
503	-0,50593593	NA	-0,00700816	4,14E-05	NA	0,55021643	-0,14061875	-2,87228819	0,44207022	0,01904584	0,00021405	NA	NA	NA	10	-19041,6098	38103,2317	266,814971	3,78E-45
3029	-1,36352414	NA	NA	4,29E-05	NA	0,54467715	NA	-2,61719209	0,45517288	0,01892094	0,00021164	9,21E-05	NA	-0,08713002	10	-19041,6234	38103,259	266,842269	3,73E-45
2992	-0,45110821	0,66723944	0,00674107	4,29E-05	-0,00076432	NA	-0,15224001	-1,91049389	NA	0,01549989	0,00020133	-0,00049532	NA	-0,09486783	12	-19039,7234	38103,464	267,047256	3,37E-45
1013	-0,84339357	NA	NA	4,07E-05	NA	0,55505772	-0,13640315	-2,9468992	0,4586198	0,01897353	0,00021429	0,00012376	NA	NA	10	-19041,8674	38103,7471	267,330334	2,92E-45
495	0,74969745	NA	-0,00245756	3,91E-05	-0,00181949	NA	-0,20513804	-2,87228819	0,44207022	0,01904584	0,00021405	NA	NA	NA	10	-19041,909	38103,8302	267,41351	2,80E-45
3031	-1,08960867	NA	-0,00576757	4,36E-05	NA	0,53973144	NA	-2,61719209	0,45517288	0,01892094	0,00021164	9,21E-05	NA	-0,08713002	11	-19041,1911	38104,3968	267,980125	2,11E-45
1015	-0,50593593	NA	-0,00700816	4,14E-05	NA	0,55021643	-0,14061875	-2,9468992	0,4586198	0,01897353	0,00021429	0,00012376	NA	NA	11	-19041,2344	38104,4834	268,066647	2,02E-45
1805	-0,28011525	NA	NA	4,24E-05	-0,0016491	NA	NA	-1,78635429	NA	NA	0,0002119	-0,00029974	0,47515083	NA	8	-19044,4391	38104,8862	268,469436	1,65E-45
1007	0,74969745	NA	-0,00245756	3,91E-05	-0,00181949	NA	-0,20513804	-2,9468992	0,4586198	0,01897353	0,00021429	0,00012376	NA	NA	11	-19041,5337	38105,0819	268,665186	1,50E-45
1807	-0,2131598	NA	-0,0015413	4,25E-05	-0,00164139	NA	NA	-1,78635429	NA	NA	0,0002119	-0,00029974	0,47515083	NA	9	-19044,4094	38106,8288	270,41204	6,26E-46
469	-1,36352414	NA	NA	4,29E-05	NA	0,54467715	NA	-2,87228819	0,44207022	0,01904584	0,00021405	NA	NA	NA	8	-19045,7832	38107,5744	271,157648	4,31E-46
1293	-0,28011525	NA	NA	4,24E-05	-0,0016491	NA	NA	-1,96172815	NA	NA	0,00021146	NA	0,48287384	NA	7	-19047,044	38108,0942	271,677453	3,32E-47
471	-1,08960867	NA	-0,00576757	4,36E-05	NA	0,53973144	NA	-2,87228819	0,44207022	0,01904584	0,00021405	NA	NA	NA	9	-19045,3509	38108,7118	272,295061	2,44E-46
981	-1,36352414	NA	NA	4,29E-05	NA	0,54467715	NA	-2,9468992	0,4586198	0,01897353	0,00021429	0,00012376	NA	NA	9	-19045,4078	38108,8256	272,408881	2,31E-46
942	-0,14195779	0,65649184	NA	4,36E-05	-0,00074998	NA	-0,15445798	-2,26386687	NA	0,01548996	0,00020413	-0,00046266	NA	NA	10	-19044,8791	38109,7704	273,353629	1,44E-46
983	-1,08960867	NA	-0,00576757	4,36E-05	NA	0,53973144	NA	-2,9468992	0,4586198	0,01897353	0,00021429	0,00012376	NA	NA	10	-19044,9755	38109,9632	273,546516	1,31E-47
1295	-0,2131598	NA	-0,0015413	4,25E-05	-0,00164139	NA	NA	-1,96172815	NA	NA	0,00021146	NA	0,48287384	NA	8	-19047,0143	38110,0366	273,619835	1,26E-46
1383	-0,65771633	NA	-0,01187559	4,14E-05	NA	NA	-0,10732692	-2,09152745	0,35956015	NA	0,00022076	NA	0,43622845	NA	9	-19046,2014	38110,4128	273,99609	1,04E-46
2958	-0,8455927	0,67951403	NA	4,64E-05	-0,00057321	NA	NA	-1,91049389	NA	0,01549989	0,00020133	-0,00049532	NA	-0,09486783	10	-19045,302	38110,6162	274,199432	9,42E-47
2509	-0,28011525	NA	NA	4,24E-05	-0,0016491	NA	NA	-2,55577604	0,44288659	0,01897716	0,00021141	NA	NA	-0,08883356	9	-19046,3207	38110,6513	274,234545	9,25E-47
944	-0,45110821	0,66723944	0,00674107	4,29E-05	-0,00076432	NA	-0,15224001	-2,26386687	NA	0,01548996	0,00020413	-0,00046266	NA	NA	11	-19044,3209	38110,6565	274,239742	9,23E-47

1895	-0,65771633	NA	-0,01187559	4,14E-05	NA	NA	-0,10732692	-2,2103983	0,38500536	NA	0,00022113	0,00018745	0,43806177	NA	10	-19045,3274	38110,6669	274,250181	9,18E-47
2960	-1,17938783	0,69098467	0,00751019	4,56E-05	-0,00059207	NA	NA	-1,91049389	NA	0,01549989	0,00020133	-0,00049532	NA	-0,09486783	11	-19044,612	38111,2387	274,821967	6,90E-47
2982	-0,79586264	0,77122371	NA	4,50E-05	NA	NA	-0,11503973	-1,91049389	NA	0,01549989	0,00020133	-0,00049532	NA	-0,09486783	10	-19045,8757	38111,7636	275,346861	5,31E-47
1381	-1,22822825	NA	NA	4,02E-05	NA	NA	-0,09995451	-2,09152745	0,35956015	NA	0,00022076	NA	0,43622845	NA	8	-19047,9459	38111,8997	275,482993	4,96E-47
1893	-1,22822825	NA	NA	4,02E-05	NA	NA	-0,09995451	-2,2103983	0,38500536	NA	0,00022113	0,00018745	0,43806177	NA	9	-19047,0718	38112,1536	275,736863	4,37E-47
3021	-0,28011525	NA	NA	4,24E-05	-0,0016491	NA	NA	-2,61719209	0,45517288	0,01892094	0,00021164	9,21E-05	NA	-0,08713002	10	-19046,1151	38112,2424	275,825688	4,18E-47
2511	-0,2131598	NA	-0,0015413	4,25E-05	-0,00164139	NA	NA	-2,55577604	0,44288659	0,01897716	0,00021141	NA	NA	-0,08883356	10	-19046,291	38112,5941	276,17737	3,50E-47
1351	-1,14142692	NA	-0,01089562	4,31E-05	NA	NA	NA	-2,09152745	0,35956015	NA	0,00022076	NA	0,43622845	NA	8	-19048,3155	38112,639	276,222301	3,43E-47
1863	-1,14142692	NA	-0,01089562	4,31E-05	NA	NA	NA	-2,2103983	0,38500536	NA	0,00022113	0,00018745	0,43806177	NA	9	-19047,4415	38112,8929	276,476171	3,02E-47
2984	-1,05409025	0,78157333	0,00543311	4,44E-05	NA	NA	-0,11273577	-1,91049389	NA	0,01549989	0,00020133	-0,00049532	NA	-0,09486783	11	-19045,5154	38113,0454	276,628718	2,80E-47
1349	-1,63084607	NA	NA	4,18E-05	NA	NA	NA	-2,09152745	0,35956015	NA	0,00022076	NA	0,43622845	NA	7	-19049,7907	38113,5876	277,170893	2,13E-47
1861	-1,63084607	NA	NA	4,18E-05	NA	NA	NA	-2,2103983	0,38500536	NA	0,00022113	0,00018745	0,43806177	NA	8	-19048,9167	38113,8413	277,42454	1,88E-47
3023	-0,2131598	NA	-0,0015413	4,25E-05	-0,00164139	NA	NA	-2,61719209	0,45517288	0,01892094	0,00021164	9,21E-05	NA	-0,08713002	11	-19046,0854	38114,1855	277,768735	1,58E-47
2478	-0,14195779	0,65649184	NA	4,36E-05	-0,00074998	NA	-0,15445798	-2,18775169	NA	0,01458917	0,00020109	NA	NA	-0,08533198	10	-19047,1425	38114,2971	277,880384	1,50E-47
2480	-0,45110821	0,66723944	0,00674107	4,29E-05	-0,00076432	NA	-0,15224001	-2,18775169	NA	0,01458917	0,00020109	NA	NA	-0,08533198	11	-19046,5843	38115,1832	278,766497	9,60E-48
2950	-1,235734	0,76857786	NA	4,69E-05	NA	NA	NA	-1,91049389	NA	0,01549989	0,00020133	-0,00049532	NA	-0,09486783	9	-19048,7744	38115,5587	279,141933	7,96E-48
461	-0,28011525	NA	NA	4,24E-05	-0,0016491	NA	NA	-2,87228819	0,44207022	0,01904584	0,00021405	NA	NA	NA	8	-19050,2749	38116,5578	280,141068	4,83E-48
2952	-1,52051301	0,78035551	0,00624724	4,63E-05	NA	NA	NA	-1,91049389	NA	0,01549989	0,00020133	-0,00049532	NA	-0,09486783	10	-19048,2983	38116,6087	280,191964	4,71E-48
973	-0,28011525	NA	NA	4,24E-05	-0,0016491	NA	NA	-2,9468992	0,4586198	0,01897353	0,00021429	0,00012376	NA	NA	9	-19049,8995	38117,809	281,3923	2,58E-48
910	-0,8455927	0,67951403	NA	4,64E-05	-0,00057321	NA	NA	-2,26386687	NA	0,01548996	0,00020413	-0,00046266	NA	NA	9	-19049,8996	38117,8091	281,392362	2,58E-48
912	-1,17938783	0,69098467	0,00751019	4,56E-05	-0,00059207	NA	NA	-2,26386687	NA	0,01548996	0,00020413	-0,00046266	NA	NA	10	-19049,2096	38118,4314	282,014675	1,89E-48
463	-0,2131598	NA	-0,0015413	4,25E-05	-0,00164139	NA	NA	-2,87228819	0,44207022	0,01904584	0,00021405	NA	NA	NA	9	-19050,2452	38118,5004	282,083671	1,83E-48
934	-0,79586264	0,77122371	NA	4,50E-05	NA	NA	-0,11503973	-2,26386687	NA	0,01548996	0,00020413	-0,00046266	NA	NA	9	-19050,4733	38118,9565	282,53979	1,46E-48
2862	-0,14195779	0,65649184	NA	4,36E-05	-0,00074998	NA	-0,15445798	-1,2176888	NA	NA	0,00020569	-0,00045432	NA	-0,0941422	10	-19049,5513	38119,1148	282,698054	1,34E-48
975	-0,2131598	NA	-0,0015413	4,25E-05	-0,00164139	NA	NA	-2,9468992	0,4586198	0,01897353	0,00021429	0,00012376	NA	NA	10	-19049,8698	38119,7518	283,335125	9,78E-49
430	-0,14195779	0,65649184	NA	4,36E-05	-0,00074998	NA	-0,15445798	-2,49206855	NA	0,01465098	0,00020366	NA	NA	NA	9	-19050,9324	38119,8748	283,458067	9,19E-49
2864	-0,45110821	0,66723944	0,00674107	4,29E-05	-0,00076432	NA	-0,15224001	-1,2176888	NA	NA	0,00020569	-0,00045432	NA	-0,0941422	11	-19048,9931	38120,0009	283,584167	8,63E-49
936	-1,05409025	0,78157333	0,00543311	4,44E-05	NA	NA	-0,11273577	-2,26386687	NA	0,01548996	0,00020413	-0,00046266	NA	NA	10	-19050,113	38120,2381	283,821426	7,67E-49
432	-0,45110821	0,66723944	0,00674107	4,29E-05	-0,00076432	NA	-0,15224001	-2,49206855	NA	0,01465098	0,00020366	NA	NA	NA	10	-19050,3743	38120,7607	284,343958	5,90E-49
2413	0,64576877	NA	NA	3,88E-05	-0,00183092	NA	-0,20438761	-1,66433243	0,4162362	NA	0,00021617	NA	NA	-0,08904476	9	-19051,9119	38121,8337	285,416973	3,45E-49
2446	-0,8455927	0,67951403	NA	4,64E-05	-0,00057321	NA	NA	-2,18775169	NA	0,01458917	0,00020109	NA	NA	-0,08533198	9	-19052,1629	38122,3358	285,919117	2,69E-49
2421	-0,84339357	NA	NA	4,07E-05	NA	0,55505772	-0,13640315	-1,66433243	0,4162362	NA	0,00021617	NA	NA	-0,08904476	9	-19052,1701	38122,3501	285,933405	2,67E-49
902	-1,235734	0,76857786	NA	4,69E-05	NA	NA	NA	-2,26386687	NA	0,01548996	0,00020413	-0,00046266	NA	NA	8	-19053,3719	38122,7518	286,335085	2,18E-49
2448	-1,17938783	0,69098467	0,00751019	4,56E-05	-0,00059207	NA	NA	-2,18775169	NA	0,01458917	0,00020109	NA	NA	-0,08533198	10	-19051,473	38122,9582	286,54143	1,97E-49
2423	-0,50593593	NA	-0,00700816	4,14E-05	NA	0,55021643	-0,14061875	-1,66433243	0,4162362	NA	0,00021617	NA	NA	-0,08904476	10	-19051,537	38123,0862	286,669496	1,85E-49
3005	0,65451489	NA	NA	3,95E-05	-0,00164357	0,48762971	-0,22170047	-1,91049389	NA	0,01549989	0,00020133	-0,00049532	NA	-0,09486783	11	-19050,5796	38123,1738	286,75711	1,77E-49
2925	0,64576877	NA	NA	3,88E-05	-0,00183092	NA	-0,20438761	-1,73983563	0,43090563	NA	0,00021643	0,00010896	NA	-0,08710954	10	-19051,6221	38123,2564	286,839727	1,69E-49
2470	-0,79586264	0,77122371	NA	4,50E-05	NA	NA	-0,11503973	-2,18775169	NA	0,01458917	0,00020109	NA	NA	-0,08533198	9	-19052,7367	38123,4833	287,066546	1,51E-49
2415	0,74969745	NA	-0,00245756	3,91E-05	-0,00181949	NA	-0,20513804	-1,66433243	0,4162362	NA	0,00021617	NA	NA	-0,08904476	10	-19051,8363	38123,6848	287,268035	1,37E-49
2933	-0,84339357	NA	NA	4,07E-05	NA	0,55505772	-0,13640315	-1,73983563	0,43090563	NA	0,00021643	0,00010896	NA	-0,08710954	10	-19051,8804	38123,7729	287,35616	1,31E-49
904	-1,52051301	0,78035551	0,00624724	4,63E-05	NA	NA	NA	-2,26386687	NA	0,01548996	0,00020413	-0,00046266	NA	NA	9	-19052,8958	38123,8016	287,384893	1,29E-49
2935	-0,50593593	NA	-0,00700816	4,14E-05	NA	0,55021643	-0,14061875	-1,73983563	0,43090563	NA	0,00021643	0,00010896	NA	-0,08710954	11	-19051,2473	38124,5092	288,092472	9,06E-50
2472	-1,05409025	0,78157333	0,00543311	4,44E-05	NA	NA	-0,11273577	-2,18775169	NA	0,01458917	0,00020109	NA	NA	-0,08533198	10	-19052,3764	38124,7649	288,348181	7,97E-50
2927	0,74969745	NA	-0,00245756	3,91E-05	-0,00181949	NA	-0,20513804	-1,73983563	0,43090563	NA	0,00021643	0,00010896	NA	-0,08710954	11	-19051,5466	38125,1077	288,691011	6,72E-50
3007	0,61699723	NA	0,00084798	3,94E-05	-0,00164748	0,4880593	-0,22142621	-1,91049389	NA	0,01549989	0,00020133	-0,00049532	NA	-0,09486783	12	-19050,5703	38125,1579	288,741177	6,55E-50
814	-0,14195779	0,65649184	NA	4,36E-05	-0,00074998	NA	-0,15445798	-1,56810442	NA	NA	0,00020849	-0,0004232	NA	NA	9	-19054,0943	38126,1985	289,781819	3,89E-51
816	-0,45110821	0,66723944	0,00674107	4,29E-05	-0,00076432	NA	-0,15224001	-1,56810442	NA	NA	0,00020849	-0,0004232	NA	NA	10	-19053,5361	38127,0844	290,667711	2,50E-50
2830	-0,8455927	0,67951403	NA	4,64E-05	-0,00057321	NA	NA	-1,2176888	NA	NA	0,00020569	-0,00045432	NA	-0,0941422	9	-19054,5718	38127,1535	290,736787	2,42E-50
2438	-1,235734	0,76857786	NA	4,69E-05	NA	NA	NA	-2,18775169	NA	0,01458917	0,00020109	NA	NA	-0,08533198	8	-19055,6353	38127,2786	290,86184	2,27E-50
2389	-1,36352414	NA	NA	4,29E-05	NA	0,54467715	NA	-1,66433243	0,4162362	NA	0,00021617	NA	NA	-0,08904476	8	-19055,7105	38127,4289	291,012174	2,10E-50
2832	-1,17938783	0,69098467	0,00751019	4,56E-05	-0,00059207	NA	NA	-1,2176888	NA	NA	0,00020569	-0,00045432	NA	-0,0941422	10	-19053,8818	38127,7758	291,3591	1,77E-50
365	0,64576877	NA	NA	3,88E-05	-0,00183092	NA	-0,20438761	-1,97841134	0,41538406	NA	0,00021885	NA	NA	NA	8	-19055,903	38127,814	291,397246	1,74E-50
398	-0,8455927	0,67951403	NA	4,64E-05	-0,00057321	NA	NA	-2,49206855	NA	0,01465098	0,00020366	NA	NA	NA	8	-19055,9529	38127,9137	291,497021	1,65E-50
2854	-0,79586264	0,77122371	NA	4,50E-05	NA	NA	-0,11503973	-1,2176888	NA	NA	0,00020569	-0,00045432	NA	-0,0941422	9	-19055,1455	38128,3009	291,884216	1,36E-50
2440	-1,52051301	0,78035551	0,00624724	4,63E-05	NA	NA	NA	-2,18775169	NA	0,01458917	0,00020109	NA	NA	-0,08533198	9	-19055,1592	38128,3284	291,911649	1,34E-50
373	-0,84339357	NA	NA	4,07E-05	NA	0,55505772	-0,13640315	-1,97841134	0,41538406	NA	0,00021885	NA	NA	NA	8	-19056,1612	38128,3304	291,913678	1,34E-50
400	-1,17938783	0,69098467	0,00751019	4,56E-05	-0,00059207	NA	NA	-2,49206855	NA	0,01465098	0,00020366	NA	NA	NA	9	-19055,2629	38128,5358	292,119113	1,21E-50
2391	-1,08960867	NA	-0,00576757	4,36E-05	NA	0,53973144	NA	-1,66433243	0,4162362	NA	0,00021617	NA	NA	-0,08904476	9	-19055,2782	38128,5663	292,149587	1,19E-50
2350	-0,14195779	0,65649184	NA	4,36E-05	-0,00074998	NA	-0,15445798	-1,50990804	0,4162362	NA	0,00020523	NA	NA	-0,08563427	9	-19055,3966	38128,8032	292,386457	1,06E-50
2901	-1,36352414	NA	NA	4,29E-05	NA	0,54467715	NA	-1,73983563	0,43090563	NA	0,00021643	0,00010896	NA	-0,08710954	9	-19055,4207	38128,8514	292,434707	1,03E-50
877	0,64576877	NA	NA	3,88E-05	-0,00183092	NA	-0,20438761	-2,0661368	0,43415925	NA	0,00021911	0,00013912	NA	NA	9	-19055,4251	38128,8601	292,443374	1,03E-50

422	-0,79586264	0,77122371	NA	4,50E-05	NA	NA	-0,11503973	-2,49206855	NA	0,01465098	0,00020366	NA	NA	NA	8	-19056,5266	38129,0612	292,644449	9,30E-51
375	-0,50593593	NA	-0,00700816	4,14E-05	NA	0,55021643	-0,14061875	-1,97841134	0,41538406	NA	0,00021885	NA	NA	NA	9	-19055,5282	38129,0663	292,649548	9,28E-51
885	-0,84339357	NA	NA	4,07E-05	NA	0,55505772	-0,13640315	-2,0661368	0,43415925	NA	0,00021911	0,00013912	NA	NA	9	-19055,6833	38129,3765	292,959806	7,95E-51
2856	-1,05409025	0,78157333	0,00543311	4,44E-05	NA	NA	-0,11273577	-1,2176888	NA	NA	0,00020569	-0,00045432	NA	-0,0941422	10	-19054,7852	38129,5826	293,165851	7,17E-52
367	0,74969745	NA	-0,00245756	3,91E-05	-0,00181949	NA	-0,20513804	-1,97841134	0,41538406	NA	0,00021885	NA	NA	NA	9	-19055,8274	38129,6648	293,248087	6,88E-51
2352	-0,45110821	0,66723944	0,00674107	4,29E-05	-0,00076432	NA	-0,15224001	-1,50990804	NA	NA	0,00020523	NA	NA	-0,08563427	10	-19054,8385	38129,6891	293,272349	6,80E-52
2903	-1,08960867	NA	-0,00576757	4,36E-05	NA	0,53973144	NA	-1,73983563	0,43090563	NA	0,00021643	0,00010896	NA	-0,08710954	10	-19054,9884	38129,9891	293,572341	5,85E-51
887	-0,50593593	NA	-0,00700816	4,14E-05	NA	0,55021643	-0,14061875	-2,0661368	0,43415925	NA	0,00021911	0,00013912	NA	NA	10	-19055,0502	38130,1126	293,695897	5,50E-51
424	-1,05409025	0,78157333	0,00543311	4,44E-05	NA	NA	-0,11273577	-2,49206855	NA	0,01465098	0,00020366	NA	NA	NA	9	-19056,1663	38130,3426	293,925863	4,90E-51
957	0,65451489	NA	NA	3,95E-05	-0,00164357	0,48762971	-0,22170047	-2,26386687	NA	0,01548996	0,00020413	-0,00046266	NA	NA	10	-19055,1772	38130,3665	293,949818	4,84E-51
879	0,74969745	NA	-0,00245756	3,91E-05	-0,00181949	NA	-0,20513804	-2,0661368	0,43415925	NA	0,00021911	0,00013912	NA	NA	10	-19055,3495	38130,7112	294,294436	4,08E-52
2822	-1,235734	0,76857786	NA	4,69E-05	NA	NA	NA	-1,2176888	NA	NA	0,00020569	-0,00045432	NA	-0,0941422	8	-19058,0441	38132,0962	295,67951	2,04E-51
959	0,61699723	NA	0,00084798	3,94E-05	-0,00164748	0,4880593	-0,22142621	-2,26386687	NA	0,01548996	0,00020413	-0,00046266	NA	NA	11	-19055,1679	38132,3504	295,933663	1,80E-51
390	-1,235734	0,76857786	NA	4,69E-05	NA	NA	NA	-2,49206855	NA	0,01465098	0,00020366	NA	NA	NA	7	-19059,4252	38132,8567	296,439965	1,39E-51
2824	-1,52051301	0,78035551	0,00624724	4,63E-05	NA	NA	NA	-1,2176888	NA	NA	0,00020569	-0,00045432	NA	-0,0941422	9	-19057,568	38133,146	296,729319	1,21E-51
341	-1,36352414	NA	NA	4,29E-05	NA	0,54467715	NA	-1,97841134	0,41538406	NA	0,00021885	NA	NA	NA	7	-19059,7016	38133,4094	296,992668	1,06E-51
392	-1,52051301	0,78035551	0,00624724	4,63E-05	NA	NA	NA	-2,49206855	NA	0,01465098	0,00020366	NA	NA	NA	8	-19058,9492	38133,9063	297,489553	8,25E-53
782	-0,8455927	0,67951403	NA	4,64E-05	-0,00057321	NA	NA	-1,56810442	NA	NA	0,00020849	-0,0004232	NA	NA	8	-19059,1148	38134,2375	297,820773	6,99E-52
302	-0,14195779	0,65649184	NA	4,36E-05	-0,00074998	NA	-0,15445798	-1,812439	NA	NA	0,00020783	NA	NA	NA	8	-19059,2211	38134,4502	298,033489	6,29E-52
853	-1,36352414	NA	NA	4,29E-05	NA	0,54467715	NA	-2,0661368	0,43415925	NA	0,00021911	0,00013912	NA	NA	8	-19059,2237	38134,4553	298,038575	6,27E-52
343	-1,08960867	NA	-0,00576757	4,36E-05	NA	0,53973144	NA	-1,97841134	0,41538406	NA	0,00021885	NA	NA	NA	8	-19059,2693	38134,5466	298,12986	5,99E-53
784	-1,17938783	0,69098467	0,00751019	4,56E-05	-0,00059207	NA	NA	-1,56810442	NA	NA	0,00020849	-0,0004232	NA	NA	9	-19058,4248	38134,8596	298,442865	5,12E-52
2493	0,65451489	NA	NA	3,95E-05	-0,00164357	0,48762971	-0,22170047	-2,18775169	NA	0,01458917	0,00020109	NA	NA	-0,08533198	10	-19057,4406	38134,8933	298,476574	5,04E-52
304	-0,45110821	0,66723944	0,00674107	4,29E-05	-0,00076432	NA	-0,15224001	-1,812439	NA	NA	0,00020783	NA	NA	NA	9	-19058,663	38135,3359	298,919159	4,04E-53
806	-0,79586264	0,77122371	NA	4,50E-05	NA	NA	-0,11503973	-1,56810442	NA	NA	0,00020849	-0,0004232	NA	NA	8	-19059,6885	38135,3849	298,968202	3,94E-52
4007	-0,65771633	NA	-0,01187559	4,14E-05	NA	NA	-0,10732692	-2,05206432	NA	0,01786019	0,00020271	-0,00039168	0,51529109	-0,14768431	11	-19056,7049	38135,4244	299,007713	3,86E-52
855	-1,08960867	NA	-0,00576757	4,36E-05	NA	0,53973144	NA	-2,0661368	0,43415925	NA	0,00021911	0,00013912	NA	NA	9	-19058,7914	38135,5927	299,175988	3,55E-53
2381	-0,28011525	NA	NA	4,24E-05	-0,0016491	NA	NA	-1,66433243	0,4162362	NA	0,00021617	NA	NA	-0,08904476	8	-19060,2022	38136,4123	299,995593	2,36E-52
808	-1,05409025	0,78157333	0,00543311	4,44E-05	NA	NA	-0,11273577	-1,56810442	NA	NA	0,00020849	-0,0004232	NA	NA	9	-19059,3282	38136,6663	300,249616	2,08E-52
2318	-0,8455927	0,67951403	NA	4,64E-05	-0,00057321	NA	NA	-1,50990804	NA	NA	0,00020523	NA	NA	-0,08563427	8	-19060,4171	38136,8421	300,425412	1,90E-52
2495	0,61699723	NA	0,00084798	3,94E-05	-0,00164748	0,4880593	-0,22142621	-2,18775169	NA	0,01458917	0,00020109	NA	NA	-0,08533198	11	-19057,4313	38136,8771	300,460419	1,87E-52
4005	-1,22822825	NA	NA	4,02E-05	NA	NA	-0,09995451	-2,05206432	NA	0,01786019	0,00020271	-0,00039168	0,51529109	-0,14768431	10	-19058,4494	38136,9109	300,494173	1,84E-52
2320	-1,17938783	0,69098467	0,00751019	4,56E-05	-0,00059207	NA	NA	-1,50990804	NA	NA	0,00020523	NA	NA	-0,08563427	9	-19059,7271	38137,4642	301,047503	1,39E-53
3975	-1,14142692	NA	-0,01089562	4,31E-05	NA	NA	NA	-2,05206432	NA	0,01786019	0,00020271	-0,00039168	0,51529109	-0,14768431	10	-19058,819	38137,6502	301,233481	1,27E-52
2893	-0,28011525	NA	NA	4,24E-05	-0,0016491	NA	NA	-1,73983563	0,43090563	NA	0,00021643	0,00010896	NA	-0,08710954	9	-19059,9124	38137,8348	301,418126	1,16E-52
2342	-0,79586264	0,77122371	NA	4,50E-05	NA	NA	-0,11503973	-1,50990804	NA	NA	0,00020523	NA	NA	-0,08563427	8	-19060,9908	38137,9896	301,57284	1,07E-52
2383	-0,2131598	NA	-0,0015413	4,25E-05	-0,00164139	NA	NA	-1,66433243	0,4162362	NA	0,00021617	NA	NA	-0,08904476	9	-19060,1725	38138,3549	301,938196	8,92E-53
3973	-1,63084607	NA	NA	4,18E-05	NA	NA	NA	-2,05206432	NA	0,01786019	0,00020271	-0,00039168	0,51529109	-0,14768431	9	-19060,2942	38138,5984	302,181629	7,90E-53
774	-1,235734	0,76857786	NA	4,69E-05	NA	NA	NA	-1,56810442	NA	NA	0,00020849	-0,0004232	NA	NA	7	-19062,5871	38139,1804	302,763718	5,91E-54
2344	-1,05409025	0,78157333	0,00543311	4,44E-05	NA	NA	-0,11273577	-1,50990804	NA	NA	0,00020523	NA	NA	-0,08563427	9	-19060,6305	38139,271	302,854254	5,64E-53
2877	0,65451489	NA	NA	3,95E-05	-0,00164357	0,48762971	-0,22170047	-1,2176888	NA	NA	0,00020569	-0,00045432	NA	-0,0941422	10	-19059,8494	38139,711	303,294244	4,53E-53
2895	-0,2131598	NA	-0,0015413	4,25E-05	-0,00164139	NA	NA	-1,73983563	0,43090563	NA	0,00021643	0,00010896	NA	-0,08710954	10	-19059,8828	38139,7777	303,360951	4,38E-53
776	-1,52051301	0,78035551	0,00624724	4,63E-05	NA	NA	NA	-1,56810442	NA	NA	0,00020849	-0,0004232	NA	NA	8	-19062,111	38140,23	303,813305	3,49E-53
445	0,65451489	NA	NA	3,95E-05	-0,00164357	0,48762971	-0,22170047	-2,49206855	NA	0,01465098	0,00020366	NA	NA	NA	9	-19061,2305	38140,471	304,054256	3,10E-53
2973	-0,31227401	NA	NA	4,32E-05	-0,00145314	0,47935513	NA	-1,91049389	NA	0,01549989	0,00020133	-0,00049532	NA	-0,09486783	10	-19060,4825	38140,9771	304,560369	2,41E-54
2879	0,61699723	NA	0,00084798	3,94E-05	-0,00164748	0,4880593	-0,22142621	-1,2176888	NA	NA	0,00020569	-0,00045432	NA	-0,0941422	11	-19059,8401	38141,6948	305,278089	1,68E-53
2310	-1,235734	0,76857786	NA	4,69E-05	NA	NA	NA	-1,50990804	NA	NA	0,00020523	NA	NA	-0,08563427	7	-19063,8894	38141,7851	305,368356	1,61E-53
3495	-0,65771633	NA	-0,01187559	4,14E-05	NA	NA	-0,10732692	-2,27275384	NA	0,01711404	0,00020255	NA	0,52288611	-0,14000571	10	-19061,0456	38142,1034	305,686716	1,37E-53
333	-0,28011525	NA	NA	4,24E-05	-0,0016491	NA	NA	-1,97841134	0,41538406	NA	0,00021885	NA	NA	NA	7	-19064,1933	38142,3928	305,976088	1,19E-53
447	0,61699723	NA	0,00084798	3,94E-05	-0,00164748	0,4880593	-0,22142621	-2,49206855	NA	0,01465098	0,00020366	NA	NA	NA	10	-19061,2212	38142,4546	306,03788	1,15E-53
270	-0,8455927	0,67951403	NA	4,64E-05	-0,00057321	NA	NA	-1,812439	NA	NA	0,00020783	NA	NA	NA	7	-19064,2416	38142,4894	306,072665	1,13E-54
2312	-1,52051301	0,78035551	0,00624724	4,63E-05	NA	NA	NA	-1,50990804	NA	NA	0,00020523	NA	NA	-0,08563427	8	-19063,4134	38142,8347	306,417943	9,50E-54
2975	-0,39160679	NA	0,00183718	4,30E-05	-0,00146197	0,48040455	NA	-1,91049389	NA	0,01549989	0,00020133	-0,00049532	NA	-0,09486783	11	-19060,4391	38142,8927	306,475981	9,23E-54
272	-1,17938783	0,69098467	0,00751019	4,56E-05	-0,00059207	NA	NA	-1,812439	NA	NA	0,00020783	NA	NA	NA	8	-19063,5516	38143,1113	306,694535	8,27E-54
845	-0,28011525	NA	NA	4,24E-05	-0,0016491	NA	NA	-2,0661368	0,43415925	NA	0,00021911	0,00013912	NA	NA	8	-19063,7154	38143,4387	307,021994	7,02E-55
3493	-1,22822825	NA	NA	4,02E-05	NA	NA	-0,09995451	-2,27275384	NA	0,01711404	0,00020255	NA	0,52288611	-0,14000571	9	-19062,7901	38143,5901	307,173398	6,51E-54
294	-0,79586264	0,77122371	NA	4,50E-05	NA	NA	-0,11503973	-1,812439	NA	NA	0,00020783	NA	NA	NA	7	-19064,8153	38143,6368	307,220093	6,36E-54
3463	-1,14142692	NA	-0,01089562	4,31E-05	NA	NA	NA	-2,27275384	NA	0,01711404	0,00020255	NA	0,52288611	-0,14000571	9	-19063,1597	38144,3294	307,912706	4,50E-54
335	-0,2131598	NA	-0,0015413	4,25E-05	-0,00164139	NA	NA	-1,97841134	0,41538406	NA	0,00021885	NA	NA	NA	8	-19064,1636	38144,3352	307,918469	4,49E-54
296	-1,05409025	0,78157333	0,00543311	4,44E-05	NA	NA	-0,11273577	-1,812439	NA	NA	0,00020783	NA	NA	NA	8	-19064,455	38144,918	308,501286	3,35E-54
3461	-1,63084607	NA	NA	4,18E-05	NA	NA	NA	-2,27275384	NA	0,01711404	0,00020255	NA	0,52288611	-0,14000571	8	-19064,6349	38145,2778	308,861076	2,80E-54

847	-0,2131598	NA	-0,0015413	4,25E-05	-0,00164139	NA	NA	-2,0661368	0,43415925	NA	0,00021911	0,00013912	NA	NA	9	-19063,6857	38145,3813	308,964597	2,66E-54
829	0,65451489	NA	NA	3,95E-05	-0,00164357	0,48762971	-0,22170047	-1,56810442	NA	NA	0,00020849	-0,0004232	NA	NA	9	-19064,3924	38146,7947	310,378008	1,31E-54
262	-1,235734	0,76857786	NA	4,69E-05	NA	NA	NA	-1,812439	NA	NA	0,00020783	NA	NA	NA	6	-19067,714	38147,4326	311,015831	9,54E-55
925	-0,31227401	NA	NA	4,32E-05	-0,00145314	0,47935513	NA	-2,26386687	NA	0,01548996	0,00020413	-0,00046266	NA	NA	9	-19065,08	38148,17	311,753299	6,60E-57
264	-1,52051301	0,78035551	0,00624724	4,63E-05	NA	NA	NA	-1,812439	NA	NA	0,00020783	NA	NA	NA	7	-19067,2379	38148,4819	312,065196	5,64E-55
831	0,61699723	NA	0,00084798	3,94E-05	-0,00164748	0,4880593	-0,22142621	-1,56810442	NA	NA	0,00020849	-0,0004232	NA	NA	10	-19064,3831	38148,7784	312,361632	4,87E-55
2365	0,65451489	NA	NA	3,95E-05	-0,00164357	0,48762971	-0,22170047	-1,50990804	NA	NA	0,00020523	NA	NA	-0,08563427	9	-19065,6947	38149,3994	312,982647	3,57E-55
927	-0,39160679	NA	0,00183718	4,30E-05	-0,00146197	0,48040455	NA	-2,26386687	NA	0,01548996	0,00020413	-0,00046266	NA	NA	10	-19065,0366	38150,0854	313,66869	2,53E-55
2367	0,61699723	NA	0,00084798	3,94E-05	-0,00164748	0,4880593	-0,22142621	-1,50990804	NA	NA	0,00020523	NA	NA	-0,08563427	10	-19065,6854	38151,383	314,96627	1,32E-56
2461	-0,31227401	NA	NA	4,32E-05	-0,00145314	0,47935513	NA	-2,18775169	NA	0,01458917	0,00020109	NA	NA	-0,08533198	9	-19067,3434	38152,6968	316,280054	6,86E-56
2463	-0,39160679	NA	0,00183718	4,30E-05	-0,00146197	0,48040455	NA	-2,18775169	NA	0,01458917	0,00020109	NA	NA	-0,08533198	10	-19067,3	38154,6122	318,195445	2,63E-57
317	0,65451489	NA	NA	3,95E-05	-0,00164357	0,48762971	-0,22170047	-1,812439	NA	NA	0,00020783	NA	NA	NA	8	-19069,5192	38155,0464	318,629678	2,12E-56
1959	-0,65771633	NA	-0,01187559	4,14E-05	NA	NA	-0,10732692	-2,58652225	NA	0,01772679	0,00020696	-0,00034396	0,48679163	NA	10	-19067,6507	38155,3135	318,896819	1,85E-56
1957	-1,22822825	NA	NA	4,02E-05	NA	NA	-0,09995451	-2,58652225	NA	0,01772679	0,00020696	-0,00034396	0,48679163	NA	9	-19069,3951	38156,8002	320,383501	8,81E-57
319	0,61699723	NA	0,00084798	3,94E-05	-0,00164748	0,4880593	-0,22142621	-1,812439	NA	NA	0,00020783	NA	NA	NA	9	-19069,5099	38157,0298	320,61308	7,86E-57
2845	-0,31227401	NA	NA	4,32E-05	-0,00145314	0,47935513	NA	-1,2176888	NA	NA	0,00020569	-0,00045432	NA	-0,0941422	9	-19069,7522	38157,5144	321,097724	6,17E-57
1927	-1,14142692	NA	-0,01089562	4,31E-05	NA	NA	NA	-2,58652225	NA	0,01772679	0,00020696	-0,00034396	0,48679163	NA	9	-19069,7648	38157,5395	321,122809	6,09E-57
3879	-0,65771633	NA	-0,01187559	4,14E-05	NA	NA	-0,10732692	-1,25486087	NA	NA	0,00020772	-0,0003441	0,50313521	-0,14558566	10	-19069,0077	38158,0275	321,610834	4,77E-57
413	-0,31227401	NA	NA	4,32E-05	-0,00145314	0,47935513	NA	-2,49206855	NA	0,01465098	0,00020366	NA	NA	NA	8	-19071,1334	38158,2747	321,857958	4,22E-57
1925	-1,63084607	NA	NA	4,18E-05	NA	NA	NA	-2,58652225	NA	0,01772679	0,00020696	-0,00034396	0,48679163	NA	8	-19071,24	38158,4879	322,071179	3,79E-57
2847	-0,39160679	NA	0,00183718	4,30E-05	-0,00146197	0,48040455	NA	-1,2176888	NA	NA	0,00020569	-0,00045432	NA	-0,0941422	10	-19069,7088	38159,4298	323,013115	2,37E-57
3877	-1,22822825	NA	NA	4,02E-05	NA	NA	-0,09995451	-1,25486087	NA	NA	0,00020772	-0,0003441	0,50313521	-0,14558566	9	-19070,7521	38159,5142	323,097516	2,27E-57
1447	-0,65771633	NA	-0,01187559	4,14E-05	NA	NA	-0,10732692	-2,75826159	NA	0,01709531	0,00020664	NA	0,4949792	NA	9	-19071,0391	38160,0882	323,671521	1,70E-57
415	-0,39160679	NA	0,00183718	4,30E-05	-0,00146197	0,48040455	NA	-2,49206855	NA	0,01465098	0,00020366	NA	NA	NA	9	-19071,0899	38160,1898	323,773127	1,62E-57
3847	-1,14142692	NA	-0,01089562	4,31E-05	NA	NA	NA	-1,25486087	NA	NA	0,00020772	-0,0003441	0,50313521	-0,14558566	9	-19071,1218	38160,2535	323,836824	1,57E-57
3845	-1,63084607	NA	NA	4,18E-05	NA	NA	NA	-1,25486087	NA	NA	0,00020772	-0,0003441	0,50313521	-0,14558566	8	-19072,597	38161,2019	324,785194	9,76E-58
1445	-1,22822825	NA	NA	4,02E-05	NA	NA	-0,09995451	-2,75826159	NA	0,01709531	0,00020664	NA	0,4949792	NA	8	-19072,7836	38161,5751	325,158424	8,10E-58
1415	-1,14142692	NA	-0,01089562	4,31E-05	NA	NA	NA	-2,75826159	NA	0,01709531	0,00020664	NA	0,4949792	NA	8	-19073,1532	38162,3145	325,897732	5,60E-58
3367	-0,65771633	NA	-0,01187559	4,14E-05	NA	NA	-0,10732692	-1,4783871	NA	NA	0,00020739	NA	0,51055393	-0,13912561	9	-19072,4016	38162,8132	326,39647	4,36E-58
1413	-1,63084607	NA	NA	4,18E-05	NA	NA	NA	-2,75826159	NA	0,01709531	0,00020664	NA	0,4949792	NA	7	-19074,6284	38163,263	326,846323	3,48E-58
3365	-1,22822825	NA	NA	4,02E-05	NA	NA	-0,09995451	-1,4783871	NA	NA	0,00020739	NA	0,51055393	-0,13912561	8	-19074,1461	38164,3001	327,883374	2,07E-59
797	-0,31227401	NA	NA	4,32E-05	-0,00145314	0,47935513	NA	-1,56810442	NA	NA	0,00020849	-0,0004232	NA	NA	8	-19074,2952	38164,5984	328,181711	1,79E-58
3335	-1,14142692	NA	-0,01089562	4,31E-05	NA	NA	NA	-1,4783871	NA	NA	0,00020739	NA	0,51055393	-0,13912561	8	-19074,5157	38165,0394	328,622682	1,43E-58
3333	-1,63084607	NA	NA	4,18E-05	NA	NA	NA	-1,4783871	NA	NA	0,00020739	NA	0,51055393	-0,13912561	7	-19075,9909	38165,988	329,571273	8,92E-59
799	-0,39160679	NA	0,00183718	4,30E-05	-0,00146197	0,48040455	NA	-1,56810442	NA	NA	0,00020849	-0,0004232	NA	NA	9	-19074,2518	38166,5136	330,09688	6,85E-59
2333	-0,31227401	NA	NA	4,32E-05	-0,00145314	0,47935513	NA	-1,50990804	NA	NA	0,00020523	NA	NA	-0,08563427	8	-19075,5976	38167,2031	330,786349	4,86E-59
2335	-0,39160679	NA	0,00183718	4,30E-05	-0,00146197	0,48040455	NA	-1,50990804	NA	NA	0,00020523	NA	NA	-0,08563427	9	-19075,5541	38169,1182	332,701518	1,86E-59
285	-0,31227401	NA	NA	4,32E-05	-0,00145314	0,47935513	NA	-1,812439	NA	NA	0,00020783	NA	NA	NA	7	-19079,4221	38172,8503	336,433602	2,88E-60
287	-0,39160679	NA	0,00183718	4,30E-05	-0,00146197	0,48040455	NA	-1,812439	NA	NA	0,00020783	NA	NA	NA	8	-19079,3787	38174,7653	338,348549	1,11E-60
1831	-0,65771633	NA	-0,01187559	4,14E-05	NA	NA	-0,10732692	-1,78635429	NA	NA	0,0002119	-0,00029974	0,47515083	NA	9	-19079,6895	38177,3889	340,972178	2,98E-61
1829	-1,22822825	NA	NA	4,02E-05	NA	NA	-0,09995451	-1,78635429	NA	NA	0,0002119	-0,00029974	0,47515083	NA	8	-19081,4339	38178,8758	342,459082	1,42E-61
1799	-1,14142692	NA	-0,01089562	4,31E-05	NA	NA	NA	-1,78635429	NA	NA	0,0002119	-0,00029974	0,47515083	NA	8	-19081,8036	38179,6151	343,19839	9,80E-62
1797	-1,63084607	NA	NA	4,18E-05	NA	NA	NA	-1,78635429	NA	NA	0,0002119	-0,00029974	0,47515083	NA	7	-19083,2788	38180,5637	344,146981	6,10E-62
1319	-0,65771633	NA	-0,01187559	4,14E-05	NA	NA	-0,10732692	-1,96172815	NA	NA	0,00021146	NA	0,48287384	NA	8	-19082,2944	38180,5967	344,179973	6,00E-62
1317	-1,22822825	NA	NA	4,02E-05	NA	NA	-0,09995451	-1,96172815	NA	NA	0,00021146	NA	0,48287384	NA	7	-19084,0388	38182,0838	345,667098	2,85E-62
1287	-1,14142692	NA	-0,01089562	4,31E-05	NA	NA	NA	-1,96172815	NA	NA	0,00021146	NA	0,48287384	NA	7	-19084,4085	38182,8231	346,406406	1,97E-62
2535	-0,65771633	NA	-0,01187559	4,14E-05	NA	NA	-0,10732692	-2,55577604	0,44288659	0,01897716	0,00021141	NA	NA	-0,08883356	10	-19081,571	38183,1542	346,737509	1,67E-62
1285	-1,63084607	NA	NA	4,18E-05	NA	NA	NA	-1,96172815	NA	NA	0,00021146	NA	0,48287384	NA	6	-19085,8836	38183,7719	347,355219	1,23E-62
2533	-1,22822825	NA	NA	4,02E-05	NA	NA	-0,09995451	-2,55577604	0,44288659	0,01897716	0,00021141	NA	NA	-0,08883356	9	-19083,3155	38184,6409	348,224191	7,94E-63
3047	-0,65771633	NA	-0,01187559	4,14E-05	NA	NA	-0,10732692	-2,61719209	0,45517288	0,01892094	0,00021164	9,21E-05	NA	-0,08713002	11	-19081,3655	38184,7456	348,328873	7,53E-63
2503	-1,14142692	NA	-0,01089562	4,31E-05	NA	NA	NA	-2,55577604	0,44288659	0,01897716	0,00021141	NA	NA	-0,08883356	9	-19083,6851	38185,3802	348,963499	5,48E-63
3045	-1,22822825	NA	NA	4,02E-05	NA	NA	-0,09995451	-2,61719209	0,45517288	0,01892094	0,00021164	9,21E-05	NA	-0,08713002	10	-19083,1099	38186,2321	349,815334	3,58E-63
2501	-1,63084607	NA	NA	4,18E-05	NA	NA	NA	-2,55577604	0,44288659	0,01897716	0,00021141	NA	NA	-0,08883356	8	-19085,1603	38186,3286	349,911868	3,41E-63
3015	-1,14142692	NA	-0,01089562	4,31E-05	NA	NA	NA	-2,61719209	0,45517288	0,01892094	0,00021164	9,21E-05	NA	-0,08713002	10	-19083,4796	38186,9714	350,554642	2,48E-63
3013	-1,63084607	NA	NA	4,18E-05	NA	NA	NA	-2,61719209	0,45517288	0,01892094	0,00021164	9,21E-05	NA	-0,08713002	9	-19084,9548	38187,9195	351,50279	1,54E-63
487	-0,65771633	NA	-0,01187559	4,14E-05	NA	NA	-0,10732692	-2,87228819	0,44207022	0,01904584	0,00021405	NA	NA	NA	9	-19085,5253	38189,0605	352,643809	8,71E-64
999	-0,65771633	NA	-0,01187559	4,14E-05	NA	NA	-0,10732692	-2,9468992	0,4586198	0,01897353	0,00021429	0,00012376	NA	NA	10	-19085,1499	38190,312	353,895264	4,66E-64
485	-1,22822825	NA	NA	4,02E-05	NA	NA	-0,09995451	-2,87228819	0,44207022	0,01904584	0,00021405	NA	NA	NA	8	-19087,2697	38190,5474	354,130713	4,14E-64
455	-1,14142692	NA	-0,01089562	4,31E-05	NA	NA	NA	-2,87228819	0,44207022	0,01904584	0,00021405	NA	NA	NA	8	-19087,6394	38191,2867	354,870021	2,86E-64
997	-1,22822825	NA	NA	4,02E-05	NA	NA	-0,09995451	-2,9468992	0,4586198	0,01897353	0,00021429	0,00012376	NA	NA	9	-19086,8944	38191,7987	355,381946	2,22E-64
453	-1,63084607	NA	NA	4,18E-05	NA	NA	NA	-2,87228819	0,44207022	0,01904584	0,00021405	NA	NA	NA	7	-19089,1146	38192,2353	355,818612	1,78E-64

2989	0,64576877	NA	NA	3,88E-05	-0,00183092	NA	-0,20438761	-1,91049389	NA	0,01549989	0,00020133	-0,00049532	NA	-0,09486783	10	-19086,1844	38192,381	355,964317	1,66E-64
967	-1,14142692	NA	-0,01089562	4,31E-05	NA	NA	NA	-2,9468992	0,4586198	0,01897353	0,00021429	0,00012376	NA	NA	9	-19087,264	38192,538	356,121254	1,53E-64
2997	-0,84339357	NA	NA	4,07E-05	NA	0,55505772	-0,13640315	-1,91049389	NA	0,01549989	0,00020133	-0,00049532	NA	-0,09486783	10	-19086,4427	38192,8975	356,48075	1,28E-64
965	-1,63084607	NA	NA	4,18E-05	NA	NA	NA	-2,9468992	0,4586198	0,01897353	0,00021429	0,00012376	NA	NA	8	-19088,7392	38193,4863	357,069623	9,53E-65
2999	-0,50593593	NA	-0,00700816	4,14E-05	NA	0,55021643	-0,14061875	-1,91049389	NA	0,01549989	0,00020133	-0,00049532	NA	-0,09486783	11	-19085,8096	38193,6338	357,217062	8,85E-66
2991	0,74969745	NA	-0,00245756	3,91E-05	-0,00181949	NA	-0,20513804	-1,91049389	NA	0,01549989	0,00020133	-0,00049532	NA	-0,09486783	11	-19086,1089	38194,2323	357,815602	6,56E-65
2965	-1,36352414	NA	NA	4,29E-05	NA	0,54467715	NA	-1,91049389	NA	0,01549989	0,00020133	-0,00049532	NA	-0,09486783	9	-19089,983	38197,976	361,559297	1,01E-65
2967	-1,08960867	NA	-0,00576757	4,36E-05	NA	0,53973144	NA	-1,91049389	NA	0,01549989	0,00020133	-0,00049532	NA	-0,09486783	10	-19089,5507	38199,1137	362,696931	5,71E-66
941	0,64576877	NA	NA	3,88E-05	-0,00183092	NA	-0,20438761	-2,26386687	NA	0,01548996	0,00020413	-0,00046266	NA	NA	9	-19090,782	38199,574	363,157247	4,54E-66
949	-0,84339357	NA	NA	4,07E-05	NA	0,55505772	-0,13640315	-2,26386687	NA	0,01548996	0,00020413	-0,00046266	NA	NA	9	-19091,0402	38200,0904	363,67368	3,51E-66
951	-0,50593593	NA	-0,00700816	4,14E-05	NA	0,55021643	-0,14061875	-2,26386687	NA	0,01548996	0,00020413	-0,00046266	NA	NA	10	-19090,4072	38200,8265	364,40977	2,43E-66
943	0,74969745	NA	-0,00245756	3,91E-05	-0,00181949	NA	-0,20513804	-2,26386687	NA	0,01548996	0,00020413	-0,00046266	NA	NA	10	-19090,7064	38201,425	365,00831	1,80E-66
2477	0,64576877	NA	NA	3,88E-05	-0,00183092	NA	-0,20438761	-2,18775169	NA	0,01458917	0,00020109	NA	NA	-0,08533198	9	-19093,0454	38204,1007	367,684002	4,72E-67
2485	-0,84339357	NA	NA	4,07E-05	NA	0,55505772	-0,13640315	-2,18775169	NA	0,01458917	0,00020109	NA	NA	-0,08533198	9	-19093,3036	38204,6172	368,200435	3,65E-67
917	-1,36352414	NA	NA	4,29E-05	NA	0,54467715	NA	-2,26386687	NA	0,01548996	0,00020413	-0,00046266	NA	NA	8	-19094,5806	38205,1692	368,752448	2,77E-67
2487	-0,50593593	NA	-0,00700816	4,14E-05	NA	0,55021643	-0,14061875	-2,18775169	NA	0,01458917	0,00020109	NA	NA	-0,08533198	10	-19092,6705	38205,3532	368,936526	2,52E-67
2479	0,74969745	NA	-0,00245756	3,91E-05	-0,00181949	NA	-0,20513804	-2,18775169	NA	0,01458917	0,00020109	NA	NA	-0,08533198	10	-19092,9698	38205,9518	369,535065	1,87E-67
919	-1,08960867	NA	-0,00576757	4,36E-05	NA	0,53973144	NA	-2,26386687	NA	0,01548996	0,00020413	-0,00046266	NA	NA	9	-19094,1483	38206,3066	369,889861	1,57E-67
2957	-0,28011525	NA	NA	4,24E-05	-0,0016491	NA	NA	-1,91049389	NA	0,01549989	0,00020133	-0,00049532	NA	-0,09486783	9	-19094,4747	38206,9594	370,542716	1,13E-67
2959	-0,2131598	NA	-0,0015413	4,25E-05	-0,00164139	NA	NA	-1,91049389	NA	0,01549989	0,00020133	-0,00049532	NA	-0,09486783	10	-19094,445	38208,9023	372,485541	4,28E-68
2407	-0,65771633	NA	-0,01187559	4,14E-05	NA	NA	-0,10732692	-1,66433243	0,4162362	NA	0,00021617	NA	NA	-0,08904476	9	-19095,4526	38208,9151	372,498335	4,25E-68
2861	0,64576877	NA	NA	3,88E-05	-0,00183092	NA	-0,20438761	-1,2176888	NA	NA	0,00020569	-0,00045432	NA	-0,0941422	9	-19095,4542	38208,9184	372,501672	4,25E-68
2869	-0,84339357	NA	NA	4,07E-05	NA	0,55505772	-0,13640315	-1,2176888	NA	NA	0,00020569	-0,00045432	NA	-0,0941422	9	-19095,7124	38209,4348	373,018105	3,28E-68
429	0,64576877	NA	NA	3,88E-05	-0,00183092	NA	-0,20438761	-2,49206855	NA	0,01465098	0,00020366	NA	NA	NA	8	-19096,8353	38209,6786	373,261906	2,90E-68
2453	-1,36352414	NA	NA	4,29E-05	NA	0,54467715	NA	-2,18775169	NA	0,01458917	0,00020109	NA	NA	-0,08533198	8	-19096,844	38209,6959	373,279203	2,88E-68
2871	-0,50593593	NA	-0,00700816	4,14E-05	NA	0,55021643	-0,14061875	-1,2176888	NA	NA	0,00020569	-0,00045432	NA	-0,0941422	10	-19095,0794	38210,1709	373,754196	2,27E-68
437	-0,84339357	NA	NA	4,07E-05	NA	0,55505772	-0,13640315	-2,49206855	NA	0,01465098	0,00020366	NA	NA	NA	8	-19097,0935	38210,1951	373,778339	2,24E-68
2919	-0,65771633	NA	-0,01187559	4,14E-05	NA	NA	-0,10732692	-1,73983563	0,43090563	NA	0,00021643	0,00010896	NA	-0,08710954	10	-19095,1628	38210,3378	373,921089	2,09E-68
2405	-1,22822825	NA	NA	4,02E-05	NA	NA	-0,09995451	-1,66433243	0,4162362	NA	0,00021617	NA	NA	-0,08904476	8	-19097,197	38210,402	373,985238	2,02E-68
2863	0,74969745	NA	-0,00245756	3,91E-05	-0,00181949	NA	-0,20513804	-1,2176888	NA	NA	0,00020569	-0,00045432	NA	-0,0941422	10	-19095,3786	38210,7695	374,352735	1,68E-68
2455	-1,08960867	NA	-0,00576757	4,36E-05	NA	0,53973144	NA	-2,18775169	NA	0,01458917	0,00020109	NA	NA	-0,08533198	9	-19096,4117	38210,8333	374,416616	1,63E-68
439	-0,50593593	NA	-0,00700816	4,14E-05	NA	0,55021643	-0,14061875	-2,49206855	NA	0,01465098	0,00020366	NA	NA	NA	9	-19096,4605	38210,9309	374,514208	1,55E-68
2375	-1,14142692	NA	-0,01089562	4,31E-05	NA	NA	NA	-1,66433243	0,4162362	NA	0,00021617	NA	NA	-0,08904476	8	-19097,5667	38211,1413	374,724546	1,40E-68
431	0,74969745	NA	-0,00245756	3,91E-05	-0,00181949	NA	-0,20513804	-2,49206855	NA	0,01465098	0,00020366	NA	NA	NA	9	-19096,7598	38211,5295	375,112747	1,15E-68
2917	-1,22822825	NA	NA	4,02E-05	NA	NA	-0,09995451	-1,73983563	0,43090563	NA	0,00021643	0,00010896	NA	-0,08710954	9	-19096,9073	38211,8245	375,407771	9,93E-69
2373	-1,63084607	NA	NA	4,18E-05	NA	NA	NA	-1,66433243	0,4162362	NA	0,00021617	NA	NA	-0,08904476	7	-19099,0418	38212,0899	375,673137	8,69E-69
2887	-1,14142692	NA	-0,01089562	4,31E-05	NA	NA	NA	-1,73983563	0,43090563	NA	0,00021643	0,00010896	NA	-0,08710954	9	-19097,2769	38212,5638	376,147079	6,86E-69
2885	-1,63084607	NA	NA	4,18E-05	NA	NA	NA	-1,73983563	0,43090563	NA	0,00021643	0,00010896	NA	-0,08710954	8	-19098,7521	38213,5122	377,095449	4,27E-69
909	-0,28011525	NA	NA	4,24E-05	-0,0016491	NA	NA	-2,26386687	NA	0,01548996	0,00020413	-0,00046266	NA	NA	8	-19099,0723	38214,1526	377,735867	3,10E-69
2837	-1,36352414	NA	NA	4,29E-05	NA	0,54467715	NA	-1,2176888	NA	NA	0,00020569	-0,00045432	NA	-0,0941422	8	-19099,2528	38214,5136	378,096873	2,59E-69
359	-0,65771633	NA	-0,01187559	4,14E-05	NA	NA	-0,10732692	-1,97841134	0,41538406	NA	0,00021885	NA	NA	NA	8	-19099,4437	38214,8953	378,478608	2,14E-69
405	-1,36352414	NA	NA	4,29E-05	NA	0,54467715	NA	-2,49206855	NA	0,01465098	0,00020366	NA	NA	NA	7	-19100,6339	38215,2741	378,857329	1,77E-69
2839	-1,08960867	NA	-0,00576757	4,36E-05	NA	0,53973144	NA	-1,2176888	NA	NA	0,00020569	-0,00045432	NA	-0,0941422	9	-19098,8205	38215,651	379,234286	1,47E-70
871	-0,65771633	NA	-0,01187559	4,14E-05	NA	NA	-0,10732692	-2,0661368	0,43415925	NA	0,00021911	0,00013912	NA	NA	9	-19098,9658	38215,9415	379,524736	1,27E-70
813	0,64576877	NA	NA	3,88E-05	-0,00183092	NA	-0,20438761	-1,56810442	NA	NA	0,00020849	-0,0004232	NA	NA	8	-19099,9972	38216,0024	379,585659	1,23E-69
911	-0,2131598	NA	-0,0015413	4,25E-05	-0,00164139	NA	NA	-2,26386687	NA	0,01548996	0,00020413	-0,00046266	NA	NA	9	-19099,0426	38216,0952	379,678471	1,17E-69
357	-1,22822825	NA	NA	4,02E-05	NA	NA	-0,09995451	-1,97841134	0,41538406	NA	0,00021885	NA	NA	NA	7	-19101,1881	38216,3825	379,965733	1,02E-69
407	-1,08960867	NA	-0,00576757	4,36E-05	NA	0,53973144	NA	-2,49206855	NA	0,01465098	0,00020366	NA	NA	NA	8	-19100,2016	38216,4112	379,99452	1,00E-70
821	-0,84339357	NA	NA	4,07E-05	NA	0,55505772	-0,13640315	-1,56810442	NA	NA	0,00020849	-0,0004232	NA	NA	8	-19100,2554	38216,5188	380,102091	9,49E-71
327	-1,14142692	NA	-0,01089562	4,31E-05	NA	NA	NA	-1,97841134	0,41538406	NA	0,00021885	NA	NA	NA	7	-19101,5578	38217,1218	380,705041	7,02E-70
823	-0,50593593	NA	-0,00700816	4,14E-05	NA	0,55021643	-0,14061875	-1,56810442	NA	NA	0,00020849	-0,0004232	NA	NA	9	-19099,6224	38217,2547	380,837961	6,57E-70
869	-1,22822825	NA	NA	4,02E-05	NA	NA	-0,09995451	-2,0661368	0,43415925	NA	0,00021911	0,00013912	NA	NA	8	-19100,7102	38217,4284	381,011639	6,03E-70
815	0,74969745	NA	-0,00245756	3,91E-05	-0,00181949	NA	-0,20513804	-1,56810442	NA	NA	0,00020849	-0,0004232	NA	NA	9	-19099,9216	38217,8532	381,4365	4,87E-70
325	-1,63084607	NA	NA	4,18E-05	NA	NA	NA	-1,97841134	0,41538406	NA	0,00021885	NA	NA	NA	6	-19103,033	38218,0706	381,653854	4,37E-70
839	-1,14142692	NA	-0,01089562	4,31E-05	NA	NA	NA	-2,0661368	0,43415925	NA	0,00021911	0,00013912	NA	NA	8	-19101,0799	38218,1677	381,750947	4,16E-70
2349	0,64576877	NA	NA	3,88E-05	-0,00183092	NA	-0,20438761	-1,50990804	NA	NA	0,00020523	NA	NA	-0,08563427	8	-19101,2995	38218,607	382,190297	3,34E-70
2445	-0,28011525	NA	NA	4,24E-05	-0,0016491	NA	NA	-2,18775169	NA	0,01458917	0,00020109	NA	NA	-0,08533198	8	-19101,3357	38218,6793	382,262623	3,22E-70
837	-1,63084607	NA	NA	4,18E-05	NA	NA	NA	-2,0661368	0,43415925	NA	0,00021911	0,00013912	NA	NA	7	-19102,555	38219,1163	382,699538	2,59E-70
2357	-0,84339357	NA	NA	4,07E-05	NA	0,55505772	-0,13640315	-1,50990804	NA	NA	0,00020523	NA	NA	-0,08563427	8	-19101,5577	38219,1235	382,70673	2,58E-70
2359	-0,50593593	NA	-0,00700816	4,14E-05	NA	0,55021643	-0,14061875	-1,50990804	NA	NA	0,00020523	NA	NA	-0,08563427	9	-19100,9247	38219,8593	383,442599	1,79E-70
2351	0,74969745	NA	-0,00245756	3,91E-05	-0,00181949	NA	-0,20513804	-1,50990804	NA	NA	0,00020523	NA	NA	-0,08563427	9	-19101,224	38220,4579	384,041138	1,32E-70

2447	-0,2131598	NA	-0,0015413	4,25E-05	-0,00164139	NA	NA	-2,18775169	NA	0,01458917	0,00020109	NA	NA	-0,08533198	9	-19101,306	38220,6219	384,205226	1,22E-70
789	-1,36352414	NA	NA	4,29E-05	NA	0,54467715	NA	-1,56810442	NA	NA	0,00020849	-0,0004232	NA	NA	7	-19103,7958	38221,5978	385,181081	7,49E-71
791	-1,08960867	NA	-0,00576757	4,36E-05	NA	0,53973144	NA	-1,56810442	NA	NA	0,00020849	-0,0004232	NA	NA	8	-19103,3635	38222,735	386,318273	4,24E-71
2829	-0,28011525	NA	NA	4,24E-05	-0,0016491	NA	NA	-1,2176888	NA	NA	0,00020569	-0,00045432	NA	-0,0941422	8	-19103,7445	38223,497	387,080293	2,90E-72
2325	-1,36352414	NA	NA	4,29E-05	NA	0,54467715	NA	-1,50990804	NA	NA	0,00020523	NA	NA	-0,08563427	7	-19105,0981	38224,2024	387,78572	2,04E-71
301	0,64576877	NA	NA	3,88E-05	-0,00183092	NA	-0,20438761	-1,812439	NA	NA	0,00020783	NA	NA	NA	7	-19105,124	38224,2543	387,83755	1,98E-71
397	-0,28011525	NA	NA	4,24E-05	-0,0016491	NA	NA	-2,49206855	NA	0,01465098	0,00020366	NA	NA	NA	7	-19105,1256	38224,2575	387,840748	1,98E-71
309	-0,84339357	NA	NA	4,07E-05	NA	0,55505772	-0,13640315	-1,812439	NA	NA	0,00020783	NA	NA	NA	7	-19105,3823	38224,7707	388,353983	1,53E-71
2327	-1,08960867	NA	-0,00576757	4,36E-05	NA	0,53973144	NA	-1,50990804	NA	NA	0,00020523	NA	NA	-0,08563427	8	-19104,6658	38225,3396	388,922911	1,15E-71
2831	-0,2131598	NA	-0,0015413	4,25E-05	-0,00164139	NA	NA	-1,2176888	NA	NA	0,00020569	-0,00045432	NA	-0,0941422	9	-19103,7148	38225,4396	389,022896	1,10E-71
311	-0,50593593	NA	-0,00700816	4,14E-05	NA	0,55021643	-0,14061875	-1,812439	NA	NA	0,00020783	NA	NA	NA	8	-19104,7492	38225,5064	389,08963	1,06E-71
303	0,74969745	NA	-0,00245756	3,91E-05	-0,00181949	NA	-0,20513804	-1,812439	NA	NA	0,00020783	NA	NA	NA	8	-19105,0485	38226,1049	389,68817	7,87E-72
399	-0,2131598	NA	-0,0015413	4,25E-05	-0,00164139	NA	NA	-2,49206855	NA	0,01465098	0,00020366	NA	NA	NA	8	-19105,0959	38226,1999	389,78313	7,50E-72
277	-1,36352414	NA	NA	4,29E-05	NA	0,54467715	NA	-1,812439	NA	NA	0,00020783	NA	NA	NA	6	-19108,9226	38229,8499	393,433194	1,21E-72
781	-0,28011525	NA	NA	4,24E-05	-0,0016491	NA	NA	-1,56810442	NA	NA	0,00020849	-0,0004232	NA	NA	7	-19108,2875	38230,5812	394,164501	8,39E-73
279	-1,08960867	NA	-0,00576757	4,36E-05	NA	0,53973144	NA	-1,812439	NA	NA	0,00020783	NA	NA	NA	7	-19108,4903	38230,9869	394,570164	6,85E-73
783	-0,2131598	NA	-0,0015413	4,25E-05	-0,00164139	NA	NA	-1,56810442	NA	NA	0,00020849	-0,0004232	NA	NA	8	-19108,2578	38232,5236	396,106882	3,18E-73
2317	-0,28011525	NA	NA	4,24E-05	-0,0016491	NA	NA	-1,50990804	NA	NA	0,00020523	NA	NA	-0,08563427	7	-19109,5898	38233,1859	396,769139	2,28E-73
2319	-0,2131598	NA	-0,0015413	4,25E-05	-0,00164139	NA	NA	-1,50990804	NA	NA	0,00020523	NA	NA	-0,08563427	8	-19109,5601	38235,1282	398,711521	8,64E-74
269	-0,28011525	NA	NA	4,24E-05	-0,0016491	NA	NA	-1,812439	NA	NA	0,00020783	NA	NA	NA	6	-19113,4143	38238,8333	402,416613	1,36E-75
271	-0,2131598	NA	-0,0015413	4,25E-05	-0,00164139	NA	NA	-1,812439	NA	NA	0,00020783	NA	NA	NA	7	-19113,3847	38240,7755	404,358774	5,13E-76
2983	-0,65771633	NA	-0,01187559	4,14E-05	NA	NA	-0,10732692	-1,91049389	NA	0,01549989	0,00020133	-0,00049532	NA	-0,09486783	10	-19129,7251	38279,4624	443,04568	2,04E-83
3580	-5,95790631	0,3741151	0,04613303	NA	-0,00180249	0,39660544	-0,62074526	-2,59012234	0,38593256	0,02081194	0,00021148	NA	0,47514204	-0,138389	13	-19126,7631	38279,5463	443,129598	1,96E-83
4092	-5,95790631	0,3741151	0,04613303	NA	-0,00180249	0,39660544	-0,62074526	-2,66976045	0,40191971	0,02073197	0,0002118	0,00011864	0,47565984	-0,13600353	14	-19126,4211	38280,8654	444,448696	1,01E-83
2981	-1,22822825	NA	NA	4,02E-05	NA	NA	-0,09995451	-1,91049389	NA	0,01549989	0,00020133	-0,00049532	NA	-0,09486783	9	-19131,4696	38280,9491	444,532362	9,70E-84
2951	-1,14142692	NA	-0,01089562	4,31E-05	NA	NA	NA	-1,91049389	NA	0,01549989	0,00020133	-0,00049532	NA	-0,09486783	9	-19131,8392	38281,6884	445,27167	6,70E-84
2949	-1,63084607	NA	NA	4,18E-05	NA	NA	NA	-1,91049389	NA	0,01549989	0,00020133	-0,00049532	NA	-0,09486783	8	-19133,3144	38282,6368	446,220039	4,17E-84
935	-0,65771633	NA	-0,01187559	4,14E-05	NA	NA	-0,10732692	-2,26386687	NA	0,01548996	0,00020413	-0,00046266	NA	NA	9	-19134,3227	38286,6553	450,238609	5,59E-85
933	-1,22822825	NA	NA	4,02E-05	NA	NA	-0,09995451	-2,26386687	NA	0,01548996	0,00020413	-0,00046266	NA	NA	8	-19136,0671	38288,1422	451,725513	2,66E-85
903	-1,14142692	NA	-0,01089562	4,31E-05	NA	NA	NA	-2,26386687	NA	0,01548996	0,00020413	-0,00046266	NA	NA	8	-19136,4368	38288,8815	452,464821	1,84E-85
901	-1,63084607	NA	NA	4,18E-05	NA	NA	NA	-2,26386687	NA	0,01548996	0,00020413	-0,00046266	NA	NA	7	-19137,912	38289,8301	453,413412	1,14E-85
2471	-0,65771633	NA	-0,01187559	4,14E-05	NA	NA	-0,10732692	-2,18775169	NA	0,01458917	0,00020109	NA	NA	-0,08533198	9	-19136,5861	38291,1821	454,765365	5,82E-86
2469	-1,22822825	NA	NA	4,02E-05	NA	NA	-0,09995451	-2,18775169	NA	0,01458917	0,00020109	NA	NA	-0,08533198	8	-19138,3305	38292,669	456,252268	2,76E-86
2439	-1,14142692	NA	-0,01089562	4,31E-05	NA	NA	NA	-2,18775169	NA	0,01458917	0,00020109	NA	NA	-0,08533198	8	-19138,7002	38293,4083	456,991576	1,91E-86
2437	-1,63084607	NA	NA	4,18E-05	NA	NA	NA	-2,18775169	NA	0,01458917	0,00020109	NA	NA	-0,08533198	7	-19140,1753	38294,3569	457,940167	1,19E-86
2855	-0,65771633	NA	-0,01187559	4,14E-05	NA	NA	-0,10732692	-1,2176888	NA	NA	0,00020569	-0,00045432	NA	-0,0941422	9	-19138,9949	38295,9998	459,583035	5,23E-87
1532	-5,95790631	0,3741151	0,04613303	NA	-0,00180249	0,39660544	-0,62074526	-3,07214819	0,38813022	0,02082345	0,00021556	NA	0,44803127	NA	12	-19136,2171	38296,4514	460,034685	4,17E-87
423	-0,65771633	NA	-0,01187559	4,14E-05	NA	NA	-0,10732692	-2,49206855	NA	0,01465098	0,00020366	NA	NA	NA	8	-19140,376	38296,76	460,343268	3,58E-87
2044	-5,95790631	0,3741151	0,04613303	NA	-0,00180249	0,39660544	-0,62074526	-3,17508673	0,41103069	0,02071909	0,00021591	0,0001702	0,4495725	NA	13	-19135,5022	38297,0245	460,607807	3,13E-87
2853	-1,22822825	NA	NA	4,02E-05	NA	NA	-0,09995451	-1,2176888	NA	NA	0,00020569	-0,00045432	NA	-0,0941422	8	-19140,7393	38297,4867	461,069938	2,49E-87
2823	-1,14142692	NA	-0,01089562	4,31E-05	NA	NA	NA	-1,2176888	NA	NA	0,00020569	-0,00045432	NA	-0,0941422	8	-19141,109	38298,226	461,809246	1,72E-87
421	-1,22822825	NA	NA	4,02E-05	NA	NA	-0,09995451	-2,49206855	NA	0,01465098	0,00020366	NA	NA	NA	7	-19142,1205	38298,2471	461,830393	1,70E-87
391	-1,14142692	NA	-0,01089562	4,31E-05	NA	NA	NA	-2,49206855	NA	0,01465098	0,00020366	NA	NA	NA	7	-19142,4901	38298,9864	462,569701	1,17E-87
2821	-1,63084607	NA	NA	4,18E-05	NA	NA	NA	-1,2176888	NA	NA	0,00020569	-0,00045432	NA	-0,0941422	7	-19142,5842	38299,1746	462,757837	1,07E-87
389	-1,63084607	NA	NA	4,18E-05	NA	NA	NA	-2,49206855	NA	0,01465098	0,00020366	NA	NA	NA	6	-19143,9653	38299,9352	463,518514	7,31E-88
3579	-8,35316771	NA	0,03864408	NA	-0,00234345	0,43617682	-0,64441699	-2,59012234	0,38593256	0,02081194	0,00021148	NA	0,47514204	-0,138389	12	-19138,6687	38301,3548	464,938034	3,59E-89
4091	-8,35316771	NA	0,03864408	NA	-0,00234345	0,43617682	-0,64441699	-2,66976045	0,40191971	0,02073197	0,0002118	0,00011864	0,47565984	-0,13600353	13	-19138,3267	38302,6736	466,256909	1,86E-88
807	-0,65771633	NA	-0,01187559	4,14E-05	NA	NA	-0,10732692	-1,56810442	NA	NA	0,00020849	-0,0004232	NA	NA	8	-19143,5379	38303,0837	466,667021	1,51E-88
805	-1,22822825	NA	NA	4,02E-05	NA	NA	-0,09995451	-1,56810442	NA	NA	0,00020849	-0,0004232	NA	NA	7	-19145,2823	38304,5709	468,154146	7,20E-89
775	-1,14142692	NA	-0,01089562	4,31E-05	NA	NA	NA	-1,56810442	NA	NA	0,00020849	-0,0004232	NA	NA	7	-19145,652	38305,3102	468,893454	4,97E-89
2343	-0,65771633	NA	-0,01187559	4,14E-05	NA	NA	-0,10732692	-1,50990804	NA	NA	0,00020523	NA	NA	-0,08563427	8	-19144,8402	38305,6884	469,271659	4,12E-89
773	-1,63084607	NA	NA	4,18E-05	NA	NA	NA	-1,56810442	NA	NA	0,00020849	-0,0004232	NA	NA	6	-19147,1272	38306,259	469,842266	3,10E-89
2341	-1,22822825	NA	NA	4,02E-05	NA	NA	-0,09995451	-1,50990804	NA	NA	0,00020523	NA	NA	-0,08563427	7	-19146,5847	38307,1755	470,758784	1,96E-89
2311	-1,14142692	NA	-0,01089562	4,31E-05	NA	NA	NA	-1,50990804	NA	NA	0,00020523	NA	NA	-0,08563427	7	-19146,9543	38307,9148	471,498092	1,35E-89
2309	-1,63084607	NA	NA	4,18E-05	NA	NA	NA	-1,50990804	NA	NA	0,00020523	NA	NA	-0,08563427	6	-19148,4295	38308,8636	472,446905	8,42E-90
3452	-5,95790631	0,3741151	0,04613303	NA	-0,00180249	0,39660544	-0,62074526	-1,61349146	0,35721774	NA	0,00021669	NA	0,46315983	-0,13737061	12	-19143,4312	38310,8796	474,462892	3,07E-90
295	-0,65771633	NA	-0,01187559	4,14E-05	NA	NA	-0,10732692	-1,812439	NA	NA	0,00020783	NA	NA	NA	7	-19148,6647	38311,3356	474,918912	2,45E-90
3964	-5,95790631	0,3741151	0,04613303	NA	-0,00180249	0,39660544	-0,62074526	-1,71083893	0,37612338	NA	0,00021703	0,00013898	0,46389079	-0,13469622	13	-19142,9579	38311,936	475,519268	1,81E-90
293	-1,22822825	NA	NA	4,02E-05	NA	NA	-0,09995451	-1,812439	NA	NA	0,00020783	NA	NA	NA	6	-19150,4092	38312,823	476,406259	1,16E-90
263	-1,14142692	NA	-0,01089562	4,31E-05	NA	NA	NA	-1,812439	NA	NA	0,00020783	NA	NA	NA	6	-19150,7788	38313,5623	477,145567	8,03E-91
261	-1,63084607	NA	NA	4,18E-05	NA	NA	NA	-1,812439	NA	NA	0,00020783	NA	NA	NA	5	-19152,254	38314,5113	478,094601	5,00E-92

3564	-9,35928532	0,43489058	0,04364193	NA	-0,00182965	NA	-0,59565606	-2,59012234	0,38593256	0,02081194	0,00021148	NA	0,47514204	-0,138389	12	-19145,8664	38315,7501	479,333337	2,69E-91
4076	-9,35928532	0,43489058	0,04364193	NA	-0,00182965	NA	-0,59565606	-2,66976045	0,40191971	0,02073197	0,0002118	0,00011864	0,47565984	-0,13600353	13	-19145,5244	38317,0689	480,652213	1,39E-91
1531	-8,35316771	NA	0,03864408	NA	-0,00234345	0,43617682	-0,64441699	-3,07214819	0,38813022	0,02082345	0,00021556	NA	0,44803127	NA	11	-19148,1227	38318,2601	481,843341	7,67E-94
2043	-8,35316771	NA	0,03864408	NA	-0,00234345	0,43617682	-0,64441699	-3,17508673	0,41103069	0,02071909	0,00021591	0,0001702	0,4495725	NA	12	-19147,4079	38318,833	482,416243	5,76E-94
3578	-6,98837994	0,26427623	NA	NA	-0,00180413	0,37036432	-0,69230587	-2,59012234	0,38593256	0,02081194	0,00021148	NA	0,47514204	-0,138389	12	-19149,087	38322,1912	485,774519	1,07E-92
4090	-6,98837994	0,26427623	NA	NA	-0,00180413	0,37036432	-0,69230587	-2,66976045	0,40191971	0,02073197	0,0002118	0,00011864	0,47565984	-0,13600353	13	-19148,745	38323,5101	487,093394	5,55E-93
1404	-5,95790631	0,3741151	0,04613303	NA	-0,00180249	0,39660544	-0,62074526	-2,09152745	0,35956015	NA	0,00022076	NA	0,43622845	NA	11	-19152,7935	38327,6015	491,184806	7,18E-94
1916	-5,95790631	0,3741151	0,04613303	NA	-0,00180249	0,39660544	-0,62074526	-2,2103983	0,38500536	NA	0,00022113	0,00018745	0,43806177	NA	12	-19151,9194	38327,8561	491,43934	6,32E-94
3572	-10,737769	0,62231984	0,04626548	NA	NA	0,40997724	-0,57836783	-2,59012234	0,38593256	0,02081194	0,00021148	NA	0,47514204	-0,138389	12	-19152,0943	38328,2059	491,78921	5,31E-94
4084	-10,737769	0,62231984	0,04626548	NA	NA	0,40997724	-0,57836783	-2,66976045	0,40191971	0,02073197	0,0002118	0,00011864	0,47565984	-0,13600353	13	-19151,7523	38329,5248	493,108085	2,75E-94
1516	-9,35928532	0,43489058	0,04364193	NA	-0,00182965	NA	-0,59565606	-3,07214819	0,38813022	0,02082345	0,00021556	NA	0,44803127	NA	11	-19155,3204	38332,6554	496,238645	5,74E-95
3451	-8,35316771	NA	0,03864408	NA	-0,00234345	0,43617682	-0,64441699	-1,61349146	0,35721774	NA	0,00021669	NA	0,46315983	-0,13737061	11	-19155,3368	38332,6883	496,271549	5,64E-95
3577	-5,46460783	NA	NA	NA	-0,00220571	0,40342229	-0,70298126	-2,59012234	0,38593256	0,02081194	0,00021148	NA	0,47514204	-0,138389	11	-19155,3931	38332,8007	496,384019	5,34E-95
2028	-9,35928532	0,43489058	0,04364193	NA	-0,00182965	NA	-0,59565606	-3,17508673	0,41103069	0,02071909	0,00021591	0,0001702	0,4495725	NA	12	-19154,6055	38333,2283	496,811546	4,31E-95
3963	-8,35316771	NA	0,03864408	NA	-0,00234345	0,43617682	-0,64441699	-1,71083893	0,37612338	NA	0,00021703	0,00013898	0,46389079	-0,13469622	12	-19154,8636	38333,7444	497,327703	3,33E-95
4089	-5,46460783	NA	NA	NA	-0,00220571	0,40342229	-0,70298126	-2,66976045	0,40191971	0,02073197	0,0002118	0,00011864	0,47565984	-0,13600353	12	-19155,0511	38334,1194	497,702673	2,76E-95
1530	-6,98837994	0,26427623	NA	NA	-0,00180413	0,37036432	-0,69230587	-3,07214819	0,38813022	0,02082345	0,00021556	NA	0,44803127	NA	11	-19158,541	38339,0965	502,679827	2,29E-96
2042	-6,98837994	0,26427623	NA	NA	-0,00180413	0,37036432	-0,69230587	-3,17508673	0,41103069	0,02071909	0,00021591	0,0001702	0,4495725	NA	12	-19157,8261	38339,6694	503,252728	1,72E-96
1524	-10,737769	0,62231984	0,04626548	NA	NA	0,40997724	-0,57836783	-3,07214819	0,38813022	0,02082345	0,00021556	NA	0,44803127	NA	11	-19161,5483	38345,1112	508,694518	1,13E-98
2036	-10,737769	0,62231984	0,04626548	NA	NA	0,40997724	-0,57836783	-3,17508673	0,41103069	0,02071909	0,00021591	0,0001702	0,4495725	NA	12	-19160,8334	38345,6841	509,267419	8,50E-98
3563	-5,75020926	NA	0,0346068	NA	-0,00247017	NA	-0,61951905	-2,59012234	0,38593256	0,02081194	0,00021148	NA	0,47514204	-0,138389	11	-19162,2197	38346,454	510,037262	5,79E-99
3436	-9,35928532	0,43489058	0,04364193	NA	-0,00182965	NA	-0,59565606	-1,61349146	0,35721774	NA	0,00021669	NA	0,46315983	-0,13737061	11	-19162,5345	38347,0836	510,666853	4,22E-99
4075	-5,75020926	NA	0,0346068	NA	-0,00247017	NA	-0,61951905	-2,66976045	0,40191971	0,02073197	0,0002118	0,00011864	0,47565984	-0,13600353	12	-19161,8777	38347,7726	511,355916	2,99E-98
3948	-9,35928532	0,43489058	0,04364193	NA	-0,00182965	NA	-0,59565606	-1,71083893	0,37612338	NA	0,00021703	0,00013898	0,46389079	-0,13469622	12	-19162,0612	38348,1397	511,723006	2,49E-98
1403	-8,35316771	NA	0,03864408	NA	-0,00234345	0,43617682	-0,64441699	-2,09152745	0,35956015	NA	0,00022076	NA	0,43622845	NA	10	-19164,6991	38349,4104	512,993685	1,32E-98
1915	-8,35316771	NA	0,03864408	NA	-0,00234345	0,43617682	-0,64441699	-2,2103983	0,38500536	NA	0,00022113	0,00018745	0,43806177	NA	11	-19163,8251	38349,6647	513,247997	1,16E-98
1529	-5,46460783	NA	NA	NA	-0,00220571	0,40342229	-0,70298126	-3,07214819	0,38813022	0,02082345	0,00021556	NA	0,44803127	NA	10	-19164,8471	38349,7063	513,289548	1,14E-98
2041	-5,46460783	NA	NA	NA	-0,00220571	0,40342229	-0,70298126	-3,17508673	0,41103069	0,02071909	0,00021591	0,0001702	0,4495725	NA	11	-19164,1322	38350,2789	513,862228	8,55E-99
4028	-5,95790631	0,3741151	0,04613303	NA	-0,00180249	0,39660544	-0,62074526	-2,05206432	NA	0,01786019	0,00020271	-0,00039168	0,51529109	-0,14768431	13	-19163,2969	38352,614	516,197316	2,66E-99
3450	-6,98837994	0,26427623	NA	NA	-0,00180413	0,37036432	-0,69230587	-1,61349146	0,35721774	NA	0,00021669	NA	0,46315983	-0,13737061	11	-19165,7551	38353,5248	517,108034	1,69E-99
3562	-6,22256645	0,32760888	NA	NA	-0,00183217	NA	-0,66370241	-2,59012234	0,38593256	0,02081194	0,00021148	NA	0,47514204	-0,138389	11	-19165,7682	38353,551	517,134323	1,66E-99
3962	-6,98837994	0,26427623	NA	NA	-0,00180413	0,37036432	-0,69230587	-1,71083893	0,37612338	NA	0,00021703	0,00013898	0,46389079	-0,13469622	12	-19165,2818	38354,5809	518,164188	9,95E-100
4074	-6,22256645	0,32760888	NA	NA	-0,00183217	NA	-0,66370241	-2,66976045	0,40191971	0,02073197	0,0002118	0,00011864	0,47565984	-0,13600353	12	-19165,4262	38354,8697	518,452977	8,61E-101
3516	-5,95790631	0,3741151	0,04613303	NA	-0,00180249	0,39660544	-0,62074526	-2,27275384	NA	0,01711404	0,00020255	NA	0,52288611	-0,14000571	12	-19167,6377	38359,2926	522,875876	9,43E-101
3444	-10,737769	0,62231984	0,04626548	NA	NA	0,40997724	-0,57836783	-1,61349146	0,35721774	NA	0,00021669	NA	0,46315983	-0,13737061	11	-19168,7624	38359,5394	523,122725	8,34E-101
3956	-10,737769	0,62231984	0,04626548	NA	NA	0,40997724	-0,57836783	-1,71083893	0,37612338	NA	0,00021703	0,00013898	0,46389079	-0,13469622	12	-19168,2892	38360,5956	524,178879	4,92E-101
1515	-5,75020926	NA	0,0346068	NA	-0,00247017	NA	-0,61951905	-3,07214819	0,38813022	0,02082345	0,00021556	NA	0,44803127	NA	10	-19171,6737	38363,3595	526,942792	1,23E-101
1388	-9,35928532	0,43489058	0,04364193	NA	-0,00182965	NA	-0,59565606	-2,09152745	0,35956015	NA	0,00022076	NA	0,43622845	NA	10	-19171,8968	38363,8057	527,388988	9,88E-102
2027	-5,75020926	NA	0,0346068	NA	-0,00247017	NA	-0,61951905	-3,17508673	0,41103069	0,02071909	0,00021591	0,0001702	0,4495725	NA	11	-19170,9588	38363,9322	527,515471	9,27E-102
1900	-9,35928532	0,43489058	0,04364193	NA	-0,00182965	NA	-0,59565606	-2,2103983	0,38500536	NA	0,00022113	0,00018745	0,43806177	NA	11	-19171,0227	38364,06	527,643301	8,70E-102
3449	-5,46460783	NA	NA	NA	-0,00220571	0,40342229	-0,70298126	-1,61349146	0,35721774	NA	0,00021669	NA	0,46315983	-0,13737061	10	-19172,0612	38364,1345	527,717756	8,38E-102
3961	-5,46460783	NA	NA	NA	-0,00220571	0,40342229	-0,70298126	-1,71083893	0,37612338	NA	0,00021703	0,00013898	0,46389079	-0,13469622	11	-19171,5879	38365,1904	528,773688	4,94E-102
3556	-9,79595589	0,69445228	0,04389505	NA	NA	NA	-0,55018722	-2,59012234	0,38593256	0,02081194	0,00021148	NA	0,47514204	-0,138389	11	-19172,5069	38367,0283	530,611612	1,97E-102
4068	-9,79595589	0,69445228	0,04389505	NA	NA	NA	-0,55018722	-2,66976045	0,40191971	0,02073197	0,0002118	0,00011864	0,47565984	-0,13600353	12	-19172,1649	38368,347	531,930266	1,02E-102
1402	-6,98837994	0,26427623	NA	NA	-0,00180413	0,37036432	-0,69230587	-2,09152745	0,35956015	NA	0,00022076	NA	0,43622845	NA	10	-19175,1174	38370,2469	533,83017	3,94E-104
1514	-6,22256645	0,32760888	NA	NA	-0,00183217	NA	-0,66370241	-3,07214819	0,38813022	0,02082345	0,00021556	NA	0,44803127	NA	10	-19175,2222	38370,4566	534,039853	3,55E-103
1914	-6,98837994	0,26427623	NA	NA	-0,00180413	0,37036432	-0,69230587	-2,2103983	0,38500536	NA	0,00022113	0,00018745	0,43806177	NA	11	-19174,2433	38370,5012	534,084482	3,47E-103
3570	-8,51824578	0,51124721	NA	NA	NA	0,38535796	-0,65213053	-2,59012234	0,38593256	0,02081194	0,00021148	NA	0,47514204	-0,138389	11	-19174,4938	38371,0022	534,585517	2,70E-104
2026	-6,22256645	0,32760888	NA	NA	-0,00183217	NA	-0,66370241	-3,17508673	0,41103069	0,02071909	0,00021591	0,0001702	0,4495725	NA	11	-19174,5073	38371,0293	534,612532	2,67E-104
3561	-3,98530292	NA	NA	NA	-0,00234183	NA	-0,67333606	-2,59012234	0,38593256	0,02081194	0,00021148	NA	0,47514204	-0,138389	10	-19175,6393	38371,2908	534,874121	2,34E-103
4082	-8,51824578	0,51124721	NA	NA	NA	0,38535796	-0,65213053	-2,66976045	0,40191971	0,02073197	0,0002118	0,00011864	0,47565984	-0,13600353	12	-19174,1518	38372,3209	535,904171	1,40E-103
1980	-5,95790631	0,3741151	0,04613303	NA	-0,00180249	0,39660544	-0,62074526	-2,58652225	NA	0,01772679	0,00020696	-0,00034396	0,48679163	NA	12	-19174,2427	38372,5027	536,085979	1,28E-103
4073	-3,98530292	NA	NA	NA	-0,00234183	NA	-0,67333606	-2,66976045	0,40191971	0,02073197	0,0002118	0,00011864	0,47565984	-0,13600353	11	-19175,2973	38372,6093	536,192554	1,21E-103
4027	-8,35316771	NA	0,03864408	NA	-0,00234345	0,43617682	-0,64441699	-2,05206432	NA	0,01786019	0,00020271	-0,00039168	0,51529109	-0,14768431	12	-19175,2026	38374,4225	538,005751	4,79E-104
3900	-5,95790631	0,3741151	0,04613303	NA	-0,00180249	0,39660544	-0,62074526	-1,25486087	NA	NA	0,00020772	-0,0003441	0,50313521	-0,14558566	12	-19175,5997	38375,2167	538,799994	3,29E-104
1396	-10,737769	0,62231984	0,04626548	NA	NA	0,40997724	-0,57836783	-2,09152745	0,35956015	NA	0,00022076	NA	0,43622845	NA	10	-19178,1247	38376,2616	539,844861	1,95E-104
1908	-10,737769	0,62231984	0,04626548	NA	NA	0,40997724	-0,57836783	-2,2103983	0,38500536	NA	0,00022113	0,00018745	0,43806177	NA	11	-19177,2506	38376,5159	540,099173	1,72E-104
1468	-5,95790631	0,3741151	0,04613303	NA	-0,00180249	0,39660544	-0,62074526	-2,75826159	NA	0,01709531	0,00020664	NA	0,4949792	NA	11	-19177,6312	38377,277	540,860237	1,17E-104
3435	-5,75020926	NA	0,0346068	NA	-0,00247017	NA	-0,61951905	-1,61349146	0,35721774	NA	0,00021669	NA	0,46315983	-0,13737061	10	-19178,8878	38377,7877	541,370999	9,09E-105
3947	-5,75020926	NA	0,0346068	NA	-0,00247017	NA	-0,61951905	-1,71083893	0,37612338	NA	0,00021703	0,00013898	0,46389079	-0,13469622	11	-19178,4145	38378,8437	542,426931	5,36E-105
3388	-5,95790631	0,3741151	0,04613303	NA	-0,00180249	0,39660544	-0,62074526	-1,4783871	NA	NA	0,00020739	NA	0,51055393	-0,13912561	11	-19178,9937	38380,0019	543,585187	3,00E-105

1401	-5,46460783	NA	NA	NA	-0,00220571	0,40342229	-0,70298126	-2,09152745	0,35956015	NA	0,00022076	NA	0,43622845	NA	9	-19181,4234	38380,8568	544,440113	1,96E-105
3515	-8,35316771	NA	0,03864408	NA	-0,00234345	0,43617682	-0,64441699	-2,27275384	NA	0,01711404	0,00020255	NA	0,52288611	-0,14000571	11	-19179,5433	38381,1013	544,684532	1,73E-105
1913	-5,46460783	NA	NA	NA	-0,00220571	0,40342229	-0,70298126	-2,2103983	0,38500536	NA	0,00022113	0,00018745	0,43806177	NA	10	-19180,5494	38381,1109	544,694204	1,72E-106
1508	-9,79595589	0,69445228	0,04389505	NA	NA	NA	-0,55018722	-3,07214819	0,38813022	0,02082345	0,00021556	NA	0,44803127	NA	10	-19181,9608	38383,9339	547,517141	4,21E-106
2020	-9,79595589	0,69445228	0,04389505	NA	NA	NA	-0,55018722	-3,17508673	0,41103069	0,02071909	0,00021591	0,0001702	0,4495725	NA	11	-19181,246	38384,5065	548,089821	3,16E-106
3434	-6,22256645	0,32760888	NA	NA	-0,00183217	NA	-0,66370241	-1,61349146	0,35721774	NA	0,00021669	NA	0,46315983	-0,13737061	10	-19182,4363	38384,8848	548,46806	2,61E-106
3946	-6,22256645	0,32760888	NA	NA	-0,00183217	NA	-0,66370241	-1,71083893	0,37612338	NA	0,00021703	0,00013898	0,46389079	-0,13469622	11	-19181,9631	38385,9407	549,523992	1,54E-106
1522	-8,51824578	0,51124721	NA	NA	NA	0,38535796	-0,65213053	-3,07214819	0,38813022	0,02082345	0,00021556	NA	0,44803127	NA	10	-19183,9478	38387,9078	551,491047	5,77E-107
1513	-3,98530292	NA	NA	NA	-0,00234183	NA	-0,67333606	-3,07214819	0,38813022	0,02082345	0,00021556	NA	0,44803127	NA	9	-19185,0933	38388,1966	551,779873	4,99E-107
2034	-8,51824578	0,51124721	NA	NA	NA	0,38535796	-0,65213053	-3,17508673	0,41103069	0,02071909	0,00021591	0,0001702	0,4495725	NA	11	-19183,2329	38388,4804	552,063726	4,33E-107
2025	-3,98530292	NA	NA	NA	-0,00234183	NA	-0,67333606	-3,17508673	0,41103069	0,02071909	0,00021591	0,0001702	0,4495725	NA	10	-19184,3784	38388,7691	552,35233	3,75E-107
4012	-9,35928532	0,43489058	0,04364193	NA	-0,00182965	NA	-0,59565606	-2,05206432	NA	0,01786019	0,00020271	-0,00039168	0,51529109	-0,14768431	12	-19182,4003	38388,8178	552,401054	3,66E-108
1979	-8,35316771	NA	0,03864408	NA	-0,00234345	0,43617682	-0,64441699	-2,58652225	NA	0,01772679	0,00020696	-0,00034396	0,48679163	NA	11	-19186,1484	38394,3114	557,894636	2,35E-108
1387	-5,75020926	NA	0,0346068	NA	-0,00247017	NA	-0,61951905	-2,09152745	0,35956015	NA	0,00022076	NA	0,43622845	NA	9	-19188,2501	38394,5101	558,093356	2,12E-108
1852	-5,95790631	0,3741151	0,04613303	NA	-0,00180249	0,39660544	-0,62074526	-1,78635429	NA	NA	0,0002119	-0,00029974	0,47515083	NA	11	-19186,2815	38394,5776	558,160895	2,05E-108
1899	-5,75020926	NA	0,0346068	NA	-0,00247017	NA	-0,61951905	-2,2103983	0,38500536	NA	0,00022113	0,00018745	0,43806177	NA	10	-19187,376	38394,7642	558,347447	1,87E-108
4026	-6,98837994	0,26427623	NA	NA	-0,00180413	0,37036432	-0,69230587	-2,05206432	NA	0,01786019	0,00020271	-0,00039168	0,51529109	-0,14768431	12	-19185,6208	38395,259	558,842236	1,46E-108
3500	-9,35928532	0,43489058	0,04364193	NA	-0,00182965	NA	-0,59565606	-2,27275384	NA	0,01711404	0,00020255	NA	0,52288611	-0,14000571	11	-19186,741	38395,4966	559,079836	1,30E-108
3899	-8,35316771	NA	0,03864408	NA	-0,00234345	0,43617682	-0,64441699	-1,25486087	NA	NA	0,00020772	-0,0003441	0,50313521	-0,14558566	11	-19187,5054	38397,0254	560,608651	6,04E-109
1340	-5,95790631	0,3741151	0,04613303	NA	-0,00180249	0,39660544	-0,62074526	-1,96172815	NA	NA	0,00021146	NA	0,48287384	NA	10	-19188,8864	38397,785	561,368246	4,13E-109
3428	-9,79595589	0,69445228	0,04389505	NA	NA	NA	-0,55018722	-1,61349146	0,35721774	NA	0,00021669	NA	0,46315983	-0,13737061	10	-19189,175	38398,3621	561,945349	3,10E-109
1467	-8,35316771	NA	0,03864408	NA	-0,00234345	0,43617682	-0,64441699	-2,75826159	NA	0,01709531	0,00020664	NA	0,4949792	NA	10	-19189,5368	38399,0858	562,669115	2,16E-109
3940	-9,79595589	0,69445228	0,04389505	NA	NA	NA	-0,55018722	-1,71083893	0,37612338	NA	0,00021703	0,00013898	0,46389079	-0,13469622	11	-19188,7017	38399,418	563,001281	1,83E-109
2556	-5,95790631	0,3741151	0,04613303	NA	-0,00180249	0,39660544	-0,62074526	-2,55577604	0,44288659	0,01897716	0,00021141	NA	NA	-0,08883356	12	-19188,1631	38400,3434	563,926668	1,15E-109
4020	-10,737769	0,62231984	0,04626548	NA	NA	0,40997724	-0,57836783	-2,05206432	NA	0,01786019	0,00020271	-0,00039168	0,51529109	-0,14768431	12	-19188,6282	38401,2736	564,856927	7,22E-112
1386	-6,22256645	0,32760888	NA	NA	-0,00183217	NA	-0,66370241	-2,09152745	0,35956015	NA	0,00022076	NA	0,43622845	NA	9	-19191,7986	38401,6071	565,190417	6,11E-110
3387	-8,35316771	NA	0,03864408	NA	-0,00234345	0,43617682	-0,64441699	-1,4783871	NA	NA	0,00020739	NA	0,51055393	-0,13912561	10	-19190,8993	38401,8108	565,394065	5,52E-110
1898	-6,22256645	0,32760888	NA	NA	-0,00183217	NA	-0,66370241	-2,2103983	0,38500536	NA	0,00022113	0,00018745	0,43806177	NA	10	-19190,9245	38401,8612	565,444508	5,38E-110
3068	-5,95790631	0,3741151	0,04613303	NA	-0,00180249	0,39660544	-0,62074526	-2,61719209	0,45517288	0,01892094	0,00021164	9,21E-05	NA	-0,08713002	13	-19187,9575	38401,9352	565,518476	5,19E-110
3514	-6,98837994	0,26427623	NA	NA	-0,00180413	0,37036432	-0,69230587	-2,27275384	NA	0,01711404	0,00020255	NA	0,52288611	-0,14000571	11	-19189,9616	38401,9377	565,521017	5,18E-111
3442	-8,51824578	0,51124721	NA	NA	NA	0,38535796	-0,65213053	-1,61349146	0,35721774	NA	0,00021669	NA	0,46315983	-0,13737061	10	-19191,1619	38402,336	565,919254	4,24E-110
3433	-3,98530292	NA	NA	NA	-0,00234183	NA	-0,67333606	-1,61349146	0,35721774	NA	0,00021669	NA	0,46315983	-0,13737061	9	-19192,3074	38402,6248	566,20808	3,67E-110
3954	-8,51824578	0,51124721	NA	NA	NA	0,38535796	-0,65213053	-1,71083893	0,37612338	NA	0,00021703	0,00013898	0,46389079	-0,13469622	11	-19190,6887	38403,3919	566,975187	2,50E-110
3945	-3,98530292	NA	NA	NA	-0,00234183	NA	-0,67333606	-1,71083893	0,37612338	NA	0,00021703	0,00013898	0,46389079	-0,13469622	10	-19191,8342	38403,6805	567,263791	2,17E-110
3554	-7,20552725	0,58574016	NA	NA	NA	NA	-0,62020516	-2,59012234	0,38593256	0,02081194	0,00021148	NA	0,47514204	-0,138389	10	-19192,5279	38405,0679	568,651184	1,08E-110
4025	-5,46460783	NA	NA	NA	-0,00220571	0,40342229	-0,70298126	-2,05206432	NA	0,01786019	0,00020271	-0,00039168	0,51529109	-0,14768431	11	-19191,9269	38405,8685	569,451736	7,26E-111
508	-5,95790631	0,3741151	0,04613303	NA	-0,00180249	0,39660544	-0,62074526	-2,87228819	0,44207022	0,01904584	0,00021405	NA	NA	NA	11	-19192,1173	38406,2492	569,832526	6,00E-111
4066	-7,20552725	0,58574016	NA	NA	NA	NA	-0,62020516	-2,66976045	0,40191971	0,02073197	0,0002118	0,00011864	0,47565984	-0,13600353	11	-19192,1859	38406,3863	569,969616	5,60E-111
1020	-5,95790631	0,3741151	0,04613303	NA	-0,00180249	0,39660544	-0,62074526	-2,9468992	0,4586198	0,01897353	0,00021429	0,00012376	NA	NA	12	-19191,7419	38407,5011	571,084423	3,21E-111
3508	-10,737769	0,62231984	0,04626548	NA	NA	0,40997724	-0,57836783	-2,27275384	NA	0,01711404	0,00020255	NA	0,52288611	-0,14000571	11	-19192,9689	38407,9524	571,535708	2,56E-111
1964	-9,35928532	0,43489058	0,04364193	NA	-0,00182965	NA	-0,59565606	-2,58652225	NA	0,01772679	0,00020696	-0,00034396	0,48679163	NA	11	-19193,346	38408,7067	572,289939	1,76E-111
3884	-9,35928532	0,43489058	0,04364193	NA	-0,00182965	NA	-0,59565606	-1,25486087	NA	NA	0,00020772	-0,0003441	0,50313521	-0,14558566	11	-19194,703	38411,4207	575,003954	4,52E-112
3513	-5,46460783	NA	NA	NA	-0,00220571	0,40342229	-0,70298126	-2,27275384	NA	0,01711404	0,00020255	NA	0,52288611	-0,14000571	10	-19196,2676	38412,5475	576,130739	2,57E-112
3571	-8,8387814	NA	0,03010563	NA	NA	0,51295904	-0,60572616	-2,59012234	0,38593256	0,02081194	0,00021148	NA	0,47514204	-0,138389	11	-19195,5445	38413,1037	576,686928	1,95E-112
1452	-9,35928532	0,43489058	0,04364193	NA	-0,00182965	NA	-0,59565606	-2,75826159	NA	0,01709531	0,00020664	NA	0,4949792	NA	10	-19196,7345	38413,4811	577,064419	1,61E-112
4083	-8,8387814	NA	0,03010563	NA	NA	0,51295904	-0,60572616	-2,66976045	0,40191971	0,02073197	0,0002118	0,00011864	0,47565984	-0,13600353	12	-19195,2025	38414,4223	578,005582	1,01E-112
1380	-9,79595589	0,69445228	0,04389505	NA	NA	NA	-0,55018722	-2,09152745	0,35956015	NA	0,00022076	NA	0,43622845	NA	9	-19198,5372	38415,0844	578,667706	7,24E-113
1978	-6,98837994	0,26427623	NA	NA	-0,00180413	0,37036432	-0,69230587	-2,58652225	NA	0,01772679	0,00020696	-0,00034396	0,48679163	NA	11	-19196,5666	38415,1478	578,731121	7,01E-113
1892	-9,79595589	0,69445228	0,04389505	NA	NA	NA	-0,55018722	-2,2103983	0,38500536	NA	0,00022113	0,00018745	0,43806177	NA	10	-19197,6632	38415,3385	578,921797	6,37E-113
3372	-9,35928532	0,43489058	0,04364193	NA	-0,00182965	NA	-0,59565606	-1,4783871	NA	NA	0,00020739	NA	0,51055393	-0,13912561	10	-19198,097	38416,2061	579,789369	4,13E-113
1851	-8,35316771	NA	0,03864408	NA	-0,00234345	0,43617682	-0,64441699	-1,78635429	NA	NA	0,0002119	-0,00029974	0,47515083	NA	10	-19198,1872	38416,3865	579,969773	3,77E-113
3898	-6,98837994	0,26427623	NA	NA	-0,00180413	0,37036432	-0,69230587	-1,25486087	NA	NA	0,00020772	-0,0003441	0,50313521	-0,14558566	11	-19197,9236	38417,8619	581,445136	1,80E-113
1394	-8,51824578	0,51124721	NA	NA	NA	0,38535796	-0,65213053	-2,09152745	0,35956015	NA	0,00022076	NA	0,43622845	NA	9	-19200,5242	38419,0583	582,641612	9,92E-114
1906	-8,51824578	0,51124721	NA	NA	NA	0,38535796	-0,65213053	-2,2103983	0,38500536	NA	0,00022113	0,00018745	0,43806177	NA	10	-19199,6501	38419,3124	582,895703	8,74E-114
1385	-3,98530292	NA	NA	NA	-0,00234183	NA	-0,67333606	-2,09152745	0,35956015	NA	0,00022076	NA	0,43622845	NA	8	-19201,6697	38419,3474	582,930659	8,59E-115
4011	-5,75020926	NA	0,0346068	NA	-0,00247017	NA	-0,61951905	-2,05206432	NA	0,01786019	0,00020271	-0,00039168	0,51529109	-0,14768431	11	-19198,7535	38419,5217	583,104979	7,87E-114
1339	-8,35316771	NA	0,03864408	NA	-0,00234345	0,43617682	-0,64441699	-1,96172815	NA	NA	0,00021146	NA	0,48287384	NA	9	-19200,7921	38419,5941	583,177346	7,59E-114
1897	-3,98530292	NA	NA	NA	-0,00234183	NA	-0,67333606	-2,2103983	0,38500536	NA	0,00022113	0,00018745	0,43806177	NA	9	-19200,7956	38419,6013	583,184528	7,56E-114
1466	-6,98837994	0,26427623	NA	NA	-0,00180413	0,37036432	-0,69230587	-2,75826159	NA	0,01709531	0,00020664	NA	0,4949792	NA	10	-19199,9551	38419,9223	583,5056	6,44E-114
1972	-10,737769	0,62231984	0,04626548	NA	NA	0,40997724	-0,57836783	-2,58652225	NA	0,01772679	0,00020696	-0,00034396	0,48679163	NA	11	-19199,574	38421,1625	584,745812	3,46E-115
1506	-7,20552725	0,58574016	NA	NA	NA	NA	-0,62020516	-3,07214819	0,38813022	0,02082345	0,00021556	NA	0,44803127	NA	9	-19201,9819	38421,9737	585,556935	2,31E-114

2555	-8,35316771	NA	0,03864408	NA	-0,00234345	0,43617682	-0,64441699	-2,55577604	0,44288659	0,01897716	0,00021141	NA	NA	-0,08883356	11	-19200,0687	38422,152	585,735325	2,11E-114
2018	-7,20552725	0,58574016	NA	NA	NA	NA	-0,62020516	-3,17508673	0,41103069	0,02071909	0,00021591	0,0001702	0,4495725	NA	10	-19201,267	38422,5461	586,129393	1,73E-115
3386	-6,98837994	0,26427623	NA	NA	-0,00180413	0,37036432	-0,69230587	-1,4783871	NA	NA	0,00020739	NA	0,51055393	-0,13912561	10	-19201,3176	38422,6473	586,23055	1,65E-114
3067	-8,35316771	NA	0,03864408	NA	-0,00234345	0,43617682	-0,64441699	-2,61719209	0,45517288	0,01892094	0,00021164	9,21E-05	NA	-0,08713002	12	-19199,8632	38423,7436	587,326911	9,53E-115
3892	-10,737769	0,62231984	0,04626548	NA	NA	0,40997724	-0,57836783	-1,25486087	NA	NA	0,00020772	-0,0003441	0,50313521	-0,14558566	11	-19200,931	38423,8765	587,459827	8,92E-115
1977	-5,46460783	NA	NA	NA	-0,00220571	0,40342229	-0,70298126	-2,58652225	NA	0,01772679	0,00020696	-0,00034396	0,48679163	NA	10	-19202,8727	38425,7576	589,340843	3,48E-115
1460	-10,737769	0,62231984	0,04626548	NA	NA	0,40997724	-0,57836783	-2,75826159	NA	0,01709531	0,00020664	NA	0,4949792	NA	10	-19202,9624	38425,937	589,520291	3,18E-115
2428	-5,95790631	0,3741151	0,04613303	NA	-0,00180249	0,39660544	-0,62074526	-1,66433243	0,4162362	NA	0,00021617	NA	NA	-0,08904476	11	-19202,0446	38426,1038	589,687051	2,93E-115
3499	-5,75020926	NA	0,0346068	NA	-0,00247017	NA	-0,61951905	-2,27275384	NA	0,01711404	0,00020255	NA	0,52288611	-0,14000571	10	-19203,0943	38426,2007	589,783982	2,79E-115
4010	-6,22256645	0,32760888	NA	NA	-0,00183217	NA	-0,66370241	-2,05206432	NA	0,01786019	0,00020271	-0,00039168	0,51529109	-0,14768431	11	-19202,3021	38426,6188	590,20204	2,26E-116
2940	-5,95790631	0,3741151	0,04613303	NA	-0,00180249	0,39660544	-0,62074526	-1,73983563	0,43090563	NA	0,00021643	0,00010896	NA	-0,08710954	12	-19201,7549	38427,527	591,110249	1,44E-115
507	-8,35316771	NA	0,03864408	NA	-0,00234345	0,43617682	-0,64441699	-2,87228819	0,44207022	0,01904584	0,00021405	NA	NA	NA	10	-19204,023	38428,0581	591,641404	1,10E-115
3897	-5,46460783	NA	NA	NA	-0,00220571	0,40342229	-0,70298126	-1,25486087	NA	NA	0,00020772	-0,0003441	0,50313521	-0,14558566	10	-19204,2297	38428,4716	592,054857	8,96E-116
3380	-10,737769	0,62231984	0,04626548	NA	NA	0,40997724	-0,57836783	-1,4783871	NA	NA	0,00020739	NA	0,51055393	-0,13912561	10	-19204,3249	38428,662	592,245241	8,15E-116
1019	-8,35316771	NA	0,03864408	NA	-0,00234345	0,43617682	-0,64441699	-2,9468992	0,4586198	0,01897353	0,00021429	0,00012376	NA	NA	11	-19203,6476	38429,3098	592,89308	5,90E-116
1523	-8,8387814	NA	0,03010563	NA	NA	0,51295904	-0,60572616	-3,07214819	0,38813022	0,02082345	0,00021556	NA	0,44803127	NA	10	-19204,9985	38430,0092	593,592458	4,16E-116
1465	-5,46460783	NA	NA	NA	-0,00220571	0,40342229	-0,70298126	-2,75826159	NA	0,01709531	0,00020664	NA	0,4949792	NA	9	-19206,2612	38430,5323	594,115544	3,20E-116
2035	-8,8387814	NA	0,03010563	NA	NA	0,51295904	-0,60572616	-3,17508673	0,41103069	0,02071909	0,00021591	0,0001702	0,4495725	NA	11	-19204,2836	38430,5819	594,165137	3,12E-116
1836	-9,35928532	0,43489058	0,04364193	NA	-0,00182965	NA	-0,59565606	-1,78635429	NA	NA	0,0002119	-0,00029974	0,47515083	NA	10	-19205,3848	38430,7818	594,365077	2,82E-117
3569	-6,3315051	NA	NA	NA	NA	0,4838605	-0,6580268	-2,59012234	0,38593256	0,02081194	0,00021148	NA	0,47514204	-0,138389	10	-19205,9591	38431,9303	595,513567	1,59E-116
380	-5,95790631	0,3741151	0,04613303	NA	-0,00180249	0,39660544	-0,62074526	-1,97841134	0,41538406	NA	0,00021885	NA	NA	NA	10	-19206,0357	38432,0836	595,666881	1,47E-116
892	-5,95790631	0,3741151	0,04613303	NA	-0,00180249	0,39660544	-0,62074526	-2,0661368	0,43415925	NA	0,00021911	0,00013912	NA	NA	11	-19205,5578	38433,1302	596,713452	8,73E-117
4081	-6,3315051	NA	NA	NA	NA	0,4838605	-0,6580268	-2,66976045	0,40191971	0,02073197	0,0002118	0,00011864	0,47565984	-0,13600353	11	-19205,6171	38433,2487	596,832	8,23E-117
3385	-5,46460783	NA	NA	NA	-0,00220571	0,40342229	-0,70298126	-1,4783871	NA	NA	0,00020739	NA	0,51055393	-0,13912561	9	-19207,6236	38433,2572	596,840494	8,19E-117
3498	-6,22256645	0,32760888	NA	NA	-0,00183217	NA	-0,66370241	-2,27275384	NA	0,01711404	0,00020255	NA	0,52288611	-0,14000571	10	-19206,6428	38433,2978	596,881043	8,03E-117
1324	-9,35928532	0,43489058	0,04364193	NA	-0,00182965	NA	-0,59565606	-1,96172815	NA	NA	0,00021146	NA	0,48287384	NA	9	-19207,9897	38433,9894	597,57265	5,68E-117
3426	-7,20552725	0,58574016	NA	NA	NA	NA	-0,62020516	-1,61349146	0,35721774	NA	0,00021669	NA	0,46315983	-0,13737061	9	-19209,196	38436,4019	599,985142	1,70E-117
2540	-9,35928532	0,43489058	0,04364193	NA	-0,00182965	NA	-0,59565606	-2,55577604	0,44288659	0,01897716	0,00021141	NA	NA	-0,08883356	11	-19207,2664	38436,5474	600,130629	1,58E-117
1850	-6,98837994	0,26427623	NA	NA	-0,00180413	0,37036432	-0,69230587	-1,78635429	NA	NA	0,0002119	-0,00029974	0,47515083	NA	10	-19208,6054	38437,223	600,806258	1,13E-117
3938	-7,20552725	0,58574016	NA	NA	NA	NA	-0,62020516	-1,71083893	0,37612338	NA	0,00021703	0,00013898	0,46389079	-0,13469622	10	-19208,7227	38437,4576	601,040853	1,00E-117
3052	-9,35928532	0,43489058	0,04364193	NA	-0,00182965	NA	-0,59565606	-2,61719209	0,45517288	0,01892094	0,00021164	9,21E-05	NA	-0,08713002	12	-19207,0608	38438,1389	601,722215	7,13E-118
1963	-5,75020926	NA	0,0346068	NA	-0,00247017	NA	-0,61951905	-2,58652225	NA	0,01772679	0,00020696	-0,00034396	0,48679163	NA	10	-19209,6993	38439,4108	602,994086	3,78E-119
4004	-9,79595589	0,69445228	0,04389505	NA	NA	NA	-0,55018722	-2,05206432	NA	0,01786019	0,00020271	-0,00039168	0,51529109	-0,14768431	11	-19209,0407	38440,0961	603,679329	2,68E-118
1338	-6,98837994	0,26427623	NA	NA	-0,00180413	0,37036432	-0,69230587	-1,96172815	NA	NA	0,00021146	NA	0,48287384	NA	9	-19211,2103	38440,4306	604,013831	2,27E-119
3883	-5,75020926	NA	0,0346068	NA	-0,00247017	NA	-0,61951905	-1,25486087	NA	NA	0,00020772	-0,0003441	0,50313521	-0,14558566	10	-19211,0563	38442,1248	605,708101	9,72E-119
492	-9,35928532	0,43489058	0,04364193	NA	-0,00182965	NA	-0,59565606	-2,87228819	0,44207022	0,01904584	0,00021405	NA	NA	NA	10	-19211,2206	38442,4534	606,036708	8,25E-119
2554	-6,98837994	0,26427623	NA	NA	-0,00180413	0,37036432	-0,69230587	-2,55577604	0,44288659	0,01897716	0,00021141	NA	NA	-0,08883356	11	-19210,487	38442,9885	606,57181	6,31E-120
1844	-10,737769	0,62231984	0,04626548	NA	NA	0,40997724	-0,57836783	-1,78635429	NA	NA	0,0002119	-0,00029974	0,47515083	NA	10	-19211,6128	38443,2377	606,820949	5,57E-119
1004	-9,35928532	0,43489058	0,04364193	NA	-0,00182965	NA	-0,59565606	-2,9468992	0,4586198	0,01897353	0,00021429	0,00012376	NA	NA	11	-19210,8453	38443,7051	607,288384	4,41E-119
4018	-8,51824578	0,51124721	NA	NA	NA	0,38535796	-0,65213053	-2,05206432	NA	0,01786019	0,00020271	-0,00039168	0,51529109	-0,14768431	11	-19211,0277	38444,07	607,653235	3,68E-119
1451	-5,75020926	NA	0,0346068	NA	-0,00247017	NA	-0,61951905	-2,75826159	NA	0,01709531	0,00020664	NA	0,4949792	NA	9	-19213,0878	38444,1855	607,768787	3,47E-119
4009	-3,98530292	NA	NA	NA	-0,00234183	NA	-0,67333606	-2,05206432	NA	0,01786019	0,00020271	-0,00039168	0,51529109	-0,14768431	10	-19212,1732	38444,3586	607,941838	3,18E-119
3443	-8,8387814	NA	0,03010563	NA	NA	0,51295904	-0,60572616	-1,61349146	0,35721774	NA	0,00021669	NA	0,46315983	-0,13737061	10	-19212,2126	38444,4374	608,020665	3,06E-119
3066	-6,98837994	0,26427623	NA	NA	-0,00180413	0,37036432	-0,69230587	-2,61719209	0,45517288	0,01892094	0,00021164	9,21E-05	NA	-0,08713002	12	-19210,2814	38444,5801	608,163396	2,85E-120
3955	-8,8387814	NA	0,03010563	NA	NA	0,51295904	-0,60572616	-1,71083893	0,37612338	NA	0,00021703	0,00013898	0,46389079	-0,13469622	11	-19211,7394	38445,4933	609,076598	1,80E-119
3548	-9,61919161	0,46601352	0,06534536	NA	-0,00131078	0,33938341	NA	-2,59012234	0,38593256	0,02081194	0,00021148	NA	0,47514204	-0,138389	12	-19210,9168	38445,8508	609,434107	1,51E-119
1332	-10,737769	0,62231984	0,04626548	NA	NA	0,40997724	-0,57836783	-1,96172815	NA	NA	0,00021146	NA	0,48287384	NA	9	-19214,2176	38446,4452	610,028522	1,12E-119
1962	-6,22256645	0,32760888	NA	NA	-0,00183217	NA	-0,66370241	-2,58652225	NA	0,01772679	0,00020696	-0,00034396	0,48679163	NA	10	-19213,2479	38446,5079	610,091147	1,09E-119
3492	-9,79595589	0,69445228	0,04389505	NA	NA	NA	-0,55018722	-2,27275384	NA	0,01711404	0,00020255	NA	0,52288611	-0,14000571	10	-19213,3814	38446,7751	610,358332	9,51E-120
3371	-5,75020926	NA	0,0346068	NA	-0,00247017	NA	-0,61951905	-1,4783871	NA	NA	0,00020739	NA	0,51055393	-0,13912561	9	-19214,4503	38446,9105	610,493737	8,88E-120
4060	-9,61919161	0,46601352	0,06534536	NA	-0,00131078	0,33938341	NA	-2,66976045	0,40191971	0,02073197	0,0002118	0,00011864	0,47565984	-0,13600353	13	-19210,5748	38447,1697	610,752982	7,80E-120
1849	-5,46460783	NA	NA	NA	-0,00220571	0,40342229	-0,70298126	-1,78635429	NA	NA	0,0002119	-0,00029974	0,47515083	NA	9	-19214,9115	38447,8329	611,416201	5,60E-120
2427	-8,35316771	NA	0,03864408	NA	-0,00234345	0,43617682	-0,64441699	-1,66433243	0,4162362	NA	0,00021617	NA	NA	-0,08904476	10	-19213,9502	38447,9127	611,495929	5,38E-120
1521	-6,3315051	NA	NA	NA	NA	0,4838605	-0,6580268	-3,07214819	0,38813022	0,02082345	0,00021556	NA	0,44803127	NA	9	-19215,413	38448,836	612,419318	3,39E-121
506	-6,98837994	0,26427623	NA	NA	-0,00180413	0,37036432	-0,69230587	-2,87228819	0,44207022	0,01904584	0,00021405	NA	NA	NA	10	-19214,4412	38448,8946	612,477889	3,29E-120
2548	-10,737769	0,62231984	0,04626548	NA	NA	0,40997724	-0,57836783	-2,55577604	0,44288659	0,01897716	0,00021141	NA	NA	-0,08883356	11	-19213,4943	38449,0032	612,586501	3,12E-120
3882	-6,22256645	0,32760888	NA	NA	-0,00183217	NA	-0,66370241	-1,25486087	NA	NA	0,00020772	-0,0003441	0,50313521	-0,14558566	10	-19214,6049	38449,2219	612,805162	2,80E-120
2939	-8,35316771	NA	0,03864408	NA	-0,00234345	0,43617682	-0,64441699	-1,73983563	0,43090563	NA	0,00021643	0,00010896	NA	-0,08710954	11	-19213,6605	38449,3356	612,918906	2,64E-120
2033	-6,3315051	NA	NA	NA	NA	0,4838605	-0,6580268	-3,17508673	0,41103069	0,02071909	0,00021591	0,0001702	0,4495725	NA	10	-19214,6982	38449,4085	612,991776	2,55E-120
1018	-6,98837994	0,26427623	NA	NA	-0,00180413	0,37036432	-0,69230587	-2,9468992	0,4586198	0,01897353	0,00021429	0,00012376	NA	NA	11	-19214,0658	38450,1463	613,729565	1,76E-120
3060	-10,737769	0,62231984	0,04626548	NA	NA	0,40997724	-0,57836783	-2,61719209	0,45517288	0,01892094	0,00021164	9,21E-05	NA	-0,08713002	12	-19213,2888	38450,5948	614,178087	1,41E-120

3506	-8,51824578	0,51124721	NA	NA	NA	0,38535796	-0,65213053	-2,27275384	NA	0,01711404	0,00020255	NA	0,52288611	-0,14000571	10	-19215,3684	38450,749	614,332238	1,30E-120
3497	-3,98530292	NA	NA	NA	-0,00234183	NA	-0,67333606	-2,27275384	NA	0,01711404	0,00020255	NA	0,52288611	-0,14000571	9	-19216,5139	38451,0378	614,621063	1,13E-120
1337	-5,46460783	NA	NA	NA	-0,00220571	0,40342229	-0,70298126	-1,96172815	NA	NA	0,00021146	NA	0,48287384	NA	8	-19217,5164	38451,0407	614,623996	1,13E-120
1450	-6,22256645	0,32760888	NA	NA	-0,00183217	NA	-0,66370241	-2,75826159	NA	0,01709531	0,00020664	NA	0,4949792	NA	9	-19216,6363	38451,2826	614,865848	9,98E-121
1378	-7,20552725	0,58574016	NA	NA	NA	NA	-0,62020516	-2,09152745	0,35956015	NA	0,00022076	NA	0,43622845	NA	8	-19218,5582	38453,1244	616,707721	3,97E-121
1890	-7,20552725	0,58574016	NA	NA	NA	NA	-0,62020516	-2,2103983	0,38500536	NA	0,00022113	0,00018745	0,43806177	NA	9	-19217,6842	38453,3783	616,96159	3,50E-122
2553	-5,46460783	NA	NA	NA	-0,00220571	0,40342229	-0,70298126	-2,55577604	0,44288659	0,01897716	0,00021141	NA	NA	-0,08883356	10	-19216,793	38453,5983	617,181532	3,14E-121
379	-8,35316771	NA	0,03864408	NA	-0,00234345	0,43617682	-0,64441699	-1,97841134	0,41538406	NA	0,00021885	NA	NA	NA	9	-19217,9414	38453,8927	617,475981	2,71E-121
3370	-6,22256645	0,32760888	NA	NA	-0,00183217	NA	-0,66370241	-1,4783871	NA	NA	0,00020739	NA	0,51055393	-0,13912561	9	-19217,9988	38454,0075	617,590798	2,56E-121
500	-10,737769	0,62231984	0,04626548	NA	NA	0,40997724	-0,57836783	-2,87228819	0,44207022	0,01904584	0,00021405	NA	NA	NA	10	-19217,4486	38454,9093	618,49258	1,63E-121
891	-8,35316771	NA	0,03864408	NA	-0,00234345	0,43617682	-0,64441699	-2,0661368	0,43415925	NA	0,00021911	0,00013912	NA	NA	10	-19217,4634	38454,9391	618,52233	1,60E-121
3065	-5,46460783	NA	NA	NA	-0,00220571	0,40342229	-0,70298126	-2,61719209	0,45517288	0,01892094	0,00021164	9,21E-05	NA	-0,08713002	11	-19216,5875	38455,1896	618,772896	1,42E-121
1012	-10,737769	0,62231984	0,04626548	NA	NA	0,40997724	-0,57836783	-2,9468992	0,4586198	0,01897353	0,00021429	0,00012376	NA	NA	11	-19217,0732	38456,161	619,744256	8,71E-122
505	-5,46460783	NA	NA	NA	-0,00220571	0,40342229	-0,70298126	-2,87228819	0,44207022	0,01904584	0,00021405	NA	NA	NA	9	-19220,7473	38459,5046	623,087832	1,64E-122
1956	-9,79595589	0,69445228	0,04389505	NA	NA	NA	-0,55018722	-2,58652225	NA	0,01772679	0,00020696	-0,00034396	0,48679163	NA	10	-19219,9865	38459,9852	623,568435	1,29E-122
1017	-5,46460783	NA	NA	NA	-0,00220571	0,40342229	-0,70298126	-2,9468992	0,4586198	0,01897353	0,00021429	0,00012376	NA	NA	10	-19220,3719	38460,756	624,339287	8,75E-123
1395	-8,8387814	NA	0,03010563	NA	NA	0,51295904	-0,60572616	-2,09152745	0,35956015	NA	0,00022076	NA	0,43622845	NA	9	-19221,5749	38461,1597	624,743023	7,15E-123
1907	-8,8387814	NA	0,03010563	NA	NA	0,51295904	-0,60572616	-2,2103983	0,38500536	NA	0,00022113	0,00018745	0,43806177	NA	10	-19220,7008	38461,4138	624,997114	6,30E-123
1835	-5,75020926	NA	0,0346068	NA	-0,00247017	NA	-0,61951905	-1,78635429	NA	NA	0,0002119	-0,00029974	0,47515083	NA	9	-19221,7381	38461,4862	625,069445	6,07E-123
2412	-9,35928532	0,43489058	0,04364193	NA	-0,00182965	NA	-0,59565606	-1,66433243	0,4162362	NA	0,00021617	NA	NA	-0,08904476	10	-19221,1479	38462,308	625,891233	4,03E-123
3876	-9,79595589	0,69445228	0,04389505	NA	NA	NA	-0,55018722	-1,25486087	NA	NA	0,00020772	-0,0003441	0,50313521	-0,14558566	10	-19221,3435	38462,6992	626,28245	3,31E-123
1500	-9,61919161	0,46601352	0,06534536	NA	-0,00131078	0,33938341	NA	-3,07214819	0,38813022	0,02082345	0,00021556	NA	0,44803127	NA	11	-19220,3708	38462,7561	626,339415	3,22E-123
3441	-6,3315051	NA	NA	NA	NA	0,4838605	-0,6580268	-1,61349146	0,35721774	NA	0,00021669	NA	0,46315983	-0,13737061	9	-19222,6271	38463,2642	626,847526	2,50E-123
2012	-9,61919161	0,46601352	0,06534536	NA	-0,00131078	0,33938341	NA	-3,17508673	0,41103069	0,02071909	0,00021591	0,0001702	0,4495725	NA	12	-19219,6559	38463,329	626,912316	2,42E-124
2924	-9,35928532	0,43489058	0,04364193	NA	-0,00182965	NA	-0,59565606	-1,73983563	0,43090563	NA	0,00021643	0,00010896	NA	-0,08710954	11	-19220,8582	38463,7309	627,314209	1,98E-123
1970	-8,51824578	0,51124721	NA	NA	NA	0,38535796	-0,65213053	-2,58652225	NA	0,01772679	0,00020696	-0,00034396	0,48679163	NA	10	-19221,9734	38463,9591	627,542341	1,76E-123
1961	-3,98530292	NA	NA	NA	-0,00234183	NA	-0,67333606	-2,58652225	NA	0,01772679	0,00020696	-0,00034396	0,48679163	NA	9	-19223,119	38464,2479	627,831167	1,53E-123
3953	-6,3315051	NA	NA	NA	NA	0,4838605	-0,6580268	-1,71083893	0,37612338	NA	0,00021703	0,00013898	0,46389079	-0,13469622	10	-19222,1539	38464,32	627,903236	1,47E-123
1323	-5,75020926	NA	0,0346068	NA	-0,00247017	NA	-0,61951905	-1,96172815	NA	NA	0,00021146	NA	0,48287384	NA	8	-19224,343	38464,694	628,27724	1,22E-123
1444	-9,79595589	0,69445228	0,04389505	NA	NA	NA	-0,55018722	-2,75826159	NA	0,01709531	0,00020664	NA	0,4949792	NA	9	-19223,375	38464,7599	628,343137	1,18E-124
3890	-8,51824578	0,51124721	NA	NA	NA	0,38535796	-0,65213053	-1,25486087	NA	NA	0,00020772	-0,0003441	0,50313521	-0,14558566	10	-19223,3305	38466,6731	630,256356	4,54E-125
3881	-3,98530292	NA	NA	NA	-0,00234183	NA	-0,67333606	-1,25486087	NA	NA	0,00020772	-0,0003441	0,50313521	-0,14558566	9	-19224,476	38466,9619	630,545182	3,93E-124
2539	-5,75020926	NA	0,0346068	NA	-0,00247017	NA	-0,61951905	-2,55577604	0,44288659	0,01897716	0,00021141	NA	NA	-0,08883356	10	-19223,6197	38467,2515	630,834775	3,40E-124
3364	-9,79595589	0,69445228	0,04389505	NA	NA	NA	-0,55018722	-1,4783871	NA	NA	0,00020739	NA	0,51055393	-0,13912561	9	-19224,7374	38467,4848	631,068086	3,03E-124
364	-9,35928532	0,43489058	0,04364193	NA	-0,00182965	NA	-0,59565606	-1,97841134	0,41538406	NA	0,00021885	NA	NA	NA	9	-19225,139	38468,288	631,871285	2,03E-124
1834	-6,22256645	0,32760888	NA	NA	-0,00183217	NA	-0,66370241	-1,78635429	NA	NA	0,0002119	-0,00029974	0,47515083	NA	9	-19225,2866	38468,5832	632,166506	1,75E-124
1458	-8,51824578	0,51124721	NA	NA	NA	0,38535796	-0,65213053	-2,75826159	NA	0,01709531	0,00020664	NA	0,4949792	NA	9	-19225,3619	38468,7338	632,317042	1,62E-124
2426	-6,98837994	0,26427623	NA	NA	-0,00180413	0,37036432	-0,69230587	-1,66433243	0,4162362	NA	0,00021617	NA	NA	-0,08904476	10	-19224,3685	38468,7491	632,332414	1,61E-124
3051	-5,75020926	NA	0,0346068	NA	-0,00247017	NA	-0,61951905	-2,61719209	0,45517288	0,01892094	0,00021164	9,21E-05	NA	-0,08713002	11	-19223,4141	38468,8429	632,42614	1,53E-124
1449	-3,98530292	NA	NA	NA	-0,00234183	NA	-0,67333606	-2,75826159	NA	0,01709531	0,00020664	NA	0,4949792	NA	8	-19226,5074	38469,0228	632,60609	1,40E-124
876	-9,35928532	0,43489058	0,04364193	NA	-0,00182965	NA	-0,59565606	-2,0661368	0,43415925	NA	0,00021911	0,00013912	NA	NA	10	-19224,6611	38469,3344	632,917634	1,20E-124
2938	-6,98837994	0,26427623	NA	NA	-0,00180413	0,37036432	-0,69230587	-1,73983563	0,43090563	NA	0,00021643	0,00010896	NA	-0,08710954	11	-19224,0788	38470,1721	633,755391	7,90E-125
3378	-8,51824578	0,51124721	NA	NA	NA	0,38535796	-0,65213053	-1,4783871	NA	NA	0,00020739	NA	0,51055393	-0,13912561	9	-19226,7244	38471,4587	635,041992	4,15E-125
3540	-13,7669049	0,65178191	0,06462958	NA	NA	0,35119819	NA	-2,59012234	0,38593256	0,02081194	0,00021148	NA	0,47514204	-0,138389	11	-19224,7991	38471,6128	635,196067	3,84E-125
3369	-3,98530292	NA	NA	NA	-0,00234183	NA	-0,67333606	-1,4783871	NA	NA	0,00020739	NA	0,51055393	-0,13912561	8	-19227,8699	38471,7478	635,331039	3,59E-125
1322	-6,22256645	0,32760888	NA	NA	-0,00183217	NA	-0,66370241	-1,96172815	NA	NA	0,00021146	NA	0,48287384	NA	8	-19227,8915	38471,791	635,374301	3,51E-125
3532	-11,9654714	0,51367836	0,06255183	NA	-0,0013444	NA	NA	-2,59012234	0,38593256	0,02081194	0,00021148	NA	0,47514204	-0,138389	11	-19224,9877	38471,9899	635,573211	3,18E-125
4052	-13,7669049	0,65178191	0,06462958	NA	NA	0,35119819	NA	-2,66976045	0,40191971	0,02073197	0,0002118	0,00011864	0,47565984	-0,13600353	12	-19224,4571	38472,9314	636,514721	1,99E-126
491	-5,75020926	NA	0,0346068	NA	-0,00247017	NA	-0,61951905	-2,87228819	0,44207022	0,01904584	0,00021405	NA	NA	NA	9	-19227,5739	38473,1578	636,741076	1,77E-125
4044	-11,9654714	0,51367836	0,06255183	NA	-0,0013444	NA	NA	-2,66976045	0,40191971	0,02073197	0,0002118	0,00011864	0,47565984	-0,13600353	12	-19224,6457	38473,3086	636,891864	1,65E-126
2538	-6,22256645	0,32760888	NA	NA	-0,00183217	NA	-0,66370241	-2,55577604	0,44288659	0,01897716	0,00021141	NA	NA	-0,08883356	10	-19227,1682	38474,3486	637,931836	9,78E-126
1003	-5,75020926	NA	0,0346068	NA	-0,00247017	NA	-0,61951905	-2,9468992	0,4586198	0,01897353	0,00021429	0,00012376	NA	NA	10	-19227,1985	38474,4093	637,99253	9,49E-126
378	-6,98837994	0,26427623	NA	NA	-0,00180413	0,37036432	-0,69230587	-1,97841134	0,41538406	NA	0,00021885	NA	NA	NA	9	-19228,3596	38474,7292	638,312466	8,09E-126
2420	-10,737769	0,62231984	0,04626548	NA	NA	0,40997724	-0,57836783	-1,66433243	0,4162362	NA	0,00021617	NA	NA	-0,08904476	10	-19227,3758	38474,7638	638,347106	7,95E-126
890	-6,98837994	0,26427623	NA	NA	-0,00180413	0,37036432	-0,69230587	-2,0661368	0,43415925	NA	0,00021911	0,00013912	NA	NA	10	-19227,8817	38475,7755	639,358816	4,79E-126
3050	-6,22256645	0,32760888	NA	NA	-0,00183217	NA	-0,66370241	-2,61719209	0,45517288	0,01892094	0,00021164	9,21E-05	NA	-0,08713002	11	-19226,9627	38475,9399	639,523201	4,41E-126
2932	-10,737769	0,62231984	0,04626548	NA	NA	0,40997724	-0,57836783	-1,73983563	0,43090563	NA	0,00021643	0,00010896	NA	-0,08710954	11	-19227,0861	38476,1868	639,770082	3,90E-126
3555	-9,52399274	NA	0,02470059	NA	NA	NA	-0,56950148	-2,59012234	0,38593256	0,02081194	0,00021148	NA	0,47514204	-0,138389	10	-19228,5842	38477,1806	640,763842	2,37E-127
3420	-9,61919161	0,46601352	0,06534536	NA	-0,00131078	0,33938341	NA	-1,61349146	0,35721774	NA	0,00021669	NA	0,46315983	-0,13737061	11	-19227,5849	38477,1843	640,767622	2,37E-126
4002	-7,20552725	0,58574016	NA	NA	NA	NA	-0,62020516	-2,05206432	NA	0,01786019	0,00020271	-0,00039168	0,51529109	-0,14768431	10	-19229,0617	38478,1356	641,718901	1,47E-126
3932	-9,61919161	0,46601352	0,06534536	NA	-0,00131078	0,33938341	NA	-1,71083893	0,37612338	NA	0,00021703	0,00013898	0,46389079	-0,13469622	12	-19227,1116	38478,2405	641,823776	1,40E-126

4067	-9,52399274	NA	0,02470059	NA	NA	NA	-0,56950148	-2,66976045	0,40191971	0,02073197	0,0002118	0,00011864	0,47565984	-0,13600353	11	-19228,2422	38478,499	642,082274	1,23E-126
2425	-5,46460783	NA	NA	NA	-0,00220571	0,40342229	-0,70298126	-1,66433243	0,4162362	NA	0,00021617	NA	NA	-0,08904476	9	-19230,6746	38479,3591	642,942358	7,99E-127
1393	-6,3315051	NA	NA	NA	NA	0,4838605	-0,6580268	-2,09152745	0,35956015	NA	0,00022076	NA	0,43622845	NA	8	-19231,9894	38479,9868	643,570105	5,84E-127
1905	-6,3315051	NA	NA	NA	NA	0,4838605	-0,6580268	-2,2103983	0,38500536	NA	0,00022113	0,00018745	0,43806177	NA	9	-19231,1154	38480,2407	643,823974	5,14E-127
490	-6,22256645	0,32760888	NA	NA	-0,00183217	NA	-0,66370241	-2,87228819	0,44207022	0,01904584	0,00021405	NA	NA	NA	9	-19231,1225	38480,2549	643,838137	5,10E-128
372	-10,737769	0,62231984	0,04626548	NA	NA	0,40997724	-0,57836783	-1,97841134	0,41538406	NA	0,00021885	NA	NA	NA	9	-19231,367	38480,7439	644,327157	4,00E-127
2937	-5,46460783	NA	NA	NA	-0,00220571	0,40342229	-0,70298126	-1,73983563	0,43090563	NA	0,00021643	0,00010896	NA	-0,08710954	10	-19230,3848	38480,7818	644,365112	3,92E-127
3547	-11,3659105	NA	0,05744064	NA	-0,00198557	0,38445946	NA	-2,59012234	0,38593256	0,02081194	0,00021148	NA	0,47514204	-0,138389	11	-19229,4097	38480,834	644,41729	3,82E-127
1002	-6,22256645	0,32760888	NA	NA	-0,00183217	NA	-0,66370241	-2,9468992	0,4586198	0,01897353	0,00021429	0,00012376	NA	NA	10	-19230,7471	38481,5063	645,089591	2,73E-127
884	-10,737769	0,62231984	0,04626548	NA	NA	0,40997724	-0,57836783	-2,0661368	0,43415925	NA	0,00021911	0,00013912	NA	NA	10	-19230,889	38481,7902	645,373507	2,37E-127
1828	-9,79595589	0,69445228	0,04389505	NA	NA	NA	-0,55018722	-1,78635429	NA	NA	0,0002119	-0,00029974	0,47515083	NA	9	-19232,0253	38482,0605	645,643794	2,07E-127
4059	-11,3659105	NA	0,05744064	NA	-0,00198557	0,38445946	NA	-2,66976045	0,40191971	0,02073197	0,0002118	0,00011864	0,47565984	-0,13600353	12	-19229,0677	38482,1527	645,735944	1,98E-127
3490	-7,20552725	0,58574016	NA	NA	NA	NA	-0,62020516	-2,27275384	NA	0,01711404	0,00020255	NA	0,52288611	-0,14000571	9	-19233,4024	38484,8148	648,398126	5,22E-128
1316	-9,79595589	0,69445228	0,04389505	NA	NA	NA	-0,55018722	-1,96172815	NA	NA	0,00021146	NA	0,48287384	NA	8	-19234,6302	38485,2683	648,851589	4,16E-129
377	-5,46460783	NA	NA	NA	-0,00220571	0,40342229	-0,70298126	-1,97841134	0,41538406	NA	0,00021885	NA	NA	NA	8	-19234,6657	38485,3394	648,922631	4,02E-128
1842	-8,51824578	0,51124721	NA	NA	NA	0,38535796	-0,65213053	-1,78635429	NA	NA	0,0002119	-0,00029974	0,47515083	NA	9	-19234,0122	38486,0344	649,6177	2,84E-128
4019	-8,8387814	NA	0,03010563	NA	NA	0,51295904	-0,60572616	-2,05206432	NA	0,01786019	0,00020271	-0,00039168	0,51529109	-0,14768431	11	-19232,0784	38486,1714	649,754646	2,65E-128
1833	-3,98530292	NA	NA	NA	-0,00234183	NA	-0,67333606	-1,78635429	NA	NA	0,0002119	-0,00029974	0,47515083	NA	8	-19235,1578	38486,3235	649,906747	2,46E-128
889	-5,46460783	NA	NA	NA	-0,00220571	0,40342229	-0,70298126	-2,0661368	0,43415925	NA	0,00021911	0,00013912	NA	NA	9	-19234,1878	38486,3855	649,968759	2,38E-128
2532	-9,79595589	0,69445228	0,04389505	NA	NA	NA	-0,55018722	-2,55577604	0,44288659	0,01897716	0,00021141	NA	NA	-0,08883356	10	-19233,9068	38487,8258	651,409125	1,16E-128
1492	-13,7669049	0,65178191	0,06462958	NA	NA	0,35119819	NA	-3,07214819	0,38813022	0,02082345	0,00021556	NA	0,44803127	NA	10	-19234,2531	38488,5183	652,101597	8,20E-129
1484	-11,9654714	0,51367836	0,06255183	NA	-0,0013444	NA	NA	-3,07214819	0,38813022	0,02082345	0,00021556	NA	0,44803127	NA	10	-19234,4416	38488,8955	652,47874	6,79E-129
2004	-13,7669049	0,65178191	0,06462958	NA	NA	0,35119819	NA	-3,17508673	0,41103069	0,02071909	0,00021591	0,0001702	0,4495725	NA	11	-19233,5382	38489,091	652,674276	6,15E-130
3553	-8,58193391	NA	NA	NA	NA	NA	-0,61393687	-2,59012234	0,38593256	0,02081194	0,00021148	NA	0,47514204	-0,138389	9	-19235,6003	38489,2105	652,793791	5,80E-129
1330	-8,51824578	0,51124721	NA	NA	NA	0,38535796	-0,65213053	-1,96172815	NA	NA	0,00021146	NA	0,48287384	NA	8	-19236,6171	38489,2422	652,825495	5,71E-129
3044	-9,79595589	0,69445228	0,04389505	NA	NA	NA	-0,55018722	-2,61719209	0,45517288	0,01892094	0,00021164	9,21E-05	NA	-0,08713002	11	-19233,7013	38489,4172	653,000489	5,23E-129
1996	-11,9654714	0,51367836	0,06255183	NA	-0,0013444	NA	NA	-3,17508673	0,41103069	0,02071909	0,00021591	0,0001702	0,4495725	NA	11	-19233,7268	38489,4681	653,05142	5,10E-130
1321	-3,98530292	NA	NA	NA	-0,00234183	NA	-0,67333606	-1,96172815	NA	NA	0,00021146	NA	0,48287384	NA	7	-19237,7626	38489,5315	653,114764	4,94E-129
4065	-8,58193391	NA	NA	NA	NA	NA	-0,61393687	-2,66976045	0,40191971	0,02073197	0,0002118	0,00011864	0,47565984	-0,13600353	10	-19235,2583	38490,5287	654,112002	3,00E-129
2546	-8,51824578	0,51124721	NA	NA	NA	0,38535796	-0,65213053	-2,55577604	0,44288659	0,01897716	0,00021141	NA	NA	-0,08883356	10	-19235,8938	38491,7998	655,38303	1,59E-130
2537	-3,98530292	NA	NA	NA	-0,00234183	NA	-0,67333606	-2,55577604	0,44288659	0,01897716	0,00021141	NA	NA	-0,08883356	9	-19237,0393	38492,0886	655,671856	1,37E-129
3507	-8,8387814	NA	0,03010563	NA	NA	0,51295904	-0,60572616	-2,27275384	NA	0,01711404	0,00020255	NA	0,52288611	-0,14000571	10	-19236,4191	38492,8504	656,433649	9,39E-130
2411	-5,75020926	NA	0,0346068	NA	-0,00247017	NA	-0,61951905	-1,66433243	0,4162362	NA	0,00021617	NA	NA	-0,08904476	9	-19237,5012	38493,0123	656,595601	8,66E-130
3058	-8,51824578	0,51124721	NA	NA	NA	0,38535796	-0,65213053	-2,61719209	0,45517288	0,01892094	0,00021164	9,21E-05	NA	-0,08713002	11	-19235,6883	38493,3911	656,974395	7,17E-130
3049	-3,98530292	NA	NA	NA	-0,00234183	NA	-0,67333606	-2,61719209	0,45517288	0,01892094	0,00021164	9,21E-05	NA	-0,08713002	10	-19236,8338	38493,6797	657,262999	6,21E-130
484	-9,79595589	0,69445228	0,04389505	NA	NA	NA	-0,55018722	-2,87228819	0,44207022	0,01904584	0,00021405	NA	NA	NA	9	-19237,8611	38493,7321	657,315425	6,04E-130
1372	-9,61919161	0,46601352	0,06534536	NA	-0,00131078	0,33938341	NA	-2,09152745	0,35956015	NA	0,00022076	NA	0,43622845	NA	10	-19236,9472	38493,9065	657,489758	5,54E-130
1507	-9,52399274	NA	0,02470059	NA	NA	NA	-0,56950148	-3,07214819	0,38813022	0,02082345	0,00021556	NA	0,44803127	NA	9	-19238,0382	38494,0863	657,669593	5,06E-130
1884	-9,61919161	0,46601352	0,06534536	NA	-0,00131078	0,33938341	NA	-2,2103983	0,38500536	NA	0,00022113	0,00018745	0,43806177	NA	11	-19236,0731	38494,1608	657,74407	4,88E-130
2923	-5,75020926	NA	0,0346068	NA	-0,00247017	NA	-0,61951905	-1,73983563	0,43090563	NA	0,00021643	0,00010896	NA	-0,08710954	10	-19237,2115	38494,4351	658,018356	4,25E-130
2019	-9,52399274	NA	0,02470059	NA	NA	NA	-0,56950148	-3,17508673	0,41103069	0,02071909	0,00021591	0,0001702	0,4495725	NA	10	-19237,3233	38494,6588	658,242051	3,80E-130
996	-9,79595589	0,69445228	0,04389505	NA	NA	NA	-0,55018722	-2,9468992	0,4586198	0,01897353	0,00021429	0,00012376	NA	NA	10	-19237,4857	38494,9836	658,56688	3,23E-130
3004	-5,95790631	0,3741151	0,04613303	NA	-0,00180249	0,39660544	-0,62074526	-1,91049389	NA	0,01549989	0,00020133	-0,00049532	NA	-0,09486783	12	-19236,3172	38496,6516	660,234839	1,40E-130
498	-8,51824578	0,51124721	NA	NA	NA	0,38535796	-0,65213053	-2,87228819	0,44207022	0,01904584	0,00021405	NA	NA	NA	9	-19239,848	38497,7061	661,289331	8,29E-131
1499	-11,3659105	NA	0,05744064	NA	-0,00198557	0,38445946	NA	-3,07214819	0,38813022	0,02082345	0,00021556	NA	0,44803127	NA	10	-19238,8637	38497,7395	661,32282	8,15E-131
489	-3,98530292	NA	NA	NA	-0,00234183	NA	-0,67333606	-2,87228819	0,44207022	0,01904584	0,00021405	NA	NA	NA	8	-19240,9936	38497,9951	661,578378	7,17E-132
1954	-7,20552725	0,58574016	NA	NA	NA	NA	-0,62020516	-2,58652225	NA	0,01772679	0,00020696	-0,00034396	0,48679163	NA	9	-19240,0075	38498,025	661,608229	7,07E-131
2011	-11,3659105	NA	0,05744064	NA	-0,00198557	0,38445946	NA	-3,17508673	0,41103069	0,02071909	0,00021591	0,0001702	0,4495725	NA	11	-19238,1488	38498,3122	661,895499	6,12E-131
1010	-8,51824578	0,51124721	NA	NA	NA	0,38535796	-0,65213053	-2,9468992	0,4586198	0,01897353	0,00021429	0,00012376	NA	NA	10	-19239,4727	38498,9575	662,540786	4,43E-131
363	-5,75020926	NA	0,0346068	NA	-0,00247017	NA	-0,61951905	-1,97841134	0,41538406	NA	0,00021885	NA	NA	NA	8	-19241,4923	38498,9926	662,575874	4,36E-131
1001	-3,98530292	NA	NA	NA	-0,00234183	NA	-0,67333606	-2,9468992	0,4586198	0,01897353	0,00021429	0,00012376	NA	NA	9	-19240,6182	38499,2463	662,829611	3,84E-131
3524	-12,0135881	0,70944129	0,06178489	NA	NA	NA	NA	-2,59012234	0,38593256	0,02081194	0,00021148	NA	0,47514204	-0,138389	10	-19239,8861	38499,7843	663,367608	2,93E-131
875	-5,75020926	NA	0,0346068	NA	-0,00247017	NA	-0,61951905	-2,0661368	0,43415925	NA	0,00021911	0,00013912	NA	NA	9	-19241,0144	38500,0387	663,622002	2,58E-131
2410	-6,22256645	0,32760888	NA	NA	-0,00183217	NA	-0,66370241	-1,66433243	0,4162362	NA	0,00021617	NA	NA	-0,08904476	9	-19241,0497	38500,1094	663,692662	2,49E-131
3874	-7,20552725	0,58574016	NA	NA	NA	NA	-0,62020516	-1,25486087	NA	NA	0,00020772	-0,0003441	0,50313521	-0,14558566	9	-19241,3645	38500,739	664,322244	1,82E-131
4036	-12,0135881	0,70944129	0,06178489	NA	NA	NA	NA	-2,66976045	0,40191971	0,02073197	0,0002118	0,00011864	0,47565984	-0,13600353	11	-19239,5441	38501,1028	664,686041	1,52E-131
2922	-6,22256645	0,32760888	NA	NA	-0,00183217	NA	-0,66370241	-1,73983563	0,43090563	NA	0,00021643	0,00010896	NA	-0,08710954	10	-19240,76	38501,5321	665,115417	1,22E-131
1442	-7,20552725	0,58574016	NA	NA	NA	NA	-0,62020516	-2,75826159	NA	0,01709531	0,00020664	NA	0,4949792	NA	8	-19243,396	38502,7999	666,383152	6,49E-132
3412	-13,7669049	0,65178191	0,06462958	NA	NA	0,35119819	NA	-1,61349146	0,35721774	NA	0,00021669	NA	0,46315983	-0,13737061	10	-19241,4672	38502,9465	666,529804	6,03E-132
3404	-11,9654714	0,51367836	0,06255183	NA	-0,0013444	NA	NA	-1,61349146	0,35721774	NA	0,00021669	NA	0,46315983	-0,13737061	10	-19241,6558	38503,3237	666,906948	5,00E-132
956	-5,95790631	0,3741151	0,04613303	NA	-0,00180249	0,39660544	-0,62074526	-2,26386687	NA	0,01548996	0,00020413	-0,00046266	NA	NA	11	-19240,9147	38503,844	667,427326	3,85E-132

3924	-13,7669049	0,65178191	0,06462958	NA	NA	0,35119819	NA	-1,71083893	0,37612338	NA	0,00021703	0,00013898	0,46389079	-0,13469622	11	-19240,9939	38504,0025	667,585736	3,56E-132
3916	-11,9654714	0,51367836	0,06255183	NA	-0,0013444	NA	NA	-1,71083893	0,37612338	NA	0,00021703	0,00013898	0,46389079	-0,13469622	11	-19241,1825	38504,3796	667,96288	2,95E-132
4017	-6,3315051	NA	NA	NA	NA	0,4838605	-0,6580268	-2,05206432	NA	0,01786019	0,00020271	-0,00039168	0,51529109	-0,14768431	10	-19242,4929	38504,998	668,581284	2,16E-132
3362	-7,20552725	0,58574016	NA	NA	NA	NA	-0,62020516	-1,4783871	NA	NA	0,00020739	NA	0,51055393	-0,13912561	8	-19244,7584	38505,5248	669,108102	1,66E-132
1971	-8,8387814	NA	0,03010563	NA	NA	0,51295904	-0,60572616	-2,58652225	NA	0,01772679	0,00020696	-0,00034396	0,48679163	NA	10	-19243,0242	38506,0605	669,643752	1,27E-132
362	-6,22256645	0,32760888	NA	NA	-0,00183217	NA	-0,66370241	-1,97841134	0,41538406	NA	0,00021885	NA	NA	NA	8	-19245,0408	38506,0897	669,672935	1,25E-133
1505	-8,58193391	NA	NA	NA	NA	NA	-0,61393687	-3,07214819	0,38813022	0,02082345	0,00021556	NA	0,44803127	NA	8	-19245,0543	38506,1165	669,699764	1,24E-132
2017	-8,58193391	NA	NA	NA	NA	NA	-0,61393687	-3,17508673	0,41103069	0,02071909	0,00021591	0,0001702	0,4495725	NA	9	-19244,3394	38506,6887	670,272	9,29E-133
874	-6,22256645	0,32760888	NA	NA	-0,00183217	NA	-0,66370241	-2,0661368	0,43415925	NA	0,00021911	0,00013912	NA	NA	9	-19244,5629	38507,1358	670,719063	7,43E-133
2492	-5,95790631	0,3741151	0,04613303	NA	-0,00180249	0,39660544	-0,62074526	-2,18775169	NA	0,01458917	0,00020109	NA	NA	-0,08533198	11	-19243,1781	38508,3708	671,954081	4,01E-133
3427	-9,52399274	NA	0,02470059	NA	NA	NA	-0,56950148	-1,61349146	0,35721774	NA	0,00021669	NA	0,46315983	-0,13737061	9	-19245,2523	38508,5145	672,097801	3,73E-133
3891	-8,8387814	NA	0,03010563	NA	NA	0,51295904	-0,60572616	-1,25486087	NA	NA	0,00020772	-0,0003441	0,50313521	-0,14558566	10	-19244,3812	38508,7745	672,357767	3,27E-134
3939	-9,52399274	NA	0,02470059	NA	NA	NA	-0,56950148	-1,71083893	0,37612338	NA	0,00021703	0,00013898	0,46389079	-0,13469622	10	-19244,779	38509,5702	673,153511	2,20E-133
1459	-8,8387814	NA	0,03010563	NA	NA	0,51295904	-0,60572616	-2,75826159	NA	0,01709531	0,00020664	NA	0,4949792	NA	9	-19246,4126	38510,8352	674,418453	1,17E-133
3505	-6,3315051	NA	NA	NA	NA	0,4838605	-0,6580268	-2,27275384	NA	0,01711404	0,00020255	NA	0,52288611	-0,14000571	9	-19246,8336	38511,6772	675,260509	7,67E-134
3419	-11,3659105	NA	0,05744064	NA	-0,00198557	0,38445946	NA	-1,61349146	0,35721774	NA	0,00021669	NA	0,46315983	-0,13737061	10	-19246,0778	38512,1677	675,751027	6,00E-134
2876	-5,95790631	0,3741151	0,04613303	NA	-0,00180249	0,39660544	-0,62074526	-1,2176888	NA	NA	0,00020569	-0,00045432	NA	-0,0941422	11	-19245,5869	38513,1885	676,771751	3,60E-134
3931	-11,3659105	NA	0,05744064	NA	-0,00198557	0,38445946	NA	-1,71083893	0,37612338	NA	0,00021703	0,00013898	0,46389079	-0,13469622	11	-19245,6045	38513,2237	676,806959	3,54E-134
3379	-8,8387814	NA	0,03010563	NA	NA	0,51295904	-0,60572616	-1,4783871	NA	NA	0,00020739	NA	0,51055393	-0,13912561	9	-19247,7751	38513,5601	677,143403	2,99E-134
2404	-9,79595589	0,69445228	0,04389505	NA	NA	NA	-0,55018722	-1,66433243	0,4162362	NA	0,00021617	NA	NA	-0,08904476	9	-19247,7884	38513,5867	677,169951	2,95E-134
444	-5,95790631	0,3741151	0,04613303	NA	-0,00180249	0,39660544	-0,62074526	-2,49206855	NA	0,01465098	0,00020366	NA	NA	NA	10	-19246,968	38513,9483	677,531542	2,46E-134
2916	-9,79595589	0,69445228	0,04389505	NA	NA	NA	-0,55018722	-1,73983563	0,43090563	NA	0,00021643	0,00010896	NA	-0,08710954	10	-19247,4986	38515,0094	678,592705	1,45E-134
3531	-9,45532716	NA	0,05329757	NA	-0,00210061	NA	NA	-2,59012234	0,38593256	0,02081194	0,00021148	NA	0,47514204	-0,138389	10	-19247,7652	38515,5427	679,125933	1,11E-134
1476	-12,0135881	0,70944129	0,06178489	NA	NA	NA	NA	-3,07214819	0,38813022	0,02082345	0,00021556	NA	0,44803127	NA	9	-19249,3401	38516,6901	680,273359	6,25E-135
4043	-9,45532716	NA	0,05329757	NA	-0,00210061	NA	NA	-2,66976045	0,40191971	0,02073197	0,0002118	0,00011864	0,47565984	-0,13600353	11	-19247,4232	38516,8611	680,444365	5,74E-135
1988	-12,0135881	0,70944129	0,06178489	NA	NA	NA	NA	-3,17508673	0,41103069	0,02071909	0,00021591	0,0001702	0,4495725	NA	10	-19248,6252	38517,2625	680,845817	4,70E-135
2418	-8,51824578	0,51124721	NA	NA	NA	0,38535796	-0,65213053	-1,66433243	0,4162362	NA	0,00021617	NA	NA	-0,08904476	9	-19249,7753	38517,5606	681,143857	4,05E-136
2409	-3,98530292	NA	NA	NA	-0,00234183	NA	-0,67333606	-1,66433243	0,4162362	NA	0,00021617	NA	NA	-0,08904476	8	-19250,9208	38517,8496	681,432904	3,50E-135
3003	-8,35316771	NA	0,03864408	NA	-0,00234345	0,43617682	-0,64441699	-1,91049389	NA	0,01549989	0,00020133	-0,00049532	NA	-0,09486783	11	-19248,2228	38518,4602	682,043496	2,58E-135
3996	-9,61919161	0,46601352	0,06534536	NA	-0,00131078	0,33938341	NA	-2,05206432	NA	0,01786019	0,00020271	-0,00039168	0,51529109	-0,14768431	12	-19247,4506	38518,9185	682,501824	2,05E-135
2930	-8,51824578	0,51124721	NA	NA	NA	0,38535796	-0,65213053	-1,73983563	0,43090563	NA	0,00021643	0,00010896	NA	-0,08710954	10	-19249,4856	38518,9833	682,566611	1,99E-135
2921	-3,98530292	NA	NA	NA	-0,00234183	NA	-0,67333606	-1,73983563	0,43090563	NA	0,00021643	0,00010896	NA	-0,08710954	9	-19250,6311	38519,2722	682,855437	1,72E-136
356	-9,79595589	0,69445228	0,04389505	NA	NA	NA	-0,55018722	-1,97841134	0,41538406	NA	0,00021885	NA	NA	NA	8	-19251,7795	38519,5669	683,150224	1,48E-135
1364	-13,7669049	0,65178191	0,06462958	NA	NA	0,35119819	NA	-2,09152745	0,35956015	NA	0,00022076	NA	0,43622845	NA	9	-19250,8295	38519,6689	683,252161	1,41E-135
1876	-13,7669049	0,65178191	0,06462958	NA	NA	0,35119819	NA	-2,2103983	0,38500536	NA	0,00022113	0,00018745	0,43806177	NA	10	-19249,9554	38519,923	683,506252	1,24E-135
1356	-11,9654714	0,51367836	0,06255183	NA	-0,0013444	NA	NA	-2,09152745	0,35956015	NA	0,00022076	NA	0,43622845	NA	9	-19251,018	38520,046	683,629305	1,17E-135
1826	-7,20552725	0,58574016	NA	NA	NA	NA	-0,62020516	-1,78635429	NA	NA	0,0002119	-0,00029974	0,47515083	NA	8	-19252,0463	38520,1005	683,683809	1,14E-135
828	-5,95790631	0,3741151	0,04613303	NA	-0,00180249	0,39660544	-0,62074526	-1,56810442	NA	NA	0,00020849	-0,0004232	NA	NA	10	-19250,1299	38520,272	683,855294	1,04E-136
1868	-11,9654714	0,51367836	0,06255183	NA	-0,0013444	NA	NA	-2,2103983	0,38500536	NA	0,00022113	0,00018745	0,43806177	NA	10	-19250,144	38520,3001	683,883396	1,03E-135
3425	-8,58193391	NA	NA	NA	NA	NA	-0,61393687	-1,61349146	0,35721774	NA	0,00021669	NA	0,46315983	-0,13737061	8	-19252,2684	38520,5447	684,127971	9,10E-136
868	-9,79595589	0,69445228	0,04389505	NA	NA	NA	-0,55018722	-2,0661368	0,43415925	NA	0,00021911	0,00013912	NA	NA	9	-19251,3016	38520,6131	684,196352	8,80E-136
3937	-8,58193391	NA	NA	NA	NA	NA	-0,61393687	-1,71083893	0,37612338	NA	0,00021703	0,00013898	0,46389079	-0,13469622	9	-19252,7951	38521,6002	685,18346	5,37E-136
2364	-5,95790631	0,3741151	0,04613303	NA	-0,00180249	0,39660544	-0,62074526	-1,50990804	NA	NA	0,00020523	NA	NA	-0,08563427	10	-19251,4322	38522,8767	686,459932	2,84E-136
1314	-7,20552725	0,58574016	NA	NA	NA	NA	-0,62020516	-1,96172815	NA	NA	0,00021146	NA	0,48287384	NA	7	-19254,6512	38523,3085	686,891826	2,29E-136
370	-8,51824578	0,51124721	NA	NA	NA	0,38535796	-0,65213053	-1,97841134	0,41538406	NA	0,00021885	NA	NA	NA	8	-19253,7664	38523,5409	687,12413	2,03E-136
361	-3,98530292	NA	NA	NA	-0,00234183	NA	-0,67333606	-1,97841134	0,41538406	NA	0,00021885	NA	NA	NA	7	-19254,912	38523,8301	687,413398	1,76E-136
882	-8,51824578	0,51124721	NA	NA	NA	0,38535796	-0,65213053	-2,0661368	0,43415925	NA	0,00021911	0,00013912	NA	NA	9	-19253,2885	38524,587	688,170258	1,21E-136
873	-3,98530292	NA	NA	NA	-0,00234183	NA	-0,67333606	-2,0661368	0,43415925	NA	0,00021911	0,00013912	NA	NA	8	-19254,434	38524,876	688,459305	1,04E-136
1969	-6,3315051	NA	NA	NA	NA	0,4838605	-0,6580268	-2,58652225	NA	0,01772679	0,00020696	-0,00034396	0,48679163	NA	9	-19253,4387	38524,8873	688,470613	1,04E-136
1379	-9,52399274	NA	0,02470059	NA	NA	NA	-0,56950148	-2,09152745	0,35956015	NA	0,00022076	NA	0,43622845	NA	8	-19254,6146	38525,2371	688,82038	8,71E-137
1891	-9,52399274	NA	0,02470059	NA	NA	NA	-0,56950148	-2,2103983	0,38500536	NA	0,00022113	0,00018745	0,43806177	NA	9	-19253,7405	38525,491	689,074249	7,67E-137
3484	-9,61919161	0,46601352	0,06534536	NA	-0,00131078	0,33938341	NA	-2,27275384	NA	0,01711404	0,00020255	NA	0,52288611	-0,14000571	11	-19251,7914	38525,5973	689,180606	7,28E-137
955	-8,35316771	NA	0,03864408	NA	-0,00234345	0,43617682	-0,64441699	-2,26386687	NA	0,01548996	0,00020413	-0,00046266	NA	NA	10	-19252,8204	38525,6529	689,236204	7,08E-137
2530	-7,20552725	0,58574016	NA	NA	NA	NA	-0,62020516	-2,55577604	0,44288659	0,01897716	0,00021141	NA	NA	-0,08883356	9	-19253,9278	38525,8656	689,448918	6,36E-137
3042	-7,20552725	0,58574016	NA	NA	NA	NA	-0,62020516	-2,61719209	0,45517288	0,01892094	0,00021164	9,21E-05	NA	-0,08713002	10	-19253,7223	38527,4568	691,040061	2,87E-137
3889	-6,3315051	NA	NA	NA	NA	0,4838605	-0,6580268	-1,25486087	NA	NA	0,00020772	-0,0003441	0,50313521	-0,14558566	9	-19254,7957	38527,6013	691,184628	2,67E-137
1843	-8,8387814	NA	0,03010563	NA	NA	0,51295904	-0,60572616	-1,78635429	NA	NA	0,0002119	-0,00029974	0,47515083	NA	9	-19255,0629	38528,1358	691,719111	2,05E-137
316	-5,95790631	0,3741151	0,04613303	NA	-0,00180249	0,39660544	-0,62074526	-1,812439	NA	NA	0,00020783	NA	NA	NA	9	-19255,2568	38528,5235	692,106742	1,68E-137
1371	-11,3659105	NA	0,05744064	NA	-0,00198557	0,38445946	NA	-2,09152745	0,35956015	NA	0,00022076	NA	0,43622845	NA	9	-19255,4401	38528,8901	692,473384	1,40E-137
1883	-11,3659105	NA	0,05744064	NA	-0,00198557	0,38445946	NA	-2,2103983	0,38500536	NA	0,00022113	0,00018745	0,43806177	NA	10	-19254,566	38529,1442	692,727475	1,24E-137
1457	-6,3315051	NA	NA	NA	NA	0,4838605	-0,6580268	-2,75826159	NA	0,01709531	0,00020664	NA	0,4949792	NA	8	-19256,8271	38529,6623	693,245535	9,53E-138

2491	-8,35316771	NA	0,03864408	NA	-0,00234345	0,43617682	-0,64441699	-2,18775169	NA	0,01458917	0,00020109	NA	NA	-0,08533198	10	-19255,0838	38530,1797	693,762959	7,36E-139
3396	-12,0135881	0,70944129	0,06178489	NA	NA	NA	NA	-1,61349146	0,35721774	NA	0,00021669	NA	0,46315983	-0,13737061	9	-19256,5542	38531,1183	694,701567	4,60E-138
1331	-8,8387814	NA	0,03010563	NA	NA	0,51295904	-0,60572616	-1,96172815	NA	NA	0,00021146	NA	0,48287384	NA	8	-19257,6678	38531,3436	694,926906	4,11E-138
482	-7,20552725	0,58574016	NA	NA	NA	NA	-0,62020516	-2,87228819	0,44207022	0,01904584	0,00021405	NA	NA	NA	8	-19257,8821	38531,7722	695,355441	3,32E-138
3908	-12,0135881	0,70944129	0,06178489	NA	NA	NA	NA	-1,71083893	0,37612338	NA	0,00021703	0,00013898	0,46389079	-0,13469622	10	-19256,0809	38532,174	695,757277	2,72E-139
3377	-6,3315051	NA	NA	NA	NA	0,4838605	-0,6580268	-1,4783871	NA	NA	0,00020739	NA	0,51055393	-0,13912561	8	-19258,1896	38532,3872	695,970485	2,44E-139
1483	-9,45532716	NA	0,05329757	NA	-0,00210061	NA	NA	-3,07214819	0,38813022	0,02082345	0,00021556	NA	0,44803127	NA	9	-19257,2192	38532,4484	696,031684	2,37E-138
2988	-9,35928532	0,43489058	0,04364193	NA	-0,00182965	NA	-0,59565606	-1,91049389	NA	0,01549989	0,00020133	-0,00049532	NA	-0,09486783	11	-19255,4205	38532,8555	696,4388	1,93E-138
1995	-9,45532716	NA	0,05329757	NA	-0,00210061	NA	NA	-3,17508673	0,41103069	0,02071909	0,00021591	0,0001702	0,4495725	NA	10	-19256,5043	38533,0209	696,604142	1,78E-138
994	-7,20552725	0,58574016	NA	NA	NA	NA	-0,62020516	-2,9468992	0,4586198	0,01897353	0,00021429	0,00012376	NA	NA	9	-19257,5067	38533,0234	696,606673	1,78E-138
2547	-8,8387814	NA	0,03010563	NA	NA	0,51295904	-0,60572616	-2,55577604	0,44288659	0,01897716	0,00021141	NA	NA	-0,08883356	10	-19256,9445	38533,9012	697,484441	1,14E-138
2875	-8,35316771	NA	0,03864408	NA	-0,00234345	0,43617682	-0,64441699	-1,2176888	NA	NA	0,00020569	-0,00045432	NA	-0,0941422	10	-19257,4926	38534,9974	698,580629	6,62E-139
3059	-8,8387814	NA	0,03010563	NA	NA	0,51295904	-0,60572616	-2,61719209	0,45517288	0,01892094	0,00021164	9,21E-05	NA	-0,08713002	11	-19256,739	38535,4925	699,075806	5,17E-140
443	-8,35316771	NA	0,03864408	NA	-0,00234345	0,43617682	-0,64441699	-2,49206855	NA	0,01465098	0,00020366	NA	NA	NA	9	-19258,8737	38535,7574	699,340642	4,53E-139
1377	-8,58193391	NA	NA	NA	NA	NA	-0,61393687	-2,09152745	0,35956015	NA	0,00022076	NA	0,43622845	NA	7	-19261,6307	38537,2675	700,850772	2,13E-139
1889	-8,58193391	NA	NA	NA	NA	NA	-0,61393687	-2,2103983	0,38500536	NA	0,00022113	0,00018745	0,43806177	NA	8	-19260,7566	38537,5211	701,104419	1,87E-139
1948	-9,61919161	0,46601352	0,06534536	NA	-0,00131078	0,33938341	NA	-2,58652225	NA	0,01772679	0,00020696	-0,00034396	0,48679163	NA	11	-19258,3964	38538,8074	702,390709	9,85E-140
3002	-6,98837994	0,26427623	NA	NA	-0,00180413	0,37036432	-0,69230587	-1,91049389	NA	0,01549989	0,00020133	-0,00049532	NA	-0,09486783	11	-19258,641	38539,2967	702,879981	7,71E-140
499	-8,8387814	NA	0,03010563	NA	NA	0,51295904	-0,60572616	-2,87228819	0,44207022	0,01904584	0,00021405	NA	NA	NA	9	-19260,8988	38539,8075	703,390742	5,97E-140
940	-9,35928532	0,43489058	0,04364193	NA	-0,00182965	NA	-0,59565606	-2,26386687	NA	0,01548996	0,00020413	-0,00046266	NA	NA	10	-19260,018	38540,0482	703,631508	5,30E-140
1011	-8,8387814	NA	0,03010563	NA	NA	0,51295904	-0,60572616	-2,9468992	0,4586198	0,01897353	0,00021429	0,00012376	NA	NA	10	-19260,5234	38541,0589	704,642197	3,20E-140
3546	-8,97566397	0,33138274	NA	NA	-0,0012458	0,28352203	NA	-2,59012234	0,38593256	0,02081194	0,00021148	NA	0,47514204	-0,138389	11	-19259,6194	38541,2535	704,836732	2,90E-140
3868	-9,61919161	0,46601352	0,06534536	NA	-0,00131078	0,33938341	NA	-1,25486087	NA	NA	0,00020772	-0,0003441	0,50313521	-0,14558566	11	-19259,7534	38541,5214	705,104724	2,54E-140
827	-8,35316771	NA	0,03864408	NA	-0,00234345	0,43617682	-0,64441699	-1,56810442	NA	NA	0,00020849	-0,0004232	NA	NA	9	-19262,0356	38542,0811	705,664394	1,92E-140
4058	-8,97566397	0,33138274	NA	NA	-0,0012458	0,28352203	NA	-2,66976045	0,40191971	0,02073197	0,0002118	0,00011864	0,47565984	-0,13600353	12	-19259,2774	38542,5721	706,155386	1,50E-140
1436	-9,61919161	0,46601352	0,06534536	NA	-0,00131078	0,33938341	NA	-2,75826159	NA	0,01709531	0,00020664	NA	0,4949792	NA	10	-19261,7849	38543,5819	707,165188	9,05E-141
2476	-9,35928532	0,43489058	0,04364193	NA	-0,00182965	NA	-0,59565606	-2,18775169	NA	0,01458917	0,00020109	NA	NA	-0,08533198	10	-19262,2814	38544,575	708,158263	5,51E-141
3988	-13,7669049	0,65178191	0,06462958	NA	NA	0,35119819	NA	-2,05206432	NA	0,01786019	0,00020271	-0,00039168	0,51529109	-0,14768431	11	-19261,333	38544,6805	708,263784	5,23E-141
2363	-8,35316771	NA	0,03864408	NA	-0,00234345	0,43617682	-0,64441699	-1,50990804	NA	NA	0,00020523	NA	NA	-0,08563427	9	-19263,3379	38544,6858	708,269032	5,21E-141
3980	-11,9654714	0,51367836	0,06255183	NA	-0,0013444	NA	NA	-2,05206432	NA	0,01786019	0,00020271	-0,00039168	0,51529109	-0,14768431	11	-19261,5215	38545,0576	708,640928	4,33E-141
2996	-10,737769	0,62231984	0,04626548	NA	NA	0,40997724	-0,57836783	-1,91049389	NA	0,01549989	0,00020133	-0,00049532	NA	-0,09486783	11	-19261,6484	38545,3114	708,894672	3,81E-141
3356	-9,61919161	0,46601352	0,06534536	NA	-0,00131078	0,33938341	NA	-1,4783871	NA	NA	0,00020739	NA	0,51055393	-0,13912561	10	-19263,1473	38546,3069	709,890138	2,32E-141
954	-6,98837994	0,26427623	NA	NA	-0,00180413	0,37036432	-0,69230587	-2,26386687	NA	0,01548996	0,00020413	-0,00046266	NA	NA	10	-19263,2386	38546,4894	710,072689	2,11E-141
3403	-9,45532716	NA	0,05329757	NA	-0,00210061	NA	NA	-1,61349146	0,35721774	NA	0,00021669	NA	0,46315983	-0,13737061	9	-19264,4333	38546,8766	710,459892	1,74E-141
1841	-6,3315051	NA	NA	NA	NA	0,4838605	-0,6580268	-1,78635429	NA	NA	0,0002119	-0,00029974	0,47515083	NA	8	-19265,4775	38546,9629	710,546193	1,67E-143
1348	-12,0135881	0,70944129	0,06178489	NA	NA	NA	NA	-2,09152745	0,35956015	NA	0,00022076	NA	0,43622845	NA	8	-19265,9165	38547,8409	711,424146	1,08E-141
3915	-9,45532716	NA	0,05329757	NA	-0,00210061	NA	NA	-1,71083893	0,37612338	NA	0,00021703	0,00013898	0,46389079	-0,13469622	10	-19263,9601	38547,9323	711,515602	1,03E-141
1860	-12,0135881	0,70944129	0,06178489	NA	NA	NA	NA	-2,2103983	0,38500536	NA	0,00022113	0,00018745	0,43806177	NA	9	-19265,0424	38548,0947	711,678015	9,48E-142
2860	-9,35928532	0,43489058	0,04364193	NA	-0,00182965	NA	-0,59565606	-1,2176888	NA	NA	0,00020569	-0,00045432	NA	-0,0941422	10	-19264,6902	38549,3927	712,975933	4,95E-142
3001	-5,46460783	NA	NA	NA	-0,00220571	0,40342229	-0,70298126	-1,91049389	NA	0,01549989	0,00020133	-0,00049532	NA	-0,09486783	10	-19264,9471	38549,9064	713,489703	3,83E-142
428	-9,35928532	0,43489058	0,04364193	NA	-0,00182965	NA	-0,59565606	-2,49206855	NA	0,01465098	0,00020366	NA	NA	NA	9	-19266,0714	38550,1527	713,735945	3,39E-142
1329	-6,3315051	NA	NA	NA	NA	0,4838605	-0,6580268	-1,96172815	NA	NA	0,00021146	NA	0,48287384	NA	7	-19268,0824	38550,1709	713,754209	3,36E-142
4003	-9,52399274	NA	0,02470059	NA	NA	NA	-0,56950148	-2,05206432	NA	0,01786019	0,00020271	-0,00039168	0,51529109	-0,14768431	10	-19265,1181	38550,2483	713,831559	3,23E-143
315	-8,35316771	NA	0,03864408	NA	-0,00234345	0,43617682	-0,64441699	-1,812439	NA	NA	0,00020783	NA	NA	NA	8	-19267,1624	38550,3328	713,916064	3,10E-142
2490	-6,98837994	0,26427623	NA	NA	-0,00180413	0,37036432	-0,69230587	-2,18775169	NA	0,01458917	0,00020109	NA	NA	-0,08533198	10	-19265,502	38551,0162	714,599444	2,20E-142
3476	-13,7669049	0,65178191	0,06462958	NA	NA	0,35119819	NA	-2,27275384	NA	0,01711404	0,00020255	NA	0,52288611	-0,14000571	10	-19265,6737	38551,3595	714,942788	1,85E-142
2402	-7,20552725	0,58574016	NA	NA	NA	NA	-0,62020516	-1,66433243	0,4162362	NA	0,00021617	NA	NA	-0,08904476	8	-19267,8094	38551,6267	715,209966	1,62E-142
3468	-11,9654714	0,51367836	0,06255183	NA	-0,0013444	NA	NA	-2,27275384	NA	0,01711404	0,00020255	NA	0,52288611	-0,14000571	10	-19265,8622	38551,7367	715,319931	1,53E-143
948	-10,737769	0,62231984	0,04626548	NA	NA	0,40997724	-0,57836783	-2,26386687	NA	0,01548996	0,00020413	-0,00046266	NA	NA	10	-19266,246	38552,5041	716,08738	1,05E-142
2545	-6,3315051	NA	NA	NA	NA	0,4838605	-0,6580268	-2,55577604	0,44288659	0,01897716	0,00021141	NA	NA	-0,08883356	9	-19267,359	38552,728	716,311302	9,35E-144
2914	-7,20552725	0,58574016	NA	NA	NA	NA	-0,62020516	-1,73983563	0,43090563	NA	0,00021643	0,00010896	NA	-0,08710954	9	-19267,5196	38553,0492	716,632499	7,96E-143
3995	-11,3659105	NA	0,05744064	NA	-0,00198557	0,38445946	NA	-2,05206432	NA	0,01786019	0,00020271	-0,00039168	0,51529109	-0,14768431	11	-19265,9436	38553,9017	717,485007	5,20E-143
3057	-6,3315051	NA	NA	NA	NA	0,4838605	-0,6580268	-2,61719209	0,45517288	0,01892094	0,00021164	9,21E-05	NA	-0,08713002	10	-19267,1535	38554,3192	717,902445	4,22E-143
2874	-6,98837994	0,26427623	NA	NA	-0,00180413	0,37036432	-0,69230587	-1,2176888	NA	NA	0,00020569	-0,00045432	NA	-0,0941422	10	-19267,9108	38555,8338	719,417114	1,98E-143
812	-9,35928532	0,43489058	0,04364193	NA	-0,00182965	NA	-0,59565606	-1,56810442	NA	NA	0,00020849	-0,0004232	NA	NA	9	-19269,2332	38556,4764	720,059698	1,43E-143
442	-6,98837994	0,26427623	NA	NA	-0,00180413	0,37036432	-0,69230587	-2,49206855	NA	0,01465098	0,00020366	NA	NA	NA	9	-19269,2919	38556,5938	720,177127	1,35E-143
3491	-9,52399274	NA	0,02470059	NA	NA	NA	-0,56950148	-2,27275384	NA	0,01711404	0,00020255	NA	0,52288611	-0,14000571	9	-19269,4588	38556,9275	720,510784	1,14E-143
2484	-10,737769	0,62231984	0,04626548	NA	NA	0,40997724	-0,57836783	-2,18775169	NA	0,01458917	0,00020109	NA	NA	-0,08533198	10	-19268,5093	38557,0309	720,614135	1,09E-143
953	-5,46460783	NA	NA	NA	-0,00220571	0,40342229	-0,70298126	-2,26386687	NA	0,01548996	0,00020413	-0,00046266	NA	NA	9	-19269,5447	38557,0994	720,682632	1,05E-144
354	-7,20552725	0,58574016	NA	NA	NA	NA	-0,62020516	-1,97841134	0,41538406	NA	0,00021885	NA	NA	NA	7	-19271,8005	38557,6072	721,190461	8,15E-144
1498	-8,97566397	0,33138274	NA	NA	-0,0012458	0,28352203	NA	-3,07214819	0,38813022	0,02082345	0,00021556	NA	0,44803127	NA	10	-19269,0734	38558,159	721,742262	6,18E-145

497	-6,3315051	NA	NA	NA	NA	0,4838605	-0,6580268	-2,87228819	0,44207022	0,01904584	0,00021405	NA	NA	NA	8	-19271,3133	38558,6345	722,217824	4,88E-144
866	-7,20552725	0,58574016	NA	NA	NA	NA	-0,62020516	-2,0661368	0,43415925	NA	0,00021911	0,00013912	NA	NA	8	-19271,3226	38558,6531	722,236367	4,83E-144
2010	-8,97566397	0,33138274	NA	NA	-0,0012458	0,28352203	NA	-3,17508673	0,41103069	0,02071909	0,00021591	0,0001702	0,4495725	NA	11	-19268,3585	38558,7317	722,314941	4,64E-144
3545	-7,52922856	NA	NA	NA	-0,00175017	0,32067571	NA	-2,59012234	0,38593256	0,02081194	0,00021148	NA	0,47514204	-0,138389	10	-19269,4449	38558,902	722,485289	4,27E-144
3530	-7,76791863	0,37580763	NA	NA	-0,00127907	NA	NA	-2,59012234	0,38593256	0,02081194	0,00021148	NA	0,47514204	-0,138389	10	-19269,508	38559,0282	722,611481	4,00E-144
2348	-9,35928532	0,43489058	0,04364193	NA	-0,00182965	NA	-0,59565606	-1,50990804	NA	NA	0,00020523	NA	NA	-0,08563427	9	-19270,5356	38559,0811	722,664336	3,90E-144
2419	-8,8387814	NA	0,03010563	NA	NA	0,51295904	-0,60572616	-1,66433243	0,4162362	NA	0,00021617	NA	NA	-0,08904476	9	-19270,826	38559,662	723,245268	2,92E-144
1009	-6,3315051	NA	NA	NA	NA	0,4838605	-0,6580268	-2,9468992	0,4586198	0,01897353	0,00021429	0,00012376	NA	NA	9	-19270,9379	38559,8858	723,469057	2,61E-145
4057	-7,52922856	NA	NA	NA	-0,00175017	0,32067571	NA	-2,66976045	0,40191971	0,02073197	0,0002118	0,00011864	0,47565984	-0,13600353	11	-19269,1029	38560,2204	723,803721	2,21E-144
4042	-7,76791863	0,37580763	NA	NA	-0,00127907	NA	NA	-2,66976045	0,40191971	0,02073197	0,0002118	0,00011864	0,47565984	-0,13600353	11	-19269,166	38560,3466	723,929913	2,07E-144
3483	-11,3659105	NA	0,05744064	NA	-0,00198557	0,38445946	NA	-2,27275384	NA	0,01711404	0,00020255	NA	0,52288611	-0,14000571	10	-19270,2843	38560,5807	724,16401	1,84E-144
1820	-9,61919161	0,46601352	0,06534536	NA	-0,00131078	0,33938341	NA	-1,78635429	NA	NA	0,0002119	-0,00029974	0,47515083	NA	10	-19270,4352	38560,8826	724,465846	1,58E-144
2931	-8,8387814	NA	0,03010563	NA	NA	0,51295904	-0,60572616	-1,73983563	0,43090563	NA	0,00021643	0,00010896	NA	-0,08710954	10	-19270,5363	38561,0847	724,668022	1,43E-144
2489	-5,46460783	NA	NA	NA	-0,00220571	0,40342229	-0,70298126	-2,18775169	NA	0,01458917	0,00020109	NA	NA	-0,08533198	9	-19271,8081	38561,6261	725,209388	1,09E-144
2868	-10,737769	0,62231984	0,04626548	NA	NA	0,40997724	-0,57836783	-1,2176888	NA	NA	0,00020569	-0,00045432	NA	-0,0941422	10	-19270,9182	38561,8485	725,431805	9,77E-145
4001	-8,58193391	NA	NA	NA	NA	NA	-0,61393687	-2,05206432	NA	0,01786019	0,00020271	-0,00039168	0,51529109	-0,14768431	9	-19272,1341	38562,2782	725,861508	7,88E-145
436	-10,737769	0,62231984	0,04626548	NA	NA	0,40997724	-0,57836783	-2,49206855	NA	0,01465098	0,00020366	NA	NA	NA	9	-19272,2993	38562,6085	726,191818	6,68E-145
826	-6,98837994	0,26427623	NA	NA	-0,00180413	0,37036432	-0,69230587	-1,56810442	NA	NA	0,00020849	-0,0004232	NA	NA	9	-19272,4538	38562,9176	726,500879	5,73E-145
2987	-5,75020926	NA	0,0346068	NA	-0,00247017	NA	-0,61951905	-1,91049389	NA	0,01549989	0,00020133	-0,00049532	NA	-0,09486783	10	-19271,7738	38563,5597	727,142946	4,15E-145
1355	-9,45532716	NA	0,05329757	NA	-0,00210061	NA	NA	-2,09152745	0,35956015	NA	0,00022076	NA	0,43622845	NA	8	-19273,7956	38563,5992	727,182471	4,07E-145
1867	-9,45532716	NA	0,05329757	NA	-0,00210061	NA	NA	-2,2103983	0,38500536	NA	0,00022113	0,00018745	0,43806177	NA	9	-19272,9216	38563,8531	727,43634	3,59E-145
1308	-9,61919161	0,46601352	0,06534536	NA	-0,00131078	0,33938341	NA	-1,96172815	NA	NA	0,00021146	NA	0,48287384	NA	9	-19273,0401	38564,0901	727,673419	3,19E-145
1940	-13,7669049	0,65178191	0,06462958	NA	NA	0,35119819	NA	-2,58652225	NA	0,01772679	0,00020696	-0,00034396	0,48679163	NA	10	-19272,2787	38564,5696	728,152891	2,51E-145
3538	-8,08714822	0,50972296	NA	NA	NA	0,2963004	NA	-2,59012234	0,38593256	0,02081194	0,00021148	NA	0,47514204	-0,138389	10	-19272,3158	38564,6438	728,22705	2,42E-145
300	-9,35928532	0,43489058	0,04364193	NA	-0,00182965	NA	-0,59565606	-1,812439	NA	NA	0,00020783	NA	NA	NA	8	-19274,3601	38564,7281	728,311368	2,32E-145
1932	-11,9654714	0,51367836	0,06255183	NA	-0,0013444	NA	NA	-2,58652225	NA	0,01772679	0,00020696	-0,00034396	0,48679163	NA	10	-19272,4673	38564,9468	728,530034	2,08E-145
3539	-11,2963041	NA	0,04958538	NA	NA	0,45107925	NA	-2,59012234	0,38593256	0,02081194	0,00021148	NA	0,47514204	-0,138389	10	-19272,6915	38565,3952	728,978456	1,66E-145
2362	-6,98837994	0,26427623	NA	NA	-0,00180413	0,37036432	-0,69230587	-1,50990804	NA	NA	0,00020523	NA	NA	-0,08563427	9	-19273,7561	38565,5222	729,105517	1,56E-145
371	-8,8387814	NA	0,03010563	NA	NA	0,51295904	-0,60572616	-1,97841134	0,41538406	NA	0,00021885	NA	NA	NA	8	-19274,8172	38565,6423	729,225541	1,47E-146
4050	-8,08714822	0,50972296	NA	NA	NA	0,2963004	NA	-2,66976045	0,40191971	0,02073197	0,0002118	0,00011864	0,47565984	-0,13600353	11	-19271,9738	38565,9622	729,545483	1,25E-145
2873	-5,46460783	NA	NA	NA	-0,00220571	0,40342229	-0,70298126	-1,2176888	NA	NA	0,00020569	-0,00045432	NA	-0,0941422	9	-19274,2169	38566,4438	730,027058	9,82E-146
2524	-9,61919161	0,46601352	0,06534536	NA	-0,00131078	0,33938341	NA	-2,55577604	0,44288659	0,01897716	0,00021141	NA	NA	-0,08883356	11	-19272,3168	38566,6481	730,231398	8,87E-146
883	-8,8387814	NA	0,03010563	NA	NA	0,51295904	-0,60572616	-2,0661368	0,43415925	NA	0,00021911	0,00013912	NA	NA	9	-19274,3392	38566,6884	730,271669	8,69E-146
4051	-11,2963041	NA	0,04958538	NA	NA	0,45107925	NA	-2,66976045	0,40191971	0,02073197	0,0002118	0,00011864	0,47565984	-0,13600353	11	-19272,3495	38566,7136	730,296888	8,58E-146
441	-5,46460783	NA	NA	NA	-0,00220571	0,40342229	-0,70298126	-2,49206855	NA	0,01465098	0,00020366	NA	NA	NA	8	-19275,598	38567,204	730,787292	6,72E-146
3860	-13,7669049	0,65178191	0,06462958	NA	NA	0,35119819	NA	-1,25486087	NA	NA	0,00020772	-0,0003441	0,50313521	-0,14558566	10	-19273,6357	38567,2836	730,866906	6,45E-146
3852	-11,9654714	0,51367836	0,06255183	NA	-0,0013444	NA	NA	-1,25486087	NA	NA	0,00020772	-0,0003441	0,50313521	-0,14558566	10	-19273,8243	38567,6608	731,244049	5,35E-146
3036	-9,61919161	0,46601352	0,06534536	NA	-0,00131078	0,33938341	NA	-2,61719209	0,45517288	0,01892094	0,00021164	9,21E-05	NA	-0,08713002	12	-19272,1112	38568,2397	731,822984	4,00E-146
820	-10,737769	0,62231984	0,04626548	NA	NA	0,40997724	-0,57836783	-1,56810442	NA	NA	0,00020849	-0,0004232	NA	NA	9	-19275,4612	38568,9323	732,51557	2,83E-146
3489	-8,58193391	NA	NA	NA	NA	NA	-0,61393687	-2,27275384	NA	0,01711404	0,00020255	NA	0,52288611	-0,14000571	8	-19276,4749	38568,9577	732,540955	2,79E-146
1428	-13,7669049	0,65178191	0,06462958	NA	NA	0,35119819	NA	-2,75826159	NA	0,01709531	0,00020664	NA	0,4949792	NA	9	-19275,6672	38569,3443	732,927592	2,30E-146
1420	-11,9654714	0,51367836	0,06255183	NA	-0,0013444	NA	NA	-2,75826159	NA	0,01709531	0,00020664	NA	0,4949792	NA	9	-19275,8558	38569,7215	733,304736	1,91E-146
1955	-9,52399274	NA	0,02470059	NA	NA	NA	-0,56950148	-2,58652225	NA	0,01772679	0,00020696	-0,00034396	0,48679163	NA	9	-19276,0638	38570,1376	733,720888	1,55E-146
2986	-6,22256645	0,32760888	NA	NA	-0,00183217	NA	-0,66370241	-1,91049389	NA	0,01549989	0,00020133	-0,00049532	NA	-0,09486783	10	-19275,3223	38570,6567	734,240007	1,20E-146
939	-5,75020926	NA	0,0346068	NA	-0,00247017	NA	-0,61951905	-2,26386687	NA	0,01548996	0,00020413	-0,00046266	NA	NA	9	-19276,3713	38570,7526	734,335876	1,14E-147
314	-6,98837994	0,26427623	NA	NA	-0,00180413	0,37036432	-0,69230587	-1,812439	NA	NA	0,00020783	NA	NA	NA	8	-19277,5807	38571,1693	734,752549	9,25E-147
2356	-10,737769	0,62231984	0,04626548	NA	NA	0,40997724	-0,57836783	-1,50990804	NA	NA	0,00020523	NA	NA	-0,08563427	9	-19276,7635	38571,5369	735,120208	7,70E-147
3348	-13,7669049	0,65178191	0,06462958	NA	NA	0,35119819	NA	-1,4783871	NA	NA	0,00020739	NA	0,51055393	-0,13912561	9	-19277,0297	38572,0693	735,652542	5,90E-147
3340	-11,9654714	0,51367836	0,06255183	NA	-0,0013444	NA	NA	-1,4783871	NA	NA	0,00020739	NA	0,51055393	-0,13912561	9	-19277,2182	38572,4464	736,029685	4,88E-147
476	-9,61919161	0,46601352	0,06534536	NA	-0,00131078	0,33938341	NA	-2,87228819	0,44207022	0,01904584	0,00021405	NA	NA	NA	10	-19276,271	38572,5542	736,137477	4,63E-147
3418	-8,97566397	0,33138274	NA	NA	-0,0012458	0,28352203	NA	-1,61349146	0,35721774	NA	0,00021669	NA	0,46315983	-0,13737061	10	-19276,2875	38572,5872	736,170469	4,55E-147
3875	-9,52399274	NA	0,02470059	NA	NA	NA	-0,56950148	-1,25486087	NA	NA	0,00020772	-0,0003441	0,50313521	-0,14558566	9	-19277,4208	38572,8516	736,434902	3,99E-147
3972	-12,0135881	0,70944129	0,06178489	NA	NA	NA	NA	-2,05206432	NA	0,01786019	0,00020271	-0,00039168	0,51529109	-0,14768431	10	-19276,4199	38572,852	736,435325	3,99E-147
825	-5,46460783	NA	NA	NA	-0,00220571	0,40342229	-0,70298126	-1,56810442	NA	NA	0,00020849	-0,0004232	NA	NA	8	-19278,7599	38573,5278	737,111044	2,84E-147
3930	-8,97566397	0,33138274	NA	NA	-0,0012458	0,28352203	NA	-1,71083893	0,37612338	NA	0,00021703	0,00013898	0,46389079	-0,13469622	11	-19275,8143	38573,6431	737,226402	2,68E-147
1947	-11,3659105	NA	0,05744064	NA	-0,00198557	0,38445946	NA	-2,58652225	NA	0,01772679	0,00020696	-0,00034396	0,48679163	NA	10	-19276,8893	38573,7908	737,374114	2,49E-147
988	-9,61919161	0,46601352	0,06534536	NA	-0,00131078	0,33938341	NA	-2,9468992	0,4586198	0,01897353	0,00021429	0,00012376	NA	NA	11	-19275,8956	38573,8059	737,389153	2,48E-149
1443	-9,52399274	NA	0,02470059	NA	NA	NA	-0,56950148	-2,75826159	NA	0,01709531	0,00020664	NA	0,4949792	NA	8	-19279,4523	38574,9125	738,49581	1,42E-147
2475	-5,75020926	NA	0,0346068	NA	-0,00247017	NA	-0,61951905	-2,18775169	NA	0,01458917	0,00020109	NA	NA	-0,08533198	9	-19278,6347	38575,2794	738,862631	1,18E-147
1497	-7,52922856	NA	NA	NA	-0,00175017	0,32067571	NA	-3,07214819	0,38813022	0,02082345	0,00021556	NA	0,44803127	NA	9	-19278,8989	38575,8078	739,39104	9,10E-148
1482	-7,76791863	0,37580763	NA	NA	-0,00127907	NA	NA	-3,07214819	0,38813022	0,02082345	0,00021556	NA	0,44803127	NA	9	-19278,962	38575,934	739,517232	8,54E-148

2361	-5,46460783	NA	NA	NA	-0,00220571	0,40342229	-0,70298126	-1,50990804	NA	NA	0,00020523	NA	NA	-0,08563427	8	-19280,0622	38576,1324	739,715682	7,73E-148
2009	-7,52922856	NA	NA	NA	-0,00175017	0,32067571	NA	-3,17508673	0,41103069	0,02071909	0,00021591	0,0001702	0,4495725	NA	10	-19278,184	38576,3802	739,963498	6,83E-148
3867	-11,3659105	NA	0,05744064	NA	-0,00198557	0,38445946	NA	-1,25486087	NA	NA	0,00020772	-0,0003441	0,50313521	-0,14558566	10	-19278,2463	38576,5049	740,088129	6,42E-148
1994	-7,76791863	0,37580763	NA	NA	-0,00127907	NA	NA	-3,17508673	0,41103069	0,02071909	0,00021591	0,0001702	0,4495725	NA	10	-19278,2471	38576,5064	740,08969	6,41E-150
308	-10,737769	0,62231984	0,04626548	NA	NA	0,40997724	-0,57836783	-1,812439	NA	NA	0,00020783	NA	NA	NA	8	-19280,588	38577,184	740,76724	4,57E-148
3363	-9,52399274	NA	0,02470059	NA	NA	NA	-0,56950148	-1,4783871	NA	NA	0,00020739	NA	0,51055393	-0,13912561	8	-19280,8148	38577,6375	741,22076	3,64E-148
938	-6,22256645	0,32760888	NA	NA	-0,00183217	NA	-0,66370241	-2,26386687	NA	0,01548996	0,00020413	-0,00046266	NA	NA	9	-19279,9199	38577,8497	741,432937	3,28E-148
2417	-6,3315051	NA	NA	NA	NA	0,4838605	-0,6580268	-1,66433243	0,4162362	NA	0,00021617	NA	NA	-0,08904476	8	-19281,2406	38578,4891	742,072349	2,38E-148
1435	-11,3659105	NA	0,05744064	NA	-0,00198557	0,38445946	NA	-2,75826159	NA	0,01709531	0,00020664	NA	0,4949792	NA	9	-19280,2778	38578,5655	742,148815	2,29E-148
3460	-12,0135881	0,70944129	0,06178489	NA	NA	NA	NA	-2,27275384	NA	0,01711404	0,00020255	NA	0,52288611	-0,14000571	9	-19280,7607	38579,5313	743,11455	1,41E-148
2929	-6,3315051	NA	NA	NA	NA	0,4838605	-0,6580268	-1,73983563	0,43090563	NA	0,00021643	0,00010896	NA	-0,08710954	9	-19280,9508	38579,9116	743,494882	1,17E-148
2859	-5,75020926	NA	0,0346068	NA	-0,00247017	NA	-0,61951905	-1,2176888	NA	NA	0,00020569	-0,00045432	NA	-0,0941422	9	-19281,0435	38580,097	743,680301	1,07E-148
427	-5,75020926	NA	0,0346068	NA	-0,00247017	NA	-0,61951905	-2,49206855	NA	0,01465098	0,00020366	NA	NA	NA	8	-19282,4246	38580,8573	744,440535	7,28E-149
3355	-11,3659105	NA	0,05744064	NA	-0,00198557	0,38445946	NA	-1,4783871	NA	NA	0,00020739	NA	0,51055393	-0,13912561	9	-19281,6403	38581,2905	744,873765	5,87E-150
1490	-8,08714822	0,50972296	NA	NA	NA	0,2963004	NA	-3,07214819	0,38813022	0,02082345	0,00021556	NA	0,44803127	NA	9	-19281,7698	38581,5495	745,132801	5,15E-149
313	-5,46460783	NA	NA	NA	-0,00220571	0,40342229	-0,70298126	-1,812439	NA	NA	0,00020783	NA	NA	NA	7	-19283,8867	38581,7797	745,362935	4,59E-151
2002	-8,08714822	0,50972296	NA	NA	NA	0,2963004	NA	-3,17508673	0,41103069	0,02071909	0,00021591	0,0001702	0,4495725	NA	10	-19281,0549	38582,122	745,705259	3,87E-149
1953	-8,58193391	NA	NA	NA	NA	NA	-0,61393687	-2,58652225	NA	0,01772679	0,00020696	-0,00034396	0,48679163	NA	8	-19283,0799	38582,1678	745,751058	3,78E-149
1491	-11,2963041	NA	0,04958538	NA	NA	0,45107925	NA	-3,07214819	0,38813022	0,02082345	0,00021556	NA	0,44803127	NA	9	-19282,1455	38582,3009	745,884207	3,54E-149
2474	-6,22256645	0,32760888	NA	NA	-0,00183217	NA	-0,66370241	-2,18775169	NA	0,01458917	0,00020109	NA	NA	-0,08533198	9	-19282,1832	38582,3764	745,959692	3,41E-149
3529	-6,18359995	NA	NA	NA	-0,00186372	NA	NA	-2,59012234	0,38593256	0,02081194	0,00021148	NA	0,47514204	-0,138389	9	-19282,3589	38582,7278	746,31112	2,81E-149
2003	-11,2963041	NA	0,04958538	NA	NA	0,45107925	NA	-3,17508673	0,41103069	0,02071909	0,00021591	0,0001702	0,4495725	NA	10	-19281,4306	38582,8734	746,456665	2,66E-149
4041	-6,18359995	NA	NA	NA	-0,00186372	NA	NA	-2,66976045	0,40191971	0,02073197	0,0002118	0,00011864	0,47565984	-0,13600353	10	-19282,0169	38584,0461	747,62933	1,48E-149
2980	-9,79595589	0,69445228	0,04389505	NA	NA	NA	-0,55018722	-1,91049389	NA	0,01549989	0,00020133	-0,00049532	NA	-0,09486783	10	-19282,0609	38584,134	747,717296	1,42E-149
3522	-10,126072	0,56333515	NA	NA	NA	NA	NA	-2,59012234	0,38593256	0,02081194	0,00021148	NA	0,47514204	-0,138389	9	-19283,1114	38584,2327	747,816017	1,35E-150
369	-6,3315051	NA	NA	NA	NA	0,4838605	-0,6580268	-1,97841134	0,41538406	NA	0,00021885	NA	NA	NA	7	-19285,2317	38584,4696	748,052844	1,20E-149
3873	-8,58193391	NA	NA	NA	NA	NA	-0,61393687	-1,25486087	NA	NA	0,00020772	-0,0003441	0,50313521	-0,14558566	8	-19284,4369	38584,8818	748,465073	9,74E-150
881	-6,3315051	NA	NA	NA	NA	0,4838605	-0,6580268	-2,0661368	0,43415925	NA	0,00021911	0,00013912	NA	NA	8	-19284,7538	38585,5155	749,09875	7,09E-150
4034	-10,126072	0,56333515	NA	NA	NA	NA	NA	-2,66976045	0,40191971	0,02073197	0,0002118	0,00011864	0,47565984	-0,13600353	10	-19282,7694	38585,5509	749,134228	6,97E-150
1812	-13,7669049	0,65178191	0,06462958	NA	NA	0,35119819	NA	-1,78635429	NA	NA	0,0002119	-0,00029974	0,47515083	NA	9	-19284,3175	38586,645	750,22825	4,03E-150
1441	-8,58193391	NA	NA	NA	NA	NA	-0,61393687	-2,75826159	NA	0,01709531	0,00020664	NA	0,4949792	NA	7	-19286,4684	38586,9429	750,526202	3,47E-150
1804	-11,9654714	0,51367836	0,06255183	NA	-0,0013444	NA	NA	-1,78635429	NA	NA	0,0002119	-0,00029974	0,47515083	NA	9	-19284,5061	38587,0221	750,605393	3,34E-150
811	-5,75020926	NA	0,0346068	NA	-0,00247017	NA	-0,61951905	-1,56810442	NA	NA	0,00020849	-0,0004232	NA	NA	8	-19285,5865	38587,181	750,764287	3,08E-151
2858	-6,22256645	0,32760888	NA	NA	-0,00183217	NA	-0,66370241	-1,2176888	NA	NA	0,00020569	-0,00045432	NA	-0,0941422	9	-19284,5921	38587,1941	750,777362	3,06E-150
426	-6,22256645	0,32760888	NA	NA	-0,00183217	NA	-0,66370241	-2,49206855	NA	0,01465098	0,00020366	NA	NA	NA	8	-19285,9732	38587,9543	751,537596	2,10E-150
2994	-8,51824578	0,51124721	NA	NA	NA	0,38535796	-0,65213053	-1,91049389	NA	0,01549989	0,00020133	-0,00049532	NA	-0,09486783	10	-19284,0479	38588,1079	751,691201	1,94E-150
2985	-3,98530292	NA	NA	NA	-0,00234183	NA	-0,67333606	-1,91049389	NA	0,01549989	0,00020133	-0,00049532	NA	-0,09486783	9	-19285,1934	38588,3967	751,980027	1,68E-150
3979	-9,45532716	NA	0,05329757	NA	-0,00210061	NA	NA	-2,05206432	NA	0,01786019	0,00020271	-0,00039168	0,51529109	-0,14768431	10	-19284,2991	38588,6104	752,19365	1,51E-150
1370	-8,97566397	0,33138274	NA	NA	-0,0012458	0,28352203	NA	-2,09152745	0,35956015	NA	0,00022076	NA	0,43622845	NA	9	-19285,6498	38589,3095	752,892827	1,06E-150
1882	-8,97566397	0,33138274	NA	NA	-0,0012458	0,28352203	NA	-2,2103983	0,38500536	NA	0,00022113	0,00018745	0,43806177	NA	10	-19284,7757	38589,5636	753,146917	9,37E-151
3361	-8,58193391	NA	NA	NA	NA	NA	-0,61393687	-1,4783871	NA	NA	0,00020739	NA	0,51055393	-0,13912561	7	-19287,8308	38589,6679	753,251152	8,90E-151
2347	-5,75020926	NA	0,0346068	NA	-0,00247017	NA	-0,61951905	-1,50990804	NA	NA	0,00020523	NA	NA	-0,08563427	8	-19286,8888	38589,7856	753,368925	8,39E-151
1300	-13,7669049	0,65178191	0,06462958	NA	NA	0,35119819	NA	-1,96172815	NA	NA	0,00021146	NA	0,48287384	NA	8	-19286,9224	38589,8528	753,436045	8,11E-151
1292	-11,9654714	0,51367836	0,06255183	NA	-0,0013444	NA	NA	-1,96172815	NA	NA	0,00021146	NA	0,48287384	NA	8	-19287,111	38590,2299	753,813188	6,72E-151
3417	-7,52922856	NA	NA	NA	-0,00175017	0,32067571	NA	-1,61349146	0,35721774	NA	0,00021669	NA	0,46315983	-0,13737061	9	-19286,113	38590,236	753,819247	6,70E-151
3402	-7,76791863	0,37580763	NA	NA	-0,00127907	NA	NA	-1,61349146	0,35721774	NA	0,00021669	NA	0,46315983	-0,13737061	9	-19286,1761	38590,3622	753,945439	6,29E-152
3929	-7,52922856	NA	NA	NA	-0,00175017	0,32067571	NA	-1,71083893	0,37612338	NA	0,00021703	0,00013898	0,46389079	-0,13469622	10	-19285,6398	38591,2917	754,874958	3,95E-151
932	-9,79595589	0,69445228	0,04389505	NA	NA	NA	-0,55018722	-2,26386687	NA	0,01548996	0,00020413	-0,00046266	NA	NA	9	-19286,6585	38591,3269	754,910225	3,88E-152
3914	-7,76791863	0,37580763	NA	NA	-0,00127907	NA	NA	-1,71083893	0,37612338	NA	0,00021703	0,00013898	0,46389079	-0,13469622	10	-19285,7029	38591,4179	755,00115	3,71E-151
1827	-9,52399274	NA	0,02470059	NA	NA	NA	-0,56950148	-1,78635429	NA	NA	0,0002119	-0,00029974	0,47515083	NA	8	-19288,1026	38592,2132	755,796468	2,49E-151
2396	-9,61919161	0,46601352	0,06534536	NA	-0,00131078	0,33938341	NA	-1,66433243	0,4162362	NA	0,00021617	NA	NA	-0,08904476	10	-19286,1983	38592,4087	755,992003	2,26E-151
2516	-13,7669049	0,65178191	0,06462958	NA	NA	0,35119819	NA	-2,55577604	0,44288659	0,01897716	0,00021141	NA	NA	-0,08883356	10	-19286,1991	38592,4103	755,99358	2,26E-151
1924	-12,0135881	0,70944129	0,06178489	NA	NA	NA	NA	-2,58652225	NA	0,01772679	0,00020696	-0,00034396	0,48679163	NA	9	-19287,3657	38592,7414	756,324654	1,91E-151
2508	-11,9654714	0,51367836	0,06255183	NA	-0,0013444	NA	NA	-2,55577604	0,44288659	0,01897716	0,00021141	NA	NA	-0,08883356	10	-19286,3876	38592,7874	756,370724	1,87E-152
2908	-9,61919161	0,46601352	0,06534536	NA	-0,00131078	0,33938341	NA	-1,73983563	0,43090563	NA	0,00021643	0,00010896	NA	-0,08710954	11	-19285,9085	38593,8317	757,414979	1,11E-151
3028	-13,7669049	0,65178191	0,06462958	NA	NA	0,35119819	NA	-2,61719209	0,45517288	0,01892094	0,00021164	9,21E-05	NA	-0,08713002	11	-19285,9935	38594,0017	757,584945	1,02E-151
810	-6,22256645	0,32760888	NA	NA	-0,00183217	NA	-0,66370241	-1,56810442	NA	NA	0,00020849	-0,0004232	NA	NA	8	-19289,1351	38594,2781	757,861348	8,87E-152
3020	-11,9654714	0,51367836	0,06255183	NA	-0,0013444	NA	NA	-2,61719209	0,45517288	0,01892094	0,00021164	9,21E-05	NA	-0,08713002	11	-19286,1821	38594,3788	757,962088	8,44E-153
3467	-9,45532716	NA	0,05329757	NA	-0,00210061	NA	NA	-2,27275384	NA	0,01711404	0,00020255	NA	0,52288611	-0,14000571	9	-19288,6398	38595,2896	758,872875	5,35E-152
946	-8,51824578	0,51124721	NA	NA	NA	0,38535796	-0,65213053	-2,26386687	NA	0,01548996	0,00020413	-0,00046266	NA	NA	9	-19288,6454	38595,3008	758,884131	5,32E-152
1315	-9,52399274	NA	0,02470059	NA	NA	NA	-0,56950148	-1,96172815	NA	NA	0,00021146	NA	0,48287384	NA	7	-19290,7075	38595,4212	759,004484	5,01E-152

299	-5,75020926	NA	0,0346068	NA	-0,00247017	NA	-0,61951905	-1,812439	NA	NA	0,00020783	NA	NA	NA	7	-19290,7134	38595,4329	759,016179	4,98E-152
3844	-12,0135881	0,70944129	0,06178489	NA	NA	NA	NA	-1,25486087	NA	NA	0,00020772	-0,0003441	0,50313521	-0,14558566	9	-19288,7227	38595,4554	759,038669	4,93E-152
937	-3,98530292	NA	NA	NA	-0,00234183	NA	-0,67333606	-2,26386687	NA	0,01548996	0,00020413	-0,00046266	NA	NA	8	-19289,791	38595,5899	759,173178	4,61E-152
2468	-9,79595589	0,69445228	0,04389505	NA	NA	NA	-0,55018722	-2,18775169	NA	0,01458917	0,00020109	NA	NA	-0,08533198	9	-19288,9219	38595,8537	759,436981	4,04E-152
1819	-11,3659105	NA	0,05744064	NA	-0,00198557	0,38445946	NA	-1,78635429	NA	NA	0,0002119	-0,00029974	0,47515083	NA	9	-19288,9281	38595,8662	759,449473	4,01E-152
3410	-8,08714822	0,50972296	NA	NA	NA	0,2963004	NA	-1,61349146	0,35721774	NA	0,00021669	NA	0,46315983	-0,13737061	9	-19288,9839	38595,9777	759,561009	3,79E-152
3411	-11,2963041	NA	0,04958538	NA	NA	0,45107925	NA	-1,61349146	0,35721774	NA	0,00021669	NA	0,46315983	-0,13737061	9	-19289,3596	38596,7291	760,312414	2,61E-152
2346	-6,22256645	0,32760888	NA	NA	-0,00183217	NA	-0,66370241	-1,50990804	NA	NA	0,00020523	NA	NA	-0,08563427	8	-19290,4374	38596,8827	760,465987	2,41E-152
3922	-8,08714822	0,50972296	NA	NA	NA	0,2963004	NA	-1,71083893	0,37612338	NA	0,00021703	0,00013898	0,46389079	-0,13469622	10	-19288,5106	38597,0334	760,61672	2,24E-152
1412	-12,0135881	0,70944129	0,06178489	NA	NA	NA	NA	-2,75826159	NA	0,01709531	0,00020664	NA	0,4949792	NA	8	-19290,7542	38597,5163	761,099576	1,76E-152
3923	-11,2963041	NA	0,04958538	NA	NA	0,45107925	NA	-1,71083893	0,37612338	NA	0,00021703	0,00013898	0,46389079	-0,13469622	10	-19288,8863	38597,7848	761,368125	1,54E-152
2531	-9,52399274	NA	0,02470059	NA	NA	NA	-0,56950148	-2,55577604	0,44288659	0,01897716	0,00021141	NA	NA	-0,08883356	9	-19289,9842	38597,9783	761,561577	1,40E-152
468	-13,7669049	0,65178191	0,06462958	NA	NA	0,35119819	NA	-2,87228819	0,44207022	0,01904584	0,00021405	NA	NA	NA	9	-19290,1533	38598,3166	761,899881	1,18E-152
348	-9,61919161	0,46601352	0,06534536	NA	-0,00131078	0,33938341	NA	-1,97841134	0,41538406	NA	0,00021885	NA	NA	NA	9	-19290,1894	38598,3888	761,972054	1,14E-152
460	-11,9654714	0,51367836	0,06255183	NA	-0,0013444	NA	NA	-2,87228819	0,44207022	0,01904584	0,00021405	NA	NA	NA	9	-19290,3419	38598,6937	762,277024	9,76E-153
1307	-11,3659105	NA	0,05744064	NA	-0,00198557	0,38445946	NA	-1,96172815	NA	NA	0,00021146	NA	0,48287384	NA	8	-19291,533	38599,074	762,657268	8,07E-154
860	-9,61919161	0,46601352	0,06534536	NA	-0,00131078	0,33938341	NA	-2,0661368	0,43415925	NA	0,00021911	0,00013912	NA	NA	10	-19289,7115	38599,4351	763,018404	6,73E-153
980	-13,7669049	0,65178191	0,06462958	NA	NA	0,35119819	NA	-2,9468992	0,4586198	0,01897353	0,00021429	0,00012376	NA	NA	10	-19289,7779	38599,5681	763,151335	6,30E-153
3043	-9,52399274	NA	0,02470059	NA	NA	NA	-0,56950148	-2,61719209	0,45517288	0,01892094	0,00021164	9,21E-05	NA	-0,08713002	10	-19289,7786	38599,5694	763,15272	6,30E-153
1481	-6,18359995	NA	NA	NA	-0,00186372	NA	NA	-3,07214819	0,38813022	0,02082345	0,00021556	NA	0,44803127	NA	8	-19291,8129	38599,6338	763,217092	6,10E-153
2482	-8,51824578	0,51124721	NA	NA	NA	0,38535796	-0,65213053	-2,18775169	NA	0,01458917	0,00020109	NA	NA	-0,08533198	9	-19290,9088	38599,8276	763,410886	5,53E-153
972	-11,9654714	0,51367836	0,06255183	NA	-0,0013444	NA	NA	-2,9468992	0,4586198	0,01897353	0,00021429	0,00012376	NA	NA	10	-19289,9665	38599,9452	763,528479	5,22E-153
2473	-3,98530292	NA	NA	NA	-0,00234183	NA	-0,67333606	-2,18775169	NA	0,01458917	0,00020109	NA	NA	-0,08533198	8	-19292,0543	38600,1167	763,699933	4,79E-153
1993	-6,18359995	NA	NA	NA	-0,00186372	NA	NA	-3,17508673	0,41103069	0,02071909	0,00021591	0,0001702	0,4495725	NA	9	-19291,098	38600,2061	763,789329	4,58E-153
3332	-12,0135881	0,70944129	0,06178489	NA	NA	NA	NA	-1,4783871	NA	NA	0,00020739	NA	0,51055393	-0,13912561	8	-19292,1166	38600,2412	763,824526	4,50E-153
2852	-9,79595589	0,69445228	0,04389505	NA	NA	NA	-0,55018722	-1,2176888	NA	NA	0,00020569	-0,00045432	NA	-0,0941422	9	-19291,3307	38600,6714	764,254651	3,63E-154
1474	-10,126072	0,56333515	NA	NA	NA	NA	NA	-3,07214819	0,38813022	0,02082345	0,00021556	NA	0,44803127	NA	8	-19292,5654	38601,1387	764,72199	2,87E-153
420	-9,79595589	0,69445228	0,04389505	NA	NA	NA	-0,55018722	-2,49206855	NA	0,01465098	0,00020366	NA	NA	NA	8	-19292,7118	38601,4316	765,014884	2,48E-154
2523	-11,3659105	NA	0,05744064	NA	-0,00198557	0,38445946	NA	-2,55577604	0,44288659	0,01897716	0,00021141	NA	NA	-0,08883356	10	-19290,8097	38601,6315	765,214803	2,25E-153
1986	-10,126072	0,56333515	NA	NA	NA	NA	NA	-3,17508673	0,41103069	0,02071909	0,00021591	0,0001702	0,4495725	NA	9	-19291,8505	38601,7109	765,294226	2,16E-153
298	-6,22256645	0,32760888	NA	NA	-0,00183217	NA	-0,66370241	-1,812439	NA	NA	0,00020783	NA	NA	NA	7	-19294,2619	38602,53	766,11324	1,43E-153
3035	-11,3659105	NA	0,05744064	NA	-0,00198557	0,38445946	NA	-2,61719209	0,45517288	0,01892094	0,00021164	9,21E-05	NA	-0,08713002	11	-19290,6041	38603,2229	766,806168	1,01E-153
483	-9,52399274	NA	0,02470059	NA	NA	NA	-0,56950148	-2,87228819	0,44207022	0,01904584	0,00021405	NA	NA	NA	8	-19293,9384	38603,8848	767,468099	7,28E-154
1825	-8,58193391	NA	NA	NA	NA	NA	-0,61393687	-1,78635429	NA	NA	0,0002119	-0,00029974	0,47515083	NA	7	-19295,1187	38604,2436	767,82686	6,08E-154
2866	-8,51824578	0,51124721	NA	NA	NA	0,38535796	-0,65213053	-1,2176888	NA	NA	0,00020569	-0,00045432	NA	-0,0941422	9	-19293,3177	38604,6453	768,228556	4,98E-154
2857	-3,98530292	NA	NA	NA	-0,00234183	NA	-0,67333606	-1,2176888	NA	NA	0,00020569	-0,00045432	NA	-0,0941422	8	-19294,4632	38604,9343	768,517603	4,31E-154
995	-9,52399274	NA	0,02470059	NA	NA	NA	-0,56950148	-2,9468992	0,4586198	0,01897353	0,00021429	0,00012376	NA	NA	9	-19293,5631	38605,1361	768,719332	3,89E-154
434	-8,51824578	0,51124721	NA	NA	NA	0,38535796	-0,65213053	-2,49206855	NA	0,01465098	0,00020366	NA	NA	NA	8	-19294,6988	38605,4055	768,98879	3,40E-154
425	-3,98530292	NA	NA	NA	-0,00234183	NA	-0,67333606	-2,49206855	NA	0,01465098	0,00020366	NA	NA	NA	7	-19295,8443	38605,6948	769,278059	2,94E-155
1369	-7,52922856	NA	NA	NA	-0,00175017	0,32067571	NA	-2,09152745	0,35956015	NA	0,00022076	NA	0,43622845	NA	8	-19295,4753	38606,9585	770,541826	1,57E-156
1354	-7,76791863	0,37580763	NA	NA	-0,00127907	NA	NA	-2,09152745	0,35956015	NA	0,00022076	NA	0,43622845	NA	8	-19295,5384	38607,0847	770,668018	1,47E-154
1881	-7,52922856	NA	NA	NA	-0,00175017	0,32067571	NA	-2,2103983	0,38500536	NA	0,00022113	0,00018745	0,43806177	NA	9	-19294,6012	38607,2124	770,795696	1,38E-154
1866	-7,76791863	0,37580763	NA	NA	-0,00127907	NA	NA	-2,2103983	0,38500536	NA	0,00022113	0,00018745	0,43806177	NA	9	-19294,6643	38607,3386	770,921887	1,29E-154
1313	-8,58193391	NA	NA	NA	NA	NA	-0,61393687	-1,96172815	NA	NA	0,00021146	NA	0,48287384	NA	6	-19297,7236	38607,4518	771,035098	1,22E-154
475	-11,3659105	NA	0,05744064	NA	-0,00198557	0,38445946	NA	-2,87228819	0,44207022	0,01904584	0,00021405	NA	NA	NA	9	-19294,7639	38607,5378	771,121104	1,17E-154
804	-9,79595589	0,69445228	0,04389505	NA	NA	NA	-0,55018722	-1,56810442	NA	NA	0,00020849	-0,0004232	NA	NA	8	-19295,8737	38607,7554	771,338637	1,05E-154
1931	-9,45532716	NA	0,05329757	NA	-0,00210061	NA	NA	-2,58652225	NA	0,01772679	0,00020696	-0,00034396	0,48679163	NA	9	-19295,2449	38608,4997	772,082979	7,24E-155
987	-11,3659105	NA	0,05744064	NA	-0,00198557	0,38445946	NA	-2,9468992	0,4586198	0,01897353	0,00021429	0,00012376	NA	NA	10	-19294,3886	38608,7893	772,372558	6,27E-156
2529	-8,58193391	NA	NA	NA	NA	NA	-0,61393687	-2,55577604	0,44288659	0,01897716	0,00021141	NA	NA	-0,08883356	8	-19297,0003	38610,0085	773,591747	3,41E-155
2340	-9,79595589	0,69445228	0,04389505	NA	NA	NA	-0,55018722	-1,50990804	NA	NA	0,00020523	NA	NA	-0,08563427	8	-19297,176	38610,36	773,943275	2,86E-155
3851	-9,45532716	NA	0,05329757	NA	-0,00210061	NA	NA	-1,25486087	NA	NA	0,00020772	-0,0003441	0,50313521	-0,14558566	9	-19296,6019	38611,2137	774,796993	1,86E-155
3041	-8,58193391	NA	NA	NA	NA	NA	-0,61393687	-2,61719209	0,45517288	0,01892094	0,00021164	9,21E-05	NA	-0,08713002	9	-19296,7947	38611,5994	775,182669	1,54E-155
818	-8,51824578	0,51124721	NA	NA	NA	0,38535796	-0,65213053	-1,56810442	NA	NA	0,00020849	-0,0004232	NA	NA	8	-19297,8607	38611,7293	775,312543	1,44E-155
809	-3,98530292	NA	NA	NA	-0,00234183	NA	-0,67333606	-1,56810442	NA	NA	0,00020849	-0,0004232	NA	NA	7	-19299,0062	38612,0185	775,601811	1,25E-155
1362	-8,08714822	0,50972296	NA	NA	NA	0,2963004	NA	-2,09152745	0,35956015	NA	0,00022076	NA	0,43622845	NA	8	-19298,3462	38612,7003	776,283588	8,87E-156
1874	-8,08714822	0,50972296	NA	NA	NA	0,2963004	NA	-2,2103983	0,38500536	NA	0,00022113	0,00018745	0,43806177	NA	9	-19297,4721	38612,9542	776,537457	7,81E-157
1419	-9,45532716	NA	0,05329757	NA	-0,00210061	NA	NA	-2,75826159	NA	0,01709531	0,00020664	NA	0,4949792	NA	8	-19298,6333	38613,2746	776,857901	6,65E-156
1363	-11,2963041	NA	0,04958538	NA	NA	0,45107925	NA	-2,09152745	0,35956015	NA	0,00022076	NA	0,43622845	NA	8	-19298,7219	38613,4517	777,034993	6,09E-157
1875	-11,2963041	NA	0,04958538	NA	NA	0,45107925	NA	-2,2103983	0,38500536	NA	0,00022113	0,00018745	0,43806177	NA	9	-19297,8478	38613,7056	777,288862	5,36E-156
3401	-6,18359995	NA	NA	NA	-0,00186372	NA	NA	-1,61349146	0,35721774	NA	0,00021669	NA	0,46315983	-0,13737061	8	-19299,027	38614,062	777,6453	4,49E-156
3994	-8,97566397	0,33138274	NA	NA	-0,0012458	0,28352203	NA	-2,05206432	NA	0,01786019	0,00020271	-0,00039168	0,51529109	-0,14768431	11	-19296,1533	38614,3212	777,904449	3,94E-156

2354	-8,51824578	0,51124721	NA	NA	NA	0,38535796	-0,65213053	-1,50990804	NA	NA	0,00020523	NA	NA	-0,08563427	8	-19299,163	38614,3339	777,917181	3,92E-156
2345	-3,98530292	NA	NA	NA	-0,00234183	NA	-0,67333606	-1,50990804	NA	NA	0,00020523	NA	NA	-0,08563427	7	-19300,3085	38614,6232	778,20645	3,39E-156
3523	-11,7728913	NA	0,04392487	NA	NA	NA	NA	-2,59012234	0,38593256	0,02081194	0,00021148	NA	0,47514204	-0,138389	9	-19298,3139	38614,6377	778,221019	3,37E-156
1796	-12,0135881	0,70944129	0,06178489	NA	NA	NA	NA	-1,78635429	NA	NA	0,0002119	-0,00029974	0,47515083	NA	8	-19299,4045	38614,817	778,400234	3,08E-156
3913	-6,18359995	NA	NA	NA	-0,00186372	NA	NA	-1,71083893	0,37612338	NA	0,00021703	0,00013898	0,46389079	-0,13469622	9	-19298,5538	38615,1175	778,700789	2,65E-156
3394	-10,126072	0,56333515	NA	NA	NA	NA	NA	-1,61349146	0,35721774	NA	0,00021669	NA	0,46315983	-0,13737061	8	-19299,7795	38615,5669	779,150197	2,11E-156
481	-8,58193391	NA	NA	NA	NA	NA	-0,61393687	-2,87228819	0,44207022	0,01904584	0,00021405	NA	NA	NA	7	-19300,9545	38615,9152	779,498491	1,78E-156
4035	-11,7728913	NA	0,04392487	NA	NA	NA	NA	-2,66976045	0,40191971	0,02073197	0,0002118	0,00011864	0,47565984	-0,13600353	10	-19297,9719	38615,956	779,539229	1,74E-156
3339	-9,45532716	NA	0,05329757	NA	-0,00210061	NA	NA	-1,4783871	NA	NA	0,00020739	NA	0,51055393	-0,13912561	8	-19299,9958	38615,9996	779,582851	1,70E-156
292	-9,79595589	0,69445228	0,04389505	NA	NA	NA	-0,55018722	-1,812439	NA	NA	0,00020783	NA	NA	NA	7	-19301,0005	38616,0073	779,590528	1,70E-156
3906	-10,126072	0,56333515	NA	NA	NA	NA	NA	-1,71083893	0,37612338	NA	0,00021703	0,00013898	0,46389079	-0,13469622	9	-19299,3062	38616,6224	780,205686	1,25E-156
993	-8,58193391	NA	NA	NA	NA	NA	-0,61393687	-2,9468992	0,4586198	0,01897353	0,00021429	0,00012376	NA	NA	8	-19300,5791	38617,1662	780,749502	9,51E-157
1284	-12,0135881	0,70944129	0,06178489	NA	NA	NA	NA	-1,96172815	NA	NA	0,00021146	NA	0,48287384	NA	7	-19302,0094	38618,025	781,60825	6,19E-157
2388	-13,7669049	0,65178191	0,06462958	NA	NA	0,35119819	NA	-1,66433243	0,4162362	NA	0,00021617	NA	NA	-0,08904476	9	-19300,0806	38618,1711	781,754406	5,75E-157
2380	-11,9654714	0,51367836	0,06255183	NA	-0,0013444	NA	NA	-1,66433243	0,4162362	NA	0,00021617	NA	NA	-0,08904476	9	-19300,2692	38618,5483	782,13155	4,76E-157
2900	-13,7669049	0,65178191	0,06462958	NA	NA	0,35119819	NA	-1,73983563	0,43090563	NA	0,00021643	0,00010896	NA	-0,08710954	10	-19299,7909	38619,5939	783,177161	2,82E-157
2892	-11,9654714	0,51367836	0,06255183	NA	-0,0013444	NA	NA	-1,73983563	0,43090563	NA	0,00021643	0,00010896	NA	-0,08710954	10	-19299,9794	38619,971	783,554304	2,34E-157
306	-8,51824578	0,51124721	NA	NA	NA	0,38535796	-0,65213053	-1,812439	NA	NA	0,00020783	NA	NA	NA	7	-19302,9875	38619,9812	783,564434	2,33E-157
297	-3,98530292	NA	NA	NA	-0,00234183	NA	-0,67333606	-1,812439	NA	NA	0,00020783	NA	NA	NA	6	-19304,133	38620,2706	783,853924	2,01E-157
2500	-12,0135881	0,70944129	0,06178489	NA	NA	NA	NA	-2,55577604	0,44288659	0,01897716	0,00021141	NA	NA	-0,08883356	9	-19301,2861	38620,5821	784,165343	1,72E-158
3482	-8,97566397	0,33138274	NA	NA	-0,0012458	0,28352203	NA	-2,27275384	NA	0,01711404	0,00020255	NA	0,52288611	-0,14000571	10	-19300,494	38621,0002	784,583453	1,40E-157
3012	-12,0135881	0,70944129	0,06178489	NA	NA	NA	NA	-2,61719209	0,45517288	0,01892094	0,00021164	9,21E-05	NA	-0,08713002	10	-19301,0805	38622,1732	785,756486	7,78E-158
2978	-7,20552725	0,58574016	NA	NA	NA	NA	-0,62020516	-1,91049389	NA	0,01549989	0,00020133	-0,00049532	NA	-0,09486783	9	-19302,0819	38622,1738	785,757089	7,77E-158
2403	-9,52399274	NA	0,02470059	NA	NA	NA	-0,56950148	-1,66433243	0,4162362	NA	0,00021617	NA	NA	-0,08904476	8	-19303,8657	38623,7393	787,322624	3,55E-158
340	-13,7669049	0,65178191	0,06462958	NA	NA	0,35119819	NA	-1,97841134	0,41538406	NA	0,00021885	NA	NA	NA	8	-19304,0717	38624,1514	787,734679	2,89E-158
332	-11,9654714	0,51367836	0,06255183	NA	-0,0013444	NA	NA	-1,97841134	0,41538406	NA	0,00021885	NA	NA	NA	8	-19304,2603	38624,5285	788,111823	2,40E-159
3537	-9,09446706	NA	NA	NA	NA	0,38722306	NA	-2,59012234	0,38593256	0,02081194	0,00021148	NA	0,47514204	-0,138389	9	-19303,4473	38624,9045	788,48781	1,98E-158
2915	-9,52399274	NA	0,02470059	NA	NA	NA	-0,56950148	-1,73983563	0,43090563	NA	0,00021643	0,00010896	NA	-0,08710954	9	-19303,576	38625,1619	788,745157	1,74E-158
852	-13,7669049	0,65178191	0,06462958	NA	NA	0,35119819	NA	-2,0661368	0,43415925	NA	0,00021911	0,00013912	NA	NA	9	-19303,5938	38625,1975	788,780807	1,71E-159
844	-11,9654714	0,51367836	0,06255183	NA	-0,0013444	NA	NA	-2,0661368	0,43415925	NA	0,00021911	0,00013912	NA	NA	9	-19303,7824	38625,5747	789,157951	1,42E-158
4049	-9,09446706	NA	NA	NA	NA	0,38722306	NA	-2,66976045	0,40191971	0,02073197	0,0002118	0,00011864	0,47565984	-0,13600353	10	-19303,1053	38626,2227	789,806021	1,03E-158
452	-12,0135881	0,70944129	0,06178489	NA	NA	NA	NA	-2,87228819	0,44207022	0,01904584	0,00021405	NA	NA	NA	8	-19305,2403	38626,4886	790,071865	8,99E-159
2395	-11,3659105	NA	0,05744064	NA	-0,00198557	0,38445946	NA	-1,66433243	0,4162362	NA	0,00021617	NA	NA	-0,08904476	9	-19304,6912	38627,3924	790,975629	5,72E-160
964	-12,0135881	0,70944129	0,06178489	NA	NA	NA	NA	-2,9468992	0,4586198	0,01897353	0,00021429	0,00012376	NA	NA	9	-19304,8649	38627,7398	791,323098	4,81E-159
2907	-11,3659105	NA	0,05744064	NA	-0,00198557	0,38445946	NA	-1,73983563	0,43090563	NA	0,00021643	0,00010896	NA	-0,08710954	10	-19304,4015	38628,8151	792,398384	2,81E-159
930	-7,20552725	0,58574016	NA	NA	NA	NA	-0,62020516	-2,26386687	NA	0,01548996	0,00020413	-0,00046266	NA	NA	8	-19306,6795	38629,367	792,95024	2,13E-159
355	-9,52399274	NA	0,02470059	NA	NA	NA	-0,56950148	-1,97841134	0,41538406	NA	0,00021885	NA	NA	NA	7	-19307,8568	38629,7198	793,303119	1,79E-159
2995	-8,8387814	NA	0,03010563	NA	NA	0,51295904	-0,60572616	-1,91049389	NA	0,01549989	0,00020133	-0,00049532	NA	-0,09486783	10	-19305,0986	38630,2093	793,792612	1,40E-159
1803	-9,45532716	NA	0,05329757	NA	-0,00210061	NA	NA	-1,78635429	NA	NA	0,0002119	-0,00029974	0,47515083	NA	8	-19307,2837	38630,5753	794,158559	1,16E-159
867	-9,52399274	NA	0,02470059	NA	NA	NA	-0,56950148	-2,0661368	0,43415925	NA	0,00021911	0,00013912	NA	NA	8	-19307,3789	38630,7657	794,349025	1,06E-159
1353	-6,18359995	NA	NA	NA	-0,00186372	NA	NA	-2,09152745	0,35956015	NA	0,00022076	NA	0,43622845	NA	7	-19308,3893	38630,7848	794,3681	1,05E-159
1865	-6,18359995	NA	NA	NA	-0,00186372	NA	NA	-2,2103983	0,38500536	NA	0,00022113	0,00018745	0,43806177	NA	8	-19307,5153	38631,0385	794,621748	9,24E-160
1475	-11,7728913	NA	0,04392487	NA	NA	NA	NA	-3,07214819	0,38813022	0,02082345	0,00021556	NA	0,44803127	NA	8	-19307,7679	38631,5437	795,126991	7,18E-160
3993	-7,52922856	NA	NA	NA	-0,00175017	0,32067571	NA	-2,05206432	NA	0,01786019	0,00020271	-0,00039168	0,51529109	-0,14768431	10	-19305,9788	38631,9697	795,553006	5,80E-160
3978	-7,76791863	0,37580763	NA	NA	-0,00127907	NA	NA	-2,05206432	NA	0,01786019	0,00020271	-0,00039168	0,51529109	-0,14768431	10	-19306,0419	38632,0959	795,679198	5,45E-160
1987	-11,7728913	NA	0,04392487	NA	NA	NA	NA	-3,17508673	0,41103069	0,02071909	0,00021591	0,0001702	0,4495725	NA	9	-19307,053	38632,1159	795,699228	5,39E-160
1346	-10,126072	0,56333515	NA	NA	NA	NA	NA	-2,09152745	0,35956015	NA	0,00022076	NA	0,43622845	NA	7	-19309,1418	38632,2897	795,872997	4,94E-160
1858	-10,126072	0,56333515	NA	NA	NA	NA	NA	-2,2103983	0,38500536	NA	0,00022113	0,00018745	0,43806177	NA	8	-19308,2677	38632,5434	796,126645	4,35E-160
347	-11,3659105	NA	0,05744064	NA	-0,00198557	0,38445946	NA	-1,97841134	0,41538406	NA	0,00021885	NA	NA	NA	8	-19308,6823	38633,3726	796,955902	2,88E-160
1291	-9,45532716	NA	0,05329757	NA	-0,00210061	NA	NA	-1,96172815	NA	NA	0,00021146	NA	0,48287384	NA	7	-19309,8886	38633,7833	797,366575	2,34E-160
2466	-7,20552725	0,58574016	NA	NA	NA	NA	-0,62020516	-2,18775169	NA	0,01458917	0,00020109	NA	NA	-0,08533198	8	-19308,9429	38633,8937	797,476996	2,22E-160
1946	-8,97566397	0,33138274	NA	NA	-0,0012458	0,28352203	NA	-2,58652225	NA	0,01772679	0,00020696	-0,00034396	0,48679163	NA	10	-19307,0991	38634,2103	797,793556	1,89E-160
859	-11,3659105	NA	0,05744064	NA	-0,00198557	0,38445946	NA	-2,0661368	0,43415925	NA	0,00021911	0,00013912	NA	NA	9	-19308,2044	38634,4188	798,00203	1,70E-161
2401	-8,58193391	NA	NA	NA	NA	NA	-0,61393687	-1,66433243	0,4162362	NA	0,00021617	NA	NA	-0,08904476	7	-19310,8818	38635,7697	799,353016	8,68E-161
2507	-9,45532716	NA	0,05329757	NA	-0,00210061	NA	NA	-2,55577604	0,44288659	0,01897716	0,00021141	NA	NA	-0,08883356	9	-19309,1652	38636,3404	799,923668	6,52E-161
3866	-8,97566397	0,33138274	NA	NA	-0,0012458	0,28352203	NA	-1,25486087	NA	NA	0,00020772	-0,0003441	0,50313521	-0,14558566	10	-19308,4561	38636,9243	800,507571	4,87E-161
2913	-8,58193391	NA	NA	NA	NA	NA	-0,61393687	-1,73983563	0,43090563	NA	0,00021643	0,00010896	NA	-0,08710954	8	-19310,592	38637,1921	800,775328	4,26E-161
947	-8,8387814	NA	0,03010563	NA	NA	0,51295904	-0,60572616	-2,26386687	NA	0,01548996	0,00020413	-0,00046266	NA	NA	9	-19309,6962	38637,4023	800,985542	3,84E-161
3986	-8,08714822	0,50972296	NA	NA	NA	0,2963004	NA	-2,05206432	NA	0,01786019	0,00020271	-0,00039168	0,51529109	-0,14768431	10	-19308,8497	38637,7115	801,294767	3,29E-161
3019	-9,45532716	NA	0,05329757	NA	-0,00210061	NA	NA	-2,61719209	0,45517288	0,01892094	0,00021164	9,21E-05	NA	-0,08713002	10	-19308,9597	38637,9315	801,514811	2,94E-161
3987	-11,2963041	NA	0,04958538	NA	NA	0,45107925	NA	-2,05206432	NA	0,01786019	0,00020271	-0,00039168	0,51529109	-0,14768431	10	-19309,2254	38638,4629	802,046173	2,26E-161

3481	-7,52922856	NA	NA	NA	-0,00175017	0,32067571	NA	-2,27275384	NA	0,01711404	0,00020255	NA	0,52288611	-0,14000571	9	-19310,3195	38638,649	802,232231	2,06E-161
2850	-7,20552725	0,58574016	NA	NA	NA	NA	-0,62020516	-1,2176888	NA	NA	0,00020569	-0,00045432	NA	-0,0941422	8	-19311,3517	38638,7114	802,294666	1,99E-161
3466	-7,76791863	0,37580763	NA	NA	-0,00127907	NA	NA	-2,27275384	NA	0,01711404	0,00020255	NA	0,52288611	-0,14000571	9	-19310,3826	38638,7751	802,358423	1,93E-161
1434	-8,97566397	0,33138274	NA	NA	-0,0012458	0,28352203	NA	-2,75826159	NA	0,01709531	0,00020664	NA	0,4949792	NA	9	-19310,4875	38638,985	802,568257	1,74E-161
418	-7,20552725	0,58574016	NA	NA	NA	NA	-0,62020516	-2,49206855	NA	0,01465098	0,00020366	NA	NA	NA	7	-19312,7328	38639,4718	803,055121	1,36E-161
3354	-8,97566397	0,33138274	NA	NA	-0,0012458	0,28352203	NA	-1,4783871	NA	NA	0,00020739	NA	0,51055393	-0,13912561	9	-19311,85	38641,7099	805,293207	4,45E-162
353	-8,58193391	NA	NA	NA	NA	NA	-0,61393687	-1,97841134	0,41538406	NA	0,00021885	NA	NA	NA	6	-19314,8729	38641,7505	805,333733	4,36E-162
1489	-9,09446706	NA	NA	NA	NA	0,38722306	NA	-3,07214819	0,38813022	0,02082345	0,00021556	NA	0,44803127	NA	8	-19312,9013	38641,8105	805,393783	4,23E-162
2483	-8,8387814	NA	0,03010563	NA	NA	0,51295904	-0,60572616	-2,18775169	NA	0,01458917	0,00020109	NA	NA	-0,08533198	9	-19311,9595	38641,929	805,512297	3,99E-163
459	-9,45532716	NA	0,05329757	NA	-0,00210061	NA	NA	-2,87228819	0,44207022	0,01904584	0,00021405	NA	NA	NA	8	-19313,1195	38642,2469	805,83019	3,40E-162
2001	-9,09446706	NA	NA	NA	NA	0,38722306	NA	-3,17508673	0,41103069	0,02071909	0,00021591	0,0001702	0,4495725	NA	9	-19312,1864	38642,3827	805,966019	3,18E-162
865	-8,58193391	NA	NA	NA	NA	NA	-0,61393687	-2,0661368	0,43415925	NA	0,00021911	0,00013912	NA	NA	7	-19314,395	38642,7961	806,379417	2,59E-162
971	-9,45532716	NA	0,05329757	NA	-0,00210061	NA	NA	-2,9468992	0,4586198	0,01897353	0,00021429	0,00012376	NA	NA	9	-19312,7441	38643,4981	807,081423	1,82E-162
3474	-8,08714822	0,50972296	NA	NA	NA	0,2963004	NA	-2,27275384	NA	0,01711404	0,00020255	NA	0,52288611	-0,14000571	9	-19313,1904	38644,3907	807,973992	1,16E-162
3475	-11,2963041	NA	0,04958538	NA	NA	0,45107925	NA	-2,27275384	NA	0,01711404	0,00020255	NA	0,52288611	-0,14000571	9	-19313,5661	38645,1421	808,725398	8,00E-163
802	-7,20552725	0,58574016	NA	NA	NA	NA	-0,62020516	-1,56810442	NA	NA	0,00020849	-0,0004232	NA	NA	7	-19315,8947	38645,7956	809,378874	5,77E-163
3395	-11,7728913	NA	0,04392487	NA	NA	NA	NA	-1,61349146	0,35721774	NA	0,00021669	NA	0,46315983	-0,13737061	8	-19314,982	38645,9719	809,555199	5,28E-163
2372	-12,0135881	0,70944129	0,06178489	NA	NA	NA	NA	-1,66433243	0,4162362	NA	0,00021617	NA	NA	-0,08904476	8	-19315,1676	38646,3431	809,92639	4,39E-163
2867	-8,8387814	NA	0,03010563	NA	NA	0,51295904	-0,60572616	-1,2176888	NA	NA	0,00020569	-0,00045432	NA	-0,0941422	9	-19314,3684	38646,7467	810,329967	3,59E-163
3907	-11,7728913	NA	0,04392487	NA	NA	NA	NA	-1,71083893	0,37612338	NA	0,00021703	0,00013898	0,46389079	-0,13469622	9	-19314,5087	38647,0274	810,610688	3,12E-163
435	-8,8387814	NA	0,03010563	NA	NA	0,51295904	-0,60572616	-2,49206855	NA	0,01465098	0,00020366	NA	NA	NA	8	-19315,7495	38647,5069	811,090201	2,45E-163
2884	-12,0135881	0,70944129	0,06178489	NA	NA	NA	NA	-1,73983563	0,43090563	NA	0,00021643	0,00010896	NA	-0,08710954	9	-19314,8778	38647,7656	811,348923	2,15E-163
2338	-7,20552725	0,58574016	NA	NA	NA	NA	-0,62020516	-1,50990804	NA	NA	0,00020523	NA	NA	-0,08563427	7	-19317,197	38648,4002	811,983512	1,57E-163
2993	-6,3315051	NA	NA	NA	NA	0,4838605	-0,6580268	-1,91049389	NA	0,01549989	0,00020133	-0,00049532	NA	-0,09486783	9	-19315,5131	38649,0362	812,619473	1,14E-163
1945	-7,52922856	NA	NA	NA	-0,00175017	0,32067571	NA	-2,58652225	NA	0,01772679	0,00020696	-0,00034396	0,48679163	NA	9	-19316,9246	38651,8591	815,442334	2,78E-164
1930	-7,76791863	0,37580763	NA	NA	-0,00127907	NA	NA	-2,58652225	NA	0,01772679	0,00020696	-0,00034396	0,48679163	NA	9	-19316,9876	38651,9852	815,568526	2,61E-164
324	-12,0135881	0,70944129	0,06178489	NA	NA	NA	NA	-1,97841134	0,41538406	NA	0,00021885	NA	NA	NA	7	-19319,1587	38652,3236	815,906885	2,21E-164
836	-12,0135881	0,70944129	0,06178489	NA	NA	NA	NA	-2,0661368	0,43415925	NA	0,00021911	0,00013912	NA	NA	8	-19318,6808	38653,3695	816,952792	1,31E-164
819	-8,8387814	NA	0,03010563	NA	NA	0,51295904	-0,60572616	-1,56810442	NA	NA	0,00020849	-0,0004232	NA	NA	8	-19318,9114	38653,8307	817,413954	1,04E-164
290	-7,20552725	0,58574016	NA	NA	NA	NA	-0,62020516	-1,812439	NA	NA	0,00020783	NA	NA	NA	6	-19321,0215	38654,0477	817,630986	9,32E-165
3865	-7,52922856	NA	NA	NA	-0,00175017	0,32067571	NA	-1,25486087	NA	NA	0,00020772	-0,0003441	0,50313521	-0,14558566	9	-19318,2816	38654,5731	818,156349	7,17E-165
3850	-7,76791863	0,37580763	NA	NA	-0,00127907	NA	NA	-1,25486087	NA	NA	0,00020772	-0,0003441	0,50313521	-0,14558566	9	-19318,3447	38654,6993	818,282541	6,73E-165
3977	-6,18359995	NA	NA	NA	-0,00186372	NA	NA	-2,05206432	NA	0,01786019	0,00020271	-0,00039168	0,51529109	-0,14768431	9	-19318,8928	38655,7956	819,378837	3,89E-165
945	-6,3315051	NA	NA	NA	NA	0,4838605	-0,6580268	-2,26386687	NA	0,01548996	0,00020413	-0,00046266	NA	NA	8	-19320,1107	38656,2293	819,812624	3,13E-165
3409	-9,09446706	NA	NA	NA	NA	0,38722306	NA	-1,61349146	0,35721774	NA	0,00021669	NA	0,46315983	-0,13737061	8	-19320,1154	38656,2387	819,82199	3,12E-166
1818	-8,97566397	0,33138274	NA	NA	-0,0012458	0,28352203	NA	-1,78635429	NA	NA	0,0002119	-0,00029974	0,47515083	NA	9	-19319,1378	38656,2856	819,868915	3,04E-165
2355	-8,8387814	NA	0,03010563	NA	NA	0,51295904	-0,60572616	-1,50990804	NA	NA	0,00020523	NA	NA	-0,08563427	8	-19320,2137	38656,4353	820,018592	2,82E-165
1433	-7,52922856	NA	NA	NA	-0,00175017	0,32067571	NA	-2,75826159	NA	0,01709531	0,00020664	NA	0,4949792	NA	8	-19320,313	38656,634	820,217257	2,56E-165
1418	-7,76791863	0,37580763	NA	NA	-0,00127907	NA	NA	-2,75826159	NA	0,01709531	0,00020664	NA	0,4949792	NA	8	-19320,3761	38656,7602	820,343449	2,40E-165
3921	-9,09446706	NA	NA	NA	NA	0,38722306	NA	-1,71083893	0,37612338	NA	0,00021703	0,00013898	0,46389079	-0,13469622	9	-19319,6421	38657,2942	820,87748	1,84E-165
3970	-10,126072	0,56333515	NA	NA	NA	NA	NA	-2,05206432	NA	0,01786019	0,00020271	-0,00039168	0,51529109	-0,14768431	9	-19319,6453	38657,3005	820,883734	1,83E-165
1938	-8,08714822	0,50972296	NA	NA	NA	0,2963004	NA	-2,58652225	NA	0,01772679	0,00020696	-0,00034396	0,48679163	NA	9	-19319,7954	38657,6008	821,184096	1,58E-165
1939	-11,2963041	NA	0,04958538	NA	NA	0,45107925	NA	-2,58652225	NA	0,01772679	0,00020696	-0,00034396	0,48679163	NA	9	-19320,1711	38658,3522	821,935501	1,08E-165
3353	-7,52922856	NA	NA	NA	-0,00175017	0,32067571	NA	-1,4783871	NA	NA	0,00020739	NA	0,51055393	-0,13912561	8	-19321,6755	38659,3589	822,942207	6,55E-167
3338	-7,76791863	0,37580763	NA	NA	-0,00127907	NA	NA	-1,4783871	NA	NA	0,00020739	NA	0,51055393	-0,13912561	8	-19321,7386	38659,4851	823,068399	6,15E-167
1306	-8,97566397	0,33138274	NA	NA	-0,0012458	0,28352203	NA	-1,96172815	NA	NA	0,00021146	NA	0,48287384	NA	8	-19321,7427	38659,4934	823,07671	6,12E-166
3858	-8,08714822	0,50972296	NA	NA	NA	0,2963004	NA	-1,25486087	NA	NA	0,00020772	-0,0003441	0,50313521	-0,14558566	9	-19321,1524	38660,3148	823,898111	4,06E-166
2481	-6,3315051	NA	NA	NA	NA	0,4838605	-0,6580268	-2,18775169	NA	0,01458917	0,00020109	NA	NA	-0,08533198	8	-19322,3741	38660,7561	824,339379	3,26E-166
3859	-11,2963041	NA	0,04958538	NA	NA	0,45107925	NA	-1,25486087	NA	NA	0,00020772	-0,0003441	0,50313521	-0,14558566	9	-19321,5281	38661,0662	824,649516	2,79E-166
3521	-15,360872	NA	NA	NA	NA	NA	NA	-2,59012234	0,38593256	0,02081194	0,00021148	NA	0,47514204	-0,138389	8	-19322,5894	38661,1868	824,770095	2,62E-166
2522	-8,97566397	0,33138274	NA	NA	-0,0012458	0,28352203	NA	-2,55577604	0,44288659	0,01897716	0,00021141	NA	NA	-0,08883356	10	-19321,0194	38662,051	825,634245	1,70E-166
307	-8,8387814	NA	0,03010563	NA	NA	0,51295904	-0,60572616	-1,812439	NA	NA	0,00020783	NA	NA	NA	7	-19324,0382	38662,0826	825,665845	1,68E-166
2379	-9,45532716	NA	0,05329757	NA	-0,00210061	NA	NA	-1,66433243	0,4162362	NA	0,00021617	NA	NA	-0,08904476	8	-19323,0467	38662,1014	825,684715	1,66E-166
1426	-8,08714822	0,50972296	NA	NA	NA	0,2963004	NA	-2,75826159	NA	0,01709531	0,00020664	NA	0,4949792	NA	8	-19323,1839	38662,3757	825,959018	1,45E-166
3465	-6,18359995	NA	NA	NA	-0,00186372	NA	NA	-2,27275384	NA	0,01711404	0,00020255	NA	0,52288611	-0,14000571	8	-19323,2335	38662,475	826,058283	1,38E-166
4033	-15,360872	NA	NA	NA	NA	NA	NA	-2,66976045	0,40191971	0,02073197	0,0002118	0,00011864	0,47565984	-0,13600353	9	-19322,2474	38662,5048	826,088084	1,36E-166
1347	-11,7728913	NA	0,04392487	NA	NA	NA	NA	-2,09152745	0,35956015	NA	0,00022076	NA	0,43622845	NA	7	-19324,3443	38662,6947	826,277999	1,23E-166
1859	-11,7728913	NA	0,04392487	NA	NA	NA	NA	-2,2103983	0,38500536	NA	0,00022113	0,00018745	0,43806177	NA	8	-19323,4702	38662,9484	826,531647	1,09E-166
2972	-9,61919161	0,46601352	0,06534536	NA	-0,00131078	0,33938341	NA	-1,91049389	NA	0,01549989	0,00020133	-0,00049532	NA	-0,09486783	11	-19320,4708	38662,9563	826,539569	1,08E-166
1427	-11,2963041	NA	0,04958538	NA	NA	0,45107925	NA	-2,75826159	NA	0,01709531	0,00020664	NA	0,4949792	NA	8	-19323,5596	38663,1271	826,710424	9,95E-169
2891	-9,45532716	NA	0,05329757	NA	-0,00210061	NA	NA	-1,73983563	0,43090563	NA	0,00021643	0,00010896	NA	-0,08710954	9	-19322,757	38663,524	827,107248	8,16E-167

3034	-8,97566397	0,33138274	NA	NA	-0,0012458	0,28352203	NA	-2,61719209	0,45517288	0,01892094	0,00021164	9,21E-05	NA	-0,08713002	11	-19320,8139	38663,6423	827,22561	7,69E-169
3458	-10,126072	0,56333515	NA	NA	NA	NA	NA	-2,27275384	NA	0,01711404	0,00020255	NA	0,52288611	-0,14000571	8	-19323,986	38663,9799	827,563181	6,49E-167
3346	-8,08714822	0,50972296	NA	NA	NA	0,2963004	NA	-1,4783871	NA	NA	0,00020739	NA	0,51055393	-0,13912561	8	-19324,5464	38665,1007	828,683968	3,71E-167
2865	-6,3315051	NA	NA	NA	NA	0,4838605	-0,6580268	-1,2176888	NA	NA	0,00020569	-0,00045432	NA	-0,0941422	8	-19324,7829	38665,5738	829,157049	2,93E-167
3347	-11,2963041	NA	0,04958538	NA	NA	0,45107925	NA	-1,4783871	NA	NA	0,00020739	NA	0,51055393	-0,13912561	8	-19324,9221	38665,8521	829,435373	2,55E-167
433	-6,3315051	NA	NA	NA	NA	0,4838605	-0,6580268	-2,49206855	NA	0,01465098	0,00020366	NA	NA	NA	7	-19326,164	38666,3342	829,917505	2,00E-167
474	-8,97566397	0,33138274	NA	NA	-0,0012458	0,28352203	NA	-2,87228819	0,44207022	0,01904584	0,00021405	NA	NA	NA	9	-19324,9737	38667,9573	831,540546	8,89E-168
331	-9,45532716	NA	0,05329757	NA	-0,00210061	NA	NA	-1,97841134	0,41538406	NA	0,00021885	NA	NA	NA	7	-19327,0379	38668,0819	831,66521	8,35E-168
843	-9,45532716	NA	0,05329757	NA	-0,00210061	NA	NA	-2,0661368	0,43415925	NA	0,00021911	0,00013912	NA	NA	8	-19326,5599	38669,1278	832,711116	4,95E-168
986	-8,97566397	0,33138274	NA	NA	-0,0012458	0,28352203	NA	-2,9468992	0,4586198	0,01897353	0,00021429	0,00012376	NA	NA	10	-19324,5983	38669,2087	832,792	4,75E-168
924	-9,61919161	0,46601352	0,06534536	NA	-0,00131078	0,33938341	NA	-2,26386687	NA	0,01548996	0,00020413	-0,00046266	NA	NA	10	-19325,0684	38670,149	833,732277	2,97E-168
817	-6,3315051	NA	NA	NA	NA	0,4838605	-0,6580268	-1,56810442	NA	NA	0,00020849	-0,0004232	NA	NA	7	-19329,3259	38672,658	836,241257	8,47E-169
1361	-9,09446706	NA	NA	NA	NA	0,38722306	NA	-2,09152745	0,35956015	NA	0,00022076	NA	0,43622845	NA	7	-19329,4777	38672,9615	836,544791	7,28E-170
1873	-9,09446706	NA	NA	NA	NA	0,38722306	NA	-2,2103983	0,38500536	NA	0,00022113	0,00018745	0,43806177	NA	8	-19328,6036	38673,2152	836,798439	6,41E-169
1817	-7,52922856	NA	NA	NA	-0,00175017	0,32067571	NA	-1,78635429	NA	NA	0,0002119	-0,00029974	0,47515083	NA	8	-19328,9633	38673,9346	837,517914	4,48E-169
1802	-7,76791863	0,37580763	NA	NA	-0,00127907	NA	NA	-1,78635429	NA	NA	0,0002119	-0,00029974	0,47515083	NA	8	-19329,0264	38674,0608	837,644106	4,20E-170
2460	-9,61919161	0,46601352	0,06534536	NA	-0,00131078	0,33938341	NA	-2,18775169	NA	0,01458917	0,00020109	NA	NA	-0,08533198	10	-19327,3318	38674,6758	838,259032	3,09E-169
2353	-6,3315051	NA	NA	NA	NA	0,4838605	-0,6580268	-1,50990804	NA	NA	0,00020523	NA	NA	-0,08563427	7	-19330,6282	38675,2626	838,845895	2,30E-169
1929	-6,18359995	NA	NA	NA	-0,00186372	NA	NA	-2,58652225	NA	0,01772679	0,00020696	-0,00034396	0,48679163	NA	8	-19329,8386	38675,6851	839,268386	1,87E-169
1305	-7,52922856	NA	NA	NA	-0,00175017	0,32067571	NA	-1,96172815	NA	NA	0,00021146	NA	0,48287384	NA	7	-19331,5682	38677,1427	840,725931	9,00E-170
1922	-10,126072	0,56333515	NA	NA	NA	NA	NA	-2,58652225	NA	0,01772679	0,00020696	-0,00034396	0,48679163	NA	8	-19330,591	38677,19	840,773284	8,79E-170
1290	-7,76791863	0,37580763	NA	NA	-0,00127907	NA	NA	-1,96172815	NA	NA	0,00021146	NA	0,48287384	NA	7	-19331,6313	38677,2688	840,852123	8,45E-170
1473	-15,360872	NA	NA	NA	NA	NA	NA	-3,07214819	0,38813022	0,02082345	0,00021556	NA	0,44803127	NA	7	-19332,0434	38678,093	841,676289	5,60E-170
3849	-6,18359995	NA	NA	NA	-0,00186372	NA	NA	-1,25486087	NA	NA	0,00020772	-0,0003441	0,50313521	-0,14558566	8	-19331,1956	38678,3991	841,982401	4,80E-170
1985	-15,360872	NA	NA	NA	NA	NA	NA	-3,17508673	0,41103069	0,02071909	0,00021591	0,0001702	0,4495725	NA	8	-19331,3285	38678,665	842,248304	4,20E-170
2844	-9,61919161	0,46601352	0,06534536	NA	-0,00131078	0,33938341	NA	-1,2176888	NA	NA	0,00020569	-0,00045432	NA	-0,0941422	10	-19329,7406	38679,4934	843,076702	2,78E-170
1810	-8,08714822	0,50972296	NA	NA	NA	0,2963004	NA	-1,78635429	NA	NA	0,0002119	-0,00029974	0,47515083	NA	8	-19331,8342	38679,6764	843,259676	2,54E-170
2521	-7,52922856	NA	NA	NA	-0,00175017	0,32067571	NA	-2,55577604	0,44288659	0,01897716	0,00021141	NA	NA	-0,08883356	9	-19330,8449	38679,6997	843,283023	2,51E-170
2506	-7,76791863	0,37580763	NA	NA	-0,00127907	NA	NA	-2,55577604	0,44288659	0,01897716	0,00021141	NA	NA	-0,08883356	9	-19330,908	38679,8259	843,409215	2,35E-170
3842	-10,126072	0,56333515	NA	NA	NA	NA	NA	-1,25486087	NA	NA	0,00020772	-0,0003441	0,50313521	-0,14558566	8	-19331,948	38679,904	843,487299	2,26E-170
412	-9,61919161	0,46601352	0,06534536	NA	-0,00131078	0,33938341	NA	-2,49206855	NA	0,01465098	0,00020366	NA	NA	NA	9	-19331,1217	38680,2534	843,836715	1,90E-170
1811	-11,2963041	NA	0,04958538	NA	NA	0,45107925	NA	-1,78635429	NA	NA	0,0002119	-0,00029974	0,47515083	NA	8	-19332,2099	38680,4278	844,011081	1,74E-170
1417	-6,18359995	NA	NA	NA	-0,00186372	NA	NA	-2,75826159	NA	0,01709531	0,00020664	NA	0,4949792	NA	7	-19333,227	38680,4603	844,043531	1,71E-170
305	-6,3315051	NA	NA	NA	NA	0,4838605	-0,6580268	-1,812439	NA	0,01786019	0,00020783	NA	NA	NA	6	-19334,4527	38680,9101	844,49337	1,37E-170
3033	-7,52922856	NA	NA	NA	-0,00175017	0,32067571	NA	-2,61719209	0,45517288	0,01892094	0,00021164	9,21E-05	NA	-0,08713002	10	-19330,6394	38681,2909	844,874166	1,13E-170
3018	-7,76791863	0,37580763	NA	NA	-0,00127907	NA	NA	-2,61719209	0,45517288	0,01892094	0,00021164	9,21E-05	NA	-0,08713002	10	-19330,7025	38681,4171	845,000358	1,06E-170
1410	-10,126072	0,56333515	NA	NA	NA	NA	NA	-2,75826159	NA	0,01709531	0,00020664	NA	0,4949792	NA	7	-19333,9795	38681,9652	845,548428	8,07E-171
1298	-8,08714822	0,50972296	NA	NA	NA	0,2963004	NA	-1,96172815	NA	NA	0,00021146	NA	0,48287384	NA	7	-19334,4391	38682,8844	846,467692	5,10E-171
3337	-6,18359995	NA	NA	NA	-0,00186372	NA	NA	-1,4783871	NA	NA	0,00020739	NA	0,51055393	-0,13912561	7	-19334,5895	38683,1852	846,768481	4,39E-171
1299	-11,2963041	NA	0,04958538	NA	NA	0,45107925	NA	-1,96172815	NA	NA	0,00021146	NA	0,48287384	NA	7	-19334,8148	38683,6358	847,219098	3,50E-172
3330	-10,126072	0,56333515	NA	NA	NA	NA	NA	-1,4783871	NA	NA	0,00020739	NA	0,51055393	-0,13912561	7	-19335,342	38684,6901	848,273378	2,07E-171
2514	-8,08714822	0,50972296	NA	NA	NA	0,2963004	NA	-2,55577604	0,44288659	0,01897716	0,00021141	NA	NA	-0,08883356	9	-19333,7158	38685,4415	849,024785	1,42E-171
473	-7,52922856	NA	NA	NA	-0,00175017	0,32067571	NA	-2,87228819	0,44207022	0,01904584	0,00021405	NA	NA	NA	8	-19334,7992	38685,6063	849,189546	1,31E-171
458	-7,76791863	0,37580763	NA	NA	-0,00127907	NA	NA	-2,87228819	0,44207022	0,01904584	0,00021405	NA	NA	NA	8	-19334,8622	38685,7325	849,315738	1,23E-171
2515	-11,2963041	NA	0,04958538	NA	NA	0,45107925	NA	-2,55577604	0,44288659	0,01897716	0,00021141	NA	NA	-0,08883356	9	-19334,0915	38686,1929	849,77619	9,75E-172
796	-9,61919161	0,46601352	0,06534536	NA	-0,00131078	0,33938341	NA	-1,56810442	NA	NA	0,00020849	-0,0004232	NA	NA	9	-19334,2836	38686,5772	850,160467	8,05E-172
985	-7,52922856	NA	NA	NA	-0,00175017	0,32067571	NA	-2,9468992	0,4586198	0,01897353	0,00021429	0,00012376	NA	NA	9	-19334,4238	38686,8575	850,440779	6,99E-172
970	-7,76791863	0,37580763	NA	NA	-0,00127907	NA	NA	-2,9468992	0,4586198	0,01897353	0,00021429	0,00012376	NA	NA	9	-19334,4869	38686,9837	850,566971	6,57E-172
3026	-8,08714822	0,50972296	NA	NA	NA	0,2963004	NA	-2,61719209	0,45517288	0,01892094	0,00021164	9,21E-05	NA	-0,08713002	10	-19333,5102	38687,0326	850,615928	6,41E-172
3971	-11,7728913	NA	0,04392487	NA	NA	NA	NA	-2,05206432	NA	0,01786019	0,00020271	-0,00039168	0,51529109	-0,14768431	9	-19334,8478	38687,7055	851,288736	4,58E-173
3027	-11,2963041	NA	0,04958538	NA	NA	0,45107925	NA	-2,61719209	0,45517288	0,01892094	0,00021164	9,21E-05	NA	-0,08713002	10	-19333,8859	38687,7841	851,367333	4,40E-172
2394	-8,97566397	0,33138274	NA	NA	-0,0012458	0,28352203	NA	-1,66433243	0,4162362	NA	0,00021617	NA	NA	-0,08904476	9	-19334,9009	38687,8118	851,395071	4,34E-172
2964	-13,7669049	0,65178191	0,06462958	NA	NA	0,35119819	NA	-1,91049389	NA	0,01549989	0,00020133	-0,00049532	NA	-0,09486783	10	-19334,3532	38688,7185	852,301751	2,76E-172
2956	-11,9654714	0,51367836	0,06255183	NA	-0,0013444	NA	NA	-1,91049389	NA	0,01549989	0,00020133	-0,00049532	NA	-0,09486783	10	-19334,5417	38689,0956	852,678895	2,28E-172
2332	-9,61919161	0,46601352	0,06534536	NA	-0,00131078	0,33938341	NA	-1,50990804	NA	NA	0,00020523	NA	NA	-0,08563427	9	-19335,5859	38689,1818	852,765105	2,19E-172
2906	-8,97566397	0,33138274	NA	NA	-0,0012458	0,28352203	NA	-1,73983563	0,43090563	NA	0,00021643	0,00010896	NA	-0,08710954	10	-19334,6112	38689,2345	852,817826	2,13E-172
466	-8,08714822	0,50972296	NA	NA	NA	0,2963004	NA	-2,87228819	0,44207022	0,01904584	0,00021405	NA	NA	NA	8	-19337,67	38691,348	854,931307	7,41E-173
467	-11,2963041	NA	0,04958538	NA	NA	0,45107925	NA	-2,87228819	0,44207022	0,01904584	0,00021405	NA	NA	NA	8	-19338,0457	38692,0994	855,682712	5,09E-174
3393	-15,360872	NA	NA	NA	NA	NA	NA	-1,61349146	0,35721774	NA	0,00021669	NA	0,46315983	-0,13737061	7	-19339,2575	38692,5212	856,104496	4,12E-173
978	-8,08714822	0,50972296	NA	NA	NA	0,2963004	NA	-2,9468992	0,4586198	0,01897353	0,00021429	0,00012376	NA	NA	9	-19337,2947	38692,5993	856,18254	3,96E-173
979	-11,2963041	NA	0,04958538	NA	NA	0,45107925	NA	-2,9468992	0,4586198	0,01897353	0,00021429	0,00012376	NA	NA	9	-19337,6704	38693,3507	856,933945	2,72E-173

3905	-15,360872	NA	NA	NA	NA	NA	NA	-1,71083893	0,37612338	NA	0,00021703	0,00013898	0,46389079	-0,13469622	8	-19338,7843	38693,5765	857,159764	2,43E-173
346	-8,97566397	0,33138274	NA	NA	-0,0012458	0,28352203	NA	-1,97841134	0,41538406	NA	0,00021885	NA	NA	NA	8	-19338,8921	38693,7921	857,375345	2,18E-173
2979	-9,52399274	NA	0,02470059	NA	NA	NA	-0,56950148	-1,91049389	NA	0,01549989	0,00020133	-0,00049532	NA	-0,09486783	9	-19338,1383	38694,2865	857,869748	1,70E-173
3459	-11,7728913	NA	0,04392487	NA	NA	NA	NA	-2,27275384	NA	0,01711404	0,00020255	NA	0,52288611	-0,14000571	8	-19339,1885	38694,3849	857,968182	1,62E-173
284	-9,61919161	0,46601352	0,06534536	NA	-0,00131078	0,33938341	NA	-1,812439	NA	NA	0,00020783	NA	NA	NA	8	-19339,4104	38694,8289	858,412137	1,30E-173
858	-8,97566397	0,33138274	NA	NA	-0,0012458	0,28352203	NA	-2,0661368	0,43415925	NA	0,00021911	0,00013912	NA	NA	9	-19338,4141	38694,8382	858,421472	1,29E-173
916	-13,7669049	0,65178191	0,06462958	NA	NA	0,35119819	NA	-2,26386687	NA	0,01548996	0,00020413	-0,00046266	NA	NA	9	-19338,9507	38695,9114	859,494681	7,56E-174
908	-11,9654714	0,51367836	0,06255183	NA	-0,0013444	NA	NA	-2,26386687	NA	0,01548996	0,00020413	-0,00046266	NA	NA	9	-19339,1393	38696,2885	859,871824	6,26E-174
1801	-6,18359995	NA	NA	NA	-0,00186372	NA	NA	-1,78635429	NA	NA	0,0002119	-0,00029974	0,47515083	NA	7	-19341,8774	38697,7609	861,344188	3,00E-175
2971	-11,3659105	NA	0,05744064	NA	-0,00198557	0,38445946	NA	-1,91049389	NA	0,01549989	0,00020133	-0,00049532	NA	-0,09486783	10	-19338,9638	38697,9397	861,522974	2,74E-174
3985	-9,09446706	NA	NA	NA	NA	0,38722306	NA	-2,05206432	NA	0,01786019	0,00020271	-0,00039168	0,51529109	-0,14768431	9	-19339,9811	38697,9722	861,555527	2,70E-174
1794	-10,126072	0,56333515	NA	NA	NA	NA	NA	-1,78635429	NA	NA	0,0002119	-0,00029974	0,47515083	NA	7	-19342,6298	38699,2658	862,849086	1,41E-174
2452	-13,7669049	0,65178191	0,06462958	NA	NA	0,35119819	NA	-2,18775169	NA	0,01458917	0,00020109	NA	NA	-0,08533198	9	-19341,2141	38700,4382	864,021436	7,87E-175
2444	-11,9654714	0,51367836	0,06255183	NA	-0,0013444	NA	NA	-2,18775169	NA	0,01458917	0,00020109	NA	NA	-0,08533198	9	-19341,4027	38700,8153	864,39858	6,51E-175
1289	-6,18359995	NA	NA	NA	-0,00186372	NA	NA	-1,96172815	NA	NA	0,00021146	NA	0,48287384	NA	6	-19344,4823	38700,9691	864,552426	6,03E-175
931	-9,52399274	NA	0,02470059	NA	NA	NA	-0,56950148	-2,26386687	NA	0,01548996	0,00020413	-0,00046266	NA	NA	8	-19342,7358	38701,4796	865,062899	4,67E-175
1282	-10,126072	0,56333515	NA	NA	NA	NA	NA	-1,96172815	NA	NA	0,00021146	NA	0,48287384	NA	6	-19345,2347	38702,474	866,057324	2,84E-175
2505	-6,18359995	NA	NA	NA	-0,00186372	NA	NA	-2,55577604	0,44288659	0,01897716	0,00021141	NA	NA	-0,08883356	8	-19343,7589	38703,5258	867,109076	1,68E-176
3473	-9,09446706	NA	NA	NA	NA	0,38722306	NA	-2,27275384	NA	0,01711404	0,00020255	NA	0,52288611	-0,14000571	8	-19344,3219	38704,6517	868,234974	9,57E-176
2498	-10,126072	0,56333515	NA	NA	NA	NA	NA	-2,55577604	0,44288659	0,01897716	0,00021141	NA	NA	-0,08883356	8	-19344,5114	38705,0307	868,613973	7,92E-176
3017	-6,18359995	NA	NA	NA	-0,00186372	NA	NA	-2,61719209	0,45517288	0,01892094	0,00021164	9,21E-05	NA	-0,08713002	9	-19343,5534	38705,1167	868,699997	7,58E-176
923	-11,3659105	NA	0,05744064	NA	-0,00198557	0,38445946	NA	-2,26386687	NA	0,01548996	0,00020413	-0,00046266	NA	NA	9	-19343,5613	38705,1326	868,715904	7,52E-176
2836	-13,7669049	0,65178191	0,06462958	NA	NA	0,35119819	NA	-1,2176888	NA	NA	0,00020569	-0,00045432	NA	-0,0941422	9	-19343,6229	38705,2558	868,839106	7,07E-176
2393	-7,52922856	NA	NA	NA	-0,00175017	0,32067571	NA	-1,66433243	0,4162362	NA	0,00021617	NA	NA	-0,08904476	8	-19344,7264	38705,4608	869,044071	6,38E-176
2378	-7,76791863	0,37580763	NA	NA	-0,00127907	NA	NA	-1,66433243	0,4162362	NA	0,00021617	NA	NA	-0,08904476	8	-19344,7895	38705,587	869,170263	5,99E-176
2828	-11,9654714	0,51367836	0,06255183	NA	-0,0013444	NA	NA	-1,2176888	NA	NA	0,00020569	-0,00045432	NA	-0,0941422	9	-19343,8115	38705,633	869,216249	5,86E-176
2467	-9,52399274	NA	0,02470059	NA	NA	NA	-0,56950148	-2,18775169	NA	0,01458917	0,00020109	NA	NA	-0,08533198	8	-19344,9992	38706,0064	869,589654	4,86E-176
404	-13,7669049	0,65178191	0,06462958	NA	NA	0,35119819	NA	-2,49206855	NA	0,01465098	0,00020366	NA	NA	NA	8	-19345,0041	38706,0161	869,59934	4,84E-176
2977	-8,58193391	NA	NA	NA	NA	NA	-0,61393687	-1,91049389	NA	0,01549989	0,00020133	-0,00049532	NA	-0,09486783	8	-19345,1543	38706,3166	869,899918	4,16E-176
396	-11,9654714	0,51367836	0,06255183	NA	-0,0013444	NA	NA	-2,49206855	NA	0,01465098	0,00020366	NA	NA	NA	8	-19345,1926	38706,3932	869,976483	4,01E-178
3010	-10,126072	0,56333515	NA	NA	NA	NA	NA	-2,61719209	0,45517288	0,01892094	0,00021164	9,21E-05	NA	-0,08713002	9	-19344,3058	38706,6216	870,204895	3,57E-176
2905	-7,52922856	NA	NA	NA	-0,00175017	0,32067571	NA	-1,73983563	0,43090563	NA	0,00021643	0,00010896	NA	-0,08710954	9	-19344,4367	38706,8833	870,466604	3,13E-176
2890	-7,76791863	0,37580763	NA	NA	-0,00127907	NA	NA	-1,73983563	0,43090563	NA	0,00021643	0,00010896	NA	-0,08710954	9	-19344,4998	38707,0095	870,592796	2,94E-176
1923	-11,7728913	NA	0,04392487	NA	NA	NA	NA	-2,58652225	NA	0,01772679	0,00020696	-0,00034396	0,48679163	NA	8	-19345,7935	38707,595	871,178286	2,20E-176
1345	-15,360872	NA	NA	NA	NA	NA	NA	-2,09152745	0,35956015	NA	0,00022076	NA	0,43622845	NA	6	-19348,6198	38709,2442	872,827518	9,63E-177
457	-6,18359995	NA	NA	NA	-0,00186372	NA	NA	-2,87228819	0,44207022	0,01904584	0,00021405	NA	NA	NA	7	-19347,7132	38709,4325	873,015819	8,76E-177
1857	-15,360872	NA	NA	NA	NA	NA	NA	-2,2103983	0,38500536	NA	0,00022113	0,00018745	0,43806177	NA	7	-19347,7457	38709,4977	873,080944	8,48E-177
2459	-11,3659105	NA	0,05744064	NA	-0,00198557	0,38445946	NA	-2,18775169	NA	0,01458917	0,00020109	NA	NA	-0,08533198	9	-19345,8247	38709,6594	873,242659	7,82E-177
3843	-11,7728913	NA	0,04392487	NA	NA	NA	NA	-1,25486087	NA	NA	0,00020772	-0,0003441	0,50313521	-0,14558566	8	-19347,1505	38710,309	873,892301	5,65E-177
969	-6,18359995	NA	NA	NA	-0,00186372	NA	NA	-2,9468992	0,4586198	0,01897353	0,00021429	0,00012376	NA	NA	8	-19347,3378	38710,6836	874,266831	4,69E-177
2851	-9,52399274	NA	0,02470059	NA	NA	NA	-0,56950148	-1,2176888	NA	NA	0,00020569	-0,00045432	NA	-0,0941422	8	-19347,408	38710,824	874,407324	4,37E-177
450	-10,126072	0,56333515	NA	NA	NA	NA	NA	-2,87228819	0,44207022	0,01904584	0,00021405	NA	NA	NA	7	-19348,4656	38710,9374	874,520717	4,13E-177
2386	-8,08714822	0,50972296	NA	NA	NA	0,2963004	NA	-1,66433243	0,4162362	NA	0,00021617	NA	NA	-0,08904476	8	-19347,5973	38711,2026	874,785833	3,62E-177
345	-7,52922856	NA	NA	NA	-0,00175017	0,32067571	NA	-1,97841134	0,41538406	NA	0,00021885	NA	NA	NA	7	-19348,7175	38711,4413	875,024566	3,21E-178
330	-7,76791863	0,37580763	NA	NA	-0,00127907	NA	NA	-1,97841134	0,41538406	NA	0,00021885	NA	NA	NA	7	-19348,7806	38711,5675	875,150758	3,01E-177
419	-9,52399274	NA	0,02470059	NA	NA	NA	-0,56950148	-2,49206855	NA	0,01465098	0,00020366	NA	NA	NA	7	-19348,7892	38711,5845	875,16778	2,99E-177
2387	-11,2963041	NA	0,04958538	NA	NA	0,45107925	NA	-1,66433243	0,4162362	NA	0,00021617	NA	NA	-0,08904476	8	-19347,973	38711,954	875,537238	2,48E-177
962	-10,126072	0,56333515	NA	NA	NA	NA	NA	-2,9468992	0,4586198	0,01897353	0,00021429	0,00012376	NA	NA	8	-19348,0902	38712,1885	875,771728	2,21E-177
788	-13,7669049	0,65178191	0,06462958	NA	NA	0,35119819	NA	-1,56810442	NA	NA	0,00020849	-0,0004232	NA	NA	8	-19348,1659	38712,3398	875,923092	2,05E-177
1411	-11,7728913	NA	0,04392487	NA	NA	NA	NA	-2,75826159	NA	0,01709531	0,00020664	NA	0,4949792	NA	7	-19349,182	38712,3702	875,95343	2,02E-177
857	-7,52922856	NA	NA	NA	-0,00175017	0,32067571	NA	-2,0661368	0,43415925	NA	0,00021911	0,00013912	NA	NA	8	-19348,2396	38712,4872	876,070472	1,90E-177
842	-7,76791863	0,37580763	NA	NA	-0,00127907	NA	NA	-2,0661368	0,43415925	NA	0,00021911	0,00013912	NA	NA	8	-19348,3027	38712,6134	876,196664	1,79E-177
2898	-8,08714822	0,50972296	NA	NA	NA	0,2963004	NA	-1,73983563	0,43090563	NA	0,00021643	0,00010896	NA	-0,08710954	9	-19347,3076	38712,6251	876,208365	1,78E-177
780	-11,9654714	0,51367836	0,06255183	NA	-0,0013444	NA	NA	-1,56810442	NA	NA	0,00020849	-0,0004232	NA	NA	8	-19348,3545	38712,717	876,300236	1,70E-179
2899	-11,2963041	NA	0,04958538	NA	NA	0,45107925	NA	-1,73983563	0,43090563	NA	0,00021643	0,00010896	NA	-0,08710954	9	-19347,6833	38713,3765	876,959771	1,22E-177
929	-8,58193391	NA	NA	NA	NA	NA	-0,61393687	-2,26386687	NA	0,01548996	0,00020413	-0,00046266	NA	NA	7	-19349,7519	38713,51	877,093291	1,14E-177
2843	-11,3659105	NA	0,05744064	NA	-0,00198557	0,38445946	NA	-1,2176888	NA	NA	0,00020569	-0,00045432	NA	-0,0941422	9	-19348,2335	38714,4771	878,060329	7,03E-178
2324	-13,7669049	0,65178191	0,06462958	NA	NA	0,35119819	NA	-1,50990804	NA	NA	0,00020523	NA	NA	-0,08563427	8	-19349,4682	38714,9445	878,527731	5,57E-178
3331	-11,7728913	NA	0,04392487	NA	NA	NA	NA	-1,4783871	NA	NA	0,00020739	NA	0,51055393	-0,13912561	7	-19350,5445	38715,0951	878,67838	5,16E-178
411	-11,3659105	NA	0,05744064	NA	-0,00198557	0,38445946	NA	-2,49206855	NA	0,01465098	0,00020366	NA	NA	NA	8	-19349,6147	38715,2373	878,820563	4,81E-178
2316	-11,9654714	0,51367836	0,06255183	NA	-0,0013444	NA	NA	-1,50990804	NA	NA	0,00020523	NA	NA	-0,08563427	8	-19349,6568	38715,3216	878,904874	4,61E-178

2948	-12,0135881	0,70944129	0,06178489	NA	NA	NA	NA	-1,91049389	NA	0,01549989	0,00020133	-0,00049532	NA	-0,09486783	9	-19349,4401	38716,8902	880,473514	2,10E-178
338	-8,08714822	0,50972296	NA	NA	NA	0,2963004	NA	-1,97841134	0,41538406	NA	0,00021885	NA	NA	NA	7	-19351,5884	38717,183	880,766327	1,82E-178
1937	-9,09446706	NA	NA	NA	NA	0,38722306	NA	-2,58652225	NA	0,01772679	0,00020696	-0,00034396	0,48679163	NA	8	-19350,9269	38717,8618	881,445077	1,29E-178
803	-9,52399274	NA	0,02470059	NA	NA	NA	-0,56950148	-1,56810442	NA	NA	0,00020849	-0,0004232	NA	NA	7	-19351,951	38717,9083	881,491532	1,27E-178
339	-11,2963041	NA	0,04958538	NA	NA	0,45107925	NA	-1,97841134	0,41538406	NA	0,00021885	NA	NA	NA	7	-19351,9641	38717,9345	881,517733	1,25E-178
2465	-8,58193391	NA	NA	NA	NA	NA	-0,61393687	-2,18775169	NA	0,01458917	0,00020109	NA	NA	-0,08533198	7	-19352,0153	38718,0368	881,620046	1,19E-178
850	-8,08714822	0,50972296	NA	NA	NA	0,2963004	NA	-2,0661368	0,43415925	NA	0,00021911	0,00013912	NA	NA	8	-19351,1105	38718,229	881,812234	1,08E-178
851	-11,2963041	NA	0,04958538	NA	NA	0,45107925	NA	-2,0661368	0,43415925	NA	0,00021911	0,00013912	NA	NA	8	-19351,4862	38718,9804	882,563639	7,40E-180
2339	-9,52399274	NA	0,02470059	NA	NA	NA	-0,56950148	-1,50990804	NA	NA	0,00020523	NA	NA	-0,08563427	7	-19353,2534	38720,5129	884,09617	3,44E-179
3857	-9,09446706	NA	NA	NA	NA	0,38722306	NA	-1,25486087	NA	NA	0,00020772	-0,0003441	0,50313521	-0,14558566	8	-19352,2839	38720,5758	884,159092	3,33E-179
276	-13,7669049	0,65178191	0,06462958	NA	NA	0,35119819	NA	-1,812439	NA	NA	0,00020783	NA	NA	NA	7	-19353,2928	38720,5917	884,174984	3,31E-179
268	-11,9654714	0,51367836	0,06255183	NA	-0,0013444	NA	NA	-1,812439	NA	NA	0,00020783	NA	NA	NA	7	-19353,4813	38720,9688	884,552127	2,74E-179
795	-11,3659105	NA	0,05744064	NA	-0,00198557	0,38445946	NA	-1,56810442	NA	NA	0,00020849	-0,0004232	NA	NA	8	-19352,7765	38721,561	885,144315	2,04E-179
1425	-9,09446706	NA	NA	NA	NA	0,38722306	NA	-2,75826159	NA	0,01709531	0,00020664	NA	0,4949792	NA	7	-19354,3154	38722,6369	886,220222	1,19E-179
2849	-8,58193391	NA	NA	NA	NA	NA	-0,61393687	-1,2176888	NA	NA	0,00020569	-0,00045432	NA	-0,0941422	7	-19354,4241	38722,8544	886,437716	1,07E-179
417	-8,58193391	NA	NA	NA	NA	NA	-0,61393687	-2,49206855	NA	0,01465098	0,00020366	NA	NA	NA	6	-19355,8052	38723,6151	887,198393	7,29E-180
900	-12,0135881	0,70944129	0,06178489	NA	NA	NA	NA	-2,26386687	NA	0,01548996	0,00020413	-0,00046266	NA	NA	8	-19354,0377	38724,0834	887,666665	5,77E-180
2331	-11,3659105	NA	0,05744064	NA	-0,00198557	0,38445946	NA	-1,50990804	NA	NA	0,00020523	NA	NA	-0,08563427	8	-19354,0789	38724,1657	887,748954	5,54E-180
3345	-9,09446706	NA	NA	NA	NA	0,38722306	NA	-1,4783871	NA	NA	0,00020739	NA	0,51055393	-0,13912561	7	-19355,6779	38725,3619	888,945171	3,05E-180
291	-9,52399274	NA	0,02470059	NA	NA	NA	-0,56950148	-1,812439	NA	NA	0,00020783	NA	NA	NA	6	-19357,0779	38726,1604	889,743645	2,04E-180
2436	-12,0135881	0,70944129	0,06178489	NA	NA	NA	NA	-2,18775169	NA	0,01458917	0,00020109	NA	NA	-0,08533198	8	-19356,3011	38728,6101	892,19342	6,00E-181
2377	-6,18359995	NA	NA	NA	-0,00186372	NA	NA	-1,66433243	0,4162362	NA	0,00021617	NA	NA	-0,08904476	7	-19357,6404	38729,2871	892,870345	4,28E-181
1795	-11,7728913	NA	0,04392487	NA	NA	NA	NA	-1,78635429	NA	NA	0,0002119	-0,00029974	0,47515083	NA	7	-19357,8323	38729,6708	893,254087	3,53E-181
283	-11,3659105	NA	0,05744064	NA	-0,00198557	0,38445946	NA	-1,812439	NA	NA	0,00020783	NA	NA	NA	7	-19357,9034	38729,8129	893,396207	3,29E-182
801	-8,58193391	NA	NA	NA	NA	NA	-0,61393687	-1,56810442	NA	NA	0,00020849	-0,0004232	NA	NA	6	-19358,9671	38729,9389	893,522146	3,09E-182
2889	-6,18359995	NA	NA	NA	-0,00186372	NA	NA	-1,73983563	0,43090563	NA	0,00021643	0,00010896	NA	-0,08710954	8	-19357,3507	38730,7094	894,292656	2,10E-181
2370	-10,126072	0,56333515	NA	NA	NA	NA	NA	-1,66433243	0,4162362	NA	0,00021617	NA	NA	-0,08904476	7	-19358,3929	38730,792	894,375242	2,02E-182
2882	-10,126072	0,56333515	NA	NA	NA	NA	NA	-1,73983563	0,43090563	NA	0,00021643	0,00010896	NA	-0,08710954	8	-19358,1032	38732,2143	895,797554	9,90E-182
2337	-8,58193391	NA	NA	NA	NA	NA	-0,61393687	-1,50990804	NA	NA	0,00020523	NA	NA	-0,08563427	6	-19360,2694	38732,5435	896,126784	8,40E-182
2955	-9,45532716	NA	0,05329757	NA	-0,00210061	NA	NA	-1,91049389	NA	0,01549989	0,00020133	-0,00049532	NA	-0,09486783	9	-19357,3193	38732,6486	896,231839	7,97E-182
1283	-11,7728913	NA	0,04392487	NA	NA	NA	NA	-1,96172815	NA	NA	0,00021146	NA	0,48287384	NA	6	-19360,4372	38732,879	896,462325	7,10E-182
2820	-12,0135881	0,70944129	0,06178489	NA	NA	NA	NA	-1,2176888	NA	NA	0,00020569	-0,00045432	NA	-0,0941422	8	-19358,7099	38733,4278	897,01109	5,40E-183
388	-12,0135881	0,70944129	0,06178489	NA	NA	NA	NA	-2,49206855	NA	0,01465098	0,00020366	NA	NA	NA	7	-19360,091	38734,1883	897,771546	3,69E-182
3969	-15,360872	NA	NA	NA	NA	NA	NA	-2,05206432	NA	0,01786019	0,00020271	-0,00039168	0,51529109	-0,14768431	8	-19359,1233	38734,2545	897,837812	3,57E-182
329	-6,18359995	NA	NA	NA	-0,00186372	NA	NA	-1,97841134	0,41538406	NA	0,00021885	NA	NA	NA	6	-19361,6316	38735,2678	898,851061	2,15E-182
2499	-11,7728913	NA	0,04392487	NA	NA	NA	NA	-2,55577604	0,44288659	0,01897716	0,00021141	NA	NA	-0,08883356	8	-19359,7139	38735,4357	899,018975	1,98E-182
841	-6,18359995	NA	NA	NA	-0,00186372	NA	NA	-2,0661368	0,43415925	NA	0,00021911	0,00013912	NA	NA	7	-19361,1536	38736,3135	899,896746	1,28E-182
322	-10,126072	0,56333515	NA	NA	NA	NA	NA	-1,97841134	0,41538406	NA	0,00021885	NA	NA	NA	6	-19362,384	38736,7727	900,355959	1,01E-182
3011	-11,7728913	NA	0,04392487	NA	NA	NA	NA	-2,61719209	0,45517288	0,01892094	0,00021164	9,21E-05	NA	-0,08713002	9	-19359,5083	38737,0266	900,609896	8,93E-183
834	-10,126072	0,56333515	NA	NA	NA	NA	NA	-2,0661368	0,43415925	NA	0,00021911	0,00013912	NA	NA	7	-19361,9061	38737,8184	901,401643	6,01E-183
289	-8,58193391	NA	NA	NA	NA	NA	-0,61393687	-1,812439	NA	NA	0,00020783	NA	NA	NA	5	-19364,0939	38738,1912	901,77448	4,99E-183
907	-9,45532716	NA	0,05329757	NA	-0,00210061	NA	NA	-2,26386687	NA	0,01548996	0,00020413	-0,00046266	NA	NA	8	-19361,9169	38739,8417	903,42499	2,18E-183
1809	-9,09446706	NA	NA	NA	NA	0,38722306	NA	-1,78635429	NA	NA	0,0002119	-0,00029974	0,47515083	NA	7	-19362,9657	38739,9376	903,520879	2,08E-183
772	-12,0135881	0,70944129	0,06178489	NA	NA	NA	NA	-1,56810442	NA	NA	0,00020849	-0,0004232	NA	NA	7	-19363,2529	38740,512	904,095298	1,56E-183
3457	-15,360872	NA	NA	NA	NA	NA	NA	-2,27275384	NA	0,01711404	0,00020255	NA	0,52288611	-0,14000571	7	-19363,464	38740,9342	904,51748	1,27E-183
451	-11,7728913	NA	0,04392487	NA	NA	NA	NA	-2,87228819	0,44207022	0,01904584	0,00021405	NA	NA	NA	7	-19363,6681	38741,3424	904,925719	1,03E-183
963	-11,7728913	NA	0,04392487	NA	NA	NA	NA	-2,9468992	0,4586198	0,01897353	0,00021429	0,00012376	NA	NA	8	-19363,2927	38742,5935	906,17673	5,52E-184
2308	-12,0135881	0,70944129	0,06178489	NA	NA	NA	NA	-1,50990804	NA	NA	0,00020523	NA	NA	-0,08563427	7	-19364,5552	38743,1167	906,699936	4,25E-184
1297	-9,09446706	NA	NA	NA	NA	0,38722306	NA	-1,96172815	NA	NA	0,00021146	NA	0,48287384	NA	6	-19365,5706	38743,1458	906,729117	4,19E-184
2443	-9,45532716	NA	0,05329757	NA	-0,00210061	NA	NA	-2,18775169	NA	0,01458917	0,00020109	NA	NA	-0,08533198	8	-19364,1803	38744,3685	907,951745	2,27E-184
2513	-9,09446706	NA	NA	NA	NA	0,38722306	NA	-2,55577604	0,44288659	0,01897716	0,00021141	NA	NA	-0,08883356	8	-19364,8473	38745,7025	909,285767	1,17E-184
3025	-9,09446706	NA	NA	NA	NA	0,38722306	NA	-2,61719209	0,45517288	0,01892094	0,00021164	9,21E-05	NA	-0,08713002	9	-19364,6417	38747,2934	910,876688	5,26E-185
260	-12,0135881	0,70944129	0,06178489	NA	NA	NA	NA	-1,812439	NA	NA	0,00020783	NA	NA	NA	6	-19368,3797	38748,7641	912,347411	2,52E-185
2827	-9,45532716	NA	0,05329757	NA	-0,00210061	NA	NA	-1,2176888	NA	NA	0,00020569	-0,00045432	NA	-0,0941422	8	-19366,5891	38749,1861	912,769415	2,04E-186
395	-9,45532716	NA	0,05329757	NA	-0,00210061	NA	NA	-2,49206855	NA	0,01465098	0,00020366	NA	NA	NA	7	-19367,9702	38749,9466	913,529871	1,40E-185
465	-9,09446706	NA	NA	NA	NA	0,38722306	NA	-2,87228819	0,44207022	0,01904584	0,00021405	NA	NA	NA	7	-19368,8015	38751,6092	915,19251	6,08E-186
977	-9,09446706	NA	NA	NA	NA	0,38722306	NA	-2,9468992	0,4586198	0,01897353	0,00021429	0,00012376	NA	NA	8	-19368,4261	38752,8602	916,443522	3,25E-187
1921	-15,360872	NA	NA	NA	NA	NA	NA	-2,58652225	NA	0,01772679	0,00020696	-0,00034396	0,48679163	NA	7	-19370,0691	38754,1443	917,727583	1,71E-186
779	-9,45532716	NA	0,05329757	NA	-0,00210061	NA	NA	-1,56810442	NA	NA	0,00020849	-0,0004232	NA	NA	7	-19371,1321	38756,2703	919,853623	5,91E-187
3841	-15,360872	NA	NA	NA	NA	NA	NA	-1,25486087	NA	NA	0,00020772	-0,0003441	0,50313521	-0,14558566	7	-19371,4261	38756,8583	920,441598	4,41E-187
2970	-8,97566397	0,33138274	NA	NA	-0,0012458	0,28352203	NA	-1,91049389	NA	0,01549989	0,00020133	-0,00049532	NA	-0,09486783	10	-19369,1735	38758,3591	921,942416	2,08E-187

2315	-9,45532716	NA	0,05329757	NA	-0,00210061	NA	NA	-1,50990804	NA	NA	0,00020523	NA	NA	-0,08563427	7	-19372,4344	38758,875	922,458261	1,61E-187
1409	-15,360872	NA	NA	NA	NA	NA	NA	-2,75826159	NA	0,01709531	0,00020664	NA	0,4949792	NA	6	-19373,4575	38758,9197	922,502949	1,57E-187
2371	-11,7728913	NA	0,04392487	NA	NA	NA	NA	-1,66433243	0,4162362	NA	0,00021617	NA	NA	-0,08904476	7	-19373,5954	38761,197	924,780244	5,04E-188
3329	-15,360872	NA	NA	NA	NA	NA	NA	-1,4783871	NA	NA	0,00020739	NA	0,51055393	-0,13912561	6	-19374,82	38761,6446	925,227899	4,03E-188
2883	-11,7728913	NA	0,04392487	NA	NA	NA	NA	-1,73983563	0,43090563	NA	0,00021643	0,00010896	NA	-0,08710954	8	-19373,3057	38762,6193	926,202555	2,47E-188
267	-9,45532716	NA	0,05329757	NA	-0,00210061	NA	NA	-1,812439	NA	NA	0,00020783	NA	NA	NA	6	-19376,2589	38764,5225	928,105736	9,55E-189
922	-8,97566397	0,33138274	NA	NA	-0,0012458	0,28352203	NA	-2,26386687	NA	0,01548996	0,00020413	-0,00046266	NA	NA	9	-19373,7711	38765,5521	929,135346	5,71E-189
323	-11,7728913	NA	0,04392487	NA	NA	NA	NA	-1,97841134	0,41538406	NA	0,00021885	NA	NA	NA	6	-19377,5865	38767,1777	930,76096	2,53E-189
835	-11,7728913	NA	0,04392487	NA	NA	NA	NA	-2,0661368	0,43415925	NA	0,00021911	0,00013912	NA	NA	7	-19377,1086	38768,2234	931,806645	1,50E-189
2458	-8,97566397	0,33138274	NA	NA	-0,0012458	0,28352203	NA	-2,18775169	NA	0,01458917	0,00020109	NA	NA	-0,08533198	9	-19376,0344	38770,0788	933,662101	5,94E-190
2385	-9,09446706	NA	NA	NA	NA	0,38722306	NA	-1,66433243	0,4162362	NA	0,00021617	NA	NA	-0,08904476	7	-19378,7288	38771,4638	935,047036	2,97E-191
2897	-9,09446706	NA	NA	NA	NA	0,38722306	NA	-1,73983563	0,43090563	NA	0,00021643	0,00010896	NA	-0,08710954	8	-19378,4391	38772,8861	936,469347	1,46E-190
2842	-8,97566397	0,33138274	NA	NA	-0,0012458	0,28352203	NA	-1,2176888	NA	NA	0,00020569	-0,00045432	NA	-0,0941422	9	-19378,4433	38774,8965	938,479771	5,34E-191
410	-8,97566397	0,33138274	NA	NA	-0,0012458	0,28352203	NA	-2,49206855	NA	0,01465098	0,00020366	NA	NA	NA	8	-19379,8244	38775,6567	939,240005	3,65E-191
2969	-7,52922856	NA	NA	NA	-0,00175017	0,32067571	NA	-1,91049389	NA	0,01549989	0,00020133	-0,00049532	NA	-0,09486783	9	-19378,999	38776,0079	939,591194	3,06E-191
2954	-7,76791863	0,37580763	NA	NA	-0,00127907	NA	NA	-1,91049389	NA	0,01549989	0,00020133	-0,00049532	NA	-0,09486783	9	-19379,0621	38776,1341	939,717386	2,87E-191
1793	-15,360872	NA	NA	NA	NA	NA	NA	-1,78635429	NA	NA	0,0002119	-0,00029974	0,47515083	NA	6	-19382,1078	38776,2203	939,803606	2,75E-191
337	-9,09446706	NA	NA	NA	NA	0,38722306	NA	-1,97841134	0,41538406	NA	0,00021885	NA	NA	NA	6	-19382,7199	38777,4445	941,027752	1,49E-191
849	-9,09446706	NA	NA	NA	NA	0,38722306	NA	-2,0661368	0,43415925	NA	0,00021911	0,00013912	NA	NA	7	-19382,242	38778,4902	942,073437	8,85E-192
1281	-15,360872	NA	NA	NA	NA	NA	NA	-1,96172815	NA	NA	0,00021146	NA	0,48287384	NA	5	-19384,7127	38779,4288	943,012066	5,54E-192
2962	-8,08714822	0,50972296	NA	NA	NA	0,2963004	NA	-1,91049389	NA	0,01549989	0,00020133	-0,00049532	NA	-0,09486783	9	-19381,8699	38781,7497	945,332956	1,73E-192
794	-8,97566397	0,33138274	NA	NA	-0,0012458	0,28352203	NA	-1,56810442	NA	NA	0,00020849	-0,0004232	NA	NA	8	-19382,9863	38781,9805	945,563758	1,55E-192
2497	-15,360872	NA	NA	NA	NA	NA	NA	-2,55577604	0,44288659	0,01897716	0,00021141	NA	NA	-0,08883356	7	-19383,9894	38781,985	945,568272	1,54E-192
2963	-11,2963041	NA	0,04958538	NA	NA	0,45107925	NA	-1,91049389	NA	0,01549989	0,00020133	-0,00049532	NA	-0,09486783	9	-19382,2456	38782,5011	946,084361	1,19E-192
921	-7,52922856	NA	NA	NA	-0,00175017	0,32067571	NA	-2,26386687	NA	0,01548996	0,00020413	-0,00046266	NA	NA	8	-19383,5966	38783,2011	946,784346	8,39E-193
906	-7,76791863	0,37580763	NA	NA	-0,00127907	NA	NA	-2,26386687	NA	0,01548996	0,00020413	-0,00046266	NA	NA	8	-19383,6596	38783,3273	946,910537	7,88E-193
3009	-15,360872	NA	NA	NA	NA	NA	NA	-2,61719209	0,45517288	0,01892094	0,00021164	9,21E-05	NA	-0,08713002	8	-19383,7839	38783,5757	947,158972	6,96E-193
2330	-8,97566397	0,33138274	NA	NA	-0,0012458	0,28352203	NA	-1,50990804	NA	NA	0,00020523	NA	NA	-0,08563427	8	-19384,2886	38784,5851	948,168396	4,20E-193
2457	-7,52922856	NA	NA	NA	-0,00175017	0,32067571	NA	-2,18775169	NA	0,01458917	0,00020109	NA	NA	-0,08533198	8	-19385,8599	38787,7278	951,311101	8,73E-194
2442	-7,76791863	0,37580763	NA	NA	-0,00127907	NA	NA	-2,18775169	NA	0,01458917	0,00020109	NA	NA	-0,08533198	8	-19385,923	38787,854	951,437293	8,20E-194
449	-15,360872	NA	NA	NA	NA	NA	NA	-2,87228819	0,44207022	0,01904584	0,00021405	NA	NA	NA	6	-19387,9437	38787,892	951,475237	8,04E-194
914	-8,08714822	0,50972296	NA	NA	NA	0,2963004	NA	-2,26386687	NA	0,01548996	0,00020413	-0,00046266	NA	NA	8	-19386,4674	38788,9428	952,526107	4,76E-194
961	-15,360872	NA	NA	NA	NA	NA	NA	-2,9468992	0,4586198	0,01897353	0,00021429	0,00012376	NA	NA	7	-19387,5683	38789,1427	952,726027	4,30E-194
915	-11,2963041	NA	0,04958538	NA	NA	0,45107925	NA	-2,26386687	NA	0,01548996	0,00020413	-0,00046266	NA	NA	8	-19386,8431	38789,6942	953,277512	3,27E-194
282	-8,97566397	0,33138274	NA	NA	-0,0012458	0,28352203	NA	-1,812439	NA	NA	0,00020783	NA	NA	NA	7	-19388,1131	38790,2324	953,815649	2,50E-194
2841	-7,52922856	NA	NA	NA	-0,00175017	0,32067571	NA	-1,2176888	NA	NA	0,00020569	-0,00045432	NA	-0,0941422	8	-19388,2688	38792,5455	956,128771	7,85E-195
2826	-7,76791863	0,37580763	NA	NA	-0,00127907	NA	NA	-1,2176888	NA	NA	0,00020569	-0,00045432	NA	-0,0941422	8	-19388,3319	38792,6717	956,254963	7,37E-195
409	-7,52922856	NA	NA	NA	-0,00175017	0,32067571	NA	-2,49206855	NA	0,01465098	0,00020366	NA	NA	NA	7	-19389,6499	38793,3059	956,889226	5,37E-195
394	-7,76791863	0,37580763	NA	NA	-0,00127907	NA	NA	-2,49206855	NA	0,01465098	0,00020366	NA	NA	NA	7	-19389,713	38793,4321	957,015418	5,04E-195
2450	-8,08714822	0,50972296	NA	NA	NA	0,2963004	NA	-2,18775169	NA	0,01458917	0,00020109	NA	NA	-0,08533198	8	-19388,7308	38793,4696	957,052862	4,95E-195
2451	-11,2963041	NA	0,04958538	NA	NA	0,45107925	NA	-2,18775169	NA	0,01458917	0,00020109	NA	NA	-0,08533198	8	-19389,1065	38794,221	957,804268	3,40E-195
2834	-8,08714822	0,50972296	NA	NA	NA	0,2963004	NA	-1,2176888	NA	NA	0,00020569	-0,00045432	NA	-0,0941422	8	-19391,1396	38798,2873	961,870532	4,45E-196
2835	-11,2963041	NA	0,04958538	NA	NA	0,45107925	NA	-1,2176888	NA	NA	0,00020569	-0,00045432	NA	-0,0941422	8	-19391,5153	38799,0387	962,621938	3,05E-197
402	-8,08714822	0,50972296	NA	NA	NA	0,2963004	NA	-2,49206855	NA	0,01465098	0,00020366	NA	NA	NA	7	-19392,5208	38799,0477	962,630988	3,04E-196
793	-7,52922856	NA	NA	NA	-0,00175017	0,32067571	NA	-1,56810442	NA	NA	0,00020849	-0,0004232	NA	NA	7	-19392,8118	38799,6297	963,212979	2,27E-196
778	-7,76791863	0,37580763	NA	NA	-0,00127907	NA	NA	-1,56810442	NA	NA	0,00020849	-0,0004232	NA	NA	7	-19392,8749	38799,7559	963,339171	2,13E-196
403	-11,2963041	NA	0,04958538	NA	NA	0,45107925	NA	-2,49206855	NA	0,01465098	0,00020366	NA	NA	NA	7	-19392,8965	38799,7991	963,382393	2,09E-196
2953	-6,18359995	NA	NA	NA	-0,00186372	NA	NA	-1,91049389	NA	0,01549989	0,00020133	-0,00049532	NA	-0,09486783	8	-19391,913	38799,834	963,417247	2,05E-196
2946	-10,126072	0,56333515	NA	NA	NA	NA	NA	-1,91049389	NA	0,01549989	0,00020133	-0,00049532	NA	-0,09486783	8	-19392,6655	38801,3389	964,922144	9,67E-197
2329	-7,52922856	NA	NA	NA	-0,00175017	0,32067571	NA	-1,50990804	NA	NA	0,00020523	NA	NA	-0,08563427	7	-19394,1141	38802,2343	965,817617	6,18E-197
2314	-7,76791863	0,37580763	NA	NA	-0,00127907	NA	NA	-1,50990804	NA	NA	0,00020523	NA	NA	-0,08563427	7	-19394,1772	38802,3605	965,943809	5,80E-197
786	-8,08714822	0,50972296	NA	NA	NA	0,2963004	NA	-1,56810442	NA	NA	0,00020849	-0,0004232	NA	NA	7	-19395,6826	38805,3715	968,95474	1,29E-198
787	-11,2963041	NA	0,04958538	NA	NA	0,45107925	NA	-1,56810442	NA	NA	0,00020849	-0,0004232	NA	NA	7	-19396,0583	38806,1229	969,706145	8,84E-198
905	-6,18359995	NA	NA	NA	-0,00186372	NA	NA	-2,26386687	NA	0,01548996	0,00020413	-0,00046266	NA	NA	7	-19396,5106	38807,0273	970,610619	5,63E-198
2369	-15,360872	NA	NA	NA	NA	NA	NA	-1,66433243	0,4162362	NA	0,00021617	NA	NA	-0,08904476	6	-19397,8709	38807,7465	971,329763	3,93E-199
281	-7,52922856	NA	NA	NA	-0,00175017	0,32067571	NA	-1,812439	NA	NA	0,00020783	NA	NA	NA	6	-19397,9386	38807,8818	971,465091	3,67E-198
2322	-8,08714822	0,50972296	NA	NA	NA	0,2963004	NA	-1,50990804	NA	NA	0,00020523	NA	NA	-0,08563427	7	-19396,985	38807,9761	971,559378	3,50E-198
266	-7,76791863	0,37580763	NA	NA	-0,00127907	NA	NA	-1,812439	NA	NA	0,00020783	NA	NA	NA	6	-19398,0017	38808,008	971,591283	3,45E-198
898	-10,126072	0,56333515	NA	NA	NA	NA	NA	-2,26386687	NA	0,01548996	0,00020413	-0,00046266	NA	NA	7	-19397,263	38808,5322	972,115517	2,65E-198
2323	-11,2963041	NA	0,04958538	NA	NA	0,45107925	NA	-1,50990804	NA	NA	0,00020523	NA	NA	-0,08563427	7	-19397,3607	38808,7275	972,310784	2,40E-198
2881	-15,360872	NA	NA	NA	NA	NA	NA	-1,73983563	0,43090563	NA	0,00021643	0,00010896	NA	-0,08710954	7	-19397,5812	38809,1686	972,751853	1,93E-198

2441	-6,18359995	NA	NA	NA	-0,00186372	NA	NA	-2,18775169	NA	0,01458917	0,00020109	NA	NA	-0,08533198	7	-19398,774	38811,5541	975,137375	5,85E-199
2434	-10,126072	0,56333515	NA	NA	NA	NA	NA	-2,18775169	NA	0,01458917	0,00020109	NA	NA	-0,08533198	7	-19399,5264	38813,059	976,642272	2,76E-199
274	-8,08714822	0,50972296	NA	NA	NA	0,2963004	NA	-1,812439	NA	NA	0,00020783	NA	NA	NA	6	-19400,8095	38813,6236	977,206853	2,08E-199
321	-15,360872	NA	NA	NA	NA	NA	NA	-1,97841134	0,41538406	NA	0,00021885	NA	NA	NA	5	-19401,8621	38813,7274	977,310701	1,97E-199
275	-11,2963041	NA	0,04958538	NA	NA	0,45107925	NA	-1,812439	NA	NA	0,00020783	NA	NA	NA	6	-19401,1852	38814,375	977,958258	1,43E-199
833	-15,360872	NA	NA	NA	NA	NA	NA	-2,0661368	0,43415925	NA	0,00021911	0,00013912	NA	NA	6	-19401,3841	38814,7729	978,356164	1,17E-199
2825	-6,18359995	NA	NA	NA	-0,00186372	NA	NA	-1,2176888	NA	NA	0,00020569	-0,00045432	NA	-0,0941422	7	-19401,1828	38816,3718	979,955045	5,26E-200
393	-6,18359995	NA	NA	NA	-0,00186372	NA	NA	-2,49206855	NA	0,01465098	0,00020366	NA	NA	NA	6	-19402,5639	38817,1324	980,715722	3,60E-201
2818	-10,126072	0,56333515	NA	NA	NA	NA	NA	-1,2176888	NA	NA	0,00020569	-0,00045432	NA	-0,0941422	7	-19401,9352	38817,8767	981,459942	2,48E-200
386	-10,126072	0,56333515	NA	NA	NA	NA	NA	-2,49206855	NA	0,01465098	0,00020366	NA	NA	NA	6	-19403,3163	38818,6373	982,220619	1,69E-200
777	-6,18359995	NA	NA	NA	-0,00186372	NA	NA	-1,56810442	NA	NA	0,00020849	-0,0004232	NA	NA	6	-19405,7258	38823,4562	987,039474	1,52E-201
770	-10,126072	0,56333515	NA	NA	NA	NA	NA	-1,56810442	NA	NA	0,00020849	-0,0004232	NA	NA	6	-19406,4782	38824,9611	988,544371	7,18E-202
2313	-6,18359995	NA	NA	NA	-0,00186372	NA	NA	-1,50990804	NA	NA	0,00020523	NA	NA	-0,08563427	6	-19407,0281	38826,0608	989,644112	4,14E-202
2306	-10,126072	0,56333515	NA	NA	NA	NA	NA	-1,50990804	NA	NA	0,00020523	NA	NA	-0,08563427	6	-19407,7805	38827,5657	991,14901	1,95E-202
265	-6,18359995	NA	NA	NA	-0,00186372	NA	NA	-1,812439	NA	NA	0,00020783	NA	NA	NA	5	-19410,8526	38831,7085	995,291808	2,46E-203
2947	-11,7728913	NA	0,04392487	NA	NA	NA	NA	-1,91049389	NA	0,01549989	0,00020133	-0,00049532	NA	-0,09486783	8	-19407,868	38831,7439	995,327146	2,42E-203
258	-10,126072	0,56333515	NA	NA	NA	NA	NA	-1,812439	NA	NA	0,00020783	NA	NA	NA	5	-19411,6051	38833,2134	996,796706	1,16E-203
899	-11,7728913	NA	0,04392487	NA	NA	NA	NA	-2,26386687	NA	0,01548996	0,00020413	-0,00046266	NA	NA	7	-19412,4655	38838,9372	1002,52052	6,62E-205
2961	-9,09446706	NA	NA	NA	NA	0,38722306	NA	-1,91049389	NA	0,01549989	0,00020133	-0,00049532	NA	-0,09486783	8	-19413,0013	38842,0107	1005,59394	1,42E-205
2435	-11,7728913	NA	0,04392487	NA	NA	NA	NA	-2,18775169	NA	0,01458917	0,00020109	NA	NA	-0,08533198	7	-19414,7289	38843,464	1007,04727	6,89E-206
2819	-11,7728913	NA	0,04392487	NA	NA	NA	NA	-1,2176888	NA	NA	0,00020569	-0,00045432	NA	-0,0941422	7	-19417,1377	38848,2817	1011,86494	6,19E-207
387	-11,7728913	NA	0,04392487	NA	NA	NA	NA	-2,49206855	NA	0,01465098	0,00020366	NA	NA	NA	6	-19418,5189	38849,0423	1012,62562	4,23E-208
913	-9,09446706	NA	NA	NA	NA	0,38722306	NA	-2,26386687	NA	0,01548996	0,00020413	-0,00046266	NA	NA	7	-19417,5989	38849,204	1012,78731	3,91E-207
2449	-9,09446706	NA	NA	NA	NA	0,38722306	NA	-2,18775169	NA	0,01458917	0,00020109	NA	NA	-0,08533198	7	-19419,8623	38853,7308	1017,31407	4,06E-208
771	-11,7728913	NA	0,04392487	NA	NA	NA	NA	-1,56810442	NA	NA	0,00020849	-0,0004232	NA	NA	6	-19421,6807	38855,3661	1018,94937	1,79E-208
2307	-11,7728913	NA	0,04392487	NA	NA	NA	NA	-1,50990804	NA	NA	0,00020523	NA	NA	-0,08563427	6	-19422,983	38857,9707	1021,55401	4,88E-209
2833	-9,09446706	NA	NA	NA	NA	0,38722306	NA	-1,2176888	NA	NA	0,00020569	-0,00045432	NA	-0,0941422	7	-19422,2711	38858,5485	1022,13174	3,65E-209
401	-9,09446706	NA	NA	NA	NA	0,38722306	NA	-2,49206855	NA	0,01465098	0,00020366	NA	NA	NA	6	-19423,6522	38859,3091	1022,89241	2,50E-209
259	-11,7728913	NA	0,04392487	NA	NA	NA	NA	-1,812439	NA	NA	0,00020783	NA	NA	NA	5	-19426,8076	38863,6184	1027,20171	2,89E-210
785	-9,09446706	NA	NA	NA	NA	0,38722306	NA	-1,56810442	NA	NA	0,00020849	-0,0004232	NA	NA	6	-19426,8141	38865,6329	1029,21617	1,06E-210
2321	-9,09446706	NA	NA	NA	NA	0,38722306	NA	-1,50990804	NA	NA	0,00020523	NA	NA	-0,08563427	6	-19428,1164	38868,2375	1031,8208	2,87E-211
273	-9,09446706	NA	NA	NA	NA	0,38722306	NA	-1,812439	NA	NA	0,00020783	NA	NA	NA	5	-19431,941	38873,8852	1037,4685	1,71E-213
2945	-15,360872	NA	NA	NA	NA	NA	NA	-1,91049389	NA	0,01549989	0,00020133	-0,00049532	NA	-0,09486783	7	-19432,1435	38878,2932	1041,87644	1,88E-214
897	-15,360872	NA	NA	NA	NA	NA	NA	-2,26386687	NA	0,01548996	0,00020413	-0,00046266	NA	NA	6	-19436,7411	38885,4868	1049,07004	5,16E-215
2433	-15,360872	NA	NA	NA	NA	NA	NA	-2,18775169	NA	0,01458917	0,00020109	NA	NA	-0,08533198	6	-19439,0044	38890,0135	1053,59679	5,37E-216
2817	-15,360872	NA	NA	NA	NA	NA	NA	-1,2176888	NA	NA	0,00020569	-0,00045432	NA	-0,0941422	6	-19441,4133	38894,8312	1058,41446	4,83E-217
385	-15,360872	NA	NA	NA	NA	NA	NA	-2,49206855	NA	0,01465098	0,00020366	NA	NA	NA	5	-19442,7944	38895,5921	1059,17536	3,30E-217
769	-15,360872	NA	NA	NA	NA	NA	NA	-1,56810442	NA	NA	0,00020849	-0,0004232	NA	NA	5	-19445,9563	38901,9158	1065,49911	1,40E-218
2305	-15,360872	NA	NA	NA	NA	NA	NA	-1,50990804	NA	NA	0,00020523	NA	NA	-0,08563427	5	-19447,2586	38904,5205	1068,10375	3,80E-219
257	-15,360872	NA	NA	NA	NA	NA	NA	-1,812439	NA	NA	0,00020783	NA	NA	NA	4	-19451,0831	38910,1684	1073,75167	2,26E-220
3838	-0,06671637	0,57575795	NA	4,34E-05	-0,00071955	0,41051829	-0,17478186	-1,61532922	-0,10289105	0,0570521	NA	-0,00065333	0,49801214	-0,5357095	13	-20495,3235	41016,6672	3180,25048	0
3840	-0,47166397	0,58945108	0,00876766	4,25E-05	-0,00073797	0,4135805	-0,1717464	-1,61532922	-0,10289105	0,0570521	NA	-0,00065333	0,49801214	-0,5357095	14	-20494,3493	41016,7219	3180,3052	0
3774	-0,06671637	0,57575795	NA	4,34E-05	-0,00071955	0,41051829	-0,17478186	-1,76635397	NA	0,05800383	NA	-0,00051675	0,48731517	-0,53620329	12	-20498,3677	41020,7526	3184,33591	0
3776	-0,47166397	0,58945108	0,00876766	4,25E-05	-0,00073797	0,4135805	-0,1717464	-1,76635397	NA	0,05800383	NA	-0,00051675	0,48731517	-0,53620329	13	-20497,3935	41020,8071	3184,39041	0
3830	-0,67953035	0,68234123	NA	4,46E-05	NA	0,41541375	-0,13795142	-1,61532922	-0,10289105	0,0570521	NA	-0,00065333	0,49801214	-0,5357095	12	-20500,5101	41025,0375	3188,62083	0
3832	-1,04079715	0,69637088	0,00755047	4,39E-05	NA	0,41816409	-0,13461471	-1,61532922	-0,10289105	0,0570521	NA	-0,00065333	0,49801214	-0,5357095	13	-20499,7909	41025,6019	3189,18521	0
3808	-1,28042467	0,61751101	0,00965662	4,55E-05	-0,00054807	0,40424516	NA	-1,61532922	-0,10289105	0,0570521	NA	-0,00065333	0,49801214	-0,5357095	13	-20500,5956	41027,2113	3190,79454	0
3806	-0,85074914	0,60334436	NA	4,65E-05	-0,00052401	0,40049867	NA	-1,61532922	-0,10289105	0,0570521	NA	-0,00065333	0,49801214	-0,5357095	12	-20501,7714	41027,5601	3191,14334	0
3766	-0,67953035	0,68234123	NA	4,46E-05	NA	0,41541375	-0,13795142	-1,76635397	NA	0,05800383	NA	-0,00051675	0,48731517	-0,53620329	11	-20503,5543	41029,1232	3192,70648	0
3768	-1,04079715	0,69637088	0,00755047	4,39E-05	NA	0,41816409	-0,13461471	-1,76635397	NA	0,05800383	NA	-0,00051675	0,48731517	-0,53620329	12	-20502,8351	41029,6874	3193,27065	0
3744	-1,28042467	0,61751101	0,00965662	4,55E-05	-0,00054807	0,40424516	NA	-1,76635397	NA	0,05800383	NA	-0,00051675	0,48731517	-0,53620329	12	-20503,6397	41031,2967	3194,87998	0
3798	-1,1987332	0,68175498	NA	4,69E-05	NA	0,40581671	NA	-1,61532922	-0,10289105	0,0570521	NA	-0,00065333	0,49801214	-0,5357095	11	-20504,6853	41031,3852	3194,96844	0
3800	-1,59179834	0,69737855	0,00852736	4,60E-05	NA	0,40932129	NA	-1,61532922	-0,10289105	0,0570521	NA	-0,00065333	0,49801214	-0,5357095	12	-20503,7689	41031,555	3195,13825	0
3742	-0,85074914	0,60334436	NA	4,65E-05	-0,00052401	0,40049867	NA	-1,76635397	NA	0,05800383	NA	-0,00051675	0,48731517	-0,53620329	11	-20504,8156	41031,6457	3195,22899	0
3734	-1,1987332	0,68175498	NA	4,69E-05	NA	0,40581671	NA	-1,76635397	NA	0,05800383	NA	-0,00051675	0,48731517	-0,53620329	10	-20507,7294	41035,471	3199,05431	0
3736	-1,59179834	0,69737855	0,00852736	4,60E-05	NA	0,40932129	NA	-1,76635397	NA	0,05800383	NA	-0,00051675	0,48731517	-0,53620329	11	-20506,813	41035,6406	3199,2239	0
3262	-0,06671637	0,57575795	NA	4,34E-05	-0,00071955	0,41051829	-0,17478186	-2,06058113	NA	0,05699765	NA	NA	0,49693597	-0,52441365	11	-20507,6124	41037,2394	3200,82269	0
3264	-0,47166397	0,58945108	0,00876766	4,25E-05	-0,00073797	0,4135805	-0,1717464	-2,06058113	NA	0,05699765	NA	NA	0,49693597	-0,52441365	12	-20506,6382	41037,2937	3200,87697	0
3326	-0,06671637	0,57575795	NA	4,34E-05	-0,00071955	0,41051829	-0,17478186	-2,04753199	-0,01846226	0,05678211	NA	NA	0,49927467	-0,52377486	12	-20507,4947	41039,0066	3202,58993	0
3328	-0,47166397	0,58945108	0,00876766	4,25E-05	-0,00073797	0,4135805	-0,1717464	-2,04753199	-0,01846226	0,05678211	NA	NA	0,49927467	-0,52377486	13	-20506,5205	41039,0611	3202,64443	0
3254	-0,67953035	0,68234123	NA	4,46E-05	NA	0,41541375	-0,13795142	-2,06058113	NA	0,05699765	NA	NA	0,49693597	-0,52441365	10	-20512,799	41045,6102	3209,19348	0

3256	-1,04079715	0,69637088	0,00755047	4,39E-05	NA	0,41816409	-0,13461471	-2,06058113	NA	0,05699765	NA	NA	0,49693597	-0,52441365	11	-20512,0798	41046,1741	3209,75743	0
3318	-0,67953035	0,68234123	NA	4,46E-05	NA	0,41541375	-0,13795142	-2,04753199	-0,01846226	0,05678211	NA	NA	0,49927467	-0,52377486	11	-20512,6813	41047,3772	3210,9605	0
3232	-1,28042467	0,61751101	0,00965662	4,55E-05	-0,00054807	0,40424516	NA	-2,06058113	NA	0,05699765	NA	NA	0,49693597	-0,52441365	11	-20512,8844	41047,7835	3211,36676	0
3320	-1,04079715	0,69637088	0,00755047	4,39E-05	NA	0,41816409	-0,13461471	-2,04753199	-0,01846226	0,05678211	NA	NA	0,49927467	-0,52377486	12	-20511,9621	41047,9414	3211,52466	0
3230	-0,85074914	0,60334436	NA	4,65E-05	-0,00052401	0,40049867	NA	-2,06058113	NA	0,05699765	NA	NA	0,49693597	-0,52441365	10	-20514,0603	41048,1327	3211,71599	0
3296	-1,28042467	0,61751101	0,00965662	4,55E-05	-0,00054807	0,40424516	NA	-2,04753199	-0,01846226	0,05678211	NA	NA	0,49927467	-0,52377486	12	-20512,7667	41049,5507	3213,13399	0
3294	-0,85074914	0,60334436	NA	4,65E-05	-0,00052401	0,40049867	NA	-2,04753199	-0,01846226	0,05678211	NA	NA	0,49927467	-0,52377486	11	-20513,9426	41049,8997	3213,48301	0
3222	-1,1987332	0,68175498	NA	4,69E-05	NA	0,40581671	NA	-2,06058113	NA	0,05699765	NA	NA	0,49693597	-0,52441365	9	-20516,9742	41051,9583	3215,54154	0
3224	-1,59179834	0,69737855	0,00852736	4,60E-05	NA	0,40932129	NA	-2,06058113	NA	0,05699765	NA	NA	0,49693597	-0,52441365	10	-20516,0577	41052,1276	3215,71091	0
3286	-1,1987332	0,68175498	NA	4,69E-05	NA	0,40581671	NA	-2,04753199	-0,01846226	0,05678211	NA	NA	0,49927467	-0,52377486	10	-20516,8564	41053,7251	3217,30833	0
3288	-1,59179834	0,69737855	0,00852736	4,60E-05	NA	0,40932129	NA	-2,04753199	-0,01846226	0,05678211	NA	NA	0,49927467	-0,52377486	11	-20515,94	41053,8946	3217,47792	0
3822	-0,14195779	0,65649184	NA	4,36E-05	-0,00074998	NA	-0,15445798	-1,61532922	-0,10289105	0,0570521	NA	-0,00065333	0,49801214	-0,5357095	12	-20520,8527	41065,7227	3229,30594	0
3824	-0,45110821	0,66723944	0,00674107	4,29E-05	-0,00076432	NA	-0,15224001	-1,61532922	-0,10289105	0,0570521	NA	-0,00065333	0,49801214	-0,5357095	13	-20520,2945	41066,6092	3230,19249	0
3758	-0,14195779	0,65649184	NA	4,36E-05	-0,00074998	NA	-0,15445798	-1,76635397	NA	0,05800383	NA	-0,00051675	0,48731517	-0,53620329	11	-20523,8969	41069,8083	3233,39159	0
3760	-0,45110821	0,66723944	0,00674107	4,29E-05	-0,00076432	NA	-0,15224001	-1,76635397	NA	0,05800383	NA	-0,00051675	0,48731517	-0,53620329	12	-20523,3387	41070,6946	3234,27793	0
3790	-0,8455927	0,67951403	NA	4,64E-05	-0,00057321	NA	NA	-1,61532922	-0,10289105	0,0570521	NA	-0,00065333	0,49801214	-0,5357095	11	-20525,8732	41073,7609	3237,34423	0
3792	-1,17938783	0,69098467	0,00751019	4,56E-05	-0,00059207	NA	NA	-1,61532922	-0,10289105	0,0570521	NA	-0,00065333	0,49801214	-0,5357095	12	-20525,1832	41074,3837	3237,96698	0
3814	-0,79586264	0,77122371	NA	4,50E-05	NA	NA	-0,11503973	-1,61532922	-0,10289105	0,0570521	NA	-0,00065333	0,49801214	-0,5357095	11	-20526,4469	41074,9084	3238,49166	0
3816	-1,05409025	0,78157333	0,00543311	4,44E-05	NA	NA	-0,11273577	-1,61532922	-0,10289105	0,0570521	NA	-0,00065333	0,49801214	-0,5357095	12	-20526,0866	41076,1905	3239,77373	0
3726	-0,8455927	0,67951403	NA	4,64E-05	-0,00057321	NA	NA	-1,76635397	NA	0,05800383	NA	-0,00051675	0,48731517	-0,53620329	10	-20528,9173	41077,8468	3241,4301	0
3728	-1,17938783	0,69098467	0,00751019	4,56E-05	-0,00059207	NA	NA	-1,76635397	NA	0,05800383	NA	-0,00051675	0,48731517	-0,53620329	11	-20528,2274	41078,4694	3242,05264	0
3782	-1,235734	0,76857786	NA	4,69E-05	NA	NA	NA	-1,61532922	-0,10289105	0,0570521	NA	-0,00065333	0,49801214	-0,5357095	10	-20529,3455	41078,7032	3242,28651	0
3750	-0,79586264	0,77122371	NA	4,50E-05	NA	NA	-0,11503973	-1,76635397	NA	0,05800383	NA	-0,00051675	0,48731517	-0,53620329	10	-20529,491	41078,9943	3242,57753	0
3784	-1,52051301	0,78035551	0,00624724	4,63E-05	NA	NA	NA	-1,61532922	-0,10289105	0,0570521	NA	-0,00065333	0,49801214	-0,5357095	11	-20528,8694	41079,7535	3243,33676	0
3752	-1,05409025	0,78157333	0,00543311	4,44E-05	NA	NA	-0,11273577	-1,76635397	NA	0,05800383	NA	-0,00051675	0,48731517	-0,53620329	11	-20529,1308	41080,2761	3243,85939	0
3718	-1,235734	0,76857786	NA	4,69E-05	NA	NA	NA	-1,76635397	NA	0,05800383	NA	-0,00051675	0,48731517	-0,53620329	9	-20532,3897	41082,7893	3246,3726	0
3720	-1,52051301	0,78035551	0,00624724	4,63E-05	NA	NA	NA	-1,76635397	NA	0,05800383	NA	-0,00051675	0,48731517	-0,53620329	10	-20531,9136	41083,8394	3247,42263	0
3246	-0,14195779	0,65649184	NA	4,36E-05	-0,00074998	NA	-0,15445798	-2,06058113	NA	0,05699765	NA	NA	0,49693597	-0,52441365	10	-20533,1416	41086,2953	3249,87859	0
3837	0,65451489	NA	NA	3,95E-05	-0,00164357	0,48762971	-0,22170047	-1,61532922	-0,10289105	0,0570521	NA	-0,00065333	0,49801214	-0,5357095	12	-20531,1508	41086,3188	3249,90213	0
3248	-0,45110821	0,66723944	0,00674107	4,29E-05	-0,00076432	NA	-0,15224001	-2,06058113	NA	0,05699765	NA	NA	0,49693597	-0,52441365	11	-20532,5834	41087,1814	3250,76471	0
3310	-0,14195779	0,65649184	NA	4,36E-05	-0,00074998	NA	-0,15445798	-2,04753199	-0,01846226	0,05678211	NA	NA	0,49927467	-0,52377486	11	-20533,0239	41088,0623	3251,64561	0
3839	0,61699723	NA	0,00084798	3,94E-05	-0,00164748	0,4880593	-0,22142621	-1,61532922	-0,10289105	0,0570521	NA	-0,00065333	0,49801214	-0,5357095	13	-20531,1415	41088,3031	3251,88642	0
3312	-0,45110821	0,66723944	0,00674107	4,29E-05	-0,00076432	NA	-0,15224001	-2,04753199	-0,01846226	0,05678211	NA	NA	0,49927467	-0,52377486	12	-20532,4657	41088,9487	3252,53194	0
3773	0,65451489	NA	NA	3,95E-05	-0,00164357	0,48762971	-0,22170047	-1,76635397	NA	0,05800383	NA	-0,00051675	0,48731517	-0,53620329	11	-20534,1949	41090,4045	3253,98778	0
3775	0,61699723	NA	0,00084798	3,94E-05	-0,00164748	0,4880593	-0,22142621	-1,76635397	NA	0,05800383	NA	-0,00051675	0,48731517	-0,53620329	12	-20534,1857	41092,3886	3255,97185	0
3214	-0,8455927	0,67951403	NA	4,64E-05	-0,00057321	NA	NA	-2,06058113	NA	0,05699765	NA	NA	0,49693597	-0,52441365	9	-20538,162	41094,334	3257,91733	0
3216	-1,17938783	0,69098467	0,00751019	4,56E-05	-0,00059207	NA	NA	-2,06058113	NA	0,05699765	NA	NA	0,49693597	-0,52441365	10	-20537,4721	41094,9564	3258,53964	0
3238	-0,79586264	0,77122371	NA	4,50E-05	NA	NA	-0,11503973	-2,06058113	NA	0,05699765	NA	NA	0,49693597	-0,52441365	9	-20538,7358	41095,4815	3259,06476	0
3278	-0,8455927	0,67951403	NA	4,64E-05	-0,00057321	NA	NA	-2,04753199	-0,01846226	0,05678211	NA	NA	0,49927467	-0,52377486	10	-20538,0443	41096,1008	3259,68412	0
3280	-1,17938783	0,69098467	0,00751019	4,56E-05	-0,00059207	NA	NA	-2,04753199	-0,01846226	0,05678211	NA	NA	0,49927467	-0,52377486	11	-20537,3544	41096,7234	3260,30665	0
3240	-1,05409025	0,78157333	0,00543311	4,44E-05	NA	NA	-0,11273577	-2,06058113	NA	0,05699765	NA	NA	0,49693597	-0,52441365	10	-20538,3755	41096,7631	3260,34639	0
3302	-0,79586264	0,77122371	NA	4,50E-05	NA	NA	-0,11503973	-2,04753199	-0,01846226	0,05678211	NA	NA	0,49927467	-0,52377486	10	-20538,6181	41097,2483	3260,83155	0
3304	-1,05409025	0,78157333	0,00543311	4,44E-05	NA	NA	-0,11273577	-2,04753199	-0,01846226	0,05678211	NA	NA	0,49927467	-0,52377486	11	-20538,2578	41098,5301	3262,1134	0
3206	-1,235734	0,76857786	NA	4,69E-05	NA	NA	NA	-2,06058113	NA	0,05699765	NA	NA	0,49693597	-0,52441365	8	-20541,6344	41099,2768	3262,86005	0
3208	-1,52051301	0,78035551	0,00624724	4,63E-05	NA	NA	NA	-2,06058113	NA	0,05699765	NA	NA	0,49693597	-0,52441365	9	-20541,1583	41100,3266	3263,90986	0
3270	-1,235734	0,76857786	NA	4,69E-05	NA	NA	NA	-2,04753199	-0,01846226	0,05678211	NA	NA	0,49927467	-0,52377486	9	-20541,5167	41101,0433	3264,62662	0
3272	-1,52051301	0,78035551	0,00624724	4,63E-05	NA	NA	NA	-2,04753199	-0,01846226	0,05678211	NA	NA	0,49927467	-0,52377486	10	-20541,0406	41102,0934	3265,67665	0
3805	-0,31227401	NA	NA	4,32E-05	-0,00145314	0,47935513	NA	-1,61532922	-0,10289105	0,0570521	NA	-0,00065333	0,49801214	-0,5357095	11	-20541,0536	41104,1219	3267,70517	0
3807	-0,39160679	NA	0,00183718	4,30E-05	-0,00146197	0,48040455	NA	-1,61532922	-0,10289105	0,0570521	NA	-0,00065333	0,49801214	-0,5357095	12	-20541,0102	41106,0377	3269,621	0
3261	0,65451489	NA	NA	3,95E-05	-0,00164357	0,48762971	-0,22170047	-2,06058113	NA	0,05699765	NA	NA	0,49693597	-0,52441365	10	-20543,4397	41106,8915	3270,47478	0
3741	-0,31227401	NA	NA	4,32E-05	-0,00145314	0,47935513	NA	-1,76635397	NA	0,05800383	NA	-0,00051675	0,48731517	-0,53620329	10	-20544,0978	41108,2078	3271,79104	0
3325	0,65451489	NA	NA	3,95E-05	-0,00164357	0,48762971	-0,22170047	-2,04753199	-0,01846226	0,05678211	NA	NA	0,49927467	-0,52377486	11	-20543,322	41108,6585	3272,2418	0
3263	0,61699723	NA	0,00084798	3,94E-05	-0,00164748	0,4880593	-0,22142621	-2,06058113	NA	0,05699765	NA	NA	0,49693597	-0,52441365	11	-20543,4304	41108,8754	3272,45863	0
3743	-0,39160679	NA	0,00183718	4,30E-05	-0,00146197	0,48040455	NA	-1,76635397	NA	0,05800383	NA	-0,00051675	0,48731517	-0,53620329	11	-20544,0544	41110,1234	3273,70665	0
3327	0,61699723	NA	0,00084798	3,94E-05	-0,00164748	0,4880593	-0,22142621	-2,04753199	-0,01846226	0,05678211	NA	NA	0,49927467	-0,52377486	12	-20543,3127	41110,6426	3274,22586	0
3229	-0,31227401	NA	NA	4,32E-05	-0,00145314	0,47935513	NA	-2,06058113	NA	0,05699765	NA	NA	0,49693597	-0,52441365	9	-20553,3425	41124,695	3288,27826	0
3293	-0,31227401	NA	NA	4,32E-05	-0,00145314	0,47935513	NA	-2,04753199	-0,01846226	0,05678211	NA	NA	0,49927467	-0,52377486	10	-20553,2248	41126,4618	3290,04506	0
3231	-0,39160679	NA	0,00183718	4,30E-05	-0,00146197	0,48040455	NA	-2,06058113	NA	0,05699765	NA	NA	0,49693597	-0,52441365	10	-20553,2991	41126,6104	3290,19366	0
3295	-0,39160679	NA	0,00183718	4,30E-05	-0,00146197	0,48040455	NA	-2,04753199	-0,01846226	0,05678211	NA	NA	0,49927467	-0,52377486	11	-20553,1814	41128,3774	3291,96067	0
3821	0,64576877	NA	NA	3,88E-05	-0,00183092	NA	-0,20438761	-1,61532922	-0,10289105	0,0570521	NA	-0,00065333	0,49801214	-0,5357095	11	-20566,7556	41155,5258	3319,10911	0
3829	-0,84339357	NA	NA	4,07E-05	NA	0,55505772	-0,13640315	-1,61532922	-0,10289105	0,0570521	NA	-0,00065333	0,49801214	-0,5357095	11	-20567,0138	41156,0423	3319,62555	0

3831	-0,50593593	NA	-0,00700816	4,14E-05	NA	0,55021643	-0,14061875	-1,61532922	-0,10289105	0,0570521	NA	-0,00065333	0,49801214	-0,5357095	12	-20566,3808	41156,7788	3320,36208	0
3823	0,74969745	NA	-0,00245756	3,91E-05	-0,00181949	NA	-0,20513804	-1,61532922	-0,10289105	0,0570521	NA	-0,00065333	0,49801214	-0,5357095	12	-20566,68	41157,3773	3320,96062	0
3757	0,64576877	NA	NA	3,88E-05	-0,00183092	NA	-0,20438761	-1,76635397	NA	0,05800383	NA	-0,00051675	0,48731517	-0,53620329	10	-20569,7998	41159,6117	3323,19499	0
3765	-0,84339357	NA	NA	4,07E-05	NA	0,55505772	-0,13640315	-1,76635397	NA	0,05800383	NA	-0,00051675	0,48731517	-0,53620329	10	-20570,058	41160,1281	3323,71142	0
3767	-0,50593593	NA	-0,00700816	4,14E-05	NA	0,55021643	-0,14061875	-1,76635397	NA	0,05800383	NA	-0,00051675	0,48731517	-0,53620329	11	-20569,4249	41160,8645	3324,44773	0
3797	-1,36352414	NA	NA	4,29E-05	NA	0,54467715	NA	-1,61532922	-0,10289105	0,0570521	NA	-0,00065333	0,49801214	-0,5357095	10	-20570,5542	41161,1206	3324,70387	0
3759	0,74969745	NA	-0,00245756	3,91E-05	-0,00181949	NA	-0,20513804	-1,76635397	NA	0,05800383	NA	-0,00051675	0,48731517	-0,53620329	11	-20569,7242	41161,463	3325,04627	0
3799	-1,08960867	NA	-0,00576757	4,36E-05	NA	0,53973144	NA	-1,61532922	-0,10289105	0,0570521	NA	-0,00065333	0,49801214	-0,5357095	11	-20570,1219	41162,2584	3325,84173	0
3733	-1,36352414	NA	NA	4,29E-05	NA	0,54467715	NA	-1,76635397	NA	0,05800383	NA	-0,00051675	0,48731517	-0,53620329	9	-20573,5984	41165,2067	3328,78997	0
3735	-1,08960867	NA	-0,00576757	4,36E-05	NA	0,53973144	NA	-1,76635397	NA	0,05800383	NA	-0,00051675	0,48731517	-0,53620329	10	-20573,1661	41166,3443	3329,9276	0
3789	-0,28011525	NA	NA	4,24E-05	-0,0016491	NA	NA	-1,61532922	-0,10289105	0,0570521	NA	-0,00065333	0,49801214	-0,5357095	10	-20575,0459	41170,104	3333,68729	0
3791	-0,2131598	NA	-0,0015413	4,25E-05	-0,00164139	NA	NA	-1,61532922	-0,10289105	0,0570521	NA	-0,00065333	0,49801214	-0,5357095	11	-20575,0162	41172,0471	3335,63034	0
3725	-0,28011525	NA	NA	4,24E-05	-0,0016491	NA	NA	-1,76635397	NA	0,05800383	NA	-0,00051675	0,48731517	-0,53620329	9	-20578,0901	41174,1901	3337,77339	0
3245	0,64576877	NA	NA	3,88E-05	-0,00183092	NA	-0,20438761	-2,06058113	NA	0,05699765	NA	NA	0,49693597	-0,52441365	9	-20579,0445	41176,0989	3339,68221	0
3727	-0,2131598	NA	-0,0015413	4,25E-05	-0,00164139	NA	NA	-1,76635397	NA	0,05800383	NA	-0,00051675	0,48731517	-0,53620329	10	-20578,0604	41176,1329	3339,71621	0
3253	-0,84339357	NA	NA	4,07E-05	NA	0,55505772	-0,13640315	-2,06058113	NA	0,05699765	NA	NA	0,49693597	-0,52441365	9	-20579,3027	41176,6154	3340,19865	0
3255	-0,50593593	NA	-0,00700816	4,14E-05	NA	0,55021643	-0,14061875	-2,06058113	NA	0,05699765	NA	NA	0,49693597	-0,52441365	10	-20578,6696	41177,3515	3340,93474	0
3309	0,64576877	NA	NA	3,88E-05	-0,00183092	NA	-0,20438761	-2,04753199	-0,01846226	0,05678211	NA	NA	0,49927467	-0,52377486	10	-20578,9268	41177,8657	3341,449	0
3247	0,74969745	NA	-0,00245756	3,91E-05	-0,00181949	NA	-0,20513804	-2,06058113	NA	0,05699765	NA	NA	0,49693597	-0,52441365	10	-20578,9689	41177,95	3341,53328	0
3317	-0,84339357	NA	NA	4,07E-05	NA	0,55505772	-0,13640315	-2,04753199	-0,01846226	0,05678211	NA	NA	0,49927467	-0,52377486	10	-20579,185	41178,3822	3341,96544	0
3319	-0,50593593	NA	-0,00700816	4,14E-05	NA	0,55021643	-0,14061875	-2,04753199	-0,01846226	0,05678211	NA	NA	0,49927467	-0,52377486	11	-20578,5519	41179,1185	3342,70175	0
2750	-0,06671637	0,57575795	NA	4,34E-05	-0,00071955	0,41051829	-0,17478186	-1,63848397	NA	0,05580518	NA	-0,00060814	NA	-0,48687105	11	-20578,6623	41179,3392	3342,92243	0
2752	-0,47166397	0,58945108	0,00876766	4,25E-05	-0,00073797	0,4135805	-0,1717464	-1,63848397	NA	0,05580518	NA	-0,00060814	NA	-0,48687105	12	-20577,6881	41179,3934	3342,97671	0
3311	0,74969745	NA	-0,00245756	3,91E-05	-0,00181949	NA	-0,20513804	-2,04753199	-0,01846226	0,05678211	NA	NA	0,49927467	-0,52377486	11	-20578,8512	41179,717	3343,30029	0
2814	-0,06671637	0,57575795	NA	4,34E-05	-0,00071955	0,41051829	-0,17478186	-1,57337207	-0,04401868	0,05538852	NA	-0,0006675	NA	-0,48613452	12	-20578,0912	41180,1996	3343,78292	0
2816	-0,47166397	0,58945108	0,00876766	4,25E-05	-0,00073797	0,4135805	-0,1717464	-1,57337207	-0,04401868	0,05538852	NA	-0,0006675	NA	-0,48613452	13	-20577,117	41180,2541	3343,83742	0
3221	-1,36352414	NA	NA	4,29E-05	NA	0,54467715	NA	-2,06058113	NA	0,05699765	NA	NA	0,49693597	-0,52441365	8	-20582,8431	41181,6941	3345,27741	0
3223	-1,08960867	NA	-0,00576757	4,36E-05	NA	0,53973144	NA	-2,06058113	NA	0,05699765	NA	NA	0,49693597	-0,52441365	9	-20582,4108	41182,8315	3346,41483	0
3285	-1,36352414	NA	NA	4,29E-05	NA	0,54467715	NA	-2,04753199	-0,01846226	0,05678211	NA	NA	0,49927467	-0,52377486	9	-20582,7254	41183,4607	3347,04398	0
3287	-1,08960867	NA	-0,00576757	4,36E-05	NA	0,53973144	NA	-2,04753199	-0,01846226	0,05678211	NA	NA	0,49927467	-0,52377486	10	-20582,2931	41184,5983	3348,18162	0
2742	-0,67953035	0,68234123	NA	4,46E-05	NA	0,41541375	-0,13795142	-1,63848397	NA	0,05580518	NA	-0,00060814	NA	-0,48687105	10	-20583,8489	41187,7099	3351,29322	0
2744	-1,04079715	0,69637088	0,00755047	4,39E-05	NA	0,41816409	-0,13461471	-1,63848397	NA	0,05580518	NA	-0,00060814	NA	-0,48687105	11	-20583,1296	41188,2739	3351,85717	0
2806	-0,67953035	0,68234123	NA	4,46E-05	NA	0,41541375	-0,13795142	-1,57337207	-0,04401868	0,05538852	NA	-0,0006675	NA	-0,48613452	11	-20583,2778	41188,5702	3352,15349	0
2808	-1,04079715	0,69637088	0,00755047	4,39E-05	NA	0,41816409	-0,13461471	-1,57337207	-0,04401868	0,05538852	NA	-0,0006675	NA	-0,48613452	12	-20582,5586	41189,1344	3352,71766	0
2720	-1,28042467	0,61751101	0,00965662	4,55E-05	-0,00054807	0,40424516	NA	-1,63848397	NA	0,05580518	NA	-0,00060814	NA	-0,48687105	11	-20583,9343	41189,8832	3353,4665	0
2718	-0,85074914	0,60334436	NA	4,65E-05	-0,00052401	0,40049867	NA	-1,63848397	NA	0,05580518	NA	-0,00060814	NA	-0,48687105	10	-20585,1101	41190,2325	3353,81573	0
3213	-0,28011525	NA	NA	4,24E-05	-0,0016491	NA	NA	-2,06058113	NA	0,05699765	NA	NA	0,49693597	-0,52441365	8	-20587,3348	41190,6776	3354,26083	0
2784	-1,28042467	0,61751101	0,00965662	4,55E-05	-0,00054807	0,40424516	NA	-1,57337207	-0,04401868	0,05538852	NA	-0,0006675	NA	-0,48613452	12	-20583,3632	41190,7437	3354,32699	0
2782	-0,85074914	0,60334436	NA	4,65E-05	-0,00052401	0,40049867	NA	-1,57337207	-0,04401868	0,05538852	NA	-0,0006675	NA	-0,48613452	11	-20584,5391	41191,0927	3354,676	0
3277	-0,28011525	NA	NA	4,24E-05	-0,0016491	NA	NA	-2,04753199	-0,01846226	0,05678211	NA	NA	0,49927467	-0,52377486	9	-20587,2171	41192,4441	3356,0274	0
3215	-0,2131598	NA	-0,0015413	4,25E-05	-0,00164139	NA	NA	-2,06058113	NA	0,05699765	NA	NA	0,49693597	-0,52441365	9	-20587,3051	41192,6202	3356,20344	0
2710	-1,1987332	0,68175498	NA	4,69E-05	NA	0,40581671	NA	-1,63848397	NA	0,05580518	NA	-0,00060814	NA	-0,48687105	9	-20588,024	41194,058	3357,64128	0
2712	-1,59179834	0,69737855	0,00852736	4,60E-05	NA	0,40932129	NA	-1,63848397	NA	0,05580518	NA	-0,00060814	NA	-0,48687105	10	-20587,1076	41194,2274	3357,81065	0
3279	-0,2131598	NA	-0,0015413	4,25E-05	-0,00164139	NA	NA	-2,04753199	-0,01846226	0,05678211	NA	NA	0,49927467	-0,52377486	10	-20587,1874	41194,3869	3357,97023	0
2774	-1,1987332	0,68175498	NA	4,69E-05	NA	0,40581671	NA	-1,57337207	-0,04401868	0,05538852	NA	-0,0006675	NA	-0,48613452	10	-20587,4529	41194,918	3358,50132	0
2776	-1,59179834	0,69737855	0,00852736	4,60E-05	NA	0,40932129	NA	-1,57337207	-0,04401868	0,05538852	NA	-0,0006675	NA	-0,48613452	11	-20586,5365	41195,0876	3358,67092	0
2238	-0,06671637	0,57575795	NA	4,34E-05	-0,00071955	0,41051829	-0,17478186	-1,98285198	NA	0,054624	NA	NA	NA	-0,47270606	10	-20591,4443	41202,9007	3366,484	0
2240	-0,47166397	0,58945108	0,00876766	4,25E-05	-0,00073797	0,4135805	-0,1717464	-1,98285198	NA	0,054624	NA	NA	NA	-0,47270606	11	-20590,4701	41202,9548	3366,53805	0
2302	-0,06671637	0,57575795	NA	4,34E-05	-0,00071955	0,41051829	-0,17478186	-2,01223022	0,04141198	0,05511629	NA	NA	NA	-0,47466077	11	-20590,8377	41203,69	3367,27325	0
2304	-0,47166397	0,58945108	0,00876766	4,25E-05	-0,00073797	0,4135805	-0,1717464	-2,01223022	0,04141198	0,05511629	NA	NA	NA	-0,47466077	12	-20589,8635	41203,7443	3367,32753	0
2230	-0,67953035	0,68234123	NA	4,46E-05	NA	0,41541375	-0,13795142	-1,98285198	NA	0,054624	NA	NA	NA	-0,47270606	9	-20596,6309	41211,2717	3374,85501	0
2232	-1,04079715	0,69637088	0,00755047	4,39E-05	NA	0,41816409	-0,13461471	-1,98285198	NA	0,054624	NA	NA	NA	-0,47270606	10	-20595,9116	41211,8355	3375,41873	0
2294	-0,67953035	0,68234123	NA	4,46E-05	NA	0,41541375	-0,13795142	-2,01223022	0,04141198	0,05511629	NA	NA	NA	-0,47466077	10	-20596,0243	41212,0608	3375,64404	0
2296	-1,04079715	0,69637088	0,00755047	4,39E-05	NA	0,41816409	-0,13461471	-2,01223022	0,04141198	0,05511629	NA	NA	NA	-0,47466077	11	-20595,3051	41212,6247	3376,20799	0
2208	-1,28042467	0,61751101	0,00965662	4,55E-05	-0,00054807	0,40424516	NA	-1,98285198	NA	0,054624	NA	NA	NA	-0,47270606	10	-20596,7163	41213,4448	3377,02806	0
2206	-0,85074914	0,60334436	NA	4,65E-05	-0,00052401	0,40049867	NA	-1,98285198	NA	0,054624	NA	NA	NA	-0,47270606	9	-20597,8921	41213,7942	3377,37752	0
2272	-1,28042467	0,61751101	0,00965662	4,55E-05	-0,00054807	0,40424516	NA	-2,01223022	0,04141198	0,05511629	NA	NA	NA	-0,47466077	11	-20596,1097	41214,234	3377,81732	0
2270	-0,85074914	0,60334436	NA	4,65E-05	-0,00052401	0,40049867	NA	-2,01223022	0,04141198	0,05511629	NA	NA	NA	-0,47466077	10	-20597,2856	41214,5833	3378,16655	0
2198	-1,1987332	0,68175498	NA	4,69E-05	NA	0,40581671	NA	-1,98285198	NA	0,054624	NA	NA	NA	-0,47270606	8	-20600,806	41217,62	3381,20328	0
2200	-1,59179834	0,69737855	0,00852736	4,60E-05	NA	0,40932129	NA	-1,98285198	NA	0,054624	NA	NA	NA	-0,47270606	9	-20599,8896	41217,7892	3381,37243	0
2262	-1,1987332	0,68175498	NA	4,69E-05	NA	0,40581671	NA	-2,01223022	0,04141198	0,05511629	NA	NA	NA	-0,47466077	9	-20600,1994	41218,4088	3381,9921	0

2264	-1,59179834	0,69737855	0,00852736	4,60E-05	NA	0,40932129	NA	-2,01223022	0,04141198	0,05511629	NA	NA	NA	-0,47466077	10	-20599,283	41218,5782	3382,16147	0
2734	-0,14195779	0,65649184	NA	4,36E-05	-0,00074998	NA	-0,15445798	-1,63848397	NA	0,05580518	NA	-0,00060814	NA	-0,48687105	10	-20604,1914	41228,3951	3391,97833	0
2798	-0,14195779	0,65649184	NA	4,36E-05	-0,00074998	NA	-0,15445798	-1,57337207	-0,04401868	0,05538852	NA	-0,0006675	NA	-0,48613452	11	-20603,6204	41229,2553	3392,8386	0
2736	-0,45110821	0,66723944	0,00674107	4,29E-05	-0,00076432	NA	-0,15224001	-1,63848397	NA	0,05580518	NA	-0,00060814	NA	-0,48687105	11	-20603,6333	41229,2812	3392,86445	0
2800	-0,45110821	0,66723944	0,00674107	4,29E-05	-0,00076432	NA	-0,15224001	-1,57337207	-0,04401868	0,05538852	NA	-0,0006675	NA	-0,48613452	12	-20603,0622	41230,1417	3393,72494	0
2702	-0,8455927	0,67951403	NA	4,64E-05	-0,00057321	NA	NA	-1,63848397	NA	0,05580518	NA	-0,00060814	NA	-0,48687105	9	-20609,2119	41236,4338	3400,01707	0
2704	-1,17938783	0,69098467	0,00751019	4,56E-05	-0,00059207	NA	NA	-1,63848397	NA	0,05580518	NA	-0,00060814	NA	-0,48687105	10	-20608,522	41237,0561	3400,63938	0
2766	-0,8455927	0,67951403	NA	4,64E-05	-0,00057321	NA	NA	-1,57337207	-0,04401868	0,05538852	NA	-0,0006675	NA	-0,48613452	10	-20608,6408	41237,2938	3400,87711	0
2726	-0,79586264	0,77122371	NA	4,50E-05	NA	NA	-0,11503973	-1,63848397	NA	0,05580518	NA	-0,00060814	NA	-0,48687105	9	-20609,7856	41237,5812	3401,1645	0
2768	-1,17938783	0,69098467	0,00751019	4,56E-05	-0,00059207	NA	NA	-1,57337207	-0,04401868	0,05538852	NA	-0,0006675	NA	-0,48613452	11	-20607,9509	41237,9164	3401,49965	0
2790	-0,79586264	0,77122371	NA	4,50E-05	NA	NA	-0,11503973	-1,57337207	-0,04401868	0,05538852	NA	-0,0006675	NA	-0,48613452	10	-20609,2145	41238,4413	3402,02454	0
2728	-1,05409025	0,78157333	0,00543311	4,44E-05	NA	NA	-0,11273577	-1,63848397	NA	0,05580518	NA	-0,00060814	NA	-0,48687105	10	-20609,4253	41238,8629	3402,44613	0
2792	-1,05409025	0,78157333	0,00543311	4,44E-05	NA	NA	-0,11273577	-1,57337207	-0,04401868	0,05538852	NA	-0,0006675	NA	-0,48613452	11	-20608,8543	41239,7231	3403,3064	0
2694	-1,235734	0,76857786	NA	4,69E-05	NA	NA	NA	-1,63848397	NA	0,05580518	NA	-0,00060814	NA	-0,48687105	8	-20612,6843	41241,3765	3404,95979	0
2758	-1,235734	0,76857786	NA	4,69E-05	NA	NA	NA	-1,57337207	-0,04401868	0,05538852	NA	-0,0006675	NA	-0,48613452	9	-20612,1132	41242,2363	3405,81961	0
2696	-1,52051301	0,78035551	0,00624724	4,63E-05	NA	NA	NA	-1,63848397	NA	0,05580518	NA	-0,00060814	NA	-0,48687105	9	-20612,2082	41242,4263	3406,0096	0
3815	-0,65771633	NA	-0,01187559	4,14E-05	NA	NA	-0,10732692	-1,61532922	-0,10289105	0,0570521	NA	-0,00065333	0,49801214	-0,5357095	11	-20610,2963	41242,6072	3406,19048	0
2760	-1,52051301	0,78035551	0,00624724	4,63E-05	NA	NA	NA	-1,57337207	-0,04401868	0,05538852	NA	-0,0006675	NA	-0,48613452	10	-20611,6371	41243,2864	3406,86964	0
3813	-1,22822825	NA	NA	4,02E-05	NA	NA	-0,09995451	-1,61532922	-0,10289105	0,0570521	NA	-0,00065333	0,49801214	-0,5357095	10	-20612,0407	41244,0937	3407,67694	0
3783	-1,14142692	NA	-0,01089562	4,31E-05	NA	NA	NA	-1,61532922	-0,10289105	0,0570521	NA	-0,00065333	0,49801214	-0,5357095	10	-20612,4104	41244,833	3408,41624	0
3781	-1,63084607	NA	NA	4,18E-05	NA	NA	NA	-1,61532922	-0,10289105	0,0570521	NA	-0,00065333	0,49801214	-0,5357095	9	-20613,8856	41245,7811	3409,36439	0
3751	-0,65771633	NA	-0,01187559	4,14E-05	NA	NA	-0,10732692	-1,76635397	NA	0,05800383	NA	-0,00051675	0,48731517	-0,53620329	10	-20613,3405	41246,6931	3410,27635	0
3749	-1,22822825	NA	NA	4,02E-05	NA	NA	-0,09995451	-1,76635397	NA	0,05800383	NA	-0,00051675	0,48731517	-0,53620329	9	-20615,0849	41248,1798	3411,76303	0
3719	-1,14142692	NA	-0,01089562	4,31E-05	NA	NA	NA	-1,76635397	NA	0,05800383	NA	-0,00051675	0,48731517	-0,53620329	9	-20615,4546	41248,9191	3412,50234	0
2749	0,65451489	NA	NA	3,95E-05	-0,00164357	0,48762971	-0,22170047	-1,63848397	NA	0,05580518	NA	-0,00060814	NA	-0,48687105	10	-20614,4895	41248,9912	3412,57452	0
2813	0,65451489	NA	NA	3,95E-05	-0,00164357	0,48762971	-0,22170047	-1,57337207	-0,04401868	0,05538852	NA	-0,0006675	NA	-0,48613452	11	-20613,9185	41249,8515	3413,43479	0
3717	-1,63084607	NA	NA	4,18E-05	NA	NA	NA	-1,76635397	NA	0,05800383	NA	-0,00051675	0,48731517	-0,53620329	8	-20616,9297	41249,8674	3413,45071	0
2751	0,61699723	NA	0,00084798	3,94E-05	-0,00164748	0,4880593	-0,22142621	-1,63848397	NA	0,05580518	NA	-0,00060814	NA	-0,48687105	11	-20614,4802	41250,9751	3414,55837	0
2815	0,61699723	NA	0,00084798	3,94E-05	-0,00164748	0,4880593	-0,22142621	-1,57337207	-0,04401868	0,05538852	NA	-0,0006675	NA	-0,48613452	12	-20613,9092	41251,8356	3415,41886	0
2222	-0,14195779	0,65649184	NA	4,36E-05	-0,00074998	NA	-0,15445798	-1,98285198	NA	0,054624	NA	NA	NA	-0,47270606	9	-20616,9734	41251,9568	3415,54012	0
2286	-0,14195779	0,65649184	NA	4,36E-05	-0,00074998	NA	-0,15445798	-2,01223022	0,04141198	0,05511629	NA	NA	NA	-0,47466077	10	-20616,3669	41252,7459	3416,32915	0
2224	-0,45110821	0,66723944	0,00674107	4,29E-05	-0,00076432	NA	-0,15224001	-1,98285198	NA	0,054624	NA	NA	NA	-0,47270606	10	-20616,4153	41252,8427	3416,42601	0
2288	-0,45110821	0,66723944	0,00674107	4,29E-05	-0,00076432	NA	-0,15224001	-2,01223022	0,04141198	0,05511629	NA	NA	NA	-0,47466077	11	-20615,8087	41253,632	3417,21527	0
2190	-0,8455927	0,67951403	NA	4,64E-05	-0,00057321	NA	NA	-1,98285198	NA	0,054624	NA	NA	NA	-0,47270606	8	-20621,9939	41259,9958	3423,57907	0
2192	-1,17938783	0,69098467	0,00751019	4,56E-05	-0,00059207	NA	NA	-1,98285198	NA	0,054624	NA	NA	NA	-0,47270606	9	-20621,304	41260,6179	3424,20117	0
2254	-0,8455927	0,67951403	NA	4,64E-05	-0,00057321	NA	NA	-2,01223022	0,04141198	0,05511629	NA	NA	NA	-0,47466077	9	-20621,3873	41260,7846	3424,36789	0
2214	-0,79586264	0,77122371	NA	4,50E-05	NA	NA	-0,11503973	-1,98285198	NA	0,054624	NA	NA	NA	-0,47270606	8	-20622,5676	41261,1432	3424,7265	0
2256	-1,17938783	0,69098467	0,00751019	4,56E-05	-0,00059207	NA	NA	-2,01223022	0,04141198	0,05511629	NA	NA	NA	-0,47466077	10	-20620,6974	41261,4069	3424,9902	0
2278	-0,79586264	0,77122371	NA	4,50E-05	NA	NA	-0,11503973	-2,01223022	0,04141198	0,05511629	NA	NA	NA	-0,47466077	9	-20621,961	41261,932	3425,51531	0
2216	-1,05409025	0,78157333	0,00543311	4,44E-05	NA	NA	-0,11273577	-1,98285198	NA	0,054624	NA	NA	NA	-0,47270606	9	-20622,2073	41262,4246	3426,00792	0
3239	-0,65771633	NA	-0,01187559	4,14E-05	NA	NA	-0,10732692	-2,06058113	NA	0,05699765	NA	NA	0,49693597	-0,52441365	9	-20622,5852	41263,1803	3426,76357	0
2280	-1,05409025	0,78157333	0,00543311	4,44E-05	NA	NA	-0,11273577	-2,01223022	0,04141198	0,05511629	NA	NA	NA	-0,47466077	10	-20621,6008	41263,2137	3426,79695	0
3237	-1,22822825	NA	NA	4,02E-05	NA	NA	-0,09995451	-2,06058113	NA	0,05699765	NA	NA	0,49693597	-0,52441365	8	-20624,3296	41264,6672	3428,25048	0
2182	-1,235734	0,76857786	NA	4,69E-05	NA	NA	NA	-1,98285198	NA	0,054624	NA	NA	NA	-0,47270606	7	-20625,4663	41264,9387	3428,52202	0
3303	-0,65771633	NA	-0,01187559	4,14E-05	NA	NA	-0,10732692	-2,04753199	-0,01846226	0,05678211	NA	NA	0,49927467	-0,52377486	10	-20622,4675	41264,9471	3428,53037	0
3207	-1,14142692	NA	-0,01089562	4,31E-05	NA	NA	NA	-2,06058113	NA	0,05699765	NA	NA	0,49693597	-0,52441365	8	-20624,6993	41265,4065	3428,98979	0
2246	-1,235734	0,76857786	NA	4,69E-05	NA	NA	NA	-2,01223022	0,04141198	0,05511629	NA	NA	NA	-0,47466077	8	-20624,8597	41265,7273	3429,31061	0
2184	-1,52051301	0,78035551	0,00624724	4,63E-05	NA	NA	NA	-1,98285198	NA	0,054624	NA	NA	NA	-0,47270606	8	-20624,9902	41265,9883	3429,57161	0
3205	-1,63084607	NA	NA	4,18E-05	NA	NA	NA	-2,06058113	NA	0,05699765	NA	NA	0,49693597	-0,52441365	7	-20626,1745	41266,3551	3429,93838	0
3301	-1,22822825	NA	NA	4,02E-05	NA	NA	-0,09995451	-2,04753199	-0,01846226	0,05678211	NA	NA	0,49927467	-0,52377486	9	-20624,2119	41266,4338	3430,01705	0
2248	-1,52051301	0,78035551	0,00624724	4,63E-05	NA	NA	NA	-2,01223022	0,04141198	0,05511629	NA	NA	NA	-0,47466077	9	-20624,3836	41266,7771	3430,36042	0
2717	-0,31227401	NA	NA	4,32E-05	-0,00145314	0,47935513	NA	-1,63848397	NA	0,05580518	NA	-0,00060814	NA	-0,48687105	9	-20624,3924	41266,7947	3430,378	0
3271	-1,14142692	NA	-0,01089562	4,31E-05	NA	NA	NA	-2,04753199	-0,01846226	0,05678211	NA	NA	0,49927467	-0,52377486	9	-20624,5816	41267,1731	3430,75636	0
2781	-0,31227401	NA	NA	4,32E-05	-0,00145314	0,47935513	NA	-1,57337207	-0,04401868	0,05538852	NA	-0,0006675	NA	-0,48613452	10	-20623,8213	41267,6548	3431,23805	0
3269	-1,63084607	NA	NA	4,18E-05	NA	NA	NA	-2,04753199	-0,01846226	0,05678211	NA	NA	0,49927467	-0,52377486	8	-20626,0567	41268,1214	3431,70473	0
2719	-0,39160679	NA	0,00183718	4,30E-05	-0,00146197	0,48040455	NA	-1,63848397	NA	0,05580518	NA	-0,00060814	NA	-0,48687105	10	-20624,349	41268,7101	3432,29339	0
2783	-0,39160679	NA	0,00183718	4,30E-05	-0,00146197	0,48040455	NA	-1,57337207	-0,04401868	0,05538852	NA	-0,0006675	NA	-0,48613452	11	-20623,7779	41269,5704	3433,15366	0
2237	0,65451489	NA	NA	3,95E-05	-0,00164357	0,48762971	-0,22170047	-1,98285198	NA	0,054624	NA	NA	NA	-0,47270606	9	-20627,2715	41272,553	3436,13631	0
2301	0,65451489	NA	NA	3,95E-05	-0,00164357	0,48762971	-0,22170047	-2,01223022	0,04141198	0,05511629	NA	NA	NA	-0,47466077	10	-20626,6649	41273,3421	3436,92534	0
2239	0,61699723	NA	0,00084798	3,94E-05	-0,00164748	0,4880593	-0,22142621	-1,98285198	NA	0,054624	NA	NA	NA	-0,47270606	10	-20627,2622	41274,5367	3438,11993	0
2303	0,61699723	NA	0,00084798	3,94E-05	-0,00164748	0,4880593	-0,22142621	-2,01223022	0,04141198	0,05511629	NA	NA	NA	-0,47466077	11	-20626,6557	41275,3259	3438,90919	0

2205	-0,31227401	NA	NA	4,32E-05	-0,00145314	0,47935513	NA	-1,98285198	NA	0,054624	NA	NA	NA	-0,47270606	8	-20637,1744	41290,3567	3453,94001	0
2269	-0,31227401	NA	NA	4,32E-05	-0,00145314	0,47935513	NA	-2,01223022	0,04141198	0,05511629	NA	NA	NA	-0,47466077	9	-20636,5678	41291,1455	3454,72882	0
2207	-0,39160679	NA	0,00183718	4,30E-05	-0,00146197	0,48040455	NA	-1,98285198	NA	0,054624	NA	NA	NA	-0,47270606	9	-20637,131	41292,2719	3455,85518	0
2271	-0,39160679	NA	0,00183718	4,30E-05	-0,00146197	0,48040455	NA	-2,01223022	0,04141198	0,05511629	NA	NA	NA	-0,47466077	10	-20636,5244	41293,0609	3456,64421	0
2733	0,64576877	NA	NA	3,88E-05	-0,00183092	NA	-0,20438761	-1,63848397	NA	0,05580518	NA	-0,00060814	NA	-0,48687105	9	-20650,0944	41318,1987	3481,78195	0
2741	-0,84339357	NA	NA	4,07E-05	NA	0,55505772	-0,13640315	-1,63848397	NA	0,05580518	NA	-0,00060814	NA	-0,48687105	9	-20650,3526	41318,7151	3482,29838	0
2797	0,64576877	NA	NA	3,88E-05	-0,00183092	NA	-0,20438761	-1,57337207	-0,04401868	0,05538852	NA	-0,0006675	NA	-0,48613452	10	-20649,5233	41319,0587	3482,642	0
2743	-0,50593593	NA	-0,00700816	4,14E-05	NA	0,55021643	-0,14061875	-1,63848397	NA	0,05580518	NA	-0,00060814	NA	-0,48687105	10	-20649,7195	41319,4512	3483,03448	0
2805	-0,84339357	NA	NA	4,07E-05	NA	0,55505772	-0,13640315	-1,57337207	-0,04401868	0,05538852	NA	-0,0006675	NA	-0,48613452	10	-20649,7815	41319,5752	3483,15843	0
2735	0,74969745	NA	-0,00245756	3,91E-05	-0,00181949	NA	-0,20513804	-1,63848397	NA	0,05580518	NA	-0,00060814	NA	-0,48687105	10	-20650,0188	41320,0497	3483,63301	0
2807	-0,50593593	NA	-0,00700816	4,14E-05	NA	0,55021643	-0,14061875	-1,57337207	-0,04401868	0,05538852	NA	-0,0006675	NA	-0,48613452	11	-20649,1484	41320,3115	3483,89474	0
2799	0,74969745	NA	-0,00245756	3,91E-05	-0,00181949	NA	-0,20513804	-1,57337207	-0,04401868	0,05538852	NA	-0,0006675	NA	-0,48613452	11	-20649,4477	41320,91	3484,49328	0
3710	-0,06671637	0,57575795	NA	4,34E-05	-0,00071955	0,41051829	-0,17478186	1,15818376	-0,19528017	NA	NA	-0,00061584	0,46809624	-0,5501339	12	-20649,4482	41322,9136	3486,49686	0
3712	-0,47166397	0,58945108	0,00876766	4,25E-05	-0,00073797	0,4135805	-0,1717464	1,15818376	-0,19528017	NA	NA	-0,00061584	0,46809624	-0,5501339	13	-20648,474	41322,9681	3486,55136	0
2709	-1,36352414	NA	NA	4,29E-05	NA	0,54467715	NA	-1,63848397	NA	0,05580518	NA	-0,00060814	NA	-0,48687105	8	-20653,893	41323,7939	3487,37715	0
2773	-1,36352414	NA	NA	4,29E-05	NA	0,54467715	NA	-1,57337207	-0,04401868	0,05538852	NA	-0,0006675	NA	-0,48613452	9	-20653,3219	41324,6537	3488,23698	0
2711	-1,08960867	NA	-0,00576757	4,36E-05	NA	0,53973144	NA	-1,63848397	NA	0,05580518	NA	-0,00060814	NA	-0,48687105	9	-20653,4607	41324,9313	3488,51457	0
2775	-1,08960867	NA	-0,00576757	4,36E-05	NA	0,53973144	NA	-1,57337207	-0,04401868	0,05538852	NA	-0,0006675	NA	-0,48613452	10	-20652,8896	41325,7913	3489,37461	0
3702	-0,67953035	0,68234123	NA	4,46E-05	NA	0,41541375	-0,13795142	1,15818376	-0,19528017	NA	NA	-0,00061584	0,46809624	-0,5501339	11	-20654,6348	41331,2842	3494,86743	0
3704	-1,04079715	0,69637088	0,00755047	4,39E-05	NA	0,41816409	-0,13461471	1,15818376	-0,19528017	NA	NA	-0,00061584	0,46809624	-0,5501339	12	-20653,9155	41331,8483	3495,4316	0
2701	-0,28011525	NA	NA	4,24E-05	-0,0016491	NA	NA	-1,63848397	NA	0,05580518	NA	-0,00060814	NA	-0,48687105	8	-20658,3847	41332,7773	3496,36057	0
3680	-1,28042467	0,61751101	0,00965662	4,55E-05	-0,00054807	0,40424516	NA	1,15818376	-0,19528017	NA	NA	-0,00061584	0,46809624	-0,5501339	12	-20654,7202	41333,4576	3497,04093	0
2765	-0,28011525	NA	NA	4,24E-05	-0,0016491	NA	NA	-1,57337207	-0,04401868	0,05538852	NA	-0,0006675	NA	-0,48613452	9	-20657,8136	41333,6371	3497,2204	0
3678	-0,85074914	0,60334436	NA	4,65E-05	-0,00052401	0,40049867	NA	1,15818376	-0,19528017	NA	NA	-0,00061584	0,46809624	-0,5501339	11	-20655,896	41333,8067	3497,38994	0
2703	-0,2131598	NA	-0,0015413	4,25E-05	-0,00164139	NA	NA	-1,63848397	NA	0,05580518	NA	-0,00060814	NA	-0,48687105	9	-20658,355	41334,7199	3498,30318	0
2767	-0,2131598	NA	-0,0015413	4,25E-05	-0,00164139	NA	NA	-1,57337207	-0,04401868	0,05538852	NA	-0,0006675	NA	-0,48613452	10	-20657,7839	41335,5799	3499,16322	0
3670	-1,1987332	0,68175498	NA	4,69E-05	NA	0,40581671	NA	1,15818376	-0,19528017	NA	NA	-0,00061584	0,46809624	-0,5501339	10	-20658,8099	41337,632	3501,21526	0
3672	-1,59179834	0,69737855	0,00852736	4,60E-05	NA	0,40932129	NA	1,15818376	-0,19528017	NA	NA	-0,00061584	0,46809624	-0,5501339	11	-20657,8935	41337,8016	3501,38486	0
2221	0,64576877	NA	NA	3,88E-05	-0,00183092	NA	-0,20438761	-1,98285198	NA	0,054624	NA	NA	NA	-0,47270606	8	-20662,8764	41341,7607	3505,34396	0
2229	-0,84339357	NA	NA	4,07E-05	NA	0,55505772	-0,13640315	-1,98285198	NA	0,054624	NA	NA	NA	-0,47270606	8	-20663,1346	41342,2771	3505,86039	0
2285	0,64576877	NA	NA	3,88E-05	-0,00183092	NA	-0,20438761	-2,01223022	0,04141198	0,05511629	NA	NA	NA	-0,47466077	9	-20662,2698	41342,5495	3506,13277	0
2231	-0,50593593	NA	-0,00700816	4,14E-05	NA	0,55021643	-0,14061875	-1,98285198	NA	0,054624	NA	NA	NA	-0,47270606	9	-20662,5015	41343,013	3506,59626	0
2293	-0,84339357	NA	NA	4,07E-05	NA	0,55505772	-0,13640315	-2,01223022	0,04141198	0,05511629	NA	NA	NA	-0,47466077	9	-20662,528	41343,0659	3506,6492	0
3198	-0,06671637	0,57575795	NA	4,34E-05	-0,00071955	0,41051829	-0,17478186	0,7406183	-0,11449835	NA	NA	NA	0,46966671	-0,53963709	11	-20660,5829	41343,1804	3506,7637	0
3200	-0,47166397	0,58945108	0,00876766	4,25E-05	-0,00073797	0,4135805	-0,1717464	0,7406183	-0,11449835	NA	NA	NA	0,46966671	-0,53963709	12	-20659,6087	41343,2347	3506,81798	0
3646	-0,06671637	0,57575795	NA	4,34E-05	-0,00071955	0,41051829	-0,17478186	0,95852238	NA	NA	NA	-0,00035257	0,44577019	-0,55202249	11	-20660,7818	41343,5781	3507,16141	0
2223	0,74969745	NA	-0,00245756	3,91E-05	-0,00181949	NA	-0,20513804	-1,98285198	NA	0,054624	NA	NA	NA	-0,47270606	9	-20662,8008	41343,6115	3507,1948	0
3648	-0,47166397	0,58945108	0,00876766	4,25E-05	-0,00073797	0,4135805	-0,1717464	0,95852238	NA	NA	NA	-0,00035257	0,44577019	-0,55202249	12	-20659,8076	41343,6324	3507,21568	0
2295	-0,50593593	NA	-0,00700816	4,14E-05	NA	0,55021643	-0,14061875	-2,01223022	0,04141198	0,05511629	NA	NA	NA	-0,47466077	10	-20661,8949	41343,802	3507,38529	0
2287	0,74969745	NA	-0,00245756	3,91E-05	-0,00181949	NA	-0,20513804	-2,01223022	0,04141198	0,05511629	NA	NA	NA	-0,47466077	10	-20662,1942	41344,4006	3507,98383	0
2197	-1,36352414	NA	NA	4,29E-05	NA	0,54467715	NA	-1,98285198	NA	0,054624	NA	NA	NA	-0,47270606	7	-20666,675	41347,3561	3510,93938	0
2261	-1,36352414	NA	NA	4,29E-05	NA	0,54467715	NA	-2,01223022	0,04141198	0,05511629	NA	NA	NA	-0,47466077	8	-20666,0684	41348,1447	3511,72797	0
2199	-1,08960867	NA	-0,00576757	4,36E-05	NA	0,53973144	NA	-1,98285198	NA	0,054624	NA	NA	NA	-0,47270606	8	-20666,2427	41348,4933	3512,07657	0
2263	-1,08960867	NA	-0,00576757	4,36E-05	NA	0,53973144	NA	-2,01223022	0,04141198	0,05511629	NA	NA	NA	-0,47466077	9	-20665,6361	41349,2821	3512,86538	0
3134	-0,06671637	0,57575795	NA	4,34E-05	-0,00071955	0,41051829	-0,17478186	0,72537252	NA	NA	NA	NA	0,45346103	-0,54414773	10	-20665,2958	41350,6038	3514,1871	0
3136	-0,47166397	0,58945108	0,00876766	4,25E-05	-0,00073797	0,4135805	-0,1717464	0,72537252	NA	NA	NA	NA	0,45346103	-0,54414773	11	-20664,3216	41350,6579	3514,24116	0
3190	-0,67953035	0,68234123	NA	4,46E-05	NA	0,41541375	-0,13795142	0,7406183	-0,11449835	NA	NA	NA	0,46966671	-0,53963709	10	-20665,7695	41351,5512	3515,13449	0
3638	-0,67953035	0,68234123	NA	4,46E-05	NA	0,41541375	-0,13795142	0,95852238	NA	NA	NA	-0,00035257	0,44577019	-0,55202249	10	-20665,9684	41351,9489	3515,5322	0
3192	-1,04079715	0,69637088	0,00755047	4,39E-05	NA	0,41816409	-0,13461471	0,7406183	-0,11449835	NA	NA	NA	0,46966671	-0,53963709	11	-20665,0503	41352,1152	3515,69844	0
3640	-1,04079715	0,69637088	0,00755047	4,39E-05	NA	0,41816409	-0,13461471	0,95852238	NA	NA	NA	-0,00035257	0,44577019	-0,55202249	11	-20665,2491	41352,5129	3516,09614	0
3168	-1,28042467	0,61751101	0,00965662	4,55E-05	-0,00054807	0,40424516	NA	0,7406183	-0,11449835	NA	NA	NA	0,46966671	-0,53963709	11	-20665,8549	41353,7245	3517,30777	0
3166	-0,85074914	0,60334436	NA	4,65E-05	-0,00052401	0,40049867	NA	0,7406183	-0,11449835	NA	NA	NA	0,46966671	-0,53963709	10	-20667,0308	41354,0737	3517,657	0
3616	-1,28042467	0,61751101	0,00965662	4,55E-05	-0,00054807	0,40424516	NA	0,95852238	NA	NA	NA	-0,00035257	0,44577019	-0,55202249	11	-20666,0538	41354,1222	3517,70547	0
3614	-0,85074914	0,60334436	NA	4,65E-05	-0,00052401	0,40049867	NA	0,95852238	NA	NA	NA	-0,00035257	0,44577019	-0,55202249	10	-20667,2296	41354,4714	3518,05471	0
2189	-0,28011525	NA	NA	4,24E-05	-0,0016491	NA	NA	-1,98285198	NA	0,054624	NA	NA	NA	-0,47270606	7	-20671,1667	41356,3395	3519,9228	0
2253	-0,28011525	NA	NA	4,24E-05	-0,0016491	NA	NA	-2,01223022	0,04141198	0,05511629	NA	NA	NA	-0,47466077	8	-20670,5601	41357,1281	3520,71139	0
3158	-1,1987332	0,68175498	NA	4,69E-05	NA	0,40581671	NA	0,7406183	-0,11449835	NA	NA	NA	0,46966671	-0,53963709	9	-20669,9447	41357,8993	3521,48255	0
3160	-1,59179834	0,69737855	0,00852736	4,60E-05	NA	0,40932129	NA	0,7406183	-0,11449835	NA	NA	NA	0,46966671	-0,53963709	10	-20669,0282	41358,0686	3521,65192	0
2191	-0,2131598	NA	-0,0015413	4,25E-05	-0,00164139	NA	NA	-1,98285198	NA	0,054624	NA	NA	NA	-0,47270606	8	-20671,137	41358,2819	3521,86518	0
3606	-1,1987332	0,68175498	NA	4,69E-05	NA	0,40581671	NA	0,95852238	NA	NA	NA	-0,00035257	0,44577019	-0,55202249	9	-20670,1435	41358,297	3521,88025	0
3608	-1,59179834	0,69737855	0,00852736	4,60E-05	NA	0,40932129	NA	0,95852238	NA	NA	NA	-0,00035257	0,44577019	-0,55202249	10	-20669,2271	41358,4663	3522,04962	0

3126	-0,67953035	0,68234123	NA	4,46E-05	NA	0,41541375	-0,13795142	0,72537252	NA	NA	NA	NA	0,45346103	-0,54414773	9	-20670,4824	41358,9748	3522,55811	0
2255	-0,2131598	NA	-0,0015413	4,25E-05	-0,00164139	NA	NA	-2,01223022	0,04141198	0,05511629	NA	NA	NA	-0,47466077	9	-20670,5304	41359,0707	3522,65399	0
3128	-1,04079715	0,69637088	0,00755047	4,39E-05	NA	0,41816409	-0,13461471	0,72537252	NA	NA	NA	NA	0,45346103	-0,54414773	10	-20669,7632	41359,5386	3523,12183	0
3104	-1,28042467	0,61751101	0,00965662	4,55E-05	-0,00054807	0,40424516	NA	0,72537252	NA	NA	NA	NA	0,45346103	-0,54414773	10	-20670,5679	41361,1479	3524,73117	0
3102	-0,85074914	0,60334436	NA	4,65E-05	-0,00052401	0,40049867	NA	0,72537252	NA	NA	NA	NA	0,45346103	-0,54414773	9	-20671,7437	41361,4973	3525,08062	0
3094	-1,1987332	0,68175498	NA	4,69E-05	NA	0,40581671	NA	0,72537252	NA	NA	NA	NA	0,45346103	-0,54414773	8	-20674,6576	41365,3231	3528,90639	0
3096	-1,59179834	0,69737855	0,00852736	4,60E-05	NA	0,40932129	NA	0,72537252	NA	NA	NA	NA	0,45346103	-0,54414773	9	-20673,7412	41365,4923	3529,07554	0
3694	-0,14195779	0,65649184	NA	4,36E-05	-0,00074998	NA	-0,15445798	1,15818376	-0,19528017	NA	NA	-0,00061584	0,46809624	-0,5501339	11	-20674,9773	41371,9693	3535,55254	0
1790	-0,06671637	0,57575795	NA	4,34E-05	-0,00071955	0,41051829	-0,17478186	-3,6371906	-0,09814646	0,05972263	NA	-0,00047389	0,39127251	NA	12	-20674,0233	41372,0638	3535,64704	0
1792	-0,47166397	0,58945108	0,00876766	4,25E-05	-0,00073797	0,4135805	-0,1717464	-3,6371906	-0,09814646	0,05972263	NA	-0,00047389	0,39127251	NA	13	-20673,0491	41372,1183	3535,70154	0
3696	-0,45110821	0,66723944	0,00674107	4,29E-05	-0,00076432	NA	-0,15224001	1,15818376	-0,19528017	NA	NA	-0,00061584	0,46809624	-0,5501339	12	-20674,4192	41372,8556	3536,43888	0
1726	-0,06671637	0,57575795	NA	4,34E-05	-0,00071955	0,41051829	-0,17478186	-3,78227707	NA	0,0606519	NA	-0,00034645	0,38158141	NA	11	-20676,8595	41375,7336	3539,31687	0
1728	-0,47166397	0,58945108	0,00876766	4,25E-05	-0,00073797	0,4135805	-0,1717464	-3,78227707	NA	0,0606519	NA	-0,00034645	0,38158141	NA	12	-20675,8853	41375,7879	3539,37115	0
3662	-0,8455927	0,67951403	NA	4,64E-05	-0,00057321	NA	NA	1,15818376	-0,19528017	NA	NA	-0,00061584	0,46809624	-0,5501339	10	-20679,9978	41380,0078	3543,59105	0
1782	-0,67953035	0,68234123	NA	4,46E-05	NA	0,41541375	-0,13795142	-3,6371906	-0,09814646	0,05972263	NA	-0,00047389	0,39127251	NA	11	-20679,2099	41380,4343	3544,01761	0
3664	-1,17938783	0,69098467	0,00751019	4,56E-05	-0,00059207	NA	NA	1,15818376	-0,19528017	NA	NA	-0,00061584	0,46809624	-0,5501339	11	-20679,3079	41380,6303	3544,21359	0
1784	-1,04079715	0,69637088	0,00755047	4,39E-05	NA	0,41816409	-0,13461471	-3,6371906	-0,09814646	0,05972263	NA	-0,00047389	0,39127251	NA	12	-20678,4906	41380,9985	3544,58178	0
3686	-0,79586264	0,77122371	NA	4,50E-05	NA	NA	-0,11503973	1,15818376	-0,19528017	NA	NA	-0,00061584	0,46809624	-0,5501339	10	-20680,5715	41381,1552	3544,73848	0
1214	-0,06671637	0,57575795	NA	4,34E-05	-0,00071955	0,41051829	-0,17478186	-3,95454473	NA	0,05999672	NA	NA	0,38996522	NA	10	-20681,2026	41382,4175	3546,00073	0
3688	-1,05409025	0,78157333	0,00543311	4,44E-05	NA	NA	-0,11273577	1,15818376	-0,19528017	NA	NA	-0,00061584	0,46809624	-0,5501339	11	-20680,2112	41382,4371	3546,02034	0
1216	-0,47166397	0,58945108	0,00876766	4,25E-05	-0,00073797	0,4135805	-0,1717464	-3,95454473	NA	0,05999672	NA	NA	0,38996522	NA	11	-20680,2285	41382,4715	3546,05479	0
1760	-1,28042467	0,61751101	0,00965662	4,55E-05	-0,00054807	0,40424516	NA	-3,6371906	-0,09814646	0,05972263	NA	-0,00047389	0,39127251	NA	12	-20679,2953	41382,6078	3546,19111	0
1758	-0,85074914	0,60334436	NA	4,65E-05	-0,00052401	0,40049867	NA	-3,6371906	-0,09814646	0,05972263	NA	-0,00047389	0,39127251	NA	11	-20680,4711	41382,9568	3546,54012	0
1278	-0,06671637	0,57575795	NA	4,34E-05	-0,00071955	0,41051829	-0,17478186	-3,92430325	-0,03654601	0,05956532	NA	NA	0,39466847	NA	11	-20680,7315	41383,4776	3547,06089	0
1280	-0,47166397	0,58945108	0,00876766	4,25E-05	-0,00073797	0,4135805	-0,1717464	-3,92430325	-0,03654601	0,05956532	NA	NA	0,39466847	NA	12	-20679,7573	41383,5319	3547,11517	0
1718	-0,67953035	0,68234123	NA	4,46E-05	NA	0,41541375	-0,13795142	-3,78227707	NA	0,0606519	NA	-0,00034645	0,38158141	NA	10	-20682,0461	41384,1044	3547,68766	0
1720	-1,04079715	0,69637088	0,00755047	4,39E-05	NA	0,41816409	-0,13461471	-3,78227707	NA	0,0606519	NA	-0,00034645	0,38158141	NA	11	-20681,3269	41384,6683	3548,25161	0
3654	-1,235734	0,76857786	NA	4,69E-05	NA	NA	NA	1,15818376	-0,19528017	NA	NA	-0,00061584	0,46809624	-0,5501339	9	-20683,4702	41384,9503	3548,53355	0
3656	-1,52051301	0,78035551	0,00624724	4,63E-05	NA	NA	NA	1,15818376	-0,19528017	NA	NA	-0,00061584	0,46809624	-0,5501339	10	-20682,9941	41386,0003	3549,58358	0
1696	-1,28042467	0,61751101	0,00965662	4,55E-05	-0,00054807	0,40424516	NA	-3,78227707	NA	0,0606519	NA	-0,00034645	0,38158141	NA	11	-20682,1315	41386,2777	3549,86094	0
1694	-0,85074914	0,60334436	NA	4,65E-05	-0,00052401	0,40049867	NA	-3,78227707	NA	0,0606519	NA	-0,00034645	0,38158141	NA	10	-20683,3074	41386,6269	3550,21017	0
1750	-1,1987332	0,68175498	NA	4,69E-05	NA	0,40581671	NA	-3,6371906	-0,09814646	0,05972263	NA	-0,00047389	0,39127251	NA	10	-20683,385	41386,7822	3550,36545	0
1752	-1,59179834	0,69737855	0,00852736	4,60E-05	NA	0,40932129	NA	-3,6371906	-0,09814646	0,05972263	NA	-0,00047389	0,39127251	NA	11	-20682,4686	41386,9518	3550,53504	0
1686	-1,1987332	0,68175498	NA	4,69E-05	NA	0,40581671	NA	-3,78227707	NA	0,0606519	NA	-0,00034645	0,38158141	NA	9	-20686,2212	41390,4524	3554,03572	0
1688	-1,59179834	0,69737855	0,00852736	4,60E-05	NA	0,40932129	NA	-3,78227707	NA	0,0606519	NA	-0,00034645	0,38158141	NA	10	-20685,3048	41390,6218	3554,20509	0
1206	-0,67953035	0,68234123	NA	4,46E-05	NA	0,41541375	-0,13795142	-3,95454473	NA	0,05999672	NA	NA	0,38996522	NA	9	-20686,3893	41390,7885	3554,37174	0
1208	-1,04079715	0,69637088	0,00755047	4,39E-05	NA	0,41816409	-0,13461471	-3,95454473	NA	0,05999672	NA	NA	0,38996522	NA	10	-20685,67	41391,3522	3554,93546	0
1270	-0,67953035	0,68234123	NA	4,46E-05	NA	0,41541375	-0,13795142	-3,92430325	-0,03654601	0,05956532	NA	NA	0,39466847	NA	10	-20685,9181	41391,8484	3555,43168	0
3182	-0,14195779	0,65649184	NA	4,36E-05	-0,00074998	NA	-0,15445798	0,7406183	-0,11449835	NA	NA	NA	0,46966671	-0,53963709	10	-20686,1121	41392,2363	3555,8196	0
1272	-1,04079715	0,69637088	0,00755047	4,39E-05	NA	0,41816409	-0,13461471	-3,92430325	-0,03654601	0,05956532	NA	NA	0,39466847	NA	11	-20685,1989	41392,4123	3555,99562	0
3709	0,65451489	NA	NA	3,95E-05	-0,00164357	0,48762971	-0,22170047	1,15818376	-0,19528017	NA	NA	-0,00061584	0,46809624	-0,5501339	11	-20685,2754	41392,5655	3556,14873	0
3630	-0,14195779	0,65649184	NA	4,36E-05	-0,00074998	NA	-0,15445798	0,95852238	NA	NA	NA	-0,00035257	0,44577019	-0,55202249	10	-20686,3109	41392,634	3556,21731	0
1184	-1,28042467	0,61751101	0,00965662	4,55E-05	-0,00054807	0,40424516	NA	-3,95454473	NA	0,05999672	NA	NA	0,38996522	NA	10	-20686,4747	41392,9615	3556,5448	0
3184	-0,45110821	0,66723944	0,00674107	4,29E-05	-0,00076432	NA	-0,15224001	0,7406183	-0,11449835	NA	NA	NA	0,46966671	-0,53963709	11	-20685,5539	41393,1224	3556,70572	0
1182	-0,85074914	0,60334436	NA	4,65E-05	-0,00052401	0,40049867	NA	-3,95454473	NA	0,05999672	NA	NA	0,38996522	NA	9	-20687,6505	41393,311	3556,89425	0
3632	-0,45110821	0,66723944	0,00674107	4,29E-05	-0,00076432	NA	-0,15224001	0,95852238	NA	NA	NA	-0,00035257	0,44577019	-0,55202249	11	-20685,7528	41393,5201	3557,10342	0
1248	-1,28042467	0,61751101	0,00965662	4,55E-05	-0,00054807	0,40424516	NA	-3,92430325	-0,03654601	0,05956532	NA	NA	0,39466847	NA	11	-20686,0035	41394,0217	3557,60495	0
1246	-0,85074914	0,60334436	NA	4,65E-05	-0,00052401	0,40049867	NA	-3,92430325	-0,03654601	0,05956532	NA	NA	0,39466847	NA	10	-20687,1794	41394,3709	3557,95419	0
3711	0,61699723	NA	0,00084798	3,94E-05	-0,00164748	0,4880593	-0,22142621	1,15818376	-0,19528017	NA	NA	-0,00061584	0,46809624	-0,5501339	12	-20685,2661	41394,5495	3558,1328	0
1174	-1,1987332	0,68175498	NA	4,69E-05	NA	0,40581671	NA	-3,95454473	NA	0,05999672	NA	NA	0,38996522	NA	8	-20690,5644	41397,1367	3560,72002	0
1176	-1,59179834	0,69737855	0,00852736	4,60E-05	NA	0,40932129	NA	-3,95454473	NA	0,05999672	NA	NA	0,38996522	NA	9	-20689,648	41397,3059	3560,88917	0
1238	-1,1987332	0,68175498	NA	4,69E-05	NA	0,40581671	NA	-3,92430325	-0,03654601	0,05956532	NA	NA	0,39466847	NA	9	-20690,0933	41398,1965	3561,77973	0
1240	-1,59179834	0,69737855	0,00852736	4,60E-05	NA	0,40932129	NA	-3,92430325	-0,03654601	0,05956532	NA	NA	0,39466847	NA	10	-20689,1768	41398,3658	3561,9491	0
3118	-0,14195779	0,65649184	NA	4,36E-05	-0,00074998	NA	-0,15445798	0,72537252	NA	NA	NA	NA	0,45346103	-0,54414773	9	-20690,825	41399,6599	3563,24322	0
3150	-0,8455927	0,67951403	NA	4,64E-05	-0,00057321	NA	NA	0,7406183	-0,11449835	NA	NA	NA	0,46966671	-0,53963709	9	-20691,1326	41400,2751	3563,85834	0
3120	-0,45110821	0,66723944	0,00674107	4,29E-05	-0,00076432	NA	-0,15224001	0,72537252	NA	NA	NA	NA	0,45346103	-0,54414773	10	-20690,2668	41400,5458	3564,12912	0
3598	-0,8455927	0,67951403	NA	4,64E-05	-0,00057321	NA	NA	0,95852238	NA	NA	NA	-0,00035257	0,44577019	-0,55202249	9	-20691,3314	41400,6728	3564,25604	0
3152	-1,17938783	0,69098467	0,00751019	4,56E-05	-0,00059207	NA	NA	0,7406183	-0,11449835	NA	NA	NA	0,46966671	-0,53963709	10	-20690,4426	41400,8974	3564,48065	0
3600	-1,17938783	0,69098467	0,00751019	4,56E-05	-0,00059207	NA	NA	0,95852238	NA	NA	NA	-0,00035257	0,44577019	-0,55202249	10	-20690,6415	41401,2951	3564,87835	0
3174	-0,79586264	0,77122371	NA	4,50E-05	NA	NA	-0,11503973	0,7406183	-0,11449835	NA	NA	NA	0,46966671	-0,53963709	9	-20691,7063	41401,4225	3565,00576	0
3622	-0,79586264	0,77122371	NA	4,50E-05	NA	NA	-0,11503973	0,95852238	NA	NA	NA	-0,00035257	0,44577019	-0,55202249	9	-20691,9051	41401,8202	3565,40347	0

3176	-1,05409025	0,78157333	0,00543311	4,44E-05	NA	NA	-0,11273577	0,7406183	-0,11449835	NA	NA	NA	0,46966671	-0,53963709	10	-20691,346	41402,7041	3566,2874	0
3624	-1,05409025	0,78157333	0,00543311	4,44E-05	NA	NA	-0,11273577	0,95852238	NA	NA	NA	-0,00035257	0,44577019	-0,55202249	10	-20691,5448	41403,1018	3566,6851	0
3142	-1,235734	0,76857786	NA	4,69E-05	NA	NA	NA	0,7406183	-0,11449835	NA	NA	NA	0,46966671	-0,53963709	8	-20694,6049	41405,2178	3568,80106	0
2727	-0,65771633	NA	-0,01187559	4,14E-05	NA	NA	-0,10732692	-1,63848397	NA	0,05580518	NA	-0,00060814	NA	-0,48687105	9	-20693,635	41405,28	3568,86331	0
3590	-1,235734	0,76857786	NA	4,69E-05	NA	NA	NA	0,95852238	NA	NA	NA	-0,00035257	0,44577019	-0,55202249	8	-20694,8038	41405,6155	3569,19876	0
2791	-0,65771633	NA	-0,01187559	4,14E-05	NA	NA	-0,10732692	-1,57337207	-0,04401868	0,05538852	NA	-0,0006675	NA	-0,48613452	10	-20693,064	41406,1401	3569,72336	0
3144	-1,52051301	0,78035551	0,00624724	4,63E-05	NA	NA	NA	0,7406183	-0,11449835	NA	NA	NA	0,46966671	-0,53963709	9	-20694,1288	41406,2676	3569,85087	0
3592	-1,52051301	0,78035551	0,00624724	4,63E-05	NA	NA	NA	0,95852238	NA	NA	NA	-0,00035257	0,44577019	-0,55202249	9	-20694,3277	41406,6653	3570,24857	0
2725	-1,22822825	NA	NA	4,02E-05	NA	NA	-0,09995451	-1,63848397	NA	0,05580518	NA	-0,00060814	NA	-0,48687105	8	-20695,3795	41406,7669	3570,35022	0
2695	-1,14142692	NA	-0,01089562	4,31E-05	NA	NA	NA	-1,63848397	NA	0,05580518	NA	-0,00060814	NA	-0,48687105	8	-20695,7491	41407,5062	3571,08953	0
2789	-1,22822825	NA	NA	4,02E-05	NA	NA	-0,09995451	-1,57337207	-0,04401868	0,05538852	NA	-0,0006675	NA	-0,48613452	9	-20694,8084	41407,6268	3571,21004	0
3086	-0,8455927	0,67951403	NA	4,64E-05	-0,00057321	NA	NA	0,72537252	NA	NA	NA	NA	0,45346103	-0,54414773	8	-20695,8455	41407,6989	3571,28218	0
3088	-1,17938783	0,69098467	0,00751019	4,56E-05	-0,00059207	NA	NA	0,72537252	NA	NA	NA	NA	0,45346103	-0,54414773	9	-20695,1555	41408,321	3571,90427	0
2759	-1,14142692	NA	-0,01089562	4,31E-05	NA	NA	NA	-1,57337207	-0,04401868	0,05538852	NA	-0,0006675	NA	-0,48613452	9	-20695,1781	41408,3661	3571,94935	0
2693	-1,63084607	NA	NA	4,18E-05	NA	NA	NA	-1,63848397	NA	0,05580518	NA	-0,00060814	NA	-0,48687105	7	-20697,2243	41408,4548	3572,03812	0
3110	-0,79586264	0,77122371	NA	4,50E-05	NA	NA	-0,11503973	0,72537252	NA	NA	NA	NA	0,45346103	-0,54414773	8	-20696,4192	41408,8463	3572,42961	0
2757	-1,63084607	NA	NA	4,18E-05	NA	NA	NA	-1,57337207	-0,04401868	0,05538852	NA	-0,0006675	NA	-0,48613452	8	-20696,6532	41409,3144	3572,89772	0
3112	-1,05409025	0,78157333	0,00543311	4,44E-05	NA	NA	-0,11273577	0,72537252	NA	NA	NA	NA	0,45346103	-0,54414773	9	-20696,0589	41410,1277	3573,71102	0
3677	-0,31227401	NA	NA	4,32E-05	-0,00145314	0,47935513	NA	1,15818376	-0,19528017	NA	NA	-0,00061584	0,46809624	-0,5501339	10	-20695,1783	41410,3687	3573,95199	0
3679	-0,39160679	NA	0,00183718	4,30E-05	-0,00146197	0,48040455	NA	1,15818376	-0,19528017	NA	NA	-0,00061584	0,46809624	-0,5501339	11	-20695,1349	41412,2843	3575,8676	0
3078	-1,235734	0,76857786	NA	4,69E-05	NA	NA	NA	0,72537252	NA	NA	NA	NA	0,45346103	-0,54414773	7	-20699,3178	41412,6418	3576,22512	0
3197	0,65451489	NA	NA	3,95E-05	-0,00164357	0,48762971	-0,22170047	0,7406183	-0,11449835	NA	NA	NA	0,46966671	-0,53963709	10	-20696,4102	41412,8325	3576,41579	0
3645	0,65451489	NA	NA	3,95E-05	-0,00164357	0,48762971	-0,22170047	0,95852238	NA	NA	NA	-0,00035257	0,44577019	-0,55202249	10	-20696,609	41413,2302	3576,8135	0
3080	-1,52051301	0,78035551	0,00624724	4,63E-05	NA	NA	NA	0,72537252	NA	NA	NA	NA	0,45346103	-0,54414773	8	-20698,8417	41413,6914	3577,27471	0
3199	0,61699723	NA	0,00084798	3,94E-05	-0,00164748	0,4880593	-0,22142621	0,7406183	-0,11449835	NA	NA	NA	0,46966671	-0,53963709	11	-20696,4009	41414,8164	3578,39964	0
3647	0,61699723	NA	0,00084798	3,94E-05	-0,00164748	0,4880593	-0,22142621	0,95852238	NA	NA	NA	-0,00035257	0,44577019	-0,55202249	11	-20696,5997	41415,2141	3578,79734	0
3133	0,65451489	NA	NA	3,95E-05	-0,00164357	0,48762971	-0,22170047	0,72537252	NA	NA	NA	NA	0,45346103	-0,54414773	9	-20701,1231	41420,2561	3583,83941	0
1774	-0,14195779	0,65649184	NA	4,36E-05	-0,00074998	NA	-0,15445798	-3,6371906	-0,09814646	0,05972263	NA	-0,00047389	0,39127251	NA	11	-20699,5524	41421,1194	3584,70272	0
1776	-0,45110821	0,66723944	0,00674107	4,29E-05	-0,00076432	NA	-0,15224001	-3,6371906	-0,09814646	0,05972263	NA	-0,00047389	0,39127251	NA	12	-20698,9943	41422,0058	3585,58906	0
3135	0,61699723	NA	0,00084798	3,94E-05	-0,00164748	0,4880593	-0,22142621	0,72537252	NA	NA	NA	NA	0,45346103	-0,54414773	10	-20701,1138	41422,2398	3585,82304	0
1710	-0,14195779	0,65649184	NA	4,36E-05	-0,00074998	NA	-0,15445798	-3,78227707	NA	0,0606519	NA	-0,00034645	0,38158141	NA	10	-20702,3887	41424,7895	3588,37277	0
1712	-0,45110821	0,66723944	0,00674107	4,29E-05	-0,00076432	NA	-0,15224001	-3,78227707	NA	0,0606519	NA	-0,00034645	0,38158141	NA	11	-20701,8305	41425,6756	3589,25889	0
2215	-0,65771633	NA	-0,01187559	4,14E-05	NA	NA	-0,10732692	-1,98285198	NA	0,054624	NA	NA	NA	-0,47270606	8	-20706,417	41428,842	3592,42532	0
1742	-0,8455927	0,67951403	NA	4,64E-05	-0,00057321	NA	NA	-3,6371906	-0,09814646	0,05972263	NA	-0,00047389	0,39127251	NA	10	-20704,5729	41429,158	3592,74123	0
2279	-0,65771633	NA	-0,01187559	4,14E-05	NA	NA	-0,10732692	-2,01223022	0,04141198	0,05511629	NA	NA	NA	-0,47466077	9	-20705,8105	41429,6309	3593,21413	0
1744	-1,17938783	0,69098467	0,00751019	4,56E-05	-0,00059207	NA	NA	-3,6371906	-0,09814646	0,05972263	NA	-0,00047389	0,39127251	NA	11	-20703,8829	41429,7805	3593,36377	0
1766	-0,79586264	0,77122371	NA	4,50E-05	NA	NA	-0,11503973	-3,6371906	-0,09814646	0,05972263	NA	-0,00047389	0,39127251	NA	10	-20705,1466	41430,3054	3593,88866	0
2213	-1,22822825	NA	NA	4,02E-05	NA	NA	-0,09995451	-1,98285198	NA	0,054624	NA	NA	NA	-0,47270606	7	-20708,1615	41430,3292	3593,91245	0
3165	-0,31227401	NA	NA	4,32E-05	-0,00145314	0,47935513	NA	0,7406183	-0,11449835	NA	NA	NA	0,46966671	-0,53963709	9	-20706,313	41430,636	3594,21927	0
3613	-0,31227401	NA	NA	4,32E-05	-0,00145314	0,47935513	NA	0,95852238	NA	NA	NA	-0,00035257	0,44577019	-0,55202249	9	-20706,5119	41431,0337	3594,61698	0
2183	-1,14142692	NA	-0,01089562	4,31E-05	NA	NA	NA	-1,98285198	NA	0,054624	NA	NA	NA	-0,47270606	7	-20708,5311	41431,0685	3594,65175	0
2277	-1,22822825	NA	NA	4,02E-05	NA	NA	-0,09995451	-2,01223022	0,04141198	0,05511629	NA	NA	NA	-0,47466077	8	-20707,5549	41431,1178	3594,70104	0
1198	-0,14195779	0,65649184	NA	4,36E-05	-0,00074998	NA	-0,15445798	-3,95454473	NA	0,05999672	NA	NA	0,38996522	NA	9	-20706,7318	41431,4736	3595,05685	0
1768	-1,05409025	0,78157333	0,00543311	4,44E-05	NA	NA	-0,11273577	-3,6371906	-0,09814646	0,05972263	NA	-0,00047389	0,39127251	NA	11	-20704,7863	41431,5872	3595,17052	0
2247	-1,14142692	NA	-0,01089562	4,31E-05	NA	NA	NA	-2,01223022	0,04141198	0,05511629	NA	NA	NA	-0,47466077	8	-20707,9246	41431,8571	3595,44034	0
2181	-1,63084607	NA	NA	4,18E-05	NA	NA	NA	-1,98285198	NA	0,054624	NA	NA	NA	-0,47270606	6	-20710,0063	41432,0173	3595,60057	0
1200	-0,45110821	0,66723944	0,00674107	4,29E-05	-0,00076432	NA	-0,15224001	-3,95454473	NA	0,05999672	NA	NA	0,38996522	NA	10	-20706,1736	41432,3595	3595,94275	0
1262	-0,14195779	0,65649184	NA	4,36E-05	-0,00074998	NA	-0,15445798	-3,92430325	-0,03654601	0,05956532	NA	NA	0,39466847	NA	10	-20706,2607	41432,5335	3596,11679	0
3167	-0,39160679	NA	0,00183718	4,30E-05	-0,00146197	0,48040455	NA	0,7406183	-0,11449835	NA	NA	NA	0,46966671	-0,53963709	10	-20706,2696	41432,5514	3596,13466	0
2245	-1,63084607	NA	NA	4,18E-05	NA	NA	NA	-2,01223022	0,04141198	0,05511629	NA	NA	NA	-0,47466077	7	-20709,3997	41432,8057	3596,38894	0
1678	-0,8455927	0,67951403	NA	4,64E-05	-0,00057321	NA	NA	-3,78227707	NA	0,0606519	NA	-0,00034645	0,38158141	NA	9	-20707,4091	41432,8282	3596,41151	0
3615	-0,39160679	NA	0,00183718	4,30E-05	-0,00146197	0,48040455	NA	0,95852238	NA	NA	NA	-0,00035257	0,44577019	-0,55202249	10	-20706,4685	41432,9491	3596,53237	0
1264	-0,45110821	0,66723944	0,00674107	4,29E-05	-0,00076432	NA	-0,15224001	-3,92430325	-0,03654601	0,05956532	NA	NA	0,39466847	NA	11	-20705,7025	41433,4196	3597,0029	0
1680	-1,17938783	0,69098467	0,00751019	4,56E-05	-0,00059207	NA	NA	-3,78227707	NA	0,0606519	NA	-0,00034645	0,38158141	NA	10	-20706,7192	41433,4505	3597,03382	0
1702	-0,79586264	0,77122371	NA	4,50E-05	NA	NA	-0,11503973	-3,78227707	NA	0,0606519	NA	-0,00034645	0,38158141	NA	9	-20707,9829	41433,9757	3597,55893	0
1734	-1,235734	0,76857786	NA	4,69E-05	NA	NA	NA	-3,6371906	-0,09814646	0,05972263	NA	-0,00047389	0,39127251	NA	9	-20708,0453	41434,1005	3597,68374	0
1736	-1,52051301	0,78035551	0,00624724	4,63E-05	NA	NA	NA	-3,6371906	-0,09814646	0,05972263	NA	-0,00047389	0,39127251	NA	10	-20707,5692	41435,1505	3598,73377	0
1704	-1,05409025	0,78157333	0,00543311	4,44E-05	NA	NA	-0,11273577	-3,78227707	NA	0,0606519	NA	-0,00034645	0,38158141	NA	10	-20707,6226	41435,2573	3598,84057	0
1670	-1,235734	0,76857786	NA	4,69E-05	NA	NA	NA	-3,78227707	NA	0,0606519	NA	-0,00034645	0,38158141	NA	8	-20710,8815	41437,7709	3601,35423	0
3101	-0,31227401	NA	NA	4,32E-05	-0,00145314	0,47935513	NA	0,72537252	NA	NA	NA	NA	0,45346103	-0,54414773	8	-20711,0259	41438,0598	3601,64312	0
1672	-1,52051301	0,78035551	0,00624724	4,63E-05	NA	NA	NA	-3,78227707	NA	0,0606519	NA	-0,00034645	0,38158141	NA	9	-20710,4054	41438,8208	3602,40404	0

1166	-0,8455927	0,67951403	NA	4,64E-05	-0,00057321	NA	NA	-3,95454473	NA	0,05999672	NA	NA	0,38996522	NA	8	-20711,7523	41439,5125	3603,09581	0
3103	-0,39160679	NA	0,00183718	4,30E-05	-0,00146197	0,48040455	NA	0,72537252	NA	NA	NA	NA	0,45346103	-0,54414773	9	-20710,9825	41439,975	3603,55828	0
1168	-1,17938783	0,69098467	0,00751019	4,56E-05	-0,00059207	NA	NA	-3,95454473	NA	0,05999672	NA	NA	0,38996522	NA	9	-20711,0623	41440,1346	3603,7179	0
1230	-0,8455927	0,67951403	NA	4,64E-05	-0,00057321	NA	NA	-3,92430325	-0,03654601	0,05956532	NA	NA	0,39466847	NA	9	-20711,2811	41440,5722	3604,15552	0
1190	-0,79586264	0,77122371	NA	4,50E-05	NA	NA	-0,11503973	-3,95454473	NA	0,05999672	NA	NA	0,38996522	NA	8	-20712,326	41440,66	3604,24324	0
1232	-1,17938783	0,69098467	0,00751019	4,56E-05	-0,00059207	NA	NA	-3,92430325	-0,03654601	0,05956532	NA	NA	0,39466847	NA	10	-20710,5912	41441,1946	3604,77783	0
1789	0,65451489	NA	NA	3,95E-05	-0,00164357	0,48762971	-0,22170047	-3,6371906	-0,09814646	0,05972263	NA	-0,00047389	0,39127251	NA	11	-20709,8505	41441,7156	3605,29891	0
1254	-0,79586264	0,77122371	NA	4,50E-05	NA	NA	-0,11503973	-3,92430325	-0,03654601	0,05956532	NA	NA	0,39466847	NA	9	-20711,8549	41441,7197	3605,30295	0
1192	-1,05409025	0,78157333	0,00543311	4,44E-05	NA	NA	-0,11273577	-3,95454473	NA	0,05999672	NA	NA	0,38996522	NA	9	-20711,9657	41441,9414	3605,52465	0
1256	-1,05409025	0,78157333	0,00543311	4,44E-05	NA	NA	-0,11273577	-3,92430325	-0,03654601	0,05956532	NA	NA	0,39466847	NA	10	-20711,4946	41443,0013	3606,58459	0
1791	0,61699723	NA	0,00084798	3,94E-05	-0,00164748	0,4880593	-0,22142621	-3,6371906	-0,09814646	0,05972263	NA	-0,00047389	0,39127251	NA	12	-20709,8412	41443,6997	3607,28298	0
1158	-1,235734	0,76857786	NA	4,69E-05	NA	NA	NA	-3,95454473	NA	0,05999672	NA	NA	0,38996522	NA	7	-20715,2246	41444,4555	3608,03875	0
1725	0,65451489	NA	NA	3,95E-05	-0,00164357	0,48762971	-0,22170047	-3,78227707	NA	0,0606519	NA	-0,00034645	0,38158141	NA	10	-20712,6868	41445,3857	3608,96896	0
1160	-1,52051301	0,78035551	0,00624724	4,63E-05	NA	NA	NA	-3,95454473	NA	0,05999672	NA	NA	0,38996522	NA	8	-20714,7485	41445,5051	3609,08834	0
1222	-1,235734	0,76857786	NA	4,69E-05	NA	NA	NA	-3,92430325	-0,03654601	0,05956532	NA	NA	0,39466847	NA	8	-20714,7535	41445,515	3609,09824	0
1224	-1,52051301	0,78035551	0,00624724	4,63E-05	NA	NA	NA	-3,92430325	-0,03654601	0,05956532	NA	NA	0,39466847	NA	9	-20714,2774	41446,5648	3610,14805	0
1727	0,61699723	NA	0,00084798	3,94E-05	-0,00164748	0,4880593	-0,22142621	-3,78227707	NA	0,0606519	NA	-0,00034645	0,38158141	NA	11	-20712,6775	41447,3695	3610,95281	0
1213	0,65451489	NA	NA	3,95E-05	-0,00164357	0,48762971	-0,22170047	-3,95454473	NA	0,05999672	NA	NA	0,38996522	NA	9	-20717,0299	41452,0698	3615,65304	0
1277	0,65451489	NA	NA	3,95E-05	-0,00164357	0,48762971	-0,22170047	-3,92430325	-0,03654601	0,05956532	NA	NA	0,39466847	NA	10	-20716,5588	41453,1297	3616,71298	0
1215	0,61699723	NA	0,00084798	3,94E-05	-0,00164748	0,4880593	-0,22142621	-3,95454473	NA	0,05999672	NA	NA	0,38996522	NA	10	-20717,0206	41454,0534	3617,63667	0
1279	0,61699723	NA	0,00084798	3,94E-05	-0,00164748	0,4880593	-0,22142621	-3,92430325	-0,03654601	0,05956532	NA	NA	0,39466847	NA	11	-20716,5495	41455,1135	3618,69682	0
1757	-0,31227401	NA	NA	4,32E-05	-0,00145314	0,47935513	NA	-3,6371906	-0,09814646	0,05972263	NA	-0,00047389	0,39127251	NA	10	-20719,7534	41459,5189	3623,10217	0
3836	-5,95790631	0,3741151	0,04613303	NA	-0,00180249	0,39660544	-0,62074526	-1,61532922	-0,10289105	0,0570521	NA	-0,00065333	0,49801214	-0,5357095	13	-20716,8883	41459,7968	3623,38008	0
1759	-0,39160679	NA	0,00183718	4,30E-05	-0,00146197	0,48040455	NA	-3,6371906	-0,09814646	0,05972263	NA	-0,00047389	0,39127251	NA	11	-20719,71	41461,4345	3625,01778	0
3693	0,64576877	NA	NA	3,88E-05	-0,00183092	NA	-0,20438761	1,15818376	-0,19528017	NA	NA	-0,00061584	0,46809624	-0,5501339	10	-20720,8802	41461,7727	3625,35594	0
3701	-0,84339357	NA	NA	4,07E-05	NA	0,55505772	-0,13640315	1,15818376	-0,19528017	NA	NA	-0,00061584	0,46809624	-0,5501339	10	-20721,1385	41462,2891	3625,87237	0
3703	-0,50593593	NA	-0,00700816	4,14E-05	NA	0,55021643	-0,14061875	1,15818376	-0,19528017	NA	NA	-0,00061584	0,46809624	-0,5501339	11	-20720,5054	41463,0254	3626,60868	0
1693	-0,31227401	NA	NA	4,32E-05	-0,00145314	0,47935513	NA	-3,78227707	NA	0,0606519	NA	-0,00034645	0,38158141	NA	9	-20722,5896	41463,1892	3626,77244	0
3695	0,74969745	NA	-0,00245756	3,91E-05	-0,00181949	NA	-0,20513804	1,15818376	-0,19528017	NA	NA	-0,00061584	0,46809624	-0,5501339	11	-20720,8047	41463,6239	3627,20722	0
3772	-5,95790631	0,3741151	0,04613303	NA	-0,00180249	0,39660544	-0,62074526	-1,76635397	NA	0,05800383	NA	-0,00051675	0,48731517	-0,53620329	12	-20719,9325	41463,8822	3627,46551	0
1695	-0,39160679	NA	0,00183718	4,30E-05	-0,00146197	0,48040455	NA	-3,78227707	NA	0,0606519	NA	-0,00034645	0,38158141	NA	10	-20722,5462	41465,1046	3628,68783	0
3669	-1,36352414	NA	NA	4,29E-05	NA	0,54467715	NA	1,15818376	-0,19528017	NA	NA	-0,00061584	0,46809624	-0,5501339	9	-20724,6788	41467,3676	3630,95092	0
3671	-1,08960867	NA	-0,00576757	4,36E-05	NA	0,53973144	NA	1,15818376	-0,19528017	NA	NA	-0,00061584	0,46809624	-0,5501339	10	-20724,2466	41468,5053	3632,08855	0
1181	-0,31227401	NA	NA	4,32E-05	-0,00145314	0,47935513	NA	-3,95454473	NA	0,05999672	NA	NA	0,38996522	NA	8	-20726,9328	41469,8735	3633,45675	0
2686	-0,06671637	0,57575795	NA	4,34E-05	-0,00071955	0,41051829	-0,17478186	1,12236839	-0,1349839	NA	NA	-0,00063299	NA	-0,50295006	11	-20724,0327	41470,0799	3633,66319	0
2688	-0,47166397	0,58945108	0,00876766	4,25E-05	-0,00073797	0,4135805	-0,1717464	1,12236839	-0,1349839	NA	NA	-0,00063299	NA	-0,50295006	12	-20723,0585	41470,1342	3633,71747	0
1245	-0,31227401	NA	NA	4,32E-05	-0,00145314	0,47935513	NA	-3,92430325	-0,03654601	0,05956532	NA	NA	0,39466847	NA	9	-20726,4616	41470,9332	3634,51646	0
1183	-0,39160679	NA	0,00183718	4,30E-05	-0,00146197	0,48040455	NA	-3,95454473	NA	0,05999672	NA	NA	0,38996522	NA	9	-20726,8893	41471,7886	3635,37191	0
1247	-0,39160679	NA	0,00183718	4,30E-05	-0,00146197	0,48040455	NA	-3,92430325	-0,03654601	0,05956532	NA	NA	0,39466847	NA	10	-20726,4182	41472,8486	3636,43185	0
3661	-0,28011525	NA	NA	4,24E-05	-0,0016491	NA	NA	1,15818376	-0,19528017	NA	NA	-0,00061584	0,46809624	-0,5501339	9	-20729,1706	41476,3511	3639,93434	0
702	-0,06671637	0,57575795	NA	4,34E-05	-0,00071955	0,41051829	-0,17478186	-3,52383686	NA	0,05863003	NA	-0,00043937	NA	NA	10	-20728,4049	41476,822	3640,40525	0
704	-0,47166397	0,58945108	0,00876766	4,25E-05	-0,00073797	0,4135805	-0,1717464	-3,52383686	NA	0,05863003	NA	-0,00043937	NA	NA	11	-20727,4307	41476,876	3640,45931	0
766	-0,06671637	0,57575795	NA	4,34E-05	-0,00071955	0,41051829	-0,17478186	-3,4425274	-0,05282396	0,05811259	NA	-0,00050954	NA	NA	11	-20727,5672	41477,149	3640,73226	0
768	-0,47166397	0,58945108	0,00876766	4,25E-05	-0,00073797	0,4135805	-0,1717464	-3,4425274	-0,05282396	0,05811259	NA	-0,00050954	NA	NA	12	-20726,593	41477,2033	3640,78654	0
3663	-0,2131598	NA	-0,0015413	4,25E-05	-0,00164139	NA	NA	1,15818376	-0,19528017	NA	NA	-0,00061584	0,46809624	-0,5501339	10	-20729,1409	41478,2939	3641,87716	0
2678	-0,67953035	0,68234123	NA	4,46E-05	NA	0,41541375	-0,13795142	1,12236839	-0,1349839	NA	NA	-0,00063299	NA	-0,50295006	10	-20729,2193	41478,4507	3642,03398	0
2680	-1,04079715	0,69637088	0,00755047	4,39E-05	NA	0,41816409	-0,13461471	1,12236839	-0,1349839	NA	NA	-0,00063299	NA	-0,50295006	11	-20728,5	41479,0146	3642,59792	0
2622	-0,06671637	0,57575795	NA	4,34E-05	-0,00071955	0,41051829	-0,17478186	0,98389174	NA	NA	NA	-0,00044751	NA	-0,50601759	10	-20729,5863	41479,1847	3642,76796	0
2624	-0,47166397	0,58945108	0,00876766	4,25E-05	-0,00073797	0,4135805	-0,1717464	0,98389174	NA	NA	NA	-0,00044751	NA	-0,50601759	11	-20728,6121	41479,2387	3642,82202	0
3260	-5,95790631	0,3741151	0,04613303	NA	-0,00180249	0,39660544	-0,62074526	-2,06058113	NA	0,05699765	NA	NA	0,49693597	-0,52441365	11	-20729,1772	41480,369	3643,95229	0
2656	-1,28042467	0,61751101	0,00965662	4,55E-05	-0,00054807	0,40424516	NA	1,12236839	-0,1349839	NA	NA	-0,00063299	NA	-0,50295006	11	-20729,3047	41480,624	3644,20725	0
2654	-0,85074914	0,60334436	NA	4,65E-05	-0,00052401	0,40049867	NA	1,12236839	-0,1349839	NA	NA	-0,00063299	NA	-0,50295006	10	-20730,4805	41480,9732	3644,55649	0
3835	-8,35316771	NA	0,03864408	NA	-0,00234345	0,43617682	-0,64441699	-1,61532922	-0,10289105	0,0570521	NA	-0,00065333	0,49801214	-0,5357095	12	-20728,794	41481,6052	3645,18851	0
3181	0,64576877	NA	NA	3,88E-05	-0,00183092	NA	-0,20438761	0,7406183	-0,11449835	NA	NA	NA	0,46966671	-0,53963709	9	-20732,015	41482,0399	3645,62322	0
3324	-5,95790631	0,3741151	0,04613303	NA	-0,00180249	0,39660544	-0,62074526	-2,04753199	-0,01846226	0,05678211	NA	NA	0,49927467	-0,52377486	12	-20729,0595	41482,1362	3645,71953	0
3629	0,64576877	NA	NA	3,88E-05	-0,00183092	NA	-0,20438761	0,95852238	NA	NA	NA	-0,00035257	0,44577019	-0,55202249	9	-20732,2138	41482,4376	3646,02093	0
3189	-0,84339357	NA	NA	4,07E-05	NA	0,55505772	-0,13640315	0,7406183	-0,11449835	NA	NA	NA	0,46966671	-0,53963709	9	-20732,2732	41482,5564	3646,13965	0
3637	-0,84339357	NA	NA	4,07E-05	NA	0,55505772	-0,13640315	0,95852238	NA	NA	NA	-0,00035257	0,44577019	-0,55202249	9	-20732,4721	41482,9541	3646,53736	0
3191	-0,50593593	NA	-0,00700816	4,14E-05	NA	0,55021643	-0,14061875	0,7406183	-0,11449835	NA	NA	NA	0,46966671	-0,53963709	10	-20731,6401	41483,2925	3646,87574	0
3639	-0,50593593	NA	-0,00700816	4,14E-05	NA	0,55021643	-0,14061875	0,95852238	NA	NA	NA	-0,00035257	0,44577019	-0,55202249	10	-20731,839	41483,6902	3647,27345	0
3183	0,74969745	NA	-0,00245756	3,91E-05	-0,00181949	NA	-0,20513804	0,7406183	-0,11449835	NA	NA	NA	0,46966671	-0,53963709	10	-20731,9394	41483,891	3647,47428	0

3631	0,74969745	NA	-0,00245756	3,91E-05	-0,00181949	NA	-0,20513804	0,95852238	NA	NA	NA	-0,00035257	0,44577019	-0,55202249	10	-20732,1383	41484,2887	3647,87199	0
2646	-1,1987332	0,68175498	NA	4,69E-05	NA	0,40581671	NA	1,12236839	-0,1349839	NA	NA	-0,00063299	NA	-0,50295006	9	-20733,3944	41484,7988	3648,38203	0
2648	-1,59179834	0,69737855	0,00852736	4,60E-05	NA	0,40932129	NA	1,12236839	-0,1349839	NA	NA	-0,00063299	NA	-0,50295006	10	-20732,478	41484,9681	3648,5514	0
694	-0,67953035	0,68234123	NA	4,46E-05	NA	0,41541375	-0,13795142	-3,52383686	NA	0,05863003	NA	-0,00043937	NA	NA	9	-20733,5915	41485,193	3648,77626	0
758	-0,67953035	0,68234123	NA	4,46E-05	NA	0,41541375	-0,13795142	-3,4425274	-0,05282396	0,05811259	NA	-0,00050954	NA	NA	10	-20732,7538	41485,5198	3649,10305	0
3771	-8,35316771	NA	0,03864408	NA	-0,00234345	0,43617682	-0,64441699	-1,76635397	NA	0,05800383	NA	-0,00051675	0,48731517	-0,53620329	11	-20731,8381	41485,6909	3649,27417	0
696	-1,04079715	0,69637088	0,00755047	4,39E-05	NA	0,41816409	-0,13461471	-3,52383686	NA	0,05863003	NA	-0,00043937	NA	NA	10	-20732,8723	41485,7567	3649,33999	0
760	-1,04079715	0,69637088	0,00755047	4,39E-05	NA	0,41816409	-0,13461471	-3,4425274	-0,05282396	0,05811259	NA	-0,00050954	NA	NA	11	-20732,0346	41486,0837	3649,667	0
672	-1,28042467	0,61751101	0,00965662	4,55E-05	-0,00054807	0,40424516	NA	-3,52383686	NA	0,05863003	NA	-0,00043937	NA	NA	10	-20733,6769	41487,366	3650,94932	0
2614	-0,67953035	0,68234123	NA	4,46E-05	NA	0,41541375	-0,13795142	0,98389174	NA	NA	NA	-0,00044751	NA	-0,50601759	9	-20734,7729	41487,5557	3651,13897	0
3157	-1,36352414	NA	NA	4,29E-05	NA	0,54467715	NA	0,7406183	-0,11449835	NA	NA	NA	0,46966671	-0,53963709	8	-20735,8136	41487,6351	3651,21842	0
736	-1,28042467	0,61751101	0,00965662	4,55E-05	-0,00054807	0,40424516	NA	-3,4425274	-0,05282396	0,05811259	NA	-0,00050954	NA	NA	11	-20732,8392	41487,693	3651,27633	0
670	-0,85074914	0,60334436	NA	4,65E-05	-0,00052401	0,40049867	NA	-3,52383686	NA	0,05863003	NA	-0,00043937	NA	NA	9	-20734,8528	41487,7155	3651,29877	0
3605	-1,36352414	NA	NA	4,29E-05	NA	0,54467715	NA	0,95852238	NA	NA	NA	-0,00035257	0,44577019	-0,55202249	8	-20736,0124	41488,0328	3651,61613	0
734	-0,85074914	0,60334436	NA	4,65E-05	-0,00052401	0,40049867	NA	-3,4425274	-0,05282396	0,05811259	NA	-0,00050954	NA	NA	10	-20734,0151	41488,0423	3651,62556	0
2616	-1,04079715	0,69637088	0,00755047	4,39E-05	NA	0,41816409	-0,13461471	0,98389174	NA	NA	NA	-0,00044751	NA	-0,50601759	10	-20734,0536	41488,1194	3651,7027	0
190	-0,06671637	0,57575795	NA	4,34E-05	-0,00071955	0,41051829	-0,17478186	-3,73836005	NA	0,05777952	NA	NA	NA	NA	9	-20735,3588	41488,7276	3652,31084	0
3159	-1,08960867	NA	-0,00576757	4,36E-05	NA	0,53973144	NA	0,7406183	-0,11449835	NA	NA	NA	0,46966671	-0,53963709	9	-20735,3813	41488,7726	3652,35584	0
192	-0,47166397	0,58945108	0,00876766	4,25E-05	-0,00073797	0,4135805	-0,1717464	-3,73836005	NA	0,05777952	NA	NA	NA	NA	10	-20734,3846	41488,7814	3652,36467	0
3607	-1,08960867	NA	-0,00576757	4,36E-05	NA	0,53973144	NA	0,95852238	NA	NA	NA	-0,00035257	0,44577019	-0,55202249	9	-20735,5802	41489,1703	3652,75354	0
3117	0,64576877	NA	NA	3,88E-05	-0,00183092	NA	-0,20438761	0,72537252	NA	NA	NA	NA	0,45346103	-0,54414773	8	-20736,7279	41489,4638	3653,04706	0
2592	-1,28042467	0,61751101	0,00965662	4,55E-05	-0,00054807	0,40424516	NA	0,98389174	NA	NA	NA	-0,00044751	NA	-0,50601759	10	-20734,8583	41489,7287	3653,31203	0
3125	-0,84339357	NA	NA	4,07E-05	NA	0,55505772	-0,13640315	0,72537252	NA	NA	NA	NA	0,45346103	-0,54414773	8	-20736,9861	41489,9802	3653,5635	0
2590	-0,85074914	0,60334436	NA	4,65E-05	-0,00052401	0,40049867	NA	0,98389174	NA	NA	NA	-0,00044751	NA	-0,50601759	9	-20736,0341	41490,0782	3653,66148	0
254	-0,06671637	0,57575795	NA	4,34E-05	-0,00071955	0,41051829	-0,17478186	-3,74999095	0,01316736	0,05794033	NA	NA	NA	NA	10	-20735,2963	41490,6048	3654,18805	0
256	-0,47166397	0,58945108	0,00876766	4,25E-05	-0,00073797	0,4135805	-0,1717464	-3,74999095	0,01316736	0,05794033	NA	NA	NA	NA	11	-20734,3221	41490,6588	3654,24211	0
3127	-0,50593593	NA	-0,00700816	4,14E-05	NA	0,55021643	-0,14061875	0,72537252	NA	NA	NA	NA	0,45346103	-0,54414773	9	-20736,3531	41490,7161	3654,29937	0
3119	0,74969745	NA	-0,00245756	3,91E-05	-0,00181949	NA	-0,20513804	0,72537252	NA	NA	NA	NA	0,45346103	-0,54414773	9	-20736,6523	41491,3146	3654,8979	0
662	-1,1987332	0,68175498	NA	4,69E-05	NA	0,40581671	NA	-3,52383686	NA	0,05863003	NA	-0,00043937	NA	NA	8	-20737,7666	41491,5413	3655,12454	0
2174	-0,06671637	0,57575795	NA	4,34E-05	-0,00071955	0,41051829	-0,17478186	0,6960215	-0,05271384	NA	NA	NA	NA	-0,49283909	10	-20735,8116	41491,6353	3655,21859	0
2110	-0,06671637	0,57575795	NA	4,34E-05	-0,00071955	0,41051829	-0,17478186	0,6894459	NA	NA	NA	NA	NA	-0,49566768	9	-20736,8362	41491,6824	3655,26566	0
2176	-0,47166397	0,58945108	0,00876766	4,25E-05	-0,00073797	0,4135805	-0,1717464	0,6960215	-0,05271384	NA	NA	NA	NA	-0,49283909	11	-20734,8374	41491,6894	3655,27265	0
664	-1,59179834	0,69737855	0,00852736	4,60E-05	NA	0,40932129	NA	-3,52383686	NA	0,05863003	NA	-0,00043937	NA	NA	9	-20736,8502	41491,7104	3655,29369	0
2112	-0,47166397	0,58945108	0,00876766	4,25E-05	-0,00073797	0,4135805	-0,1717464	0,6894459	NA	NA	NA	NA	NA	-0,49566768	10	-20735,862	41491,7362	3655,3195	0
726	-1,1987332	0,68175498	NA	4,69E-05	NA	0,40581671	NA	-3,4425274	-0,05282396	0,05811259	NA	-0,00050954	NA	NA	9	-20736,9289	41491,8678	3655,45111	0
728	-1,59179834	0,69737855	0,00852736	4,60E-05	NA	0,40932129	NA	-3,4425274	-0,05282396	0,05811259	NA	-0,00050954	NA	NA	10	-20736,0125	41492,0372	3655,62048	0
2582	-1,1987332	0,68175498	NA	4,69E-05	NA	0,40581671	NA	0,98389174	NA	NA	NA	-0,00044751	NA	-0,50601759	8	-20738,948	41493,904	3657,48725	0
2584	-1,59179834	0,69737855	0,00852736	4,60E-05	NA	0,40932129	NA	0,98389174	NA	NA	NA	-0,00044751	NA	-0,50601759	9	-20738,0316	41494,0731	3657,6564	0
3093	-1,36352414	NA	NA	4,29E-05	NA	0,54467715	NA	0,72537252	NA	NA	NA	NA	0,45346103	-0,54414773	7	-20740,5265	41495,0592	3658,64249	0
3820	-9,35928532	0,43489058	0,04364193	NA	-0,00182965	NA	-0,59565606	-1,61532922	-0,10289105	0,0570521	NA	-0,00065333	0,49801214	-0,5357095	12	-20735,9916	41496,0005	3659,58382	0
3095	-1,08960867	NA	-0,00576757	4,36E-05	NA	0,53973144	NA	0,72537252	NA	NA	NA	NA	0,45346103	-0,54414773	8	-20740,0942	41496,1964	3659,77968	0
3149	-0,28011525	NA	NA	4,24E-05	-0,0016491	NA	NA	0,7406183	-0,11449835	NA	NA	NA	0,46966671	-0,53963709	8	-20740,3053	41496,6186	3660,20184	0
3597	-0,28011525	NA	NA	4,24E-05	-0,0016491	NA	NA	0,95852238	NA	NA	NA	-0,00035257	0,44577019	-0,55202249	8	-20740,5042	41497,0163	3660,59955	0
182	-0,67953035	0,68234123	NA	4,46E-05	NA	0,41541375	-0,13795142	-3,73836005	NA	0,05777952	NA	NA	NA	NA	8	-20740,5454	41497,0988	3660,68207	0
184	-1,04079715	0,69637088	0,00755047	4,39E-05	NA	0,41816409	-0,13461471	-3,73836005	NA	0,05777952	NA	NA	NA	NA	9	-20739,8262	41497,6623	3661,24557	0
3151	-0,2131598	NA	-0,0015413	4,25E-05	-0,00164139	NA	NA	0,7406183	-0,11449835	NA	NA	NA	0,46966671	-0,53963709	9	-20740,2756	41498,5612	3662,14445	0
3599	-0,2131598	NA	-0,0015413	4,25E-05	-0,00164139	NA	NA	0,95852238	NA	NA	NA	-0,00035257	0,44577019	-0,55202249	9	-20740,4745	41498,9589	3662,54215	0
246	-0,67953035	0,68234123	NA	4,46E-05	NA	0,41541375	-0,13795142	-3,74999095	0,01316736	0,05794033	NA	NA	NA	NA	9	-20740,4829	41498,9758	3662,55906	0
160	-1,28042467	0,61751101	0,00965662	4,55E-05	-0,00054807	0,40424516	NA	-3,73836005	NA	0,05777952	NA	NA	NA	NA	9	-20740,6308	41499,2716	3662,8549	0
248	-1,04079715	0,69637088	0,00755047	4,39E-05	NA	0,41816409	-0,13461471	-3,74999095	0,01316736	0,05794033	NA	NA	NA	NA	10	-20739,7637	41499,5395	3663,12278	0
158	-0,85074914	0,60334436	NA	4,65E-05	-0,00052401	0,40049867	NA	-3,73836005	NA	0,05777952	NA	NA	NA	NA	8	-20741,8067	41499,6213	3663,20458	0
2166	-0,67953035	0,68234123	NA	4,46E-05	NA	0,41541375	-0,13795142	0,6960215	-0,05271384	NA	NA	NA	NA	-0,49283909	9	-20740,9982	41500,0063	3663,5896	0
2102	-0,67953035	0,68234123	NA	4,46E-05	NA	0,41541375	-0,13795142	0,6894459	NA	NA	NA	NA	NA	-0,49566768	8	-20742,0228	41500,0536	3663,6369	0
3756	-9,35928532	0,43489058	0,04364193	NA	-0,00182965	NA	-0,59565606	-1,76635397	NA	0,05800383	NA	-0,00051675	0,48731517	-0,53620329	11	-20739,0358	41500,0862	3663,66947	0
2168	-1,04079715	0,69637088	0,00755047	4,39E-05	NA	0,41816409	-0,13461471	0,6960215	-0,05271384	NA	NA	NA	NA	-0,49283909	10	-20740,2789	41500,57	3664,15332	0
2104	-1,04079715	0,69637088	0,00755047	4,39E-05	NA	0,41816409	-0,13461471	0,6894459	NA	NA	NA	NA	NA	-0,49566768	9	-20741,3036	41500,6171	3664,2004	0
224	-1,28042467	0,61751101	0,00965662	4,55E-05	-0,00054807	0,40424516	NA	-3,74999095	0,01316736	0,05794033	NA	NA	NA	NA	10	-20740,5683	41501,1488	3664,73211	0
222	-0,85074914	0,60334436	NA	4,65E-05	-0,00052401	0,40049867	NA	-3,74999095	0,01316736	0,05794033	NA	NA	NA	NA	9	-20741,7442	41501,4983	3665,08157	0
3259	-8,35316771	NA	0,03864408	NA	-0,00234345	0,43617682	-0,64441699	-2,06058113	NA	0,05699765	NA	NA	0,49693597	-0,52441365	10	-20741,0829	41502,1779	3665,76117	0
2144	-1,28042467	0,61751101	0,00965662	4,55E-05	-0,00054807	0,40424516	NA	0,6960215	-0,05271384	NA	NA	NA	NA	-0,49283909	10	-20741,0836	41502,1794	3665,76265	0
2080	-1,28042467	0,61751101	0,00965662	4,55E-05	-0,00054807	0,40424516	NA	0,6894459	NA	NA	NA	NA	NA	-0,49566768	9	-20742,1082	41502,2265	3665,80973	0

3834	-6,98837994	0,26427623	NA	NA	-0,00180413	0,37036432	-0,69230587	-1,61532922	-0,10289105	0,0570521	NA	-0,00065333	0,49801214	-0,5357095	12	-20739,2122	41502,4417	3666,025	0
2142	-0,85074914	0,60334436	NA	4,65E-05	-0,00052401	0,40049867	NA	0,6960215	-0,05271384	NA	NA	NA	NA	-0,49283909	9	-20742,2594	41502,5288	3666,11211	0
2078	-0,85074914	0,60334436	NA	4,65E-05	-0,00052401	0,40049867	NA	0,6894459	NA	NA	NA	NA	NA	-0,49566768	8	-20743,2841	41502,5761	3666,15941	0
150	-1,1987332	0,68175498	NA	4,69E-05	NA	0,40581671	NA	-3,73836005	NA	0,05777952	NA	NA	NA	NA	7	-20744,7205	41503,4473	3667,03057	0
152	-1,59179834	0,69737855	0,00852736	4,60E-05	NA	0,40932129	NA	-3,73836005	NA	0,05777952	NA	NA	NA	NA	8	-20743,8041	41503,6162	3667,19949	0
3323	-8,35316771	NA	0,03864408	NA	-0,00234345	0,43617682	-0,64441699	-2,04753199	-0,01846226	0,05678211	NA	NA	0,49927467	-0,52377486	11	-20740,9652	41503,9449	3667,52818	0
3085	-0,28011525	NA	NA	4,24E-05	-0,0016491	NA	NA	0,72537252	NA	NA	NA	NA	0,45346103	-0,54414773	7	-20745,0182	41504,0426	3667,62591	0
214	-1,1987332	0,68175498	NA	4,69E-05	NA	0,40581671	NA	-3,74999095	0,01316736	0,05794033	NA	NA	NA	NA	8	-20744,658	41505,3241	3668,90734	0
216	-1,59179834	0,69737855	0,00852736	4,60E-05	NA	0,40932129	NA	-3,74999095	0,01316736	0,05794033	NA	NA	NA	NA	9	-20743,7416	41505,4932	3669,07649	0
3087	-0,2131598	NA	-0,0015413	4,25E-05	-0,00164139	NA	NA	0,72537252	NA	NA	NA	NA	0,45346103	-0,54414773	8	-20744,9885	41505,985	3669,56829	0
2134	-1,1987332	0,68175498	NA	4,69E-05	NA	0,40581671	NA	0,6960215	-0,05271384	NA	NA	NA	NA	-0,49283909	8	-20745,1733	41506,3546	3669,93788	0
2070	-1,1987332	0,68175498	NA	4,69E-05	NA	0,40581671	NA	0,6894459	NA	NA	NA	NA	NA	-0,49566768	7	-20746,198	41506,4021	3669,9854	0
2136	-1,59179834	0,69737855	0,00852736	4,60E-05	NA	0,40932129	NA	0,6960215	-0,05271384	NA	NA	NA	NA	-0,49283909	9	-20744,2569	41506,5237	3670,10703	0
3770	-6,98837994	0,26427623	NA	NA	-0,00180413	0,37036432	-0,69230587	-1,76635397	NA	0,05800383	NA	-0,00051675	0,48731517	-0,53620329	11	-20742,2564	41506,5274	3670,11065	0
2072	-1,59179834	0,69737855	0,00852736	4,60E-05	NA	0,40932129	NA	0,6894459	NA	NA	NA	NA	NA	-0,49566768	8	-20745,2815	41506,571	3670,15432	0
3828	-10,737769	0,62231984	0,04626548	NA	NA	0,40997724	-0,57836783	-1,61532922	-0,10289105	0,0570521	NA	-0,00065333	0,49801214	-0,5357095	12	-20742,2196	41508,4564	3672,03969	0
1773	0,64576877	NA	NA	3,88E-05	-0,00183092	NA	-0,20438761	-3,6371906	-0,09814646	0,05972263	NA	-0,00047389	0,39127251	NA	10	-20745,4553	41510,9228	3674,50612	0
1781	-0,84339357	NA	NA	4,07E-05	NA	0,55505772	-0,13640315	-3,6371906	-0,09814646	0,05972263	NA	-0,00047389	0,39127251	NA	10	-20745,7136	41511,4393	3675,02255	0
1783	-0,50593593	NA	-0,00700816	4,14E-05	NA	0,55021643	-0,14061875	-3,6371906	-0,09814646	0,05972263	NA	-0,00047389	0,39127251	NA	11	-20745,0805	41512,1756	3675,75886	0
3764	-10,737769	0,62231984	0,04626548	NA	NA	0,40997724	-0,57836783	-1,76635397	NA	0,05800383	NA	-0,00051675	0,48731517	-0,53620329	11	-20745,2637	41512,5421	3676,12534	0
1775	0,74969745	NA	-0,00245756	3,91E-05	-0,00181949	NA	-0,20513804	-3,6371906	-0,09814646	0,05972263	NA	-0,00047389	0,39127251	NA	11	-20745,3798	41512,7741	3676,3574	0
3833	-5,46460783	NA	NA	NA	-0,00220571	0,40342229	-0,70298126	-1,61532922	-0,10289105	0,0570521	NA	-0,00065333	0,49801214	-0,5357095	11	-20745,5183	41513,0512	3676,6345	0
1709	0,64576877	NA	NA	3,88E-05	-0,00183092	NA	-0,20438761	-3,78227707	NA	0,0606519	NA	-0,00034645	0,38158141	NA	9	-20748,2916	41514,5931	3678,17639	0
1717	-0,84339357	NA	NA	4,07E-05	NA	0,55505772	-0,13640315	-3,78227707	NA	0,0606519	NA	-0,00034645	0,38158141	NA	9	-20748,5498	41515,1095	3678,69282	0
1719	-0,50593593	NA	-0,00700816	4,14E-05	NA	0,55021643	-0,14061875	-3,78227707	NA	0,0606519	NA	-0,00034645	0,38158141	NA	10	-20747,9167	41515,8456	3679,42891	0
1711	0,74969745	NA	-0,00245756	3,91E-05	-0,00181949	NA	-0,20513804	-3,78227707	NA	0,0606519	NA	-0,00034645	0,38158141	NA	10	-20748,216	41516,4442	3680,02745	0
1749	-1,36352414	NA	NA	4,29E-05	NA	0,54467715	NA	-3,6371906	-0,09814646	0,05972263	NA	-0,00047389	0,39127251	NA	9	-20749,2539	41516,5178	3680,1011	0
3244	-9,35928532	0,43489058	0,04364193	NA	-0,00182965	NA	-0,59565606	-2,06058113	NA	0,05699765	NA	NA	0,49693597	-0,52441365	10	-20748,2805	41516,5732	3680,15647	0
3769	-5,46460783	NA	NA	NA	-0,00220571	0,40342229	-0,70298126	-1,76635397	NA	0,05800383	NA	-0,00051675	0,48731517	-0,53620329	10	-20748,5625	41517,1371	3680,72037	0
1751	-1,08960867	NA	-0,00576757	4,36E-05	NA	0,53973144	NA	-3,6371906	-0,09814646	0,05972263	NA	-0,00047389	0,39127251	NA	10	-20748,8216	41517,6555	3681,23873	0
3308	-9,35928532	0,43489058	0,04364193	NA	-0,00182965	NA	-0,59565606	-2,04753199	-0,01846226	0,05678211	NA	NA	0,49927467	-0,52377486	11	-20748,1628	41518,3402	3681,92349	0
2670	-0,14195779	0,65649184	NA	4,36E-05	-0,00074998	NA	-0,15445798	1,12236839	-0,1349839	NA	NA	-0,00063299	NA	-0,50295006	10	-20749,5618	41519,1358	3682,71909	0
2672	-0,45110821	0,66723944	0,00674107	4,29E-05	-0,00076432	NA	-0,15224001	1,12236839	-0,1349839	NA	NA	-0,00063299	NA	-0,50295006	11	-20749,0037	41520,0219	3683,6052	0
1685	-1,36352414	NA	NA	4,29E-05	NA	0,54467715	NA	-3,78227707	NA	0,0606519	NA	-0,00034645	0,38158141	NA	8	-20752,0902	41520,1883	3683,77159	0
1197	0,64576877	NA	NA	3,88E-05	-0,00183092	NA	-0,20438761	-3,95454473	NA	0,05999672	NA	NA	0,38996522	NA	8	-20752,6347	41521,2774	3684,86069	0
1687	-1,08960867	NA	-0,00576757	4,36E-05	NA	0,53973144	NA	-3,78227707	NA	0,0606519	NA	-0,00034645	0,38158141	NA	9	-20751,6579	41521,3257	3684,909	0
1205	-0,84339357	NA	NA	4,07E-05	NA	0,55505772	-0,13640315	-3,95454473	NA	0,05999672	NA	NA	0,38996522	NA	8	-20752,8929	41521,7938	3685,37713	0
1261	0,64576877	NA	NA	3,88E-05	-0,00183092	NA	-0,20438761	-3,92430325	-0,03654601	0,05956532	NA	NA	0,39466847	NA	9	-20752,1636	41522,3371	3685,92041	0
1207	-0,50593593	NA	-0,00700816	4,14E-05	NA	0,55021643	-0,14061875	-3,95454473	NA	0,05999672	NA	NA	0,38996522	NA	9	-20752,2599	41522,5297	3686,113	0
1269	-0,84339357	NA	NA	4,07E-05	NA	0,55505772	-0,13640315	-3,92430325	-0,03654601	0,05956532	NA	NA	0,39466847	NA	9	-20752,4218	41522,8536	3686,43684	0
3258	-6,98837994	0,26427623	NA	NA	-0,00180413	0,37036432	-0,69230587	-2,06058113	NA	0,05699765	NA	NA	0,49693597	-0,52441365	10	-20751,5011	41523,0144	3686,59765	0
1199	0,74969745	NA	-0,00245756	3,91E-05	-0,00181949	NA	-0,20513804	-3,95454473	NA	0,05999672	NA	NA	0,38996522	NA	9	-20752,5592	41523,1283	3686,71153	0
1271	-0,50593593	NA	-0,00700816	4,14E-05	NA	0,55021643	-0,14061875	-3,92430325	-0,03654601	0,05956532	NA	NA	0,39466847	NA	10	-20751,7887	41523,5897	3687,17293	0
1263	0,74969745	NA	-0,00245756	3,91E-05	-0,00181949	NA	-0,20513804	-3,92430325	-0,03654601	0,05956532	NA	NA	0,39466847	NA	10	-20752,088	41524,1882	3687,77147	0
3322	-6,98837994	0,26427623	NA	NA	-0,00180413	0,37036432	-0,69230587	-2,04753199	-0,01846226	0,05678211	NA	NA	0,49927467	-0,52377486	11	-20751,3834	41524,7814	3688,36467	0
1741	-0,28011525	NA	NA	4,24E-05	-0,0016491	NA	NA	-3,6371906	-0,09814646	0,05972263	NA	-0,00047389	0,39127251	NA	9	-20753,7456	41525,5012	3689,08452	0
686	-0,14195779	0,65649184	NA	4,36E-05	-0,00074998	NA	-0,15445798	-3,52383686	NA	0,05863003	NA	-0,00043937	NA	NA	9	-20753,9341	41525,8781	3689,46137	0
750	-0,14195779	0,65649184	NA	4,36E-05	-0,00074998	NA	-0,15445798	-3,4425274	-0,05282396	0,05811259	NA	-0,00050954	NA	NA	10	-20753,0964	41526,2049	3689,78816	0
3819	-5,75020926	NA	0,0346068	NA	-0,00247017	NA	-0,61951905	-1,61532922	-0,10289105	0,0570521	NA	-0,00065333	0,49801214	-0,5357095	11	-20752,3449	41526,7045	3690,28774	0
688	-0,45110821	0,66723944	0,00674107	4,29E-05	-0,00076432	NA	-0,15224001	-3,52383686	NA	0,05863003	NA	-0,00043937	NA	NA	10	-20753,3759	41526,764	3690,34727	0
1173	-1,36352414	NA	NA	4,29E-05	NA	0,54467715	NA	-3,95454473	NA	0,05999672	NA	NA	0,38996522	NA	7	-20756,4333	41526,8728	3690,45612	0
752	-0,45110821	0,66723944	0,00674107	4,29E-05	-0,00076432	NA	-0,15224001	-3,4425274	-0,05282396	0,05811259	NA	-0,00050954	NA	NA	11	-20752,5382	41527,091	3690,67428	0
2638	-0,8455927	0,67951403	NA	4,64E-05	-0,00057321	NA	NA	1,12236839	-0,1349839	NA	NA	-0,00063299	NA	-0,50295006	9	-20754,5823	41527,1745	3690,75782	0
1743	-0,2131598	NA	-0,0015413	4,25E-05	-0,00164139	NA	NA	-3,6371906	-0,09814646	0,05972263	NA	-0,00047389	0,39127251	NA	10	-20753,7159	41527,4441	3691,02734	0
2640	-1,17938783	0,69098467	0,00751019	4,56E-05	-0,00059207	NA	NA	1,12236839	-0,1349839	NA	NA	-0,00063299	NA	-0,50295006	10	-20753,8923	41527,7969	3691,38013	0
1237	-1,36352414	NA	NA	4,29E-05	NA	0,54467715	NA	-3,92430325	-0,03654601	0,05956532	NA	NA	0,39466847	NA	8	-20755,9622	41527,9323	3691,51561	0
1175	-1,08960867	NA	-0,00576757	4,36E-05	NA	0,53973144	NA	-3,95454473	NA	0,05999672	NA	NA	0,38996522	NA	8	-20756,001	41528,01	3691,59331	0
2606	-0,14195779	0,65649184	NA	4,36E-05	-0,00074998	NA	-0,15445798	0,98389174	NA	NA	NA	-0,00044751	NA	-0,50601759	9	-20755,1154	41528,2408	3691,82408	0
2662	-0,79586264	0,77122371	NA	4,50E-05	NA	NA	-0,11503973	1,12236839	-0,1349839	NA	NA	-0,00063299	NA	-0,50295006	9	-20755,156	41528,322	3691,90525	0
3252	-10,737769	0,62231984	0,04626548	NA	NA	0,40997724	-0,57836783	-2,06058113	NA	0,05699765	NA	NA	0,49693597	-0,52441365	10	-20754,5084	41529,0291	3692,61235	0
1239	-1,08960867	NA	-0,00576757	4,36E-05	NA	0,53973144	NA	-3,92430325	-0,03654601	0,05956532	NA	NA	0,39466847	NA	9	-20755,5299	41529,0697	3692,65302	0

2608	-0,45110821	0,66723944	0,00674107	4,29E-05	-0,00076432	NA	-0,15224001	0,98389174	NA	NA	NA	-0,00044751	NA	-0,50601759	10	-20754,5573	41529,1267	3692,70998	0
1677	-0,28011525	NA	NA	4,24E-05	-0,0016491	NA	NA	-3,78227707	NA	0,0606519	NA	-0,00034645	0,38158141	NA	8	-20756,5819	41529,1717	3692,75501	0
2664	-1,05409025	0,78157333	0,00543311	4,44E-05	NA	NA	-0,11273577	1,12236839	-0,1349839	NA	NA	-0,00063299	NA	-0,50295006	10	-20754,7957	41529,6036	3693,18689	0
3755	-5,75020926	NA	0,0346068	NA	-0,00247017	NA	-0,61951905	-1,76635397	NA	0,05800383	NA	-0,00051675	0,48731517	-0,53620329	10	-20755,3891	41530,7903	3694,37362	0
3316	-10,737769	0,62231984	0,04626548	NA	NA	0,40997724	-0,57836783	-2,04753199	-0,01846226	0,05678211	NA	NA	0,49927467	-0,52377486	11	-20754,3907	41530,7961	3694,37936	0
1679	-0,2131598	NA	-0,0015413	4,25E-05	-0,00164139	NA	NA	-3,78227707	NA	0,0606519	NA	-0,00034645	0,38158141	NA	9	-20756,5522	41531,1143	3694,69761	0
2630	-1,235734	0,76857786	NA	4,69E-05	NA	NA	NA	1,12236839	-0,1349839	NA	NA	-0,00063299	NA	-0,50295006	8	-20758,0547	41532,1173	3695,70054	0
2632	-1,52051301	0,78035551	0,00624724	4,63E-05	NA	NA	NA	1,12236839	-0,1349839	NA	NA	-0,00063299	NA	-0,50295006	9	-20757,5786	41533,1671	3696,75035	0
3257	-5,46460783	NA	NA	NA	-0,00220571	0,40342229	-0,70298126	-2,06058113	NA	0,05699765	NA	NA	0,49693597	-0,52441365	9	-20757,8072	41533,6243	3697,2076	0
3818	-6,22256645	0,32760888	NA	NA	-0,00183217	NA	-0,66370241	-1,61532922	-0,10289105	0,0570521	NA	-0,00065333	0,49801214	-0,5357095	11	-20755,8935	41533,8015	3697,3848	0
654	-0,8455927	0,67951403	NA	4,64E-05	-0,00057321	NA	NA	-3,52383686	NA	0,05863003	NA	-0,00043937	NA	NA	8	-20758,9545	41533,9171	3697,50033	0
718	-0,8455927	0,67951403	NA	4,64E-05	-0,00057321	NA	NA	-3,4425274	-0,05282396	0,05811259	NA	-0,00050954	NA	NA	9	-20758,1168	41534,2436	3697,8269	0
656	-1,17938783	0,69098467	0,00751019	4,56E-05	-0,00059207	NA	NA	-3,52383686	NA	0,05863003	NA	-0,00043937	NA	NA	9	-20758,2646	41534,5391	3698,12242	0
720	-1,17938783	0,69098467	0,00751019	4,56E-05	-0,00059207	NA	NA	-3,4425274	-0,05282396	0,05811259	NA	-0,00050954	NA	NA	10	-20757,4269	41534,8659	3698,44921	0
678	-0,79586264	0,77122371	NA	4,50E-05	NA	NA	-0,11503973	-3,52383686	NA	0,05863003	NA	-0,00043937	NA	NA	8	-20759,5283	41535,0645	3698,64776	0
742	-0,79586264	0,77122371	NA	4,50E-05	NA	NA	-0,11503973	-3,4425274	-0,05282396	0,05811259	NA	-0,00050954	NA	NA	9	-20758,6905	41535,391	3698,97433	0
3321	-5,46460783	NA	NA	NA	-0,00220571	0,40342229	-0,70298126	-2,04753199	-0,01846226	0,05678211	NA	NA	0,49927467	-0,52377486	10	-20757,6895	41535,3911	3698,97439	0
1165	-0,28011525	NA	NA	4,24E-05	-0,0016491	NA	NA	-3,95454473	NA	0,05999672	NA	NA	0,38996522	NA	7	-20760,925	41535,8563	3699,43954	0
2574	-0,8455927	0,67951403	NA	4,64E-05	-0,00057321	NA	NA	0,98389174	NA	NA	NA	-0,00044751	NA	-0,50601759	8	-20760,1359	41536,2798	3699,86304	0
680	-1,05409025	0,78157333	0,00543311	4,44E-05	NA	NA	-0,11273577	-3,52383686	NA	0,05863003	NA	-0,00043937	NA	NA	9	-20759,168	41536,3459	3699,92917	0
744	-1,05409025	0,78157333	0,00543311	4,44E-05	NA	NA	-0,11273577	-3,4425274	-0,05282396	0,05811259	NA	-0,00050954	NA	NA	10	-20758,3303	41536,6727	3700,25596	0
2576	-1,17938783	0,69098467	0,00751019	4,56E-05	-0,00059207	NA	NA	0,98389174	NA	NA	NA	-0,00044751	NA	-0,50601759	9	-20759,4459	41536,9019	3700,48513	0
1229	-0,28011525	NA	NA	4,24E-05	-0,0016491	NA	NA	-3,92430325	-0,03654601	0,05956532	NA	NA	0,39466847	NA	8	-20760,4539	41536,9157	3700,49903	0
2598	-0,79586264	0,77122371	NA	4,50E-05	NA	NA	-0,11503973	0,98389174	NA	NA	NA	-0,00044751	NA	-0,50601759	8	-20760,7096	41537,4272	3701,01047	0
174	-0,14195779	0,65649184	NA	4,36E-05	-0,00074998	NA	-0,15445798	-3,73836005	NA	0,05777952	NA	NA	NA	NA	8	-20760,888	41537,7839	3701,36718	0
1167	-0,2131598	NA	-0,0015413	4,25E-05	-0,00164139	NA	NA	-3,95454473	NA	0,05999672	NA	NA	0,38996522	NA	8	-20760,8953	41537,7986	3701,38192	0
3754	-6,22256645	0,32760888	NA	NA	-0,00183217	NA	-0,66370241	-1,76635397	NA	0,05800383	NA	-0,00051675	0,48731517	-0,53620329	10	-20758,9376	41537,8874	3701,47068	0
176	-0,45110821	0,66723944	0,00674107	4,29E-05	-0,00076432	NA	-0,15224001	-3,73836005	NA	0,05777952	NA	NA	NA	NA	9	-20760,3298	41538,6696	3702,25285	0
2600	-1,05409025	0,78157333	0,00543311	4,44E-05	NA	NA	-0,11273577	0,98389174	NA	NA	NA	-0,00044751	NA	-0,50601759	9	-20760,3493	41538,7086	3702,29188	0
1231	-0,2131598	NA	-0,0015413	4,25E-05	-0,00164139	NA	NA	-3,92430325	-0,03654601	0,05956532	NA	NA	0,39466847	NA	9	-20760,4242	41538,8584	3702,44163	0
646	-1,235734	0,76857786	NA	4,69E-05	NA	NA	NA	-3,52383686	NA	0,05863003	NA	-0,00043937	NA	NA	7	-20762,4269	41538,86	3702,44327	0
710	-1,235734	0,76857786	NA	4,69E-05	NA	NA	NA	-3,4425274	-0,05282396	0,05811259	NA	-0,00050954	NA	NA	8	-20761,5892	41539,1863	3702,76962	0
238	-0,14195779	0,65649184	NA	4,36E-05	-0,00074998	NA	-0,15445798	-3,74999095	0,01316736	0,05794033	NA	NA	NA	NA	9	-20760,8255	41539,6609	3703,24417	0
2685	0,65451489	NA	NA	3,95E-05	-0,00164357	0,48762971	-0,22170047	1,12236839	-0,1349839	NA	NA	-0,00063299	NA	-0,50295006	10	-20759,8599	41539,732	3703,31528	0
648	-1,52051301	0,78035551	0,00624724	4,63E-05	NA	NA	NA	-3,52383686	NA	0,05863003	NA	-0,00043937	NA	NA	8	-20761,9508	41539,9096	3703,49286	0
712	-1,52051301	0,78035551	0,00624724	4,63E-05	NA	NA	NA	-3,4425274	-0,05282396	0,05811259	NA	-0,00050954	NA	NA	9	-20761,1131	41540,2361	3703,81943	0
240	-0,45110821	0,66723944	0,00674107	4,29E-05	-0,00076432	NA	-0,15224001	-3,74999095	0,01316736	0,05794033	NA	NA	NA	NA	10	-20760,2673	41540,5468	3704,13006	0
2158	-0,14195779	0,65649184	NA	4,36E-05	-0,00074998	NA	-0,15445798	0,6960215	-0,05271384	NA	NA	NA	NA	-0,49283909	9	-20761,3407	41540,6914	3704,27471	0
2094	-0,14195779	0,65649184	NA	4,36E-05	-0,00074998	NA	-0,15445798	0,6894459	NA	NA	NA	NA	NA	-0,49566768	8	-20762,3654	41540,7387	3704,32201	0
2566	-1,235734	0,76857786	NA	4,69E-05	NA	NA	NA	0,98389174	NA	NA	NA	-0,00044751	NA	-0,50601759	7	-20763,6083	41541,2227	3704,80598	0
2160	-0,45110821	0,66723944	0,00674107	4,29E-05	-0,00076432	NA	-0,15224001	0,6960215	-0,05271384	NA	NA	NA	NA	-0,49283909	10	-20760,7826	41541,5773	3705,1606	0
2096	-0,45110821	0,66723944	0,00674107	4,29E-05	-0,00076432	NA	-0,15224001	0,6894459	NA	NA	NA	NA	NA	-0,49566768	9	-20761,8072	41541,6244	3705,20768	0
2687	0,61699723	NA	0,00084798	3,94E-05	-0,00164748	0,4880593	-0,22142621	1,12236839	-0,1349839	NA	NA	-0,00063299	NA	-0,50295006	11	-20759,8506	41541,7158	3705,29912	0
2568	-1,52051301	0,78035551	0,00624724	4,63E-05	NA	NA	NA	0,98389174	NA	NA	NA	-0,00044751	NA	-0,50601759	8	-20763,1322	41542,2723	3705,85557	0
142	-0,8455927	0,67951403	NA	4,64E-05	-0,00057321	NA	NA	-3,73836005	NA	0,05777952	NA	NA	NA	NA	7	-20765,9084	41545,8231	3709,40636	0
144	-1,17938783	0,69098467	0,00751019	4,56E-05	-0,00059207	NA	NA	-3,73836005	NA	0,05777952	NA	NA	NA	NA	8	-20765,2185	41546,4449	3710,02823	0
701	0,65451489	NA	NA	3,95E-05	-0,00164357	0,48762971	-0,22170047	-3,52383686	NA	0,05863003	NA	-0,00043937	NA	NA	9	-20764,2322	41546,4743	3710,05756	0
765	0,65451489	NA	NA	3,95E-05	-0,00164357	0,48762971	-0,22170047	-3,4425274	-0,05282396	0,05811259	NA	-0,00050954	NA	NA	10	-20763,3945	41546,8011	3710,38435	0
166	-0,79586264	0,77122371	NA	4,50E-05	NA	NA	-0,11503973	-3,73836005	NA	0,05777952	NA	NA	NA	NA	7	-20766,4822	41546,9705	3710,55378	0
3243	-5,75020926	NA	0,0346068	NA	-0,00247017	NA	-0,61951905	-2,06058113	NA	0,05699765	NA	NA	0,49693597	-0,52441365	9	-20764,6338	41547,2776	3710,86084	0
3812	-9,79595589	0,69445228	0,04389505	NA	NA	NA	-0,55018722	-1,61532922	-0,10289105	0,0570521	NA	-0,00065333	0,49801214	-0,5357095	11	-20762,6321	41547,2788	3710,86209	0
206	-0,8455927	0,67951403	NA	4,64E-05	-0,00057321	NA	NA	-3,74999095	0,01316736	0,05794033	NA	NA	NA	NA	8	-20765,8459	41547,6998	3711,28313	0
168	-1,05409025	0,78157333	0,00543311	4,44E-05	NA	NA	-0,11273577	-3,73836005	NA	0,05777952	NA	NA	NA	NA	8	-20766,1219	41548,2517	3711,83498	0
208	-1,17938783	0,69098467	0,00751019	4,56E-05	-0,00059207	NA	NA	-3,74999095	0,01316736	0,05794033	NA	NA	NA	NA	9	-20765,156	41548,3219	3711,90522	0
703	0,61699723	NA	0,00084798	3,94E-05	-0,00164748	0,4880593	-0,22142621	-3,52383686	NA	0,05863003	NA	-0,00043937	NA	NA	10	-20764,2229	41548,4579	3712,04119	0
2126	-0,8455927	0,67951403	NA	4,64E-05	-0,00057321	NA	NA	0,6960215	-0,05271384	NA	NA	NA	NA	-0,49283909	8	-20766,3612	41548,7304	3712,31367	0
2062	-0,8455927	0,67951403	NA	4,64E-05	-0,00057321	NA	NA	0,6894459	NA	NA	NA	NA	NA	-0,49566768	7	-20767,3859	41548,7779	3712,36118	0
767	0,61699723	NA	0,00084798	3,94E-05	-0,00164748	0,4880593	-0,22142621	-3,4425274	-0,05282396	0,05811259	NA	-0,00050954	NA	NA	11	-20763,3852	41548,7849	3712,3682	0
2621	0,65451489	NA	NA	3,95E-05	-0,00164357	0,48762971	-0,22170047	0,98389174	NA	NA	NA	-0,00044751	NA	-0,50601759	9	-20765,4135	41548,837	3712,42027	0
230	-0,79586264	0,77122371	NA	4,50E-05	NA	NA	-0,11503973	-3,74999095	0,01316736	0,05794033	NA	NA	NA	NA	8	-20766,4197	41548,8473	3712,43055	0
3687	-0,65771633	NA	-0,01187559	4,14E-05	NA	NA	-0,10732692	1,15818376	-0,19528017	NA	NA	-0,00061584	0,46809624	-0,5501339	10	-20764,4209	41548,854	3712,4373	0

3307	-5,75020926	NA	0,0346068	NA	-0,00247017	NA	-0,61951905	-2,04753199	-0,01846226	0,05678211	NA	NA	0,49927467	-0,52377486	10	-20764,5161	41549,0444	3712,62763	0
2128	-1,17938783	0,69098467	0,00751019	4,56E-05	-0,00059207	NA	NA	0,6960215	-0,05271384	NA	NA	NA	NA	-0,49283909	9	-20765,6713	41549,3525	3712,93576	0
2064	-1,17938783	0,69098467	0,00751019	4,56E-05	-0,00059207	NA	NA	0,6894459	NA	NA	NA	NA	NA	-0,49566768	8	-20766,6959	41549,3998	3712,98305	0
2150	-0,79586264	0,77122371	NA	4,50E-05	NA	NA	-0,11503973	0,6960215	-0,05271384	NA	NA	NA	NA	-0,49283909	8	-20766,9349	41549,8778	3713,4611	0
2086	-0,79586264	0,77122371	NA	4,50E-05	NA	NA	-0,11503973	0,6894459	NA	NA	NA	NA	NA	-0,49566768	7	-20767,9596	41549,9253	3713,50861	0
232	-1,05409025	0,78157333	0,00543311	4,44E-05	NA	NA	-0,11273577	-3,74999095	0,01316736	0,05794033	NA	NA	NA	NA	9	-20766,0594	41550,1287	3713,71197	0
3685	-1,22822825	NA	NA	4,02E-05	NA	NA	-0,09995451	1,15818376	-0,19528017	NA	NA	-0,00061584	0,46809624	-0,5501339	9	-20766,1654	41550,3407	3713,92398	0
134	-1,235734	0,76857786	NA	4,69E-05	NA	NA	NA	-3,73836005	NA	0,05777952	NA	NA	NA	NA	6	-20769,3808	41550,7662	3714,34952	0
2623	0,61699723	NA	0,00084798	3,94E-05	-0,00164748	0,4880593	-0,22142621	0,98389174	NA	NA	NA	-0,00044751	NA	-0,50601759	10	-20765,4042	41550,8206	3714,4039	0
3655	-1,14142692	NA	-0,01089562	4,31E-05	NA	NA	NA	1,15818376	-0,19528017	NA	NA	-0,00061584	0,46809624	-0,5501339	9	-20766,535	41551,08	3714,66329	0
2152	-1,05409025	0,78157333	0,00543311	4,44E-05	NA	NA	-0,11273577	0,6960215	-0,05271384	NA	NA	NA	NA	-0,49283909	9	-20766,5746	41551,1592	3714,74251	0
2088	-1,05409025	0,78157333	0,00543311	4,44E-05	NA	NA	-0,11273577	0,6894459	NA	NA	NA	NA	NA	-0,49566768	8	-20767,5993	41551,2065	3714,78981	0
3826	-8,51824578	0,51124721	NA	NA	NA	0,38535796	-0,65213053	-1,61532922	-0,10289105	0,0570521	NA	-0,00065333	0,49801214	-0,5357095	11	-20764,6191	41551,2527	3714,836	0
3748	-9,79595589	0,69445228	0,04389505	NA	NA	NA	-0,55018722	-1,76635397	NA	0,05800383	NA	-0,00051675	0,48731517	-0,53620329	10	-20765,6763	41551,3647	3714,94797	0
3817	-3,98530292	NA	NA	NA	-0,00234183	NA	-0,67333606	-1,61532922	-0,10289105	0,0570521	NA	-0,00065333	0,49801214	-0,5357095	10	-20765,7646	41551,5413	3715,1246	0
136	-1,52051301	0,78035551	0,00624724	4,63E-05	NA	NA	NA	-3,73836005	NA	0,05777952	NA	NA	NA	NA	7	-20768,9047	41551,8156	3715,39889	0
3653	-1,63084607	NA	NA	4,18E-05	NA	NA	NA	1,15818376	-0,19528017	NA	NA	-0,00061584	0,46809624	-0,5501339	8	-20768,0102	41552,0284	3715,61166	0
198	-1,235734	0,76857786	NA	4,69E-05	NA	NA	NA	-3,74999095	0,01316736	0,05794033	NA	NA	NA	NA	7	-20769,3183	41552,6428	3716,22607	0
2118	-1,235734	0,76857786	NA	4,69E-05	NA	NA	NA	0,6960215	-0,05271384	NA	NA	NA	NA	-0,49283909	7	-20769,8336	41553,6733	3717,25661	0
200	-1,52051301	0,78035551	0,00624724	4,63E-05	NA	NA	NA	-3,74999095	0,01316736	0,05794033	NA	NA	NA	NA	8	-20768,8422	41553,6924	3717,27566	0
2054	-1,235734	0,76857786	NA	4,69E-05	NA	NA	NA	0,6894459	NA	NA	NA	NA	NA	-0,49566768	6	-20770,8582	41553,7211	3717,30435	0
3242	-6,22256645	0,32760888	NA	NA	-0,00183217	NA	-0,66370241	-2,06058113	NA	0,05699765	NA	NA	0,49693597	-0,52441365	9	-20768,1823	41554,3746	3717,9579	0
2120	-1,52051301	0,78035551	0,00624724	4,63E-05	NA	NA	NA	0,6960215	-0,05271384	NA	NA	NA	NA	-0,49283909	8	-20769,3575	41554,7229	3718,3062	0
2056	-1,52051301	0,78035551	0,00624724	4,63E-05	NA	NA	NA	0,6894459	NA	NA	NA	NA	NA	-0,49566768	7	-20770,3821	41554,7704	3718,35372	0
3762	-8,51824578	0,51124721	NA	NA	NA	0,38535796	-0,65213053	-1,76635397	NA	0,05800383	NA	-0,00051675	0,48731517	-0,53620329	10	-20767,6632	41555,3386	3718,92187	0
3753	-3,98530292	NA	NA	NA	-0,00234183	NA	-0,67333606	-1,76635397	NA	0,05800383	NA	-0,00051675	0,48731517	-0,53620329	9	-20768,8087	41555,6274	3719,2107	0
3306	-6,22256645	0,32760888	NA	NA	-0,00183217	NA	-0,66370241	-2,04753199	-0,01846226	0,05678211	NA	NA	0,49927467	-0,52377486	10	-20768,0646	41556,1414	3719,72469	0
2653	-0,31227401	NA	NA	4,32E-05	-0,00145314	0,47935513	NA	1,12236839	-0,1349839	NA	NA	-0,00063299	NA	-0,50295006	9	-20769,7628	41557,5355	3721,11876	0
189	0,65451489	NA	NA	3,95E-05	-0,00164357	0,48762971	-0,22170047	-3,73836005	NA	0,05777952	NA	NA	NA	NA	8	-20771,1861	41558,3801	3721,96337	0
2655	-0,39160679	NA	0,00183718	4,30E-05	-0,00146197	0,48040455	NA	1,12236839	-0,1349839	NA	NA	-0,00063299	NA	-0,50295006	10	-20769,7194	41559,4509	3723,03415	0
253	0,65451489	NA	NA	3,95E-05	-0,00164357	0,48762971	-0,22170047	-3,74999095	0,01316736	0,05794033	NA	NA	NA	NA	9	-20771,1236	41560,2571	3723,84036	0
191	0,61699723	NA	0,00084798	3,94E-05	-0,00164748	0,4880593	-0,22142621	-3,73836005	NA	0,05777952	NA	NA	NA	NA	9	-20771,1768	41560,3635	3723,94677	0
2173	0,65451489	NA	NA	3,95E-05	-0,00164357	0,48762971	-0,22170047	0,6960215	-0,05271384	NA	NA	NA	NA	-0,49283909	9	-20771,6388	41561,2876	3724,8709	0
2109	0,65451489	NA	NA	3,95E-05	-0,00164357	0,48762971	-0,22170047	0,6894459	NA	NA	NA	NA	NA	-0,49566768	8	-20772,6635	41561,3349	3724,9182	0
255	0,61699723	NA	0,00084798	3,94E-05	-0,00164748	0,4880593	-0,22142621	-3,74999095	0,01316736	0,05794033	NA	NA	NA	NA	10	-20771,1143	41562,2407	3725,82398	0
2175	0,61699723	NA	0,00084798	3,94E-05	-0,00164748	0,4880593	-0,22142621	0,6960215	-0,05271384	NA	NA	NA	NA	-0,49283909	10	-20771,6295	41563,2712	3726,85453	0
2111	0,61699723	NA	0,00084798	3,94E-05	-0,00164748	0,4880593	-0,22142621	0,6894459	NA	NA	NA	NA	NA	-0,49566768	9	-20772,6542	41563,3183	3726,9016	0
669	-0,31227401	NA	NA	4,32E-05	-0,00145314	0,47935513	NA	-3,52383686	NA	0,05863003	NA	-0,00043937	NA	NA	8	-20774,135	41564,278	3727,86127	0
733	-0,31227401	NA	NA	4,32E-05	-0,00145314	0,47935513	NA	-3,4425274	-0,05282396	0,05811259	NA	-0,00050954	NA	NA	9	-20773,2973	41564,6046	3728,18783	0
671	-0,39160679	NA	0,00183718	4,30E-05	-0,00146197	0,48040455	NA	-3,52383686	NA	0,05863003	NA	-0,00043937	NA	NA	9	-20774,0916	41566,1932	3729,77644	0
735	-0,39160679	NA	0,00183718	4,30E-05	-0,00146197	0,48040455	NA	-3,4425274	-0,05282396	0,05811259	NA	-0,00050954	NA	NA	10	-20773,2539	41566,5199	3730,10322	0
2589	-0,31227401	NA	NA	4,32E-05	-0,00145314	0,47935513	NA	0,98389174	NA	NA	NA	-0,00044751	NA	-0,50601759	8	-20775,3164	41566,6407	3730,22398	0
3236	-9,79595589	0,69445228	0,04389505	NA	NA	NA	-0,55018722	-2,06058113	NA	0,05699765	NA	NA	0,49693597	-0,52441365	9	-20774,921	41567,8519	3731,43519	0
2591	-0,39160679	NA	0,00183718	4,30E-05	-0,00146197	0,48040455	NA	0,98389174	NA	NA	NA	-0,00044751	NA	-0,50601759	9	-20775,273	41568,5559	3732,13915	0
3175	-0,65771633	NA	-0,01187559	4,14E-05	NA	NA	-0,10732692	0,7406183	-0,11449835	NA	NA	NA	0,46966671	-0,53963709	9	-20775,5557	41569,1213	3732,70458	0
3623	-0,65771633	NA	-0,01187559	4,14E-05	NA	NA	-0,10732692	0,95852238	NA	NA	NA	-0,00035257	0,44577019	-0,55202249	9	-20775,7545	41569,519	3733,10229	0
3300	-9,79595589	0,69445228	0,04389505	NA	NA	NA	-0,55018722	-2,04753199	-0,01846226	0,05678211	NA	NA	0,49927467	-0,52377486	10	-20774,8033	41569,6187	3733,20198	0
3173	-1,22822825	NA	NA	4,02E-05	NA	NA	-0,09995451	0,7406183	-0,11449835	NA	NA	NA	0,46966671	-0,53963709	8	-20777,3001	41570,6082	3734,19149	0
3621	-1,22822825	NA	NA	4,02E-05	NA	NA	-0,09995451	0,95852238	NA	NA	NA	-0,00035257	0,44577019	-0,55202249	8	-20777,499	41571,0059	3734,58919	0
3143	-1,14142692	NA	-0,01089562	4,31E-05	NA	NA	NA	0,7406183	-0,11449835	NA	NA	NA	0,46966671	-0,53963709	8	-20777,6698	41571,3475	3734,9308	0
3591	-1,14142692	NA	-0,01089562	4,31E-05	NA	NA	NA	0,95852238	NA	NA	NA	-0,00035257	0,44577019	-0,55202249	8	-20777,8686	41571,7452	3735,3285	0
3250	-8,51824578	0,51124721	NA	NA	NA	0,38535796	-0,65213053	-2,06058113	NA	0,05699765	NA	NA	0,49693597	-0,52441365	9	-20776,9079	41571,8258	3735,4091	0
3241	-3,98530292	NA	NA	NA	-0,00234183	NA	-0,67333606	-2,06058113	NA	0,05699765	NA	NA	0,49693597	-0,52441365	8	-20778,0535	41572,1149	3735,69814	0
3141	-1,63084607	NA	NA	4,18E-05	NA	NA	NA	0,7406183	-0,11449835	NA	NA	NA	0,46966671	-0,53963709	7	-20779,145	41572,2961	3735,87939	0
3589	-1,63084607	NA	NA	4,18E-05	NA	NA	NA	0,95852238	NA	NA	NA	-0,00035257	0,44577019	-0,55202249	7	-20779,3438	41572,6938	3736,27709	0
3314	-8,51824578	0,51124721	NA	NA	NA	0,38535796	-0,65213053	-2,04753199	-0,01846226	0,05678211	NA	NA	0,49927467	-0,52377486	10	-20776,7902	41573,5926	3737,17589	0
3305	-3,98530292	NA	NA	NA	-0,00234183	NA	-0,67333606	-2,04753199	-0,01846226	0,05678211	NA	NA	0,49927467	-0,52377486	9	-20777,9357	41573,8814	3737,46471	0
157	-0,31227401	NA	NA	4,32E-05	-0,00145314	0,47935513	NA	-3,73836005	NA	0,05777952	NA	NA	NA	NA	7	-20781,0889	41576,184	3739,76729	0
3111	-0,65771633	NA	-0,01187559	4,14E-05	NA	NA	-0,10732692	0,72537252	NA	NA	NA	NA	0,45346103	-0,54414773	8	-20780,2686	41576,5451	3740,12843	0
3109	-1,22822825	NA	NA	4,02E-05	NA	NA	-0,09995451	0,72537252	NA	NA	NA	NA	0,45346103	-0,54414773	7	-20782,013	41578,0323	3741,61555	0
221	-0,31227401	NA	NA	4,32E-05	-0,00145314	0,47935513	NA	-3,74999095	0,01316736	0,05794033	NA	NA	NA	NA	8	-20781,0264	41578,0608	3741,64406	0

159	-0,39160679	NA	0,00183718	4,30E-05	-0,00146197	0,48040455	NA	-3,73836005	NA	0,05777952	NA	NA	NA	NA	8	-20781,0455	41578,099	3741,68224	0
3079	-1,14142692	NA	-0,01089562	4,31E-05	NA	NA	NA	0,72537252	NA	NA	NA	NA	0,45346103	-0,54414773	7	-20782,3827	41578,7716	3742,35486	0
2141	-0,31227401	NA	NA	4,32E-05	-0,00145314	0,47935513	NA	0,6960215	-0,05271384	NA	NA	NA	NA	-0,49283909	8	-20781,5417	41579,0913	3742,6746	0
2077	-0,31227401	NA	NA	4,32E-05	-0,00145314	0,47935513	NA	0,6894459	NA	NA	NA	NA	NA	-0,49566768	7	-20782,5663	41579,1388	3742,72212	0
3077	-1,63084607	NA	NA	4,18E-05	NA	NA	NA	0,72537252	NA	NA	NA	NA	0,45346103	-0,54414773	6	-20783,8579	41579,7204	3743,30367	0
223	-0,39160679	NA	0,00183718	4,30E-05	-0,00146197	0,48040455	NA	-3,74999095	0,01316736	0,05794033	NA	NA	NA	NA	9	-20780,983	41579,976	3743,55923	0
2143	-0,39160679	NA	0,00183718	4,30E-05	-0,00146197	0,48040455	NA	0,6960215	-0,05271384	NA	NA	NA	NA	-0,49283909	9	-20781,4983	41581,0065	3744,58977	0
2079	-0,39160679	NA	0,00183718	4,30E-05	-0,00146197	0,48040455	NA	0,6894459	NA	NA	NA	NA	NA	-0,49566768	8	-20782,5229	41581,0538	3744,63707	0
3810	-7,20552725	0,58574016	NA	NA	NA	NA	-0,62020516	-1,61532922	-0,10289105	0,0570521	NA	-0,00065333	0,49801214	-0,5357095	10	-20782,6531	41585,3184	3748,90166	0
3746	-7,20552725	0,58574016	NA	NA	NA	NA	-0,62020516	-1,76635397	NA	0,05800383	NA	-0,00051675	0,48731517	-0,53620329	9	-20785,6973	41589,4045	3752,98776	0
3827	-8,8387814	NA	0,03010563	NA	NA	0,51295904	-0,60572616	-1,61532922	-0,10289105	0,0570521	NA	-0,00065333	0,49801214	-0,5357095	11	-20785,6698	41593,3541	3756,93741	0
3763	-8,8387814	NA	0,03010563	NA	NA	0,51295904	-0,60572616	-1,76635397	NA	0,05800383	NA	-0,00051675	0,48731517	-0,53620329	10	-20788,7139	41597,44	3761,02328	0
1767	-0,65771633	NA	-0,01187559	4,14E-05	NA	NA	-0,10732692	-3,6371906	-0,09814646	0,05972263	NA	-0,00047389	0,39127251	NA	10	-20788,996	41598,0042	3761,58748	0
1765	-1,22822825	NA	NA	4,02E-05	NA	NA	-0,09995451	-3,6371906	-0,09814646	0,05972263	NA	-0,00047389	0,39127251	NA	9	-20790,7405	41599,4909	3763,07416	0
1735	-1,14142692	NA	-0,01089562	4,31E-05	NA	NA	NA	-3,6371906	-0,09814646	0,05972263	NA	-0,00047389	0,39127251	NA	9	-20791,1101	41600,2302	3763,81347	0
1733	-1,63084607	NA	NA	4,18E-05	NA	NA	NA	-3,6371906	-0,09814646	0,05972263	NA	-0,00047389	0,39127251	NA	8	-20792,5853	41601,1786	3764,76184	0
1703	-0,65771633	NA	-0,01187559	4,14E-05	NA	NA	-0,10732692	-3,78227707	NA	0,0606519	NA	-0,00034645	0,38158141	NA	9	-20791,8323	41601,6745	3765,25775	0
1701	-1,22822825	NA	NA	4,02E-05	NA	NA	-0,09995451	-3,78227707	NA	0,0606519	NA	-0,00034645	0,38158141	NA	8	-20793,5767	41603,1614	3766,74466	0
1671	-1,14142692	NA	-0,01089562	4,31E-05	NA	NA	NA	-3,78227707	NA	0,0606519	NA	-0,00034645	0,38158141	NA	8	-20793,9464	41603,9007	3767,48396	0
1669	-1,63084607	NA	NA	4,18E-05	NA	NA	NA	-3,78227707	NA	0,0606519	NA	-0,00034645	0,38158141	NA	7	-20795,4215	41604,8493	3768,43256	0
3234	-7,20552725	0,58574016	NA	NA	NA	NA	-0,62020516	-2,06058113	NA	0,05699765	NA	NA	0,49693597	-0,52441365	8	-20794,942	41605,8919	3769,47521	0
3298	-7,20552725	0,58574016	NA	NA	NA	NA	-0,62020516	-2,04753199	-0,01846226	0,05678211	NA	NA	0,49927467	-0,52377486	9	-20794,8243	41607,6585	3771,24178	0
1191	-0,65771633	NA	-0,01187559	4,14E-05	NA	NA	-0,10732692	-3,95454473	NA	0,05999672	NA	NA	0,38996522	NA	8	-20796,1754	41608,3588	3771,94206	0
2669	0,64576877	NA	NA	3,88E-05	-0,00183092	NA	-0,20438761	1,12236839	-0,1349839	NA	NA	-0,00063299	NA	-0,50295006	9	-20795,4647	41608,9394	3772,52271	0
1255	-0,65771633	NA	-0,01187559	4,14E-05	NA	NA	-0,10732692	-3,92430325	-0,03654601	0,05956532	NA	NA	0,39466847	NA	9	-20795,7043	41609,4185	3773,00177	0
2677	-0,84339357	NA	NA	4,07E-05	NA	0,55505772	-0,13640315	1,12236839	-0,1349839	NA	NA	-0,00063299	NA	-0,50295006	9	-20795,723	41609,4559	3773,03914	0
1189	-1,22822825	NA	NA	4,02E-05	NA	NA	-0,09995451	-3,95454473	NA	0,05999672	NA	NA	0,38996522	NA	7	-20797,9199	41609,8459	3773,42918	0
2679	-0,50593593	NA	-0,00700816	4,14E-05	NA	0,55021643	-0,14061875	1,12236839	-0,1349839	NA	NA	-0,00063299	NA	-0,50295006	10	-20795,0899	41610,192	3773,77523	0
1159	-1,14142692	NA	-0,01089562	4,31E-05	NA	NA	NA	-3,95454473	NA	0,05999672	NA	NA	0,38996522	NA	7	-20798,2895	41610,5852	3774,16849	0
2671	0,74969745	NA	-0,00245756	3,91E-05	-0,00181949	NA	-0,20513804	1,12236839	-0,1349839	NA	NA	-0,00063299	NA	-0,50295006	10	-20795,3892	41610,7905	3774,37377	0
1253	-1,22822825	NA	NA	4,02E-05	NA	NA	-0,09995451	-3,92430325	-0,03654601	0,05956532	NA	NA	0,39466847	NA	8	-20797,4487	41610,9054	3774,48867	0
1157	-1,63084607	NA	NA	4,18E-05	NA	NA	NA	-3,95454473	NA	0,05999672	NA	NA	0,38996522	NA	6	-20799,7647	41611,534	3775,1173	0
1223	-1,14142692	NA	-0,01089562	4,31E-05	NA	NA	NA	-3,92430325	-0,03654601	0,05956532	NA	NA	0,39466847	NA	8	-20797,8184	41611,6447	3775,22798	0
3825	-6,3315051	NA	NA	NA	NA	0,4838605	-0,6580268	-1,61532922	-0,10289105	0,0570521	NA	-0,00065333	0,49801214	-0,5357095	10	-20796,0843	41612,1808	3775,76405	0
1221	-1,63084607	NA	NA	4,18E-05	NA	NA	NA	-3,92430325	-0,03654601	0,05956532	NA	NA	0,39466847	NA	7	-20799,2936	41612,5933	3776,17657	0
3251	-8,8387814	NA	0,03010563	NA	NA	0,51295904	-0,60572616	-2,06058113	NA	0,05699765	NA	NA	0,49693597	-0,52441365	9	-20797,9586	41613,9272	3777,51051	0
2645	-1,36352414	NA	NA	4,29E-05	NA	0,54467715	NA	1,12236839	-0,1349839	NA	NA	-0,00063299	NA	-0,50295006	8	-20799,2633	41614,5346	3778,11791	0
2647	-1,08960867	NA	-0,00576757	4,36E-05	NA	0,53973144	NA	1,12236839	-0,1349839	NA	NA	-0,00063299	NA	-0,50295006	9	-20798,831	41615,672	3779,25532	0
685	0,64576877	NA	NA	3,88E-05	-0,00183092	NA	-0,20438761	-3,52383686	NA	0,05863003	NA	-0,00043937	NA	NA	8	-20799,837	41615,6819	3779,26521	0
3315	-8,8387814	NA	0,03010563	NA	NA	0,51295904	-0,60572616	-2,04753199	-0,01846226	0,05678211	NA	NA	0,49927467	-0,52377486	10	-20797,8409	41615,694	3779,2773	0
749	0,64576877	NA	NA	3,88E-05	-0,00183092	NA	-0,20438761	-3,4425274	-0,05282396	0,05811259	NA	-0,00050954	NA	NA	9	-20798,9993	41616,0085	3779,59178	0
693	-0,84339357	NA	NA	4,07E-05	NA	0,55505772	-0,13640315	-3,52383686	NA	0,05863003	NA	-0,00043937	NA	NA	8	-20800,0952	41616,1984	3779,78165	0
3761	-6,3315051	NA	NA	NA	NA	0,4838605	-0,6580268	-1,76635397	NA	0,05800383	NA	-0,00051675	0,48731517	-0,53620329	9	-20799,1285	41616,2669	3779,85014	0
757	-0,84339357	NA	NA	4,07E-05	NA	0,55505772	-0,13640315	-3,4425274	-0,05282396	0,05811259	NA	-0,00050954	NA	NA	9	-20799,2575	41616,5249	3780,10821	0
695	-0,50593593	NA	-0,00700816	4,14E-05	NA	0,55021643	-0,14061875	-3,52383686	NA	0,05863003	NA	-0,00043937	NA	NA	9	-20799,4621	41616,9342	3780,51752	0
759	-0,50593593	NA	-0,00700816	4,14E-05	NA	0,55021643	-0,14061875	-3,4425274	-0,05282396	0,05811259	NA	-0,00050954	NA	NA	10	-20798,6244	41617,261	3780,84431	0
687	0,74969745	NA	-0,00245756	3,91E-05	-0,00181949	NA	-0,20513804	-3,52383686	NA	0,05863003	NA	-0,00043937	NA	NA	9	-20799,7614	41617,5328	3781,11606	0
751	0,74969745	NA	-0,00245756	3,91E-05	-0,00181949	NA	-0,20513804	-3,4425274	-0,05282396	0,05811259	NA	-0,00050954	NA	NA	10	-20798,9237	41617,8596	3781,44284	0
2605	0,64576877	NA	NA	3,88E-05	-0,00183092	NA	-0,20438761	0,98389174	NA	NA	NA	-0,00044751	NA	-0,50601759	8	-20801,0183	41618,0446	3781,62792	0
2613	-0,84339357	NA	NA	4,07E-05	NA	0,55505772	-0,13640315	0,98389174	NA	NA	NA	-0,00044751	NA	-0,50601759	8	-20801,2766	41618,5611	3782,14436	0
2615	-0,50593593	NA	-0,00700816	4,14E-05	NA	0,55021643	-0,14061875	0,98389174	NA	NA	NA	-0,00044751	NA	-0,50601759	9	-20800,6435	41619,2969	3782,88023	0
2607	0,74969745	NA	-0,00245756	3,91E-05	-0,00181949	NA	-0,20513804	0,98389174	NA	NA	NA	-0,00044751	NA	-0,50601759	9	-20800,9428	41619,8955	3783,47877	0
661	-1,36352414	NA	NA	4,29E-05	NA	0,54467715	NA	-3,52383686	NA	0,05863003	NA	-0,00043937	NA	NA	7	-20803,6356	41621,2774	3784,86064	0
725	-1,36352414	NA	NA	4,29E-05	NA	0,54467715	NA	-3,4425274	-0,05282396	0,05811259	NA	-0,00050954	NA	NA	8	-20802,7979	41621,6037	3785,18698	0
663	-1,08960867	NA	-0,00576757	4,36E-05	NA	0,53973144	NA	-3,52383686	NA	0,05863003	NA	-0,00043937	NA	NA	8	-20803,2033	41622,4145	3785,99783	0
2748	-5,95790631	0,3741151	0,04613303	NA	-0,00180249	0,39660544	-0,62074526	-1,63848397	NA	0,05580518	NA	-0,00060814	NA	-0,48687105	11	-20800,2271	41622,4688	3786,05203	0
727	-1,08960867	NA	-0,00576757	4,36E-05	NA	0,53973144	NA	-3,4425274	-0,05282396	0,05811259	NA	-0,00050954	NA	NA	9	-20802,3656	41622,7411	3786,3244	0
2812	-5,95790631	0,3741151	0,04613303	NA	-0,00180249	0,39660544	-0,62074526	-1,57337207	-0,04401868	0,05538852	NA	-0,0006675	NA	-0,48613452	12	-20799,656	41623,3292	3786,91252	0
2637	-0,28011525	NA	NA	4,24E-05	-0,0016491	NA	NA	1,12236839	-0,1349839	NA	NA	-0,00063299	NA	-0,50295006	8	-20803,755	41623,518	3787,10133	0
2581	-1,36352414	NA	NA	4,29E-05	NA	0,54467715	NA	0,98389174	NA	NA	NA	-0,00044751	NA	-0,50601759	7	-20804,8169	41623,6401	3787,22335	0
2583	-1,08960867	NA	-0,00576757	4,36E-05	NA	0,53973144	NA	0,98389174	NA	NA	NA	-0,00044751	NA	-0,50601759	8	-20804,3846	41624,7773	3788,36054	0

2639	-0,2131598	NA	-0,0015413	4,25E-05	-0,00164139	NA	NA	1,12236839	-0,1349839	NA	NA	-0,00063299	NA	-0,50295006	9	-20803,7253	41625,4607	3789,04393	0
3804	-9,61919161	0,46601352	0,06534536	NA	-0,00131078	0,33938341	NA	-1,61532922	-0,10289105	0,0570521	NA	-0,00065333	0,49801214	-0,5357095	12	-20801,042	41626,1013	3789,68459	0
173	0,64576877	NA	NA	3,88E-05	-0,00183092	NA	-0,20438761	-3,73836005	NA	0,05777952	NA	NA	NA	NA	7	-20806,7909	41627,588	3791,17124	0
181	-0,84339357	NA	NA	4,07E-05	NA	0,55505772	-0,13640315	-3,73836005	NA	0,05777952	NA	NA	NA	NA	7	-20807,0491	41628,1044	3791,68767	0
183	-0,50593593	NA	-0,00700816	4,14E-05	NA	0,55021643	-0,14061875	-3,73836005	NA	0,05777952	NA	NA	NA	NA	8	-20806,416	41628,84	3792,42332	0
175	0,74969745	NA	-0,00245756	3,91E-05	-0,00181949	NA	-0,20513804	-3,73836005	NA	0,05777952	NA	NA	NA	NA	8	-20806,7153	41629,4386	3793,02186	0
237	0,64576877	NA	NA	3,88E-05	-0,00183092	NA	-0,20438761	-3,74999095	0,01316736	0,05794033	NA	NA	NA	NA	8	-20806,7284	41629,4647	3793,04801	0
245	-0,84339357	NA	NA	4,07E-05	NA	0,55505772	-0,13640315	-3,74999095	0,01316736	0,05794033	NA	NA	NA	NA	8	-20806,9866	41629,9812	3793,56444	0
3740	-9,61919161	0,46601352	0,06534536	NA	-0,00131078	0,33938341	NA	-1,76635397	NA	0,05800383	NA	-0,00051675	0,48731517	-0,53620329	11	-20804,0862	41630,187	3793,77024	0
653	-0,28011525	NA	NA	4,24E-05	-0,0016491	NA	NA	-3,52383686	NA	0,05863003	NA	-0,00043937	NA	NA	7	-20808,1273	41630,2608	3793,84406	0
2157	0,64576877	NA	NA	3,88E-05	-0,00183092	NA	-0,20438761	0,6960215	-0,05271384	NA	NA	NA	NA	-0,49283909	8	-20807,2437	41630,4953	3794,07855	0
2093	0,64576877	NA	NA	3,88E-05	-0,00183092	NA	-0,20438761	0,6894459	NA	NA	NA	NA	NA	-0,49566768	7	-20808,2683	41630,5428	3794,12607	0
717	-0,28011525	NA	NA	4,24E-05	-0,0016491	NA	NA	-3,4425274	-0,05282396	0,05811259	NA	-0,00050954	NA	NA	8	-20807,2896	41630,5871	3794,1704	0
247	-0,50593593	NA	-0,00700816	4,14E-05	NA	0,55021643	-0,14061875	-3,74999095	0,01316736	0,05794033	NA	NA	NA	NA	9	-20806,3535	41630,717	3794,30031	0
2165	-0,84339357	NA	NA	4,07E-05	NA	0,55505772	-0,13640315	0,6960215	-0,05271384	NA	NA	NA	NA	-0,49283909	8	-20807,5019	41631,0117	3794,59498	0
2101	-0,84339357	NA	NA	4,07E-05	NA	0,55505772	-0,13640315	0,6894459	NA	NA	NA	NA	NA	-0,49566768	7	-20808,5265	41631,0592	3794,6425	0
239	0,74969745	NA	-0,00245756	3,91E-05	-0,00181949	NA	-0,20513804	-3,74999095	0,01316736	0,05794033	NA	NA	NA	NA	9	-20806,6528	41631,3156	3794,89885	0
2167	-0,50593593	NA	-0,00700816	4,14E-05	NA	0,55021643	-0,14061875	0,6960215	-0,05271384	NA	NA	NA	NA	-0,49283909	9	-20806,8688	41631,7476	3795,33085	0
2103	-0,50593593	NA	-0,00700816	4,14E-05	NA	0,55021643	-0,14061875	0,6894459	NA	NA	NA	NA	NA	-0,49566768	8	-20807,8935	41631,7949	3795,37815	0
655	-0,2131598	NA	-0,0015413	4,25E-05	-0,00164139	NA	NA	-3,52383686	NA	0,05863003	NA	-0,00043937	NA	NA	8	-20808,0976	41632,2032	3795,78644	0
2159	0,74969745	NA	-0,00245756	3,91E-05	-0,00181949	NA	-0,20513804	0,6960215	-0,05271384	NA	NA	NA	NA	-0,49283909	9	-20807,1681	41632,3461	3795,92939	0
2095	0,74969745	NA	-0,00245756	3,91E-05	-0,00181949	NA	-0,20513804	0,6894459	NA	NA	NA	NA	NA	-0,49566768	8	-20808,1927	41632,3934	3795,97669	0
719	-0,2131598	NA	-0,0015413	4,25E-05	-0,00164139	NA	NA	-3,4425274	-0,05282396	0,05811259	NA	-0,00050954	NA	NA	9	-20807,2599	41632,5297	3796,11301	0
2573	-0,28011525	NA	NA	4,24E-05	-0,0016491	NA	NA	0,98389174	NA	NA	NA	-0,00044751	NA	-0,50601759	7	-20809,3086	41632,6235	3796,20677	0
3249	-6,3315051	NA	NA	NA	NA	0,4838605	-0,6580268	-2,06058113	NA	0,05699765	NA	NA	0,49693597	-0,52441365	8	-20808,3732	41632,7543	3796,33759	0
149	-1,36352414	NA	NA	4,29E-05	NA	0,54467715	NA	-3,73836005	NA	0,05777952	NA	NA	NA	NA	6	-20810,5895	41633,1836	3796,76689	0
151	-1,08960867	NA	-0,00576757	4,36E-05	NA	0,53973144	NA	-3,73836005	NA	0,05777952	NA	NA	NA	NA	7	-20810,1572	41634,3206	3797,90386	0
3313	-6,3315051	NA	NA	NA	NA	0,4838605	-0,6580268	-2,04753199	-0,01846226	0,05678211	NA	NA	0,49927467	-0,52377486	9	-20808,2555	41634,5209	3798,10416	0
2575	-0,2131598	NA	-0,0015413	4,25E-05	-0,00164139	NA	NA	0,98389174	NA	NA	NA	-0,00044751	NA	-0,50601759	8	-20809,279	41634,5659	3798,14915	0
213	-1,36352414	NA	NA	4,29E-05	NA	0,54467715	NA	-3,74999095	0,01316736	0,05794033	NA	NA	NA	NA	7	-20810,527	41635,0602	3798,64343	0
2133	-1,36352414	NA	NA	4,29E-05	NA	0,54467715	NA	0,6960215	-0,05271384	NA	NA	NA	NA	-0,49283909	7	-20811,0423	41636,0907	3799,67397	0
2069	-1,36352414	NA	NA	4,29E-05	NA	0,54467715	NA	0,6894459	NA	NA	NA	NA	NA	-0,49566768	6	-20812,0669	41636,1384	3799,72171	0
215	-1,08960867	NA	-0,00576757	4,36E-05	NA	0,53973144	NA	-3,74999095	0,01316736	0,05794033	NA	NA	NA	NA	8	-20810,0947	41636,1973	3799,78063	0
2135	-1,08960867	NA	-0,00576757	4,36E-05	NA	0,53973144	NA	0,6960215	-0,05271384	NA	NA	NA	NA	-0,49283909	8	-20810,61	41637,2279	3800,81117	0
2071	-1,08960867	NA	-0,00576757	4,36E-05	NA	0,53973144	NA	0,6894459	NA	NA	NA	NA	NA	-0,49566768	7	-20811,6346	41637,2754	3800,85868	0
141	-0,28011525	NA	NA	4,24E-05	-0,0016491	NA	NA	-3,73836005	NA	0,05777952	NA	NA	NA	NA	6	-20815,0812	41642,167	3805,7503	0
205	-0,28011525	NA	NA	4,24E-05	-0,0016491	NA	NA	-3,74999095	0,01316736	0,05794033	NA	NA	NA	NA	7	-20815,0187	41644,0436	3807,62685	0
143	-0,2131598	NA	-0,0015413	4,25E-05	-0,00164139	NA	NA	-3,73836005	NA	0,05777952	NA	NA	NA	NA	7	-20815,0515	41644,1092	3807,69246	0
2747	-8,35316771	NA	0,03864408	NA	-0,00234345	0,43617682	-0,64441699	-1,63848397	NA	0,05580518	NA	-0,00060814	NA	-0,48687105	10	-20812,1327	41644,2776	3807,86091	0
2125	-0,28011525	NA	NA	4,24E-05	-0,0016491	NA	NA	0,6960215	-0,05271384	NA	NA	NA	NA	-0,49283909	7	-20815,534	41645,0741	3808,65739	0
2061	-0,28011525	NA	NA	4,24E-05	-0,0016491	NA	NA	0,6894459	NA	NA	NA	NA	NA	-0,49566768	6	-20816,5586	41645,1219	3808,70513	0
2811	-8,35316771	NA	0,03864408	NA	-0,00234345	0,43617682	-0,64441699	-1,57337207	-0,04401868	0,05538852	NA	-0,0006675	NA	-0,48613452	11	-20811,5616	41645,1379	3808,72118	0
207	-0,2131598	NA	-0,0015413	4,25E-05	-0,00164139	NA	NA	-3,74999095	0,01316736	0,05794033	NA	NA	NA	NA	8	-20814,989	41645,986	3809,56924	0
2236	-5,95790631	0,3741151	0,04613303	NA	-0,00180249	0,39660544	-0,62074526	-1,98285198	NA	0,054624	NA	NA	NA	-0,47270606	10	-20813,0091	41646,0303	3809,61359	0
3228	-9,61919161	0,46601352	0,06534536	NA	-0,00131078	0,33938341	NA	-2,06058113	NA	0,05699765	NA	NA	0,49693597	-0,52441365	10	-20813,3309	41646,674	3810,25724	0
2300	-5,95790631	0,3741151	0,04613303	NA	-0,00180249	0,39660544	-0,62074526	-2,01223022	0,04141198	0,05511629	NA	NA	NA	-0,47466077	11	-20812,4025	41646,8196	3810,40285	0
2127	-0,2131598	NA	-0,0015413	4,25E-05	-0,00164139	NA	NA	0,6960215	-0,05271384	NA	NA	NA	NA	-0,49283909	8	-20815,5043	41647,0165	3810,59978	0
2063	-0,2131598	NA	-0,0015413	4,25E-05	-0,00164139	NA	NA	0,6894459	NA	NA	NA	NA	NA	-0,49566768	7	-20816,5289	41647,064	3810,64729	0
3292	-9,61919161	0,46601352	0,06534536	NA	-0,00131078	0,33938341	NA	-2,04753199	-0,01846226	0,05678211	NA	NA	0,49927467	-0,52377486	11	-20813,2132	41648,441	3812,02426	0
3796	-13,7669049	0,65178191	0,06462958	NA	NA	0,35119819	NA	-1,61532922	-0,10289105	0,0570521	NA	-0,00065333	0,49801214	-0,5357095	11	-20814,9243	41651,8633	3815,44655	0
3788	-11,9654714	0,51367836	0,06255183	NA	-0,0013444	NA	NA	-1,61532922	-0,10289105	0,0570521	NA	-0,00065333	0,49801214	-0,5357095	11	-20815,1129	41652,2404	3815,82369	0
3732	-13,7669049	0,65178191	0,06462958	NA	NA	0,35119819	NA	-1,76635397	NA	0,05800383	NA	-0,00051675	0,48731517	-0,53620329	10	-20817,9685	41655,9491	3819,53242	0
3724	-11,9654714	0,51367836	0,06255183	NA	-0,0013444	NA	NA	-1,76635397	NA	0,05800383	NA	-0,00051675	0,48731517	-0,53620329	10	-20818,1571	41656,3263	3819,90956	0
3811	-9,52399274	NA	0,02470059	NA	NA	NA	-0,56950148	-1,61532922	-0,10289105	0,0570521	NA	-0,00065333	0,49801214	-0,5357095	10	-20818,7094	41657,431	3821,01432	0
2732	-9,35928532	0,43489058	0,04364193	NA	-0,00182965	NA	-0,59565606	-1,63848397	NA	0,05580518	NA	-0,00060814	NA	-0,48687105	10	-20819,3304	41658,6729	3822,25621	0
2796	-9,35928532	0,43489058	0,04364193	NA	-0,00182965	NA	-0,59565606	-1,57337207	-0,04401868	0,05538852	NA	-0,0006675	NA	-0,48613452	11	-20818,7593	41659,5332	3823,11648	0
3803	-11,3659105	NA	0,05744064	NA	-0,00198557	0,38445946	NA	-1,61532922	-0,10289105	0,0570521	NA	-0,00065333	0,49801214	-0,5357095	11	-20819,5349	41661,0845	3824,66777	0
3747	-9,52399274	NA	0,02470059	NA	NA	NA	-0,56950148	-1,76635397	NA	0,05800383	NA	-0,00051675	0,48731517	-0,53620329	9	-20821,7536	41661,5171	3825,10042	0
2746	-6,98837994	0,26427623	NA	NA	-0,00180413	0,37036432	-0,69230587	-1,63848397	NA	0,05580518	NA	-0,00060814	NA	-0,48687105	10	-20822,551	41665,1141	3828,69739	0
3739	-11,3659105	NA	0,05744064	NA	-0,00198557	0,38445946	NA	-1,76635397	NA	0,05800383	NA	-0,00051675	0,48731517	-0,53620329	10	-20822,5791	41665,1704	3828,75364	0
2810	-6,98837994	0,26427623	NA	NA	-0,00180413	0,37036432	-0,69230587	-1,57337207	-0,04401868	0,05538852	NA	-0,0006675	NA	-0,48613452	11	-20821,9799	41665,9744	3829,55766	0

2235	-8,35316771	NA	0,03864408	NA	-0,00234345	0,43617682	-0,64441699	-1,98285198	NA	0,054624	NA	NA	NA	-0,47270606	9	-20824,9147	41667,8394	3831,42269	0
2299	-8,35316771	NA	0,03864408	NA	-0,00234345	0,43617682	-0,64441699	-2,01223022	0,04141198	0,05511629	NA	NA	NA	-0,47466077	10	-20824,3081	41668,6284	3832,21173	0
3809	-8,58193391	NA	NA	NA	NA	NA	-0,61393687	-1,61532922	-0,10289105	0,0570521	NA	-0,00065333	0,49801214	-0,5357095	9	-20825,7255	41669,461	3833,04427	0
2740	-10,737769	0,62231984	0,04626548	NA	NA	0,40997724	-0,57836783	-1,63848397	NA	0,05580518	NA	-0,00060814	NA	-0,48687105	10	-20825,5583	41671,1288	3834,71208	0
2804	-10,737769	0,62231984	0,04626548	NA	NA	0,40997724	-0,57836783	-1,57337207	-0,04401868	0,05538852	NA	-0,0006675	NA	-0,48613452	11	-20824,9872	41671,9891	3835,57235	0
3220	-13,7669049	0,65178191	0,06462958	NA	NA	0,35119819	NA	-2,06058113	NA	0,05699765	NA	NA	0,49693597	-0,52441365	9	-20827,2132	41672,4364	3836,01965	0
3212	-11,9654714	0,51367836	0,06255183	NA	-0,0013444	NA	NA	-2,06058113	NA	0,05699765	NA	NA	0,49693597	-0,52441365	9	-20827,4018	41672,8135	3836,39679	0
3745	-8,58193391	NA	NA	NA	NA	NA	-0,61393687	-1,76635397	NA	0,05800383	NA	-0,00051675	0,48731517	-0,53620329	8	-20828,7697	41673,5473	3837,13059	0
3284	-13,7669049	0,65178191	0,06462958	NA	NA	0,35119819	NA	-2,04753199	-0,01846226	0,05678211	NA	NA	0,49927467	-0,52377486	10	-20827,0955	41674,2032	3837,78644	0
3276	-11,9654714	0,51367836	0,06255183	NA	-0,0013444	NA	NA	-2,04753199	-0,01846226	0,05678211	NA	NA	0,49927467	-0,52377486	10	-20827,2841	41674,5803	3838,16358	0
2745	-5,46460783	NA	NA	NA	-0,00220571	0,40342229	-0,70298126	-1,63848397	NA	0,05580518	NA	-0,00060814	NA	-0,48687105	9	-20828,8571	41675,7241	3839,30734	0
2809	-5,46460783	NA	NA	NA	-0,00220571	0,40342229	-0,70298126	-1,57337207	-0,04401868	0,05538852	NA	-0,0006675	NA	-0,48613452	10	-20828,286	41676,5841	3840,16738	0
3235	-9,52399274	NA	0,02470059	NA	NA	NA	-0,56950148	-2,06058113	NA	0,05699765	NA	NA	0,49693597	-0,52441365	8	-20830,9983	41678,0046	3841,58786	0
3299	-9,52399274	NA	0,02470059	NA	NA	NA	-0,56950148	-2,04753199	-0,01846226	0,05678211	NA	NA	0,49927467	-0,52377486	9	-20830,8806	41679,7712	3843,35443	0
3780	-12,0135881	0,70944129	0,06178489	NA	NA	NA	NA	-1,61532922	-0,10289105	0,0570521	NA	-0,00065333	0,49801214	-0,5357095	10	-20830,0113	41680,0348	3843,61809	0
3227	-11,3659105	NA	0,05744064	NA	-0,00198557	0,38445946	NA	-2,06058113	NA	0,05699765	NA	NA	0,49693597	-0,52441365	9	-20831,8238	41681,6576	3845,24087	0
2220	-9,35928532	0,43489058	0,04364193	NA	-0,00182965	NA	-0,59565606	-1,98285198	NA	0,054624	NA	NA	NA	-0,47270606	9	-20832,1124	41682,2347	3845,818	0
2284	-9,35928532	0,43489058	0,04364193	NA	-0,00182965	NA	-0,59565606	-2,01223022	0,04141198	0,05511629	NA	NA	NA	-0,47466077	10	-20831,5058	41683,0238	3846,60703	0
3291	-11,3659105	NA	0,05744064	NA	-0,00198557	0,38445946	NA	-2,04753199	-0,01846226	0,05678211	NA	NA	0,49927467	-0,52377486	10	-20831,7061	41683,4244	3847,00766	0
3716	-12,0135881	0,70944129	0,06178489	NA	NA	NA	NA	-1,76635397	NA	0,05800383	NA	-0,00051675	0,48731517	-0,53620329	9	-20833,0555	41684,1209	3847,70418	0
2234	-6,98837994	0,26427623	NA	NA	-0,00180413	0,37036432	-0,69230587	-1,98285198	NA	0,054624	NA	NA	NA	-0,47270606	9	-20835,333	41688,6759	3852,25918	0
2731	-5,75020926	NA	0,0346068	NA	-0,00247017	NA	-0,61951905	-1,63848397	NA	0,05580518	NA	-0,00060814	NA	-0,48687105	9	-20835,6837	41689,3773	3852,96058	0
2298	-6,98837994	0,26427623	NA	NA	-0,00180413	0,37036432	-0,69230587	-2,01223022	0,04141198	0,05511629	NA	NA	NA	-0,47466077	10	-20834,7264	41689,4649	3853,04821	0
3233	-8,58193391	NA	NA	NA	NA	NA	-0,61393687	-2,06058113	NA	0,05699765	NA	NA	0,49693597	-0,52441365	7	-20838,0144	41690,035	3853,61826	0
2795	-5,75020926	NA	0,0346068	NA	-0,00247017	NA	-0,61951905	-1,57337207	-0,04401868	0,05538852	NA	-0,0006675	NA	-0,48613452	10	-20835,1126	41690,2373	3853,82063	0
3297	-8,58193391	NA	NA	NA	NA	NA	-0,61393687	-2,04753199	-0,01846226	0,05678211	NA	NA	0,49927467	-0,52377486	8	-20837,8967	41691,8013	3855,3846	0
2228	-10,737769	0,62231984	0,04626548	NA	NA	0,40997724	-0,57836783	-1,98285198	NA	0,054624	NA	NA	NA	-0,47270606	9	-20838,3403	41694,6906	3858,27387	0
2292	-10,737769	0,62231984	0,04626548	NA	NA	0,40997724	-0,57836783	-2,01223022	0,04141198	0,05511629	NA	NA	NA	-0,47466077	10	-20837,7337	41695,4796	3859,0629	0
3787	-9,45532716	NA	0,05329757	NA	-0,00210061	NA	NA	-1,61532922	-0,10289105	0,0570521	NA	-0,00065333	0,49801214	-0,5357095	10	-20837,8905	41695,7931	3859,37641	0
2663	-0,65771633	NA	-0,01187559	4,14E-05	NA	NA	-0,10732692	1,12236839	-0,1349839	NA	NA	-0,00063299	NA	-0,50295006	9	-20839,0054	41696,0208	3859,60407	0
2730	-6,22256645	0,32760888	NA	NA	-0,00183217	NA	-0,66370241	-1,63848397	NA	0,05580518	NA	-0,00060814	NA	-0,48687105	9	-20839,2322	41696,4744	3860,05764	0
2794	-6,22256645	0,32760888	NA	NA	-0,00183217	NA	-0,66370241	-1,57337207	-0,04401868	0,05538852	NA	-0,0006675	NA	-0,48613452	10	-20838,6611	41697,3344	3860,91769	0
2661	-1,22822825	NA	NA	4,02E-05	NA	NA	-0,09995451	1,12236839	-0,1349839	NA	NA	-0,00063299	NA	-0,50295006	8	-20840,7499	41697,5077	3861,09097	0
2631	-1,14142692	NA	-0,01089562	4,31E-05	NA	NA	NA	1,12236839	-0,1349839	NA	NA	-0,00063299	NA	-0,50295006	8	-20841,1195	41698,247	3861,83028	0
2629	-1,63084607	NA	NA	4,18E-05	NA	NA	NA	1,12236839	-0,1349839	NA	NA	-0,00063299	NA	-0,50295006	7	-20842,5947	41699,1956	3862,77887	0
2233	-5,46460783	NA	NA	NA	-0,00220571	0,40342229	-0,70298126	-1,98285198	NA	0,054624	NA	NA	NA	-0,47270606	8	-20841,6391	41699,2861	3862,86934	0
3723	-9,45532716	NA	0,05329757	NA	-0,00210061	NA	NA	-1,76635397	NA	0,05800383	NA	-0,00051675	0,48731517	-0,53620329	9	-20840,9346	41699,8792	3863,46251	0
2297	-5,46460783	NA	NA	NA	-0,00220571	0,40342229	-0,70298126	-2,01223022	0,04141198	0,05511629	NA	NA	NA	-0,47466077	9	-20841,0325	41700,0749	3863,65816	0
3204	-12,0135881	0,70944129	0,06178489	NA	NA	NA	NA	-2,06058113	NA	0,05699765	NA	NA	0,49693597	-0,52441365	8	-20842,3002	41700,6084	3864,19163	0
3268	-12,0135881	0,70944129	0,06178489	NA	NA	NA	NA	-2,04753199	-0,01846226	0,05678211	NA	NA	0,49927467	-0,52377486	9	-20842,1825	41702,3749	3865,9582	0
679	-0,65771633	NA	-0,01187559	4,14E-05	NA	NA	-0,10732692	-3,52383686	NA	0,05863003	NA	-0,00043937	NA	NA	8	-20843,3777	41702,7633	3866,34658	0
743	-0,65771633	NA	-0,01187559	4,14E-05	NA	NA	-0,10732692	-3,4425274	-0,05282396	0,05811259	NA	-0,00050954	NA	NA	9	-20842,54	41703,0899	3866,67314	0
677	-1,22822825	NA	NA	4,02E-05	NA	NA	-0,09995451	-3,52383686	NA	0,05863003	NA	-0,00043937	NA	NA	7	-20845,1221	41704,2504	3867,8337	0
741	-1,22822825	NA	NA	4,02E-05	NA	NA	-0,09995451	-3,4425274	-0,05282396	0,05811259	NA	-0,00050954	NA	NA	8	-20844,2844	41704,5768	3868,16005	0
647	-1,14142692	NA	-0,01089562	4,31E-05	NA	NA	NA	-3,52383686	NA	0,05863003	NA	-0,00043937	NA	NA	7	-20845,4918	41704,9897	3868,57301	0
2599	-0,65771633	NA	-0,01187559	4,14E-05	NA	NA	-0,10732692	0,98389174	NA	NA	NA	-0,00044751	NA	-0,50601759	8	-20844,559	41705,126	3868,70929	0
711	-1,14142692	NA	-0,01089562	4,31E-05	NA	NA	NA	-3,4425274	-0,05282396	0,05811259	NA	-0,00050954	NA	NA	8	-20844,6541	41705,3161	3868,89936	0
645	-1,63084607	NA	NA	4,18E-05	NA	NA	NA	-3,52383686	NA	0,05863003	NA	-0,00043937	NA	NA	6	-20846,967	41705,9385	3869,52182	0
709	-1,63084607	NA	NA	4,18E-05	NA	NA	NA	-3,4425274	-0,05282396	0,05811259	NA	-0,00050954	NA	NA	7	-20846,1292	41706,2647	3869,84795	0
2597	-1,22822825	NA	NA	4,02E-05	NA	NA	-0,09995451	0,98389174	NA	NA	NA	-0,00044751	NA	-0,50601759	7	-20846,3035	41706,6131	3870,19641	0
2567	-1,14142692	NA	-0,01089562	4,31E-05	NA	NA	NA	0,98389174	NA	NA	NA	-0,00044751	NA	-0,50601759	7	-20846,6731	41707,3524	3870,93572	0
1662	-0,06671637	0,57575795	NA	4,34E-05	-0,00071955	0,41051829	-0,17478186	-0,7738147	-0,19560429	NA	NA	-0,00045111	0,35589058	NA	11	-20842,7706	41707,5559	3871,13917	0
1664	-0,47166397	0,58945108	0,00876766	4,25E-05	-0,00073797	0,4135805	-0,1717464	-0,7738147	-0,19560429	NA	NA	-0,00045111	0,35589058	NA	12	-20841,7965	41707,6102	3871,19345	0
2565	-1,63084607	NA	NA	4,18E-05	NA	NA	NA	0,98389174	NA	NA	NA	-0,00044751	NA	-0,50601759	6	-20848,1483	41708,3013	3871,88453	0
2724	-9,79595589	0,69445228	0,04389505	NA	NA	NA	-0,55018722	-1,63848397	NA	0,05580518	NA	-0,00060814	NA	-0,48687105	9	-20845,9708	41709,9517	3873,53493	0
2788	-9,79595589	0,69445228	0,04389505	NA	NA	NA	-0,55018722	-1,57337207	-0,04401868	0,05538852	NA	-0,0006675	NA	-0,48613452	10	-20845,3998	41710,8117	3874,39498	0
2219	-5,75020926	NA	0,0346068	NA	-0,00247017	NA	-0,61951905	-1,98285198	NA	0,054624	NA	NA	NA	-0,47270606	8	-20848,4657	41712,9393	3876,52259	0
2283	-5,75020926	NA	0,0346068	NA	-0,00247017	NA	-0,61951905	-2,01223022	0,04141198	0,05511629	NA	NA	NA	-0,47466077	9	-20847,8591	41713,7281	3877,3114	0
2738	-8,51824578	0,51124721	NA	NA	NA	0,38535796	-0,65213053	-1,63848397	NA	0,05580518	NA	-0,00060814	NA	-0,48687105	9	-20847,9578	41713,9256	3877,50884	0
2729	-3,98530292	NA	NA	NA	-0,00234183	NA	-0,67333606	-1,63848397	NA	0,05580518	NA	-0,00060814	NA	-0,48687105	8	-20849,1033	41714,2146	3877,79788	0
167	-0,65771633	NA	-0,01187559	4,14E-05	NA	NA	-0,10732692	-3,73836005	NA	0,05777952	NA	NA	NA	NA	7	-20850,3316	41714,6693	3878,2526	0

2802	-8,51824578	0,51124721	NA	NA	NA	0,38535796	-0,65213053	-1,57337207	-0,04401868	0,05538852	NA	-0,0006675	NA	-0,48613452	10	-20847,3867	41714,7856	3878,36888	0
2793	-3,98530292	NA	NA	NA	-0,00234183	NA	-0,67333606	-1,57337207	-0,04401868	0,05538852	NA	-0,0006675	NA	-0,48613452	9	-20848,5322	41715,0744	3878,65771	0
1654	-0,67953035	0,68234123	NA	4,46E-05	NA	0,41541375	-0,13795142	-0,7738147	-0,19560429	NA	NA	-0,00045111	0,35589058	NA	10	-20847,9573	41715,9267	3879,50996	0
165	-1,22822825	NA	NA	4,02E-05	NA	NA	-0,09995451	-3,73836005	NA	0,05777952	NA	NA	NA	NA	6	-20852,076	41716,1567	3879,73995	0
3211	-9,45532716	NA	0,05329757	NA	-0,00210061	NA	NA	-2,06058113	NA	0,05699765	NA	NA	0,49693597	-0,52441365	8	-20850,1794	41716,3667	3879,94996	0
1656	-1,04079715	0,69637088	0,00755047	4,39E-05	NA	0,41816409	-0,13461471	-0,7738147	-0,19560429	NA	NA	-0,00045111	0,35589058	NA	11	-20847,238	41716,4906	3880,0739	0
231	-0,65771633	NA	-0,01187559	4,14E-05	NA	NA	-0,10732692	-3,74999095	0,01316736	0,05794033	NA	NA	NA	NA	8	-20850,2691	41716,5461	3880,12937	0
135	-1,14142692	NA	-0,01089562	4,31E-05	NA	NA	NA	-3,73836005	NA	0,05777952	NA	NA	NA	NA	6	-20852,4457	41716,896	3880,47926	0
2151	-0,65771633	NA	-0,01187559	4,14E-05	NA	NA	-0,10732692	0,6960215	-0,05271384	NA	NA	NA	NA	-0,49283909	8	-20850,7843	41717,5766	3881,15991	0
2087	-0,65771633	NA	-0,01187559	4,14E-05	NA	NA	-0,10732692	0,6894459	NA	NA	NA	NA	NA	-0,49566768	7	-20851,809	41717,6242	3881,20743	0
133	-1,63084607	NA	NA	4,18E-05	NA	NA	NA	-3,73836005	NA	0,05777952	NA	NA	NA	NA	5	-20853,9208	41717,845	3881,42829	0
229	-1,22822825	NA	NA	4,02E-05	NA	NA	-0,09995451	-3,74999095	0,01316736	0,05794033	NA	NA	NA	NA	7	-20852,0135	41718,0332	3881,6165	0
1632	-1,28042467	0,61751101	0,00965662	4,55E-05	-0,00054807	0,40424516	NA	-0,7738147	-0,19560429	NA	NA	-0,00045111	0,35589058	NA	11	-20848,0427	41718,1	3881,68323	0
1150	-0,06671637	0,57575795	NA	4,34E-05	-0,00071955	0,41051829	-0,17478186	-1,05468211	-0,1361791	NA	NA	NA	0,35946641	NA	10	-20849,0575	41718,1271	3881,71041	0
3275	-9,45532716	NA	0,05329757	NA	-0,00210061	NA	NA	-2,04753199	-0,01846226	0,05678211	NA	NA	0,49927467	-0,52377486	9	-20850,0616	41718,1332	3881,71652	0
1152	-0,47166397	0,58945108	0,00876766	4,25E-05	-0,00073797	0,4135805	-0,1717464	-1,05468211	-0,1361791	NA	NA	NA	0,35946641	NA	11	-20848,0833	41718,1812	3881,76446	0
1630	-0,85074914	0,60334436	NA	4,65E-05	-0,00052401	0,40049867	NA	-0,7738147	-0,19560429	NA	NA	-0,00045111	0,35589058	NA	10	-20849,2185	41718,4492	3882,03247	0
199	-1,14142692	NA	-0,01089562	4,31E-05	NA	NA	NA	-3,74999095	0,01316736	0,05794033	NA	NA	NA	NA	7	-20852,3832	41718,7725	3882,35581	0
2149	-1,22822825	NA	NA	4,02E-05	NA	NA	-0,09995451	0,6960215	-0,05271384	NA	NA	NA	NA	-0,49283909	7	-20852,5288	41719,0638	3882,64704	0
2085	-1,22822825	NA	NA	4,02E-05	NA	NA	-0,09995451	0,6894459	NA	NA	NA	NA	NA	-0,49566768	6	-20853,5534	41719,1115	3882,69478	0
197	-1,63084607	NA	NA	4,18E-05	NA	NA	NA	-3,74999095	0,01316736	0,05794033	NA	NA	NA	NA	6	-20853,8583	41719,7213	3883,30462	0
2119	-1,14142692	NA	-0,01089562	4,31E-05	NA	NA	NA	0,6960215	-0,05271384	NA	NA	NA	NA	-0,49283909	7	-20852,8984	41719,8031	3883,38635	0
2055	-1,14142692	NA	-0,01089562	4,31E-05	NA	NA	NA	0,6894459	NA	NA	NA	NA	NA	-0,49566768	6	-20853,9231	41719,8508	3883,43409	0
2218	-6,22256645	0,32760888	NA	NA	-0,00183217	NA	-0,66370241	-1,98285198	NA	0,054624	NA	NA	NA	-0,47270606	8	-20852,0142	41720,0364	3883,61965	0
2117	-1,63084607	NA	NA	4,18E-05	NA	NA	NA	0,6960215	-0,05271384	NA	NA	NA	NA	-0,49283909	6	-20854,3736	41720,7519	3884,33516	0
2053	-1,63084607	NA	NA	4,18E-05	NA	NA	NA	0,6894459	NA	NA	NA	NA	NA	-0,49566768	5	-20855,3983	41720,7998	3884,38312	0
2282	-6,22256645	0,32760888	NA	NA	-0,00183217	NA	-0,66370241	-2,01223022	0,04141198	0,05511629	NA	NA	NA	-0,47466077	9	-20851,4076	41720,8252	3884,40846	0
3802	-8,97566397	0,33138274	NA	NA	-0,0012458	0,28352203	NA	-1,61532922	-0,10289105	0,0570521	NA	-0,00065333	0,49801214	-0,5357095	11	-20849,7447	41721,5039	3885,08721	0
1622	-1,1987332	0,68175498	NA	4,69E-05	NA	0,40581671	NA	-0,7738147	-0,19560429	NA	NA	-0,00045111	0,35589058	NA	9	-20852,1324	41722,2747	3885,85801	0
1624	-1,59179834	0,69737855	0,00852736	4,60E-05	NA	0,40932129	NA	-0,7738147	-0,19560429	NA	NA	-0,00045111	0,35589058	NA	10	-20851,216	41722,4441	3886,02738	0
3738	-8,97566397	0,33138274	NA	NA	-0,0012458	0,28352203	NA	-1,76635397	NA	0,05800383	NA	-0,00051675	0,48731517	-0,53620329	10	-20852,7888	41725,5898	3889,17309	0
1142	-0,67953035	0,68234123	NA	4,46E-05	NA	0,41541375	-0,13795142	-1,05468211	-0,1361791	NA	NA	NA	0,35946641	NA	9	-20854,2441	41726,4981	3890,08142	0
1144	-1,04079715	0,69637088	0,00755047	4,39E-05	NA	0,41816409	-0,13461471	-1,05468211	-0,1361791	NA	NA	NA	0,35946641	NA	10	-20853,5248	41727,0619	3890,64514	0
1120	-1,28042467	0,61751101	0,00965662	4,55E-05	-0,00054807	0,40424516	NA	-1,05468211	-0,1361791	NA	NA	NA	0,35946641	NA	10	-20854,3295	41728,6712	3892,25447	0
1598	-0,06671637	0,57575795	NA	4,34E-05	-0,00071955	0,41051829	-0,17478186	-0,97642003	NA	NA	NA	-0,00019403	0,33432079	NA	10	-20854,445	41728,9021	3892,48537	0
1600	-0,47166397	0,58945108	0,00876766	4,25E-05	-0,00073797	0,4135805	-0,1717464	-0,97642003	NA	NA	NA	-0,00019403	0,33432079	NA	11	-20853,4708	41728,9562	3892,53943	0
1118	-0,85074914	0,60334436	NA	4,65E-05	-0,00052401	0,40049867	NA	-1,05468211	-0,1361791	NA	NA	NA	0,35946641	NA	9	-20855,5053	41729,0207	3892,60393	0
1086	-0,06671637	0,57575795	NA	4,34E-05	-0,00071955	0,41051829	-0,17478186	-1,09054773	NA	NA	NA	NA	0,33971014	NA	9	-20855,88	41729,77	3893,35332	0
1088	-0,47166397	0,58945108	0,00876766	4,25E-05	-0,00073797	0,4135805	-0,1717464	-1,09054773	NA	NA	NA	NA	0,33971014	NA	10	-20854,9059	41729,8239	3893,40716	0
1110	-1,1987332	0,68175498	NA	4,69E-05	NA	0,40581671	NA	-1,05468211	-0,1361791	NA	NA	NA	0,35946641	NA	8	-20858,4192	41732,8464	3896,4297	0
1112	-1,59179834	0,69737855	0,00852736	4,60E-05	NA	0,40932129	NA	-1,05468211	-0,1361791	NA	NA	NA	0,35946641	NA	9	-20857,5028	41733,0156	3896,59884	0
2212	-9,79595589	0,69445228	0,04389505	NA	NA	NA	-0,55018722	-1,98285198	NA	0,054624	NA	NA	NA	-0,47270606	8	-20858,7528	41733,5137	3897,09694	0
2276	-9,79595589	0,69445228	0,04389505	NA	NA	NA	-0,55018722	-2,01223022	0,04141198	0,05511629	NA	NA	NA	-0,47466077	9	-20858,1463	41734,3025	3897,88575	0
1590	-0,67953035	0,68234123	NA	4,46E-05	NA	0,41541375	-0,13795142	-0,97642003	NA	NA	NA	-0,00019403	0,33432079	NA	9	-20859,6316	41737,2731	3900,85638	0
2226	-8,51824578	0,51124721	NA	NA	NA	0,38535796	-0,65213053	-1,98285198	NA	0,054624	NA	NA	NA	-0,47270606	8	-20860,7398	41737,4876	3901,07084	0
2217	-3,98530292	NA	NA	NA	-0,00234183	NA	-0,67333606	-1,98285198	NA	0,054624	NA	NA	NA	-0,47270606	7	-20861,8853	41737,7768	3901,36011	0
1592	-1,04079715	0,69637088	0,00755047	4,39E-05	NA	0,41816409	-0,13461471	-0,97642003	NA	NA	NA	-0,00019403	0,33432079	NA	10	-20858,9123	41737,8368	3901,42011	0
1078	-0,67953035	0,68234123	NA	4,46E-05	NA	0,41541375	-0,13795142	-1,09054773	NA	NA	NA	NA	0,33971014	NA	8	-20861,0667	41738,1413	3901,72455	0
2290	-8,51824578	0,51124721	NA	NA	NA	0,38535796	-0,65213053	-2,01223022	0,04141198	0,05511629	NA	NA	NA	-0,47466077	9	-20860,1332	41738,2764	3901,85965	0
2281	-3,98530292	NA	NA	NA	-0,00234183	NA	-0,67333606	-2,01223022	0,04141198	0,05511629	NA	NA	NA	-0,47466077	8	-20861,2787	41738,5654	3902,1487	0
1080	-1,04079715	0,69637088	0,00755047	4,39E-05	NA	0,41816409	-0,13461471	-1,09054773	NA	NA	NA	NA	0,33971014	NA	9	-20860,3474	41738,7048	3902,28805	0
3801	-7,52922856	NA	NA	NA	-0,00175017	0,32067571	NA	-1,61532922	-0,10289105	0,0570521	NA	-0,00065333	0,49801214	-0,5357095	10	-20859,5702	41739,1525	3902,73577	0
3786	-7,76791863	0,37580763	NA	NA	-0,00127907	NA	NA	-1,61532922	-0,10289105	0,0570521	NA	-0,00065333	0,49801214	-0,5357095	10	-20859,6333	41739,2787	3902,86196	0
1568	-1,28042467	0,61751101	0,00965662	4,55E-05	-0,00054807	0,40424516	NA	-0,97642003	NA	NA	NA	-0,00019403	0,33432079	NA	10	-20859,717	41739,4462	3903,02944	0
1566	-0,85074914	0,60334436	NA	4,65E-05	-0,00052401	0,40049867	NA	-0,97642003	NA	NA	NA	-0,00019403	0,33432079	NA	9	-20860,8928	41739,7956	3903,37889	0
1056	-1,28042467	0,61751101	0,00965662	4,55E-05	-0,00054807	0,40424516	NA	-1,09054773	NA	NA	NA	NA	0,33971014	NA	9	-20861,1521	41740,3141	3903,89738	0
1054	-0,85074914	0,60334436	NA	4,65E-05	-0,00052401	0,40049867	NA	-1,09054773	NA	NA	NA	NA	0,33971014	NA	8	-20862,3279	41740,6638	3904,24706	0
3226	-8,97566397	0,33138274	NA	NA	-0,0012458	0,28352203	NA	-2,06058113	NA	0,05699765	NA	NA	0,49693597	-0,52441365	9	-20862,0335	41742,077	3905,66031	0
3737	-7,52922856	NA	NA	NA	-0,00175017	0,32067571	NA	-1,76635397	NA	0,05800383	NA	-0,00051675	0,48731517	-0,53620329	9	-20862,6143	41743,2386	3906,82186	0
3722	-7,76791863	0,37580763	NA	NA	-0,00127907	NA	NA	-1,76635397	NA	0,05800383	NA	-0,00051675	0,48731517	-0,53620329	9	-20862,6774	41743,3648	3906,94806	0
1558	-1,1987332	0,68175498	NA	4,69E-05	NA	0,40581671	NA	-0,97642003	NA	NA	NA	-0,00019403	0,33432079	NA	8	-20863,8067	41743,6214	3907,20466	0

1560	-1,59179834	0,69737855	0,00852736	4,60E-05	NA	0,40932129	NA	-0,97642003	NA	NA	NA	-0,00019403	0,33432079	NA	9	-20862,8903	41743,7905	3907,37381	0
3290	-8,97566397	0,33138274	NA	NA	-0,0012458	0,28352203	NA	-2,04753199	-0,01846226	0,05678211	NA	NA	0,49927467	-0,52377486	10	-20861,9158	41743,8438	3907,4271	0
1046	-1,1987332	0,68175498	NA	4,69E-05	NA	0,40581671	NA	-1,09054773	NA	NA	NA	NA	0,33971014	NA	7	-20865,2418	41744,4898	3908,07305	0
1048	-1,59179834	0,69737855	0,00852736	4,60E-05	NA	0,40932129	NA	-1,09054773	NA	NA	NA	NA	0,33971014	NA	8	-20864,3254	41744,6587	3908,24198	0
3794	-8,08714822	0,50972296	NA	NA	NA	0,2963004	NA	-1,61532922	-0,10289105	0,0570521	NA	-0,00065333	0,49801214	-0,5357095	10	-20862,441	41744,8943	3908,47753	0
3795	-11,2963041	NA	0,04958538	NA	NA	0,45107925	NA	-1,61532922	-0,10289105	0,0570521	NA	-0,00065333	0,49801214	-0,5357095	10	-20862,8167	41745,6457	3909,22894	0
2722	-7,20552725	0,58574016	NA	NA	NA	NA	-0,62020516	-1,63848397	NA	0,05580518	NA	-0,00060814	NA	-0,48687105	8	-20865,9919	41747,9917	3911,57495	0
2786	-7,20552725	0,58574016	NA	NA	NA	NA	-0,62020516	-1,57337207	-0,04401868	0,05538852	NA	-0,0006675	NA	-0,48613452	9	-20865,4208	41748,8515	3912,43477	0
3730	-8,08714822	0,50972296	NA	NA	NA	0,2963004	NA	-1,76635397	NA	0,05800383	NA	-0,00051675	0,48731517	-0,53620329	9	-20865,4852	41748,9803	3912,56363	0
3731	-11,2963041	NA	0,04958538	NA	NA	0,45107925	NA	-1,76635397	NA	0,05800383	NA	-0,00051675	0,48731517	-0,53620329	9	-20865,8609	41749,7318	3913,31503	0
2739	-8,8387814	NA	0,03010563	NA	NA	0,51295904	-0,60572616	-1,63848397	NA	0,05580518	NA	-0,00060814	NA	-0,48687105	9	-20869,0085	41756,027	3919,61025	0
1646	-0,14195779	0,65649184	NA	4,36E-05	-0,00074998	NA	-0,15445798	-0,7738147	-0,19560429	NA	NA	-0,00045111	0,35589058	NA	10	-20868,2998	41756,6118	3920,19507	0
2803	-8,8387814	NA	0,03010563	NA	NA	0,51295904	-0,60572616	-1,57337207	-0,04401868	0,05538852	NA	-0,0006675	NA	-0,48613452	10	-20868,4374	41756,887	3920,47029	0
1648	-0,45110821	0,66723944	0,00674107	4,29E-05	-0,00076432	NA	-0,15224001	-0,7738147	-0,19560429	NA	NA	-0,00045111	0,35589058	NA	11	-20867,7417	41757,4979	3921,08118	0
3225	-7,52922856	NA	NA	NA	-0,00175017	0,32067571	NA	-2,06058113	NA	0,05699765	NA	NA	0,49693597	-0,52441365	8	-20871,859	41759,726	3923,30931	0
3210	-7,76791863	0,37580763	NA	NA	-0,00127907	NA	NA	-2,06058113	NA	0,05699765	NA	NA	0,49693597	-0,52441365	8	-20871,9221	41759,8522	3923,4355	0
3289	-7,52922856	NA	NA	NA	-0,00175017	0,32067571	NA	-2,04753199	-0,01846226	0,05678211	NA	NA	0,49927467	-0,52377486	9	-20871,7413	41761,4926	3925,07588	0
3274	-7,76791863	0,37580763	NA	NA	-0,00127907	NA	NA	-2,04753199	-0,01846226	0,05678211	NA	NA	0,49927467	-0,52377486	9	-20871,8044	41761,6188	3925,20207	0
3785	-6,18359995	NA	NA	NA	-0,00186372	NA	NA	-1,61532922	-0,10289105	0,0570521	NA	-0,00065333	0,49801214	-0,5357095	9	-20872,4842	41762,9783	3926,5616	0
3778	-10,126072	0,56333515	NA	NA	NA	NA	NA	-1,61532922	-0,10289105	0,0570521	NA	-0,00065333	0,49801214	-0,5357095	9	-20873,2366	41764,4832	3928,0665	0
1614	-0,8455927	0,67951403	NA	4,64E-05	-0,00057321	NA	NA	-0,7738147	-0,19560429	NA	NA	-0,00045111	0,35589058	NA	9	-20873,3203	41764,6505	3928,2338	0
1616	-1,17938783	0,69098467	0,00751019	4,56E-05	-0,00059207	NA	NA	-0,7738147	-0,19560429	NA	NA	-0,00045111	0,35589058	NA	10	-20872,6303	41765,2728	3928,85612	0
3218	-8,08714822	0,50972296	NA	NA	NA	0,2963004	NA	-2,06058113	NA	0,05699765	NA	NA	0,49693597	-0,52441365	8	-20874,7299	41765,4678	3929,05107	0
1638	-0,79586264	0,77122371	NA	4,50E-05	NA	NA	-0,11503973	-0,7738147	-0,19560429	NA	NA	-0,00045111	0,35589058	NA	9	-20873,894	41765,798	3929,38123	0
3708	-5,95790631	0,3741151	0,04613303	NA	-0,00180249	0,39660544	-0,62074526	1,15818376	-0,19528017	NA	NA	-0,00061584	0,46809624	-0,5501339	12	-20871,013	41766,0432	3929,62646	0
3219	-11,2963041	NA	0,04958538	NA	NA	0,45107925	NA	-2,06058113	NA	0,05699765	NA	NA	0,49693597	-0,52441365	8	-20875,1056	41766,2192	3929,80248	0
3721	-6,18359995	NA	NA	NA	-0,00186372	NA	NA	-1,76635397	NA	0,05800383	NA	-0,00051675	0,48731517	-0,53620329	8	-20875,5283	41767,0646	3930,64792	0
1640	-1,05409025	0,78157333	0,00543311	4,44E-05	NA	NA	-0,11273577	-0,7738147	-0,19560429	NA	NA	-0,00045111	0,35589058	NA	10	-20873,5337	41767,0796	3930,66287	0
1134	-0,14195779	0,65649184	NA	4,36E-05	-0,00074998	NA	-0,15445798	-1,05468211	-0,1361791	NA	NA	NA	0,35946641	NA	9	-20874,5866	41767,1833	3930,76653	0
3282	-8,08714822	0,50972296	NA	NA	NA	0,2963004	NA	-2,04753199	-0,01846226	0,05678211	NA	NA	0,49927467	-0,52377486	9	-20874,6122	41767,2344	3930,81764	0
3283	-11,2963041	NA	0,04958538	NA	NA	0,45107925	NA	-2,04753199	-0,01846226	0,05678211	NA	NA	0,49927467	-0,52377486	9	-20874,9879	41767,9858	3931,56905	0
1136	-0,45110821	0,66723944	0,00674107	4,29E-05	-0,00076432	NA	-0,15224001	-1,05468211	-0,1361791	NA	NA	NA	0,35946641	NA	10	-20874,0285	41768,0691	3931,65242	0
3714	-10,126072	0,56333515	NA	NA	NA	NA	NA	-1,76635397	NA	0,05800383	NA	-0,00051675	0,48731517	-0,53620329	8	-20876,2808	41768,5695	3932,15281	0
1606	-1,235734	0,76857786	NA	4,69E-05	NA	NA	NA	-0,7738147	-0,19560429	NA	NA	-0,00045111	0,35589058	NA	8	-20876,7926	41769,5932	3933,17653	0
1608	-1,52051301	0,78035551	0,00624724	4,63E-05	NA	NA	NA	-0,7738147	-0,19560429	NA	NA	-0,00045111	0,35589058	NA	9	-20876,3166	41770,6431	3934,22634	0
2210	-7,20552725	0,58574016	NA	NA	NA	NA	-0,62020516	-1,98285198	NA	0,054624	NA	NA	NA	-0,47270606	7	-20878,7739	41771,5539	3935,13717	0
2274	-7,20552725	0,58574016	NA	NA	NA	NA	-0,62020516	-2,01223022	0,04141198	0,05511629	NA	NA	NA	-0,47466077	8	-20878,1673	41772,3425	3935,92576	0
2737	-6,3315051	NA	NA	NA	NA	0,4838605	-0,6580268	-1,63848397	NA	0,05580518	NA	-0,00060814	NA	-0,48687105	8	-20879,423	41774,8541	3938,43733	0
1102	-0,8455927	0,67951403	NA	4,64E-05	-0,00057321	NA	NA	-1,05468211	-0,1361791	NA	NA	NA	0,35946641	NA	8	-20879,6071	41775,2222	3938,80548	0
2801	-6,3315051	NA	NA	NA	NA	0,4838605	-0,6580268	-1,57337207	-0,04401868	0,05538852	NA	-0,0006675	NA	-0,48613452	9	-20878,852	41775,7139	3939,29715	0
1104	-1,17938783	0,69098467	0,00751019	4,56E-05	-0,00059207	NA	NA	-1,05468211	-0,1361791	NA	NA	NA	0,35946641	NA	9	-20878,9172	41775,8443	3939,42758	0
1126	-0,79586264	0,77122371	NA	4,50E-05	NA	NA	-0,11503973	-1,05468211	-0,1361791	NA	NA	NA	0,35946641	NA	8	-20880,1808	41776,3696	3939,95291	0
1661	0,65451489	NA	NA	3,95E-05	-0,00164357	0,48762971	-0,22170047	-0,7738147	-0,19560429	NA	NA	-0,00045111	0,35589058	NA	10	-20878,5979	41777,208	3940,79126	0
1128	-1,05409025	0,78157333	0,00543311	4,44E-05	NA	NA	-0,11273577	-1,05468211	-0,1361791	NA	NA	NA	0,35946641	NA	9	-20879,8205	41777,651	3941,23433	0
1582	-0,14195779	0,65649184	NA	4,36E-05	-0,00074998	NA	-0,15445798	-0,97642003	NA	NA	NA	-0,00019403	0,33432079	NA	9	-20879,9741	41777,9582	3941,54149	0
1070	-0,14195779	0,65649184	NA	4,36E-05	-0,00074998	NA	-0,15445798	-1,09054773	NA	NA	NA	NA	0,33971014	NA	8	-20881,4092	41778,8264	3942,40966	0
1584	-0,45110821	0,66723944	0,00674107	4,29E-05	-0,00076432	NA	-0,15224001	-0,97642003	NA	NA	NA	-0,00019403	0,33432079	NA	10	-20879,416	41778,8441	3942,42739	0
1663	0,61699723	NA	0,00084798	3,94E-05	-0,00164748	0,4880593	-0,22142621	-0,7738147	-0,19560429	NA	NA	-0,00045111	0,35589058	NA	11	-20878,5886	41779,1918	3942,77511	0
2227	-8,8387814	NA	0,03010563	NA	NA	0,51295904	-0,60572616	-1,98285198	NA	0,054624	NA	NA	NA	-0,47270606	8	-20881,7905	41779,589	3943,17225	0
1072	-0,45110821	0,66723944	0,00674107	4,29E-05	-0,00076432	NA	-0,15224001	-1,09054773	NA	NA	NA	NA	0,33971014	NA	9	-20880,8511	41779,7121	3943,29533	0
1094	-1,235734	0,76857786	NA	4,69E-05	NA	NA	NA	-1,05468211	-0,1361791	NA	NA	NA	0,35946641	NA	7	-20883,0795	41780,1652	3943,74843	0
2291	-8,8387814	NA	0,03010563	NA	NA	0,51295904	-0,60572616	-2,01223022	0,04141198	0,05511629	NA	NA	NA	-0,47466077	9	-20881,1839	41780,3778	3943,96107	0
1096	-1,52051301	0,78035551	0,00624724	4,63E-05	NA	NA	NA	-1,05468211	-0,1361791	NA	NA	NA	0,35946641	NA	8	-20882,6034	41781,2147	3944,79802	0
3209	-6,18359995	NA	NA	NA	-0,00186372	NA	NA	-2,06058113	NA	0,05699765	NA	NA	0,49693597	-0,52441365	7	-20884,7731	41783,5523	3947,13559	0
3202	-10,126072	0,56333515	NA	NA	NA	NA	NA	-2,06058113	NA	0,05699765	NA	NA	0,49693597	-0,52441365	7	-20885,5255	41785,0572	3948,64048	0
3273	-6,18359995	NA	NA	NA	-0,00186372	NA	NA	-2,04753199	-0,01846226	0,05678211	NA	NA	0,49927467	-0,52377486	8	-20884,6553	41785,3187	3948,90193	0
1550	-0,8455927	0,67951403	NA	4,64E-05	-0,00057321	NA	NA	-0,97642003	NA	NA	NA	-0,00019403	0,33432079	NA	8	-20884,9946	41785,9972	3949,58045	0
3196	-5,95790631	0,3741151	0,04613303	NA	-0,00180249	0,39660544	-0,62074526	0,7406183	-0,11449835	NA	NA	NA	0,46966671	-0,53963709	11	-20882,1477	41786,31	3949,8933	0
1552	-1,17938783	0,69098467	0,00751019	4,56E-05	-0,00059207	NA	NA	-0,97642003	NA	NA	NA	-0,00019403	0,33432079	NA	9	-20884,3047	41786,6193	3950,20254	0
3644	-5,95790631	0,3741151	0,04613303	NA	-0,00180249	0,39660544	-0,62074526	0,95852238	NA	NA	NA	-0,00035257	0,44577019	-0,55202249	11	-20882,3466	41786,7077	3950,291	0
3266	-10,126072	0,56333515	NA	NA	NA	NA	NA	-2,04753199	-0,01846226	0,05678211	NA	NA	0,49927467	-0,52377486	8	-20885,4078	41786,8236	3950,40683	0

1038	-0,8455927	0,67951403	NA	4,64E-05	-0,00057321	NA	NA	-1,09054773	NA	NA	NA	NA	0,33971014	NA	7	-20886,4297	41786,8656	3950,44884	0
1574	-0,79586264	0,77122371	NA	4,50E-05	NA	NA	-0,11503973	-0,97642003	NA	NA	NA	-0,00019403	0,33432079	NA	8	-20885,5683	41787,1446	3950,72788	0
1040	-1,17938783	0,69098467	0,00751019	4,56E-05	-0,00059207	NA	NA	-1,09054773	NA	NA	NA	NA	0,33971014	NA	8	-20885,7397	41787,4874	3951,07071	0
1149	0,65451489	NA	NA	3,95E-05	-0,00164357	0,48762971	-0,22170047	-1,05468211	-0,1361791	NA	NA	NA	0,35946641	NA	9	-20884,8847	41787,7794	3951,36272	0
3707	-8,35316771	NA	0,03864408	NA	-0,00234345	0,43617682	-0,64441699	1,15818376	-0,19528017	NA	NA	-0,00061584	0,46809624	-0,5501339	11	-20882,9186	41787,8518	3951,43512	0
1062	-0,79586264	0,77122371	NA	4,50E-05	NA	NA	-0,11503973	-1,09054773	NA	NA	NA	NA	0,33971014	NA	7	-20887,0034	41788,013	3951,59627	0
1576	-1,05409025	0,78157333	0,00543311	4,44E-05	NA	NA	-0,11273577	-0,97642003	NA	NA	NA	-0,00019403	0,33432079	NA	9	-20885,208	41788,426	3952,00929	0
2716	-9,61919161	0,46601352	0,06534536	NA	-0,00131078	0,33938341	NA	-1,63848397	NA	0,05580518	NA	-0,00060814	NA	-0,48687105	10	-20884,3808	41788,7737	3952,35698	0
1064	-1,05409025	0,78157333	0,00543311	4,44E-05	NA	NA	-0,11273577	-1,09054773	NA	NA	NA	NA	0,33971014	NA	8	-20886,6431	41789,2942	3952,87746	0
2780	-9,61919161	0,46601352	0,06534536	NA	-0,00131078	0,33938341	NA	-1,57337207	-0,04401868	0,05538852	NA	-0,0006675	NA	-0,48613452	11	-20883,8097	41789,634	3953,21725	0
1151	0,61699723	NA	0,00084798	3,94E-05	-0,00164748	0,4880593	-0,22142621	-1,05468211	-0,1361791	NA	NA	NA	0,35946641	NA	10	-20884,8754	41789,7631	3953,34634	0
1542	-1,235734	0,76857786	NA	4,69E-05	NA	NA	NA	-0,97642003	NA	NA	NA	-0,00019403	0,33432079	NA	7	-20888,467	41790,9401	3954,52339	0
1030	-1,235734	0,76857786	NA	4,69E-05	NA	NA	NA	-1,09054773	NA	NA	NA	NA	0,33971014	NA	6	-20889,902	41791,8087	3955,39201	0
1544	-1,52051301	0,78035551	0,00624724	4,63E-05	NA	NA	NA	-0,97642003	NA	NA	NA	-0,00019403	0,33432079	NA	8	-20887,9909	41791,9897	3955,57298	0
1032	-1,52051301	0,78035551	0,00624724	4,63E-05	NA	NA	NA	-1,09054773	NA	NA	NA	NA	0,33971014	NA	7	-20889,426	41792,8581	3956,44137	0
3132	-5,95790631	0,3741151	0,04613303	NA	-0,00180249	0,39660544	-0,62074526	0,72537252	NA	NA	NA	NA	0,45346103	-0,54414773	10	-20886,8606	41793,7334	3957,3167	0
3779	-11,7728913	NA	0,04392487	NA	NA	NA	NA	-1,61532922	-0,10289105	0,0570521	NA	-0,00065333	0,49801214	-0,5357095	9	-20888,4391	41794,8882	3958,4715	0
1629	-0,31227401	NA	NA	4,32E-05	-0,00145314	0,47935513	NA	-0,7738147	-0,19560429	NA	NA	-0,00045111	0,35589058	NA	9	-20888,5008	41795,0115	3958,59474	0
638	-0,06671637	0,57575795	NA	4,34E-05	-0,00071955	0,41051829	-0,17478186	-0,66565225	-0,15004816	NA	NA	-0,00048657	NA	NA	10	-20888,066	41796,1442	3959,72752	0
640	-0,47166397	0,58945108	0,00876766	4,25E-05	-0,00073797	0,4135805	-0,1717464	-0,66565225	-0,15004816	NA	NA	-0,00048657	NA	NA	11	-20887,0918	41796,1983	3959,78158	0
1631	-0,39160679	NA	0,00183718	4,30E-05	-0,00146197	0,48040455	NA	-0,7738147	-0,19560429	NA	NA	-0,00045111	0,35589058	NA	10	-20888,4573	41796,9269	3960,51013	0
2225	-6,3315051	NA	NA	NA	NA	0,4838605	-0,6580268	-1,98285198	NA	0,054624	NA	NA	NA	-0,47270606	7	-20892,205	41798,4163	3961,99956	0
1597	0,65451489	NA	NA	3,95E-05	-0,00164357	0,48762971	-0,22170047	-0,97642003	NA	NA	NA	-0,00019403	0,33432079	NA	9	-20890,2722	41798,5544	3962,13768	0
3715	-11,7728913	NA	0,04392487	NA	NA	NA	NA	-1,76635397	NA	0,05800383	NA	-0,00051675	0,48731517	-0,53620329	8	-20891,4833	41798,9745	3962,55782	0
2289	-6,3315051	NA	NA	NA	NA	0,4838605	-0,6580268	-2,01223022	0,04141198	0,05511629	NA	NA	NA	-0,47466077	8	-20891,5985	41799,2049	3962,78815	0
1085	0,65451489	NA	NA	3,95E-05	-0,00164357	0,48762971	-0,22170047	-1,09054773	NA	NA	NA	NA	0,33971014	NA	8	-20891,7073	41799,4226	3963,00585	0
1599	0,61699723	NA	0,00084798	3,94E-05	-0,00164748	0,4880593	-0,22142621	-0,97642003	NA	NA	NA	-0,00019403	0,33432079	NA	10	-20890,2629	41800,538	3964,12131	0
1087	0,61699723	NA	0,00084798	3,94E-05	-0,00164748	0,4880593	-0,22142621	-1,09054773	NA	NA	NA	NA	0,33971014	NA	9	-20891,698	41801,406	3964,98926	0
3692	-9,35928532	0,43489058	0,04364193	NA	-0,00182965	NA	-0,59565606	1,15818376	-0,19528017	NA	NA	-0,00061584	0,46809624	-0,5501339	11	-20890,1163	41802,2471	3965,83042	0
630	-0,67953035	0,68234123	NA	4,46E-05	NA	0,41541375	-0,13795142	-0,66565225	-0,15004816	NA	NA	-0,00048657	NA	NA	9	-20893,2526	41804,5153	3968,09853	0
632	-1,04079715	0,69637088	0,00755047	4,39E-05	NA	0,41816409	-0,13461471	-0,66565225	-0,15004816	NA	NA	-0,00048657	NA	NA	10	-20892,5334	41805,079	3968,66225	0
3793	-9,09446706	NA	NA	NA	NA	0,38722306	NA	-1,61532922	-0,10289105	0,0570521	NA	-0,00065333	0,49801214	-0,5357095	9	-20893,5725	41805,155	3968,73829	0
1117	-0,31227401	NA	NA	4,32E-05	-0,00145314	0,47935513	NA	-1,05468211	-0,1361791	NA	NA	NA	0,35946641	NA	8	-20894,7876	41805,5831	3969,16642	0
608	-1,28042467	0,61751101	0,00965662	4,55E-05	-0,00054807	0,40424516	NA	-0,66565225	-0,15004816	NA	NA	-0,00048657	NA	NA	10	-20893,3381	41806,6883	3970,27158	0
606	-0,85074914	0,60334436	NA	4,65E-05	-0,00052401	0,40049867	NA	-0,66565225	-0,15004816	NA	NA	-0,00048657	NA	NA	9	-20894,5139	41807,0378	3970,62104	0
1119	-0,39160679	NA	0,00183718	4,30E-05	-0,00146197	0,48040455	NA	-1,05468211	-0,1361791	NA	NA	NA	0,35946641	NA	9	-20894,7442	41807,4983	3971,08159	0
3195	-8,35316771	NA	0,03864408	NA	-0,00234345	0,43617682	-0,64441699	0,7406183	-0,11449835	NA	NA	NA	0,46966671	-0,53963709	10	-20894,0534	41808,1189	3971,70218	0
574	-0,06671637	0,57575795	NA	4,34E-05	-0,00071955	0,41051829	-0,17478186	-0,82885018	NA	NA	NA	-0,0002841	NA	NA	9	-20895,077	41808,1639	3971,74718	0
576	-0,47166397	0,58945108	0,00876766	4,25E-05	-0,00073797	0,4135805	-0,1717464	-0,82885018	NA	NA	NA	-0,0002841	NA	NA	10	-20894,1028	41808,2177	3971,80102	0
3643	-8,35316771	NA	0,03864408	NA	-0,00234345	0,43617682	-0,64441699	0,95852238	NA	NA	NA	-0,00035257	0,44577019	-0,55202249	10	-20894,2522	41808,5166	3972,09988	0
3706	-6,98837994	0,26427623	NA	NA	-0,00180413	0,37036432	-0,69230587	1,15818376	-0,19528017	NA	NA	-0,00061584	0,46809624	-0,5501339	11	-20893,3369	41808,6883	3972,2716	0
126	-0,06671637	0,57575795	NA	4,34E-05	-0,00071955	0,41051829	-0,17478186	-0,96744453	-0,08610767	NA	NA	NA	NA	NA	9	-20895,347	41808,7039	3972,28719	0
128	-0,47166397	0,58945108	0,00876766	4,25E-05	-0,00073797	0,4135805	-0,1717464	-0,96744453	-0,08610767	NA	NA	NA	NA	NA	10	-20894,3728	41808,7577	3972,34103	0
3729	-9,09446706	NA	NA	NA	NA	0,38722306	NA	-1,76635397	NA	0,05800383	NA	-0,00051675	0,48731517	-0,53620329	8	-20896,6167	41809,2413	3972,82461	0
598	-1,1987332	0,68175498	NA	4,69E-05	NA	0,40581671	NA	-0,66565225	-0,15004816	NA	NA	-0,00048657	NA	NA	8	-20897,4278	41810,8635	3974,44681	0
600	-1,59179834	0,69737855	0,00852736	4,60E-05	NA	0,40932129	NA	-0,66565225	-0,15004816	NA	NA	-0,00048657	NA	NA	9	-20896,5114	41811,0327	3974,61596	0
62	-0,06671637	0,57575795	NA	4,34E-05	-0,00071955	0,41051829	-0,17478186	-0,99369134	NA	NA	NA	NA	NA	NA	8	-20898,1404	41812,2888	3975,87211	0
2204	-9,61919161	0,46601352	0,06534536	NA	-0,00131078	0,33938341	NA	-1,98285198	NA	0,054624	NA	NA	NA	-0,47270606	9	-20897,1628	41812,3355	3975,91877	0
64	-0,47166397	0,58945108	0,00876766	4,25E-05	-0,00073797	0,4135805	-0,1717464	-0,99369134	NA	NA	NA	NA	NA	NA	9	-20897,1662	41812,3424	3975,92572	0
2268	-9,61919161	0,46601352	0,06534536	NA	-0,00131078	0,33938341	NA	-2,01223022	0,04141198	0,05511629	NA	NA	NA	-0,47466077	10	-20896,5562	41813,1245	3976,7078	0
2708	-13,7669049	0,65178191	0,06462958	NA	NA	0,35119819	NA	-1,63848397	NA	0,05580518	NA	-0,00060814	NA	-0,48687105	9	-20898,2631	41814,5361	3978,11939	0
3700	-10,737769	0,62231984	0,04626548	NA	NA	0,40997724	-0,57836783	1,15818376	-0,19528017	NA	NA	-0,00061584	0,46809624	-0,5501339	11	-20896,3442	41814,703	3978,28629	0
2700	-11,9654714	0,51367836	0,06255183	NA	-0,0013444	NA	NA	-1,63848397	NA	0,05580518	NA	-0,00060814	NA	-0,48687105	9	-20898,4516	41814,9133	3978,49653	0
1788	-5,95790631	0,3741151	0,04613303	NA	-0,00180249	0,39660544	-0,62074526	-3,6371906	-0,09814646	0,05972263	NA	-0,00047389	0,39127251	NA	12	-20895,5881	41815,1934	3978,77664	0
2772	-13,7669049	0,65178191	0,06462958	NA	NA	0,35119819	NA	-1,57337207	-0,04401868	0,05538852	NA	-0,0006675	NA	-0,48613452	10	-20897,692	41815,3962	3978,97943	0
3203	-11,7728913	NA	0,04392487	NA	NA	NA	NA	-2,06058113	NA	0,05699765	NA	NA	0,49693597	-0,52441365	7	-20900,728	41815,4622	3979,04548	0
3131	-8,35316771	NA	0,03864408	NA	-0,00234345	0,43617682	-0,64441699	0,72537252	NA	NA	NA	NA	0,45346103	-0,54414773	9	-20898,7663	41815,5425	3979,1258	0
2764	-11,9654714	0,51367836	0,06255183	NA	-0,0013444	NA	NA	-1,57337207	-0,04401868	0,05538852	NA	-0,0006675	NA	-0,48613452	10	-20897,8806	41815,7733	3979,35658	0
1565	-0,31227401	NA	NA	4,32E-05	-0,00145314	0,47935513	NA	-0,97642003	NA	NA	NA	-0,00019403	0,33432079	NA	8	-20900,1751	41816,3581	3979,94139	0
566	-0,67953035	0,68234123	NA	4,46E-05	NA	0,41541375	-0,13795142	-0,82885018	NA	NA	NA	-0,0002841	NA	NA	8	-20900,2636	41816,5351	3980,11842	0
118	-0,67953035	0,68234123	NA	4,46E-05	NA	0,41541375	-0,13795142	-0,96744453	-0,08610767	NA	NA	NA	NA	NA	8	-20900,5336	41817,0751	3980,65843	0

568	-1,04079715	0,69637088	0,00755047	4,39E-05	NA	0,41816409	-0,13461471	-0,82885018	NA	NA	NA	-0,0002841	NA	NA	9	-20899,5443	41817,0986	3980,68192	0
1053	-0,31227401	NA	NA	4,32E-05	-0,00145314	0,47935513	NA	-1,09054773	NA	NA	NA	NA	0,33971014	NA	7	-20901,6102	41817,2265	3980,80978	0
3267	-11,7728913	NA	0,04392487	NA	NA	NA	NA	-2,04753199	-0,01846226	0,05678211	NA	NA	0,49927467	-0,52377486	8	-20900,6103	41817,2286	3980,81183	0
120	-1,04079715	0,69637088	0,00755047	4,39E-05	NA	0,41816409	-0,13461471	-0,96744453	-0,08610767	NA	NA	NA	NA	NA	9	-20899,8143	41817,6386	3981,22193	0
1567	-0,39160679	NA	0,00183718	4,30E-05	-0,00146197	0,48040455	NA	-0,97642003	NA	NA	NA	-0,00019403	0,33432079	NA	9	-20900,1317	41818,2733	3981,85655	0
544	-1,28042467	0,61751101	0,00965662	4,55E-05	-0,00054807	0,40424516	NA	-0,82885018	NA	NA	NA	-0,0002841	NA	NA	9	-20900,349	41818,708	3982,29125	0
1724	-5,95790631	0,3741151	0,04613303	NA	-0,00180249	0,39660544	-0,62074526	-3,78227707	NA	0,0606519	NA	-0,00034645	0,38158141	NA	11	-20898,4243	41818,8632	3982,44647	0
542	-0,85074914	0,60334436	NA	4,65E-05	-0,00052401	0,40049867	NA	-0,82885018	NA	NA	NA	-0,0002841	NA	NA	8	-20901,5248	41819,0576	3982,64093	0
1055	-0,39160679	NA	0,00183718	4,30E-05	-0,00146197	0,48040455	NA	-1,09054773	NA	NA	NA	NA	0,33971014	NA	8	-20901,5667	41819,1414	3982,72473	0
96	-1,28042467	0,61751101	0,00965662	4,55E-05	-0,00054807	0,40424516	NA	-0,96744453	-0,08610767	NA	NA	NA	NA	NA	9	-20900,619	41819,248	3982,83126	0
3705	-5,46460783	NA	NA	NA	-0,00220571	0,40342229	-0,70298126	1,15818376	-0,19528017	NA	NA	-0,00061584	0,46809624	-0,5501339	10	-20899,6429	41819,298	3982,88132	0
94	-0,85074914	0,60334436	NA	4,65E-05	-0,00052401	0,40049867	NA	-0,96744453	-0,08610767	NA	NA	NA	NA	NA	8	-20901,7948	41819,5977	3983,18094	0
2723	-9,52399274	NA	0,02470059	NA	NA	NA	-0,56950148	-1,63848397	NA	0,05580518	NA	-0,00060814	NA	-0,48687105	8	-20902,0482	41820,1043	3983,6876	0
54	-0,67953035	0,68234123	NA	4,46E-05	NA	0,41541375	-0,13795142	-0,99369134	NA	NA	NA	NA	NA	NA	7	-20903,327	41820,6603	3984,24356	0
2787	-9,52399274	NA	0,02470059	NA	NA	NA	-0,56950148	-1,57337207	-0,04401868	0,05538852	NA	-0,0006675	NA	-0,48613452	9	-20901,4771	41820,9642	3984,54743	0
56	-1,04079715	0,69637088	0,00755047	4,39E-05	NA	0,41816409	-0,13461471	-0,99369134	NA	NA	NA	NA	NA	NA	8	-20902,6078	41821,2236	3984,80684	0
3180	-9,35928532	0,43489058	0,04364193	NA	-0,00182965	NA	-0,59565606	0,7406183	-0,11449835	NA	NA	NA	0,46966671	-0,53963709	10	-20901,251	41822,5142	3986,09748	0
32	-1,28042467	0,61751101	0,00965662	4,55E-05	-0,00054807	0,40424516	NA	-0,99369134	NA	NA	NA	NA	NA	NA	8	-20903,4125	41822,8329	3986,41617	0
534	-1,1987332	0,68175498	NA	4,69E-05	NA	0,40581671	NA	-0,82885018	NA	NA	NA	-0,0002841	NA	NA	7	-20904,4387	41822,8836	3986,46691	0
3628	-9,35928532	0,43489058	0,04364193	NA	-0,00182965	NA	-0,59565606	0,95852238	NA	NA	NA	-0,00035257	0,44577019	-0,55202249	10	-20901,4499	41822,9119	3986,49519	0
536	-1,59179834	0,69737855	0,00852736	4,60E-05	NA	0,40932129	NA	-0,82885018	NA	NA	NA	-0,0002841	NA	NA	8	-20903,5223	41823,0526	3986,63584	0
30	-0,85074914	0,60334436	NA	4,65E-05	-0,00052401	0,40049867	NA	-0,99369134	NA	NA	NA	NA	NA	NA	7	-20904,5883	41823,1828	3986,76607	0
86	-1,1987332	0,68175498	NA	4,69E-05	NA	0,40581671	NA	-0,96744453	-0,08610767	NA	NA	NA	NA	NA	7	-20904,7087	41823,4236	3987,00692	0
88	-1,59179834	0,69737855	0,00852736	4,60E-05	NA	0,40932129	NA	-0,96744453	-0,08610767	NA	NA	NA	NA	NA	8	-20903,7923	41823,5926	3987,17585	0
2715	-11,3659105	NA	0,05744064	NA	-0,00198557	0,38445946	NA	-1,63848397	NA	0,05580518	NA	-0,00060814	NA	-0,48687105	9	-20902,8737	41823,7573	3987,34061	0
2779	-11,3659105	NA	0,05744064	NA	-0,00198557	0,38445946	NA	-1,57337207	-0,04401868	0,05538852	NA	-0,0006675	NA	-0,48613452	10	-20902,3026	41824,6174	3988,20065	0
1212	-5,95790631	0,3741151	0,04613303	NA	-0,00180249	0,39660544	-0,62074526	-3,95454473	NA	0,05999672	NA	NA	0,38996522	NA	10	-20902,7674	41825,5471	3989,13033	0
3217	-9,09446706	NA	NA	NA	NA	0,38722306	NA	-2,06058113	NA	0,05699765	NA	NA	0,49693597	-0,52441365	7	-20905,8614	41825,729	3989,31228	0
1276	-5,95790631	0,3741151	0,04613303	NA	-0,00180249	0,39660544	-0,62074526	-3,92430325	-0,03654601	0,05956532	NA	NA	0,39466847	NA	11	-20902,2963	41826,6072	3990,19049	0
22	-1,1987332	0,68175498	NA	4,69E-05	NA	0,40581671	NA	-0,99369134	NA	NA	NA	NA	NA	NA	6	-20907,5022	41827,009	3990,59228	0
24	-1,59179834	0,69737855	0,00852736	4,60E-05	NA	0,40932129	NA	-0,99369134	NA	NA	NA	NA	NA	NA	7	-20906,5858	41827,1777	3990,76099	0
3281	-9,09446706	NA	NA	NA	NA	0,38722306	NA	-2,04753199	-0,01846226	0,05678211	NA	NA	0,49927467	-0,52377486	8	-20905,7437	41827,4953	3991,07862	0
3194	-6,98837994	0,26427623	NA	NA	-0,00180413	0,37036432	-0,69230587	0,7406183	-0,11449835	NA	NA	NA	0,46966671	-0,53963709	10	-20904,4716	41828,9554	3992,53866	0
3642	-6,98837994	0,26427623	NA	NA	-0,00180413	0,37036432	-0,69230587	0,95852238	NA	NA	NA	-0,00035257	0,44577019	-0,55202249	10	-20904,6705	41829,3531	3992,93637	0
3116	-9,35928532	0,43489058	0,04364193	NA	-0,00182965	NA	-0,59565606	0,72537252	NA	NA	NA	NA	0,45346103	-0,54414773	9	-20905,9639	41829,9378	3993,5211	0
2721	-8,58193391	NA	NA	NA	NA	NA	-0,61393687	-1,63848397	NA	0,05580518	NA	-0,00060814	NA	-0,48687105	7	-20909,0643	41832,1347	3995,718	0
3691	-5,75020926	NA	0,0346068	NA	-0,00247017	NA	-0,61951905	1,15818376	-0,19528017	NA	NA	-0,00061584	0,46809624	-0,5501339	10	-20906,4696	41832,9513	3996,53457	0
2785	-8,58193391	NA	NA	NA	NA	NA	-0,61393687	-1,57337207	-0,04401868	0,05538852	NA	-0,0006675	NA	-0,48613452	8	-20908,4932	41832,9943	3996,5776	0
3188	-10,737769	0,62231984	0,04626548	NA	NA	0,40997724	-0,57836783	0,7406183	-0,11449835	NA	NA	NA	0,46966671	-0,53963709	10	-20907,479	41834,9701	3998,55335	0
3636	-10,737769	0,62231984	0,04626548	NA	NA	0,40997724	-0,57836783	0,95852238	NA	NA	NA	-0,00035257	0,44577019	-0,55202249	10	-20907,6778	41835,3678	3998,95106	0
3130	-6,98837994	0,26427623	NA	NA	-0,00180413	0,37036432	-0,69230587	0,72537252	NA	NA	NA	NA	0,45346103	-0,54414773	9	-20909,1845	41836,379	3999,96228	0
1787	-8,35316771	NA	0,03864408	NA	-0,00234345	0,43617682	-0,64441699	-3,6371906	-0,09814646	0,05972263	NA	-0,00047389	0,39127251	NA	11	-20907,4937	41837,002	4000,5853	0
2196	-13,7669049	0,65178191	0,06462958	NA	NA	0,35119819	NA	-1,98285198	NA	0,054624	NA	NA	NA	-0,47270606	8	-20911,0451	41838,0981	4001,68139	0
2188	-11,9654714	0,51367836	0,06255183	NA	-0,0013444	NA	NA	-1,98285198	NA	0,054624	NA	NA	NA	-0,47270606	8	-20911,2336	41838,4753	4002,05854	0
2260	-13,7669049	0,65178191	0,06462958	NA	NA	0,35119819	NA	-2,01223022	0,04141198	0,05511629	NA	NA	NA	-0,47466077	9	-20910,4385	41838,8869	4002,4702	0
2252	-11,9654714	0,51367836	0,06255183	NA	-0,0013444	NA	NA	-2,01223022	0,04141198	0,05511629	NA	NA	NA	-0,47466077	9	-20910,6271	41839,2641	4002,84735	0
3193	-5,46460783	NA	NA	NA	-0,00220571	0,40342229	-0,70298126	0,7406183	-0,11449835	NA	NA	NA	0,46966671	-0,53963709	9	-20910,7777	41839,5653	4003,14861	0
3641	-5,46460783	NA	NA	NA	-0,00220571	0,40342229	-0,70298126	0,95852238	NA	NA	NA	-0,00035257	0,44577019	-0,55202249	9	-20910,9765	41839,963	4003,54631	0
3690	-6,22256645	0,32760888	NA	NA	-0,00183217	NA	-0,66370241	1,15818376	-0,19528017	NA	NA	-0,00061584	0,46809624	-0,5501339	10	-20910,0181	41840,0483	4003,63163	0
1723	-8,35316771	NA	0,03864408	NA	-0,00234345	0,43617682	-0,64441699	-3,78227707	NA	0,0606519	NA	-0,00034645	0,38158141	NA	10	-20910,33	41840,6721	4004,25535	0
3777	-15,360872	NA	NA	NA	NA	NA	NA	-1,61532922	-0,10289105	0,0570521	NA	-0,00065333	0,49801214	-0,5357095	8	-20912,7147	41841,4373	4005,02057	0
3124	-10,737769	0,62231984	0,04626548	NA	NA	0,40997724	-0,57836783	0,72537252	NA	NA	NA	NA	0,45346103	-0,54414773	9	-20912,1919	41842,3937	4005,97697	0
2692	-12,0135881	0,70944129	0,06178489	NA	NA	NA	NA	-1,63848397	NA	0,05580518	NA	-0,00060814	NA	-0,48687105	8	-20913,3501	41842,7081	4006,29137	0
2756	-12,0135881	0,70944129	0,06178489	NA	NA	NA	NA	-1,57337207	-0,04401868	0,05538852	NA	-0,0006675	NA	-0,48613452	9	-20912,779	41843,5679	4007,15119	0
2211	-9,52399274	NA	0,02470059	NA	NA	NA	-0,56950148	-1,98285198	NA	0,054624	NA	NA	NA	-0,47270606	7	-20914,8302	41843,6666	4007,24983	0
2275	-9,52399274	NA	0,02470059	NA	NA	NA	-0,56950148	-2,01223022	0,04141198	0,05511629	NA	NA	NA	-0,47466077	8	-20914,2236	41844,4551	4008,03842	0
622	-0,14195779	0,65649184	NA	4,36E-05	-0,00074998	NA	-0,15445798	-0,66565225	-0,15004816	NA	NA	-0,00048657	NA	NA	9	-20913,5952	41845,2004	4008,78364	0
3713	-15,360872	NA	NA	NA	NA	NA	NA	-1,76635397	NA	0,05800383	NA	-0,00051675	0,48731517	-0,53620329	7	-20915,7588	41845,5238	4009,10711	0
624	-0,45110821	0,66723944	0,00674107	4,29E-05	-0,00076432	NA	-0,15224001	-0,66565225	-0,15004816	NA	NA	-0,00048657	NA	NA	10	-20913,037	41846,0863	4009,66953	0
1645	0,64576877	NA	NA	3,88E-05	-0,00183092	NA	-0,20438761	-0,7738147	-0,19560429	NA	NA	-0,00045111	0,35589058	NA	9	-20914,2027	41846,4154	4009,99869	0
1653	-0,84339357	NA	NA	4,07E-05	NA	0,55505772	-0,13640315	-0,7738147	-0,19560429	NA	NA	-0,00045111	0,35589058	NA	9	-20914,4609	41846,9318	4010,51512	0

3129	-5,46460783	NA	NA	NA	-0,00220571	0,40342229	-0,70298126	0,72537252	NA	NA	NA	NA	0,45346103	-0,54414773	8	-20915,4906	41846,9892	4010,57245	0
2203	-11,3659105	NA	0,05744064	NA	-0,00198557	0,38445946	NA	-1,98285198	NA	0,054624	NA	NA	NA	-0,47270606	8	-20915,6557	41847,3193	4010,90262	0
1211	-8,35316771	NA	0,03864408	NA	-0,00234345	0,43617682	-0,64441699	-3,95454473	NA	0,05999672	NA	NA	0,38996522	NA	9	-20914,6731	41847,3562	4010,93943	0
1655	-0,50593593	NA	-0,00700816	4,14E-05	NA	0,55021643	-0,14061875	-0,7738147	-0,19560429	NA	NA	-0,00045111	0,35589058	NA	10	-20913,8279	41847,6679	4011,25121	0
2267	-11,3659105	NA	0,05744064	NA	-0,00198557	0,38445946	NA	-2,01223022	0,04141198	0,05511629	NA	NA	NA	-0,47466077	9	-20915,0491	41848,1081	4011,69143	0
1647	0,74969745	NA	-0,00245756	3,91E-05	-0,00181949	NA	-0,20513804	-0,7738147	-0,19560429	NA	NA	-0,00045111	0,35589058	NA	10	-20914,1272	41848,2665	4011,84975	0
1275	-8,35316771	NA	0,03864408	NA	-0,00234345	0,43617682	-0,64441699	-3,92430325	-0,03654601	0,05956532	NA	NA	0,39466847	NA	10	-20914,202	41848,4161	4011,99936	0
1772	-9,35928532	0,43489058	0,04364193	NA	-0,00182965	NA	-0,59565606	-3,6371906	-0,09814646	0,05972263	NA	-0,00047389	0,39127251	NA	11	-20914,6914	41851,3973	4014,9806	0
1621	-1,36352414	NA	NA	4,29E-05	NA	0,54467715	NA	-0,7738147	-0,19560429	NA	NA	-0,00045111	0,35589058	NA	8	-20918,0013	41852,0106	4015,59389	0
1623	-1,08960867	NA	-0,00576757	4,36E-05	NA	0,53973144	NA	-0,7738147	-0,19560429	NA	NA	-0,00045111	0,35589058	NA	9	-20917,569	41853,148	4016,7313	0
3179	-5,75020926	NA	0,0346068	NA	-0,00247017	NA	-0,61951905	0,7406183	-0,11449835	NA	NA	NA	0,46966671	-0,53963709	9	-20917,6043	41853,2186	4016,80185	0
590	-0,8455927	0,67951403	NA	4,64E-05	-0,00057321	NA	NA	-0,66565225	-0,15004816	NA	NA	-0,00048657	NA	NA	8	-20918,6157	41853,2393	4016,8226	0
3684	-9,79595589	0,69445228	0,04389505	NA	NA	NA	-0,55018722	1,15818376	-0,19528017	NA	NA	-0,00061584	0,46809624	-0,5501339	10	-20916,7567	41853,5256	4017,10892	0
3627	-5,75020926	NA	0,0346068	NA	-0,00247017	NA	-0,61951905	0,95852238	NA	NA	NA	-0,00035257	0,44577019	-0,55202249	9	-20917,8032	41853,6163	4017,19955	0
592	-1,17938783	0,69098467	0,00751019	4,56E-05	-0,00059207	NA	NA	-0,66565225	-0,15004816	NA	NA	-0,00048657	NA	NA	9	-20917,9257	41853,8614	4017,44469	0
614	-0,79586264	0,77122371	NA	4,50E-05	NA	NA	-0,11503973	-0,66565225	-0,15004816	NA	NA	-0,00048657	NA	NA	8	-20919,1894	41854,3867	4017,97002	0
1708	-9,35928532	0,43489058	0,04364193	NA	-0,00182965	NA	-0,59565606	-3,78227707	NA	0,0606519	NA	-0,00034645	0,38158141	NA	10	-20917,5276	41855,0674	4018,65065	0
616	-1,05409025	0,78157333	0,00543311	4,44E-05	NA	NA	-0,11273577	-0,66565225	-0,15004816	NA	NA	-0,00048657	NA	NA	9	-20918,8291	41855,6682	4019,25144	0
2209	-8,58193391	NA	NA	NA	NA	NA	-0,61393687	-1,98285198	NA	0,054624	NA	NA	NA	-0,47270606	6	-20921,8463	41855,6972	4019,28045	0
2273	-8,58193391	NA	NA	NA	NA	NA	-0,61393687	-2,01223022	0,04141198	0,05511629	NA	NA	NA	-0,47466077	7	-20921,2397	41856,4855	4020,06881	0
1133	0,64576877	NA	NA	3,88E-05	-0,00183092	NA	-0,20438761	-1,05468211	-0,1361791	NA	NA	NA	0,35946641	NA	8	-20920,4896	41856,9871	4020,57037	0
558	-0,14195779	0,65649184	NA	4,36E-05	-0,00074998	NA	-0,15445798	-0,82885018	-0,1361791	NA	NA	-0,0002841	NA	NA	8	-20920,6061	41857,2202	4020,80353	0
3698	-8,51824578	0,51124721	NA	NA	NA	0,38535796	-0,65213053	1,15818376	-0,19528017	NA	NA	-0,00061584	0,46809624	-0,5501339	10	-20918,7437	41857,4995	4021,08282	0
1141	-0,84339357	NA	NA	4,07E-05	NA	0,55505772	-0,13640315	-1,05468211	-0,1361791	NA	NA	NA	0,35946641	NA	8	-20920,7478	41857,5035	4021,0868	0
110	-0,14195779	0,65649184	NA	4,36E-05	-0,00074998	NA	-0,15445798	-0,96744453	-0,08610767	NA	NA	NA	NA	NA	8	-20920,8761	41857,7603	4021,34354	0
3689	-3,98530292	NA	NA	NA	-0,00234183	NA	-0,67333606	1,15818376	-0,19528017	NA	NA	-0,00061584	0,46809624	-0,5501339	9	-20919,8892	41857,7884	4021,37165	0
1786	-6,98837994	0,26427623	NA	NA	-0,00180413	0,37036432	-0,69230587	-3,6371906	-0,09814646	0,05972263	NA	-0,00047389	0,39127251	NA	11	-20917,912	41857,8385	4021,42178	0
560	-0,45110821	0,66723944	0,00674107	4,29E-05	-0,00076432	NA	-0,15224001	-0,82885018	NA	NA	NA	-0,0002841	NA	NA	9	-20920,048	41858,1059	4021,6892	0
582	-1,235734	0,76857786	NA	4,69E-05	NA	NA	NA	-0,66565225	-0,15004816	NA	NA	-0,00048657	NA	NA	7	-20922,088	41858,1823	4021,76554	0
1143	-0,50593593	NA	-0,00700816	4,14E-05	NA	0,55021643	-0,14061875	-1,05468211	-0,1361791	NA	NA	NA	0,35946641	NA	9	-20920,1147	41858,2394	4021,82267	0
2699	-9,45532716	NA	0,05329757	NA	-0,00210061	NA	NA	-1,63848397	NA	0,05580518	NA	-0,00060814	NA	-0,48687105	8	-20921,2292	41858,4664	4022,04969	0
112	-0,45110821	0,66723944	0,00674107	4,29E-05	-0,00076432	NA	-0,15224001	-0,96744453	-0,08610767	NA	NA	NA	NA	NA	9	-20920,318	41858,6459	4022,22921	0
1135	0,74969745	NA	-0,00245756	3,91E-05	-0,00181949	NA	-0,20513804	-1,05468211	-0,1361791	NA	NA	NA	0,35946641	NA	9	-20920,414	41858,8379	4022,42121	0
584	-1,52051301	0,78035551	0,00624724	4,63E-05	NA	NA	NA	-0,66565225	-0,15004816	NA	NA	-0,00048657	NA	NA	8	-20921,6119	41859,2318	4022,81513	0
2763	-9,45532716	NA	0,05329757	NA	-0,00210061	NA	NA	-1,57337207	-0,04401868	0,05538852	NA	-0,0006675	NA	-0,48613452	9	-20920,6581	41859,3262	4022,90952	0
3178	-6,22256645	0,32760888	NA	NA	-0,00183217	NA	-0,66370241	0,7406183	-0,11449835	NA	NA	NA	0,46966671	-0,53963709	9	-20921,1528	41860,3156	4023,89891	0
3115	-5,75020926	NA	0,0346068	NA	-0,00247017	NA	-0,61951905	0,72537252	NA	NA	NA	NA	0,45346103	-0,54414773	8	-20922,3172	41860,6424	4024,22569	0
3626	-6,22256645	0,32760888	NA	NA	-0,00183217	NA	-0,66370241	0,95852238	NA	NA	NA	-0,00035257	0,44577019	-0,55202249	9	-20921,3517	41860,7133	4024,29661	0
1613	-0,28011525	NA	NA	4,24E-05	-0,0016491	NA	NA	-0,7738147	-0,19560429	NA	NA	-0,00045111	0,35589058	NA	8	-20922,493	41860,994	4024,57731	0
46	-0,14195779	0,65649184	NA	4,36E-05	-0,00074998	NA	-0,15445798	-0,99369134	NA	NA	NA	NA	NA	NA	7	-20923,6696	41861,3454	4024,92867	0
1722	-6,98837994	0,26427623	NA	NA	-0,00180413	0,37036432	-0,69230587	-3,78227707	NA	0,0606519	NA	-0,00034645	0,38158141	NA	10	-20920,7482	41861,5086	4025,09183	0
1196	-9,35928532	0,43489058	0,04364193	NA	-0,00182965	NA	-0,59565606	-3,95454473	NA	0,05999672	NA	NA	0,38996522	NA	9	-20921,8708	41861,7515	4025,33473	0
3201	-15,360872	NA	NA	NA	NA	NA	NA	-2,06058113	NA	0,05699765	NA	NA	0,49693597	-0,52441365	6	-20925,0035	41862,0117	4025,595	0
48	-0,45110821	0,66723944	0,00674107	4,29E-05	-0,00076432	NA	-0,15224001	-0,99369134	NA	NA	NA	NA	NA	NA	8	-20923,1114	41862,2308	4025,81412	0
1109	-1,36352414	NA	NA	4,29E-05	NA	0,54467715	NA	-1,05468211	-0,1361791	NA	NA	NA	0,35946641	NA	7	-20924,2882	41862,5825	4026,16579	0
1260	-9,35928532	0,43489058	0,04364193	NA	-0,00182965	NA	-0,59565606	-3,92430325	-0,03654601	0,05956532	NA	NA	0,39466847	NA	10	-20921,3996	41862,8114	4026,39467	0
1615	-0,2131598	NA	-0,0015413	4,25E-05	-0,00164139	NA	NA	-0,7738147	-0,19560429	NA	NA	-0,00045111	0,35589058	NA	9	-20922,4633	41862,9366	4026,51991	0
1111	-1,08960867	NA	-0,00576757	4,36E-05	NA	0,53973144	NA	-1,05468211	-0,1361791	NA	NA	NA	0,35946641	NA	8	-20923,8559	41863,7197	4027,30298	0
3265	-15,360872	NA	NA	NA	NA	NA	NA	-2,04753199	-0,01846226	0,05678211	NA	NA	0,49927467	-0,52377486	7	-20924,8858	41863,7779	4027,36113	0
1780	-10,737769	0,62231984	0,04626548	NA	NA	0,40997724	-0,57836783	-3,6371906	-0,09814646	0,05972263	NA	-0,00047389	0,39127251	NA	11	-20920,9193	41863,8532	4027,43647	0
526	-0,8455927	0,67951403	NA	4,64E-05	-0,00057321	NA	NA	-0,82885018	NA	NA	NA	-0,0002841	NA	NA	7	-20925,6266	41865,2594	4028,8427	0
637	0,65451489	NA	NA	3,95E-05	-0,00164357	0,48762971	-0,22170047	-0,66565225	-0,15004816	NA	NA	-0,00048657	NA	NA	9	-20923,8933	41865,7966	4029,37983	0
78	-0,8455927	0,67951403	NA	4,64E-05	-0,00057321	NA	NA	-0,96744453	-0,08610767	NA	NA	NA	NA	NA	7	-20925,8966	41865,7994	4029,38271	0
528	-1,17938783	0,69098467	0,00751019	4,56E-05	-0,00059207	NA	NA	-0,82885018	NA	NA	NA	-0,0002841	NA	NA	8	-20924,9367	41865,8813	4029,46457	0
2180	-12,0135881	0,70944129	0,06178489	NA	NA	NA	NA	-1,98285198	NA	0,054624	NA	NA	NA	-0,47270606	7	-20926,1321	41866,2703	4029,8536	0
550	-0,79586264	0,77122371	NA	4,50E-05	NA	NA	-0,11503973	-0,82885018	NA	NA	NA	-0,0002841	NA	NA	7	-20926,2003	41866,4069	4029,99013	0
80	-1,17938783	0,69098467	0,00751019	4,56E-05	-0,00059207	NA	NA	-0,96744453	-0,08610767	NA	NA	NA	NA	NA	8	-20925,2067	41866,4213	4030,00458	0
102	-0,79586264	0,77122371	NA	4,50E-05	NA	NA	-0,11503973	-0,96744453	-0,08610767	NA	NA	NA	NA	NA	7	-20926,4703	41866,9469	4030,53014	0
2244	-12,0135881	0,70944129	0,06178489	NA	NA	NA	NA	-2,01223022	0,04141198	0,05511629	NA	NA	NA	-0,47466077	8	-20925,5255	41867,0589	4030,64219	0
1716	-10,737769	0,62231984	0,04626548	NA	NA	0,40997724	-0,57836783	-3,78227707	NA	0,0606519	NA	-0,00034645	0,38158141	NA	10	-20923,7555	41867,5232	4031,10652	0
552	-1,05409025	0,78157333	0,00543311	4,44E-05	NA	NA	-0,11273577	-0,82885018	NA	NA	NA	-0,0002841	NA	NA	8	-20925,84	41867,688	4031,27132	0

3114	-6,22256645	0,32760888	NA	NA	-0,00183217	NA	-0,66370241	0,72537252	NA	NA	NA	NA	0,45346103	-0,54414773	8	-20925,8658	41867,7395	4031,32275	0
1581	0,64576877	NA	NA	3,88E-05	-0,00183092	NA	-0,20438761	-0,97642003	NA	NA	NA	-0,00019403	0,33432079	NA	8	-20925,877	41867,7621	4031,34533	0
639	0,61699723	NA	0,00084798	3,94E-05	-0,00164748	0,4880593	-0,22142621	-0,66565225	-0,15004816	NA	NA	-0,00048657	NA	NA	10	-20923,884	41867,7802	4031,36345	0
1210	-6,98837994	0,26427623	NA	NA	-0,00180413	0,37036432	-0,69230587	-3,95454473	NA	0,05999672	NA	NA	0,38996522	NA	9	-20925,0913	41868,1926	4031,77591	0
104	-1,05409025	0,78157333	0,00543311	4,44E-05	NA	NA	-0,11273577	-0,96744453	-0,08610767	NA	NA	NA	NA	NA	8	-20926,11	41868,2281	4031,81133	0
1589	-0,84339357	NA	NA	4,07E-05	NA	0,55505772	-0,13640315	-0,97642003	NA	NA	NA	-0,00019403	0,33432079	NA	8	-20926,1353	41868,2785	4031,86177	0
1785	-5,46460783	NA	NA	NA	-0,00220571	0,40342229	-0,70298126	-3,6371906	-0,09814646	0,05972263	NA	-0,00047389	0,39127251	NA	10	-20924,218	41868,4482	4032,0315	0
1069	0,64576877	NA	NA	3,88E-05	-0,00183092	NA	-0,20438761	-1,09054773	NA	NA	NA	NA	0,33971014	NA	7	-20927,3121	41868,6304	4032,21373	0
1591	-0,50593593	NA	-0,00700816	4,14E-05	NA	0,55021643	-0,14061875	-0,97642003	NA	NA	NA	-0,00019403	0,33432079	NA	9	-20925,5022	41869,0144	4032,59764	0
1077	-0,84339357	NA	NA	4,07E-05	NA	0,55505772	-0,13640315	-1,09054773	NA	NA	NA	NA	0,33971014	NA	7	-20927,5703	41869,1469	4032,73016	0
1274	-6,98837994	0,26427623	NA	NA	-0,00180413	0,37036432	-0,69230587	-3,92430325	-0,03654601	0,05956532	NA	NA	0,39466847	NA	10	-20924,6202	41869,2526	4032,83585	0
14	-0,8455927	0,67951403	NA	4,64E-05	-0,00057321	NA	NA	-0,99369134	NA	NA	NA	NA	NA	NA	6	-20928,6901	41869,3848	4032,96807	0
1583	0,74969745	NA	-0,00245756	3,91E-05	-0,00181949	NA	-0,20513804	-0,97642003	NA	NA	NA	-0,00019403	0,33432079	NA	9	-20925,8015	41869,6129	4033,19618	0
1079	-0,50593593	NA	-0,00700816	4,14E-05	NA	0,55021643	-0,14061875	-1,09054773	NA	NA	NA	NA	0,33971014	NA	8	-20926,9373	41869,8825	4033,46581	0
16	-1,17938783	0,69098467	0,00751019	4,56E-05	-0,00059207	NA	NA	-0,99369134	NA	NA	NA	NA	NA	NA	7	-20928,0001	41870,0064	4033,58972	0
518	-1,235734	0,76857786	NA	4,69E-05	NA	NA	NA	-0,82885018	NA	NA	NA	-0,0002841	NA	NA	6	-20929,099	41870,2026	4033,78587	0
1071	0,74969745	NA	-0,00245756	3,91E-05	-0,00181949	NA	-0,20513804	-1,09054773	NA	NA	NA	NA	0,33971014	NA	8	-20927,2366	41870,4811	4034,06435	0
38	-0,79586264	0,77122371	NA	4,50E-05	NA	NA	-0,11503973	-0,99369134	NA	NA	NA	NA	NA	NA	6	-20929,2638	41870,5322	4034,1155	0
70	-1,235734	0,76857786	NA	4,69E-05	NA	NA	NA	-0,96744453	-0,08610767	NA	NA	NA	NA	NA	6	-20929,369	41870,7426	4034,32588	0
520	-1,52051301	0,78035551	0,00624724	4,63E-05	NA	NA	NA	-0,82885018	NA	NA	NA	-0,0002841	NA	NA	7	-20928,6229	41871,252	4034,83523	0
1101	-0,28011525	NA	NA	4,24E-05	-0,0016491	NA	NA	-1,05468211	-0,1361791	NA	NA	NA	0,35946641	NA	7	-20928,7799	41871,5659	4035,14921	0
72	-1,52051301	0,78035551	0,00624724	4,63E-05	NA	NA	NA	-0,96744453	-0,08610767	NA	NA	NA	NA	NA	7	-20928,8929	41871,792	4035,37524	0
40	-1,05409025	0,78157333	0,00543311	4,44E-05	NA	NA	-0,11273577	-0,99369134	NA	NA	NA	NA	NA	NA	7	-20928,9035	41871,8132	4035,39647	0
1721	-5,46460783	NA	NA	NA	-0,00220571	0,40342229	-0,70298126	-3,78227707	NA	0,0606519	NA	-0,00034645	0,38158141	NA	9	-20927,0543	41872,1185	4035,70178	0
1557	-1,36352414	NA	NA	4,29E-05	NA	0,54467715	NA	-0,97642003	NA	NA	NA	-0,00019403	0,33432079	NA	7	-20929,6756	41873,3575	4036,94076	0
1103	-0,2131598	NA	-0,0015413	4,25E-05	-0,00164139	NA	NA	-1,05468211	-0,1361791	NA	NA	NA	0,35946641	NA	8	-20928,7502	41873,5083	4037,09159	0
3172	-9,79595589	0,69445228	0,04389505	NA	NA	NA	-0,55018722	0,7406183	-0,11449835	NA	NA	NA	0,46966671	-0,53963709	9	-20927,8915	41873,7929	4037,3762	0
3620	-9,79595589	0,69445228	0,04389505	NA	NA	NA	-0,55018722	0,95852238	NA	NA	NA	-0,00035257	0,44577019	-0,55202249	9	-20928,0903	41874,1906	4037,7739	0
1204	-10,737769	0,62231984	0,04626548	NA	NA	0,40997724	-0,57836783	-3,95454473	NA	0,05999672	NA	NA	0,38996522	NA	9	-20928,0987	41874,2073	4037,7906	0
1045	-1,36352414	NA	NA	4,29E-05	NA	0,54467715	NA	-1,09054773	NA	NA	NA	NA	0,33971014	NA	6	-20931,1107	41874,2261	4037,80937	0
6	-1,235734	0,76857786	NA	4,69E-05	NA	NA	NA	-0,99369134	NA	NA	NA	NA	NA	NA	5	-20932,1624	41874,3282	4037,91146	0
1559	-1,08960867	NA	-0,00576757	4,36E-05	NA	0,53973144	NA	-0,97642003	NA	NA	NA	-0,00019403	0,33432079	NA	8	-20929,2434	41874,4947	4038,07795	0
1268	-10,737769	0,62231984	0,04626548	NA	NA	0,40997724	-0,57836783	-3,92430325	-0,03654601	0,05956532	NA	NA	0,39466847	NA	10	-20927,6275	41875,2673	4038,85054	0
1047	-1,08960867	NA	-0,00576757	4,36E-05	NA	0,53973144	NA	-1,09054773	NA	NA	NA	NA	0,33971014	NA	7	-20930,6784	41875,3631	4038,94634	0
8	-1,52051301	0,78035551	0,00624724	4,63E-05	NA	NA	NA	-0,99369134	NA	NA	NA	NA	NA	NA	6	-20931,6863	41875,3773	4038,9606	0
3186	-8,51824578	0,51124721	NA	NA	NA	0,38535796	-0,65213053	0,7406183	-0,11449835	NA	NA	NA	0,46966671	-0,53963709	9	-20929,8784	41877,7668	4041,35011	0
573	0,65451489	NA	NA	3,95E-05	-0,00164357	0,48762971	-0,22170047	-0,82885018	NA	NA	NA	-0,0002841	NA	NA	8	-20930,9042	41877,8164	4041,39972	0
3177	-3,98530292	NA	NA	NA	-0,00234183	NA	-0,67333606	0,7406183	-0,11449835	NA	NA	NA	0,46966671	-0,53963709	8	-20931,024	41878,0559	4041,63915	0
3634	-8,51824578	0,51124721	NA	NA	NA	0,38535796	-0,65213053	0,95852238	NA	NA	NA	-0,00035257	0,44577019	-0,55202249	9	-20930,0773	41878,1645	4041,74781	0
125	0,65451489	NA	NA	3,95E-05	-0,00164357	0,48762971	-0,22170047	-0,96744453	-0,08610767	NA	NA	NA	NA	NA	8	-20931,1742	41878,3564	4041,93973	0
3625	-3,98530292	NA	NA	NA	-0,00234183	NA	-0,67333606	0,95852238	NA	NA	NA	-0,00035257	0,44577019	-0,55202249	8	-20931,2228	41878,4536	4042,03686	0
1209	-5,46460783	NA	NA	NA	-0,00220571	0,40342229	-0,70298126	-3,95454473	NA	0,05999672	NA	NA	0,38996522	NA	8	-20931,3974	41878,8028	4042,38608	0
575	0,61699723	NA	0,00084798	3,94E-05	-0,00164748	0,4880593	-0,22142621	-0,82885018	NA	NA	NA	-0,0002841	NA	NA	9	-20930,8949	41879,7998	4043,38312	0
1273	-5,46460783	NA	NA	NA	-0,00220571	0,40342229	-0,70298126	-3,92430325	-0,03654601	0,05956532	NA	NA	0,39466847	NA	9	-20930,9263	41879,8625	4043,44579	0
127	0,61699723	NA	0,00084798	3,94E-05	-0,00164748	0,4880593	-0,22142621	-0,96744453	-0,08610767	NA	NA	NA	NA	NA	9	-20931,1649	41880,3398	4043,92313	0
3108	-9,79595589	0,69445228	0,04389505	NA	NA	NA	-0,55018722	0,72537252	NA	NA	NA	NA	0,45346103	-0,54414773	8	-20932,6044	41881,2168	4044,80004	0
61	0,65451489	NA	NA	3,95E-05	-0,00164357	0,48762971	-0,22170047	-0,99369134	NA	NA	NA	NA	NA	NA	7	-20933,9677	41881,9416	4045,52486	0
2187	-9,45532716	NA	0,05329757	NA	-0,00210061	NA	NA	-1,98285198	NA	0,054624	NA	NA	NA	-0,47270606	7	-20934,0112	41882,0286	4045,61192	0
1771	-5,75020926	NA	0,0346068	NA	-0,00247017	NA	-0,61951905	-3,6371906	-0,09814646	0,05972263	NA	-0,00047389	0,39127251	NA	10	-20931,0447	41882,1015	4045,68475	0
1549	-0,28011525	NA	NA	4,24E-05	-0,0016491	NA	NA	-0,97642003	NA	NA	NA	-0,00019403	0,33432079	NA	7	-20934,1674	41882,3409	4045,92418	0
2251	-9,45532716	NA	0,05329757	NA	-0,00210061	NA	NA	-2,01223022	0,04141198	0,05511629	NA	NA	NA	-0,47466077	8	-20933,4046	41882,8172	4046,40051	0
1037	-0,28011525	NA	NA	4,24E-05	-0,0016491	NA	NA	-1,09054773	NA	NA	NA	NA	0,33971014	NA	6	-20935,6024	41883,2095	4046,79279	0
605	-0,31227401	NA	NA	4,32E-05	-0,00145314	0,47935513	NA	-0,66565225	-0,15004816	NA	NA	-0,00048657	NA	NA	8	-20933,7961	41883,6003	4047,18353	0
63	0,61699723	NA	0,00084798	3,94E-05	-0,00164748	0,4880593	-0,22142621	-0,99369134	NA	NA	NA	NA	NA	NA	8	-20933,9584	41883,9248	4047,50804	0
2714	-8,97566397	0,33138274	NA	NA	-0,0012458	0,28352203	NA	-1,63848397	NA	0,05580518	NA	-0,00060814	NA	-0,48687105	9	-20933,0834	41884,1768	4047,76005	0
1551	-0,2131598	NA	-0,0015413	4,25E-05	-0,00164139	NA	NA	-0,97642003	NA	NA	NA	-0,00019403	0,33432079	NA	8	-20934,1377	41884,2833	4047,86656	0
2778	-8,97566397	0,33138274	NA	NA	-0,0012458	0,28352203	NA	-1,57337207	-0,04401868	0,05538852	NA	-0,0006675	NA	-0,48613452	10	-20932,5123	41885,0368	4048,6201	0
1039	-0,2131598	NA	-0,0015413	4,25E-05	-0,00164139	NA	NA	-1,09054773	NA	NA	NA	NA	0,33971014	NA	7	-20935,5727	41885,1517	4048,73495	0
3122	-8,51824578	0,51124721	NA	NA	NA	0,38535796	-0,65213053	0,72537252	NA	NA	NA	NA	0,45346103	-0,54414773	8	-20934,5914	41885,1907	4048,77395	0
3113	-3,98530292	NA	NA	NA	-0,00234183	NA	-0,67333606	0,72537252	NA	NA	NA	NA	0,45346103	-0,54414773	7	-20935,7369	41885,4799	4049,06322	0
607	-0,39160679	NA	0,00183718	4,30E-05	-0,00146197	0,48040455	NA	-0,66565225	-0,15004816	NA	NA	-0,00048657	NA	NA	9	-20933,7527	41885,5154	4049,0987	0

1707	-5,75020926	NA	0,0346068	NA	-0,00247017	NA	-0,61951905	-3,78227707	NA	0,0606519	NA	-0,00034645	0,38158141	NA	9	-20933,8809	41885,7717	4049,35502	0
1770	-6,22256645	0,32760888	NA	NA	-0,00183217	NA	-0,66370241	-3,6371906	-0,09814646	0,05972263	NA	-0,00047389	0,39127251	NA	10	-20934,5932	41889,1985	4052,78181	0
3682	-7,20552725	0,58574016	NA	NA	NA	NA	-0,62020516	1,15818376	-0,19528017	NA	NA	-0,00061584	0,46809624	-0,5501339	9	-20936,7777	41891,5654	4055,14871	0
1195	-5,75020926	NA	0,0346068	NA	-0,00247017	NA	-0,61951905	-3,95454473	NA	0,05999672	NA	NA	0,38996522	NA	8	-20938,224	41892,456	4056,03932	0
1706	-6,22256645	0,32760888	NA	NA	-0,00183217	NA	-0,66370241	-3,78227707	NA	0,0606519	NA	-0,00034645	0,38158141	NA	9	-20937,4294	41892,8688	4056,45208	0
1259	-5,75020926	NA	0,0346068	NA	-0,00247017	NA	-0,61951905	-3,92430325	-0,03654601	0,05956532	NA	NA	0,39466847	NA	9	-20937,7529	41893,5158	4057,09904	0
541	-0,31227401	NA	NA	4,32E-05	-0,00145314	0,47935513	NA	-0,82885018	NA	NA	NA	-0,0002841	NA	NA	7	-20940,8071	41895,6204	4059,20364	0
93	-0,31227401	NA	NA	4,32E-05	-0,00145314	0,47935513	NA	-0,96744453	-0,08610767	NA	NA	NA	NA	NA	7	-20941,0771	41896,1604	4059,74365	0
543	-0,39160679	NA	0,00183718	4,30E-05	-0,00146197	0,48040455	NA	-0,82885018	NA	NA	NA	-0,0002841	NA	NA	8	-20940,7637	41897,5353	4061,11859	0
95	-0,39160679	NA	0,00183718	4,30E-05	-0,00146197	0,48040455	NA	-0,96744453	-0,08610767	NA	NA	NA	NA	NA	8	-20941,0337	41898,0753	4061,6586	0
1194	-6,22256645	0,32760888	NA	NA	-0,00183217	NA	-0,66370241	-3,95454473	NA	0,05999672	NA	NA	0,38996522	NA	8	-20941,7726	41899,5531	4063,13638	0
3699	-8,8387814	NA	0,03010563	NA	NA	0,51295904	-0,60572616	1,15818376	-0,19528017	NA	NA	-0,00061584	0,46809624	-0,5501339	10	-20939,7944	41899,601	4063,18423	0
29	-0,31227401	NA	NA	4,32E-05	-0,00145314	0,47935513	NA	-0,99369134	NA	NA	NA	NA	NA	NA	6	-20943,8705	41899,7457	4063,32901	0
1258	-6,22256645	0,32760888	NA	NA	-0,00183217	NA	-0,66370241	-3,92430325	-0,03654601	0,05956532	NA	NA	0,39466847	NA	9	-20941,3014	41900,6128	4064,1961	0
31	-0,39160679	NA	0,00183718	4,30E-05	-0,00146197	0,48040455	NA	-0,99369134	NA	NA	NA	NA	NA	NA	7	-20943,8271	41901,6605	4065,24373	0
2713	-7,52922856	NA	NA	NA	-0,00175017	0,32067571	NA	-1,63848397	NA	0,05580518	NA	-0,00060814	NA	-0,48687105	8	-20942,9089	41901,8258	4065,40905	0
2698	-7,76791863	0,37580763	NA	NA	-0,00127907	NA	NA	-1,63848397	NA	0,05580518	NA	-0,00060814	NA	-0,48687105	8	-20942,972	41901,952	4065,53524	0
1764	-9,79595589	0,69445228	0,04389505	NA	NA	NA	-0,55018722	-3,6371906	-0,09814646	0,05972263	NA	-0,00047389	0,39127251	NA	10	-20941,3318	41902,6758	4066,2591	0
2777	-7,52922856	NA	NA	NA	-0,00175017	0,32067571	NA	-1,57337207	-0,04401868	0,05538852	NA	-0,0006675	NA	-0,48613452	9	-20942,3378	41902,6856	4066,26888	0
2762	-7,76791863	0,37580763	NA	NA	-0,00127907	NA	NA	-1,57337207	-0,04401868	0,05538852	NA	-0,0006675	NA	-0,48613452	9	-20942,4009	41902,8118	4066,39507	0
1700	-9,79595589	0,69445228	0,04389505	NA	NA	NA	-0,55018722	-3,78227707	NA	0,0606519	NA	-0,00034645	0,38158141	NA	9	-20944,1681	41906,3461	4069,92937	0
1778	-8,51824578	0,51124721	NA	NA	NA	0,38535796	-0,65213053	-3,6371906	-0,09814646	0,05972263	NA	-0,00047389	0,39127251	NA	10	-20943,3188	41906,6497	4070,233	0
1769	-3,98530292	NA	NA	NA	-0,00234183	NA	-0,67333606	-3,6371906	-0,09814646	0,05972263	NA	-0,00047389	0,39127251	NA	9	-20944,4643	41906,9386	4070,52183	0
2706	-8,08714822	0,50972296	NA	NA	NA	0,2963004	NA	-1,63848397	NA	0,05580518	NA	-0,00060814	NA	-0,48687105	8	-20945,7798	41907,5675	4071,15081	0
2202	-8,97566397	0,33138274	NA	NA	-0,0012458	0,28352203	NA	-1,98285198	NA	0,054624	NA	NA	NA	-0,47270606	8	-20945,8654	41907,7388	4071,32206	0
2707	-11,2963041	NA	0,04958538	NA	NA	0,45107925	NA	-1,63848397	NA	0,05580518	NA	-0,00060814	NA	-0,48687105	8	-20946,1555	41908,3189	4071,90222	0
2770	-8,08714822	0,50972296	NA	NA	NA	0,2963004	NA	-1,57337207	-0,04401868	0,05538852	NA	-0,0006675	NA	-0,48613452	9	-20945,2087	41908,4274	4072,01064	0
2266	-8,97566397	0,33138274	NA	NA	-0,0012458	0,28352203	NA	-2,01223022	0,04141198	0,05511629	NA	NA	NA	-0,47466077	9	-20945,2588	41908,5276	4072,11087	0
2771	-11,2963041	NA	0,04958538	NA	NA	0,45107925	NA	-1,57337207	-0,04401868	0,05538852	NA	-0,0006675	NA	-0,48613452	9	-20945,5844	41909,1788	4072,76204	0
1714	-8,51824578	0,51124721	NA	NA	NA	0,38535796	-0,65213053	-3,78227707	NA	0,0606519	NA	-0,00034645	0,38158141	NA	9	-20946,155	41910,32	4073,90327	0
1705	-3,98530292	NA	NA	NA	-0,00234183	NA	-0,67333606	-3,78227707	NA	0,0606519	NA	-0,00034645	0,38158141	NA	8	-20947,3005	41910,609	4074,19232	0
3170	-7,20552725	0,58574016	NA	NA	NA	NA	-0,62020516	0,7406183	-0,11449835	NA	NA	NA	0,46966671	-0,53963709	8	-20947,9125	41911,8329	4075,41621	0
3618	-7,20552725	0,58574016	NA	NA	NA	NA	-0,62020516	0,95852238	NA	NA	NA	-0,00035257	0,44577019	-0,55202249	8	-20948,1113	41912,2306	4075,81392	0
1188	-9,79595589	0,69445228	0,04389505	NA	NA	NA	-0,55018722	-3,95454473	NA	0,05999672	NA	NA	0,38996522	NA	8	-20948,5112	41913,0304	4076,61367	0
2684	-5,95790631	0,3741151	0,04613303	NA	-0,00180249	0,39660544	-0,62074526	1,12236839	-0,1349839	NA	NA	-0,00063299	NA	-0,50295006	11	-20945,5975	41913,2095	4076,79279	0
1252	-9,79595589	0,69445228	0,04389505	NA	NA	NA	-0,55018722	-3,92430325	-0,03654601	0,05956532	NA	NA	0,39466847	NA	9	-20948,0401	41914,0901	4077,67338	0
1202	-8,51824578	0,51124721	NA	NA	NA	0,38535796	-0,65213053	-3,95454473	NA	0,05999672	NA	NA	0,38996522	NA	8	-20950,4982	41917,0043	4080,58758	0
1193	-3,98530292	NA	NA	NA	-0,00234183	NA	-0,67333606	-3,95454473	NA	0,05999672	NA	NA	0,38996522	NA	7	-20951,6437	41917,2936	4080,87685	0
1266	-8,51824578	0,51124721	NA	NA	NA	0,38535796	-0,65213053	-3,92430325	-0,03654601	0,05956532	NA	NA	0,39466847	NA	9	-20950,027	41918,064	4081,64729	0
1257	-3,98530292	NA	NA	NA	-0,00234183	NA	-0,67333606	-3,92430325	-0,03654601	0,05956532	NA	NA	0,39466847	NA	8	-20951,1725	41918,3531	4081,93634	0
3697	-6,3315051	NA	NA	NA	NA	0,4838605	-0,6580268	1,15818376	-0,19528017	NA	NA	-0,00061584	0,46809624	-0,5501339	9	-20950,2089	41918,4278	4082,01109	0
3106	-7,20552725	0,58574016	NA	NA	NA	NA	-0,62020516	0,72537252	NA	NA	NA	NA	0,45346103	-0,54414773	7	-20952,6254	41919,257	4082,84028	0
3187	-8,8387814	NA	0,03010563	NA	NA	0,51295904	-0,60572616	0,7406183	-0,11449835	NA	NA	NA	0,46966671	-0,53963709	9	-20950,9291	41919,8682	4083,45152	0
700	-5,95790631	0,3741151	0,04613303	NA	-0,00180249	0,39660544	-0,62074526	-3,52383686	NA	0,05863003	NA	-0,00043937	NA	NA	10	-20949,9697	41919,9516	4083,53485	0
3635	-8,8387814	NA	0,03010563	NA	NA	0,51295904	-0,60572616	0,95852238	NA	NA	NA	-0,00035257	0,44577019	-0,55202249	9	-20951,128	41920,2659	4083,84922	0
764	-5,95790631	0,3741151	0,04613303	NA	-0,00180249	0,39660544	-0,62074526	-3,4425274	-0,05282396	0,05811259	NA	-0,00050954	NA	NA	11	-20949,132	41920,2786	4083,86186	0
2620	-5,95790631	0,3741151	0,04613303	NA	-0,00180249	0,39660544	-0,62074526	0,98389174	NA	NA	NA	-0,00044751	NA	-0,50601759	10	-20951,1511	41922,3143	4085,89756	0
2201	-7,52922856	NA	NA	NA	-0,00175017	0,32067571	NA	-1,98285198	NA	0,054624	NA	NA	NA	-0,47270606	7	-20955,6909	41925,388	4088,97128	0
2186	-7,76791863	0,37580763	NA	NA	-0,00127907	NA	NA	-1,98285198	NA	0,054624	NA	NA	NA	-0,47270606	7	-20955,754	41925,5142	4089,09747	0
2697	-6,18359995	NA	NA	NA	-0,00186372	NA	NA	-1,63848397	NA	0,05580518	NA	-0,00060814	NA	-0,48687105	7	-20955,8229	41925,652	4089,23532	0
2265	-7,52922856	NA	NA	NA	-0,00175017	0,32067571	NA	-2,01223022	0,04141198	0,05511629	NA	NA	NA	-0,47466077	8	-20955,0843	41926,1766	4089,75987	0
2250	-7,76791863	0,37580763	NA	NA	-0,00127907	NA	NA	-2,01223022	0,04141198	0,05511629	NA	NA	NA	-0,47466077	8	-20955,1474	41926,3028	4089,88606	0
2761	-6,18359995	NA	NA	NA	-0,00186372	NA	NA	-1,57337207	-0,04401868	0,05538852	NA	-0,0006675	NA	-0,48613452	8	-20955,2518	41926,5116	4090,09493	0
2690	-10,126072	0,56333515	NA	NA	NA	NA	NA	-1,63848397	NA	0,05580518	NA	-0,00060814	NA	-0,48687105	7	-20956,5754	41927,1569	4090,74022	0
3123	-8,8387814	NA	0,03010563	NA	NA	0,51295904	-0,60572616	0,72537252	NA	NA	NA	NA	0,45346103	-0,54414773	8	-20955,6421	41927,2921	4090,87536	0
2754	-10,126072	0,56333515	NA	NA	NA	NA	NA	-1,57337207	-0,04401868	0,05538852	NA	-0,0006675	NA	-0,48613452	8	-20956,0043	41928,0165	4091,59982	0
2194	-8,08714822	0,50972296	NA	NA	NA	0,2963004	NA	-1,98285198	NA	0,054624	NA	NA	NA	-0,47270606	7	-20958,5618	41931,1298	4094,71304	0
188	-5,95790631	0,3741151	0,04613303	NA	-0,00180249	0,39660544	-0,62074526	-3,73836005	NA	0,05777952	NA	NA	NA	NA	9	-20956,9236	41931,8572	4095,44043	0
2195	-11,2963041	NA	0,04958538	NA	NA	0,45107925	NA	-1,98285198	NA	0,054624	NA	NA	NA	-0,47270606	7	-20958,9375	41931,8812	4095,46445	0
2258	-8,08714822	0,50972296	NA	NA	NA	0,2963004	NA	-2,01223022	0,04141198	0,05511629	NA	NA	NA	-0,47466077	8	-20957,9552	41931,9184	4095,50163	0
3676	-9,61919161	0,46601352	0,06534536	NA	-0,00131078	0,33938341	NA	1,15818376	-0,19528017	NA	NA	-0,00061584	0,46809624	-0,5501339	11	-20955,1667	41932,3479	4095,93119	0

2259	-11,2963041	NA	0,04958538	NA	NA	0,45107925	NA	-2,01223022	0,04141198	0,05511629	NA	NA	NA	-0,47466077	8	-20958,3309	41932,6698	4096,25304	0
1639	-0,65771633	NA	-0,01187559	4,14E-05	NA	NA	-0,10732692	-0,7738147	-0,19560429	NA	NA	-0,00045111	0,35589058	NA	9	-20957,7434	41933,4968	4097,08005	0
252	-5,95790631	0,3741151	0,04613303	NA	-0,00180249	0,39660544	-0,62074526	-3,74999095	0,01316736	0,05794033	NA	NA	NA	NA	10	-20956,8611	41933,7344	4097,31765	0
2172	-5,95790631	0,3741151	0,04613303	NA	-0,00180249	0,39660544	-0,62074526	0,6960215	-0,05271384	NA	NA	NA	NA	-0,49283909	10	-20957,3764	41934,7649	4098,34819	0
2108	-5,95790631	0,3741151	0,04613303	NA	-0,00180249	0,39660544	-0,62074526	0,6894459	NA	NA	NA	NA	NA	-0,49566768	9	-20958,401	41934,812	4098,39526	0
1637	-1,22822825	NA	NA	4,02E-05	NA	NA	-0,09995451	-0,7738147	-0,19560429	NA	NA	-0,00045111	0,35589058	NA	8	-20959,4879	41934,9837	4098,56695	0
621	0,64576877	NA	NA	3,88E-05	-0,00183092	NA	-0,20438761	-0,66565225	-0,15004816	NA	NA	-0,00048657	NA	NA	8	-20959,4981	41935,0042	4098,58748	0
2683	-8,35316771	NA	0,03864408	NA	-0,00234345	0,43617682	-0,64441699	1,12236839	-0,1349839	NA	NA	-0,00063299	NA	-0,50295006	10	-20957,5031	41935,0184	4098,60166	0
629	-0,84339357	NA	NA	4,07E-05	NA	0,55505772	-0,13640315	-0,66565225	-0,15004816	NA	NA	-0,00048657	NA	NA	8	-20959,7563	41935,5206	4099,10391	0
1607	-1,14142692	NA	-0,01089562	4,31E-05	NA	NA	NA	-0,7738147	-0,19560429	NA	NA	-0,00045111	0,35589058	NA	8	-20959,8575	41935,723	4099,30626	0
631	-0,50593593	NA	-0,00700816	4,14E-05	NA	0,55021643	-0,14061875	-0,66565225	-0,15004816	NA	NA	-0,00048657	NA	NA	9	-20959,1233	41936,2565	4099,83978	0
1605	-1,63084607	NA	NA	4,18E-05	NA	NA	NA	-0,7738147	-0,19560429	NA	NA	-0,00045111	0,35589058	NA	7	-20961,3327	41936,6716	4100,25485	0
623	0,74969745	NA	-0,00245756	3,91E-05	-0,00181949	NA	-0,20513804	-0,66565225	-0,15004816	NA	NA	-0,00048657	NA	NA	9	-20959,4225	41936,855	4100,43832	0
3185	-6,3315051	NA	NA	NA	NA	0,4838605	-0,6580268	0,7406183	-0,11449835	NA	NA	NA	0,46966671	-0,53963709	8	-20961,3437	41938,6953	4102,2786	0
3633	-6,3315051	NA	NA	NA	NA	0,4838605	-0,6580268	0,95852238	NA	NA	NA	-0,00035257	0,44577019	-0,55202249	8	-20961,5425	41939,093	4102,6763	0
597	-1,36352414	NA	NA	4,29E-05	NA	0,54467715	NA	-0,66565225	-0,15004816	NA	NA	-0,00048657	NA	NA	7	-20963,2967	41940,5996	4104,1829	0
1762	-7,20552725	0,58574016	NA	NA	NA	NA	-0,62020516	-3,6371906	-0,09814646	0,05972263	NA	-0,00047389	0,39127251	NA	9	-20961,3528	41940,7156	4104,29889	0
599	-1,08960867	NA	-0,00576757	4,36E-05	NA	0,53973144	NA	-0,66565225	-0,15004816	NA	NA	-0,00048657	NA	NA	8	-20962,8644	41941,7368	4105,3201	0
699	-8,35316771	NA	0,03864408	NA	-0,00234345	0,43617682	-0,64441699	-3,52383686	NA	0,05863003	NA	-0,00043937	NA	NA	9	-20961,8754	41941,7607	4105,34395	0
763	-8,35316771	NA	0,03864408	NA	-0,00234345	0,43617682	-0,64441699	-3,4425274	-0,05282396	0,05811259	NA	-0,00050954	NA	NA	10	-20961,0376	41942,0875	4105,67074	0
1127	-0,65771633	NA	-0,01187559	4,14E-05	NA	NA	-0,10732692	-1,05468211	-0,1361791	NA	NA	NA	0,35946641	NA	8	-20964,0302	41944,0685	4107,65173	0
2619	-8,35316771	NA	0,03864408	NA	-0,00234345	0,43617682	-0,64441699	0,98389174	NA	NA	NA	-0,00044751	NA	-0,50601759	9	-20963,0567	41944,1234	4107,70666	0
1698	-7,20552725	0,58574016	NA	NA	NA	NA	-0,62020516	-3,78227707	NA	0,0606519	NA	-0,00034645	0,38158141	NA	8	-20964,1891	41944,3861	4107,96938	0
1125	-1,22822825	NA	NA	4,02E-05	NA	NA	-0,09995451	-1,05468211	-0,1361791	NA	NA	NA	0,35946641	NA	7	-20965,7747	41945,5556	4109,13886	0
3121	-6,3315051	NA	NA	NA	NA	0,4838605	-0,6580268	0,72537252	NA	NA	NA	NA	0,45346103	-0,54414773	7	-20966,0566	41946,1194	4109,70266	0
1095	-1,14142692	NA	-0,01089562	4,31E-05	NA	NA	NA	-1,05468211	-0,1361791	NA	NA	NA	0,35946641	NA	7	-20966,1443	41946,2949	4109,87817	0
557	0,64576877	NA	NA	3,88E-05	-0,00183092	NA	-0,20438761	-0,82885018	NA	NA	NA	-0,0002841	NA	NA	7	-20966,5091	41947,0243	4110,60759	0
1093	-1,63084607	NA	NA	4,18E-05	NA	NA	NA	-1,05468211	-0,1361791	NA	NA	NA	0,35946641	NA	6	-20967,6195	41947,2437	4110,82698	0
565	-0,84339357	NA	NA	4,07E-05	NA	0,55505772	-0,13640315	-0,82885018	NA	NA	NA	-0,0002841	NA	NA	7	-20966,7673	41947,5407	4111,12402	0
109	0,64576877	NA	NA	3,88E-05	-0,00183092	NA	-0,20438761	-0,96744453	-0,08610767	NA	NA	NA	NA	NA	7	-20966,7791	41947,5643	4111,1476	0
117	-0,84339357	NA	NA	4,07E-05	NA	0,55505772	-0,13640315	-0,96744453	-0,08610767	NA	NA	NA	NA	NA	7	-20967,0373	41948,0808	4111,66403	0
567	-0,50593593	NA	-0,00700816	4,14E-05	NA	0,55021643	-0,14061875	-0,82885018	NA	NA	NA	-0,0002841	NA	NA	8	-20966,1342	41948,2764	4111,85967	0
1779	-8,8387814	NA	0,03010563	NA	NA	0,51295904	-0,60572616	-3,6371906	-0,09814646	0,05972263	NA	-0,00047389	0,39127251	NA	10	-20964,3695	41948,7511	4112,33441	0
119	-0,50593593	NA	-0,00700816	4,14E-05	NA	0,55021643	-0,14061875	-0,96744453	-0,08610767	NA	NA	NA	NA	NA	8	-20966,4042	41948,8164	4112,39968	0
559	0,74969745	NA	-0,00245756	3,91E-05	-0,00181949	NA	-0,20513804	-0,82885018	NA	NA	NA	-0,0002841	NA	NA	8	-20966,4335	41948,8749	4112,45821	0
2185	-6,18359995	NA	NA	NA	-0,00186372	NA	NA	-1,98285198	NA	0,054624	NA	NA	NA	-0,47270606	6	-20968,6049	41949,2145	4112,79777	0
2668	-9,35928532	0,43489058	0,04364193	NA	-0,00182965	NA	-0,59565606	1,12236839	-0,1349839	NA	NA	-0,00063299	NA	-0,50295006	10	-20964,7008	41949,4137	4112,99697	0
111	0,74969745	NA	-0,00245756	3,91E-05	-0,00181949	NA	-0,20513804	-0,96744453	-0,08610767	NA	NA	NA	NA	NA	8	-20966,7035	41949,4149	4112,99822	0
589	-0,28011525	NA	NA	4,24E-05	-0,0016491	NA	NA	-0,66565225	-0,15004816	NA	NA	-0,00048657	NA	NA	7	-20967,7884	41949,583	4113,16632	0
2249	-6,18359995	NA	NA	NA	-0,00186372	NA	NA	-2,01223022	0,04141198	0,05511629	NA	NA	NA	-0,47466077	7	-20967,9983	41950,0029	4113,58614	0
2178	-10,126072	0,56333515	NA	NA	NA	NA	NA	-1,98285198	NA	0,054624	NA	NA	NA	-0,47270606	6	-20969,3574	41950,7194	4114,30267	0
1186	-7,20552725	0,58574016	NA	NA	NA	NA	-0,62020516	-3,95454473	NA	0,05999672	NA	NA	0,38996522	NA	7	-20968,5322	41951,0706	4114,65391	0
45	0,64576877	NA	NA	3,88E-05	-0,00183092	NA	-0,20438761	-0,99369134	NA	NA	NA	NA	NA	NA	6	-20969,5725	41951,1497	4114,73296	0
2242	-10,126072	0,56333515	NA	NA	NA	NA	NA	-2,01223022	0,04141198	0,05511629	NA	NA	NA	-0,47466077	7	-20968,7508	41951,5078	4115,09104	0
591	-0,2131598	NA	-0,0015413	4,25E-05	-0,00164139	NA	NA	-0,66565225	-0,15004816	NA	NA	-0,00048657	NA	NA	8	-20967,7587	41951,5254	4115,1087	0
53	-0,84339357	NA	NA	4,07E-05	NA	0,55505772	-0,13640315	-0,99369134	NA	NA	NA	NA	NA	NA	6	-20969,8307	41951,6661	4115,24939	0
1250	-7,20552725	0,58574016	NA	NA	NA	NA	-0,62020516	-3,92430325	-0,03654601	0,05956532	NA	NA	0,39466847	NA	8	-20968,0611	41952,1301	4115,7134	0
55	-0,50593593	NA	-0,00700816	4,14E-05	NA	0,55021643	-0,14061875	-0,99369134	NA	NA	NA	NA	NA	NA	7	-20969,1977	41952,4015	4115,98482	0
1715	-8,8387814	NA	0,03010563	NA	NA	0,51295904	-0,60572616	-3,78227707	NA	0,0606519	NA	-0,00034645	0,38158141	NA	9	-20967,2057	41952,4214	4116,00469	0
3164	-9,61919161	0,46601352	0,06534536	NA	-0,00131078	0,33938341	NA	0,7406183	-0,11449835	NA	NA	NA	0,46966671	-0,53963709	10	-20966,3014	41952,615	4116,19825	0
533	-1,36352414	NA	NA	4,29E-05	NA	0,54467715	NA	-0,82885018	NA	NA	NA	-0,0002841	NA	NA	6	-20970,3077	41952,62	4116,20323	0
47	0,74969745	NA	-0,00245756	3,91E-05	-0,00181949	NA	-0,20513804	-0,99369134	NA	NA	NA	NA	NA	NA	7	-20969,4969	41953,0001	4116,58336	0
3612	-9,61919161	0,46601352	0,06534536	NA	-0,00131078	0,33938341	NA	0,95852238	NA	NA	NA	-0,00035257	0,44577019	-0,55202249	10	-20966,5003	41953,0127	4116,59596	0
85	-1,36352414	NA	NA	4,29E-05	NA	0,54467715	NA	-0,96744453	-0,08610767	NA	NA	NA	NA	NA	6	-20970,5777	41953,16	4116,74324	0
187	-8,35316771	NA	0,03864408	NA	-0,00234345	0,43617682	-0,64441699	-3,73836005	NA	0,05777952	NA	NA	NA	NA	8	-20968,8293	41953,6665	4117,24976	0
535	-1,08960867	NA	-0,00576757	4,36E-05	NA	0,53973144	NA	-0,82885018	NA	NA	NA	-0,0002841	NA	NA	7	-20969,8754	41953,7569	4117,3402	0
87	-1,08960867	NA	-0,00576757	4,36E-05	NA	0,53973144	NA	-0,96744453	-0,08610767	NA	NA	NA	NA	NA	7	-20970,1454	41954,2969	4117,88021	0
1575	-0,65771633	NA	-0,01187559	4,14E-05	NA	NA	-0,10732692	-0,97642003	NA	NA	NA	-0,00019403	0,33432079	NA	8	-20969,4177	41954,8434	4118,4267	0
251	-8,35316771	NA	0,03864408	NA	-0,00234345	0,43617682	-0,64441699	-3,74999095	0,01316736	0,05794033	NA	NA	NA	NA	9	-20968,7668	41955,5435	4119,12675	0
1063	-0,65771633	NA	-0,01187559	4,14E-05	NA	NA	-0,10732692	-1,09054773	NA	NA	NA	NA	0,33971014	NA	7	-20970,8528	41955,7118	4119,29509	0
2682	-6,98837994	0,26427623	NA	NA	-0,00180413	0,37036432	-0,69230587	1,12236839	-0,1349839	NA	NA	-0,00063299	NA	-0,50295006	10	-20967,9214	41955,8549	4119,43815	0

684	-9,35928532	0,43489058	0,04364193	NA	-0,00182965	NA	-0,59565606	-3,52383686	NA	0,05863003	NA	-0,00043937	NA	NA	9	-20969,073	41956,156	4119,73925	0
1573	-1,22822825	NA	NA	4,02E-05	NA	NA	-0,09995451	-0,97642003	NA	NA	NA	-0,00019403	0,33432079	NA	7	-20971,1622	41956,3305	4119,91382	0
748	-9,35928532	0,43489058	0,04364193	NA	-0,00182965	NA	-0,59565606	-3,4425274	-0,05282396	0,05811259	NA	-0,00050954	NA	NA	10	-20968,2353	41956,4828	4120,06604	0
2171	-8,35316771	NA	0,03864408	NA	-0,00234345	0,43617682	-0,64441699	0,6960215	-0,05271384	NA	NA	NA	NA	-0,49283909	9	-20969,282	41956,574	4120,15729	0
2107	-8,35316771	NA	0,03864408	NA	-0,00234345	0,43617682	-0,64441699	0,6894459	NA	NA	NA	NA	NA	-0,49566768	8	-20970,3067	41956,6213	4120,20458	0
21	-1,36352414	NA	NA	4,29E-05	NA	0,54467715	NA	-0,99369134	NA	NA	NA	NA	NA	NA	5	-20973,3711	41956,7455	4120,32882	0
1543	-1,14142692	NA	-0,01089562	4,31E-05	NA	NA	NA	-0,97642003	NA	NA	NA	-0,00019403	0,33432079	NA	7	-20971,5318	41957,0699	4120,65313	0
1061	-1,22822825	NA	NA	4,02E-05	NA	NA	-0,09995451	-1,09054773	NA	NA	NA	NA	0,33971014	NA	6	-20972,5973	41957,1992	4120,78243	0
2691	-11,7728913	NA	0,04392487	NA	NA	NA	NA	-1,63848397	NA	0,05580518	NA	-0,00060814	NA	-0,48687105	7	-20971,7779	41957,5619	4121,14522	0
23	-1,08960867	NA	-0,00576757	4,36E-05	NA	0,53973144	NA	-0,99369134	NA	NA	NA	NA	NA	NA	6	-20972,9388	41957,8823	4121,46557	0
1031	-1,14142692	NA	-0,01089562	4,31E-05	NA	NA	NA	-1,09054773	NA	NA	NA	NA	0,33971014	NA	6	-20972,9669	41957,9385	4121,52174	0
1541	-1,63084607	NA	NA	4,18E-05	NA	NA	NA	-0,97642003	NA	NA	NA	-0,00019403	0,33432079	NA	6	-20973,007	41958,0187	4121,60194	0
3668	-13,7669049	0,65178191	0,06462958	NA	NA	0,35119819	NA	1,15818376	-0,19528017	NA	NA	-0,00061584	0,46809624	-0,5501339	10	-20969,049	41958,1101	4121,69337	0
2755	-11,7728913	NA	0,04392487	NA	NA	NA	NA	-1,57337207	-0,04401868	0,05538852	NA	-0,0006675	NA	-0,48613452	8	-20971,2068	41958,4215	4122,00483	0
3660	-11,9654714	0,51367836	0,06255183	NA	-0,0013444	NA	NA	1,15818376	-0,19528017	NA	NA	-0,00061584	0,46809624	-0,5501339	10	-20969,2375	41958,4872	4122,07052	0
2604	-9,35928532	0,43489058	0,04364193	NA	-0,00182965	NA	-0,59565606	0,98389174	NA	NA	NA	-0,00044751	NA	-0,50601759	9	-20970,2544	41958,5187	4122,10196	0
1029	-1,63084607	NA	NA	4,18E-05	NA	NA	NA	-1,09054773	NA	NA	NA	NA	0,33971014	NA	5	-20974,4421	41958,8875	4122,47078	0
1203	-8,8387814	NA	0,03010563	NA	NA	0,51295904	-0,60572616	-3,95454473	NA	0,05999672	NA	NA	0,38996522	NA	8	-20971,5489	41959,1057	4122,68899	0
3100	-9,61919161	0,46601352	0,06534536	NA	-0,00131078	0,33938341	NA	0,72537252	NA	NA	NA	NA	0,45346103	-0,54414773	9	-20971,0143	41960,0386	4123,62187	0
1267	-8,8387814	NA	0,03010563	NA	NA	0,51295904	-0,60572616	-3,92430325	-0,03654601	0,05956532	NA	NA	0,39466847	NA	9	-20971,0777	41960,1654	4123,7487	0
525	-0,28011525	NA	NA	4,24E-05	-0,0016491	NA	NA	-0,82885018	NA	NA	NA	-0,0002841	NA	NA	6	-20974,7994	41961,6034	4125,18665	0
2676	-10,737769	0,62231984	0,04626548	NA	NA	0,40997724	-0,57836783	1,12236839	-0,1349839	NA	NA	-0,00063299	NA	-0,50295006	10	-20970,9287	41961,8696	4125,45284	0
77	-0,28011525	NA	NA	4,24E-05	-0,0016491	NA	NA	-0,96744453	-0,08610767	NA	NA	NA	NA	NA	6	-20975,0694	41962,1434	4125,72666	0
698	-6,98837994	0,26427623	NA	NA	-0,00180413	0,37036432	-0,69230587	-3,52383686	NA	0,05863003	NA	-0,00043937	NA	NA	9	-20972,2936	41962,5972	4126,18043	0
762	-6,98837994	0,26427623	NA	NA	-0,00180413	0,37036432	-0,69230587	-3,4425274	-0,05282396	0,05811259	NA	-0,00050954	NA	NA	10	-20971,4559	41962,9239	4126,50722	0
527	-0,2131598	NA	-0,0015413	4,25E-05	-0,00164139	NA	NA	-0,82885018	NA	NA	NA	-0,0002841	NA	NA	7	-20974,7697	41963,5455	4127,12881	0
3683	-9,52399274	NA	0,02470059	NA	NA	NA	-0,56950148	1,15818376	-0,19528017	NA	NA	-0,00061584	0,46809624	-0,5501339	9	-20972,8341	41963,6781	4127,26137	0
79	-0,2131598	NA	-0,0015413	4,25E-05	-0,00164139	NA	NA	-0,96744453	-0,08610767	NA	NA	NA	NA	NA	7	-20975,0397	41964,0855	4127,66882	0
2618	-6,98837994	0,26427623	NA	NA	-0,00180413	0,37036432	-0,69230587	0,98389174	NA	NA	NA	-0,00044751	NA	-0,50601759	9	-20973,475	41964,9599	4128,54314	0
13	-0,28011525	NA	NA	4,24E-05	-0,0016491	NA	NA	-0,99369134	NA	NA	NA	NA	NA	NA	5	-20977,8628	41965,729	4129,31224	0
2681	-5,46460783	NA	NA	NA	-0,00220571	0,40342229	-0,70298126	1,12236839	-0,1349839	NA	NA	-0,00063299	NA	-0,50295006	9	-20974,2274	41966,4648	4130,04809	0
3675	-11,3659105	NA	0,05744064	NA	-0,00198557	0,38445946	NA	1,15818376	-0,19528017	NA	NA	-0,00061584	0,46809624	-0,5501339	10	-20973,6596	41967,3313	4130,91459	0
1777	-6,3315051	NA	NA	NA	NA	0,4838605	-0,6580268	-3,6371906	-0,09814646	0,05972263	NA	-0,00047389	0,39127251	NA	9	-20974,784	41967,578	4131,16127	0
15	-0,2131598	NA	-0,0015413	4,25E-05	-0,00164139	NA	NA	-0,99369134	NA	NA	NA	NA	NA	NA	6	-20977,8331	41967,6709	4131,25418	0
2705	-9,09446706	NA	NA	NA	NA	0,38722306	NA	-1,63848397	NA	0,05580518	NA	-0,00060814	NA	-0,48687105	7	-20976,9113	41967,8287	4131,41202	0
172	-9,35928532	0,43489058	0,04364193	NA	-0,00182965	NA	-0,59565606	-3,73836005	NA	0,05777952	NA	NA	NA	NA	8	-20976,0269	41968,0618	4131,64506	0
692	-10,737769	0,62231984	0,04626548	NA	NA	0,40997724	-0,57836783	-3,52383686	NA	0,05863003	NA	-0,00043937	NA	NA	9	-20975,3009	41968,6118	4132,19513	0
2769	-9,09446706	NA	NA	NA	NA	0,38722306	NA	-1,57337207	-0,04401868	0,05538852	NA	-0,0006675	NA	-0,48613452	8	-20976,3402	41968,6883	4132,27162	0
756	-10,737769	0,62231984	0,04626548	NA	NA	0,40997724	-0,57836783	-3,4425274	-0,05282396	0,05811259	NA	-0,00050954	NA	NA	10	-20974,4632	41968,9386	4132,52191	0
236	-9,35928532	0,43489058	0,04364193	NA	-0,00182965	NA	-0,59565606	-3,74999095	0,01316736	0,05794033	NA	NA	NA	NA	9	-20975,9644	41969,9388	4133,52205	0
2156	-9,35928532	0,43489058	0,04364193	NA	-0,00182965	NA	-0,59565606	0,6960215	-0,05271384	NA	NA	NA	NA	-0,49283909	9	-20976,4797	41970,9693	4134,55259	0
2612	-10,737769	0,62231984	0,04626548	NA	NA	0,40997724	-0,57836783	0,98389174	NA	NA	NA	-0,00044751	NA	-0,50601759	9	-20976,4823	41970,9746	4134,55784	0
2092	-9,35928532	0,43489058	0,04364193	NA	-0,00182965	NA	-0,59565606	0,6894459	NA	NA	NA	NA	NA	-0,49566768	8	-20977,5043	41971,0166	4134,59989	0
1713	-6,3315051	NA	NA	NA	NA	0,4838605	-0,6580268	-3,78227707	NA	0,0606519	NA	-0,00034645	0,38158141	NA	8	-20977,6203	41971,2485	4134,83177	0
697	-5,46460783	NA	NA	NA	-0,00220571	0,40342229	-0,70298126	-3,52383686	NA	0,05863003	NA	-0,00043937	NA	NA	8	-20978,5997	41973,2073	4136,7906	0
761	-5,46460783	NA	NA	NA	-0,00220571	0,40342229	-0,70298126	-3,4425274	-0,05282396	0,05811259	NA	-0,00050954	NA	NA	9	-20977,762	41973,5339	4137,11717	0
186	-6,98837994	0,26427623	NA	NA	-0,00180413	0,37036432	-0,69230587	-3,73836005	NA	0,05777952	NA	NA	NA	NA	8	-20979,2475	41974,503	4138,08624	0
2617	-5,46460783	NA	NA	NA	-0,00220571	0,40342229	-0,70298126	0,98389174	NA	NA	NA	-0,00044751	NA	-0,50601759	8	-20979,781	41975,57	4139,15331	0
3681	-8,58193391	NA	NA	NA	NA	NA	-0,61393687	1,15818376	-0,19528017	NA	NA	-0,00061584	0,46809624	-0,5501339	8	-20979,8501	41975,7083	4139,29154	0
250	-6,98837994	0,26427623	NA	NA	-0,00180413	0,37036432	-0,69230587	-3,74999095	0,01316736	0,05794033	NA	NA	NA	NA	9	-20979,185	41976,38	4139,96323	0
2170	-6,98837994	0,26427623	NA	NA	-0,00180413	0,37036432	-0,69230587	0,6960215	-0,05271384	NA	NA	NA	NA	-0,49283909	9	-20979,7003	41977,4105	4140,99377	0
2106	-6,98837994	0,26427623	NA	NA	-0,00180413	0,37036432	-0,69230587	0,6894459	NA	NA	NA	NA	NA	-0,49566768	8	-20980,7249	41977,4578	4141,04107	0
1201	-6,3315051	NA	NA	NA	NA	0,4838605	-0,6580268	-3,95454473	NA	0,05999672	NA	NA	0,38996522	NA	7	-20981,9634	41977,933	4141,51629	0
3156	-13,7669049	0,65178191	0,06462958	NA	NA	0,35119819	NA	0,7406183	-0,11449835	NA	NA	NA	0,46966671	-0,53963709	9	-20980,1837	41978,3774	4141,96066	0
3148	-11,9654714	0,51367836	0,06255183	NA	-0,0013444	NA	NA	0,7406183	-0,11449835	NA	NA	NA	0,46966671	-0,53963709	9	-20980,3723	41978,7545	4142,3378	0
3604	-13,7669049	0,65178191	0,06462958	NA	NA	0,35119819	NA	0,95852238	NA	NA	NA	-0,00035257	0,44577019	-0,55202249	9	-20980,3826	41978,7751	4142,35836	0
1265	-6,3315051	NA	NA	NA	NA	0,4838605	-0,6580268	-3,92430325	-0,03654601	0,05956532	NA	NA	0,39466847	NA	8	-20981,4923	41978,9925	4142,57578	0
3596	-11,9654714	0,51367836	0,06255183	NA	-0,0013444	NA	NA	0,95852238	NA	NA	NA	-0,00035257	0,44577019	-0,55202249	9	-20980,5711	41979,1522	4142,7355	0
2667	-5,75020926	NA	0,0346068	NA	-0,00247017	NA	-0,61951905	1,12236839	-0,1349839	NA	NA	-0,00063299	NA	-0,50295006	9	-20981,0541	41980,1181	4143,70134	0
180	-10,737769	0,62231984	0,04626548	NA	NA	0,40997724	-0,57836783	-3,73836005	NA	0,05777952	NA	NA	NA	NA	8	-20982,2548	41980,5177	4144,10093	0
2179	-11,7728913	NA	0,04392487	NA	NA	NA	NA	-1,98285198	NA	0,054624	NA	NA	NA	-0,47270606	6	-20984,5599	41981,1244	4144,70767	0

1756	-9,61919161	0,46601352	0,06534536	NA	-0,00131078	0,33938341	NA	-3,6371906	-0,09814646	0,05972263	NA	-0,00047389	0,39127251	NA	11	-20979,7417	41981,4981	4145,08137	0
2243	-11,7728913	NA	0,04392487	NA	NA	NA	NA	-2,01223022	0,04141198	0,05511629	NA	NA	NA	-0,47466077	7	-20983,9533	41981,9128	4145,49604	0
244	-10,737769	0,62231984	0,04626548	NA	NA	0,40997724	-0,57836783	-3,74999095	0,01316736	0,05794033	NA	NA	NA	NA	9	-20982,1923	41982,3946	4145,97792	0
2164	-10,737769	0,62231984	0,04626548	NA	NA	0,40997724	-0,57836783	0,6960215	-0,05271384	NA	NA	NA	NA	-0,49283909	9	-20982,7076	41983,4252	4147,00846	0
2100	-10,737769	0,62231984	0,04626548	NA	NA	0,40997724	-0,57836783	0,6894459	NA	NA	NA	NA	NA	-0,49566768	8	-20983,7323	41983,4725	4147,05576	0
3171	-9,52399274	NA	0,02470059	NA	NA	NA	-0,56950148	0,7406183	-0,11449835	NA	NA	NA	0,46966671	-0,53963709	8	-20983,9688	41983,9456	4147,52887	0
3619	-9,52399274	NA	0,02470059	NA	NA	NA	-0,56950148	0,95852238	NA	NA	NA	-0,00035257	0,44577019	-0,55202249	8	-20984,1677	41984,3433	4147,92658	0
185	-5,46460783	NA	NA	NA	-0,00220571	0,40342229	-0,70298126	-3,73836005	NA	0,05777952	NA	NA	NA	NA	7	-20985,5536	41985,1133	4148,69663	0
1692	-9,61919161	0,46601352	0,06534536	NA	-0,00131078	0,33938341	NA	-3,78227707	NA	0,0606519	NA	-0,00034645	0,38158141	NA	10	-20982,578	41985,1681	4148,75142	0
3092	-13,7669049	0,65178191	0,06462958	NA	NA	0,35119819	NA	0,72537252	NA	NA	NA	NA	0,45346103	-0,54414773	8	-20984,8966	41985,8012	4149,3845	0
3084	-11,9654714	0,51367836	0,06255183	NA	-0,0013444	NA	NA	0,72537252	NA	NA	NA	NA	0,45346103	-0,54414773	8	-20985,0852	41986,1784	4149,76164	0
3652	-12,0135881	0,70944129	0,06178489	NA	NA	NA	NA	1,15818376	-0,19528017	NA	NA	-0,00061584	0,46809624	-0,5501339	9	-20984,136	41986,2819	4149,86513	0
683	-5,75020926	NA	0,0346068	NA	-0,00247017	NA	-0,61951905	-3,52383686	NA	0,05863003	NA	-0,00043937	NA	NA	8	-20985,4263	41986,8606	4150,44384	0
249	-5,46460783	NA	NA	NA	-0,00220571	0,40342229	-0,70298126	-3,74999095	0,01316736	0,05794033	NA	NA	NA	NA	8	-20985,4911	41986,9901	4150,5734	0
747	-5,75020926	NA	0,0346068	NA	-0,00247017	NA	-0,61951905	-3,4425274	-0,05282396	0,05811259	NA	-0,00050954	NA	NA	9	-20984,5886	41987,1871	4150,77041	0
2666	-6,22256645	0,32760888	NA	NA	-0,00183217	NA	-0,66370241	1,12236839	-0,1349839	NA	NA	-0,00063299	NA	-0,50295006	9	-20984,6026	41987,2151	4150,7984	0
3163	-11,3659105	NA	0,05744064	NA	-0,00198557	0,38445946	NA	0,7406183	-0,11449835	NA	NA	NA	0,46966671	-0,53963709	9	-20984,7943	41987,5986	4151,18188	0
3611	-11,3659105	NA	0,05744064	NA	-0,00198557	0,38445946	NA	0,95852238	NA	NA	NA	-0,00035257	0,44577019	-0,55202249	9	-20984,9932	41987,9963	4151,57958	0
2169	-5,46460783	NA	NA	NA	-0,00220571	0,40342229	-0,70298126	0,6960215	-0,05271384	NA	NA	NA	NA	-0,49283909	8	-20986,0063	41988,0207	4151,60394	0
2105	-5,46460783	NA	NA	NA	-0,00220571	0,40342229	-0,70298126	0,6894459	NA	NA	NA	NA	NA	-0,49566768	7	-20987,031	41988,0682	4151,65145	0
2603	-5,75020926	NA	0,0346068	NA	-0,00247017	NA	-0,61951905	0,98389174	NA	NA	NA	-0,00044751	NA	-0,50601759	8	-20986,6077	41989,2233	4152,80655	0
3107	-9,52399274	NA	0,02470059	NA	NA	NA	-0,56950148	0,72537252	NA	NA	NA	NA	0,45346103	-0,54414773	7	-20988,6817	41991,3697	4154,95294	0
2193	-9,09446706	NA	NA	NA	NA	0,38722306	NA	-1,98285198	NA	0,054624	NA	NA	NA	-0,47270606	6	-20989,6933	41991,3912	4154,97447	0
1180	-9,61919161	0,46601352	0,06534536	NA	-0,00131078	0,33938341	NA	-3,95454473	NA	0,05999672	NA	NA	0,38996522	NA	9	-20986,9211	41991,8522	4155,4355	0
2257	-9,09446706	NA	NA	NA	NA	0,38722306	NA	-2,01223022	0,04141198	0,05511629	NA	NA	NA	-0,47466077	7	-20989,0867	41992,1796	4155,76283	0
1244	-9,61919161	0,46601352	0,06534536	NA	-0,00131078	0,33938341	NA	-3,92430325	-0,03654601	0,05956532	NA	NA	0,39466847	NA	10	-20986,45	41992,9122	4156,49544	0
682	-6,22256645	0,32760888	NA	NA	-0,00183217	NA	-0,66370241	-3,52383686	NA	0,05863003	NA	-0,00043937	NA	NA	8	-20988,9748	41993,9576	4157,5409	0
746	-6,22256645	0,32760888	NA	NA	-0,00183217	NA	-0,66370241	-3,4425274	-0,05282396	0,05811259	NA	-0,00050954	NA	NA	9	-20988,1371	41994,2842	4157,86747	0
3099	-11,3659105	NA	0,05744064	NA	-0,00198557	0,38445946	NA	0,72537252	NA	NA	NA	NA	0,45346103	-0,54414773	8	-20989,5072	41995,0224	4158,60572	0
3169	-8,58193391	NA	NA	NA	NA	NA	-0,61393687	0,7406183	-0,11449835	NA	NA	NA	0,46966671	-0,53963709	7	-20990,9849	41995,976	4159,55927	0
2602	-6,22256645	0,32760888	NA	NA	-0,00183217	NA	-0,66370241	0,98389174	NA	NA	NA	-0,00044751	NA	-0,50601759	8	-20990,1562	41996,3203	4159,90361	0
3617	-8,58193391	NA	NA	NA	NA	NA	-0,61393687	0,95852238	NA	NA	NA	-0,00035257	0,44577019	-0,55202249	7	-20991,1837	41996,3737	4159,95697	0
171	-5,75020926	NA	0,0346068	NA	-0,00247017	NA	-0,61951905	-3,73836005	NA	0,05777952	NA	NA	NA	NA	7	-20992,3802	41998,7666	4162,34987	0
235	-5,75020926	NA	0,0346068	NA	-0,00247017	NA	-0,61951905	-3,74999095	0,01316736	0,05794033	NA	NA	NA	NA	8	-20992,3177	42000,6434	4164,22664	0
2660	-9,79595589	0,69445228	0,04389505	NA	NA	NA	-0,55018722	1,12236839	-0,1349839	NA	NA	-0,00063299	NA	-0,50295006	9	-20991,3412	42000,6924	4164,27568	0
2155	-5,75020926	NA	0,0346068	NA	-0,00247017	NA	-0,61951905	0,6960215	-0,05271384	NA	NA	NA	NA	-0,49283909	8	-20992,833	42001,6739	4165,25718	0
2091	-5,75020926	NA	0,0346068	NA	-0,00247017	NA	-0,61951905	0,6894459	NA	NA	NA	NA	NA	-0,49566768	7	-20993,8576	42001,7214	4165,3047	0
3659	-9,45532716	NA	0,05329757	NA	-0,00210061	NA	NA	1,15818376	-0,19528017	NA	NA	-0,00061584	0,46809624	-0,5501339	9	-20992,0151	42002,0402	4165,62346	0
3105	-8,58193391	NA	NA	NA	NA	NA	-0,61393687	0,72537252	NA	NA	NA	NA	0,45346103	-0,54414773	6	-20995,6978	42003,4003	4166,98355	0
2689	-15,360872	NA	NA	NA	NA	NA	NA	-1,63848397	NA	0,05580518	NA	-0,00060814	NA	-0,48687105	6	-20996,0534	42004,1115	4167,69474	0
2674	-8,51824578	0,51124721	NA	NA	NA	0,38535796	-0,65213053	1,12236839	-0,1349839	NA	NA	-0,00063299	NA	-0,50295006	9	-20993,3282	42004,6663	4168,24959	0
2665	-3,98530292	NA	NA	NA	-0,00234183	NA	-0,67333606	1,12236839	-0,1349839	NA	NA	-0,00063299	NA	-0,50295006	8	-20994,4737	42004,9554	4168,53864	0
2753	-15,360872	NA	NA	NA	NA	NA	NA	-1,57337207	-0,04401868	0,05538852	NA	-0,0006675	NA	-0,48613452	7	-20995,4823	42004,9708	4168,55412	0
170	-6,22256645	0,32760888	NA	NA	-0,00183217	NA	-0,66370241	-3,73836005	NA	0,05777952	NA	NA	NA	NA	7	-20995,9287	42005,8637	4169,44693	0
3140	-12,0135881	0,70944129	0,06178489	NA	NA	NA	NA	0,7406183	-0,11449835	NA	NA	NA	0,46966671	-0,53963709	8	-20995,2707	42006,5494	4170,13264	0
3588	-12,0135881	0,70944129	0,06178489	NA	NA	NA	NA	0,95852238	NA	NA	NA	-0,00035257	0,44577019	-0,55202249	8	-20995,4696	42006,9471	4170,53034	0
1748	-13,7669049	0,65178191	0,06462958	NA	NA	0,35119819	NA	-3,6371906	-0,09814646	0,05972263	NA	-0,00047389	0,39127251	NA	10	-20993,6241	42007,2603	4170,84355	0
676	-9,79595589	0,69445228	0,04389505	NA	NA	NA	-0,55018722	-3,52383686	NA	0,05863003	NA	-0,00043937	NA	NA	8	-20995,7135	42007,4349	4171,01819	0
1740	-11,9654714	0,51367836	0,06255183	NA	-0,0013444	NA	NA	-3,6371906	-0,09814646	0,05972263	NA	-0,00047389	0,39127251	NA	10	-20993,8126	42007,6374	4171,2207	0
234	-6,22256645	0,32760888	NA	NA	-0,00183217	NA	-0,66370241	-3,74999095	0,01316736	0,05794033	NA	NA	NA	NA	8	-20995,8662	42007,7404	4171,3237	0
740	-9,79595589	0,69445228	0,04389505	NA	NA	NA	-0,55018722	-3,4425274	-0,05282396	0,05811259	NA	-0,00050954	NA	NA	9	-20994,8758	42007,7615	4171,34476	0
2154	-6,22256645	0,32760888	NA	NA	-0,00183217	NA	-0,66370241	0,6960215	-0,05271384	NA	NA	NA	NA	-0,49283909	8	-20996,3815	42008,771	4172,35424	0
2090	-6,22256645	0,32760888	NA	NA	-0,00183217	NA	-0,66370241	0,6894459	NA	NA	NA	NA	NA	-0,49566768	7	-20997,4061	42008,8185	4172,40176	0
2596	-9,79595589	0,69445228	0,04389505	NA	NA	NA	-0,55018722	0,98389174	NA	NA	NA	-0,00044751	NA	-0,50601759	8	-20996,8948	42009,7976	4173,3809	0
1684	-13,7669049	0,65178191	0,06462958	NA	NA	0,35119819	NA	-3,78227707	NA	0,0606519	NA	-0,00034645	0,38158141	NA	9	-20996,4603	42010,9305	4174,51382	0
1676	-11,9654714	0,51367836	0,06255183	NA	-0,0013444	NA	NA	-3,78227707	NA	0,0606519	NA	-0,00034645	0,38158141	NA	9	-20996,6489	42011,3077	4174,89097	0
690	-8,51824578	0,51124721	NA	NA	NA	0,38535796	-0,65213053	-3,52383686	NA	0,05863003	NA	-0,00043937	NA	NA	8	-20997,7004	42011,4088	4174,9921	0
681	-3,98530292	NA	NA	NA	-0,00234183	NA	-0,67333606	-3,52383686	NA	0,05863003	NA	-0,00043937	NA	NA	7	-20998,8459	42011,6981	4175,28137	0
754	-8,51824578	0,51124721	NA	NA	NA	0,38535796	-0,65213053	-3,4425274	-0,05282396	0,05811259	NA	-0,00050954	NA	NA	9	-20996,8627	42011,7354	4175,31867	0
745	-3,98530292	NA	NA	NA	-0,00234183	NA	-0,67333606	-3,4425274	-0,05282396	0,05811259	NA	-0,00050954	NA	NA	8	-20998,0082	42012,0244	4175,60771	0
1763	-9,52399274	NA	0,02470059	NA	NA	NA	-0,56950148	-3,6371906	-0,09814646	0,05972263	NA	-0,00047389	0,39127251	NA	9	-20997,4092	42012,8283	4176,41155	0

2610	-8,51824578	0,51124721	NA	NA	NA	0,38535796	-0,65213053	0,98389174	NA	NA	NA	-0,00044751	NA	-0,50601759	8	-20998,8818	42013,7715	4177,35481	0
3076	-12,0135881	0,70944129	0,06178489	NA	NA	NA	NA	0,72537252	NA	NA	NA	NA	0,45346103	-0,54414773	7	-20999,9836	42013,9734	4177,5567	0
2601	-3,98530292	NA	NA	NA	-0,00234183	NA	-0,67333606	0,98389174	NA	NA	NA	-0,00044751	NA	-0,50601759	7	-21000,0273	42014,0608	4177,64408	0
1755	-11,3659105	NA	0,05744064	NA	-0,00198557	0,38445946	NA	-3,6371906	-0,09814646	0,05972263	NA	-0,00047389	0,39127251	NA	10	-20998,2347	42016,4815	4180,06478	0
1699	-9,52399274	NA	0,02470059	NA	NA	NA	-0,56950148	-3,78227707	NA	0,0606519	NA	-0,00034645	0,38158141	NA	8	-21000,2454	42016,4988	4180,08204	0
1172	-13,7669049	0,65178191	0,06462958	NA	NA	0,35119819	NA	-3,95454473	NA	0,05999672	NA	NA	0,38996522	NA	8	-21000,8034	42017,6148	4181,19813	0
1164	-11,9654714	0,51367836	0,06255183	NA	-0,0013444	NA	NA	-3,95454473	NA	0,05999672	NA	NA	0,38996522	NA	8	-21000,992	42017,992	4181,57527	0
1236	-13,7669049	0,65178191	0,06462958	NA	NA	0,35119819	NA	-3,92430325	-0,03654601	0,05956532	NA	NA	0,39466847	NA	9	-21000,3323	42018,6746	4182,25784	0
1228	-11,9654714	0,51367836	0,06255183	NA	-0,0013444	NA	NA	-3,92430325	-0,03654601	0,05956532	NA	NA	0,39466847	NA	9	-21000,5209	42019,0517	4182,63498	0
164	-9,79595589	0,69445228	0,04389505	NA	NA	NA	-0,55018722	-3,73836005	NA	0,05777952	NA	NA	NA	NA	7	-21002,6674	42019,3409	4182,92422	0
1691	-11,3659105	NA	0,05744064	NA	-0,00198557	0,38445946	NA	-3,78227707	NA	0,0606519	NA	-0,00034645	0,38158141	NA	9	-21001,0709	42020,1518	4183,73505	0
228	-9,79595589	0,69445228	0,04389505	NA	NA	NA	-0,55018722	-3,74999095	0,01316736	0,05794033	NA	NA	NA	NA	8	-21002,6049	42021,2177	4184,80099	0
615	-0,65771633	NA	-0,01187559	4,14E-05	NA	NA	-0,10732692	-0,66565225	-0,15004816	NA	NA	-0,00048657	NA	NA	8	-21003,0388	42022,0856	4185,66884	0
2148	-9,79595589	0,69445228	0,04389505	NA	NA	NA	-0,55018722	0,6960215	-0,05271384	NA	NA	NA	NA	-0,49283909	8	-21003,1201	42022,2483	4185,83153	0
2084	-9,79595589	0,69445228	0,04389505	NA	NA	NA	-0,55018722	0,6894459	NA	NA	NA	NA	NA	-0,49566768	7	-21004,1448	42022,2958	4185,87905	0
3147	-9,45532716	NA	0,05329757	NA	-0,00210061	NA	NA	0,7406183	-0,11449835	NA	NA	NA	0,46966671	-0,53963709	8	-21003,1499	42022,3077	4185,89096	0
3595	-9,45532716	NA	0,05329757	NA	-0,00210061	NA	NA	0,95852238	NA	NA	NA	-0,00035257	0,44577019	-0,55202249	8	-21003,3487	42022,7054	4186,28867	0
1187	-9,52399274	NA	0,02470059	NA	NA	NA	-0,56950148	-3,95454473	NA	0,05999672	NA	NA	0,38996522	NA	7	-21004,5885	42023,1833	4186,76657	0
178	-8,51824578	0,51124721	NA	NA	NA	0,38535796	-0,65213053	-3,73836005	NA	0,05777952	NA	NA	NA	NA	7	-21004,6543	42023,3148	4186,89813	0
613	-1,22822825	NA	NA	4,02E-05	NA	NA	-0,09995451	-0,66565225	-0,15004816	NA	NA	-0,00048657	NA	NA	7	-21004,7832	42023,5727	4187,15597	0
169	-3,98530292	NA	NA	NA	-0,00234183	NA	-0,67333606	-3,73836005	NA	0,05777952	NA	NA	NA	NA	6	-21005,7998	42023,6043	4187,18762	0
1251	-9,52399274	NA	0,02470059	NA	NA	NA	-0,56950148	-3,92430325	-0,03654601	0,05956532	NA	NA	0,39466847	NA	8	-21004,1174	42024,2428	4187,82606	0
583	-1,14142692	NA	-0,01089562	4,31E-05	NA	NA	NA	-0,66565225	-0,15004816	NA	NA	-0,00048657	NA	NA	7	-21005,1529	42024,312	4187,89528	0
1761	-8,58193391	NA	NA	NA	NA	NA	-0,61393687	-3,6371906	-0,09814646	0,05972263	NA	-0,00047389	0,39127251	NA	8	-21004,4252	42024,8584	4188,44172	0
242	-8,51824578	0,51124721	NA	NA	NA	0,38535796	-0,65213053	-3,74999095	0,01316736	0,05794033	NA	NA	NA	NA	8	-21004,5918	42025,1916	4188,7749	0
581	-1,63084607	NA	NA	4,18E-05	NA	NA	NA	-0,66565225	-0,15004816	NA	NA	-0,00048657	NA	NA	6	-21006,6281	42025,2608	4188,84409	0
233	-3,98530292	NA	NA	NA	-0,00234183	NA	-0,67333606	-3,74999095	0,01316736	0,05794033	NA	NA	NA	NA	7	-21005,7373	42025,4809	4189,06416	0
2162	-8,51824578	0,51124721	NA	NA	NA	0,38535796	-0,65213053	0,6960215	-0,05271384	NA	NA	NA	NA	-0,49283909	8	-21005,1071	42026,2222	4189,80544	0
2098	-8,51824578	0,51124721	NA	NA	NA	0,38535796	-0,65213053	0,6894459	NA	NA	NA	NA	NA	-0,49566768	7	-21006,1317	42026,2697	4189,85295	0
2153	-3,98530292	NA	NA	NA	-0,00234183	NA	-0,67333606	0,6960215	-0,05271384	NA	NA	NA	NA	-0,49283909	7	-21006,2526	42026,5114	4190,0947	0
2089	-3,98530292	NA	NA	NA	-0,00234183	NA	-0,67333606	0,6894459	NA	NA	NA	NA	NA	-0,49566768	6	-21007,2773	42026,5592	4190,14244	0
1179	-11,3659105	NA	0,05744064	NA	-0,00198557	0,38445946	NA	-3,95454473	NA	0,05999672	NA	NA	0,38996522	NA	8	-21005,4141	42026,8361	4190,41935	0
2177	-15,360872	NA	NA	NA	NA	NA	NA	-1,98285198	NA	0,054624	NA	NA	NA	-0,47270606	5	-21008,8354	42027,6741	4191,25741	0
3674	-8,97566397	0,33138274	NA	NA	-0,0012458	0,28352203	NA	1,15818376	-0,19528017	NA	NA	-0,00061584	0,46809624	-0,5501339	10	-21003,8693	42027,7508	4191,33404	0
1243	-11,3659105	NA	0,05744064	NA	-0,00198557	0,38445946	NA	-3,92430325	-0,03654601	0,05956532	NA	NA	0,39466847	NA	9	-21004,9429	42027,8958	4191,47906	0
2241	-15,360872	NA	NA	NA	NA	NA	NA	-2,01223022	0,04141198	0,05511629	NA	NA	NA	-0,47466077	6	-21008,2288	42028,4623	4192,04556	0
1697	-8,58193391	NA	NA	NA	NA	NA	-0,61393687	-3,78227707	NA	0,0606519	NA	-0,00034645	0,38158141	NA	7	-21007,2615	42028,5292	4192,11243	0
3083	-9,45532716	NA	0,05329757	NA	-0,00210061	NA	NA	0,72537252	NA	NA	NA	NA	0,45346103	-0,54414773	7	-21007,8628	42029,7317	4193,31503	0
551	-0,65771633	NA	-0,01187559	4,14E-05	NA	NA	-0,10732692	-0,82885018	NA	NA	NA	-0,0002841	NA	NA	7	-21010,0497	42034,1057	4197,68895	0
103	-0,65771633	NA	-0,01187559	4,14E-05	NA	NA	-0,10732692	-0,96744453	-0,08610767	NA	NA	NA	NA	NA	7	-21010,3197	42034,6457	4198,22896	0
1185	-8,58193391	NA	NA	NA	NA	NA	-0,61393687	-3,95454473	NA	0,05999672	NA	NA	0,38996522	NA	6	-21011,6046	42035,2139	4198,79718	0
1732	-12,0135881	0,70944129	0,06178489	NA	NA	NA	NA	-3,6371906	-0,09814646	0,05972263	NA	-0,00047389	0,39127251	NA	9	-21008,711	42035,432	4199,01532	0
549	-1,22822825	NA	NA	4,02E-05	NA	NA	-0,09995451	-0,82885018	NA	NA	NA	-0,0002841	NA	NA	6	-21011,7942	42035,593	4199,1763	0
101	-1,22822825	NA	NA	4,02E-05	NA	NA	-0,09995451	-0,96744453	-0,08610767	NA	NA	NA	NA	NA	6	-21012,0642	42036,133	4199,71631	0
1249	-8,58193391	NA	NA	NA	NA	NA	-0,61393687	-3,92430325	-0,03654601	0,05956532	NA	NA	0,39466847	NA	7	-21011,1335	42036,2732	4199,85645	0
519	-1,14142692	NA	-0,01089562	4,31E-05	NA	NA	NA	-0,82885018	NA	NA	NA	-0,0002841	NA	NA	6	-21012,1638	42036,3323	4199,91561	0
71	-1,14142692	NA	-0,01089562	4,31E-05	NA	NA	NA	-0,96744453	-0,08610767	NA	NA	NA	NA	NA	6	-21012,4338	42036,8723	4200,45561	0
517	-1,63084607	NA	NA	4,18E-05	NA	NA	NA	-0,82885018	NA	NA	NA	-0,0002841	NA	NA	5	-21013,639	42037,2814	4200,86464	0
69	-1,63084607	NA	NA	4,18E-05	NA	NA	NA	-0,96744453	-0,08610767	NA	NA	NA	NA	NA	5	-21013,909	42037,8214	4201,40465	0
39	-0,65771633	NA	-0,01187559	4,14E-05	NA	NA	-0,10732692	-0,99369134	NA	NA	NA	NA	NA	NA	6	-21013,1132	42038,231	4201,81432	0
2658	-7,20552725	0,58574016	NA	NA	NA	NA	-0,62020516	1,12236839	-0,1349839	NA	NA	-0,00063299	NA	-0,50295006	8	-21011,3622	42038,7324	4202,3157	0
1668	-12,0135881	0,70944129	0,06178489	NA	NA	NA	NA	-3,78227707	NA	0,0606519	NA	-0,00034645	0,38158141	NA	8	-21011,5473	42039,1025	4202,68581	0
37	-1,22822825	NA	NA	4,02E-05	NA	NA	-0,09995451	-0,99369134	NA	NA	NA	NA	NA	NA	5	-21014,8576	42039,7186	4203,30189	0
7	-1,14142692	NA	-0,01089562	4,31E-05	NA	NA	NA	-0,99369134	NA	NA	NA	NA	NA	NA	5	-21015,2273	42040,4579	4204,0412	0
5	-1,63084607	NA	NA	4,18E-05	NA	NA	NA	-0,99369134	NA	NA	NA	NA	NA	NA	4	-21016,7025	42041,4072	4204,99045	0
3673	-7,52922856	NA	NA	NA	-0,00175017	0,32067571	NA	1,15818376	-0,19528017	NA	NA	-0,00061584	0,46809624	-0,5501339	9	-21013,6948	42045,3995	4208,98281	0
674	-7,20552725	0,58574016	NA	NA	NA	NA	-0,62020516	-3,52383686	NA	0,05863003	NA	-0,00043937	NA	NA	7	-21015,7345	42045,4752	4209,05843	0
3658	-7,76791863	0,37580763	NA	NA	-0,00127907	NA	NA	1,15818376	-0,19528017	NA	NA	-0,00061584	0,46809624	-0,5501339	9	-21013,7579	42045,5257	4209,10901	0
1156	-12,0135881	0,70944129	0,06178489	NA	NA	NA	NA	-3,95454473	NA	0,05999672	NA	NA	0,38996522	NA	7	-21015,8904	42045,7871	4209,37033	0
738	-7,20552725	0,58574016	NA	NA	NA	NA	-0,62020516	-3,4425274	-0,05282396	0,05811259	NA	-0,00050954	NA	NA	8	-21014,8968	42045,8015	4209,38478	0
2675	-8,8387814	NA	0,03010563	NA	NA	0,51295904	-0,60572616	1,12236839	-0,1349839	NA	NA	-0,00063299	NA	-0,50295006	9	-21014,3789	42046,7677	4210,351	0

1220	-12,0135881	0,70944129	0,06178489	NA	NA	NA	NA	-3,92430325	-0,03654601	0,05956532	NA	NA	0,39466847	NA	8	-21015,4193	42046,8465	4210,42982	0
2594	-7,20552725	0,58574016	NA	NA	NA	NA	-0,62020516	0,98389174	NA	NA	NA	-0,00044751	NA	-0,50601759	7	-21016,9158	42047,8379	4211,42114	0
3162	-8,97566397	0,33138274	NA	NA	-0,0012458	0,28352203	NA	0,7406183	-0,11449835	NA	NA	NA	0,46966671	-0,53963709	9	-21015,004	42048,018	4211,60132	0
3610	-8,97566397	0,33138274	NA	NA	-0,0012458	0,28352203	NA	0,95852238	NA	NA	NA	-0,00035257	0,44577019	-0,55202249	9	-21015,2029	42048,4157	4211,99902	0
3666	-8,08714822	0,50972296	NA	NA	NA	0,2963004	NA	1,15818376	-0,19528017	NA	NA	-0,00061584	0,46809624	-0,5501339	9	-21016,5657	42051,1413	4214,72458	0
1739	-9,45532716	NA	0,05329757	NA	-0,00210061	NA	NA	-3,6371906	-0,09814646	0,05972263	NA	-0,00047389	0,39127251	NA	9	-21016,5902	42051,1904	4214,77364	0
3667	-11,2963041	NA	0,04958538	NA	NA	0,45107925	NA	1,15818376	-0,19528017	NA	NA	-0,00061584	0,46809624	-0,5501339	9	-21016,9414	42051,8927	4215,47598	0
691	-8,8387814	NA	0,03010563	NA	NA	0,51295904	-0,60572616	-3,52383686	NA	0,05863003	NA	-0,00043937	NA	NA	8	-21018,7511	42053,5102	4217,09351	0
755	-8,8387814	NA	0,03010563	NA	NA	0,51295904	-0,60572616	-3,4425274	-0,05282396	0,05811259	NA	-0,00050954	NA	NA	9	-21017,9134	42053,8368	4217,42008	0
1675	-9,45532716	NA	0,05329757	NA	-0,00210061	NA	NA	-3,78227707	NA	0,0606519	NA	-0,00034645	0,38158141	NA	8	-21019,4264	42054,8609	4218,44413	0
3098	-8,97566397	0,33138274	NA	NA	-0,0012458	0,28352203	NA	0,72537252	NA	NA	NA	NA	0,45346103	-0,54414773	8	-21019,717	42055,4419	4219,02516	0
2611	-8,8387814	NA	0,03010563	NA	NA	0,51295904	-0,60572616	0,98389174	NA	NA	NA	-0,00044751	NA	-0,50601759	8	-21019,9325	42055,8729	4219,45622	0
162	-7,20552725	0,58574016	NA	NA	NA	NA	-0,62020516	-3,73836005	NA	0,05777952	NA	NA	NA	NA	6	-21022,6884	42057,3814	4220,96468	0
226	-7,20552725	0,58574016	NA	NA	NA	NA	-0,62020516	-3,74999095	0,01316736	0,05794033	NA	NA	NA	NA	7	-21022,6259	42059,2579	4222,84123	0
2146	-7,20552725	0,58574016	NA	NA	NA	NA	-0,62020516	0,6960215	-0,05271384	NA	NA	NA	NA	-0,49283909	7	-21023,1411	42060,2885	4223,87177	0
2082	-7,20552725	0,58574016	NA	NA	NA	NA	-0,62020516	0,6894459	NA	NA	NA	NA	NA	-0,49566768	6	-21024,1658	42060,3362	4223,91951	0
1163	-9,45532716	NA	0,05329757	NA	-0,00210061	NA	NA	-3,95454473	NA	0,05999672	NA	NA	0,38996522	NA	7	-21023,7696	42061,5454	4225,12866	0
1227	-9,45532716	NA	0,05329757	NA	-0,00210061	NA	NA	-3,92430325	-0,03654601	0,05956532	NA	NA	0,39466847	NA	8	-21023,2985	42062,6049	4226,18815	0
179	-8,8387814	NA	0,03010563	NA	NA	0,51295904	-0,60572616	-3,73836005	NA	0,05777952	NA	NA	NA	NA	7	-21025,705	42065,4163	4228,99954	0
2673	-6,3315051	NA	NA	NA	NA	0,4838605	-0,6580268	1,12236839	-0,1349839	NA	NA	-0,00063299	NA	-0,50295006	8	-21024,7934	42065,5948	4229,17808	0
3161	-7,52922856	NA	NA	NA	-0,00175017	0,32067571	NA	0,7406183	-0,11449835	NA	NA	NA	0,46966671	-0,53963709	8	-21024,8295	42065,667	4229,25032	0
3146	-7,76791863	0,37580763	NA	NA	-0,00127907	NA	NA	0,7406183	-0,11449835	NA	NA	NA	0,46966671	-0,53963709	8	-21024,8926	42065,7932	4229,37651	0
3609	-7,52922856	NA	NA	NA	-0,00175017	0,32067571	NA	0,95852238	NA	NA	NA	-0,00035257	0,44577019	-0,55202249	8	-21025,0284	42066,0647	4229,64802	0
3594	-7,76791863	0,37580763	NA	NA	-0,00127907	NA	NA	0,95852238	NA	NA	NA	-0,00035257	0,44577019	-0,55202249	8	-21025,0915	42066,1909	4229,77422	0
243	-8,8387814	NA	0,03010563	NA	NA	0,51295904	-0,60572616	-3,74999095	0,01316736	0,05794033	NA	NA	NA	NA	8	-21025,6425	42067,293	4230,87631	0
2163	-8,8387814	NA	0,03010563	NA	NA	0,51295904	-0,60572616	0,6960215	-0,05271384	NA	NA	NA	NA	-0,49283909	8	-21026,1578	42068,3236	4231,90685	0
2099	-8,8387814	NA	0,03010563	NA	NA	0,51295904	-0,60572616	0,6894459	NA	NA	NA	NA	NA	-0,49566768	7	-21027,1824	42068,3711	4231,95436	0
3657	-6,18359995	NA	NA	NA	-0,00186372	NA	NA	1,15818376	-0,19528017	NA	NA	-0,00061584	0,46809624	-0,5501339	8	-21026,6088	42069,2256	4232,80887	0
3650	-10,126072	0,56333515	NA	NA	NA	NA	NA	1,15818376	-0,19528017	NA	NA	-0,00061584	0,46809624	-0,5501339	8	-21027,3613	42070,7305	4234,31376	0
3154	-8,08714822	0,50972296	NA	NA	NA	0,2963004	NA	0,7406183	-0,11449835	NA	NA	NA	0,46966671	-0,53963709	8	-21027,7004	42071,4088	4234,99208	0
3602	-8,08714822	0,50972296	NA	NA	NA	0,2963004	NA	0,95852238	NA	NA	NA	-0,00035257	0,44577019	-0,55202249	8	-21027,8993	42071,8065	4235,38979	0
3155	-11,2963041	NA	0,04958538	NA	NA	0,45107925	NA	0,7406183	-0,11449835	NA	NA	NA	0,46966671	-0,53963709	8	-21028,0761	42072,1602	4235,74349	0
689	-6,3315051	NA	NA	NA	NA	0,4838605	-0,6580268	-3,52383686	NA	0,05863003	NA	-0,00043937	NA	NA	7	-21029,1657	42072,3375	4235,92081	0
3603	-11,2963041	NA	0,04958538	NA	NA	0,45107925	NA	0,95852238	NA	NA	NA	-0,00035257	0,44577019	-0,55202249	8	-21028,275	42072,5579	4236,14119	0
753	-6,3315051	NA	NA	NA	NA	0,4838605	-0,6580268	-3,4425274	-0,05282396	0,05811259	NA	-0,00050954	NA	NA	8	-21028,328	42072,6639	4236,24716	0
3097	-7,52922856	NA	NA	NA	-0,00175017	0,32067571	NA	0,72537252	NA	NA	NA	NA	0,45346103	-0,54414773	7	-21029,5425	42073,0911	4236,67438	0
3082	-7,76791863	0,37580763	NA	NA	-0,00127907	NA	NA	0,72537252	NA	NA	NA	NA	0,45346103	-0,54414773	7	-21029,6056	42073,2173	4236,80058	0
2609	-6,3315051	NA	NA	NA	NA	0,4838605	-0,6580268	0,98389174	NA	NA	NA	-0,00044751	NA	-0,50601759	7	-21030,347	42074,7002	4238,28352	0
1754	-8,97566397	0,33138274	NA	NA	-0,0012458	0,28352203	NA	-3,6371906	-0,09814646	0,05972263	NA	-0,00047389	0,39127251	NA	10	-21028,4444	42076,9009	4240,48422	0
3090	-8,08714822	0,50972296	NA	NA	NA	0,2963004	NA	0,72537252	NA	NA	NA	NA	0,45346103	-0,54414773	7	-21032,4133	42078,8329	4242,41615	0
2652	-9,61919161	0,46601352	0,06534536	NA	-0,00131078	0,33938341	NA	1,12236839	-0,1349839	NA	NA	-0,00063299	NA	-0,50295006	10	-21029,7511	42079,5145	4243,09774	0
3091	-11,2963041	NA	0,04958538	NA	NA	0,45107925	NA	0,72537252	NA	NA	NA	NA	0,45346103	-0,54414773	7	-21032,789	42079,5843	4243,16755	0
1690	-8,97566397	0,33138274	NA	NA	-0,0012458	0,28352203	NA	-3,78227707	NA	0,0606519	NA	-0,00034645	0,38158141	NA	9	-21031,2806	42080,5712	4244,15449	0
177	-6,3315051	NA	NA	NA	NA	0,4838605	-0,6580268	-3,73836005	NA	0,05777952	NA	NA	NA	NA	6	-21036,1196	42084,2438	4247,82706	0
241	-6,3315051	NA	NA	NA	NA	0,4838605	-0,6580268	-3,74999095	0,01316736	0,05794033	NA	NA	NA	NA	7	-21036,0571	42086,1203	4249,70361	0
668	-9,61919161	0,46601352	0,06534536	NA	-0,00131078	0,33938341	NA	-3,52383686	NA	0,05863003	NA	-0,00043937	NA	NA	9	-21034,1234	42086,2567	4249,84002	0
732	-9,61919161	0,46601352	0,06534536	NA	-0,00131078	0,33938341	NA	-3,4425274	-0,05282396	0,05811259	NA	-0,00050954	NA	NA	10	-21033,2857	42086,5835	4250,16681	0
2161	-6,3315051	NA	NA	NA	NA	0,4838605	-0,6580268	0,6960215	-0,05271384	NA	NA	NA	NA	-0,49283909	7	-21036,5723	42087,1509	4250,73415	0
2097	-6,3315051	NA	NA	NA	NA	0,4838605	-0,6580268	0,6894459	NA	NA	NA	NA	NA	-0,49566768	6	-21037,597	42087,1986	4250,78189	0
1178	-8,97566397	0,33138274	NA	NA	-0,0012458	0,28352203	NA	-3,95454473	NA	0,05999672	NA	NA	0,38996522	NA	8	-21035,6238	42087,2555	4250,83879	0
1242	-8,97566397	0,33138274	NA	NA	-0,0012458	0,28352203	NA	-3,92430325	-0,03654601	0,05956532	NA	NA	0,39466847	NA	9	-21035,1526	42088,3152	4251,89851	0
2588	-9,61919161	0,46601352	0,06534536	NA	-0,00131078	0,33938341	NA	0,98389174	NA	NA	NA	-0,00044751	NA	-0,50601759	9	-21035,3048	42088,6195	4252,20273	0
3145	-6,18359995	NA	NA	NA	-0,00186372	NA	NA	0,7406183	-0,11449835	NA	NA	NA	0,46966671	-0,53963709	7	-21037,7436	42089,4933	4253,07659	0
3593	-6,18359995	NA	NA	NA	-0,00186372	NA	NA	0,95852238	NA	NA	NA	-0,00035257	0,44577019	-0,55202249	7	-21037,9424	42089,891	4253,4743	0
3138	-10,126072	0,56333515	NA	NA	NA	NA	NA	0,7406183	-0,11449835	NA	NA	NA	0,46966671	-0,53963709	7	-21038,496	42090,9982	4254,58149	0
3586	-10,126072	0,56333515	NA	NA	NA	NA	NA	0,95852238	NA	NA	NA	-0,00035257	0,44577019	-0,55202249	7	-21038,6949	42091,3959	4254,9792	0
1753	-7,52922856	NA	NA	NA	-0,00175017	0,32067571	NA	-3,6371906	-0,09814646	0,05972263	NA	-0,00047389	0,39127251	NA	9	-21038,2699	42094,5497	4258,133	0
1738	-7,76791863	0,37580763	NA	NA	-0,00127907	NA	NA	-3,6371906	-0,09814646	0,05972263	NA	-0,00047389	0,39127251	NA	9	-21038,333	42094,6759	4258,25919	0
3081	-6,18359995	NA	NA	NA	-0,00186372	NA	NA	0,72537252	NA	NA	NA	NA	0,45346103	-0,54414773	6	-21042,4565	42096,9176	4260,50088	0
156	-9,61919161	0,46601352	0,06534536	NA	-0,00131078	0,33938341	NA	-3,73836005	NA	0,05777952	NA	NA	NA	NA	8	-21041,0773	42098,1625	4261,74583	0
1689	-7,52922856	NA	NA	NA	-0,00175017	0,32067571	NA	-3,78227707	NA	0,0606519	NA	-0,00034645	0,38158141	NA	8	-21041,1061	42098,2202	4261,80349	0

1674	-7,76791863	0,37580763	NA	NA	-0,00127907	NA	NA	-3,78227707	NA	0,0606519	NA	-0,00034645	0,38158141	NA	8	-21041,1692	42098,3464	4261,92968	0
3074	-10,126072	0,56333515	NA	NA	NA	NA	NA	0,72537252	NA	NA	NA	NA	0,45346103	-0,54414773	6	-21043,2089	42098,4225	4262,00578	0
220	-9,61919161	0,46601352	0,06534536	NA	-0,00131078	0,33938341	NA	-3,74999095	0,01316736	0,05794033	NA	NA	NA	NA	9	-21041,0148	42100,0395	4263,62282	0
1746	-8,08714822	0,50972296	NA	NA	NA	0,2963004	NA	-3,6371906	-0,09814646	0,05972263	NA	-0,00047389	0,39127251	NA	9	-21041,1408	42100,2915	4263,87476	0
1747	-11,2963041	NA	0,04958538	NA	NA	0,45107925	NA	-3,6371906	-0,09814646	0,05972263	NA	-0,00047389	0,39127251	NA	9	-21041,5165	42101,0429	4264,62616	0
2140	-9,61919161	0,46601352	0,06534536	NA	-0,00131078	0,33938341	NA	0,6960215	-0,05271384	NA	NA	NA	NA	-0,49283909	9	-21041,5301	42101,0701	4264,65336	0
2076	-9,61919161	0,46601352	0,06534536	NA	-0,00131078	0,33938341	NA	0,6894459	NA	NA	NA	NA	NA	-0,49566768	8	-21042,5547	42101,1174	4264,70066	0
3651	-11,7728913	NA	0,04392487	NA	NA	NA	NA	1,15818376	-0,19528017	NA	NA	-0,00061584	0,46809624	-0,5501339	8	-21042,5638	42101,1355	4264,71877	0
1682	-8,08714822	0,50972296	NA	NA	NA	0,2963004	NA	-3,78227707	NA	0,0606519	NA	-0,00034645	0,38158141	NA	8	-21043,977	42103,962	4267,54525	0
1683	-11,2963041	NA	0,04958538	NA	NA	0,45107925	NA	-3,78227707	NA	0,0606519	NA	-0,00034645	0,38158141	NA	8	-21044,3527	42104,7134	4268,29666	0
1177	-7,52922856	NA	NA	NA	-0,00175017	0,32067571	NA	-3,95454473	NA	0,05999672	NA	NA	0,38996522	NA	7	-21045,4493	42104,9047	4268,48801	0
1162	-7,76791863	0,37580763	NA	NA	-0,00127907	NA	NA	-3,95454473	NA	0,05999672	NA	NA	0,38996522	NA	7	-21045,5124	42105,0309	4268,61421	0
2644	-13,7669049	0,65178191	0,06462958	NA	NA	0,35119819	NA	1,12236839	-0,1349839	NA	NA	-0,00063299	NA	-0,50295006	9	-21043,6335	42105,2769	4268,86014	0
2636	-11,9654714	0,51367836	0,06255183	NA	-0,0013444	NA	NA	1,12236839	-0,1349839	NA	NA	-0,00063299	NA	-0,50295006	9	-21043,822	42105,654	4269,23728	0
1241	-7,52922856	NA	NA	NA	-0,00175017	0,32067571	NA	-3,92430325	-0,03654601	0,05956532	NA	NA	0,39466847	NA	8	-21044,9781	42105,9642	4269,5475	0
1226	-7,76791863	0,37580763	NA	NA	-0,00127907	NA	NA	-3,92430325	-0,03654601	0,05956532	NA	NA	0,39466847	NA	8	-21045,0412	42106,0904	4269,6737	0
1170	-8,08714822	0,50972296	NA	NA	NA	0,2963004	NA	-3,95454473	NA	0,05999672	NA	NA	0,38996522	NA	7	-21048,3202	42110,6465	4274,22978	0
2659	-9,52399274	NA	0,02470059	NA	NA	NA	-0,56950148	1,12236839	-0,1349839	NA	NA	-0,00063299	NA	-0,50295006	8	-21047,4186	42110,8451	4274,42836	0
1171	-11,2963041	NA	0,04958538	NA	NA	0,45107925	NA	-3,95454473	NA	0,05999672	NA	NA	0,38996522	NA	7	-21048,6959	42111,3979	4274,98118	0
3665	-9,09446706	NA	NA	NA	NA	0,38722306	NA	1,15818376	-0,19528017	NA	NA	-0,00061584	0,46809624	-0,5501339	8	-21047,6972	42111,4023	4274,98556	0
1234	-8,08714822	0,50972296	NA	NA	NA	0,2963004	NA	-3,92430325	-0,03654601	0,05956532	NA	NA	0,39466847	NA	8	-21047,849	42111,706	4275,28927	0
660	-13,7669049	0,65178191	0,06462958	NA	NA	0,35119819	NA	-3,52383686	NA	0,05863003	NA	-0,00043937	NA	NA	8	-21048,0057	42112,0194	4275,60265	0
724	-13,7669049	0,65178191	0,06462958	NA	NA	0,35119819	NA	-3,4425274	-0,05282396	0,05811259	NA	-0,00050954	NA	NA	9	-21047,168	42112,3459	4275,92922	0
652	-11,9654714	0,51367836	0,06255183	NA	-0,0013444	NA	NA	-3,52383686	NA	0,05863003	NA	-0,00043937	NA	NA	8	-21048,1943	42112,3965	4275,97979	0
1235	-11,2963041	NA	0,04958538	NA	NA	0,45107925	NA	-3,92430325	-0,03654601	0,05956532	NA	NA	0,39466847	NA	8	-21048,2247	42112,4574	4276,04067	0
716	-11,9654714	0,51367836	0,06255183	NA	-0,0013444	NA	NA	-3,4425274	-0,05282396	0,05811259	NA	-0,00050954	NA	NA	9	-21047,3566	42112,7231	4276,30636	0
2580	-13,7669049	0,65178191	0,06462958	NA	NA	0,35119819	NA	0,98389174	NA	NA	NA	-0,00044751	NA	-0,50601759	8	-21049,1871	42114,3821	4277,96536	0
2651	-11,3659105	NA	0,05744064	NA	-0,00198557	0,38445946	NA	1,12236839	-0,1349839	NA	NA	-0,00063299	NA	-0,50295006	9	-21048,2441	42114,4981	4278,08136	0
2572	-11,9654714	0,51367836	0,06255183	NA	-0,0013444	NA	NA	0,98389174	NA	NA	NA	-0,00044751	NA	-0,50601759	8	-21049,3756	42114,7592	4278,3425	0
675	-9,52399274	NA	0,02470059	NA	NA	NA	-0,56950148	-3,52383686	NA	0,05863003	NA	-0,00043937	NA	NA	7	-21051,7908	42117,5878	4281,17109	0
739	-9,52399274	NA	0,02470059	NA	NA	NA	-0,56950148	-3,4425274	-0,05282396	0,05811259	NA	-0,00050954	NA	NA	8	-21050,9531	42117,9142	4281,49743	0
1737	-6,18359995	NA	NA	NA	-0,00186372	NA	NA	-3,6371906	-0,09814646	0,05972263	NA	-0,00047389	0,39127251	NA	8	-21051,1839	42118,3758	4281,95905	0
1730	-10,126072	0,56333515	NA	NA	NA	NA	NA	-3,6371906	-0,09814646	0,05972263	NA	-0,00047389	0,39127251	NA	8	-21051,9364	42119,8807	4283,46395	0
2595	-9,52399274	NA	0,02470059	NA	NA	NA	-0,56950148	0,98389174	NA	NA	NA	-0,00044751	NA	-0,50601759	7	-21052,9722	42119,9505	4283,5338	0
667	-11,3659105	NA	0,05744064	NA	-0,00198557	0,38445946	NA	-3,52383686	NA	0,05863003	NA	-0,00043937	NA	NA	8	-21052,6163	42121,2406	4284,82387	0
3139	-11,7728913	NA	0,04392487	NA	NA	NA	NA	0,7406183	-0,11449835	NA	NA	NA	0,46966671	-0,53963709	7	-21053,6985	42121,4032	4284,98649	0
731	-11,3659105	NA	0,05744064	NA	-0,00198557	0,38445946	NA	-3,4425274	-0,05282396	0,05811259	NA	-0,00050954	NA	NA	9	-21051,7786	42121,5672	4285,15044	0
3587	-11,7728913	NA	0,04392487	NA	NA	NA	NA	0,95852238	NA	NA	NA	-0,00035257	0,44577019	-0,55202249	7	-21053,8974	42121,8009	4285,3842	0
1673	-6,18359995	NA	NA	NA	-0,00186372	NA	NA	-3,78227707	NA	0,0606519	NA	-0,00034645	0,38158141	NA	7	-21054,0201	42122,0465	4285,62976	0
2657	-8,58193391	NA	NA	NA	NA	NA	-0,61393687	1,12236839	-0,1349839	NA	NA	-0,00063299	NA	-0,50295006	7	-21054,4346	42122,8755	4286,45875	0
1666	-10,126072	0,56333515	NA	NA	NA	NA	NA	-3,78227707	NA	0,0606519	NA	-0,00034645	0,38158141	NA	7	-21054,7726	42123,5514	4287,13466	0
2587	-11,3659105	NA	0,05744064	NA	-0,00198557	0,38445946	NA	0,98389174	NA	NA	NA	-0,00044751	NA	-0,50601759	8	-21053,7977	42123,6033	4287,18658	0
148	-13,7669049	0,65178191	0,06462958	NA	NA	0,35119819	NA	-3,73836005	NA	0,05777952	NA	NA	NA	NA	7	-21054,9596	42123,9254	4287,50867	0
140	-11,9654714	0,51367836	0,06255183	NA	-0,0013444	NA	NA	-3,73836005	NA	0,05777952	NA	NA	NA	NA	7	-21055,1482	42124,3025	4287,88582	0
212	-13,7669049	0,65178191	0,06462958	NA	NA	0,35119819	NA	-3,74999095	0,01316736	0,05794033	NA	NA	NA	NA	8	-21054,8971	42125,8022	4289,38545	0
204	-11,9654714	0,51367836	0,06255183	NA	-0,0013444	NA	NA	-3,74999095	0,01316736	0,05794033	NA	NA	NA	NA	8	-21055,0857	42126,1793	4289,76259	0
2132	-13,7669049	0,65178191	0,06462958	NA	NA	0,35119819	NA	0,6960215	-0,05271384	NA	NA	NA	NA	-0,49283909	8	-21055,4124	42126,8327	4290,41599	0
2068	-13,7669049	0,65178191	0,06462958	NA	NA	0,35119819	NA	0,6894459	NA	NA	NA	NA	NA	-0,49566768	7	-21056,437	42126,8802	4290,4635	0
2124	-11,9654714	0,51367836	0,06255183	NA	-0,0013444	NA	NA	0,6960215	-0,05271384	NA	NA	NA	NA	-0,49283909	8	-21055,6009	42127,2099	4290,79313	0
2060	-11,9654714	0,51367836	0,06255183	NA	-0,0013444	NA	NA	0,6894459	NA	NA	NA	NA	NA	-0,49566768	7	-21056,6256	42127,2574	4290,84065	0
1161	-6,18359995	NA	NA	NA	-0,00186372	NA	NA	-3,95454473	NA	0,05999672	NA	NA	0,38996522	NA	6	-21058,3633	42128,7312	4292,31451	0
3075	-11,7728913	NA	0,04392487	NA	NA	NA	NA	0,72537252	NA	NA	NA	NA	0,45346103	-0,54414773	6	-21058,4114	42128,8275	4292,41078	0
163	-9,52399274	NA	0,02470059	NA	NA	NA	-0,56950148	-3,73836005	NA	0,05777952	NA	NA	NA	NA	6	-21058,7447	42129,4941	4293,07734	0
673	-8,58193391	NA	NA	NA	NA	NA	-0,61393687	-3,52383686	NA	0,05863003	NA	-0,00043937	NA	NA	6	-21058,8069	42129,6184	4293,2017	0
1225	-6,18359995	NA	NA	NA	-0,00186372	NA	NA	-3,92430325	-0,03654601	0,05956532	NA	NA	0,39466847	NA	7	-21057,8922	42129,7905	4293,37378	0
737	-8,58193391	NA	NA	NA	NA	NA	-0,61393687	-3,4425274	-0,05282396	0,05811259	NA	-0,00050954	NA	NA	7	-21057,9692	42129,9445	4293,52783	0
1154	-10,126072	0,56333515	NA	NA	NA	NA	NA	-3,95454473	NA	0,05999672	NA	NA	0,38996522	NA	6	-21059,1157	42130,2361	4293,81941	0
1218	-10,126072	0,56333515	NA	NA	NA	NA	NA	-3,92430325	-0,03654601	0,05956532	NA	NA	0,39466847	NA	7	-21058,6446	42131,2954	4294,87868	0
227	-9,52399274	NA	0,02470059	NA	NA	NA	-0,56950148	-3,74999095	0,01316736	0,05794033	NA	NA	NA	NA	7	-21058,6822	42131,3706	4294,95388	0
3153	-9,09446706	NA	NA	NA	NA	0,38722306	NA	0,7406183	-0,11449835	NA	NA	NA	0,46966671	-0,53963709	7	-21058,8319	42131,67	4295,25328	0
2593	-8,58193391	NA	NA	NA	NA	NA	-0,61393687	0,98389174	NA	NA	NA	-0,00044751	NA	-0,50601759	6	-21059,9882	42131,9811	4295,56441	0

3601	-9,09446706	NA	NA	NA	NA	0,38722306	NA	0,95852238	NA	NA	NA	-0,00035257	0,44577019	-0,55202249	7	-21059,0308	42132,0677	4295,65099	0
2147	-9,52399274	NA	0,02470059	NA	NA	NA	-0,56950148	0,6960215	-0,05271384	NA	NA	NA	NA	-0,49283909	7	-21059,1975	42132,4011	4295,98443	0
2083	-9,52399274	NA	0,02470059	NA	NA	NA	-0,56950148	0,6894459	NA	NA	NA	NA	NA	-0,49566768	6	-21060,2221	42132,4489	4296,03216	0
155	-11,3659105	NA	0,05744064	NA	-0,00198557	0,38445946	NA	-3,73836005	NA	0,05777952	NA	NA	NA	NA	7	-21059,5702	42133,1466	4296,7299	0
2628	-12,0135881	0,70944129	0,06178489	NA	NA	NA	NA	1,12236839	-0,1349839	NA	NA	-0,00063299	NA	-0,50295006	8	-21058,7204	42133,4488	4297,03212	0
219	-11,3659105	NA	0,05744064	NA	-0,00198557	0,38445946	NA	-3,74999095	0,01316736	0,05794033	NA	NA	NA	NA	8	-21059,5077	42135,0234	4298,60667	0
2139	-11,3659105	NA	0,05744064	NA	-0,00198557	0,38445946	NA	0,6960215	-0,05271384	NA	NA	NA	NA	-0,49283909	8	-21060,023	42136,0539	4299,63721	0
2075	-11,3659105	NA	0,05744064	NA	-0,00198557	0,38445946	NA	0,6894459	NA	NA	NA	NA	NA	-0,49566768	7	-21061,0476	42136,1014	4299,68473	0
3089	-9,09446706	NA	NA	NA	NA	0,38722306	NA	0,72537252	NA	NA	NA	NA	0,45346103	-0,54414773	6	-21063,5448	42139,0943	4302,67757	0
644	-12,0135881	0,70944129	0,06178489	NA	NA	NA	NA	-3,52383686	NA	0,05863003	NA	-0,00043937	NA	NA	7	-21063,0927	42140,1916	4303,77485	0
708	-12,0135881	0,70944129	0,06178489	NA	NA	NA	NA	-3,4425274	-0,05282396	0,05811259	NA	-0,00050954	NA	NA	8	-21062,255	42140,5179	4304,1012	0
161	-8,58193391	NA	NA	NA	NA	NA	-0,61393687	-3,73836005	NA	0,05777952	NA	NA	NA	NA	5	-21065,7608	42141,5249	4305,10817	0
2564	-12,0135881	0,70944129	0,06178489	NA	NA	NA	NA	0,98389174	NA	NA	NA	-0,00044751	NA	-0,50601759	7	-21064,274	42142,5543	4306,13756	0
225	-8,58193391	NA	NA	NA	NA	NA	-0,61393687	-3,74999095	0,01316736	0,05794033	NA	NA	NA	NA	6	-21065,6983	42143,4012	4306,9845	0
2145	-8,58193391	NA	NA	NA	NA	NA	-0,61393687	0,6960215	-0,05271384	NA	NA	NA	NA	-0,49283909	6	-21066,2136	42144,4318	4308,01504	0
2081	-8,58193391	NA	NA	NA	NA	NA	-0,61393687	0,6894459	NA	NA	NA	NA	NA	-0,49566768	5	-21067,2382	42144,4797	4308,063	0
3649	-15,360872	NA	NA	NA	NA	NA	NA	1,15818376	-0,19528017	NA	NA	-0,00061584	0,46809624	-0,5501339	7	-21066,8393	42147,6848	4311,26806	0
2635	-9,45532716	NA	0,05329757	NA	-0,00210061	NA	NA	1,12236839	-0,1349839	NA	NA	-0,00063299	NA	-0,50295006	8	-21066,5996	42149,2072	4312,79045	0
1731	-11,7728913	NA	0,04392487	NA	NA	NA	NA	-3,6371906	-0,09814646	0,05972263	NA	-0,00047389	0,39127251	NA	8	-21067,1389	42150,2857	4313,86895	0
1660	-5,95790631	0,3741151	0,04613303	NA	-0,00180249	0,39660544	-0,62074526	-0,7738147	-0,19560429	NA	NA	-0,00045111	0,35589058	NA	11	-21064,3354	42150,6855	4314,26877	0
132	-12,0135881	0,70944129	0,06178489	NA	NA	NA	NA	-3,73836005	NA	0,05777952	NA	NA	NA	NA	6	-21070,0466	42152,0978	4315,6811	0
1667	-11,7728913	NA	0,04392487	NA	NA	NA	NA	-3,78227707	NA	0,0606519	NA	-0,00034645	0,38158141	NA	7	-21069,9751	42153,9564	4317,53966	0
196	-12,0135881	0,70944129	0,06178489	NA	NA	NA	NA	-3,74999095	0,01316736	0,05794033	NA	NA	NA	NA	7	-21069,9841	42153,9744	4317,55765	0
2116	-12,0135881	0,70944129	0,06178489	NA	NA	NA	NA	0,6960215	-0,05271384	NA	NA	NA	NA	-0,49283909	7	-21070,4994	42155,0049	4318,58819	0
2052	-12,0135881	0,70944129	0,06178489	NA	NA	NA	NA	0,6894459	NA	NA	NA	NA	NA	-0,49566768	6	-21071,524	42155,0527	4318,63593	0
651	-9,45532716	NA	0,05329757	NA	-0,00210061	NA	NA	-3,52383686	NA	0,05863003	NA	-0,00043937	NA	NA	7	-21070,9719	42155,9499	4319,53318	0
715	-9,45532716	NA	0,05329757	NA	-0,00210061	NA	NA	-3,4425274	-0,05282396	0,05811259	NA	-0,00050954	NA	NA	8	-21070,1341	42156,2762	4319,85952	0
2571	-9,45532716	NA	0,05329757	NA	-0,00210061	NA	NA	0,98389174	NA	NA	NA	-0,00044751	NA	-0,50601759	7	-21072,1532	42158,3126	4321,89589	0
1745	-9,09446706	NA	NA	NA	NA	0,38722306	NA	-3,6371906	-0,09814646	0,05972263	NA	-0,00047389	0,39127251	NA	8	-21072,2722	42160,5525	4324,13574	0
1155	-11,7728913	NA	0,04392487	NA	NA	NA	NA	-3,95454473	NA	0,05999672	NA	NA	0,38996522	NA	6	-21074,3182	42160,6411	4324,22441	0
1148	-5,95790631	0,3741151	0,04613303	NA	-0,00180249	0,39660544	-0,62074526	-1,05468211	-0,1361791	NA	NA	NA	0,35946641	NA	10	-21070,6223	42161,2567	4324,84001	0
1219	-11,7728913	NA	0,04392487	NA	NA	NA	NA	-3,92430325	-0,03654601	0,05956532	NA	NA	0,39466847	NA	7	-21073,8471	42161,7004	4325,28368	0
1681	-9,09446706	NA	NA	NA	NA	0,38722306	NA	-3,78227707	NA	0,0606519	NA	-0,00034645	0,38158141	NA	7	-21075,1085	42164,2232	4327,80645	0
139	-9,45532716	NA	0,05329757	NA	-0,00210061	NA	NA	-3,73836005	NA	0,05777952	NA	NA	NA	NA	6	-21077,9258	42167,8561	4331,43943	0
3137	-15,360872	NA	NA	NA	NA	NA	NA	0,7406183	-0,11449835	NA	NA	NA	0,46966671	-0,53963709	6	-21077,974	42167,9527	4331,53601	0
3585	-15,360872	NA	NA	NA	NA	NA	NA	0,95852238	NA	NA	NA	-0,00035257	0,44577019	-0,55202249	6	-21078,1729	42168,3504	4331,93372	0
203	-9,45532716	NA	0,05329757	NA	-0,00210061	NA	NA	-3,74999095	0,01316736	0,05794033	NA	NA	NA	NA	7	-21077,8633	42169,7327	4333,31598	0
2123	-9,45532716	NA	0,05329757	NA	-0,00210061	NA	NA	0,6960215	-0,05271384	NA	NA	NA	NA	-0,49283909	7	-21078,3785	42170,7632	4334,34652	0
2059	-9,45532716	NA	0,05329757	NA	-0,00210061	NA	NA	0,6894459	NA	NA	NA	NA	NA	-0,49566768	6	-21079,4032	42170,811	4334,39426	0
1169	-9,09446706	NA	NA	NA	NA	0,38722306	NA	-3,95454473	NA	0,05999672	NA	NA	0,38996522	NA	6	-21079,4516	42170,9079	4334,4912	0
1233	-9,09446706	NA	NA	NA	NA	0,38722306	NA	-3,92430325	-0,03654601	0,05956532	NA	NA	0,39466847	NA	7	-21078,9805	42171,9672	4335,55047	0
1596	-5,95790631	0,3741151	0,04613303	NA	-0,00180249	0,39660544	-0,62074526	-0,97642003	NA	NA	NA	-0,00019403	0,33432079	NA	10	-21076,0098	42172,0317	4335,61497	0
1659	-8,35316771	NA	0,03864408	NA	-0,00234345	0,43617682	-0,64441699	-0,7738147	-0,19560429	NA	NA	-0,00045111	0,35589058	NA	10	-21076,2411	42172,4944	4336,07765	0
1084	-5,95790631	0,3741151	0,04613303	NA	-0,00180249	0,39660544	-0,62074526	-1,09054773	NA	NA	NA	NA	0,33971014	NA	9	-21077,4448	42172,8996	4336,48292	0
2650	-8,97566397	0,33138274	NA	NA	-0,0012458	0,28352203	NA	1,12236839	-0,1349839	NA	NA	-0,00063299	NA	-0,50295006	9	-21078,4538	42174,9175	4338,50081	0
3073	-15,360872	NA	NA	NA	NA	NA	NA	0,72537252	NA	NA	NA	NA	0,45346103	-0,54414773	5	-21082,687	42175,3772	4338,96052	0
666	-8,97566397	0,33138274	NA	NA	-0,0012458	0,28352203	NA	-3,52383686	NA	0,05863003	NA	-0,00043937	NA	NA	8	-21082,826	42181,66	4345,24331	0
730	-8,97566397	0,33138274	NA	NA	-0,0012458	0,28352203	NA	-3,4425274	-0,05282396	0,05811259	NA	-0,00050954	NA	NA	9	-21081,9883	42181,9866	4345,56988	0
1147	-8,35316771	NA	0,03864408	NA	-0,00234345	0,43617682	-0,64441699	-1,05468211	-0,1361791	NA	NA	NA	0,35946641	NA	9	-21082,5279	42183,0658	4346,64911	0
2586	-8,97566397	0,33138274	NA	NA	-0,0012458	0,28352203	NA	0,98389174	NA	NA	NA	-0,00044751	NA	-0,50601759	8	-21084,0074	42184,0227	4347,60602	0
1644	-9,35928532	0,43489058	0,04364193	NA	-0,00182965	NA	-0,59565606	-0,7738147	-0,19560429	NA	NA	-0,00045111	0,35589058	NA	10	-21083,4388	42186,8897	4350,47295	0
2649	-7,52922856	NA	NA	NA	-0,00175017	0,32067571	NA	1,12236839	-0,1349839	NA	NA	-0,00063299	NA	-0,50295006	8	-21088,2793	42192,5665	4356,14981	0
2634	-7,76791863	0,37580763	NA	NA	-0,00127907	NA	NA	1,12236839	-0,1349839	NA	NA	-0,00063299	NA	-0,50295006	8	-21088,3424	42192,6927	4356,276	0
1658	-6,98837994	0,26427623	NA	NA	-0,00180413	0,37036432	-0,69230587	-0,7738147	-0,19560429	NA	NA	-0,00045111	0,35589058	NA	10	-21086,6593	42193,3309	4356,91413	0
154	-8,97566397	0,33138274	NA	NA	-0,0012458	0,28352203	NA	-3,73836005	NA	0,05777952	NA	NA	NA	NA	7	-21089,7799	42193,5661	4357,14934	0
1595	-8,35316771	NA	0,03864408	NA	-0,00234345	0,43617682	-0,64441699	-0,97642003	NA	NA	NA	-0,00019403	0,33432079	NA	9	-21087,9154	42193,8408	4357,42407	0
1083	-8,35316771	NA	0,03864408	NA	-0,00234345	0,43617682	-0,64441699	-1,09054773	NA	NA	NA	NA	0,33971014	NA	8	-21089,3505	42194,709	4358,29224	0
218	-8,97566397	0,33138274	NA	NA	-0,0012458	0,28352203	NA	-3,74999095	0,01316736	0,05794033	NA	NA	NA	NA	8	-21089,7174	42195,4428	4359,02611	0
2138	-8,97566397	0,33138274	NA	NA	-0,0012458	0,28352203	NA	0,6960215	-0,05271384	NA	NA	NA	NA	-0,49283909	8	-21090,2327	42196,4734	4360,05665	0
2074	-8,97566397	0,33138274	NA	NA	-0,0012458	0,28352203	NA	0,6894459	NA	NA	NA	NA	NA	-0,49566768	7	-21091,2573	42196,5209	4360,10417	0
1729	-15,360872	NA	NA	NA	NA	NA	NA	-3,6371906	-0,09814646	0,05972263	NA	-0,00047389	0,39127251	NA	7	-21091,4144	42196,835	4360,41825	0

1132	-9,35928532	0,43489058	0,04364193	NA	-0,00182965	NA	-0,59565606	-1,05468211	-0,1361791	NA	NA	NA	0,35946641	NA	9	-21089,7256	42197,4611	4361,04441	0
2642	-8,08714822	0,50972296	NA	NA	NA	0,2963004	NA	1,12236839	-0,1349839	NA	NA	-0,00063299	NA	-0,50295006	8	-21091,1502	42198,3083	4361,89157	0
2643	-11,2963041	NA	0,04958538	NA	NA	0,45107925	NA	1,12236839	-0,1349839	NA	NA	-0,00063299	NA	-0,50295006	8	-21091,5259	42199,0597	4362,64297	0
665	-7,52922856	NA	NA	NA	-0,00175017	0,32067571	NA	-3,52383686	NA	0,05863003	NA	-0,00043937	NA	NA	7	-21092,6515	42199,3093	4362,89253	0
1652	-10,737769	0,62231984	0,04626548	NA	NA	0,40997724	-0,57836783	-0,7738147	-0,19560429	NA	NA	-0,00045111	0,35589058	NA	10	-21089,6667	42199,3455	4362,92882	0
650	-7,76791863	0,37580763	NA	NA	-0,00127907	NA	NA	-3,52383686	NA	0,05863003	NA	-0,00043937	NA	NA	7	-21092,7146	42199,4354	4363,01873	0
729	-7,52922856	NA	NA	NA	-0,00175017	0,32067571	NA	-3,4425274	-0,05282396	0,05811259	NA	-0,00050954	NA	NA	8	-21091,8138	42199,6356	4363,21888	0
714	-7,76791863	0,37580763	NA	NA	-0,00127907	NA	NA	-3,4425274	-0,05282396	0,05811259	NA	-0,00050954	NA	NA	8	-21091,8769	42199,7618	4363,34507	0
1665	-15,360872	NA	NA	NA	NA	NA	NA	-3,78227707	NA	0,0606519	NA	-0,00034645	0,38158141	NA	6	-21094,2506	42200,5059	4364,08918	0
2585	-7,52922856	NA	NA	NA	-0,00175017	0,32067571	NA	0,98389174	NA	NA	NA	-0,00044751	NA	-0,50601759	7	-21093,8329	42201,672	4365,25524	0
2570	-7,76791863	0,37580763	NA	NA	-0,00127907	NA	NA	0,98389174	NA	NA	NA	-0,00044751	NA	-0,50601759	7	-21093,896	42201,7982	4365,38144	0
1146	-6,98837994	0,26427623	NA	NA	-0,00180413	0,37036432	-0,69230587	-1,05468211	-0,1361791	NA	NA	NA	0,35946641	NA	9	-21092,9462	42203,9023	4367,48559	0
1657	-5,46460783	NA	NA	NA	-0,00220571	0,40342229	-0,70298126	-0,7738147	-0,19560429	NA	NA	-0,00045111	0,35589058	NA	9	-21092,9654	42203,9408	4367,52407	0
658	-8,08714822	0,50972296	NA	NA	NA	0,2963004	NA	-3,52383686	NA	0,05863003	NA	-0,00043937	NA	NA	7	-21095,5224	42205,051	4368,6343	0
722	-8,08714822	0,50972296	NA	NA	NA	0,2963004	NA	-3,4425274	-0,05282396	0,05811259	NA	-0,00050954	NA	NA	8	-21094,6847	42205,3774	4368,96064	0
659	-11,2963041	NA	0,04958538	NA	NA	0,45107925	NA	-3,52383686	NA	0,05863003	NA	-0,00043937	NA	NA	7	-21095,8981	42205,8024	4369,3857	0
723	-11,2963041	NA	0,04958538	NA	NA	0,45107925	NA	-3,4425274	-0,05282396	0,05811259	NA	-0,00050954	NA	NA	8	-21095,0604	42206,1288	4369,71205	0
1153	-15,360872	NA	NA	NA	NA	NA	NA	-3,95454473	NA	0,05999672	NA	NA	0,38996522	NA	5	-21098,5938	42207,1909	4370,77415	0
2578	-8,08714822	0,50972296	NA	NA	NA	0,2963004	NA	0,98389174	NA	NA	NA	-0,00044751	NA	-0,50601759	7	-21096,7038	42207,4137	4370,99701	0
2579	-11,2963041	NA	0,04958538	NA	NA	0,45107925	NA	0,98389174	NA	NA	NA	-0,00044751	NA	-0,50601759	7	-21097,0795	42208,1651	4371,74841	0
1580	-9,35928532	0,43489058	0,04364193	NA	-0,00182965	NA	-0,59565606	-0,97642003	NA	NA	NA	-0,00019403	0,33432079	NA	9	-21095,1131	42208,2361	4371,81937	0
1217	-15,360872	NA	NA	NA	NA	NA	NA	-3,92430325	-0,03654601	0,05956532	NA	NA	0,39466847	NA	6	-21098,1226	42208,2499	4371,8332	0
1068	-9,35928532	0,43489058	0,04364193	NA	-0,00182965	NA	-0,59565606	-1,09054773	NA	NA	NA	NA	0,33971014	NA	8	-21096,5482	42209,1043	4372,68754	0
1140	-10,737769	0,62231984	0,04626548	NA	NA	0,40997724	-0,57836783	-1,05468211	-0,1361791	NA	NA	NA	0,35946641	NA	9	-21095,9535	42209,917	4373,50028	0
153	-7,52922856	NA	NA	NA	-0,00175017	0,32067571	NA	-3,73836005	NA	0,05777952	NA	NA	NA	NA	6	-21099,6054	42211,2155	4374,79878	0
138	-7,76791863	0,37580763	NA	NA	-0,00127907	NA	NA	-3,73836005	NA	0,05777952	NA	NA	NA	NA	6	-21099,6685	42211,3417	4374,92497	0
217	-7,52922856	NA	NA	NA	-0,00175017	0,32067571	NA	-3,74999095	0,01316736	0,05794033	NA	NA	NA	NA	7	-21099,5429	42213,0921	4376,67533	0
202	-7,76791863	0,37580763	NA	NA	-0,00127907	NA	NA	-3,74999095	0,01316736	0,05794033	NA	NA	NA	NA	7	-21099,606	42213,2182	4376,80152	0
2137	-7,52922856	NA	NA	NA	-0,00175017	0,32067571	NA	0,6960215	-0,05271384	NA	NA	NA	NA	-0,49283909	7	-21100,0582	42214,1226	4377,70587	0
2073	-7,52922856	NA	NA	NA	-0,00175017	0,32067571	NA	0,6894459	NA	NA	NA	NA	NA	-0,49566768	6	-21101,0828	42214,1703	4377,75361	0
2122	-7,76791863	0,37580763	NA	NA	-0,00127907	NA	NA	0,6960215	-0,05271384	NA	NA	NA	NA	-0,49283909	7	-21100,1213	42214,2488	4377,83206	0
2058	-7,76791863	0,37580763	NA	NA	-0,00127907	NA	NA	0,6894459	NA	NA	NA	NA	NA	-0,49566768	6	-21101,1459	42214,2965	4377,8798	0
1145	-5,46460783	NA	NA	NA	-0,00220571	0,40342229	-0,70298126	-1,05468211	-0,1361791	NA	NA	NA	0,35946641	NA	8	-21099,2523	42214,5125	4378,09576	0
1594	-6,98837994	0,26427623	NA	NA	-0,00180413	0,37036432	-0,69230587	-0,97642003	NA	NA	NA	-0,00019403	0,33432079	NA	9	-21098,3337	42214,6773	4378,26055	0
1082	-6,98837994	0,26427623	NA	NA	-0,00180413	0,37036432	-0,69230587	-1,09054773	NA	NA	NA	NA	0,33971014	NA	8	-21099,7687	42215,5454	4379,12872	0
2633	-6,18359995	NA	NA	NA	-0,00186372	NA	NA	1,12236839	-0,1349839	NA	NA	-0,00063299	NA	-0,50295006	7	-21101,1933	42216,3928	4379,97608	0
146	-8,08714822	0,50972296	NA	NA	NA	0,2963004	NA	-3,73836005	NA	0,05777952	NA	NA	NA	NA	6	-21102,4763	42216,9573	4380,54054	0
1643	-5,75020926	NA	0,0346068	NA	-0,00247017	NA	-0,61951905	-0,7738147	-0,19560429	NA	NA	-0,00045111	0,35589058	NA	9	-21099,792	42217,594	4381,17732	0
147	-11,2963041	NA	0,04958538	NA	NA	0,45107925	NA	-3,73836005	NA	0,05777952	NA	NA	NA	NA	6	-21102,852	42217,7087	4381,29195	0
2626	-10,126072	0,56333515	NA	NA	NA	NA	NA	1,12236839	-0,1349839	NA	NA	-0,00063299	NA	-0,50295006	7	-21101,9458	42217,8977	4381,48098	0
210	-8,08714822	0,50972296	NA	NA	NA	0,2963004	NA	-3,74999095	0,01316736	0,05794033	NA	NA	NA	NA	7	-21102,4138	42218,8338	4382,41709	0
211	-11,2963041	NA	0,04958538	NA	NA	0,45107925	NA	-3,74999095	0,01316736	0,05794033	NA	NA	NA	NA	7	-21102,7895	42219,5852	4383,1685	0
2130	-8,08714822	0,50972296	NA	NA	NA	0,2963004	NA	0,6960215	-0,05271384	NA	NA	NA	NA	-0,49283909	7	-21102,9291	42219,8644	4383,44763	0
2066	-8,08714822	0,50972296	NA	NA	NA	0,2963004	NA	0,6894459	NA	NA	NA	NA	NA	-0,49566768	6	-21103,9537	42219,9121	4383,49537	0
2131	-11,2963041	NA	0,04958538	NA	NA	0,45107925	NA	0,6960215	-0,05271384	NA	NA	NA	NA	-0,49283909	7	-21103,3048	42220,6158	4384,19904	0
2067	-11,2963041	NA	0,04958538	NA	NA	0,45107925	NA	0,6894459	NA	NA	NA	NA	NA	-0,49566768	6	-21104,3294	42220,6635	4384,24678	0
1588	-10,737769	0,62231984	0,04626548	NA	NA	0,40997724	-0,57836783	-0,97642003	NA	NA	NA	-0,00019403	0,33432079	NA	9	-21101,341	42220,692	4384,27525	0
1076	-10,737769	0,62231984	0,04626548	NA	NA	0,40997724	-0,57836783	-1,09054773	NA	NA	NA	NA	0,33971014	NA	8	-21102,7761	42221,5601	4385,14342	0
649	-6,18359995	NA	NA	NA	-0,00186372	NA	NA	-3,52383686	NA	0,05863003	NA	-0,00043937	NA	NA	6	-21105,5656	42223,1358	4386,71903	0
713	-6,18359995	NA	NA	NA	-0,00186372	NA	NA	-3,4425274	-0,05282396	0,05811259	NA	-0,00050954	NA	NA	7	-21104,7278	42223,4619	4387,04515	0
642	-10,126072	0,56333515	NA	NA	NA	NA	NA	-3,52383686	NA	0,05863003	NA	-0,00043937	NA	NA	6	-21106,318	42224,6406	4388,22393	0
1642	-6,22256645	0,32760888	NA	NA	-0,00183217	NA	-0,66370241	-0,7738147	-0,19560429	NA	NA	-0,00045111	0,35589058	NA	9	-21103,3406	42224,6911	4388,27438	0
706	-10,126072	0,56333515	NA	NA	NA	NA	NA	-3,4425274	-0,05282396	0,05811259	NA	-0,00050954	NA	NA	7	-21105,4803	42224,9668	4388,55005	0
1593	-5,46460783	NA	NA	NA	-0,00220571	0,40342229	-0,70298126	-0,97642003	NA	NA	NA	-0,00019403	0,33432079	NA	8	-21104,6397	42225,2874	4388,87072	0
2569	-6,18359995	NA	NA	NA	-0,00186372	NA	NA	0,98389174	NA	NA	NA	-0,00044751	NA	-0,50601759	6	-21106,7469	42225,4985	4389,08174	0
1081	-5,46460783	NA	NA	NA	-0,00220571	0,40342229	-0,70298126	-1,09054773	NA	NA	NA	NA	0,33971014	NA	7	-21106,0748	42226,1558	4389,73911	0
2562	-10,126072	0,56333515	NA	NA	NA	NA	NA	0,98389174	NA	NA	NA	-0,00044751	NA	-0,50601759	6	-21107,4994	42227,0034	4390,58664	0
1131	-5,75020926	NA	0,0346068	NA	-0,00247017	NA	-0,61951905	-1,05468211	-0,1361791	NA	NA	NA	0,35946641	NA	8	-21106,0789	42228,1657	4391,749	0
137	-6,18359995	NA	NA	NA	-0,00186372	NA	NA	-3,73836005	NA	0,05777952	NA	NA	NA	NA	5	-21112,5195	42235,0422	4398,6255	0
1130	-6,22256645	0,32760888	NA	NA	-0,00183217	NA	-0,66370241	-1,05468211	-0,1361791	NA	NA	NA	0,35946641	NA	8	-21109,6274	42235,2628	4398,84606	0
130	-10,126072	0,56333515	NA	NA	NA	NA	NA	-3,73836005	NA	0,05777952	NA	NA	NA	NA	5	-21113,2719	42236,5471	4400,1304	0

201	-6,18359995	NA	NA	NA	-0,00186372	NA	NA	-3,74999095	0,01316736	0,05794033	NA	NA	NA	NA	6	-21112,457	42236,9185	4400,50183	0
2121	-6,18359995	NA	NA	NA	-0,00186372	NA	NA	0,6960215	-0,05271384	NA	NA	NA	NA	-0,49283909	6	-21112,9722	42237,9491	4401,53237	0
2057	-6,18359995	NA	NA	NA	-0,00186372	NA	NA	0,6894459	NA	NA	NA	NA	NA	-0,49566768	5	-21113,9969	42237,997	4401,58033	0
1636	-9,79595589	0,69445228	0,04389505	NA	NA	NA	-0,55018722	-0,7738147	-0,19560429	NA	NA	-0,00045111	0,35589058	NA	9	-21110,0792	42238,1684	4401,75167	0
194	-10,126072	0,56333515	NA	NA	NA	NA	NA	-3,74999095	0,01316736	0,05794033	NA	NA	NA	NA	6	-21113,2094	42238,4234	4402,00672	0
1579	-5,75020926	NA	0,0346068	NA	-0,00247017	NA	-0,61951905	-0,97642003	NA	NA	NA	-0,00019403	0,33432079	NA	8	-21111,4664	42238,9407	4402,52396	0
636	-5,95790631	0,3741151	0,04613303	NA	-0,00180249	0,39660544	-0,62074526	-0,66565225	-0,15004816	NA	NA	-0,00048657	NA	NA	10	-21109,6308	42239,2738	4402,85712	0
2114	-10,126072	0,56333515	NA	NA	NA	NA	NA	0,6960215	-0,05271384	NA	NA	NA	NA	-0,49283909	6	-21113,7247	42239,454	4403,03726	0
2050	-10,126072	0,56333515	NA	NA	NA	NA	NA	0,6894459	NA	NA	NA	NA	NA	-0,49566768	5	-21114,7493	42239,5019	4403,08523	0
1067	-5,75020926	NA	0,0346068	NA	-0,00247017	NA	-0,61951905	-1,09054773	NA	NA	NA	NA	0,33971014	NA	7	-21112,9014	42239,8091	4403,39235	0
1650	-8,51824578	0,51124721	NA	NA	NA	0,38535796	-0,65213053	-0,7738147	-0,19560429	NA	NA	-0,00045111	0,35589058	NA	9	-21112,0662	42242,1423	4405,72557	0
1641	-3,98530292	NA	NA	NA	-0,00234183	NA	-0,67333606	-0,7738147	-0,19560429	NA	NA	-0,00045111	0,35589058	NA	8	-21113,2117	42242,4313	4406,01462	0
1578	-6,22256645	0,32760888	NA	NA	-0,00183217	NA	-0,66370241	-0,97642003	NA	NA	NA	-0,00019403	0,33432079	NA	8	-21115,0149	42246,0377	4409,62102	0
1066	-6,22256645	0,32760888	NA	NA	-0,00183217	NA	-0,66370241	-1,09054773	NA	NA	NA	NA	0,33971014	NA	7	-21116,45	42246,9061	4410,48942	0
2627	-11,7728913	NA	0,04392487	NA	NA	NA	NA	1,12236839	-0,1349839	NA	NA	-0,00063299	NA	-0,50295006	7	-21117,1483	42248,3027	4411,88598	0
1124	-9,79595589	0,69445228	0,04389505	NA	NA	NA	-0,55018722	-1,05468211	-0,1361791	NA	NA	NA	0,35946641	NA	8	-21116,3661	42248,7401	4412,32335	0
572	-5,95790631	0,3741151	0,04613303	NA	-0,00180249	0,39660544	-0,62074526	-0,82885018	NA	NA	NA	-0,0002841	NA	NA	9	-21116,6418	42251,2935	4414,87678	0
124	-5,95790631	0,3741151	0,04613303	NA	-0,00180249	0,39660544	-0,62074526	-0,96744453	-0,08610767	NA	NA	NA	NA	NA	9	-21116,9118	42251,8335	4415,41679	0
1138	-8,51824578	0,51124721	NA	NA	NA	0,38535796	-0,65213053	-1,05468211	-0,1361791	NA	NA	NA	0,35946641	NA	8	-21118,353	42252,714	4416,29725	0
1129	-3,98530292	NA	NA	NA	-0,00234183	NA	-0,67333606	-1,05468211	-0,1361791	NA	NA	NA	0,35946641	NA	7	-21119,4985	42253,0032	4416,58652	0
643	-11,7728913	NA	0,04392487	NA	NA	NA	NA	-3,52383686	NA	0,05863003	NA	-0,00043937	NA	NA	6	-21121,5205	42255,0457	4418,62893	0
707	-11,7728913	NA	0,04392487	NA	NA	NA	NA	-3,4425274	-0,05282396	0,05811259	NA	-0,00050954	NA	NA	7	-21120,6828	42255,3718	4418,95505	0
60	-5,95790631	0,3741151	0,04613303	NA	-0,00180249	0,39660544	-0,62074526	-0,99369134	NA	NA	NA	NA	NA	NA	8	-21119,7052	42255,4184	4419,00171	0
2563	-11,7728913	NA	0,04392487	NA	NA	NA	NA	0,98389174	NA	NA	NA	-0,00044751	NA	-0,50601759	6	-21122,7019	42257,4084	4420,99164	0
2641	-9,09446706	NA	NA	NA	NA	0,38722306	NA	1,12236839	-0,1349839	NA	NA	-0,00063299	NA	-0,50295006	7	-21122,2817	42258,5695	4422,15277	0
1572	-9,79595589	0,69445228	0,04389505	NA	NA	NA	-0,55018722	-0,97642003	NA	NA	NA	-0,00019403	0,33432079	NA	8	-21121,7535	42259,515	4423,09831	0
1060	-9,79595589	0,69445228	0,04389505	NA	NA	NA	-0,55018722	-1,09054773	NA	NA	NA	NA	0,33971014	NA	7	-21123,1886	42260,3834	4423,9667	0
635	-8,35316771	NA	0,03864408	NA	-0,00234345	0,43617682	-0,64441699	-0,66565225	-0,15004816	NA	NA	-0,00048657	NA	NA	9	-21121,5365	42261,0829	4424,66622	0
1586	-8,51824578	0,51124721	NA	NA	NA	0,38535796	-0,65213053	-0,97642003	NA	NA	NA	-0,00019403	0,33432079	NA	8	-21123,7405	42263,4889	4427,07222	0
1577	-3,98530292	NA	NA	NA	-0,00234183	NA	-0,67333606	-0,97642003	NA	NA	NA	-0,00019403	0,33432079	NA	7	-21124,886	42263,7782	4427,36149	0
1074	-8,51824578	0,51124721	NA	NA	NA	0,38535796	-0,65213053	-1,09054773	NA	NA	NA	NA	0,33971014	NA	7	-21125,1756	42264,3573	4427,94061	0
1065	-3,98530292	NA	NA	NA	-0,00234183	NA	-0,67333606	-1,09054773	NA	NA	NA	NA	0,33971014	NA	6	-21126,3211	42264,6468	4428,2301	0
657	-9,09446706	NA	NA	NA	NA	0,38722306	NA	-3,52383686	NA	0,05863003	NA	-0,00043937	NA	NA	6	-21126,6539	42265,3124	4428,89572	0
721	-9,09446706	NA	NA	NA	NA	0,38722306	NA	-3,4425274	-0,05282396	0,05811259	NA	-0,00050954	NA	NA	7	-21125,8162	42265,6386	4429,22184	0
131	-11,7728913	NA	0,04392487	NA	NA	NA	NA	-3,73836005	NA	0,05777952	NA	NA	NA	NA	5	-21128,4744	42266,9521	4430,5354	0
2577	-9,09446706	NA	NA	NA	NA	0,38722306	NA	0,98389174	NA	NA	NA	-0,00044751	NA	-0,50601759	6	-21127,8353	42267,6752	4431,25843	0
195	-11,7728913	NA	0,04392487	NA	NA	NA	NA	-3,74999095	0,01316736	0,05794033	NA	NA	NA	NA	6	-21128,4119	42268,8284	4432,41173	0
2115	-11,7728913	NA	0,04392487	NA	NA	NA	NA	0,6960215	-0,05271384	NA	NA	NA	NA	-0,49283909	6	-21128,9272	42269,859	4433,44227	0
2051	-11,7728913	NA	0,04392487	NA	NA	NA	NA	0,6894459	NA	NA	NA	NA	NA	-0,49566768	5	-21129,9518	42269,9069	4433,49023	0
571	-8,35316771	NA	0,03864408	NA	-0,00234345	0,43617682	-0,64441699	-0,82885018	NA	NA	NA	-0,0002841	NA	NA	8	-21128,5474	42273,1028	4436,6861	0
123	-8,35316771	NA	0,03864408	NA	-0,00234345	0,43617682	-0,64441699	-0,96744453	-0,08610767	NA	NA	NA	NA	NA	8	-21128,8174	42273,6428	4437,22611	0
620	-9,35928532	0,43489058	0,04364193	NA	-0,00182965	NA	-0,59565606	-0,66565225	-0,15004816	NA	NA	-0,00048657	NA	NA	9	-21128,7341	42275,4782	4439,06152	0
1634	-7,20552725	0,58574016	0,04364193	NA	NA	NA	-0,62020516	-0,7738147	-0,19560429	NA	NA	-0,00045111	0,35589058	NA	8	-21130,1002	42276,2084	4439,79168	0
145	-9,09446706	NA	NA	NA	NA	0,38722306	NA	-3,73836005	NA	0,05777952	NA	NA	NA	NA	5	-21133,6078	42277,2189	4440,80219	0
59	-8,35316771	NA	0,03864408	NA	-0,00234345	0,43617682	-0,64441699	-0,99369134	NA	NA	NA	NA	NA	NA	7	-21131,6109	42277,228	4440,81125	0
209	-9,09446706	NA	NA	NA	NA	0,38722306	NA	-3,74999095	0,01316736	0,05794033	NA	NA	NA	NA	6	-21133,5453	42279,0952	4442,67852	0
2129	-9,09446706	NA	NA	NA	NA	0,38722306	NA	0,6960215	-0,05271384	NA	NA	NA	NA	-0,49283909	6	-21134,0606	42280,1258	4443,70906	0
2065	-9,09446706	NA	NA	NA	NA	0,38722306	NA	0,6894459	NA	NA	NA	NA	NA	-0,49566768	5	-21135,0852	42280,1737	4443,75702	0
634	-6,98837994	0,26427623	NA	NA	-0,00180413	0,37036432	-0,69230587	-0,66565225	-0,15004816	NA	NA	-0,00048657	NA	NA	9	-21131,9547	42281,9194	4445,5027	0
1651	-8,8387814	NA	0,03010563	NA	NA	0,51295904	-0,60572616	-0,7738147	-0,19560429	NA	NA	-0,00045111	0,35589058	NA	9	-21133,1169	42284,2437	4447,82698	0
1122	-7,20552725	0,58574016	NA	NA	NA	NA	-0,62020516	-1,05468211	-0,1361791	NA	NA	NA	0,35946641	NA	7	-21136,3871	42286,7803	4450,36358	0
556	-9,35928532	0,43489058	0,04364193	NA	-0,00182965	NA	-0,59565606	-0,82885018	NA	NA	NA	-0,0002841	NA	NA	8	-21135,7451	42287,4981	4451,08141	0
628	-10,737769	0,62231984	0,04626548	NA	NA	0,40997724	-0,57836783	-0,66565225	-0,15004816	NA	NA	-0,00048657	NA	NA	9	-21134,9621	42287,9341	4451,51739	0
108	-9,35928532	0,43489058	0,04364193	NA	-0,00182965	NA	-0,59565606	-0,96744453	-0,08610767	NA	NA	NA	NA	NA	8	-21136,0151	42288,0381	4451,62141	0
44	-9,35928532	0,43489058	0,04364193	NA	-0,00182965	NA	-0,59565606	-0,99369134	NA	NA	NA	NA	NA	NA	7	-21138,8085	42291,6233	4455,20655	0
633	-5,46460783	NA	NA	NA	-0,00220571	0,40342229	-0,70298126	-0,66565225	-0,15004816	NA	NA	-0,00048657	NA	NA	8	-21138,2608	42292,5296	4456,11287	0
570	-6,98837994	0,26427623	NA	NA	-0,00180413	0,37036432	-0,69230587	-0,82885018	NA	NA	NA	-0,0002841	NA	NA	8	-21138,9657	42293,9393	4457,52259	0
122	-6,98837994	0,26427623	NA	NA	-0,00180413	0,37036432	-0,69230587	-0,96744453	-0,08610767	NA	NA	NA	NA	NA	8	-21139,2357	42294,4793	4458,0626	0
1139	-8,8387814	NA	0,03010563	NA	NA	0,51295904	-0,60572616	-1,05468211	-0,1361791	NA	NA	NA	0,35946641	NA	8	-21139,4037	42294,8154	4458,39866	0
2625	-15,360872	NA	NA	NA	NA	NA	NA	1,12236839	-0,1349839	NA	NA	-0,00063299	NA	-0,50295006	6	-21141,4238	42294,8522	4458,4355	0
1570	-7,20552725	0,58574016	NA	NA	NA	NA	-0,62020516	-0,97642003	NA	NA	NA	-0,00019403	0,33432079	NA	7	-21141,7745	42297,5553	4461,13855	0

58	-6,98837994	0,26427623	NA	NA	-0,00180413	0,37036432	-0,69230587	-0,99369134	NA	NA	NA	NA	NA	NA	7	-21142,0291	42298,0645	4461,64773	0
1058	-7,20552725	0,58574016	NA	NA	NA	NA	-0,62020516	-1,09054773	NA	NA	NA	NA	0,33971014	NA	6	-21143,2096	42298,4239	4462,00716	0
564	-10,737769	0,62231984	0,04626548	NA	NA	0,40997724	-0,57836783	-0,82885018	NA	NA	NA	-0,0002841	NA	NA	8	-21141,973	42299,954	4463,53728	0
116	-10,737769	0,62231984	0,04626548	NA	NA	0,40997724	-0,57836783	-0,96744453	-0,08610767	NA	NA	NA	NA	NA	8	-21142,243	42300,494	4464,07729	0
641	-15,360872	NA	NA	NA	NA	NA	NA	-3,52383686	NA	0,05863003	NA	-0,00043937	NA	NA	5	-21145,796	42301,5954	4465,17867	0
705	-15,360872	NA	NA	NA	NA	NA	NA	-3,4425274	-0,05282396	0,05811259	NA	-0,00050954	NA	NA	6	-21144,9583	42301,9213	4465,50457	0
1649	-6,3315051	NA	NA	NA	NA	0,4838605	-0,6580268	-0,7738147	-0,19560429	NA	NA	-0,00045111	0,35589058	NA	8	-21143,5314	42303,0708	4466,65407	0
2561	-15,360872	NA	NA	NA	NA	NA	NA	0,98389174	NA	NA	NA	-0,00044751	NA	-0,50601759	5	-21146,9774	42303,9581	4467,54138	0
52	-10,737769	0,62231984	0,04626548	NA	NA	0,40997724	-0,57836783	-0,99369134	NA	NA	NA	NA	NA	NA	7	-21145,0365	42304,0791	4467,66243	0
569	-5,46460783	NA	NA	NA	-0,00220571	0,40342229	-0,70298126	-0,82885018	NA	NA	NA	-0,0002841	NA	NA	7	-21145,2718	42304,5497	4468,13297	0
121	-5,46460783	NA	NA	NA	-0,00220571	0,40342229	-0,70298126	-0,96744453	-0,08610767	NA	NA	NA	NA	NA	7	-21145,5418	42305,0897	4468,67298	0
1587	-8,8387814	NA	0,03010563	NA	NA	0,51295904	-0,60572616	-0,97642003	NA	NA	NA	-0,00019403	0,33432079	NA	8	-21144,7912	42305,5904	4469,17363	0
619	-5,75020926	NA	0,0346068	NA	-0,00247017	NA	-0,61951905	-0,66565225	-0,15004816	NA	NA	-0,00048657	NA	NA	8	-21145,0874	42306,1828	4469,76611	0
1075	-8,8387814	NA	0,03010563	NA	NA	0,51295904	-0,60572616	-1,09054773	NA	NA	NA	NA	0,33971014	NA	7	-21146,2263	42306,4587	4470,04202	0
57	-5,46460783	NA	NA	NA	-0,00220571	0,40342229	-0,70298126	-0,99369134	NA	NA	NA	NA	NA	NA	6	-21148,3352	42308,6751	4472,25834	0
618	-6,22256645	0,32760888	NA	NA	-0,00183217	NA	-0,66370241	-0,66565225	-0,15004816	NA	NA	-0,00048657	NA	NA	8	-21148,636	42313,2799	4476,86317	0
129	-15,360872	NA	NA	NA	NA	NA	NA	-3,73836005	NA	0,05777952	NA	NA	NA	NA	4	-21152,7499	42313,5021	4477,08536	0
1137	-6,3315051	NA	NA	NA	NA	0,4838605	-0,6580268	-1,05468211	-0,1361791	NA	NA	NA	0,35946641	NA	7	-21149,8182	42313,6427	4477,22597	0
193	-15,360872	NA	NA	NA	NA	NA	NA	-3,74999095	0,01316736	0,05794033	NA	NA	NA	NA	5	-21152,6874	42315,3782	4478,96147	0
2113	-15,360872	NA	NA	NA	NA	NA	NA	0,6960215	-0,05271384	NA	NA	NA	NA	-0,49283909	5	-21153,2027	42316,4087	4479,99201	0
2049	-15,360872	NA	NA	NA	NA	NA	NA	0,6894459	NA	NA	NA	NA	NA	-0,49566768	4	-21154,2274	42316,4569	4480,04019	0
1628	-9,61919161	0,46601352	0,06534536	NA	-0,00131078	0,33938341	NA	-0,7738147	-0,19560429	NA	NA	-0,00045111	0,35589058	NA	10	-21148,4891	42316,9904	4480,57372	0
555	-5,75020926	NA	0,0346068	NA	-0,00247017	NA	-0,61951905	-0,82885018	NA	NA	NA	-0,0002841	NA	NA	7	-21152,0984	42318,2029	4481,78622	0
107	-5,75020926	NA	0,0346068	NA	-0,00247017	NA	-0,61951905	-0,96744453	-0,08610767	NA	NA	NA	NA	NA	7	-21152,3684	42318,7429	4482,32623	0
43	-5,75020926	NA	0,0346068	NA	-0,00247017	NA	-0,61951905	-0,99369134	NA	NA	NA	NA	NA	NA	6	-21155,1618	42322,3283	4485,91159	0
1585	-6,3315051	NA	NA	NA	NA	0,4838605	-0,6580268	-0,97642003	NA	NA	NA	-0,00019403	0,33432079	NA	7	-21155,2057	42324,4177	4488,00093	0
1073	-6,3315051	NA	NA	NA	NA	0,4838605	-0,6580268	-1,09054773	NA	NA	NA	NA	0,33971014	NA	6	-21156,6408	42325,2863	4488,86955	0
554	-6,22256645	0,32760888	NA	NA	-0,00183217	NA	-0,66370241	-0,82885018	NA	NA	NA	-0,0002841	NA	NA	7	-21155,6469	42325,3	4488,88328	0
106	-6,22256645	0,32760888	NA	NA	-0,00183217	NA	-0,66370241	-0,96744453	-0,08610767	NA	NA	NA	NA	NA	7	-21155,9169	42325,84	4489,42329	0
612	-9,79595589	0,69445228	0,04389505	NA	NA	NA	-0,55018722	-0,66565225	-0,15004816	NA	NA	-0,00048657	NA	NA	8	-21155,3746	42326,7572	4490,34046	0
1116	-9,61919161	0,46601352	0,06534536	NA	-0,00131078	0,33938341	NA	-1,05468211	-0,1361791	NA	NA	NA	0,35946641	NA	9	-21154,776	42327,5619	4491,14518	0
42	-6,22256645	0,32760888	NA	NA	-0,00183217	NA	-0,66370241	-0,99369134	NA	NA	NA	NA	NA	NA	6	-21158,7104	42329,4254	4493,00865	0
626	-8,51824578	0,51124721	NA	NA	NA	0,38535796	-0,65213053	-0,66565225	-0,15004816	NA	NA	-0,00048657	NA	NA	8	-21157,3616	42330,7311	4494,31437	0
617	-3,98530292	NA	NA	NA	-0,00234183	NA	-0,67333606	-0,66565225	-0,15004816	NA	NA	-0,00048657	NA	NA	7	-21158,5071	42331,0204	4494,60363	0
1564	-9,61919161	0,46601352	0,06534536	NA	-0,00131078	0,33938341	NA	-0,97642003	NA	NA	NA	-0,00019403	0,33432079	NA	9	-21160,1635	42338,3369	4501,92014	0
548	-9,79595589	0,69445228	0,04389505	NA	NA	NA	-0,55018722	-0,82885018	NA	NA	NA	-0,0002841	NA	NA	7	-21162,3855	42338,7773	4502,36057	0
1052	-9,61919161	0,46601352	0,06534536	NA	-0,00131078	0,33938341	NA	-1,09054773	NA	NA	NA	NA	0,33971014	NA	8	-21161,5985	42339,205	4502,78831	0
100	-9,79595589	0,69445228	0,04389505	NA	NA	NA	-0,55018722	-0,96744453	-0,08610767	NA	NA	NA	NA	NA	7	-21162,6556	42339,3173	4502,90058	0
562	-8,51824578	0,51124721	NA	NA	NA	0,38535796	-0,65213053	-0,82885018	NA	NA	NA	-0,0002841	NA	NA	7	-21164,3725	42342,7512	4506,33447	0
1620	-13,7669049	0,65178191	0,06462958	NA	NA	0,35119819	NA	-0,7738147	-0,19560429	NA	NA	-0,00045111	0,35589058	NA	9	-21162,3714	42342,7528	4506,33612	0
36	-9,79595589	0,69445228	0,04389505	NA	NA	NA	-0,55018722	-0,99369134	NA	NA	NA	NA	NA	NA	6	-21165,449	42342,9027	4506,48594	0
553	-3,98530292	NA	NA	NA	-0,00234183	NA	-0,67333606	-0,82885018	NA	NA	NA	-0,0002841	NA	NA	6	-21165,518	42343,0407	4506,62396	0
1612	-11,9654714	0,51367836	0,06255183	NA	-0,0013444	NA	NA	-0,7738147	-0,19560429	NA	NA	-0,00045111	0,35589058	NA	9	-21162,56	42343,13	4506,71327	0
114	-8,51824578	0,51124721	NA	NA	NA	0,38535796	-0,65213053	-0,96744453	-0,08610767	NA	NA	NA	NA	NA	7	-21164,6425	42343,2912	4506,87448	0
105	-3,98530292	NA	NA	NA	-0,00234183	NA	-0,67333606	-0,96744453	-0,08610767	NA	NA	NA	NA	NA	6	-21165,788	42343,5807	4507,16397	0
50	-8,51824578	0,51124721	NA	NA	NA	0,38535796	-0,65213053	-0,99369134	NA	NA	NA	NA	NA	NA	6	-21167,436	42346,8766	4510,45984	0
41	-3,98530292	NA	NA	NA	-0,00234183	NA	-0,67333606	-0,99369134	NA	NA	NA	NA	NA	NA	5	-21168,5815	42347,1663	4510,74955	0
1635	-9,52399274	NA	0,02470059	NA	NA	NA	-0,56950148	-0,7738147	-0,19560429	NA	NA	-0,00045111	0,35589058	NA	8	-21166,1566	42348,3211	4511,90434	0
1627	-11,3659105	NA	0,05744064	NA	-0,00198557	0,38445946	NA	-0,7738147	-0,19560429	NA	NA	-0,00045111	0,35589058	NA	9	-21166,9821	42351,9741	4515,55735	0
1108	-13,7669049	0,65178191	0,06462958	NA	NA	0,35119819	NA	-1,05468211	-0,1361791	NA	NA	NA	0,35946641	NA	8	-21168,6583	42353,3245	4516,9078	0
1100	-11,9654714	0,51367836	0,06255183	NA	-0,0013444	NA	NA	-1,05468211	-0,1361791	NA	NA	NA	0,35946641	NA	8	-21168,8469	42353,7017	4517,28495	0
1123	-9,52399274	NA	0,02470059	NA	NA	NA	-0,56950148	-1,05468211	-0,1361791	NA	NA	NA	0,35946641	NA	7	-21172,4434	42358,893	4522,47624	0
1633	-8,58193391	NA	NA	NA	NA	NA	-0,61393687	-0,7738147	-0,19560429	NA	NA	-0,00045111	0,35589058	NA	7	-21173,1726	42360,3515	4523,93473	0
1115	-11,3659105	NA	0,05744064	NA	-0,00198557	0,38445946	NA	-1,05468211	-0,1361791	NA	NA	NA	0,35946641	NA	8	-21173,2689	42362,5457	4526,12903	0
1556	-13,7669049	0,65178191	0,06462958	NA	NA	0,35119819	NA	-0,97642003	NA	NA	NA	-0,00019403	0,33432079	NA	8	-21174,0458	42364,0995	4527,68277	0
1548	-11,9654714	0,51367836	0,06255183	NA	-0,0013444	NA	NA	-0,97642003	NA	NA	NA	-0,00019403	0,33432079	NA	8	-21174,2343	42364,4766	4528,05991	0
610	-7,20552725	0,58574016	NA	NA	NA	NA	-0,62020516	-0,66565225	-0,15004816	NA	NA	-0,00048657	NA	NA	7	-21175,3956	42364,7974	4528,3807	0
1044	-13,7669049	0,65178191	0,06462958	NA	NA	0,35119819	NA	-1,09054773	NA	NA	NA	NA	0,33971014	NA	7	-21175,4808	42364,9679	4528,55116	0
1036	-11,9654714	0,51367836	0,06255183	NA	-0,0013444	NA	NA	-1,09054773	NA	NA	NA	NA	0,33971014	NA	7	-21175,6694	42365,345	4528,9283	0
1571	-9,52399274	NA	0,02470059	NA	NA	NA	-0,56950148	-0,97642003	NA	NA	NA	-0,00019403	0,33432079	NA	7	-21177,8309	42369,6679	4533,25121	0
1059	-9,52399274	NA	0,02470059	NA	NA	NA	-0,56950148	-1,09054773	NA	NA	NA	NA	0,33971014	NA	6	-21179,266	42370,5365	4534,11982	0

1121	-8,58193391	NA	NA	NA	NA	NA	-0,61393687	-1,05468211	-0,1361791	NA	NA	NA	0,35946641	NA	6	-21179,4595	42370,9236	4534,50686	0
1604	-12,0135881	0,70944129	0,06178489	NA	NA	NA	NA	-0,7738147	-0,19560429	NA	NA	-0,00045111	0,35589058	NA	8	-21177,4584	42370,9248	4534,50811	0
627	-8,8387814	NA	0,03010563	NA	NA	0,51295904	-0,60572616	-0,66565225	-0,15004816	NA	NA	-0,00048657	NA	NA	8	-21178,4123	42372,8325	4536,41578	0
1563	-11,3659105	NA	0,05744064	NA	-0,00198557	0,38445946	NA	-0,97642003	NA	NA	NA	-0,00019403	0,33432079	NA	8	-21178,6564	42373,3207	4536,90399	0
1051	-11,3659105	NA	0,05744064	NA	-0,00198557	0,38445946	NA	-1,09054773	NA	NA	NA	NA	0,33971014	NA	7	-21180,0915	42374,1891	4537,77238	0
546	-7,20552725	0,58574016	NA	NA	NA	NA	-0,62020516	-0,82885018	NA	NA	NA	-0,0002841	NA	NA	6	-21182,4066	42376,8177	4540,40102	0
98	-7,20552725	0,58574016	NA	NA	NA	NA	-0,62020516	-0,96744453	-0,08610767	NA	NA	NA	NA	NA	6	-21182,6766	42377,3578	4540,94103	0
34	-7,20552725	0,58574016	NA	NA	NA	NA	-0,62020516	-0,99369134	NA	NA	NA	NA	NA	NA	5	-21185,47	42380,9433	4544,52661	0
1092	-12,0135881	0,70944129	0,06178489	NA	NA	NA	NA	-1,05468211	-0,1361791	NA	NA	NA	0,35946641	NA	7	-21183,7453	42381,4967	4545,08001	0
1569	-8,58193391	NA	NA	NA	NA	NA	-0,61393687	-0,97642003	NA	NA	NA	-0,00019403	0,33432079	NA	6	-21184,847	42381,6985	4545,28182	0
1057	-8,58193391	NA	NA	NA	NA	NA	-0,61393687	-1,09054773	NA	NA	NA	NA	0,33971014	NA	5	-21186,282	42382,5674	4546,15066	0
563	-8,8387814	NA	0,03010563	NA	NA	0,51295904	-0,60572616	-0,82885018	NA	NA	NA	-0,0002841	NA	NA	7	-21185,4232	42384,8526	4548,43588	0
115	-8,8387814	NA	0,03010563	NA	NA	0,51295904	-0,60572616	-0,96744453	-0,08610767	NA	NA	NA	NA	NA	7	-21185,6932	42385,3926	4548,97589	0
1611	-9,45532716	NA	0,05329757	NA	-0,00210061	NA	NA	-0,7738147	-0,19560429	NA	NA	-0,00045111	0,35589058	NA	8	-21185,3376	42386,6832	4550,26643	0
51	-8,8387814	NA	0,03010563	NA	NA	0,51295904	-0,60572616	-0,99369134	NA	NA	NA	NA	NA	NA	6	-21188,4867	42388,978	4552,56125	0
625	-6,3315051	NA	NA	NA	NA	0,4838605	-0,6580268	-0,66565225	-0,15004816	NA	NA	-0,00048657	NA	NA	7	-21188,8268	42391,6598	4555,24308	0
1540	-12,0135881	0,70944129	0,06178489	NA	NA	NA	NA	-0,97642003	NA	NA	NA	-0,00019403	0,33432079	NA	7	-21189,1328	42392,2717	4555,85497	0
1028	-12,0135881	0,70944129	0,06178489	NA	NA	NA	NA	-1,09054773	NA	NA	NA	NA	0,33971014	NA	6	-21190,5678	42393,1403	4556,72359	0
1099	-9,45532716	NA	0,05329757	NA	-0,00210061	NA	NA	-1,05468211	-0,1361791	NA	NA	NA	0,35946641	NA	7	-21191,6244	42397,2551	4560,83833	0
561	-6,3315051	NA	NA	NA	NA	0,4838605	-0,6580268	-0,82885018	NA	NA	NA	-0,0002841	NA	NA	6	-21195,8377	42403,6801	4567,26341	0
113	-6,3315051	NA	NA	NA	NA	0,4838605	-0,6580268	-0,96744453	-0,08610767	NA	NA	NA	NA	NA	6	-21196,1077	42404,2201	4567,80342	0
604	-9,61919161	0,46601352	0,06534536	NA	-0,00131078	0,33938341	NA	-0,66565225	-0,15004816	NA	NA	-0,00048657	NA	NA	9	-21193,7845	42405,579	4569,16229	0
49	-6,3315051	NA	NA	NA	NA	0,4838605	-0,6580268	-0,99369134	NA	NA	NA	NA	NA	NA	5	-21198,9012	42407,8057	4571,389	0
1547	-9,45532716	NA	0,05329757	NA	-0,00210061	NA	NA	-0,97642003	NA	NA	NA	-0,00019403	0,33432079	NA	7	-21197,0119	42408,03	4571,6133	0
1035	-9,45532716	NA	0,05329757	NA	-0,00210061	NA	NA	-1,09054773	NA	NA	NA	NA	0,33971014	NA	6	-21198,447	42408,8986	4572,48191	0
1626	-8,97566397	0,33138274	NA	NA	-0,0012458	0,28352203	NA	-0,7738147	-0,19560429	NA	NA	-0,00045111	0,35589058	NA	9	-21197,1918	42412,3935	4575,97679	0
540	-9,61919161	0,46601352	0,06534536	NA	-0,00131078	0,33938341	NA	-0,82885018	NA	NA	NA	-0,0002841	NA	NA	8	-21200,7955	42417,5989	4581,18218	0
92	-9,61919161	0,46601352	0,06534536	NA	-0,00131078	0,33938341	NA	-0,96744453	-0,08610767	NA	NA	NA	NA	NA	8	-21201,0655	42418,1389	4581,72218	0
28	-9,61919161	0,46601352	0,06534536	NA	-0,00131078	0,33938341	NA	-0,99369134	NA	NA	NA	NA	NA	NA	7	-21203,8589	42421,724	4585,30732	0
1114	-8,97566397	0,33138274	NA	NA	-0,0012458	0,28352203	NA	-1,05468211	-0,1361791	NA	NA	NA	0,35946641	NA	8	-21203,4786	42422,9652	4586,54847	0
1625	-7,52922856	NA	NA	NA	-0,00175017	0,32067571	NA	-0,7738147	-0,19560429	NA	NA	-0,00045111	0,35589058	NA	8	-21207,0173	42430,0425	4593,62579	0
1610	-7,76791863	0,37580763	NA	NA	-0,00127907	NA	NA	-0,7738147	-0,19560429	NA	NA	-0,00045111	0,35589058	NA	8	-21207,0804	42430,1687	4593,75198	0
596	-13,7669049	0,65178191	0,06462958	NA	NA	0,35119819	NA	-0,66565225	-0,15004816	NA	NA	-0,00048657	NA	NA	8	-21207,6668	42431,3416	4594,92491	0
588	-11,9654714	0,51367836	0,06255183	NA	-0,0013444	NA	NA	-0,66565225	-0,15004816	NA	NA	-0,00048657	NA	NA	8	-21207,8554	42431,7188	4595,30206	0
1562	-8,97566397	0,33138274	NA	NA	-0,0012458	0,28352203	NA	-0,97642003	NA	NA	NA	-0,00019403	0,33432079	NA	8	-21208,8661	42433,7402	4597,32343	0
1050	-8,97566397	0,33138274	NA	NA	-0,0012458	0,28352203	NA	-1,09054773	NA	NA	NA	NA	0,33971014	NA	7	-21210,3012	42434,6085	4598,19182	0
1618	-8,08714822	0,50972296	NA	NA	NA	0,2963004	NA	-0,7738147	-0,19560429	NA	NA	-0,00045111	0,35589058	NA	8	-21209,8882	42435,7843	4599,36755	0
1619	-11,2963041	NA	0,04958538	NA	NA	0,45107925	NA	-0,7738147	-0,19560429	NA	NA	-0,00045111	0,35589058	NA	8	-21210,2639	42436,5357	4600,11895	0
611	-9,52399274	NA	0,02470059	NA	NA	NA	-0,56950148	-0,66565225	-0,15004816	NA	NA	-0,00048657	NA	NA	7	-21211,4519	42436,9101	4600,49335	0
603	-11,3659105	NA	0,05744064	NA	-0,00198557	0,38445946	NA	-0,66565225	-0,15004816	NA	NA	-0,00048657	NA	NA	8	-21212,2774	42440,5629	4604,14614	0
1113	-7,52922856	NA	NA	NA	-0,00175017	0,32067571	NA	-1,05468211	-0,1361791	NA	NA	NA	0,35946641	NA	7	-21213,3041	42440,6144	4604,19769	0
1098	-7,76791863	0,37580763	NA	NA	-0,00127907	NA	NA	-1,05468211	-0,1361791	NA	NA	NA	0,35946641	NA	7	-21213,3672	42440,7406	4604,32388	0
532	-13,7669049	0,65178191	0,06462958	NA	NA	0,35119819	NA	-0,82885018	NA	NA	NA	-0,0002841	NA	NA	7	-21214,6778	42443,3617	4606,94502	0
524	-11,9654714	0,51367836	0,06255183	NA	-0,0013444	NA	NA	-0,82885018	NA	NA	NA	-0,0002841	NA	NA	7	-21214,8663	42443,7389	4607,32217	0
84	-13,7669049	0,65178191	0,06462958	NA	NA	0,35119819	NA	-0,96744453	-0,08610767	NA	NA	NA	NA	NA	7	-21214,9478	42443,9018	4607,48503	0
76	-11,9654714	0,51367836	0,06255183	NA	-0,0013444	NA	NA	-0,96744453	-0,08610767	NA	NA	NA	NA	NA	7	-21215,1364	42444,2789	4607,86217	0
1106	-8,08714822	0,50972296	NA	NA	NA	0,2963004	NA	-1,05468211	-0,1361791	NA	NA	NA	0,35946641	NA	7	-21216,175	42446,3562	4609,93945	0
1107	-11,2963041	NA	0,04958538	NA	NA	0,45107925	NA	-1,05468211	-0,1361791	NA	NA	NA	0,35946641	NA	7	-21216,5507	42447,1076	4610,69086	0
20	-13,7669049	0,65178191	0,06462958	NA	NA	0,35119819	NA	-0,99369134	NA	NA	NA	NA	NA	NA	6	-21217,7412	42447,4871	4611,07039	0
12	-11,9654714	0,51367836	0,06255183	NA	-0,0013444	NA	NA	-0,99369134	NA	NA	NA	NA	NA	NA	6	-21217,9298	42447,8643	4611,44753	0
547	-9,52399274	NA	0,02470059	NA	NA	NA	-0,56950148	-0,82885018	NA	NA	NA	-0,0002841	NA	NA	6	-21218,4629	42448,9304	4612,51368	0
609	-8,58193391	NA	NA	NA	NA	NA	-0,61393687	-0,66565225	-0,15004816	NA	NA	-0,00048657	NA	NA	6	-21218,468	42448,9407	4612,52397	0
99	-9,52399274	NA	0,02470059	NA	NA	NA	-0,56950148	-0,96744453	-0,08610767	NA	NA	NA	NA	NA	6	-21218,7329	42449,4704	4613,05369	0
1561	-7,52922856	NA	NA	NA	-0,00175017	0,32067571	NA	-0,97642003	NA	NA	NA	-0,00019403	0,33432079	NA	7	-21218,6916	42451,3894	4614,97265	0
1546	-7,76791863	0,37580763	NA	NA	-0,00127907	NA	NA	-0,97642003	NA	NA	NA	-0,00019403	0,33432079	NA	7	-21218,7547	42451,5156	4615,09885	0
1049	-7,52922856	NA	NA	NA	-0,00175017	0,32067571	NA	-1,09054773	NA	NA	NA	NA	0,33971014	NA	6	-21220,1267	42452,258	4615,84127	0
1034	-7,76791863	0,37580763	NA	NA	-0,00127907	NA	NA	-1,09054773	NA	NA	NA	NA	0,33971014	NA	6	-21220,1898	42452,3842	4615,96746	0
539	-11,3659105	NA	0,05744064	NA	-0,00198557	0,38445946	NA	-0,82885018	NA	NA	NA	-0,0002841	NA	NA	7	-21219,2884	42452,583	4616,16625	0
35	-9,52399274	NA	0,02470059	NA	NA	NA	-0,56950148	-0,99369134	NA	NA	NA	NA	NA	NA	5	-21221,5263	42453,056	4616,63927	0
91	-11,3659105	NA	0,05744064	NA	-0,00198557	0,38445946	NA	-0,96744453	-0,08610767	NA	NA	NA	NA	NA	7	-21219,5584	42453,123	4616,70625	0
1609	-6,18359995	NA	NA	NA	-0,00186372	NA	NA	-0,7738147	-0,19560429	NA	NA	-0,00045111	0,35589058	NA	7	-21219,9313	42453,8688	4617,45206	0

1602	-10,126072	0,56333515	NA	NA	NA	NA	NA	-0,7738147	-0,19560429	NA	NA	-0,00045111	0,35589058	NA	7	-21220,6837	42455,3737	4618,95696	0
27	-11,3659105	NA	0,05744064	NA	-0,00198557	0,38445946	NA	-0,99369134	NA	NA	NA	NA	NA	NA	6	-21222,3518	42456,7083	4620,29161	0
1554	-8,08714822	0,50972296	NA	NA	NA	0,2963004	NA	-0,97642003	NA	NA	NA	-0,00019403	0,33432079	NA	7	-21221,5625	42457,1311	4620,71442	0
1555	-11,2963041	NA	0,04958538	NA	NA	0,45107925	NA	-0,97642003	NA	NA	NA	-0,00019403	0,33432079	NA	7	-21221,9382	42457,8825	4621,46582	0
1042	-8,08714822	0,50972296	NA	NA	NA	0,2963004	NA	-1,09054773	NA	NA	NA	NA	0,33971014	NA	6	-21222,9976	42457,9998	4621,58303	0
1043	-11,2963041	NA	0,04958538	NA	NA	0,45107925	NA	-1,09054773	NA	NA	NA	NA	0,33971014	NA	6	-21223,3733	42458,7512	4622,33443	0
580	-12,0135881	0,70944129	0,06178489	NA	NA	NA	NA	-0,66565225	-0,15004816	NA	NA	-0,00048657	NA	NA	7	-21222,7538	42459,5138	4623,09712	0
545	-8,58193391	NA	NA	NA	NA	NA	-0,61393687	-0,82885018	NA	NA	NA	-0,0002841	NA	NA	5	-21225,479	42460,9612	4624,54452	0
97	-8,58193391	NA	NA	NA	NA	NA	-0,61393687	-0,96744453	-0,08610767	NA	NA	NA	NA	NA	5	-21225,749	42461,5012	4625,08453	0
1097	-6,18359995	NA	NA	NA	-0,00186372	NA	NA	-1,05468211	-0,1361791	NA	NA	NA	0,35946641	NA	6	-21226,2181	42464,4409	4628,02419	0
33	-8,58193391	NA	NA	NA	NA	NA	-0,61393687	-0,99369134	NA	NA	NA	NA	NA	NA	4	-21228,5424	42465,0871	4628,67033	0
1090	-10,126072	0,56333515	NA	NA	NA	NA	NA	-1,05468211	-0,1361791	NA	NA	NA	0,35946641	NA	6	-21226,9706	42465,9458	4629,52908	0
516	-12,0135881	0,70944129	0,06178489	NA	NA	NA	NA	-0,82885018	NA	NA	NA	-0,0002841	NA	NA	6	-21229,7648	42471,5342	4635,11745	0
68	-12,0135881	0,70944129	0,06178489	NA	NA	NA	NA	-0,96744453	-0,08610767	NA	NA	NA	NA	NA	6	-21230,0348	42472,0742	4635,65746	0
1545	-6,18359995	NA	NA	NA	-0,00186372	NA	NA	-0,97642003	NA	NA	NA	-0,00019403	0,33432079	NA	6	-21231,6056	42475,2159	4638,79915	0
587	-9,45532716	NA	0,05329757	NA	-0,00210061	NA	NA	-0,66565225	-0,15004816	NA	NA	-0,00048657	NA	NA	7	-21230,633	42475,2722	4638,85545	0
4	-12,0135881	0,70944129	0,06178489	NA	NA	NA	NA	-0,99369134	NA	NA	NA	NA	NA	NA	5	-21232,8282	42475,6598	4639,24304	0
1033	-6,18359995	NA	NA	NA	-0,00186372	NA	NA	-1,09054773	NA	NA	NA	NA	0,33971014	NA	5	-21233,0407	42476,0847	4639,66798	0
1538	-10,126072	0,56333515	NA	NA	NA	NA	NA	-0,97642003	NA	NA	NA	-0,00019403	0,33432079	NA	6	-21232,3581	42476,7208	4640,30405	0
1026	-10,126072	0,56333515	NA	NA	NA	NA	NA	-1,09054773	NA	NA	NA	NA	0,33971014	NA	5	-21233,7931	42477,5896	4641,17288	0
1603	-11,7728913	NA	0,04392487	NA	NA	NA	NA	-0,7738147	-0,19560429	NA	NA	-0,00045111	0,35589058	NA	7	-21235,8862	42485,7787	4649,36196	0
523	-9,45532716	NA	0,05329757	NA	-0,00210061	NA	NA	-0,82885018	NA	NA	NA	-0,0002841	NA	NA	6	-21237,6439	42487,2925	4650,87577	0
75	-9,45532716	NA	0,05329757	NA	-0,00210061	NA	NA	-0,96744453	-0,08610767	NA	NA	NA	NA	NA	6	-21237,9139	42487,8325	4651,41578	0
11	-9,45532716	NA	0,05329757	NA	-0,00210061	NA	NA	-0,99369134	NA	NA	NA	NA	NA	NA	5	-21240,7074	42491,4181	4655,00136	0
1617	-9,09446706	NA	NA	NA	NA	0,38722306	NA	-0,7738147	-0,19560429	NA	NA	-0,00045111	0,35589058	NA	7	-21241,0196	42496,0455	4659,62875	0
1091	-11,7728913	NA	0,04392487	NA	NA	NA	NA	-1,05468211	-0,1361791	NA	NA	NA	0,35946641	NA	6	-21242,1731	42496,3508	4659,93408	0
602	-8,97566397	0,33138274	NA	NA	-0,0012458	0,28352203	NA	-0,66565225	-0,15004816	NA	NA	-0,00048657	NA	NA	8	-21242,4872	42500,9823	4664,56558	0
1105	-9,09446706	NA	NA	NA	NA	0,38722306	NA	-1,05468211	-0,1361791	NA	NA	NA	0,35946641	NA	6	-21247,3065	42506,6176	4670,20088	0
1539	-11,7728913	NA	0,04392487	NA	NA	NA	NA	-0,97642003	NA	NA	NA	-0,00019403	0,33432079	NA	6	-21247,5606	42507,1258	4670,70905	0
1027	-11,7728913	NA	0,04392487	NA	NA	NA	NA	-1,09054773	NA	NA	NA	NA	0,33971014	NA	5	-21248,9956	42507,9946	4671,57788	0
538	-8,97566397	0,33138274	NA	NA	-0,0012458	0,28352203	NA	-0,82885018	NA	NA	NA	-0,0002841	NA	NA	7	-21249,4981	42513,0024	4676,58569	0
90	-8,97566397	0,33138274	NA	NA	-0,0012458	0,28352203	NA	-0,96744453	-0,08610767	NA	NA	NA	NA	NA	7	-21249,7681	42513,5424	4677,1257	0
26	-8,97566397	0,33138274	NA	NA	-0,0012458	0,28352203	NA	-0,99369134	NA	NA	NA	NA	NA	NA	6	-21252,5616	42517,1278	4680,71106	0
1553	-9,09446706	NA	NA	NA	NA	0,38722306	NA	-0,97642003	NA	NA	NA	-0,00019403	0,33432079	NA	6	-21252,694	42517,3926	4680,97584	0
1041	-9,09446706	NA	NA	NA	NA	0,38722306	NA	-1,09054773	NA	NA	NA	NA	0,33971014	NA	5	-21254,129	42518,2614	4681,84468	0
601	-7,52922856	NA	NA	NA	-0,00175017	0,32067571	NA	-0,66565225	-0,15004816	NA	NA	-0,00048657	NA	NA	7	-21252,3127	42518,6315	4682,2148	0
586	-7,76791863	0,37580763	NA	NA	-0,00127907	NA	NA	-0,66565225	-0,15004816	NA	NA	-0,00048657	NA	NA	7	-21252,3758	42518,7577	4682,34099	0
594	-8,08714822	0,50972296	NA	NA	NA	0,2963004	NA	-0,66565225	-0,15004816	NA	NA	-0,00048657	NA	NA	7	-21255,1835	42524,3733	4687,95656	0
595	-11,2963041	NA	0,04958538	NA	NA	0,45107925	NA	-0,66565225	-0,15004816	NA	NA	-0,00048657	NA	NA	7	-21255,5592	42525,1247	4688,70797	0
537	-7,52922856	NA	NA	NA	-0,00175017	0,32067571	NA	-0,82885018	NA	NA	NA	-0,0002841	NA	NA	6	-21259,3236	42530,6519	4694,23513	0
522	-7,76791863	0,37580763	NA	NA	-0,00127907	NA	NA	-0,82885018	NA	NA	NA	-0,0002841	NA	NA	6	-21259,3867	42530,778	4694,36132	0
89	-7,52922856	NA	NA	NA	-0,00175017	0,32067571	NA	-0,96744453	-0,08610767	NA	NA	NA	NA	NA	6	-21259,5936	42531,1919	4694,77514	0
74	-7,76791863	0,37580763	NA	NA	-0,00127907	NA	NA	-0,96744453	-0,08610767	NA	NA	NA	NA	NA	6	-21259,6567	42531,3181	4694,90133	0
1601	-15,360872	NA	NA	NA	NA	NA	NA	-0,7738147	-0,19560429	NA	NA	-0,00045111	0,35589058	NA	6	-21260,1618	42532,3282	4695,91148	0
25	-7,52922856	NA	NA	NA	-0,00175017	0,32067571	NA	-0,99369134	NA	NA	NA	NA	NA	NA	5	-21262,3871	42534,7774	4698,36072	0
10	-7,76791863	0,37580763	NA	NA	-0,00127907	NA	NA	-0,99369134	NA	NA	NA	NA	NA	NA	5	-21262,4502	42534,9036	4698,48691	0
530	-8,08714822	0,50972296	NA	NA	NA	0,2963004	NA	-0,82885018	NA	NA	NA	-0,0002841	NA	NA	6	-21262,1945	42536,3936	4699,97689	0
82	-8,08714822	0,50972296	NA	NA	NA	0,2963004	NA	-0,96744453	-0,08610767	NA	NA	NA	NA	NA	6	-21262,4645	42536,9336	4700,5169	0
531	-11,2963041	NA	0,04958538	NA	NA	0,45107925	NA	-0,82885018	NA	NA	NA	-0,0002841	NA	NA	6	-21262,5702	42537,145	4700,7283	0
83	-11,2963041	NA	0,04958538	NA	NA	0,45107925	NA	-0,96744453	-0,08610767	NA	NA	NA	NA	NA	6	-21262,8402	42537,685	4701,26831	0
18	-8,08714822	0,50972296	NA	NA	NA	0,2963004	NA	-0,99369134	NA	NA	NA	NA	NA	NA	5	-21265,2579	42540,5192	4704,10248	0
19	-11,2963041	NA	0,04958538	NA	NA	0,45107925	NA	-0,99369134	NA	NA	NA	NA	NA	NA	5	-21265,6336	42541,2706	4704,85389	0
585	-6,18359995	NA	NA	NA	-0,00186372	NA	NA	-0,66565225	-0,15004816	NA	NA	-0,00048657	NA	NA	6	-21265,2267	42542,458	4706,0413	0
1089	-15,360872	NA	NA	NA	NA	NA	NA	-1,05468211	-0,1361791	NA	NA	NA	0,35946641	NA	5	-21266,4486	42542,9005	4706,48382	0
578	-10,126072	0,56333515	NA	NA	NA	NA	NA	-0,66565225	-0,15004816	NA	NA	-0,00048657	NA	NA	6	-21265,9791	42543,9629	4707,54619	0
1537	-15,360872	NA	NA	NA	NA	NA	NA	-0,97642003	NA	NA	NA	-0,00019403	0,33432079	NA	5	-21271,8361	42553,6755	4717,25879	0
521	-6,18359995	NA	NA	NA	-0,00186372	NA	NA	-0,82885018	NA	NA	NA	-0,0002841	NA	NA	5	-21272,2376	42554,4786	4718,06185	0
1025	-15,360872	NA	NA	NA	NA	NA	NA	-1,09054773	NA	NA	NA	NA	0,33971014	NA	4	-21273,2712	42554,5446	4718,12784	0
73	-6,18359995	NA	NA	NA	-0,00186372	NA	NA	-0,96744453	-0,08610767	NA	NA	NA	NA	NA	5	-21272,5076	42555,0186	4718,60186	0
514	-10,126072	0,56333515	NA	NA	NA	NA	NA	-0,82885018	NA	NA	NA	-0,0002841	NA	NA	5	-21272,9901	42555,9835	4719,56674	0
66	-10,126072	0,56333515	NA	NA	NA	NA	NA	-0,96744453	-0,08610767	NA	NA	NA	NA	NA	5	-21273,2601	42556,5235	4720,10675	0

9	-6,18359995	NA	NA	NA	-0,00186372	NA	NA	-0,99369134	NA	NA	NA	NA	NA	NA	4	-21275,3011	42558,6044	4722,18766	0
2	-10,126072	0,56333515	NA	NA	NA	NA	NA	-0,99369134	NA	NA	NA	NA	NA	NA	4	-21276,0535	42560,1093	4723,69256	0
579	-11,7728913	NA	0,04392487	NA	NA	NA	NA	-0,66565225	-0,15004816	NA	NA	-0,00048657	NA	NA	6	-21281,1816	42574,3679	4737,9512	0
593	-9,09446706	NA	NA	NA	NA	0,38722306	NA	-0,66565225	-0,15004816	NA	NA	-0,00048657	NA	NA	6	-21286,315	42584,6347	4748,21799	0
515	-11,7728913	NA	0,04392487	NA	NA	NA	NA	-0,82885018	NA	NA	NA	-0,0002841	NA	NA	5	-21288,1926	42586,3885	4749,97175	0
67	-11,7728913	NA	0,04392487	NA	NA	NA	NA	-0,96744453	-0,08610767	NA	NA	NA	NA	NA	5	-21288,4626	42586,9285	4750,51175	0
3	-11,7728913	NA	0,04392487	NA	NA	NA	NA	-0,99369134	NA	NA	NA	NA	NA	NA	4	-21291,256	42590,5143	4754,09756	0
529	-9,09446706	NA	NA	NA	NA	0,38722306	NA	-0,82885018	NA	NA	NA	-0,0002841	NA	NA	5	-21293,326	42596,6553	4760,23854	0
81	-9,09446706	NA	NA	NA	NA	0,38722306	NA	-0,96744453	-0,08610767	NA	NA	NA	NA	NA	5	-21293,596	42597,1953	4760,77855	0
17	-9,09446706	NA	NA	NA	NA	0,38722306	NA	-0,99369134	NA	NA	NA	NA	NA	NA	4	-21296,3894	42600,7811	4764,36435	0
577	-15,360872	NA	NA	NA	NA	NA	NA	-0,66565225	-0,15004816	NA	NA	-0,00048657	NA	NA	5	-21305,4572	42620,9177	4784,50094	0
513	-15,360872	NA	NA	NA	NA	NA	NA	-0,82885018	NA	NA	NA	-0,0002841	NA	NA	4	-21312,4681	42632,9384	4796,52171	0
65	-15,360872	NA	NA	NA	NA	NA	NA	-0,96744453	-0,08610767	NA	NA	NA	NA	NA	4	-21312,7381	42633,4784	4797,06172	0
1	-15,360872	NA	NA	NA	NA	NA	NA	-0,99369134	NA	NA	NA	NA	NA	NA	3	-21315,5316	42637,0645	4800,64774	0
